# Phylogenetic Profiling of Mitochondrial Proteins and Integration Analysis of Bacterial Transcription Units Suggest Evolution of F1Fo ATP Synthase from Multiple Modules

**DOI:** 10.1007/s00239-017-9819-3

**Published:** 2017-11-24

**Authors:** Yulong Niu, Shayan Moghimyfiroozabad, Sepehr Safaie, Yi Yang, Elizabeth A. Jonas, Kambiz N. Alavian

**Affiliations:** 1 Division of Brain Sciences, Department of Medicine, Imperial College London, E508, Burlington Danes Hammersmith Hospital, DuCane Road, London, W12 0NN UK; 2 Key Lab of Bio-Resources and Eco-Environment of Ministry of Education, College of Life Sciences, Sichuan University, Chengdu, People’s Republic of China; 3 Department of Biology, The Bahá’í Institute for Higher Education (BIHE), Tehran, Iran; 4 Department of Mathematics and Computer Science, The Bahá’í Institute for Higher Education (BIHE), Tehran, Iran; 5 Department of Internal Medicine, Endocrinology, Yale University, New Haven, CT USA

**Keywords:** ATP synthase, Modular evolution, Phylogenetic profile, Transcription units, ATPase

## Abstract

ATP synthase is a complex universal enzyme responsible for ATP synthesis across all kingdoms of life. The F-type ATP synthase has been suggested to have evolved from two functionally independent, catalytic (F1) and membrane bound (Fo), ancestral modules. While the modular evolution of the synthase is supported by studies indicating independent assembly of the two subunits, the presence of intermediate assembly products suggests a more complex evolutionary process. We analyzed the phylogenetic profiles of the human mitochondrial proteins and bacterial transcription units to gain additional insight into the evolution of the F-type ATP synthase complex. In this study, we report the presence of intermediary modules based on the phylogenetic profiles of the human mitochondrial proteins. The two main intermediary modules comprise the α_3_β_3_ hexamer in the F1 and the c-subunit ring in the Fo. A comprehensive analysis of bacterial transcription units of F1Fo ATP synthase revealed that while a long and constant order of F1Fo ATP synthase genes exists in a majority of bacterial genomes, highly conserved combinations of separate transcription units are present among certain bacterial classes and phyla. Based on our findings, we propose a model that includes the involvement of multiple modules in the evolution of F1Fo ATP synthase. The central and peripheral stalk subunits provide a link for the integration of the F1/Fo modules.

**Electronic supplementary material:**

The online version of this article (10.1007/s00239-017-9819-3) contains supplementary material, which is available to authorized users.

## Introduction

Oxidative phosphorylation is the main source of ATP production in aerobic organisms. The redox reactions of the last step of aerobic respiration are performed by a series of evolutionary conserved protein complexes, or the electron transport chain (ETC). The main function of ETC is to produce a proton gradient across the cellular or mitochondrial membranes. ATP synthase utilizes this gradient to generate ATP (Mitchell 1961). Three types of ATP synthase have been identified: the A-, V-, and F-type ATPases. The A-type (A1Ao) ATPase exists in the archaea and a small number of bacteria (Ballmoos et al. 2008; Lewalter and Muller 2006). The V-type (V1Vo) ATPase exists in the eukaryotic cytoplasmic membranes (vacuoles) (Beyenbach and Wieczorek 2006). The F-type (F1Fo) ATP synthase is found in the mitochondria and chloroplasts of most eukaryotes and in the bacterial cells. The F1Fo ATP synthase has a range of structural features that distinguish them from the A- and V-type ATPases (Muller and Gruber 2003). A novel subfamily of F-type ATPases, named as “N-ATPase,” were identified in bacteria and some archaeal species (*Methanosarcina acetivorans* and *M. barkeri*). The N-type ATPase is thought to be acquired by a lateral gene transfer (Sumi et al. 1997; Dibrova et al. 2010). A distinctive property of the N-ATPase is believed to be its sodium translocating function, based on the presence of a full set of sodium-binding residues on their membrane-bound c subunits (Dibrova et al. 2010). The three ATPase types share a common ancestor, which has undergone structural and functional changes. The major subunits of A-type ATPases share about 50 and 25% sequence identity with V-type and F-type ATPases, respectively (Hilario and Gogarten 1998). Structurally, the A-, V-, and F-ATPases have three components: (i) a membrane-bound subunit, Ao/Vo/Fo, that includes the proton translocator, (ii) a connecting central stalk subunit, (iii) the catalytic subunit, A1/V1/F1 and (iv) the peripheral stalks, which are the least conserved component among the ATPases subtypes (Wang and Oster 1998; Gruber et al. 2001; Lee et al. 2010).

The bacterial F-type ATPase comprises 8 subunits (α_3_, β_3_, γ, δ, ε, a, b_2_, and c_9−14_) while the more complex mitochondrial F-ATPase consists of up to 17 subunits (α_3_, β_3_, γ, δ, ε, a, b_2_, c_8−14_, d, e, f, g, A_6_L, F_6_, I, K, and OSCP) (Buchanan and Walker 1996; Stock et al. 2000). The F1 complex is composed of an α and β hexameric subunit which includes the ATP catalytic site (Stock et al. 2000). The membrane-bound Fo component comprises a homomeric c-subunit ring and the ‘a’ subunit containing the proton translocator plus the b subunit (Lau et al. 2008; Rubinstein et al. 2003). The F1 and Fo components are connected by a central and a peripheral stalk. The mitochondrial F1Fo central stalk is formed by an elongated γ subunit and two regulator factors δ and ε subunits. Though universal, major differences exist between the exact subunit construction of F1Fo ATP synthase of prokaryotic vs. eukaryotic species. The bacterial peripheral stalk, for instance, is composed of δ and b_2_ subunits while the bovine version of the peripheral stalk is formed by Oligomycin sensitivity conferral protein (OSCP), F_6_, b, and d subunits, essential for the ATP synthase activity (Collinson et al. 1996). The N-terminal part of OSCP in mammals can interact directly with α subunit, and acts as a stator to prevent F1 hexamer rotation with the central rotary element (Rubinstein et al. 2003; Carbajo et al. 2005). The transfer of protons through Fo subunits causes the rotation of membrane-bound c-subunits and of the connected central stalk. ATP synthesis is achieved through a conformational change in the F1 as the γ subunit of the stalk rotates (Noji et al. 1997; Gibbons et al. 2000). The F1 hexamer can also hydrolyze ATP, which results in reversal of the direction of rotation of γ and the c-subunit ring. This process drives the protons against their concentration gradient, from the matrix to the intermembrane space of the mitochondria (Boyer 1997; Xing et al. 2005).

The two structurally and functionally distinct parts of the ATP synthase, F1 and Fo, have been suggested to comprise evolutionary modules (Walker 1998; Mulkidjanian et al. 2007). This view was supported by the evidence that the genes encoding F1 and Fo subunits are found gathered into separate transcription units in bacteria like *Rhodospirillum rubrum* (Falk and Walker 1988). Mulkidjanian et al. suggested an interesting evolutionary model that F1Fo ATP synthase originated from membrane protein translocases, which themselves evolved from RNA translocases. Following this model, the central stalk was originally the translocated protein trapped in the channel of the membrane translocase, and the peripheral stalk was formed to provide an elastic connection between the ancestral hexamer and the c-subunit ring (Mulkidjanian et al. 2007). A more recent study suggested that the assembly of yeast mitochondrial F1Fo ATP synthase is not a single linear process (as previously thought) but involves two separate regulated pathways (Rak et al. 2011).

In the present study, we generated the phylogenetic profiles of human mitochondrially targeted proteins by integrating the whole genome information of 759 different prokaryotic and eukaryotic species. We show for the first time that the F1-Fo ATP synthase subunits were consistently gathered into three major groups: F1, c-rings, and the remaining subunits of the Fo component. By analysis of transcription units from more than 2000 bacterial species, we also concluded (i) a constant order and conserved combination patterns of F1Fo ATP synthase subunits in bacterial genomes, (ii) F1 (or F1 plus b subunit) and Fo components were significantly gathered into different transcription units, and (iii) α/β and c-ring, respectively, in F1 and Fo components could be independent modules. Based on these results, we postulated that the F1Fo ATP synthase evolved from at least four modules, the α/β and c-ring and the remainder of the F1 and separately the Fo components. The central and peripheral stalk subunits may provide the evolutionary linkage for the combination of these different modules.

## Materials and Methods

### Phylogenetic Profiling of Human Mitochondria Proteins

Genomic sequences and annotation information were retrieved from the KEGG database (retrieved at 02/15/2014) (Kanehisa et al. 2006), and BLASTP (Camacho et al. 2009) was used for comparisons of protein sequences between human and the rest of 758 species. We constructed the phylogenetic profiles of each human protein sequence according the BLASTP E-value, and the “presence” and “absence” were recorded by using the E-value threshold 0.001.

We manually checked the homologs of F1Fo ATP synthase, 93.7% (4191/4473) were homologous subunits of F-type ATP synthase. For the total 213 homologous proteins (13 in Eukaryota, 36 in Bacteria, and 164 in Archaea) of V/A-type ATP synthase, they were either A subunit or B subunit which were homologs of α and β subunits, respectively. Bedsides, these detected A/B subunits were from species with complete or partial lack of F-type ATP synthase, demonstrating that we have detected accurate homologs of human F1Fo ATP synthase without messing up with V/A-type ATPase paralogs. We proposed six independent methods to measure the distance between pairs of genes (Glazko and Mushegian 2004; Kensche et al. 2008), namely, Euclidean, Manhattan, Jaccard, tau, mutual information, and Pearson absolute anti-correlation distance. A total of 1006 human mitochondrial proteins were chosen from MitoCarta database (Pagliarini et al. 2008) for hierarchical cluster analysis, and grouped number was set as 5, 10, 15, 20, 25, 30, 35, 40, 45, and 50. Moreover, PCA was applied for the group analysis of mitochondrial F1Fo ATP synthase proteins.

### Hierarchical Cluster Methods Used in Phylogenetic Profile Analysis

To get robust cluster analysis in phylogenetic profiling analysis, we applied a variety of hierarchical cluster methods (Glazko and Mushegian 2004; Kensche et al. 2008). Each pair of human mitochondrial proteins was denoted as two vectors containing either 0 (“absence”) and 1 (“presence”), for example $X,Y\in \{\text{0,1}{\}}^{n}$.

The ${L}_{p}$-norms is

1 $${d_P}={\left[ {\sum\limits_{{i=1}}^{n} {{{\left| {{x_i} - {y_i}} \right|}^p}} } \right]^{1/p}}$$

where $n$ is the total number of species used in phylogenetic profiling; $p=1$ is the Manhattan distance; and $p=2$ is the Euclidean distance.

Jaccard distance is defined from co-occurrence of presences

2 $${d_J}=1 - \frac{{{\text{Card}}\left( {{x_i}=1 \cap {y_i}=1} \right)}}{{{\text{Card}}\left( {{x_i}=1 \cup {y_i}=1} \right)}}.$$

Mutual information is used to measure distance (Korber et al. 1993; Huynen et al. 2000),

3 $$I\left( {X;Y} \right)=\sum\limits_{{x \in \left\{ {1,0} \right\}}} {\sum\limits_{{y \in \left\{ {1,0} \right\}}} {p\left( {x,y} \right)\log \left( {\frac{{p\left( {x,y} \right)}}{{p\left( x \right)p\left( y \right)}}} \right)} },$$

where $p \left(x\right)$ is the probability that a symbol (here 0 or 1) appears in the gene vector $X$regardless of the probability of a symbol combination appearing in gene vectors $X$ and *Y*. The mutual information distance is defined as ${d}_{M}=1-I (X;Y)$.

The Kendall tau rank distance is defined as

4 $$K\left( {\tau 1,\tau 2} \right)=\sum\limits_{{\{ i,j\} \in P}} {{{\overline {K}}_{i,j}}\left( {\tau 1,\tau 2} \right)},$$

where $\tau 1$ and $\tau 2$ are the rankings of elements in $X$ and $Y$; $P$ is the paired combination in $\tau 1$ and $\tau 2$. If $i$ and $j$ are in the same order, ${\overline{K}}_{i,j}=0$; otherwise, ${\overline{K}}_{i,j}=1$.

We used the Pearson correlation coefficient

5 $${\text{cor}}\left( {X,Y} \right)=\frac{{\sum\nolimits_{{i=1}}^{n} {\left( {{x_i} - \overline {X}} \right)\left( {{y_i} - \overline {Y}} \right)} }}{{\left( {n - 1} \right){S_X}{S_Y}}}$$

where $\overline{X}$ and $\overline{Y}$ are the sample means of $X$ and *Y*, and ${S}_{X}$ and ${S}_{Y}$ are the sample standard deviations of $X$ and $Y$. The Pearson absolute anti-correlation distance is ${d}_{P}=1-\left|\text{c}\text{o}\text{r} \left(X,Y\right)\right|$.

We applied the Euclidean distance measurement to generate the distance tree (Fig. 2a–c) with phylogenetic profiles of human mitochondria proteins, in which BLASTP E-value 0.001 was used as the threshold. We then used the threshold 0.1, 0.01, and 0.0001 to validate the distance tree (Suppl. Fig. 2). The group number of PCA was set as 2, 3 (Suppl. Fig. 3), and 4 (Fig. 2d) to cluster the mitochondria F1Fo ATP synthase subunits.

### Identification of Evolutionarily Conserved Modules

We applied the clustering by inferred models of evolution (CLIME) algorithm with default phylogenetic tree (Bick et al. 2012) and parameters to identify evolutionarily conserved modules (ECMs) of F1Fo ATP synthase subunits (Li et al. 2014). The input phylogenetic profile using human as the reference was prepared as described in CLIME, and the same threshold (BLASTP E-value < 0.001) was set. The single “Prokaryotes” was generated from 696 prokaryotic species, in which the “presence” was assigned if more 5% homologs were detected.

### Prediction of Bacterial Transcription Units

Orthology of bacterial F1Fo ATP synthase genes was provided in KEGG Orthology database (http://www.genome.jp/kegg/ko.html). We included 2248 different bacterial strains, and retrieved their transcription unit information from the BioCyc database (http://biocyc.org/).

Bacterial transcription unit data of F1Fo ATP synthase genes were retrieved from BioCyc (retrieved on 04/10/2014), and most of them were predicted by computational methods (Romero and Karp 2004). We improved the prediction method by combining the continuous transcription units if they were in the same transcription direction and had no interrupted gene between them (Fig. 3b and Suppl. File 1). We at the same time reserved the raw transcription unit data (Suppl. File 2) and used the parallel analysis procedure (Suppl. Fig. 4).

### Bacterial Transcription Unit Analysis

The frequency of transcription unit modules presented in Fig. 3b was generated by mapping the transcription unit data to the most common gene order “ε-β-γ-α-δ-b-c-a” without considering the transcription direction. We defined the standard cTU as “ε-β-γ-α-δ-b-c-a” because this long ordered transcription unit was observed in most species in which all the F1Fo ATP synthase genes were maintained together in the same transcription units. In this process, we merged the homology of the synthase genes if they were continuously arranged in the same transcription unit; for example, we transferred the transcription unit “ε-β-γ-α-δ-b-b′” to “ε-β-γ-α-δ-b,” as the gene order was the most important feature with which we were concerned.

We used the upper confidence intervals of the binomial distribution as the cutoff line (Fig. 3b and Suppl. Fig. 4)

6 $$C{I_{{\text{up}}}}=p+{Z_\alpha }\sqrt {\frac{{p(1 - p)}}{N}},$$

where $p$ is the possibility to choose one pair of cut points as denoted in Fig. 3a, here $p=1/C\left(9, 2\right)=1/36$, ${Z}_{\alpha }$ is the normal distribution quantile with $\alpha =0.05$, and $N$ is the total number of transcription units.

The δ/b Rosetta Stone was defined by appearance of the b and δ subunit motifs in the same protein sequence. The KEGG SSDB (Sequence Similarity DataBase, http://www.kegg.jp/kegg/ssdb/) was used to validate the δ/b Rosetta Stone (Suppl. Fig. 5).

### Phylogenetic Tree

The 16S rDNA sequences were retrieved and used to reconstruct phylogenetic tree of bacteria species in transcription unit analysis. The bacterial class/phylum tree was constructed from concatenating 16S rDNA sequences in each class or phylum.

Alignments were generated using the MAFFT program (Katoh and Standley 2013), and the phylogenetic tree was constructed by setting the maximum likelihood in RAxML (Randomized Axelerated Maximum Likelihood) program (Stamatakis 2014) with default parameters. The transcription units were combined with the phylogenetic tree visualized in the iTOL (Interactive Tree Of Life) tool (Letunic and Bork 2011). The root of phylogenetic trees was determined at *Thermotogae* as was suggested by Battistuzzi and Hedges (2009). In the phylogenetic trees showing combination of transcription units and chimera fusions in bacterial phyla or classes, *Fervidobacterium nodosum* was set as the outer group.

### Statistical Analysis

All the statistical analysis was conducted using the open-source R Project (http://www.r-project.org/).

## Results

### Construction of the Phylogenetic Profile of Mitochondrial Proteins

Based on the assumption that functionally related proteins are likely to evolve in a correlated manner, phylogenetic profiling has been used to predict functional and structural interactions between proteins (Pellegrini et al. 1999). To perform a comprehensive phylogenetic profile analysis across a wide range of eukaryotic and prokaryotic taxa, we considered all fully sequenced genomes and included at least one species from every class according to the taxonomy information provided in KEGG database (Kanehisa et al. 2006). The final list included 759 species including 82 Archaeal, 614 Bacterial, and 63 Eukaryotic species (Suppl. Table 1). The phylogenetic profiles were built for a total of 1006 human genes including the nuclear and mitochondrial protein-coding sequences for the mitochondrial proteins annotated by the MitoCarta database (Pagliarini et al. 2008) (Suppl. Table 2).

### Subunits of Mitochondrial F1Fo ATP Synthase are Clustered in Distinct Groups

**Fig. 1:**
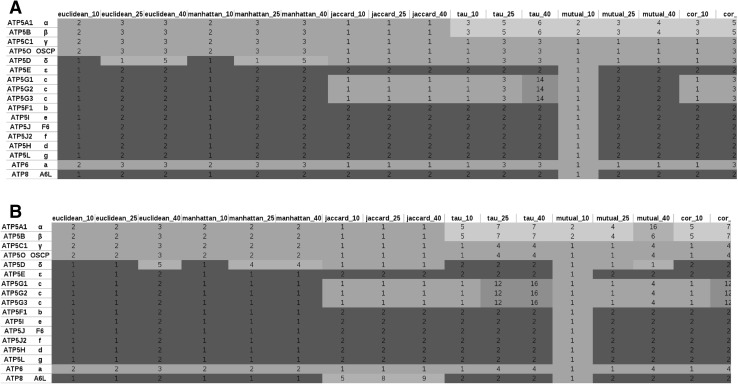
Groups of mitochondrial F1Fo ATP synthase from mitochondrial proteins. Hierarchical clusters of phylogenetic profiles using 758 (**a**) or 490 randomly chosen (**b**) species with independent distance measurements: Euclidean, Manhattan, Jaccard, Kendall tau, mutual information, and Pearson absolute anti-correlation distance. Group number is set to 10, 25, or 40, and distinct groups are marked with different colors. (Color figure online)

To accurately assess the evolutionary correlation of mitochondrial F1Fo ATP synthase subunits, we chose a two-step approach. First, we performed a hierarchical cluster analysis to examine whether the mitochondrial proteins would be grouped into different categories. By this analysis, the proteins that clustered into the same group need to be gained and lost together, based on the phylogenetic profiling data. Then, to get robust results, we applied 6 independent methods, Euclidean, Manhattan, Jaccard, Kendall tau, mutual information, and Pearson absolute anti-correlation distance, to calculate the distance within each gene pair. Using each method, we generated hierarchical groups of 5, to 50 (in increments of 5) mitochondrial protein-coding genes (Suppl. Table 3). Using the Euclidean distance and Manhattan distance methods, we revealed two distinct clusters of ATP synthase subunits. The catalytic subunits of the synthase, α (ATP5A1) and β (ATP5B), the central and lateral stalk subunits, γ (ATP5C1) and OSCP (ATP5O) (respectively), in addition to the a subunit of Fo clustered together (red). The rest of the subunits clustered into a second group (green). The use of Jaccard method placed the three homologs of the c subunit (ATP5G1, ATP5G2, and ATP5G3) in the first cluster (Fig. 1a).

To test the specificity of group members, we gradually increased the number of hierarchical groups from 5 to 50 (Suppl. Table 3). Interestingly, we detected two additional subgroups which had not been reported previously. The first subgroup consists of the α_3_β_3_ hexamer (purple), which contained the catalytic site of the F1 subunit. This appeared as an independent subgroup even when a small group number (10) was set with the methods of Kendall tau, mutual information, and Pearson absolute anti-correlation distance. Upon increasing the group number to 40, another subgroup containing the three homologs (ATP5G1/G2/G3) of the mitochondrial inner membrane-bound c-subunit appeared as a separately evolved group from the other Fo components according to Kendall tau and Pearson absolute anti-correlation distance methods (Fig. 1a, yellow).

To verify that this pattern did not appear as a result of using the specific species list chosen for the profiling analysis, we randomly selected 490 species including 50 Archaeal, 400 Bacterial, and 40 Eukaryotic species (Suppl. Table 4). The same analysis procedures, cluster methods, and hierarchical group numbers were applied to the newly generated phylogenetic profiles (Fig. 1b). These results strongly confirmed placement of the synthase subunits into four distinct clusters (Fig. 1b and Suppl. Table 5).

### The Phylogenetic Profiles of the Subunits Within Each Evolutionary Module are Tightly Correlated

**Fig. 2:**
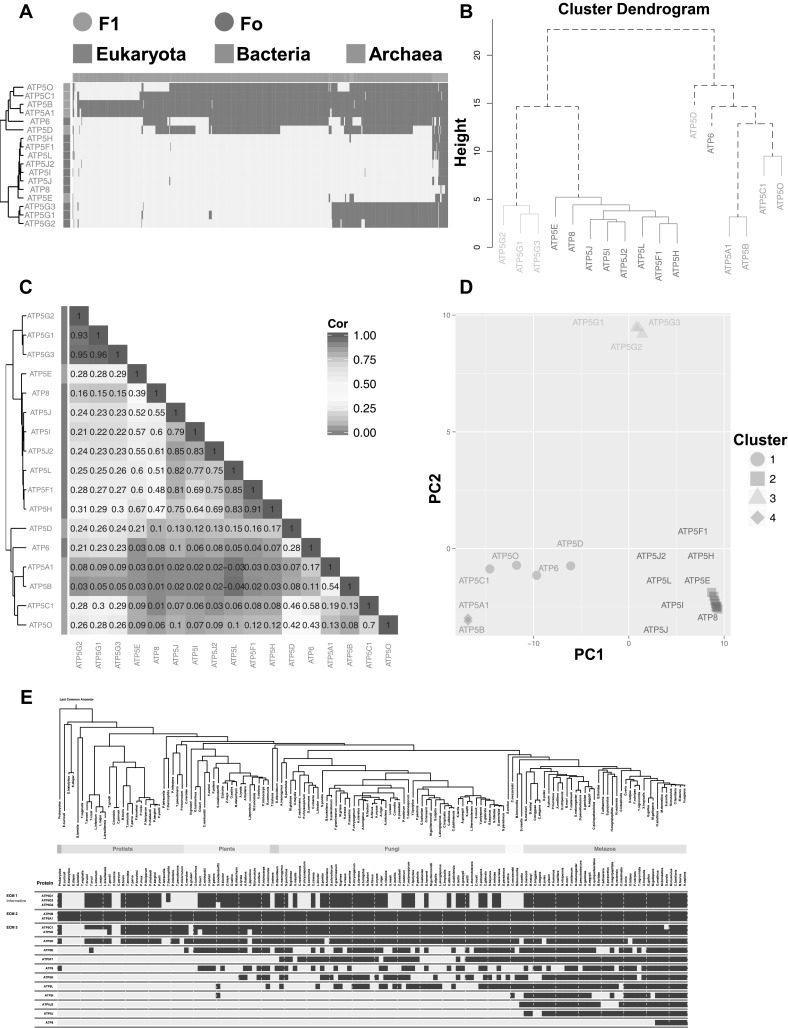
Cluster analysis for human mitochondrial F1Fo ATP synthase subunits. **a** The phylogenetic profile for human 17 subunits of the F1Fo ATP synthase clustered both by species (columns) and genes (rows). The top color bar indicates the three domains of life, and the left color bar represents the corresponding F1 and Fo subunits arranged in a cluster dendrogram. The blue and gray bars in the phylogenetic profiles indicate presence or absence in a given species, respectively. **b** Distance tree of mitochondrial F1Fo ATP synthase subunits generated by Euclidean distance with the group number set to 6. Different colors are used to indicate identified clusters. **c** Correlation matrix of mitochondrial F1Fo ATP synthase subunits. The Pearson correlation coefficients of paired genes are marked in numbers and color-coded from blue to red. The left cluster dendrogram indicates the Euclidean distance between subunits of F1 and Fo marked with red and green, respectively. **d** Principal component analysis (PCA) plot of the 17 subunits of F1Fo ATP synthases with a cluster number of 4. Identified groups are shown with different colors and shapes. **e** The evolutionarily conserved modules (ECMs) of human F1Fo ATP synthase. Blue and gray blocks indicate present and absent homologs, respectively. (Color figure online)

The finding that the α_3_β_3_ hexamer and the c-subunit homologs comprised distinct evolutionary modules prompted further investigation into the correlation of the genes within the evolutionary modules of the mitochondrial ATP synthase. We generated a distance tree and a correlation matrix from the evolutionary profile data of genes coding for 17 synthase subunits (Fig. 2a). The F1 subunits α and β (purple), γ and OSCP (red), appeared in the same cluster (Fig. 2b). The appearance of two different subgroups may be due to the fact that α/β or homologous A/B subunits in V/A-type ATPase subunits are present in all three domains of life, whereas γ and OSCP subunits exist in most eukaryotic and bacterial species but no significant homologs found in *Archaea* (Fig. 2a). The pairing of the subunits was confirmed by the high Pearson correlation coefficient value (0.54 for α/β and 0.70 for γ/OSCP) (Fig. 2c). In species harboring entire F1Fo ATP synthases, we detected the corresponding homologs of α and β subunits (Suppl. Fig. 1A). At the same threshold (BLASTP E-value smaller than 0.001), in organisms (mostly in *Archaea* and some *Eukaryota* and *Bacteria*) only possessing V/A-type ATPase or partial F-type ATPase, in the absence of α and/or β, the corresponding A/B subunits were chosen as the homologs (Suppl. Fig. 1B). The widely distributed homologs of α/β made them distinct from the rest of the subunits of the F1Fo ATP synthase. This subgroup was therefore considered as a separate evolutionary module.

Although all Fo subunits, c (ATP5G1/G2/G3), d (ATP5H), b (ATP5F1), g (ATP5L), f (ATP5J2), e (ATP5I), F_6_ (ATP5J), and A_6_L (ATP8) belonged to the same clade (Fig. 2b), the c-ring subunits (yellow) had a distinct pattern of phylogenetic profiles and high correlation coefficients (> 0.93 for each pair), which confirmed their existence as a separate subgroup (Fig. 2b, c). Changing the threshold of BLAST E-value (from 0.1 to 0.0001) for generating the distance trees did not alter the results and the α/β, γ/OSCP, and c-ring subgroups were consistently observed (Suppl. Fig. 2). The use of principal component analysis (PCA) highlighted the presence of four evolutionary modules: one comprised the c-ring subunits (yellow); the second included the α and β subunits (purple); the third and fourth included the known F1 (red) and Fo (green) modules each comprised the rest of the respective subunits of each compartment (Fig. 2d and Suppl. Fig. 3).

To further confirm these cluster modules, we applied clustering by inferred models of evolution (CLIME) method (Li et al. 2014) to the phylogenetic profiles. By combining the phylogenetic tree and profiles, CLIME can identify evolutionarily conserved modules (ECMs) from protein complexes and pathways. Three ECMs, α/β, OSCP/γ, and the c-rings, were detected (Fig. 2e), confirming the presence of multiple evolutionary modules for the F1Fo ATP synthase. We noticed that δ (ATP5D) and ε (ATP5E) subunits, components of central stalk, were clustered into separate groups (Fig. 2b), mainly due to their different gain and loss patterns. The absence of ε subunit in many protists (Fig. 2e) may have resulted from the incomplete genes annotation or different evolutionary scenarios for δ and ε subunit (Koumandou and Kossida 2014).

### The Bacterial F1/Fo Subunits are Gathered into Different Modules

The bacterial F1Fo ATP synthase comprises 8 subunits. Subunits α, β, γ, δ, and ε belong to the F1 component, and the Fo component consists of the a, b (and b′), and c subunits (Deckers-Hebestreit and Altendorf 1996). While the genes coding for all eight subunits are arranged in the same operon and transcribed together in some bacteria, including *Escherichia coli* (Nielsen et al. 1984), the genes coding for the F1 and Fo components are clustered into separate operons in the genomes of other bacterial species including *R. rubrum* (Falk and Walker 1988). This raised the possibility that the F1Fo ATP synthase has evolved from two different modules (Falk and Walker 1988). To further investigate this premise, we performed a comprehensive analysis of transcription units containing the genes coding for bacterial F1Fo ATP synthase. For this analysis, we included a dataset of putative transcription units from 2248 strains or substrains of bacteria, covering 568 different genera, predicted by computational methods (Suppl. File 1) (Romero and Karp 2004).

As anticipated, a large percentage of species contained all eight subunits in the same transcription unit (cTU, complete transcription unit). Interestingly, the observation that ATP synthase genes were divided into different transcription units (iTU, incomplete transcription unit) was not rare. In addition to the iTUs reported in *Rhodospirillum* and *Synechococcus*, the genera *Rickettsia* and *Methylobacterium* contained “ε-β-γ-α-δ” and “b-c-a” iTUs, whereas the “ε-β-γ-α-δ-b,” “c,” and “a” iTUs were found in the genus *Helicobacter* (Suppl. File 1 and Suppl. Table 6). With a few exceptions, genes in most cTUs follow a constant order as “ε-β-γ-α-δ-b-c-a,” confirmed by experimental data from *E. coli* (Gay and Walker 1981), *Bacillus subtilis* (Santana et al. 1994), and *Clostridium pasteurianum* (Das and Ljungdahl 2003). The order of genes in iTUs, according to our data and previous studies (McCarn et al. 1988; Borghese et al. 1998), was similar to the order of genes in cTUs, suggesting that multiple distinct evolutionary modules, comprised a combination of ordered iTUs, exist within the bacterial F1Fo ATP synthase.

**Fig. 3:**
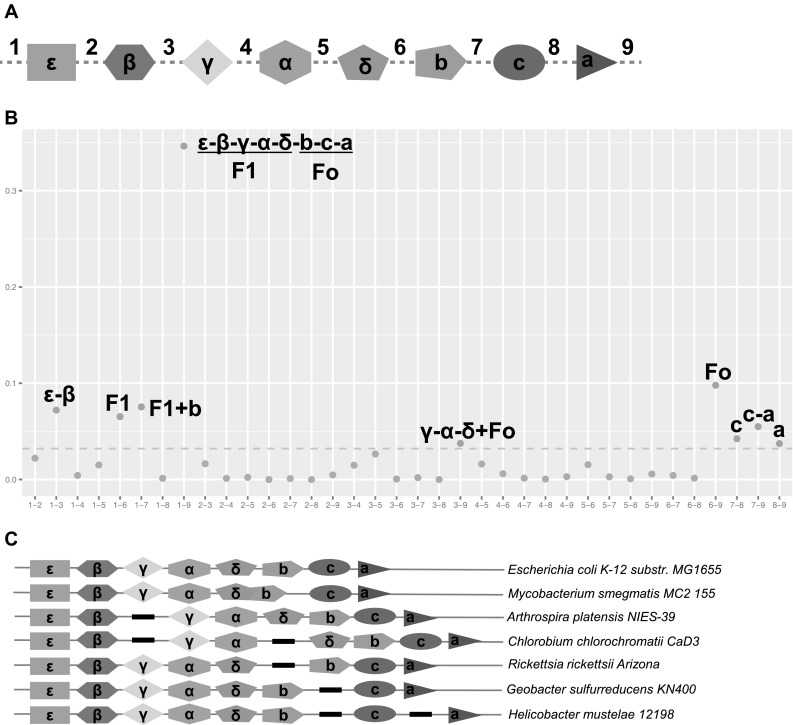
Transcription unit modules of bacterial F1Fo ATP synthase. **a** The standard transcription units with cut points. **b** The frequency of transcription unit modules mapped to the standard one in A. Red dots denote that transcription unit modules are significantly higher than the upper confidence interval. The green dots are the modules which did not reach statistical significance. The dashed gray line marks the upper confidence interval (0.03) with the threshold 0.05. **c** Representative patterns of transcription units detected in bacterial genomes. The F1Fo ATP synthase genes are marked with different colors and shapes. The schematic patterns do not indicate the length of the genome or the actual transcription direction. (Color figure online)

To test this hypothesis, we calculated the frequency of iTUs in various bacterial genomes. We used the most common gene order “ε-β-γ-α-δ-b-c-a” (in either direction) as the standard order, and then mapped each iTU and cTU accordingly (Fig. 3a). We neglected the transcriptional direction, which meant the “ε-β-γ-α-δ” and the “δ-α-γ-β-ε” were treated as the same iTU. As expected, we observed that standard order had the highest (34.6%) frequency, and the Fo module (“b-c-a”) and the F1 module (“ε-β-γ-α-δ”) had the second (9.8%) and the fifth (6.5%) highest frequencies, respectively. We identified three novel combinations, i.e., “ε-β” plus “γ-α” plus “δ-b-c-a,” “ε-β-γ-α-δ-b,” plus “c-a,” and “ε-β-γ-α-δ-b” plus “c” plus “a” (Fig 3b, c) in addition to the previously reported combination patters (“ε-β-γ-α-δ” plus “b-c-a” and “ε-β” plus “γ-α-δ-b-c-a”). The frequencies of nine modules were significantly higher than the upper confidence interval (0.03 with the threshold of 0.05) (Fig. 3b), suggesting that the modules are highly unlikely to be chosen randomly in bacterial genomes.

Prediction of the bacterial transcription units based on the BioCyc database (http://biocyc.org/) validated most of the identified modules, comprising the F1Fo ATP synthase subunits (Suppl. Fig. 4 and Suppl. File 2). Interestingly, a Rosetta Stone protein (a fusion protein generated by the combination of separate proteins) of δ and b subunits was observed in *Gordonia* genus and some *Mycobacterium* species (Fig. 3c and Suppl. Fig. 5). The identified δ/b Rosetta Stone was located in the middle of transcription units, between the α and another single b subunit (Suppl. Table 7). The δ/b Rosetta Stone is likely to result from a fusion event in the transcription unit, in which the δ and the two heterologous b subunit genes were arranged in a sequential order. Since iTUs such as “γ-α-δ-b-b′-c-a” and “ε-β-γ-α-δ-b-b′” were observed, it is likely that this fusional combination was a common way to generate multiple-domain proteins (Pasek et al. 2006; Wang and Caetano-Anolles 2009). The δ/b Rosetta Stone was unlikely to be directly generated by integrating the “b′-b-c-a” and “ε-β-γ-α-δ,” since these two kinds of iTUs were simultaneously found in some bacterial genomes.

Our transcription unit analysis revealed that the bacterial F1Fo ATP synthase genes were included in the same transcription unit in many bacterial genomes and they were arranged in a highly conserved order. The proximity of the F1Fo ATP synthase genes in transcription units is a dominant trait among various bacterial genomes, most likely to have been caused by positive selection. Further analysis of the diverse pool of bacterial species revealed at least five combinations of transcription units which contained parts of ATP synthase genes, arranged in a similar order to that of the standard cTU (Fig. 3c).

### Transcription Units of F1Fo ATP Synthase Genes Were Conserved Among Bacterial Classes

The formation of transcription units containing multiple genes or operons has been ascribed to the process of horizontal gene transfer (HGT), in which genes close to each other are transferred together from one species to another. These genes may be functionally unrelated at first, but random genome rearrangements would move them closer and they would then transfer together. An operon then is formed after the deletion of intervening DNA sequences between adjacent genes (Lawrence and Roth 1996). An alternative model posits that operon formation is driven by co-regulation of genes instead of HGT. According to this model, individually transcribed genes with similar expression and regulation patterns integrate into one transcription unit to reduce the amount of regulatory information (Price et al. 2005).

**Fig. 4:**
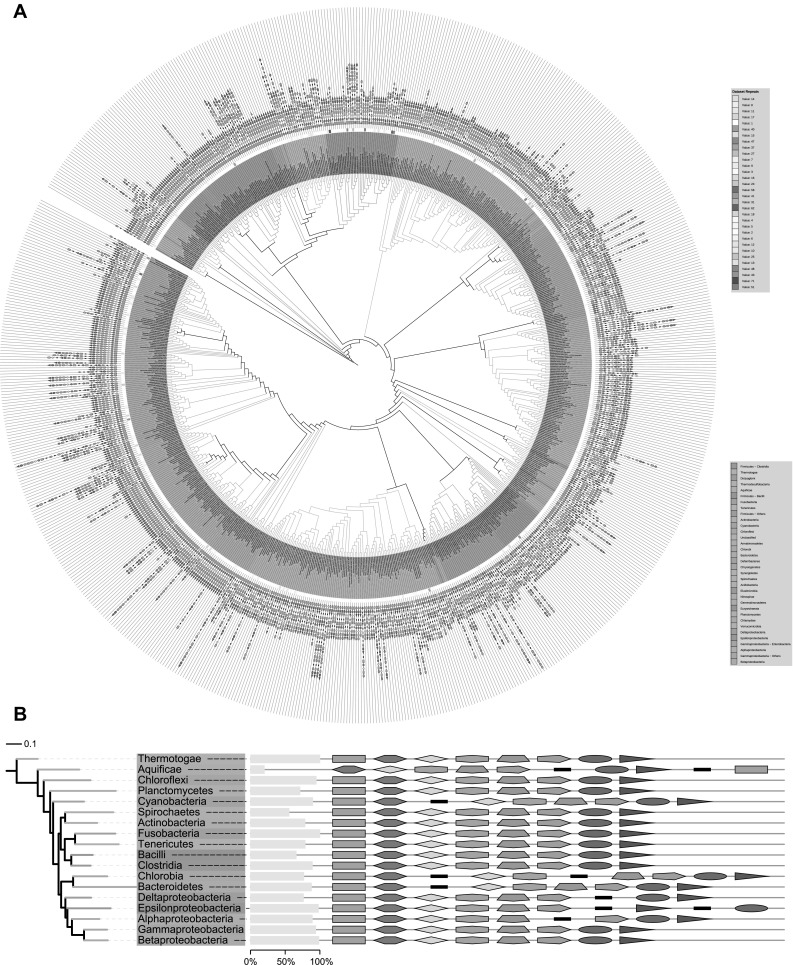
Dominant patterns of transcription units in bacterial classes and phyla. **a** A maximum-likelihood tree based on the bacterial 16S rDNA sequences. Bacterial classes and phyla are shown with distinct colors. A gradient of red color is used to represent the repeated transcription unit (modules) in the same genus. **b** A class/phylum phylogenetic tree combined with the dominant patterns of transcription units in each bacterial class and phylum. The histogram indicates the percentage of dominant patterns. (Color figure online)

To confirm this model, we constructed a phylogenetic tree based on the bacterial 16S rDNA sequences, and then mapped the transcription units modules to each branch (Fig. 4a). Interestingly, we did not observe a random distribution of different types of transcription and bacteria in the same class inclined to have the same transcription unit combination for the ATP synthase genes. For example, a considerable proportion of transcription units in the *Gammaproteobacteria* (94.0%), *Betaproteobacteria* (98.6%), *Actinobacteria* (78.6%), and many other bacterial classes (or phyla) were standard cTUs, and the genes in cTUs were generally arranged in a constant order. The phyla *Bacteroidetes* (87.8%) and *Cyanobacteria* (89.7%) had a combination of two distinct iTUs “ε-β” and “γ-α-δ-b-c-a,” while *Deltaproteobacteria* (76.3%) had “ε-β-γ-α-δ-b” and “c-a”; *Alphaproteobacteria* (89.1%) had “ε-β-γ-α-δ” and “b-c-a” and *Epsilonproteobacteria* (97.8%) had a triple transcription units “ε-β-γ-α-δ-b,” “c,” and “a” (Fig. 4b and Suppl. Table 8). The constant order of genes suggests that HGT may not be the major force in evolution of the F1Fo ATP synthase operons.

**Fig. 5:**
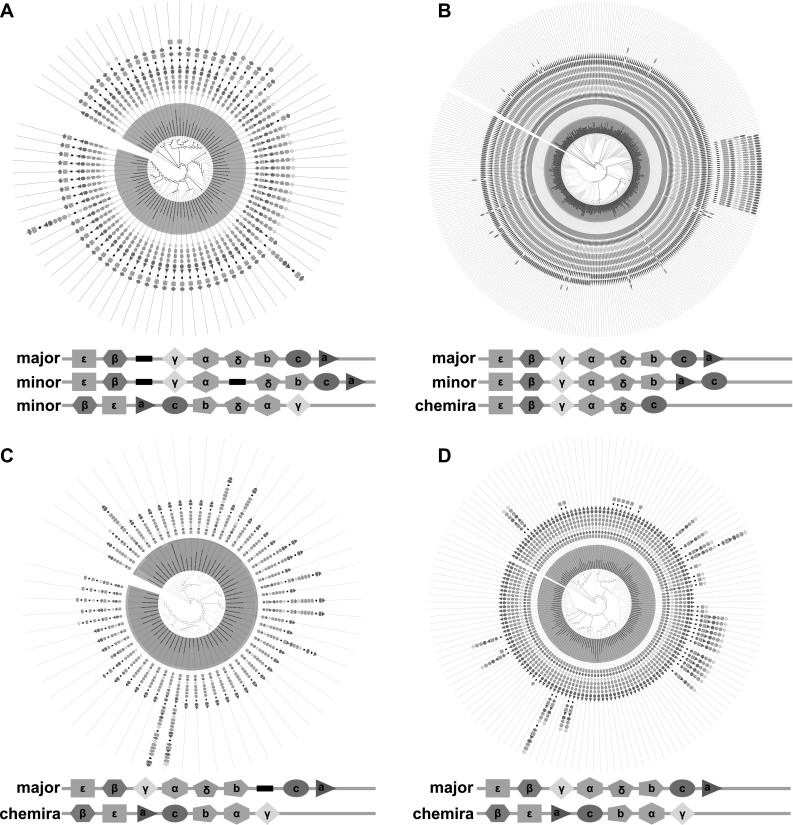
Distribution of minor cTUs and chimera fusions supporting the modular evolution. **a**–**d** The reconstructed phylogenetic tree combined with predicted transcription units containing F1Fo ATP synthase subunits in *Bacteroidetes, Bacilli, Deltaproteobacteria*, and *Betaproteobacteria*, respectively. Species in one genus are marked with the same color in bars and tree branches. (Color figure online)

To further investigate the possible evolutionary routes of bacterial F1Fo ATP synthase, we then mapped predicted transcription units into reconstructed phylogenetic trees in each bacterial phylum/class. Interestingly, the standard long transcription unit “ε-β-γ-α-δ-b-c-a” was not the only version of cTU. For example, the phylum *Bacteroidetes* possessed one minor cTU “β-ε-a-c-b-δ-α-γ” (Fig. 5a). In the class *Bacilli*, we found that the standard cTUs only occupy 65.9% of the 396 different species, while more than 30.8% of genomes contained another type of cTU, “ε-β-γ-α-δ-b-a-c” (Fig. 5b). Furthermore, phylogenetic analysis showed these minor cTUs concentrated in the species with rather close phylogenetic relationships, including the genera *Bacteroides* and *Prevotella* in *Bacteroidetes* (Fig. 5a) and genera *Lactococcus* and *Streptococcus* in *Bacilli* (Fig. 5b), indicating a relatively ancient gene inheritance. The minor cTUs could have been formed by the fusion of incomplete transcription modules, as the species adjacent to “β-ε-a-c-b-δ-α-γ” contained a combination pattern of “β-ε” plus “a-c-b-δ” plus “α-γ” or “β-ε” plus “a-c-b-δ-α-γ” (Fig. 5a). The minor cTU “ε-β-γ-α-δ-b-a-c” was possibly formed by the combination of “ε-β-γ-α-δ-b” and an inverse order of “c-a,” which took a large percentage in the class *Deltaproteobacteria* (Fig. 5b). Another novel trait in bacterial species was the presence of chimera fusions consisting of partial subunits in addition to the entire F1Fo ATP synthase. One of the frequent chimera fusions was “β-ε-a-c-b-α-γ” that was widely present in the classes *Deltaproteobacteria* (Fig. 5c), *Betaproteobacteria* (Fig. 5d), *Alphaproteobacteria* (Suppl. Fig. 6A), *Gammaproteobacteria* (Suppl. Fig. 6C), the phylum *Cyanobacteria* (Suppl. Table 6), and *Chlorobi* (Suppl. Table 6). The order of this kind of chimera fusion was consistent with the previously identified N-ATPase (Sumi et al. 1997; Dibrova et al. 2010), which is thought to be an early divergence from the ancestral ATPase (Dibrova et al. 2010). Other types of chimera fusion such as “ε-β-γ-α-δ-c” in *Bacilli* (Fig. 5b) and “ε-β-γ-α-b-c-a” in *Clostridia* (Suppl. Fig. 6B) were also detected. Considering these non-random modules (Fig. 3b) and other modules such as the F1 module (“ε-β-γ-α-δ”), “c,” “ε-β,” and “a-c-b,” the presence of chimera transcription units was not surprising and corroborating the modular evolution of F1Fo ATP synthase. Of these minor cTU and chimera fusions, only one special type of chimera fusion (“α-β”) in the phylum *Tenericutes* (Suppl. Fig. 6D, F) could not be predicted by direct combination of iTUs.

## Discussion

The F1Fo ATP synthase is a universal enzyme responsible for coupling ion movement across mitochondrial, chloroplast, or bacterial membranes with ATP synthesis. The structure of the synthase comprises a catalytic core (F1) and a membrane-bound subunit (Fo). The two functionally distinct subunits have been proposed to have evolved from functionally unrelated proteins (Mulkidjanian et al. 2007; Falk and Walker 1988; Rak et al. 2011). While the F1 subunit was proposed to have evolved from an ATP-dependent helicase, the Fo has been suggested to have evolved from a passive ion channel (Gomis-Ruth et al. 2001). The formation of the assembly intermediates (Rak et al. 2011; Havlickova et al. 2010) has suggested a more complex evolutionary process that may underlie the functional complexity of the synthase subunits.

### The Phylogenetic Profiles of the F1Fo ATP Synthase Subunits Support Modular Evolution

Our analysis of the phylogenetic profiles of the synthase subunits revealed two previously unknown evolutionary modules, the c-ring and α_3_β_3_ hexamer, in addition to the previously proposed F1 an Fo units (Walker 1998). The presence of the additional two modules, deduced from the phylogenetic profile correlations, could simply be attributed to the high sequence similarity between subunits within the same module. For instance, the amino acid sequences of α and β subunits show a high sequence similarity (BLASTP E-value < 0.0001 and 26% sequence identities) in both human and *E. coli*. This pair of subunits is known to have duplicated before the last universal common ancestor and have been universally inherited as a pair since then (Iwabe et al. 1989; Shih and Matzke 2013). Alternatively, the presence of the modules could be explained through the functional correlation and formation of the assembly intermediates. This notion is supported by the studies showing that the F1 assembly is independent from the rest of the complex (Tzagoloff 1969) and that the oligomerization of the c subunits forms a *bona fide* assembly intermediate (Rak et al. 2011). Furthermore, it has been shown that the disruption of the F1Fo assembly results in accumulation of the c-subunit ring structure as an independent subcomplex (Havlickova et al. 2010). The finding that the c-subunit ring constitutes an independent evolutionary module, in combination with our recent results ascribing a role for the c-subunit in formation of a non-selective ion channel in the mitochondrial inner membrane (Alavian et al. 2014) suggest that the ancestral function of the c-subunit evolutionary module, as a passive ion channel, may be conserved throughout evolution.

### Modular Evolution of F1Fo ATP Synthase Involved Fusion of Incomplete Transcription Units

Although a large number of bacterial classes possess a long and highly conserved transcription unit (cTU), our results strongly suggest that the bacterial F1Fo ATP synthase genes evolved from several distinct transcription unit modules. Gene order in transcription units is generally conserved among the phylogenetically close species, but this conservation is rapidly lost over a short period of evolutionary time (Tamames 2001). The order of the ATP synthase standard cTU, “ε-β-γ-α-δ-b-c-a,” however, remained rather stable even in distant bacterial classes (Fig. 4b), which argued against the involvement of horizontal gene transfer in this process. The constant order of ATP synthase genes in their genomic context is not a unique occurrence. The genes in ribosome, glutamate ABC transport system, and flagellum-related operons are also arranged in a highly ordered manner. The constant order is known to result from a conservative rearrangement of discrete sets of genes, which may be functionally related, have physical interaction, or share similar transcriptional regulatory elements (Tamames 2001; Lathe et al. 2000). Alternatively, these observed iTUs may have appeared due to the splits within the cTU, possibly by bacterial genome rearrangement. This scenario, however, seems unlikely since the frequency of genome rearrangement is high during evolution and the splitting of cTUs may interrupt the continuously arranged genes resulting in broken transcription units or replacement of native genes with foreign genes (Price et al. 2006). As the integrity of the transcription unit is of great importance for keeping the stoichiometric accuracy of the ATP synthase holoenzyme, any unexpected interruption would cause disadvantageous influences on the organisms. The highly ordered ATP synthase genes, therefore, seem to have been under a positive selection that forced them together. This was confirmed by the example of fusion of δ and b subunits of ATP synthase to a Rosetta stone.

The modular evolution of the synthase complex is further supported by the presence of the other types of cTUs and chimera fusion products in some bacteria. The presence of these unusual fusion products was predicted since it would be implausible to form long ordered transcription units by limited genome rearrangements during evolution. Further phylogenetic analysis of these minor cTUs supported the fusion model. In the phylum *Bacteroidetes*, bacteria species concentrated in genera *Bacteroides* and *Prevotella* had the minor cTU “β-ε-a-c-b-δ-α-γ” (Fig. 5a). At the same time, the combination patterns of “β-ε” plus “a-c-b-δ” plus “γ-α” were found at the nearby branches, which could be the resources formatting the minor cTUs. ATPase complexes translated from the minor cTUs were predicted as the actual F1Fo ATP synthase, because we simultaneously found no or partial A-type ATPase subunits in species with minor cTUs (Suppl. Table 6). Beside, we also identified a batch of chimera fusions consisting of F1Fo ATP synthase subunits. One of these chimera fusions (“β-ε-a-c-b-α-γ”) is identified as part of the N-ATPase operons (Dibrova et al. 2010). In agreement with the previous results on the N-ATPase (Dibrova et al. 2010), we found this chimera fusion transfer between bacterial species as one single unit. Interestingly, the N-ATPase operons share a similar architecture including the “β-ε,” “a-c-b-α-γ,” and one or two additional subunits between the first two modules (Suppl. Fig. 6E). Considering the early division of the N-ATPase, an ancient fusion event possibly participated in the formation of N-ATPase. The presence of these minor cTUs and chimera fusions strongly supports the involvement of distinct modules in the evolution of bacterial F1Fo ATP synthase.

### The Subunits in the Central and Peripheral Stalks May Provide the Link for the Integration of F1 and Fo

One of the common features observed by both the phylogenetic profiling and bacterial transcription unit analyses was that OSCP (δ in bacteria) and γ subunits were tightly gathered together with α and β subunits in the F1 component. More specifically, using a variety of independent methods, when the group number was set to 10, phylogenetic profiling results revealed that OSCP and γ subunits were consistently clustered into the same group with the α and β subunits (Fig. 1a, b). Additionally, transcription units such as “ε-β,” “γ-α-δ-b,” “ε-β-γ-α-δ,” and “ε-β-γ-α-δ-b” were frequently detected in bacterial genomes (Fig. 3b). These data, in addition to the known structure of F1Fo ATP synthase, showing direct interaction between γ and OSCP and the α_3_β_3_ hexamer (Rubinstein et al. 2003; Carbajo et al. 2005), suggest that these two subunits evolved together with the α_3_β_3_ hexamer to form the F1 component. In this model, the ancestral F1 and Fo subunits initially evolved separately (Mulkidjanian et al. 2007); the ancestral α_3_β_3_ hexamer, central stalk and part of peripheral stalk then may have formed as a stable protein complex. They were finally integrated with the self-assembled membrane-bound c-subunit ring structure. Bacterial and yeast studies have identified assembly intermediates of the F1Fo ATP synthase. They have not, however, determined whether the lateral stalk forms by sequential attachment of one of its subunits to the c ring or if it exists as an independent unit, which would then incorporate into the Fo (Rak et al. 2011; Houstek et al. 2006; Ruhle and Leister 2015). Our findings of the co-evolution of OSCP (δ in bacteria) with the α_3_β_3_ hexamer and the presence of the δ subunit in the same iTUs as α and b subunits suggest sequential attachment of the stalk subunits to the F1 compartment first and then integration into the Fo. The interaction of the b subunit and the stalk subunits with the c-subunit ring, therefore, may provide the link for the integration of F1 and Fo.

## Conclusion

Based on our analysis of the phylogenetic profiles of the human mitochondrial proteins, we present the presence of intermediary evolutionary modules for the F-type ATP synthase complex, in addition to the previously known overall F1 and Fo units. The intermediary modules comprise the α_3_β_3_ hexamer in the F1 and the c-subunit ring in the Fo as well as the central and lateral stalk subunits of the ATP synthase. Analysis of the bacterial transcription units supported the presence of the additional modules, as highly conserved combinations of separate transcription units are present among certain bacterial classes and phyla. Based on these findings, we propose a model that includes the involvement of multiple modules in the evolution of F1Fo ATP synthase. The central and peripheral stalk subunits provide a link for the integration of the F1/Fo modules.

## Electronic supplementary material

Below is the link to the electronic supplementary material.

Supple. File 1: Bacterial transcription unit data with improved prediction method. Supplementary material 1 (PDF 392 KB)

Supple. File 2: Raw bacterial transcription unit data retrieved from BioCyc database. (PDF 419 KB)

Supplementary material 3 (DOCX 38 KB)

Supplementary material 4 (PDF 2284 KB)

Supplementary material 5 (XLS 4206 KB)

## Abbreviations

OSCP: Oligomycin sensitivity conferral protein
HGT: Horizontal gene transfer
ECMs: Evolutionarily conserved modules
CLIME: Clustering by inferred models of evolution
cTU: Conserved transcription unit
iTU: Incomplete transcription unit
