# Supplementary material for: Phylogenetic Profiling of Mitochondrial Proteins and Integration Analysis of Bacterial Transcription Units Suggest Evolution of F1Fo ATP Synthase from Multiple Modules
Source: J Mol Evol. 2017 Nov 24;85(5):219–33. doi: 10.1007/s00239-017-9819-3 (PMC5709465; doi:10.1007/s00239-017-9819-3)
Supplement: Supplementary file 2 — Supple. File 2: Raw bacterial transcription unit data retrieved from BioCyc database. (PDF 419 KB) [file 239_2017_9819_MOESM2_ESM.pdf]

```
$`ABUT1036172-WGS`
$`ABUT1036172-WGS`$`TUSFG-642`
       bKO2        bKO1     deltaKO     alphaKO     gammaKO      betaKO
"GSFG-1587" "GSFG-1586" "GSFG-1585" "GSFG-1584" "GSFG-1583" "GSFG-1582"
  epsilonKO
"GSFG-1581"

$`ABUT1036172-WGS`$`TUSFG-679`
        cKO
"GSFG-1668"

$`ABUT1036172-WGS`$`TUSFG-781`
        aKO
"GSFG-1948"


$HACI382638
$HACI382638$`TUJAU-219`
 epsilonKO     betaKO    gammaKO    alphaKO    deltaKO       bKO2       bKO1
"GJAU-550" "GJAU-549" "GJAU-548" "GJAU-547" "GJAU-546" "GJAU-545" "GJAU-544"

$HACI382638$`TUJAU-638`
        cKO
"GJAU-1475"

$HACI382638$`TUJAU-462`
        aKO
"GJAU-1109"


$MHYO872331
$MHYO872331$`TUHIP-119`
 epsilonKO    betaKO1    gammaKO   alphaKO1    deltaKO        bKO        cKO
"GHIP-229" "GHIP-228" "GHIP-227" "GHIP-226" "GHIP-225" "GHIP-224" "GHIP-223"
       aKO
"GHIP-222"

$MHYO872331$`TUHIP-340`
  alphaKO2    betaKO2
"GHIP-683" "GHIP-682"


$ECOL386585
$ECOL386585$`TUJFA-2489`
        aKO         cKO         bKO     deltaKO     alphaKO     gammaKO
"GJFA-4648" "GJFA-4647" "GJFA-4646" "GJFA-4645" "GJFA-4644" "GJFA-4643"
     betaKO   epsilonKO
"GJFA-4642" "GJFA-4641"


$ECOL585057
$ECOL585057$`TUJ8I-2313`
        aKO         cKO         bKO     deltaKO     alphaKO     gammaKO
```

"GJ8I-4492" "GJ8I-4491" "GJ8I-4490" "GJ8I-4489" "GJ8I-4488" "GJ8I-4487"
betaKO epsilonKO
"GJ8I-4486" "GJ8I-4485"

$ECOL331111
$ECOL331111$`TUH7P-2335`
aKO cKO bKO deltaKO alphaKO gammaKO
"GH7P-4230" "GH7P-4229" "GH7P-4228" "GH7P-4227" "GH7P-4226" "GH7P-4225"
betaKO epsilonKO
"GH7P-4224" "GH7P-4223"

$ECOL331112
$ECOL331112$`TUHHI-2080`
aKO cKO bKO deltaKO alphaKO gammaKO
"GHHI-3947" "GHHI-3946" "GHHI-3945" "GHHI-3944" "GHHI-3943" "GHHI-3942"
betaKO epsilonKO
"GHHI-3941" "GHHI-3940"

$ECOL409438
$ECOL409438$`TUHUU-2254`
aKO cKO bKO deltaKO alphaKO gammaKO
"GHUU-4095" "GHUU-4094" "GHUU-4093" "GHUU-4092" "GHUU-4091" "GHUU-4090"
betaKO epsilonKO
"GHUU-4089" "GHUU-4088"

$ECOL585035
$ECOL585035$`TUJWP-2182`
aKO cKO bKO deltaKO alphaKO gammaKO
"GJWP-4146" "GJWP-4145" "GJWP-4144" "GJWP-4143" "GJWP-4142" "GJWP-4141"
betaKO epsilonKO
"GJWP-4140" "GJWP-4139"

$ECOL536056
$ECOL536056$`TUJMW-2312`
epsilonKO betaKO gammaKO alphaKO deltaKO bKO
"GJMW-4349" "GJMW-4348" "GJMW-4347" "GJMW-4346" "GJMW-4345" "GJMW-4344"
cKO aKO
"GJMW-4343" "GJMW-4342"

$ECLO1045856
$ECLO1045856$`TUHCE-2307`
epsilonKO betaKO gammaKO alphaKO
"GHCE-4637" "GHCE-4636" "GHCE-4635" "GHCE-4634"

$ECLO1045856$`TUHCE-2306`
deltaKO bKO cKO aKO
"GHCE-4632" "GHCE-4631" "GHCE-4630" "GHCE-4629"

```
$EELI515620
$EELI515620$`TUH1N-1013`
   deltaKO1    alphaKO1    gammaKO1     betaKO1  epsilonKO1 
"GH1N-1482" "GH1N-1481" "GH1N-1480" "GH1N-1479" "GH1N-1478" 

$EELI515620$`TUH1N-1218`
        aKO2         cKO2         bKO2    deltaKO2    alphaKO2    gammaKO2 
"GH1N-1933" "GH1N-1932" "GH1N-1931" "GH1N-1930" "GH1N-1929" "GH1N-1928" 
    betaKO2  epsilonKO2 
"GH1N-1927" "GH1N-1926" 

$EELI515620$`TUH1N-1015`
        aKO1         cKO1 
"GH1N-1485" "GH1N-1484" 

$EELI515620$`TUH1N-1014`
        bKO1 
"GH1N-1483" 



$EFAE226185
$EFAE226185$`TUHI1-1377`
         aKO          cKO          bKO      deltaKO      alphaKO      gammaKO 
"GHI1-2557" "GHI1-2556" "GHI1-2555" "GHI1-2554" "GHI1-2553" "GHI1-2552" 
      betaKO   epsilonKO 
"GHI1-2551" "GHI1-2550" 



$MHYO295358
$MHYO295358$`TUH8B-30`
  betaKO1   gammaKO  alphaKO1   deltaKO       bKO       cKO       aKO 
"GH8B-55" "GH8B-54" "GH8B-53" "GH8B-52" "GH8B-51" "GH8B-50" "GH8B-49" 

$MHYO295358$`TUH8B-253`
  alphaKO2    betaKO2 
"GH8B-502" "GH8B-501" 

$MHYO295358$noTU
epsilonKO 
       NA 



$MCAP340047
$MCAP340047$`TUC0H-41`
epsilonKO   betaKO1   gammaKO  alphaKO1   deltaKO       bKO       cKO       aKO 
"GC0H-85" "GC0H-84" "GC0H-83" "GC0H-82" "GC0H-81" "GC0H-80" "GC0H-79" "GC0H-78" 

$MCAP340047$`TUC0H-192`
   betaKO2   alphaKO2 
"GC0H-357" "GC0H-356" 



$`EFAE1305849-WGS`
```

```
$`EFAE1305849-WGS`$`TUSMY-1122`
        aKO         cKO         bKO     deltaKO     alphaKO     gammaKO
"GSMY-2127" "GSMY-2126" "GSMY-2125" "GSMY-2124" "GSMY-2123" "GSMY-2122"
     betaKO   epsilonKO
"GSMY-2121" "GSMY-2120"


$EFAE1155766
$EFAE1155766$`TULCU-1086`
        aKO         cKO         bKO     deltaKO     alphaKO     gammaKO
"GLCU-2094" "GLCU-2093" "GLCU-2092" "GLCU-2091" "GLCU-2090" "GLCU-2089"
     betaKO   epsilonKO
"GLCU-2088" "GLCU-2087"


$EFAE1206105
$EFAE1206105$`TULCS-1215`
        aKO         cKO         bKO     deltaKO     alphaKO     gammaKO
"GLCS-2228" "GLCS-2227" "GLCS-2226" "GLCS-2225" "GLCS-2224" "GLCS-2223"
     betaKO   epsilonKO
"GLCS-2222" "GLCS-2221"


$EFER585054
$EFER585054$`TUJJM-2063`
        aKO         cKO         bKO     deltaKO     alphaKO     gammaKO
"GJJM-4035" "GJJM-4034" "GJJM-4033" "GJJM-4032" "GJJM-4031" "GJJM-4030"
     betaKO   epsilonKO
"GJJM-4029" "GJJM-4028"


$EFAE474186
$EFAE474186$`TULCT-1023`
        aKO         cKO         bKO     deltaKO     alphaKO     gammaKO
"GLCT-2052" "GLCT-2051" "GLCT-2050" "GLCT-2049" "GLCT-2048" "GLCT-2047"
     betaKO   epsilonKO
"GLCT-2046" "GLCT-2045"


$EFAE936153
$EFAE936153$`TULCR-1533`
        aKO         cKO         bKO     deltaKO     alphaKO     gammaKO
"GLCR-2768" "GLCR-2767" "GLCR-2766" "GLCR-2765" "GLCR-2764" "GLCR-2763"
     betaKO   epsilonKO
"GLCR-2762" "GLCR-2761"


$`EFAE1104325-WGS`
$`EFAE1104325-WGS`$`TUSMZ-528`
 epsilonKO     betaKO   gammaKO    alphaKO    deltaKO        bKO        cKO
"GSMZ-735" "GSMZ-734" "GSMZ-733" "GSMZ-732" "GSMZ-731" "GSMZ-730" "GSMZ-729"
       aKO
"GSMZ-728"
```

```
$`EFAE1261557-WGS`
$`EFAE1261557-WGS`$`TUSOE-1117`
       aKO         cKO         bKO     deltaKO     alphaKO     gammaKO 
"GSOE-2145" "GSOE-2144" "GSOE-2143" "GSOE-2142" "GSOE-2141" "GSOE-2140" 
     betaKO   epsilonKO 
"GSOE-2139" "GSOE-2138" 


$EFAE333849
$EFAE333849$`TULCV-1276`
       aKO         cKO         bKO     deltaKO     alphaKO     gammaKO 
"GLCV-2092" "GLCV-2091" "GLCV-2090" "GLCV-2089" "GLCV-2088" "GLCV-2087" 
     betaKO   epsilonKO 
"GLCV-2086" "GLCV-2085" 


$MINF481448
$MINF481448$`TUJEI-410`
  gammaKO1   alphaKO1       bKO1 
"GJEI-861" "GJEI-860" "GJEI-859" 

$MINF481448$`TUJEI-1204`
 epsilonKO2     betaKO2    gammaKO2    alphaKO2 
"GJEI-2469" "GJEI-2468" "GJEI-2467" "GJEI-2466" 

$MINF481448$`TUJEI-409`
      cKO1       aKO1 epsilonKO1    betaKO1 
"GJEI-858" "GJEI-857" "GJEI-855" "GJEI-854" 

$MINF481448$`TUJEI-1203`
    deltaKO        bKO2        cKO2 
"GJEI-2465" "GJEI-2464" "GJEI-2463" 

$MINF481448$`TUJEI-1202`
       aKO2 
"GJEI-2462" 


$EHIR768486
$EHIR768486$`TULCW-929`
  epsilonKO      betaKO     gammaKO     alphaKO     deltaKO         bKO 
"GLCW-1693" "GLCW-1692" "GLCW-1691" "GLCW-1690" "GLCW-1689" "GLCW-1688" 
        cKO         aKO 
"GLCW-1687" "GLCW-1686" 


$`MCAN1205676-WGS`
$`MCAN1205676-WGS`$`TUSQV-735`
  epsilonKO      betaKO     gammaKO     alphaKO         bKO         cKO 
"GSQV-1370" "GSQV-1369" "GSQV-1368" "GSQV-1367" "GSQV-1365" "GSQV-1364" 
        aKO 
"GSQV-1363" 
```

```
$`MCAN1205676-WGS`$noTU
deltaKO
     NA


$EICT634503
$EICT634503$`TUCMY-2205`
  epsilonKO      betaKO     gammaKO     alphaKO     deltaKO         bKO
"GCMY-3889" "GCMY-3888" "GCMY-3887" "GCMY-3886" "GCMY-3885" "GCMY-3884"
        cKO         aKO
"GCMY-3883" "GCMY-3882"


$ECOL714962
$ECOL714962$`TUI9T-2135`
        aKO         cKO         bKO     deltaKO     alphaKO     gammaKO
"GI9T-4185" "GI9T-4184" "GI9T-4183" "GI9T-4182" "GI9T-4181" "GI9T-4180"
     betaKO   epsilonKO
"GI9T-4179" "GI9T-4178"


$ECOL595495
$ECOL595495$`TUI1Q-2443`
  epsilonKO      betaKO     gammaKO     alphaKO     deltaKO         bKO
"GI1Q-4724" "GI1Q-4723" "GI1Q-4722" "GI1Q-4721" "GI1Q-4720" "GI1Q-4719"
        cKO         aKO
"GI1Q-4718" "GI1Q-4717"


$ECOL885275
$ECOL885275$`TUJE6-2179`
    deltaKO     alphaKO     gammaKO      betaKO   epsilonKO
"GJE6-4270" "GJE6-4269" "GJE6-4268" "GJE6-4267" "GJE6-4266"

$ECOL885275$`TUJE6-2180`
        aKO         cKO         bKO
"GJE6-4274" "GJE6-4273" "GJE6-4272"


$ECOL885276
$ECOL885276$`TUJE7-2179`
    deltaKO     alphaKO     gammaKO      betaKO   epsilonKO
"GJE7-4270" "GJE7-4269" "GJE7-4268" "GJE7-4267" "GJE7-4266"

$ECOL885276$`TUJE7-2180`
        aKO         cKO         bKO
"GJE7-4274" "GJE7-4273" "GJE7-4272"


$ELEN479437
$ELEN479437$`TUHWY-558`
  epsilonKO      betaKO     gammaKO     alphaKO     deltaKO         bKO
"GHWY-1061" "GHWY-1060" "GHWY-1059" "GHWY-1058" "GHWY-1057" "GHWY-1056"
        cKO         aKO
```

```
"GHWY-1055" "GHWY-1054"


$ECOL591946
$ECOL591946$`TUJE3-1919`
        aKO         cKO         bKO     deltaKO     alphaKO     gammaKO
"GJE3-3798" "GJE3-3797" "GJE3-3796" "GJE3-3795" "GJE3-3794" "GJE3-3793"
     betaKO   epsilonKO
"GJE3-3792" "GJE3-3791"


$ECOL316401
$ECOL316401$`TULD0-2286`
        aKO         cKO         bKO     deltaKO     alphaKO     gammaKO
"GLD0-4096" "GLD0-4095" "GLD0-4094" "GLD0-4093" "GLD0-4092" "GLD0-4091"
     betaKO   epsilonKO
"GLD0-4090" "GLD0-4089"


$`LLAC1046624-WGS`
$`LLAC1046624-WGS`$`TUSPE-1114`
        cKO         aKO         bKO     deltaKO     alphaKO     gammaKO
"GSPE-1791" "GSPE-1790" "GSPE-1789" "GSPE-1788" "GSPE-1787" "GSPE-1786"
     betaKO   epsilonKO
"GSPE-1785" "GSPE-1784"


$ELIT314225
$ELIT314225$`TUHLE-942`
  epsilonKO      betaKO     gammaKO     alphaKO     deltaKO
"GHLE-1791" "GHLE-1790" "GHLE-1788" "GHLE-1787" "GHLE-1786"

$ELIT314225$`TUHLE-808`
        aKO         cKO
"GHLE-1538" "GHLE-1537"

$ELIT314225$`TUHLE-807`
       bKO2        bKO1
"GHLE-1536" "GHLE-1535"


$ECOL685038
$ECOL685038$`TUI9V-1996`
        aKO         cKO         bKO     deltaKO     alphaKO     gammaKO
"GI9V-3771" "GI9V-3770" "GI9V-3769" "GI9V-3768" "GI9V-3767" "GI9V-3766"
     betaKO   epsilonKO
"GI9V-3765" "GI9V-3764"


$MCAT749219
$MCAT749219$`TUHBK-240`
   gammaKO    alphaKO    deltaKO        bKO        cKO        aKO
"GHBK-396" "GHBK-395" "GHBK-394" "GHBK-393" "GHBK-392" "GHBK-391"
```

$MCAT749219$`TUHBK-241`
 epsilonKO   betaKO
"GHBK-399" "GHBK-398"

$AMAC529120
$AMAC529120$`TUL7I-2271`
       aKO   cKO   bKO   deltaKO   alphaKO   gammaKO
"GL7I-3927" "GL7I-3926" "GL7I-3925" "GL7I-3924" "GL7I-3923" "GL7I-3922"
    betaKO   epsilonKO
"GL7I-3921" "GL7I-3920"

$`AMAC1300259-WGS`
$`AMAC1300259-WGS`$`TUSF4-2186`
       aKO   cKO   bKO   deltaKO   alphaKO   gammaKO
"GSF4-3927" "GSF4-3926" "GSF4-3925" "GSF4-3924" "GSF4-3923" "GSF4-3922"
    betaKO   epsilonKO
"GSF4-3921" "GSF4-3920"

$`AMAC1300256-WGS`
$`AMAC1300256-WGS`$`TUSGA-2186`
       aKO   cKO   bKO   deltaKO   alphaKO   gammaKO
"GSGA-3917" "GSGA-3916" "GSGA-3915" "GSGA-3914" "GSGA-3913" "GSGA-3912"
    betaKO   epsilonKO
"GSGA-3911" "GSGA-3910"

$`AMAC1300258-WGS`
$`AMAC1300258-WGS`$`TUSF5-2420`
       aKO   cKO   bKO   deltaKO   alphaKO   gammaKO
"GSF5-4113" "GSF5-4112" "GSF5-4111" "GSF5-4110" "GSF5-4109" "GSF5-4108"
    betaKO   epsilonKO
"GSF5-4107" "GSF5-4106"

$ECOL216592
$ECOL216592$`TUCV7-2212`
       aKO   cKO   bKO   deltaKO   alphaKO   gammaKO
"GCV7-4171" "GCV7-4170" "GCV7-4169" "GCV7-4168" "GCV7-4167" "GCV7-4166"
    betaKO   epsilonKO
"GCV7-4165" "GCV7-4164"

$ECOL1048689
$ECOL1048689$`TULD6-2339`
       aKO   cKO   bKO   deltaKO   alphaKO   gammaKO
"GLD6-4352" "GLD6-4351" "GLD6-4350" "GLD6-4349" "GLD6-4348" "GLD6-4347"
    betaKO   epsilonKO
"GLD6-4346" "GLD6-4345"

$ECOL869729

```
$ECOL869729$`TUI9Z-2007`
        aKO         cKO         bKO     deltaKO     alphaKO     gammaKO 
"GI9Z-3762" "GI9Z-3761" "GI9Z-3760" "GI9Z-3759" "GI9Z-3758" "GI9Z-3757" 
     betaKO   epsilonKO 
"GI9Z-3756" "GI9Z-3755" 


$MMYC862259
$MMYC862259$`TUI4X-324`
  alphaKO1    betaKO1 
"GI4X-604" "GI4X-603" 

$MMYC862259$`TUI4X-438`
       aKO        cKO        bKO    deltaKO   alphaKO2    gammaKO    betaKO2 
"GI4X-842" "GI4X-841" "GI4X-840" "GI4X-839" "GI4X-838" "GI4X-837" "GI4X-836" 
 epsilonKO 
"GI4X-835" 


$ECOL566546
$ECOL566546$`TUJE5-2137`
        bKO     deltaKO     alphaKO 
"GJE5-4029" "GJE5-4028" "GJE5-4027" 

$ECOL566546$`TUJE5-2136`
    gammaKO      betaKO   epsilonKO 
"GJE5-4026" "GJE5-4025" "GJE5-4024" 

$ECOL566546$`TUJE5-2138`
        cKO 
"GJE5-4030" 

$ECOL566546$`TUJE5-2139`
        aKO 
"GJE5-4031" 


$ECOL741093
$ECOL741093$`TULD7-2460`
        aKO         cKO         bKO     deltaKO     alphaKO     gammaKO 
"GLD7-4500" "GLD7-4499" "GLD7-4498" "GLD7-4497" "GLD7-4496" "GLD7-4495" 
     betaKO   epsilonKO 
"GLD7-4494" "GLD7-4493" 


$EMIN445932
$EMIN445932$`TUHM0-706`
    alphaKO     deltaKO         bKO         cKO         aKO 
"GHM0-1565" "GHM0-1564" "GHM0-1563" "GHM0-1562" "GHM0-1561" 

$EMIN445932$`TUHM0-707`
  epsilonKO      betaKO     gammaKO 
"GHM0-1569" "GHM0-1568" "GHM0-1567" 
```

```
$MCUR548479
$MCUR548479$`TUH17-621`
  epsilonKO      betaKO     gammaKO     alphaKO     deltaKO         bKO
"GH17-1139" "GH17-1138" "GH17-1137" "GH17-1136" "GH17-1135" "GH17-1134"
        cKO         aKO
"GH17-1133" "GH17-1132"


$`EMUN1300150-WGS`
$`EMUN1300150-WGS`$`TUSN0-1263`
        aKO         cKO         bKO     deltaKO     alphaKO     gammaKO
"GSN0-2027" "GSN0-2026" "GSN0-2025" "GSN0-2024" "GSN0-2023" "GSN0-2022"
     betaKO   epsilonKO
"GSN0-2021" "GSN0-2020"


$ECOL1033813
$ECOL1033813$`TUI9U-2026`
        aKO         cKO         bKO     deltaKO     alphaKO     gammaKO
"GI9U-3976" "GI9U-3975" "GI9U-3974" "GI9U-3973" "GI9U-3972" "GI9U-3971"
     betaKO   epsilonKO
"GI9U-3970" "GI9U-3969"


$ECLO716541
$ECLO716541$`TUH13-2874`
  epsilonKO      betaKO     gammaKO     alphaKO     deltaKO         bKO
"GH13-5225" "GH13-5224" "GH13-5223" "GH13-5222" "GH13-5221" "GH13-5220"
        cKO         aKO
"GH13-5219" "GH13-5218"


$`ECLO1104326-WGS`
$`ECLO1104326-WGS`$`TUSMQ-2383`
  epsilonKO      betaKO     gammaKO     alphaKO     deltaKO         bKO
"GSMQ-4628" "GSMQ-4627" "GSMQ-4626" "GSMQ-4625" "GSMQ-4624" "GSMQ-4623"
        cKO         aKO
"GSMQ-4622" "GSMQ-4621"


$ECLO1211025
$ECLO1211025$`TULCQ-10`
epsilonKO    betaKO   gammaKO   alphaKO   deltaKO       bKO       cKO       aKO
"GLCQ-25" "GLCQ-24" "GLCQ-23" "GLCQ-22" "GLCQ-21" "GLCQ-20" "GLCQ-19" "GLCQ-18"


$ESP399742
$ESP399742$`TUJ0E-2272`
  epsilonKO      betaKO     gammaKO     alphaKO     deltaKO         bKO
"GJ0E-4241" "GJ0E-4240" "GJ0E-4239" "GJ0E-4238" "GJ0E-4237" "GJ0E-4236"
        cKO         aKO
"GJ0E-4235" "GJ0E-4234"
```

$MMOB267748
$MMOB267748$`TUH6Y-85`
betaKO1 alphaKO1
"GH6Y-174" "GH6Y-173"

$MMOB267748$`TUH6Y-107`
aKO cKO bKO deltaKO alphaKO2 gammaKO betaKO2
"GH6Y-224" "GH6Y-223" "GH6Y-222" "GH6Y-221" "GH6Y-220" "GH6Y-218" "GH6Y-217"
epsilonKO
"GH6Y-216"

$MMOB267748$`TUH6Y-138`
alphaKO3 betaKO3
"GH6Y-311" "GH6Y-310"

$ECOL1072459
$ECOL1072459$`TULD5-2323`
bKO deltaKO alphaKO
"GLD5-4372" "GLD5-4371" "GLD5-4370"

$ECOL1072459$`TULD5-2322`
gammaKO betaKO epsilonKO
"GLD5-4369" "GLD5-4368" "GLD5-4367"

$ECOL1072459$`TULD5-2324`
cKO
"GLD5-4373"

$ECOL1072459$`TULD5-2325`
aKO
"GLD5-4374"

$ECOL585395
$ECOL585395$`TUJA9-2407`
alphaKO deltaKO bKO cKO aKO
"GJA9-4589" "GJA9-4588" "GJA9-4587" "GJA9-4586" "GJA9-4585"

$ECOL585395$`TUJA9-2408`
epsilonKO betaKO gammaKO
"GJA9-4592" "GJA9-4591" "GJA9-4590"

$ECOL585396
$ECOL585396$`TUJCW-2732`
bKO deltaKO alphaKO
"GJCW-4726" "GJCW-4725" "GJCW-4724"

$ECOL585396$`TUJCW-2731`
gammaKO betaKO epsilonKO
"GJCW-4723" "GJCW-4722" "GJCW-4721"

```
$ECOL585396$`TUJCW-2733`
        cKO
"GJCW-4727"

$ECOL585396$`TUJCW-2734`
        aKO
"GJCW-4728"


$ECOL573235
$ECOL573235$`TUCY7-2722`
    alphaKO     deltaKO         bKO
"GCY7-5010" "GCY7-5009" "GCY7-5008"

$ECOL573235$`TUCY7-2723`
  epsilonKO      betaKO     gammaKO
"GCY7-5013" "GCY7-5012" "GCY7-5011"

$ECOL573235$`TUCY7-2721`
        cKO
"GCY7-5007"

$ECOL573235$`TUCY7-2720`
        aKO
"GCY7-5006"


$`MCAN1205675-WGS`
$`MCAN1205675-WGS`$`TUSQW-743`
  epsilonKO      betaKO     gammaKO     alphaKO         bKO         cKO
"GSQW-1379" "GSQW-1378" "GSQW-1377" "GSQW-1376" "GSQW-1374" "GSQW-1373"
        aKO
"GSQW-1372"

$`MCAN1205675-WGS`$noTU
deltaKO
     NA


$ECOL701177
$ECOL701177$`TUI1N-2370`
        aKO         cKO         bKO     deltaKO     alphaKO     gammaKO
"GI1N-4535" "GI1N-4534" "GI1N-4533" "GI1N-4532" "GI1N-4531" "GI1N-4530"
     betaKO   epsilonKO
"GI1N-4529" "GI1N-4528"


$EOLI929562
$EOLI929562$`TULCO-1085`
    alphaKO     gammaKO
"GLCO-1294" "GLCO-1293"

$EOLI929562$`TULCO-1357`
     betaKO
```

"GLCO-1683"

$EOLI929562$`TULCO-2867`
aKO cKO bKO deltaKO
"GLCO-4025" "GLCO-4024" "GLCO-4023" "GLCO-4022"

$EOLI929562$`TULCO-1358`
epsilonKO
"GLCO-1684"

$EPYR644651
$EPYR644651$`TULCX-35`
aKO cKO bKO deltaKO alphaKO gammaKO
"GLCX-4057" "GLCX-4056" "GLCX-4055" "GLCX-4054" "GLCX-4053" "GLCX-4052"
betaKO epsilonKO
"GLCX-4051" "GLCX-4050"

$EPYR634499
$EPYR634499$`TUJIP-35`
aKO cKO bKO deltaKO alphaKO gammaKO
"GJIP-3794" "GJIP-3793" "GJIP-3792" "GJIP-3791" "GJIP-3790" "GJIP-3789"
betaKO epsilonKO
"GJIP-3788" "GJIP-3787"

$`EREC657317-WGS`
$`EREC657317-WGS`$`TUSND-149`
epsilonKO1 betaKO1 gammaKO1 alphaKO cKO1
"GSND-254" "GSND-253" "GSND-252" "GSND-251" "GSND-250"

$`EREC657317-WGS`$`TUSND-548`
aKO cKO2 bKO deltaKO gammaKO2 betaKO2 epsilonKO2
"GSND-917" "GSND-916" "GSND-915" "GSND-914" "GSND-913" "GSND-912" "GSND-911"

$MMYC272632
$MMYC272632$`TUI1G-339`
alphaKO1 betaKO1
"GI1G-606" "GI1G-605"

$MMYC272632$`TUI1G-487`
aKO cKO bKO deltaKO alphaKO2 gammaKO betaKO2
"GI1G-885" "GI1G-884" "GI1G-883" "GI1G-882" "GI1G-881" "GI1G-880" "GI1G-879"
epsilonKO
"GI1G-878"

$ERUM302409
$ERUM302409$`TUHVW-50`
deltaKO alphaKO
"GHVW-79" "GHVW-78"

```
$ERUM302409$`TUHVW-320`
    betaKO  epsilonKO
"GHVW-496" "GHVW-495"

$ERUM302409$`TUHVW-281`
   gammaKO
"GHVW-430"

$ERUM302409$`TUHVW-571`
      bKO2       bKO1        cKO        aKO
"GHVW-917" "GHVW-916" "GHVW-915" "GHVW-914"


$ERHU650150
$ERHU650150$`TUHGV-192`
 epsilonKO     betaKO    gammaKO    alphaKO    deltaKO        bKO        cKO
"GHGV-395" "GHGV-394" "GHGV-393" "GHGV-392" "GHGV-391" "GHGV-390" "GHGV-389"
       aKO
"GHGV-388"


$ESP215689
$ESP215689$`TULCY-669`
        aKO         cKO         bKO     deltaKO     alphaKO     gammaKO
"GLCY-1187" "GLCY-1186" "GLCY-1185" "GLCY-1184" "GLCY-1183" "GLCY-1182"
     betaKO   epsilonKO
"GLCY-1181" "GLCY-1180"


$`ERHU1313290-WGS`
$`ERHU1313290-WGS`$`TUSN1-867`
  epsilonKO      betaKO     gammaKO     alphaKO     deltaKO         bKO
"GSN1-1789" "GSN1-1788" "GSN1-1787" "GSN1-1786" "GSN1-1785" "GSN1-1784"
        cKO         aKO
"GSN1-1783" "GSN1-1782"


$`EREC657318-WGS`
$`EREC657318-WGS`$`TUSNC-1577`
 epsilonKO2     betaKO2    gammaKO2     alphaKO       cKO2
"GSNC-2747" "GSNC-2746" "GSNC-2745" "GSNC-2744" "GSNC-2743"

$`EREC657318-WGS`$`TUSNC-472`
epsilonKO1    betaKO1   gammaKO1    deltaKO        bKO       cKO1        aKO
"GSNC-807" "GSNC-806" "GSNC-805" "GSNC-804" "GSNC-803" "GSNC-802" "GSNC-801"


$`APHA1184254-WGS`
$`APHA1184254-WGS`$`TUSFP-815`
    deltaKO     alphaKO
"GSFP-1238" "GSFP-1237"

$`APHA1184254-WGS`$`TUSFP-295`
    betaKO
```

```
"GSFP-483"

$`APHA1184254-WGS`$`TUSFP-405`
  gammaKO1
"GSFP-659"

$`APHA1184254-WGS`$`TUSFP-463`
  gammaKO2
"GSFP-744"

$`APHA1184254-WGS`$`TUSFP-294`
 epsilonKO
"GSFP-482"

$`APHA1184254-WGS`$`TUSFP-725`
        aKO         cKO        bKO2        bKO1
"GSFP-1102" "GSFP-1101" "GSFP-1100" "GSFP-1099"


$`MCAN1205674-WGS`
$`MCAN1205674-WGS`$`TUSQX-765`
  epsilonKO      betaKO     gammaKO     alphaKO         bKO         cKO
"GSQX-1416" "GSQX-1415" "GSQX-1414" "GSQX-1413" "GSQX-1411" "GSQX-1410"
        aKO
"GSQX-1409"

$`MCAN1205674-WGS`$noTU
deltaKO
     NA


$ERUM254945
$ERUM254945$`TUJ2L-48`
  deltaKO   alphaKO
"GJ2L-82" "GJ2L-81"

$ERUM254945$`TUJ2L-315`
    betaKO  epsilonKO
"GJ2L-506" "GJ2L-505"

$ERUM254945$`TUJ2L-269`
   gammaKO
"GJ2L-435"

$ERUM254945$`TUJ2L-571`
      bKO2       bKO1        cKO        aKO
"GJ2L-924" "GJ2L-923" "GJ2L-922" "GJ2L-921"


$CSAK290339
$CSAK290339$`TUJ80-2177`
        aKO         cKO         bKO     deltaKO     alphaKO     gammaKO
"GJ80-4001" "GJ80-4000" "GJ80-3999" "GJ80-3998" "GJ80-3997" "GJ80-3996"
     betaKO   epsilonKO
```

```
"GJ80-3995" "GJ80-3994"


$ECLO701347
$ECLO701347$`TUH9V-2320`
  epsilonKO      betaKO     gammaKO     alphaKO     deltaKO         bKO
"GH9V-4531" "GH9V-4530" "GH9V-4529" "GH9V-4528" "GH9V-4527" "GH9V-4526"
        cKO         aKO
"GH9V-4525" "GH9V-4524"


$MNOD460265
$MNOD460265$`TUCZK-4948`
    deltaKO     alphaKO     gammaKO      betaKO   epsilonKO
"GCZK-7468" "GCZK-7467" "GCZK-7466" "GCZK-7465" "GCZK-7464"

$MNOD460265$`TUCZK-5140`
        cKO         aKO
"GCZK-7746" "GCZK-7745"

$MNOD460265$`TUCZK-5141`
       bKO2        bKO1
"GCZK-7748" "GCZK-7747"


$ECOL431946
$ECOL431946$`TUIA0-1949`
        aKO         cKO         bKO     deltaKO     alphaKO     gammaKO
"GIA0-3657" "GIA0-3656" "GIA0-3655" "GIA0-3654" "GIA0-3653" "GIA0-3652"
     betaKO   epsilonKO
"GIA0-3651" "GIA0-3650"


$ESIB262543
$ESIB262543$`TUHBP-1427`
        aKO         cKO         bKO     deltaKO     alphaKO     gammaKO
"GHBP-2764" "GHBP-2763" "GHBP-2762" "GHBP-2761" "GHBP-2760" "GHBP-2759"
     betaKO   epsilonKO
"GHBP-2758" "GHBP-2757"


$ECOL1133852
$ECOL1133852$`TULD4-2741`
  epsilonKO      betaKO     gammaKO     alphaKO     deltaKO         bKO
"GLD4-5061" "GLD4-5060" "GLD4-5059" "GLD4-5058" "GLD4-5057" "GLD4-5056"
        cKO         aKO
"GLD4-5055" "GLD4-5054"


$ECOL1134782
$ECOL1134782$`TULD2-2765`
  epsilonKO      betaKO     gammaKO     alphaKO     deltaKO         bKO
"GLD2-5053" "GLD2-5052" "GLD2-5051" "GLD2-5050" "GLD2-5049" "GLD2-5048"
        cKO         aKO
```

```
"GLD2-5047" "GLD2-5046"


$ECOL1133853
$ECOL1133853$`TULD3-2751`
  epsilonKO      betaKO     gammaKO     alphaKO     deltaKO         bKO
"GLD3-5127" "GLD3-5126" "GLD3-5125" "GLD3-5124" "GLD3-5123" "GLD3-5122"
        cKO         aKO
"GLD3-5121" "GLD3-5120"


$`ESIR717961-WGS`
$`ESIR717961-WGS`$`TUSNG-259`
       aKO        cKO        bKO    deltaKO    alphaKO    gammaKO     betaKO
"GSNG-438" "GSNG-437" "GSNG-436" "GSNG-435" "GSNG-434" "GSNG-432" "GSNG-431"
 epsilonKO
"GSNG-429"


$`ESIR657319-WGS`
$`ESIR657319-WGS`$`TUSO8-1149`
  epsilonKO      betaKO     gammaKO     alphaKO     deltaKO         bKO
"GSO8-2156" "GSO8-2154" "GSO8-2153" "GSO8-2151" "GSO8-2150" "GSO8-2149"
        cKO         aKO
"GSO8-2148" "GSO8-2147"


$`MCYN1246955-WGS`
$`MCYN1246955-WGS`$`TUSSK-151`
 epsilonKO    betaKO1    gammaKO   alphaKO1    deltaKO        bKO        cKO
"GSSK-320" "GSSK-319" "GSSK-318" "GSSK-317" "GSSK-316" "GSSK-315" "GSSK-314"
       aKO
"GSSK-313"

$`MCYN1246955-WGS`$`TUSSK-300`
  alphaKO2    betaKO2
"GSSK-633" "GSSK-632"


$ETAS465817
$ETAS465817$`TUI36-121`
        aKO         cKO         bKO     deltaKO     alphaKO     gammaKO
"GI36-3589" "GI36-3588" "GI36-3587" "GI36-3586" "GI36-3585" "GI36-3584"
     betaKO   epsilonKO
"GI36-3583" "GI36-3582"


$`ETAR1288122-WGS`
$`ETAR1288122-WGS`$`TUSMO-1936`
  epsilonKO      betaKO     gammaKO     alphaKO     deltaKO         bKO
"GSMO-3505" "GSMO-3504" "GSMO-3503" "GSMO-3502" "GSMO-3501" "GSMO-3500"
        cKO         aKO
"GSMO-3499" "GSMO-3498"
```

```
$`MPUT1292033-WGS`
$`MPUT1292033-WGS`$`TUSRW-211`
  alphaKO1    betaKO1
"GSRW-405" "GSRW-404"

$`MPUT1292033-WGS`$`TUSRW-329`
       aKO        cKO        bKO    deltaKO   alphaKO2    gammaKO    betaKO2
"GSRW-673" "GSRW-672" "GSRW-671" "GSRW-670" "GSRW-669" "GSRW-668" "GSRW-667"
 epsilonKO
"GSRW-666"


$`AMAC1300255-WGS`
$`AMAC1300255-WGS`$`TUSFE-2325`
        aKO         cKO         bKO     deltaKO     alphaKO     gammaKO
"GSFE-3864" "GSFE-3863" "GSFE-3862" "GSFE-3861" "GSFE-3860" "GSFE-3859"
     betaKO   epsilonKO
"GSFE-3858" "GSFE-3857"


$`AMAC1300257-WGS`
$`AMAC1300257-WGS`$`TUSF3-2144`
        aKO         cKO         bKO     deltaKO     alphaKO     gammaKO
"GSF3-3859" "GSF3-3858" "GSF3-3857" "GSF3-3856" "GSF3-3855" "GSF3-3854"
     betaKO   epsilonKO
"GSF3-3853" "GSF3-3852"


$ETAR718251
$ETAR718251$`TULCN-1891`
  epsilonKO      betaKO     gammaKO     alphaKO     deltaKO         bKO
"GLCN-3307" "GLCN-3306" "GLCN-3305" "GLCN-3304" "GLCN-3303" "GLCN-3302"
        cKO         aKO
"GLCN-3301" "GLCN-3300"


$ETAR498217
$ETAR498217$`TUJC4-2082`
    alphaKO     deltaKO         bKO         cKO
"GJC4-3645" "GJC4-3644" "GJC4-3643" "GJC4-3642"

$ETAR498217$`TUJC4-2083`
  epsilonKO      betaKO     gammaKO
"GJC4-3648" "GJC4-3647" "GJC4-3646"

$ETAR498217$`TUJC4-2081`
        aKO
"GJC4-3641"


$ECOL544404
$ECOL544404$`TUKCX-2610`
        bKO     deltaKO     alphaKO
```

```
"GKCX-4779" "GKCX-4778" "GKCX-4777"

$ECOL544404$`TUKCX-2609`
   gammaKO      betaKO   epsilonKO
"GKCX-4776" "GKCX-4775" "GKCX-4774"

$ECOL544404$`TUKCX-2611`
        cKO
"GKCX-4780"

$ECOL544404$`TUKCX-2612`
        aKO
"GKCX-4781"



$ECOL585056
$ECOL585056$`TUCW1-9878`
        aKO          cKO          bKO     deltaKO     alphaKO     gammaKO
"GCW1-1534" "GCW1-1537" "GCW1-1538" "GCW1-1540" "GCW1-1533" "GCW1-1539"
     betaKO   epsilonKO
"GCW1-1536" "GCW1-1535"



$ECOL696406
$ECOL696406$`TUJE4-2623`
        aKO          cKO          bKO     deltaKO     alphaKO     gammaKO
"GJE4-4503" "GJE4-4502" "GJE4-4501" "GJE4-4500" "GJE4-4499" "GJE4-4498"
     betaKO   epsilonKO
"GJE4-4497" "GJE4-4496"



$`EVIE926556-WGS`
$`EVIE926556-WGS`$`TUSMN-329`
       aKO        cKO        bKO    deltaKO    alphaKO    gammaKO
"GSMN-612" "GSMN-611" "GSMN-610" "GSMN-609" "GSMN-608" "GSMN-607"

$`EVIE926556-WGS`$`TUSMN-1025`
  epsilonKO      betaKO
"GSMN-1767" "GSMN-1766"



$`MCAN1205677-WGS`
$`MCAN1205677-WGS`$`TUSQY-763`
  epsilonKO      betaKO     gammaKO     alphaKO          bKO          cKO
"GSQY-1413" "GSQY-1412" "GSQY-1411" "GSQY-1410" "GSQY-1408" "GSQY-1407"
        aKO
"GSQY-1406"

$`MCAN1205677-WGS`$noTU
deltaKO
     NA



$ESP502558
```

```
$ESP502558$`TUI1S-1103`
       bKO     deltaKO     alphaKO     gammaKO      betaKO   epsilonKO
"GI1S-1946" "GI1S-1945" "GI1S-1944" "GI1S-1943" "GI1S-1942" "GI1S-1941"

$ESP502558$`TUI1S-1104`
       cKO
"GI1S-1947"

$ESP502558$`TUI1S-1105`
       aKO
"GI1S-1948"


$AGRO
$AGRO$`TUN-16467`
  deltaKO   alphaKO   gammaKO    betaKO epsilonKO
"ATU2625" "ATU2624" "ATU2623" "ATU2622" "ATU2621"

$AGRO$`TUN-15352`
     bKO2      bKO1       aKO
"ATU0717" "ATU0716" "ATU0714"

$AGRO$noTU
cKO
 NA


$FALO546269
$FALO546269$`TUHHW-449`
      aKO        cKO        bKO    deltaKO    alphaKO    gammaKO     betaKO
"GHHW-829" "GHHW-827" "GHHW-826" "GHHW-825" "GHHW-824" "GHHW-823" "GHHW-822"
 epsilonKO
"GHHW-821"


$`FAES1166018-WGS`
$`FAES1166018-WGS`$`TUSNK-912`
    alphaKO     gammaKO
"GSNK-1356" "GSNK-1355"

$`FAES1166018-WGS`$`TUSNK-3580`
     betaKO
"GSNK-5462"

$`FAES1166018-WGS`$`TUSNK-3021`
       aKO         cKO         bKO     deltaKO
"GSNK-4536" "GSNK-4535" "GSNK-4534" "GSNK-4533"

$`FAES1166018-WGS`$`TUSNK-3579`
  epsilonKO
"GSNK-5461"


$FALN326424
```

```
$FALN326424$`TUJ82-3496`
       aKO         cKO         bKO     deltaKO     alphaKO     gammaKO 
"GJ82-5837" "GJ82-5836" "GJ82-5835" "GJ82-5834" "GJ82-5833" "GJ82-5832" 
     betaKO   epsilonKO 
"GJ82-5831" "GJ82-5830" 


$FAUR767434
$FAUR767434$`TULDK-103`
 epsilonKO     betaKO    gammaKO    alphaKO    deltaKO        bKO        cKO 
"GLDK-203" "GLDK-202" "GLDK-201" "GLDK-200" "GLDK-199" "GLDK-198" "GLDK-197" 

$FAUR767434$`TULDK-102`
       aKO 
"GLDK-196" 


$FBAC531844
$FBAC531844$`TUHFZ-461`
   gammaKO    alphaKO    deltaKO        bKO        cKO        aKO 
"GHFZ-830" "GHFZ-829" "GHFZ-828" "GHFZ-827" "GHFZ-826" "GHFZ-825" 

$FBAC531844$`TUHFZ-745`
  epsilonKO      betaKO 
"GHFZ-1294" "GHFZ-1293" 


$MSP313603
$MSP313603$`TUH3X-730`
       aKO         cKO         bKO     deltaKO     alphaKO     gammaKO 
"GH3X-1399" "GH3X-1398" "GH3X-1397" "GH3X-1396" "GH3X-1395" "GH3X-1394" 

$MSP313603$`TUH3X-909`
  epsilonKO      betaKO 
"GH3X-1742" "GH3X-1741" 


$FBAL550540
$FBAL550540$`TUHY2-1987`
       aKO         cKO         bKO     deltaKO     alphaKO     gammaKO 
"GHY2-3937" "GHY2-3936" "GHY2-3935" "GHY2-3934" "GHY2-3933" "GHY2-3932" 
     betaKO   epsilonKO 
"GHY2-3931" "GHY2-3930" 


$FBRA1034807
$FBRA1034807$`TUHGD-72`
      aKO        cKO        bKO    deltaKO    alphaKO    gammaKO 
"GHGD-147" "GHGD-146" "GHGD-145" "GHGD-144" "GHGD-143" "GHGD-142" 

$FBRA1034807$`TUHGD-1570`
     betaKO   epsilonKO 
"GHGD-2765" "GHGD-2764" 
```

```
$FNOV984129
$FNOV984129$`TULDD-846`
       aKO         cKO         bKO     deltaKO     alphaKO     gammaKO 
"GLDD-1733" "GLDD-1732" "GLDD-1731" "GLDD-1730" "GLDD-1729" "GLDD-1728" 
     betaKO   epsilonKO 
"GLDD-1727" "GLDD-1726" 


$MEXT661410
$MEXT661410$`TUJA1-1354`
    deltaKO     alphaKO     gammaKO      betaKO   epsilonKO 
"GJA1-2081" "GJA1-2080" "GJA1-2079" "GJA1-2078" "GJA1-2076" 

$MEXT661410$`TUJA1-2471`
        aKO         cKO        bKO2        bKO1 
"GJA1-3885" "GJA1-3884" "GJA1-3883" "GJA1-3882" 


$`HCIN1206745-WGS`
$`HCIN1206745-WGS`$`TUSP1-547`
  epsilonKO      betaKO     gammaKO     alphaKO     deltaKO        bKO2 
"GSP1-1314" "GSP1-1313" "GSP1-1312" "GSP1-1311" "GSP1-1310" "GSP1-1309" 
       bKO1 
"GSP1-1308" 

$`HCIN1206745-WGS`$`TUSP1-166`
       cKO 
"GSP1-344" 

$`HCIN1206745-WGS`$`TUSP1-225`
       aKO 
"GSP1-488" 


$NHAM323097
$NHAM323097$`TUHP7-693`
   deltaKO    alphaKO    gammaKO     betaKO  epsilonKO 
"GHP7-542" "GHP7-541" "GHP7-540" "GHP7-538" "GHP7-537" 

$NHAM323097$`TUHP7-522`
       aKO        cKO 
"GHP7-273" "GHP7-272" 

$NHAM323097$`TUHP7-521`
      bKO2       bKO1 
"GHP7-271" "GHP7-270" 


$FNOV676032
$FNOV676032$`TULDF-843`
       aKO         cKO         bKO     deltaKO     alphaKO     gammaKO 
"GLDF-1755" "GLDF-1754" "GLDF-1753" "GLDF-1752" "GLDF-1751" "GLDF-1750" 
     betaKO   epsilonKO 
```

```
"GLDF-1749" "GLDF-1748"


$FCOL1041826
$FCOL1041826$`TUHZN-969`
       aKO         cKO         bKO     deltaKO     alphaKO     gammaKO
"GHZN-1662" "GHZN-1661" "GHZN-1660" "GHZN-1659" "GHZN-1658" "GHZN-1657"

$FCOL1041826$`TUHZN-1484`
  epsilonKO      betaKO
"GHZN-2601" "GHZN-2600"


$FIND1094466
$FIND1094466$`TULDB-1354`
       aKO         cKO         bKO     deltaKO     alphaKO     gammaKO
"GLDB-2553" "GLDB-2552" "GLDB-2551" "GLDB-2550" "GLDB-2549" "GLDB-2548"

$FIND1094466$`TULDB-1339`
     betaKO   epsilonKO
"GLDB-2511" "GLDB-2510"


$FJOH376686
$FJOH376686$`TUIXN-627`
    gammaKO     alphaKO     deltaKO         bKO         cKO         aKO
"GIXN-1082" "GIXN-1081" "GIXN-1080" "GIXN-1079" "GIXN-1078" "GIXN-1077"

$FJOH376686$`TUIXN-475`
    betaKO
"GIXN-840"

$FJOH376686$`TUIXN-474`
 epsilonKO
"GIXN-839"


$FLIT880071
$FLIT880071$`TULDC-2181`
       cKO         bKO     deltaKO     alphaKO     gammaKO
"GLDC-2979" "GLDC-2978" "GLDC-2977" "GLDC-2976" "GLDC-2975"

$FLIT880071$`TULDC-491`
    betaKO  epsilonKO
"GLDC-684" "GLDC-683"

$FLIT880071$`TULDC-2182`
       aKO
"GLDC-2980"


$FNOA1163389
$FNOA1163389$`TULDE-290`
 epsilonKO     betaKO    gammaKO    alphaKO    deltaKO        bKO        cKO
```

```
"GLDE-572" "GLDE-571" "GLDE-570" "GLDE-569" "GLDE-568" "GLDE-567" "GLDE-566"
       aKO
"GLDE-565"



$`FNUC469604-WGS`
$`FNUC469604-WGS`$`TUSO2-661`
  epsilonKO      betaKO     gammaKO     alphaKO     deltaKO         bKO
"GSO2-1577" "GSO2-1576" "GSO2-1575" "GSO2-1574" "GSO2-1573" "GSO2-1572"
        cKO         aKO
"GSO2-1571" "GSO2-1570"



$FNOD381764
$FNOD381764$`TUC5M-144`
       aKO        cKO        bKO    deltaKO    alphaKO    gammaKO     betaKO
"GC5M-334" "GC5M-333" "GC5M-332" "GC5M-331" "GC5M-330" "GC5M-329" "GC5M-328"
 epsilonKO
"GC5M-327"



$`FNUC190304-WGS`
$`FNUC190304-WGS`$`TUSNP-456`
       cKO        bKO    deltaKO    alphaKO    gammaKO
"GSNP-960" "GSNP-959" "GSNP-958" "GSNP-957" "GSNP-956"

$`FNUC190304-WGS`$`TUSNP-455`
    betaKO  epsilonKO
"GSNP-955" "GSNP-954"

$`FNUC190304-WGS`$`TUSNP-457`
       aKO
"GSNP-961"



$`FPRA657322-WGS`
$`FPRA657322-WGS`$`TUSNI-717`
       aKO1        cKO1        bKO1     deltaKO    alphaKO1    gammaKO1
"GSNI-1185" "GSNI-1184" "GSNI-1183" "GSNI-1182" "GSNI-1181" "GSNI-1180"
    betaKO1  epsilonKO1
"GSNI-1179" "GSNI-1178"

$`FPRA657322-WGS`$`TUSNI-1347`
 epsilonKO2     betaKO2    gammaKO2    alphaKO2        bKO2        cKO2
"GSNI-2337" "GSNI-2336" "GSNI-2335" "GSNI-2334" "GSNI-2333" "GSNI-2332"
       aKO2
"GSNI-2331"



$AVIT311402
$AVIT311402$`TUH2Y-3043`
    deltaKO     alphaKO     gammaKO      betaKO   epsilonKO
"GH2Y-3090" "GH2Y-3089" "GH2Y-3088" "GH2Y-3087" "GH2Y-3086"
```

$AVIT311402$`TUH2Y-1626`
bKO2 bKO1 cKO aKO
"GH2Y-714" "GH2Y-713" "GH2Y-712" "GH2Y-711"

$MSP754477
$MSP754477$`TULGI-884`
aKO cKO bKO deltaKO alphaKO gammaKO
"GLGI-1911" "GLGI-1910" "GLGI-1909" "GLGI-1908" "GLGI-1907" "GLGI-1906"
betaKO epsilonKO
"GLGI-1905" "GLGI-1904"

$FPEN771875
$FPEN771875$`TULDA-269`
aKO cKO bKO deltaKO alphaKO gammaKO betaKO
"GLDA-655" "GLDA-654" "GLDA-653" "GLDA-652" "GLDA-651" "GLDA-650" "GLDA-649"

$FPEN771875$`TULDA-268`
epsilonKO
"GLDA-648"

$FPHI484022
$FPHI484022$`TUHVB-456`
epsilonKO betaKO gammaKO alphaKO deltaKO bKO cKO
"GHVB-987" "GHVB-986" "GHVB-985" "GHVB-984" "GHVB-983" "GHVB-982" "GHVB-981"
aKO
"GHVB-980"

$`FPRA718252-WGS`
$`FPRA718252-WGS`$`TUSNH-40`
epsilonKO1 betaKO1 gammaKO1 alphaKO1 bKO1 cKO1 aKO1
"GSNH-111" "GSNH-110" "GSNH-109" "GSNH-108" "GSNH-107" "GSNH-106" "GSNH-105"

$`FPRA718252-WGS`$`TUSNH-702`
epsilonKO2 betaKO2 gammaKO2 alphaKO2 deltaKO bKO2
"GSNH-1233" "GSNH-1232" "GSNH-1231" "GSNH-1230" "GSNH-1229" "GSNH-1228"
cKO2 aKO2
"GSNH-1227" "GSNH-1226"

$FSP106370
$FSP106370$`TUI1F-2205`
alphaKO gammaKO
"GI1F-3757" "GI1F-3756"

$FSP106370$`TUI1F-2204`
betaKO epsilonKO
"GI1F-3755" "GI1F-3754"

$FSP106370$`TUI1F-2206`
cKO bKO deltaKO

"GI1F-3760" "GI1F-3759" "GI1F-3758"

$FSP106370$`TUI1F-2207`
aKO
"GI1F-3761"

$FSP298653
$FSP298653$`TUHPI-617`
epsilonKO betaKO gammaKO alphaKO deltaKO bKO
"GHPI-1039" "GHPI-1038" "GHPI-1037" "GHPI-1036" "GHPI-1035" "GHPI-1034"
cKO aKO
"GHPI-1033" "GHPI-1032"

$FSP298654
$FSP298654$`TUHN6-584`
epsilonKO betaKO gammaKO alphaKO deltaKO bKO cKO
"GHN6-955" "GHN6-954" "GHN6-953" "GHN6-952" "GHN6-951" "GHN6-950" "GHN6-949"
aKO
"GHN6-948"

$FSP573569
$FSP573569$`TUHZ6-273`
epsilonKO betaKO gammaKO alphaKO deltaKO bKO cKO
"GHZ6-576" "GHZ6-575" "GHZ6-574" "GHZ6-573" "GHZ6-572" "GHZ6-571" "GHZ6-570"
aKO
"GHZ6-569"

$FSIN717231
$FSIN717231$`TUI70-715`
bKO2 bKO1 deltaKO alphaKO gammaKO betaKO
"GI70-1727" "GI70-1726" "GI70-1725" "GI70-1724" "GI70-1723" "GI70-1722"
epsilonKO
"GI70-1721"

$FSIN717231$`TUI70-197`
cKO aKO
"GI70-509" "GI70-508"

$FSYM656024
$FSYM656024$`TUHLT-2278`
aKO cKO bKO deltaKO alphaKO gammaKO
"GHLT-3879" "GHLT-3878" "GHLT-3877" "GHLT-3876" "GHLT-3875" "GHLT-3874"
betaKO epsilonKO
"GHLT-3873" "GHLT-3872"

$PAMO264201
$PAMO264201$`TUH0M-1031`
cKO bKO deltaKO alphaKO gammaKO betaKO

"GH0M-1709" "GH0M-1708" "GH0M-1707" "GH0M-1706" "GH0M-1705" "GH0M-1704"
epsilonKO
"GH0M-1703"

$PAMO264201$`TUH0M-1032`
aKO
"GH0M-1710"

$FTUL458234
$FTUL458234$`TUH31-845`
aKO cKO bKO deltaKO alphaKO gammaKO
"GH31-1738" "GH31-1737" "GH31-1736" "GH31-1735" "GH31-1734" "GH31-1733"
betaKO epsilonKO
"GH31-1732" "GH31-1731"

$`MELS1064535-WGS`
$`MELS1064535-WGS`$`TUSQ9-891`
aKO cKO bKO deltaKO alphaKO gammaKO
"GSQ9-1977" "GSQ9-1976" "GSQ9-1975" "GSQ9-1974" "GSQ9-1973" "GSQ9-1972"
betaKO epsilonKO
"GSQ9-1971" "GSQ9-1970"

$FTUL393115
$FTUL393115$`TUJUT-25`
epsilonKO betaKO gammaKO alphaKO deltaKO bKO cKO aKO
"GJUT-66" "GJUT-65" "GJUT-64" "GJUT-63" "GJUT-62" "GJUT-61" "GJUT-60" "GJUT-59"

$FTUL1001542
$FTUL1001542$`TULDJ-20`
epsilonKO betaKO gammaKO alphaKO deltaKO bKO cKO aKO
"GLDJ-62" "GLDJ-61" "GLDJ-60" "GLDJ-59" "GLDJ-58" "GLDJ-57" "GLDJ-56" "GLDJ-55"

$AMAR320483
$AMAR320483$`TUIWE-491`
deltaKO alphaKO
"GIWE-857" "GIWE-856"

$AMAR320483$`TUIWE-291`
betaKO epsilonKO
"GIWE-483" "GIWE-482"

$AMAR320483$`TUIWE-273`
gammaKO
"GIWE-450"

$AMAR320483$`TUIWE-467`
bKO2 bKO1 cKO
"GIWE-811" "GIWE-810" "GIWE-809"

```
$AMAR320483$`TUIWE-602`
       aKO 
"GIWE-807" 


$AMAC1004788
$AMAC1004788$`TUL7V-2246`
        aKO         cKO         bKO     deltaKO     alphaKO     gammaKO 
"GL7V-3965" "GL7V-3964" "GL7V-3963" "GL7V-3962" "GL7V-3961" "GL7V-3960" 
     betaKO   epsilonKO 
"GL7V-3959" "GL7V-3958" 


$`AMAC1300253-WGS`
$`AMAC1300253-WGS`$`TUSF0-2023`
        aKO         cKO         bKO     deltaKO     alphaKO      betaKO 
"GSF0-3415" "GSF0-3414" "GSF0-3413" "GSF0-3412" "GSF0-3411" "GSF0-3410" 
  epsilonKO 
"GSF0-3409" 

$`AMAC1300253-WGS`$noTU
gammaKO 
     NA 


$AMIR446462
$AMIR446462$`TUH7R-3345`
        aKO         cKO         bKO     deltaKO     alphaKO     gammaKO 
"GH7R-6207" "GH7R-6206" "GH7R-6205" "GH7R-6204" "GH7R-6203" "GH7R-6202" 
     betaKO   epsilonKO 
"GH7R-6201" "GH7R-6200" 


$FTUL393011
$FTUL393011$`TUHFN-845`
        aKO         cKO         bKO     deltaKO     alphaKO     gammaKO 
"GHFN-1782" "GHFN-1781" "GHFN-1780" "GHFN-1779" "GHFN-1778" "GHFN-1777" 
     betaKO   epsilonKO 
"GHFN-1776" "GHFN-1775" 


$FTUL351581
$FTUL351581$`TULDH-810`
        aKO         cKO         bKO     deltaKO     alphaKO     gammaKO 
"GLDH-1660" "GLDH-1659" "GLDH-1658" "GLDH-1657" "GLDH-1656" "GLDH-1655" 
     betaKO   epsilonKO 
"GLDH-1654" "GLDH-1653" 


$ASP137722
$ASP137722$`TUBYD-3414`
    deltaKO     alphaKO 
"GBYD-2537" "GBYD-2536" 
```

```
$ASP137722$`TUBYD-3412`
    betaKO1
"GBYD-2534"

$ASP137722$`TUBYD-1002`
   gammaKO2        cKO2        aKO2  epsilonKO2     betaKO2
"GBYD-4824" "GBYD-4822" "GBYD-4821" "GBYD-4818" "GBYD-4817"

$ASP137722$`TUBYD-3413`
   gammaKO1
"GBYD-2535"

$ASP137722$`TUBYD-3411`
 epsilonKO1
"GBYD-2533"

$ASP137722$`TUBYD-2359`
      cKO1
"GBYD-754"

$ASP137722$`TUBYD-2360`
      aKO1
"GBYD-755"

$ASP137722$`TUBYD-2358`
      bKO2       bKO1
"GBYD-753" "GBYD-752"


$FTUL376619
$FTUL376619$`TUI22-934`
        aKO         cKO         bKO     deltaKO     alphaKO     gammaKO
"GI22-1850" "GI22-1849" "GI22-1848" "GI22-1847" "GI22-1846" "GI22-1845"
     betaKO   epsilonKO
"GI22-1844" "GI22-1843"


$FTUL441952
$FTUL441952$`TUIY0-55`
 epsilonKO     betaKO    gammaKO    alphaKO    deltaKO        bKO        cKO
"GIY0-129" "GIY0-128" "GIY0-127" "GIY0-126" "GIY0-125" "GIY0-124" "GIY0-123"
       aKO
"GIY0-122"


$MVER666681
$MVER666681$`TUHRP-1528`
        aKO         cKO         bKO     deltaKO     alphaKO     gammaKO
"GHRP-2845" "GHRP-2844" "GHRP-2843" "GHRP-2842" "GHRP-2841" "GHRP-2840"
     betaKO   epsilonKO
"GHRP-2839" "GHRP-2838"


$FNOV401614
```

```
$FNOV401614$`TUC4M-781`
       aKO        cKO        bKO    deltaKO    alphaKO    gammaKO 
"GC4M-1651" "GC4M-1650" "GC4M-1649" "GC4M-1648" "GC4M-1647" "GC4M-1646" 
    betaKO  epsilonKO 
"GC4M-1645" "GC4M-1644" 


$FTUL510831
$FTUL510831$`TULDG-24`
epsilonKO    betaKO  gammaKO   alphaKO   deltaKO       bKO       cKO       aKO 
"GLDG-68" "GLDG-67" "GLDG-66" "GLDG-65" "GLDG-64" "GLDG-63" "GLDG-62" "GLDG-60" 


$`FTUL1232394-WGS`
$`FTUL1232394-WGS`$`TUSO0-877`
       aKO        cKO        bKO    deltaKO    alphaKO    gammaKO 
"GSO0-1740" "GSO0-1739" "GSO0-1738" "GSO0-1737" "GSO0-1736" "GSO0-1735" 
    betaKO  epsilonKO 
"GSO0-1734" "GSO0-1733" 


$FTUL1001534
$FTUL1001534$`TULDI-20`
epsilonKO    betaKO  gammaKO   alphaKO   deltaKO       bKO       cKO       aKO 
"GLDI-62" "GLDI-61" "GLDI-60" "GLDI-59" "GLDI-58" "GLDI-57" "GLDI-56" "GLDI-55" 


$FTUL177416
$FTUL177416$`TUNBP-25`
epsilonKO    betaKO  gammaKO   alphaKO   deltaKO       bKO       cKO       aKO 
"GNBP-66" "GNBP-65" "GNBP-64" "GNBP-63" "GNBP-62" "GNBP-61" "GNBP-60" "GNBP-59" 


$FTUL418136
$FTUL418136$`TUHXJ-53`
 epsilonKO     betaKO   gammaKO    alphaKO    deltaKO        bKO        cKO 
"GHXJ-132" "GHXJ-131" "GHXJ-130" "GHXJ-129" "GHXJ-128" "GHXJ-127" "GHXJ-126" 
       aKO 
"GHXJ-125" 


$`FNUC469607-WGS`
$`FNUC469607-WGS`$`TUSNO-291`
       aKO        cKO        bKO    deltaKO    alphaKO    gammaKO     betaKO 
"GSNO-632" "GSNO-631" "GSNO-630" "GSNO-629" "GSNO-628" "GSNO-627" "GSNO-626" 
 epsilonKO 
"GSNO-625" 


$LMON552536
$LMON552536$`TUIW4-39`
epsilonKO1    betaKO1   gammaKO1   alphaKO1   deltaKO1        bKO       cKO1 
 "GIW4-67"  "GIW4-66"  "GIW4-65"  "GIW4-64"  "GIW4-63"  "GIW4-62"  "GIW4-61" 
       aKO 
```

"GIW4-60"

$LMON552536$`TUIW4-1334`
cKO2 deltaKO2 alphaKO2 gammaKO2 betaKO2 epsilonKO2
"GIW4-2586" "GIW4-2585" "GIW4-2584" "GIW4-2583" "GIW4-2582" "GIW4-2581"

$GSP983545
$GSP983545$`TUH3Q-2611`
aKO cKO bKO deltaKO alphaKO gammaKO
"GH3Q-4344" "GH3Q-4343" "GH3Q-4342" "GH3Q-4341" "GH3Q-4340" "GH3Q-4339"
betaKO epsilonKO
"GH3Q-4338" "GH3Q-4337"

$GANA1005058
$GANA1005058$`TUHKG-633`
aKO cKO bKO deltaKO alphaKO gammaKO
"GHKG-1242" "GHKG-1241" "GHKG-1240" "GHKG-1239" "GHKG-1238" "GHKG-1237"
betaKO epsilonKO
"GHKG-1236" "GHKG-1235"

$GAUR379066
$GAUR379066$`TUI3W-346`
epsilonKO betaKO gammaKO alphaKO
"GI3W-725" "GI3W-724" "GI3W-723" "GI3W-722"

$GAUR379066$`TUI3W-1158`
aKO cKO bKO deltaKO
"GI3W-2541" "GI3W-2540" "GI3W-2539" "GI3W-2538"

$MGLU582744
$MGLU582744$`TUHXQ-1466`
aKO cKO bKO deltaKO alphaKO gammaKO
"GHXQ-2877" "GHXQ-2876" "GHXQ-2875" "GHXQ-2874" "GHXQ-2873" "GHXQ-2872"
betaKO epsilonKO
"GHXQ-2871" "GHXQ-2870"

$GBET391165
$GBET391165$`TUHON-1008`
deltaKO alphaKO gammaKO betaKO epsilonKO
"GHON-2109" "GHON-2108" "GHON-2107" "GHON-2106" "GHON-2105"

$GBET391165$`TUHON-925`
bKO2 bKO1 cKO aKO
"GHON-1917" "GHON-1916" "GHON-1915" "GHON-1914"

$GBEM404380
$GBEM404380$`TUHFR-2249`
bKO2 bKO1 deltaKO alphaKO gammaKO betaKO

"GHFR-4021" "GHFR-4020" "GHFR-4019" "GHFR-4018" "GHFR-4017" "GHFR-4016"
epsilonKO
"GHFR-4015"

$GBEM404380$`TUHFR-2238`
cKO aKO
"GHFR-3998" "GHFR-3997"

$GBRO526226
$GBRO526226$`TUHJF-1058`
epsilonKO betaKO gammaKO alphaKO deltaKO bKO
"GHJF-1942" "GHJF-1941" "GHJF-1940" "GHJF-1939" "GHJF-1938" "GHJF-1937"
cKO aKO
"GHJF-1936" "GHJF-1935"

$GCAP395494
$GCAP395494$`TUHXI-1341`
aKO cKO bKO deltaKO alphaKO gammaKO
"GHXI-3000" "GHXI-2999" "GHXI-2998" "GHXI-2997" "GHXI-2996" "GHXI-2995"
betaKO epsilonKO
"GHXI-2994" "GHXI-2993"

$GSP691437
$GSP691437$`TUI2V-1812`
aKO cKO bKO deltaKO alphaKO gammaKO
"GI2V-3467" "GI2V-3466" "GI2V-3465" "GI2V-3464" "GI2V-3463" "GI2V-3462"
betaKO epsilonKO
"GI2V-3461" "GI2V-3460"

$GDIA272568
$GDIA272568$`TUJPS-397`
epsilonKO betaKO gammaKO alphaKO deltaKO
"GJPS-707" "GJPS-706" "GJPS-705" "GJPS-704" "GJPS-703"

$GDIA272568$`TUJPS-654`
aKO cKO bKO2 bKO1
"GJPS-1199" "GJPS-1198" "GJPS-1197" "GJPS-1196"

$PMAR167539
$PMAR167539$`TUJN2-884`
aKO cKO bKO2 bKO1 deltaKO alphaKO
"GJN2-1645" "GJN2-1644" "GJN2-1643" "GJN2-1642" "GJN2-1641" "GJN2-1640"
gammaKO
"GJN2-1639"

$PMAR167539$`TUJN2-881`
epsilonKO betaKO
"GJN2-1628" "GJN2-1627"

```
$GSP443143
$GSP443143$`TUHZL-2492`
      bKO2        bKO1     deltaKO     alphaKO     gammaKO      betaKO 
"GHZL-4496" "GHZL-4495" "GHZL-4494" "GHZL-4493" "GHZL-4492" "GHZL-4491" 
  epsilonKO 
"GHZL-4490" 

$GSP443143$`TUHZL-2480`
        cKO         aKO 
"GHZL-4472" "GHZL-4471" 


$GSP1173025
$GSP1173025$`TULDM-1279`
      aKO1        cKO1        bKO1    alphaKO1    gammaKO1 
"GLDM-1879" "GLDM-1878" "GLDM-1877" "GLDM-1876" "GLDM-1875" 

$GSP1173025$`TULDM-1608`
      bKO2     deltaKO    alphaKO2    gammaKO2 
"GLDM-2394" "GLDM-2393" "GLDM-2392" "GLDM-2391" 

$GSP1173025$`TULDM-1280`
    betaKO1  epsilonKO1 
"GLDM-1883" "GLDM-1882" 

$GSP1173025$`TULDM-1580`
 epsilonKO2     betaKO2 
"GLDM-2337" "GLDM-2336" 

$GSP1173025$`TULDM-1610`
      aKO2        cKO2 
"GLDM-2397" "GLDM-2396" 

$GSP1173025$`TULDM-1609`
      bKO3 
"GLDM-2395" 


$GSP443144
$GSP443144$`TUHKM-2293`
      bKO2        bKO1     deltaKO     alphaKO     gammaKO      betaKO 
"GHKM-4116" "GHKM-4115" "GHKM-4114" "GHKM-4113" "GHKM-4112" "GHKM-4111" 
  epsilonKO 
"GHKM-4110" 

$GSP443144$`TUHKM-2282`
        cKO         aKO 
"GHKM-4092" "GHKM-4091" 


$GSP316067
$GSP316067$`TUHSV-251`
      bKO2       bKO1    deltaKO    alphaKO    gammaKO     betaKO  epsilonKO 
```

"GHSV-458" "GHSV-457" "GHSV-456" "GHSV-455" "GHSV-454" "GHSV-453" "GHSV-452"

$GSP316067$`TUHSV-254`
aKO cKO
"GHSV-464" "GHSV-463"

$MSP754476
$MSP754476$`TULGH-115`
aKO cKO bKO deltaKO alphaKO gammaKO betaKO
"GLGH-226" "GLGH-225" "GLGH-224" "GLGH-223" "GLGH-222" "GLGH-221" "GLGH-220"
epsilonKO
"GLGH-219"

$GKAU235909
$GKAU235909$`TUJO7-1901`
aKO cKO bKO deltaKO alphaKO gammaKO
"GJO7-3477" "GJO7-3476" "GJO7-3475" "GJO7-3474" "GJO7-3473" "GJO7-3472"
betaKO epsilonKO
"GJO7-3471" "GJO7-3470"

$GLOV398767
$GLOV398767$`TUH32-1488`
bKO2 bKO1 deltaKO alphaKO gammaKO betaKO
"GH32-3225" "GH32-3224" "GH32-3223" "GH32-3222" "GH32-3221" "GH32-3220"
epsilonKO
"GH32-3219"

$GLOV398767$`TUH32-1481`
cKO aKO
"GH32-3193" "GH32-3192"

$GMAL682795
$GMAL682795$`TUHBV-1227`
epsilonKO betaKO gammaKO alphaKO deltaKO bKO2
"GHBV-2069" "GHBV-2068" "GHBV-2067" "GHBV-2066" "GHBV-2065" "GHBV-2064"
bKO1
"GHBV-2063"

$GMAL682795$`TUHBV-851`
cKO aKO
"GHBV-1397" "GHBV-1396"

$GSP581103
$GSP581103$`TUHT2-2042`
aKO cKO bKO deltaKO alphaKO gammaKO
"GHT2-3822" "GHT2-3821" "GHT2-3820" "GHT2-3819" "GHT2-3818" "GHT2-3817"
betaKO epsilonKO
"GHT2-3816" "GHT2-3815"

```
$GNIT1085623
$GNIT1085623$`TUJU2-1865`
      aKO1       cKO1       bKO1    alphaKO1    gammaKO1
"GJU2-3363" "GJU2-3362" "GJU2-3361" "GJU2-3360" "GJU2-3359"

$GNIT1085623$`TUJU2-2066`
      aKO2       cKO2       bKO2     deltaKO    alphaKO2    gammaKO2
"GJU2-3703" "GJU2-3702" "GJU2-3701" "GJU2-3700" "GJU2-3699" "GJU2-3698"
   betaKO2  epsilonKO2
"GJU2-3697" "GJU2-3696"

$GNIT1085623$`TUJU2-1866`
   betaKO1  epsilonKO1
"GJU2-3367" "GJU2-3366"


$PMAR59919
$PMAR59919$`TUJMQ-731`
       aKO         cKO        bKO2        bKO1     deltaKO     alphaKO
"GJMQ-1496" "GJMQ-1495" "GJMQ-1494" "GJMQ-1493" "GJMQ-1492" "GJMQ-1491"
   gammaKO
"GJMQ-1490"

$PMAR59919$`TUJMQ-726`
  epsilonKO      betaKO
"GJMQ-1479" "GJMQ-1478"


$GOBS526225
$GOBS526225$`TUI00-2318`
       aKO         cKO         bKO     deltaKO     alphaKO     gammaKO
"GI00-4191" "GI00-4190" "GI00-4189" "GI00-4188" "GI00-4187" "GI00-4186"
    betaKO   epsilonKO
"GI00-4185" "GI00-4183"


$GOXY1224746
$GOXY1224746$`TULDO-560`
   deltaKO    alphaKO    gammaKO     betaKO  epsilonKO
"GLDO-800" "GLDO-799" "GLDO-798" "GLDO-797" "GLDO-796"

$GOXY1224746$`TULDO-658`
      bKO2        bKO1         cKO         aKO
"GLDO-1007" "GLDO-1006" "GLDO-1005" "GLDO-1004"


$GOXY290633
$GOXY290633$`TUHB3-837`
 epsilonKO1     betaKO1    gammaKO1    alphaKO1     deltaKO
"GHB3-1312" "GHB3-1311" "GHB3-1310" "GHB3-1309" "GHB3-1308"

$GOXY290633$`TUHB3-1279`
   gammaKO2    alphaKO2        bKO3        cKO2        aKO2  epsilonKO2
```

```
"GHB3-2173" "GHB3-2172" "GHB3-2171" "GHB3-2170" "GHB3-2169" "GHB3-2166"
   betaKO2
"GHB3-2165"

$GOXY290633$`TUHB3-738`
      aKO1        cKO1        bKO2        bKO1
"GHB3-1111" "GHB3-1110" "GHB3-1109" "GHB3-1108"


$GPRO83406
$GPRO83406$`TUIWA-2162`
       aKO         cKO         bKO     deltaKO     alphaKO     gammaKO
"GIWA-3856" "GIWA-3855" "GIWA-3854" "GIWA-3853" "GIWA-3852" "GIWA-3851"
    betaKO   epsilonKO
"GIWA-3850" "GIWA-3849"


$GPOL1112204
$GPOL1112204$`TUJWY-1107`
  epsilonKO      betaKO     gammaKO     alphaKO         bKO         cKO
"GJWY-1808" "GJWY-1807" "GJWY-1806" "GJWY-1805" "GJWY-1803" "GJWY-1802"
       aKO
"GJWY-1801"

$GPOL1112204$noTU
deltaKO
    NA


$MSP887061
$MSP887061$`TUHJT-1411`
       bKO     deltaKO     alphaKO     gammaKO      betaKO   epsilonKO
"GHJT-2762" "GHJT-2761" "GHJT-2760" "GHJT-2759" "GHJT-2758" "GHJT-2757"

$MSP887061$`TUHJT-1412`
       aKO
"GHJT-2764"

$MSP887061$noTU
cKO
 NA


$`GPSY1129794-WGS`
$`GPSY1129794-WGS`$`TUSNU-3305`
       aKO         cKO         bKO     deltaKO     alphaKO     gammaKO
"GSNU-5679" "GSNU-5678" "GSNU-5677" "GSNU-5676" "GSNU-5675" "GSNU-5674"
    betaKO   epsilonKO
"GSNU-5673" "GSNU-5672"


$AMAC1004785
$AMAC1004785$`TUL7U-2192`
       aKO         cKO         bKO     deltaKO     alphaKO     gammaKO
```

"GL7U-3845" "GL7U-3844" "GL7U-3843" "GL7U-3842" "GL7U-3841" "GL7U-3840"
betaKO epsilonKO
"GL7U-3839" "GL7U-3838"

$GTHE1111068
$GTHE1111068$`TUJY8-1958`
aKO cKO bKO deltaKO alphaKO gammaKO
"GJY8-3857" "GJY8-3856" "GJY8-3855" "GJY8-3854" "GJY8-3853" "GJY8-3852"
betaKO epsilonKO
"GJY8-3851" "GJY8-3850"

$GTHE634956
$GTHE634956$`TUHH3-2065`
aKO cKO bKO deltaKO alphaKO gammaKO
"GHH3-3902" "GHH3-3901" "GHH3-3900" "GHH3-3899" "GHH3-3898" "GHH3-3897"
betaKO epsilonKO
"GHH3-3896" "GHH3-3895"

$`PMAR74547-WGS`
$`PMAR74547-WGS`$`TUSSL-844`
aKO cKO bKO2 bKO1 deltaKO alphaKO
"GSSL-1505" "GSSL-1504" "GSSL-1503" "GSSL-1502" "GSSL-1501" "GSSL-1500"
gammaKO
"GSSL-1499"

$`PMAR74547-WGS`$`TUSSL-840`
epsilonKO betaKO
"GSSL-1485" "GSSL-1484"

$GTHE420246
$GTHE420246$`TUIXT-1836`
bKO deltaKO alphaKO gammaKO betaKO epsilonKO
"GIXT-3401" "GIXT-3400" "GIXT-3399" "GIXT-3398" "GIXT-3397" "GIXT-3396"

$GTHE420246$`TUIXT-1837`
aKO cKO
"GIXT-3403" "GIXT-3402"

$GURA351605
$GURA351605$`TUI6A-2575`
epsilonKO betaKO gammaKO alphaKO deltaKO bKO2
"GI6A-4319" "GI6A-4318" "GI6A-4317" "GI6A-4316" "GI6A-4315" "GI6A-4314"
bKO1
"GI6A-4313"

$GURA351605$`TUI6A-2568`
cKO
"GI6A-4304"

$GURA351605$`TUI6A-2567`
aKO
"GI6A-4303"

$GVAG553190
$GVAG553190$`TUJPT-92`
betaKO gammaKO alphaKO deltaKO bKO cKO aKO
"GJPT-148" "GJPT-147" "GJPT-146" "GJPT-145" "GJPT-144" "GJPT-143" "GJPT-142"

$GVAG553190$`TUJPT-93`
epsilonKO
"GJPT-149"

$GVAG525284
$GVAG525284$`TUI3V-2`
aKO cKO bKO deltaKO alphaKO gammaKO betaKO epsilonKO
"GI3V-9" "GI3V-8" "GI3V-7" "GI3V-6" "GI3V-5" "GI3V-4" "GI3V-3" "GI3V-2"

$GVAG1009464
$GVAG1009464$`TULDL-71`
epsilonKO betaKO gammaKO alphaKO deltaKO bKO cKO
"GLDL-139" "GLDL-138" "GLDL-137" "GLDL-136" "GLDL-135" "GLDL-134" "GLDL-133"
aKO
"GLDL-132"

$GVIO251221
$GVIO251221$`TUH9A-1665`
aKO cKO bKO2 bKO1 deltaKO alphaKO
"GH9A-2946" "GH9A-2945" "GH9A-2944" "GH9A-2943" "GH9A-2942" "GH9A-2941"

$GVIO251221$`TUH9A-1478`
betaKO
"GH9A-2602"

$GVIO251221$`TUH9A-2521`
gammaKO
"GH9A-4367"

$GVIO251221$`TUH9A-1477`
epsilonKO
"GH9A-2600"

$APAS634453
$APAS634453$`TUL7D-296`
epsilonKO betaKO gammaKO alphaKO deltaKO
"GL7D-121" "GL7D-120" "GL7D-119" "GL7D-118" "GL7D-117"

$APAS634453$`TUL7D-1516`
aKO cKO

"GL7D-2467" "GL7D-2466"

$APAS634453$`TUL7D-1515`
bKO2 bKO1
"GL7D-2465" "GL7D-2464"

$CSP266779
$CSP266779$`TUI09-1971`
epsilonKO betaKO gammaKO alphaKO deltaKO
"GI09-3276" "GI09-3275" "GI09-3274" "GI09-3273" "GI09-3272"

$CSP266779$`TUI09-609`
bKO2 bKO1 cKO aKO
"GI09-706" "GI09-705" "GI09-704" "GI09-703"

$GSP471223
$GSP471223$`TUH2C-1921`
aKO cKO bKO deltaKO alphaKO gammaKO
"GH2C-3423" "GH2C-3422" "GH2C-3421" "GH2C-3420" "GH2C-3419" "GH2C-3418"
betaKO epsilonKO
"GH2C-3417" "GH2C-3416"

$GXYL634177
$GXYL634177$`TUHBT-977`
deltaKO alphaKO gammaKO betaKO epsilonKO
"GHBT-1446" "GHBT-1445" "GHBT-1444" "GHBT-1443" "GHBT-1442"

$GXYL634177$`TUHBT-1174`
aKO cKO bKO2 bKO1
"GHBT-1826" "GHBT-1825" "GHBT-1824" "GHBT-1823"

$PPRO338966
$PPRO338966$`TUHL0-443`
epsilonKO1 betaKO1 gammaKO1 alphaKO1 deltaKO1 bKO2 bKO1
"GHL0-611" "GHL0-610" "GHL0-609" "GHL0-608" "GHL0-607" "GHL0-606" "GHL0-605"

$PPRO338966$`TUHL0-559`
betaKO2 epsilonKO2 aKO2 cKO2 bKO3 alphaKO2 gammaKO2
"GHL0-861" "GHL0-860" "GHL0-858" "GHL0-857" "GHL0-856" "GHL0-855" "GHL0-854"

$PPRO338966$`TUHL0-924`
epsilonKO3 betaKO3 gammaKO3 alphaKO3 deltaKO2 bKO5
"GHL0-1534" "GHL0-1533" "GHL0-1532" "GHL0-1531" "GHL0-1530" "GHL0-1529"
bKO4
"GHL0-1528"

$PPRO338966$`TUHL0-442`
cKO1 aKO1
"GHL0-604" "GHL0-603"

$PPRO338966$`TUHL0-923`
cKO3 aKO3
"GHL0-1527" "GHL0-1526"

$GSP550542
$GSP550542$`TUH52-1888`
aKO cKO bKO deltaKO alphaKO gammaKO
"GH52-3567" "GH52-3566" "GH52-3565" "GH52-3564" "GH52-3563" "GH52-3562"
betaKO epsilonKO
"GH52-3561" "GH52-3560"

$GSP544556
$GSP544556$`TUI3L-1898`
aKO cKO bKO deltaKO alphaKO gammaKO
"GI3L-3538" "GI3L-3537" "GI3L-3536" "GI3L-3535" "GI3L-3534" "GI3L-3533"
betaKO epsilonKO
"GI3L-3532" "GI3L-3531"

$PSP481743
$PSP481743$`TUH8K-3623`
aKO cKO bKO deltaKO alphaKO gammaKO
"GH8K-6010" "GH8K-6009" "GH8K-6008" "GH8K-6007" "GH8K-6006" "GH8K-6005"
betaKO epsilonKO
"GH8K-6004" "GH8K-6003"

$HSP65093
$HSP65093$`TULDX-267`
gammaKO1 alphaKO1 deltaKO bKO2
"GLDX-431" "GLDX-430" "GLDX-429" "GLDX-428"

$HSP65093$`TULDX-1569`
alphaKO2 gammaKO2
"GLDX-2549" "GLDX-2548"

$HSP65093$`TULDX-1224`
epsilonKO1 betaKO1
"GLDX-1988" "GLDX-1987"

$HSP65093$`TULDX-1570`
betaKO2 epsilonKO2 aKO2 cKO2 bKO3
"GLDX-2556" "GLDX-2555" "GLDX-2552" "GLDX-2551" "GLDX-2550"

$HSP65093$`TULDX-266`
bKO1 cKO1 aKO1
"GLDX-427" "GLDX-426" "GLDX-425"

$HPAR557723
$HPAR557723$`TUH24-901`
epsilonKO betaKO gammaKO alphaKO deltaKO bKO

"GH24-1627" "GH24-1626" "GH24-1625" "GH24-1624" "GH24-1623" "GH24-1622"
cKO aKO
"GH24-1621" "GH24-1620"

$HARS204773
$HARS204773$`TUJCA-1952`
aKO cKO bKO deltaKO alphaKO gammaKO
"GJCA-3300" "GJCA-3299" "GJCA-3298" "GJCA-3297" "GJCA-3296" "GJCA-3295"
betaKO epsilonKO
"GJCA-3294" "GJCA-3293"

$HHYD656519
$HHYD656519$`TUHYV-220`
epsilonKO betaKO gammaKO alphaKO deltaKO bKO cKO
"GHYV-414" "GHYV-413" "GHYV-412" "GHYV-411" "GHYV-410" "GHYV-409" "GHYV-408"
aKO
"GHYV-405"

$HAUR316274
$HAUR316274$`TUHYA-2611`
gammaKO alphaKO deltaKO bKO
"GHYA-4114" "GHYA-4113" "GHYA-4112" "GHYA-4111"

$HAUR316274$`TUHYA-2612`
betaKO
"GHYA-4115"

$HAUR316274$`TUHYA-2613`
epsilonKO
"GHYA-4116"

$HAUR316274$`TUHYA-2610`
cKO aKO
"GHYA-4110" "GHYA-4109"

$MSP426117
$MSP426117$`TUI2I-4069`
deltaKO alphaKO gammaKO betaKO epsilonKO
"GI2I-6719" "GI2I-6718" "GI2I-6717" "GI2I-6716" "GI2I-6715"

$MSP426117$`TUI2I-4273`
cKO aKO
"GI2I-7028" "GI2I-7027"

$MSP426117$`TUI2I-4274`
bKO2 bKO1
"GI2I-7030" "GI2I-7029"

$HBAL582402

$HBAL582402$`TUHMV-138`
epsilonKO betaKO gammaKO alphaKO deltaKO
"GHMV-156" "GHMV-154" "GHMV-153" "GHMV-152" "GHMV-151"

$HBAL582402$`TUHMV-664`
aKO cKO
"GHMV-1071" "GHMV-1070"

$HBAL582402$`TUHMV-663`
bKO2 bKO1
"GHMV-1069" "GHMV-1068"

$`PPRO298386-WGS`
$`PPRO298386-WGS`$`TUSSB-3352`
aKO1 cKO1 bKO1 deltaKO1 alphaKO1 gammaKO1
"GSSB-3601" "GSSB-3600" "GSSB-3599" "GSSB-3598" "GSSB-3597" "GSSB-3596"
betaKO1 epsilonKO1
"GSSB-3595" "GSSB-3594"

$`PPRO298386-WGS`$`TUSSB-80`
epsilonKO2 betaKO2 gammaKO2 alphaKO2 deltaKO2 bKO2
"GSSB-3743" "GSSB-3742" "GSSB-3741" "GSSB-3740" "GSSB-3739" "GSSB-3738"
cKO2 aKO2
"GSSB-3737" "GSSB-3736"

$`HBIZ1002804-WGS`
$`HBIZ1002804-WGS`$`TUSO5-558`
bKO2 bKO1 deltaKO alphaKO gammaKO betaKO
"GSO5-1495" "GSO5-1494" "GSO5-1493" "GSO5-1492" "GSO5-1491" "GSO5-1490"
epsilonKO
"GSO5-1489"

$`HBIZ1002804-WGS`$`TUSO5-605`
cKO
"GSO5-1610"

$`HBIZ1002804-WGS`$`TUSO5-120`
aKO
"GSO5-287"

$HPYL1163742
$HPYL1163742$`TULEK-473`
bKO2 bKO1 deltaKO alphaKO gammaKO betaKO
"GLEK-1081" "GLEK-1080" "GLEK-1079" "GLEK-1078" "GLEK-1077" "GLEK-1076"
epsilonKO
"GLEK-1075"

$HPYL1163742$`TULEK-515`
cKO
"GLEK-1165"

$HPYL1163742$`TULEK-343`
aKO
"GLEK-770"

$HCET182217
$HCET182217$`TULDW-753`
bKO2 bKO1 deltaKO alphaKO gammaKO betaKO
"GLDW-1679" "GLDW-1678" "GLDW-1677" "GLDW-1676" "GLDW-1675" "GLDW-1674"
epsilonKO
"GLDW-1673"

$HCET182217$`TULDW-297`
cKO
"GLDW-640"

$HCET182217$`TULDW-642`
aKO
"GLDW-1418"

$HCHE349521
$HCHE349521$`TUHAL-536`
betaKO1 epsilonKO1 aKO1 cKO1 bKO1 alphaKO1 gammaKO1
"GHAL-891" "GHAL-890" "GHAL-888" "GHAL-887" "GHAL-886" "GHAL-885" "GHAL-884"

$HCHE349521$`TUHAL-3833`
aKO2 cKO2 bKO2 deltaKO alphaKO2 gammaKO2
"GHAL-6846" "GHAL-6845" "GHAL-6844" "GHAL-6843" "GHAL-6842" "GHAL-6841"
betaKO2 epsilonKO2
"GHAL-6840" "GHAL-6839"

$HCET1163745
$HCET1163745$`TULDY-652`
bKO2 bKO1 deltaKO alphaKO gammaKO betaKO
"GLDY-1472" "GLDY-1471" "GLDY-1470" "GLDY-1469" "GLDY-1468" "GLDY-1467"
epsilonKO
"GLDY-1466"

$HCET1163745$`TULDY-277`
cKO
"GLDY-602"

$HCET1163745$`TULDY-153`
aKO
"GLDY-325"

$HPYL1163743
$HPYL1163743$`TULEH-456`
bKO2 bKO1 deltaKO alphaKO gammaKO betaKO
"GLEH-1060" "GLEH-1059" "GLEH-1058" "GLEH-1057" "GLEH-1056" "GLEH-1055"
epsilonKO

"GLEH-1054"

$HPYL1163743$`TULEH-499`
cKO
"GLEH-1142"

$HPYL1163743$`TULEH-212`
aKO
"GLEH-503"

$HDEF572265
$HDEF572265$`TUJAB-38`
cKO bKO deltaKO alphaKO gammaKO betaKO
"GJAB-2363" "GJAB-2362" "GJAB-2361" "GJAB-2360" "GJAB-2359" "GJAB-2358"
epsilonKO
"GJAB-2357"

$HDEF572265$`TUJAB-39`
aKO
"GJAB-2364"

$HDEN582899
$HDEN582899$`TUIWL-1862`
epsilonKO betaKO gammaKO alphaKO deltaKO
"GIWL-3449" "GIWL-3447" "GIWL-3445" "GIWL-3444" "GIWL-3443"

$HDEN582899$`TUIWL-1613`
bKO2 bKO1 cKO aKO
"GIWL-2929" "GIWL-2928" "GIWL-2927" "GIWL-2926"

$`HDEN670307-WGS`
$`HDEN670307-WGS`$`TUSOP-2021`
epsilonKO betaKO gammaKO alphaKO deltaKO
"GSOP-3697" "GSOP-3695" "GSOP-3693" "GSOP-3692" "GSOP-3691"

$`HDEN670307-WGS`$`TUSOP-1721`
bKO2 bKO1 cKO aKO
"GSOP-3113" "GSOP-3112" "GSOP-3111" "GSOP-3110"

$MEXT419610
$MEXT419610$`TUI32-895`
deltaKO alphaKO
"GI32-1508" "GI32-1507"

$MEXT419610$`TUI32-894`
gammaKO betaKO epsilonKO
"GI32-1506" "GI32-1505" "GI32-1504"

$MEXT419610$`TUI32-1948`
aKO cKO

"GI32-3227" "GI32-3226"

$MEXT419610$`TUI32-1946`
bKO1
"GI32-3224"

$MEXT419610$`TUI32-1947`
bKO2
"GI32-3225"

$HCIN1172562
$HCIN1172562$`TULDZ-303`
bKO2 bKO1 deltaKO alphaKO gammaKO betaKO epsilonKO
"GLDZ-704" "GLDZ-703" "GLDZ-702" "GLDZ-701" "GLDZ-700" "GLDZ-699" "GLDZ-698"

$HCIN1172562$`TULDZ-170`
cKO
"GLDZ-342"

$HCIN1172562$`TULDZ-227`
aKO
"GLDZ-477"

$`PPUT1211579-WGS`
$`PPUT1211579-WGS`$`TUSSX-2960`
aKO cKO bKO deltaKO alphaKO gammaKO
"GSSX-5535" "GSSX-5534" "GSSX-5533" "GSSX-5532" "GSSX-5531" "GSSX-5530"
betaKO epsilonKO
"GSSX-5529" "GSSX-5528"

$HDUC233412
$HDUC233412$`TUH5F-4`
epsilonKO betaKO gammaKO alphaKO deltaKO bKO cKO aKO
"GH5F-11" "GH5F-10" "GH5F-9" "GH5F-8" "GH5F-7" "GH5F-6" "GH5F-5" "GH5F-4"

$HPYL907240
$HPYL907240$`TULEG-494`
bKO2 bKO1 deltaKO alphaKO gammaKO betaKO
"GLEG-1128" "GLEG-1127" "GLEG-1126" "GLEG-1125" "GLEG-1124" "GLEG-1123"
epsilonKO
"GLEG-1122"

$HPYL907240$`TULEG-538`
cKO
"GLEG-1214"

$HPYL907240$`TULEG-360`
aKO
"GLEG-827"

```
$`HPYL1234600-WGS`
$`HPYL1234600-WGS`$`TUSOF-502`
      bKO2       bKO1     deltaKO     alphaKO     gammaKO      betaKO
"GSOF-1155" "GSOF-1154" "GSOF-1153" "GSOF-1152" "GSOF-1151" "GSOF-1150"
  epsilonKO
"GSOF-1149"

$`HPYL1234600-WGS`$`TUSOF-548`
        cKO
"GSOF-1245"

$`HPYL1234600-WGS`$`TUSOF-365`
      aKO
"GSOF-837"


$`HPYL1321941-WGS`
$`HPYL1321941-WGS`$`TUSOK-537`
  epsilonKO      betaKO     gammaKO     alphaKO     deltaKO       bKO2
"GSOK-1319" "GSOK-1318" "GSOK-1317" "GSOK-1316" "GSOK-1315" "GSOK-1314"
      bKO1
"GSOK-1313"

$`HPYL1321941-WGS`$`TUSOK-492`
        cKO
"GSOK-1223"

$`HPYL1321941-WGS`$`TUSOK-124`
      aKO
"GSOK-289"


$HPYL1055530
$HPYL1055530$`TULER-472`
      bKO2       bKO1     deltaKO     alphaKO     gammaKO      betaKO
"GLER-1100" "GLER-1099" "GLER-1098" "GLER-1097" "GLER-1096" "GLER-1095"
  epsilonKO
"GLER-1094"

$HPYL1055530$`TULER-519`
        cKO
"GLER-1184"

$HPYL1055530$`TULER-358`
      aKO
"GLER-827"


$HPYL1055528
$HPYL1055528$`TULEL-488`
      bKO2       bKO1     deltaKO     alphaKO     gammaKO      betaKO
"GLEL-1072" "GLEL-1071" "GLEL-1070" "GLEL-1069" "GLEL-1068" "GLEL-1067"
  epsilonKO
```

"GLEL-1066"

$HPYL1055528$`TULEL-532`
cKO
"GLEL-1162"

$HPYL1055528$`TULEL-363`
aKO
"GLEL-792"

$`HPYL1234365-WGS`
$`HPYL1234365-WGS`$`TUSOD-503`
bKO2 bKO1 deltaKO alphaKO gammaKO betaKO
"GSOD-1158" "GSOD-1157" "GSOD-1156" "GSOD-1155" "GSOD-1154" "GSOD-1153"
epsilonKO
"GSOD-1152"

$`HPYL1234365-WGS`$`TUSOD-549`
cKO
"GSOD-1247"

$`HPYL1234365-WGS`$`TUSOD-365`
aKO
"GSOD-839"

$HPYL907239
$HPYL907239$`TULES-493`
bKO2 bKO1 deltaKO alphaKO gammaKO betaKO
"GLES-1081" "GLES-1080" "GLES-1079" "GLES-1078" "GLES-1077" "GLES-1076"
epsilonKO
"GLES-1075"

$HPYL907239$`TULES-540`
cKO
"GLES-1166"

$HPYL907239$`TULES-371`
aKO
"GLES-779"

$HPYL1055529
$HPYL1055529$`TULEM-481`
bKO2 bKO1 deltaKO alphaKO gammaKO betaKO
"GLEM-1142" "GLEM-1141" "GLEM-1140" "GLEM-1139" "GLEM-1138" "GLEM-1137"
epsilonKO
"GLEM-1136"

$HPYL1055529$`TULEM-523`
cKO
"GLEM-1231"

```
$HPYL1055529$`TULEM-344`
      aKO
"GLEM-806"


$AMIS512565
$AMIS512565$`TUL7J-3530`
   alphaKO    gammaKO     betaKO
"GL7J-7259" "GL7J-7258" "GL7J-7257"

$AMIS512565$`TUL7J-3531`
       cKO         bKO     deltaKO
"GL7J-7262" "GL7J-7261" "GL7J-7260"

$AMIS512565$`TUL7J-3528`
  epsilonKO
"GL7J-7255"

$AMIS512565$`TUL7J-3532`
       aKO
"GL7J-7263"


$PSP748280
$PSP748280$`TUHJ9-1278`
   gammaKO1    alphaKO1        bKO1        cKO1        aKO1  epsilonKO1
"GHJ9-2470" "GHJ9-2469" "GHJ9-2468" "GHJ9-2467" "GHJ9-2466" "GHJ9-2464"
    betaKO1
"GHJ9-2463"

$PSP748280$`TUHJ9-1963`
       aKO2        cKO2        bKO2     deltaKO    alphaKO2    gammaKO2
"GHJ9-3791" "GHJ9-3790" "GHJ9-3789" "GHJ9-3788" "GHJ9-3787" "GHJ9-3786"
    betaKO2  epsilonKO2
"GHJ9-3785" "GHJ9-3784"


$MFLA265072
$MFLA265072$`TUHWJ-1348`
        aKO          cKO          bKO      deltaKO      alphaKO      gammaKO
"GHWJ-2811" "GHWJ-2810" "GHWJ-2809" "GHWJ-2808" "GHWJ-2807" "GHWJ-2806"
     betaKO    epsilonKO
"GHWJ-2805" "GHWJ-2804"


$AMET293826
$AMET293826$`TUI5P-213`
 epsilonKO     betaKO    gammaKO    alphaKO    deltaKO        bKO        cKO
"GI5P-371" "GI5P-370" "GI5P-369" "GI5P-368" "GI5P-367" "GI5P-366" "GI5P-365"
       aKO
"GI5P-363"


$AMUC349741
```

```
$AMUC349741$`TUHZ7-292`
      bKO    deltaKO    alphaKO    gammaKO     betaKO  epsilonKO
"GHZ7-530" "GHZ7-529" "GHZ7-528" "GHZ7-527" "GHZ7-526" "GHZ7-525"

$AMUC349741$`TUHZ7-293`
      aKO        cKO
"GHZ7-532" "GHZ7-531"


$AMUL926570
$AMUL926570$`TUI8V-1024`
  epsilonKO      betaKO     gammaKO     alphaKO     deltaKO
"GI8V-1752" "GI8V-1751" "GI8V-1750" "GI8V-1749" "GI8V-1748"

$AMUL926570$`TUI8V-471`
      aKO        cKO       bKO2       bKO1
"GI8V-501" "GI8V-500" "GI8V-499" "GI8V-498"


$HPYL1127122
$HPYL1127122$`TULEU-551`
      bKO2        bKO1     deltaKO     alphaKO     gammaKO      betaKO
"GLEU-1203" "GLEU-1202" "GLEU-1201" "GLEU-1200" "GLEU-1199" "GLEU-1198"
  epsilonKO
"GLEU-1197"

$HPYL1127122$`TULEU-600`
       cKO
"GLEU-1295"

$HPYL1127122$`TULEU-250`
      aKO
"GLEU-559"


$HHAL349124
$HHAL349124$`TUI3I-1`
       aKO         cKO         bKO     deltaKO     alphaKO     gammaKO
"GI3I-2490" "GI3I-2489" "GI3I-2488" "GI3I-2487" "GI3I-2486" "GI3I-2485"
    betaKO   epsilonKO
"GI3I-2484" "GI3I-2483"


$HHAL866895
$HHAL866895$`TULDT-2046`
       aKO         cKO         bKO     deltaKO     alphaKO     gammaKO
"GLDT-3639" "GLDT-3638" "GLDT-3637" "GLDT-3636" "GLDT-3635" "GLDT-3634"
    betaKO   epsilonKO
"GLDT-3633" "GLDT-3632"


$`HHAL748449-WGS`
$`HHAL748449-WGS`$`TUSO1-1273`
       aKO         cKO         bKO     deltaKO     alphaKO     gammaKO
```

```
"GSO1-2462" "GSO1-2460" "GSO1-2459" "GSO1-2458" "GSO1-2457" "GSO1-2456"
     betaKO   epsilonKO
"GSO1-2455" "GSO1-2454"


$`HHEI1216962-WGS`
$`HHEI1216962-WGS`$`TUSO6-926`
  epsilonKO      betaKO     gammaKO     alphaKO     deltaKO        bKO1
"GSO6-1979" "GSO6-1978" "GSO6-1977" "GSO6-1976" "GSO6-1975" "GSO6-1974"
       bKO2
"GSO6-1973"

$`HHEI1216962-WGS`$`TUSO6-650`
        cKO
"GSO6-1333"

$`HHEI1216962-WGS`$`TUSO6-905`
        aKO
"GSO6-1920"


$HPYL1163740
$HPYL1163740$`TULEO-489`
       bKO2        bKO1     deltaKO     alphaKO     gammaKO      betaKO
"GLEO-1105" "GLEO-1104" "GLEO-1103" "GLEO-1102" "GLEO-1101" "GLEO-1100"
  epsilonKO
"GLEO-1099"

$HPYL1163740$`TULEO-533`
        cKO
"GLEO-1192"

$HPYL1163740$`TULEO-235`
       aKO
"GLEO-552"


$`RBAL243090-WGS`
$`RBAL243090-WGS`$`TUSTO-2848`
 epsilonKO1     betaKO1    gammaKO1    alphaKO1     deltaKO        bKO1
"GSTO-5699" "GSTO-5698" "GSTO-5697" "GSTO-5696" "GSTO-5695" "GSTO-5694"
       cKO1        aKO1
"GSTO-5693" "GSTO-5691"

$`RBAL243090-WGS`$`TUSTO-1376`
   gammaKO2    alphaKO2        bKO2        cKO2        aKO2  epsilonKO2
"GSTO-2759" "GSTO-2758" "GSTO-2757" "GSTO-2756" "GSTO-2755" "GSTO-2752"
    betaKO2
"GSTO-2751"


$HPYL1163741
$HPYL1163741$`TULEP-463`
       bKO2        bKO1     deltaKO     alphaKO     gammaKO      betaKO
```

"GLEP-1101" "GLEP-1100" "GLEP-1099" "GLEP-1098" "GLEP-1097" "GLEP-1096"
epsilonKO
"GLEP-1095"

$HPYL1163741$`TULEP-504`
cKO
"GLEP-1188"

$HPYL1163741$`TULEP-350`
aKO
"GLEP-831"

$MFLO265311
$MFLO265311$`TUHIB-54`
epsilonKO betaKO gammaKO alphaKO deltaKO bKO cKO
"GHIB-137" "GHIB-136" "GHIB-135" "GHIB-134" "GHIB-133" "GHIB-132" "GHIB-131"
aKO
"GHIB-130"

$AARA574087
$AARA574087$`TUHPK-1098`
aKO cKO bKO deltaKO alphaKO gammaKO
"GHPK-2306" "GHPK-2304" "GHPK-2303" "GHPK-2302" "GHPK-2301" "GHPK-2300"
betaKO epsilonKO
"GHPK-2299" "GHPK-2298"

$AASI452471
$AASI452471$`TUKEN-48`
gammaKO alphaKO deltaKO bKO cKO aKO
"GKEN-70" "GKEN-69" "GKEN-68" "GKEN-67" "GKEN-66" "GKEN-65"

$AASI452471$`TUKEN-704`
betaKO
"GKEN-1016"

$AASI452471$`TUKEN-781`
epsilonKO
"GKEN-1123"

$AACT668336
$AACT668336$`TUJBF-195`
epsilonKO betaKO gammaKO alphaKO deltaKO bKO cKO
"GJBF-324" "GJBF-323" "GJBF-322" "GJBF-321" "GJBF-320" "GJBF-319" "GJBF-318"
aKO
"GJBF-317"

$AAUR290340
$AAUR290340$`TUI59-1679`
aKO cKO bKO alphaKO gammaKO betaKO

```
"GI59-2599" "GI59-2598" "GI59-2597" "GI59-2595" "GI59-2594" "GI59-2593"

$AAUR290340$noTU
  deltaKO epsilonKO
       NA        NA



$HPYL1163739
$HPYL1163739$`TULEQ-447`
       bKO2        bKO1     deltaKO     alphaKO     gammaKO      betaKO
"GLEQ-1070" "GLEQ-1069" "GLEQ-1068" "GLEQ-1067" "GLEQ-1066" "GLEQ-1065"
  epsilonKO
"GLEQ-1064"

$HPYL1163739$`TULEQ-499`
        cKO
"GLEQ-1180"

$HPYL1163739$`TULEQ-329`
       aKO
"GLEQ-785"



$HHYD760192
$HHYD760192$`TUI21-2313`
    gammaKO     alphaKO     deltaKO         bKO         cKO         aKO
"GI21-3291" "GI21-3290" "GI21-3289" "GI21-3288" "GI21-3287" "GI21-3286"

$HHYD760192$`TUI21-2216`
     betaKO
"GI21-3141"

$HHYD760192$`TUI21-2215`
  epsilonKO
"GI21-3140"



$HINF262727
$HINF262727$`TULDQ-58`
 epsilonKO     betaKO    gammaKO    alphaKO    deltaKO        bKO        cKO
"GLDQ-102" "GLDQ-101" "GLDQ-100"  "GLDQ-99"  "GLDQ-98"  "GLDQ-97"  "GLDQ-96"
       aKO
 "GLDQ-95"



$HINF866630
$HINF866630$`TUJN7-927`
  epsilonKO      betaKO     gammaKO     alphaKO     deltaKO         bKO
"GJN7-1695" "GJN7-1694" "GJN7-1693" "GJN7-1692" "GJN7-1691" "GJN7-1690"
        cKO
"GJN7-1689"

$HINF866630$`TUJN7-926`
        aKO
```

```
"GJN7-1688"


$RBEL391896
$RBEL391896$`TUH75-834`
   deltaKO     alphaKO     gammaKO      betaKO
"GH75-1413" "GH75-1412" "GH75-1411" "GH75-1410"

$RBEL391896$`TUH75-833`
  epsilonKO
"GH75-1409"

$RBEL391896$`TUH75-49`
      aKO       cKO      bKO2      bKO1
"GH75-78" "GH75-77" "GH75-76" "GH75-75"


$HINF935897
$HINF935897$`TUJ9O-382`
 epsilonKO     betaKO    gammaKO    alphaKO    deltaKO        bKO        cKO
"GJ9O-690" "GJ9O-689" "GJ9O-688" "GJ9O-687" "GJ9O-686" "GJ9O-685" "GJ9O-684"

$HINF935897$`TUJ9O-381`
       aKO
"GJ9O-683"


$`HINF71421-WGS`
$`HINF71421-WGS`$`TUSNX-259`
       aKO        cKO        bKO    deltaKO    alphaKO    gammaKO     betaKO
"GSNX-498" "GSNX-497" "GSNX-496" "GSNX-495" "GSNX-494" "GSNX-493" "GSNX-492"
 epsilonKO
"GSNX-491"


$MFER943945
$MFER943945$`TUH8M-136`
 epsilonKO     betaKO    gammaKO    alphaKO    deltaKO        bKO        cKO
"GH8M-283" "GH8M-282" "GH8M-281" "GH8M-280" "GH8M-279" "GH8M-278" "GH8M-277"
       aKO
"GH8M-276"


$HINF374930
$HINF374930$`TUJDD-57`
 epsilonKO     betaKO    gammaKO    alphaKO    deltaKO        bKO        cKO
"GJDD-110" "GJDD-109" "GJDD-108" "GJDD-107" "GJDD-106" "GJDD-105" "GJDD-104"
       aKO
"GJDD-103"


$HINF374931
$HINF374931$`TUJA4-529`
       aKO        cKO        bKO    deltaKO    alphaKO    gammaKO     betaKO
```

"GJA4-978" "GJA4-977" "GJA4-976" "GJA4-975" "GJA4-974" "GJA4-973" "GJA4-972"
epsilonKO
"GJA4-971"

$HINF281310
$HINF281310$`TUJ89-293`
aKO cKO bKO deltaKO alphaKO gammaKO betaKO
"GJ89-578" "GJ89-577" "GJ89-576" "GJ89-575" "GJ89-574" "GJ89-573" "GJ89-572"
epsilonKO
"GJ89-571"

$HINF862964
$HINF862964$`TUHI0-341`
bKO deltaKO alphaKO gammaKO betaKO epsilonKO
"GHI0-638" "GHI0-637" "GHI0-636" "GHI0-635" "GHI0-634" "GHI0-633"

$HINF862964$`TUHI0-342`
cKO
"GHI0-639"

$HINF862964$`TUHI0-343`
aKO
"GHI0-640"

$HINF262728
$HINF262728$`TULDR-49`
epsilonKO betaKO gammaKO alphaKO deltaKO bKO cKO aKO
"GLDR-96" "GLDR-95" "GLDR-94" "GLDR-93" "GLDR-92" "GLDR-91" "GLDR-90" "GLDR-89"

$HMOD498761
$HMOD498761$`TUI46-486`
aKO cKO bKO deltaKO alphaKO gammaKO
"GI46-871" "GI46-870" "GI46-869" "GI46-868" "GI46-867" "GI46-866"

$HMOD498761$`TUI46-485`
betaKO epsilonKO
"GI46-865" "GI46-864"

$HMAR760142
$HMAR760142$`TUHVE-313`
bKO2 bKO1 deltaKO alphaKO gammaKO betaKO
"GHVE-1031" "GHVE-1030" "GHVE-1029" "GHVE-1028" "GHVE-1027" "GHVE-1026"
epsilonKO
"GHVE-1025"

$HMAR760142$`TUHVE-276`
cKO aKO
"GHVE-879" "GHVE-878"

$RCEN414684
$RCEN414684$`TUHCM-192`
  gammaKO1   alphaKO1   bKO1   cKO1   aKO1 epsilonKO1   betaKO1
"GHCM-357" "GHCM-356" "GHCM-355" "GHCM-354" "GHCM-353" "GHCM-350" "GHCM-349"

$RCEN414684$`TUHCM-1231`
    deltaKO   alphaKO2   gammaKO2   betaKO2 epsilonKO2
"GHCM-2222" "GHCM-2221" "GHCM-2220" "GHCM-2219" "GHCM-2218"

$RCEN414684$`TUHCM-2011`
       bKO3   bKO2   cKO2   aKO2
"GHCM-3489" "GHCM-3488" "GHCM-3487" "GHCM-3486"

$HNEA555778
$HNEA555778$`TUIVV-1196`
        aKO   cKO   bKO   deltaKO   alphaKO   gammaKO
"GIVV-2383" "GIVV-2382" "GIVV-2381" "GIVV-2380" "GIVV-2379" "GIVV-2378"
     betaKO   epsilonKO
"GIVV-2377" "GIVV-2376"

$HNEP228405
$HNEP228405$`TUI69-996`
    deltaKO   alphaKO   gammaKO   betaKO   epsilonKO
"GI69-1895" "GI69-1894" "GI69-1893" "GI69-1892" "GI69-1891"

$HNEP228405$`TUI69-1010`
       bKO2   bKO1   cKO   aKO
"GI69-1923" "GI69-1922" "GI69-1921" "GI69-1920"

$HOCH502025
$HOCH502025$`TUI43-3468`
       bKO1   deltaKO   alphaKO   gammaKO   betaKO   epsilonKO
"GI43-6111" "GI43-6110" "GI43-6109" "GI43-6108" "GI43-6107" "GI43-6106"

$HOCH502025$`TUI43-2499`
        aKO   cKO
"GI43-4506" "GI43-4505"

$HOCH502025$`TUI43-3469`
       bKO2
"GI43-6112"

$MFER637387
$MFER637387$`TUI6C-109`
 epsilonKO   betaKO   gammaKO   alphaKO   deltaKO   bKO   cKO
"GI6C-218" "GI6C-217" "GI6C-216" "GI6C-215" "GI6C-214" "GI6C-213" "GI6C-212"
       aKO
"GI6C-211"

```
$HORE373903
$HORE373903$`TUHB1-928`
       bKO     deltaKO     alphaKO     gammaKO      betaKO   epsilonKO
"GHB1-1849" "GHB1-1848" "GHB1-1847" "GHB1-1846" "GHB1-1845" "GHB1-1844"

$HORE373903$`TUHB1-929`
       aKO         cKO
"GHB1-1852" "GHB1-1851"


$HPYL357544
$HPYL357544$`TUH1F-484`
      bKO2        bKO1     deltaKO     alphaKO     gammaKO      betaKO
"GH1F-1101" "GH1F-1100" "GH1F-1099" "GH1F-1098" "GH1F-1097" "GH1F-1096"
  epsilonKO
"GH1F-1095"

$HPYL357544$`TUH1F-526`
       cKO
"GH1F-1188"

$HPYL357544$`TUH1F-368`
      aKO
"GH1F-825"


$`HPAR1322346-WGS`
$`HPAR1322346-WGS`$`TUSNY-1166`
  epsilonKO      betaKO     gammaKO     alphaKO     deltaKO         bKO
"GSNY-2093" "GSNY-2092" "GSNY-2091" "GSNY-2090" "GSNY-2089" "GSNY-2088"
       cKO         aKO
"GSNY-2087" "GSNY-2086"


$HPYL592205
$HPYL592205$`TUJAG-470`
      bKO2        bKO1     deltaKO     alphaKO     gammaKO      betaKO
"GJAG-1092" "GJAG-1091" "GJAG-1090" "GJAG-1089" "GJAG-1088" "GJAG-1087"
  epsilonKO
"GJAG-1086"

$HPYL592205$`TUJAG-509`
       cKO
"GJAG-1174"

$HPYL592205$`TUJAG-215`
      aKO
"GJAG-521"


$HPYL765963
$HPYL765963$`TUH9T-486`
      bKO2        bKO1     deltaKO     alphaKO     gammaKO      betaKO
```

"GH9T-1082" "GH9T-1081" "GH9T-1080" "GH9T-1079" "GH9T-1078" "GH9T-1077"
epsilonKO
"GH9T-1076"

$HPYL765963$`TUH9T-528`
cKO
"GH9T-1168"

$HPYL765963$`TUH9T-373`
aKO
"GH9T-813"

$HPYL1055527
$HPYL1055527$`TULEB-98`
epsilonKO betaKO gammaKO alphaKO deltaKO bKO2 bKO1
"GLEB-195" "GLEB-194" "GLEB-193" "GLEB-192" "GLEB-191" "GLEB-190" "GLEB-189"

$HPYL1055527$`TULEB-559`
cKO
"GLEB-1228"

$HPYL1055527$`TULEB-227`
aKO
"GLEB-468"

$LMON1030009
$LMON1030009$`TULFW-58`
epsilonKO1 betaKO1 gammaKO1 alphaKO1 deltaKO1 cKO1
"GLFW-129" "GLFW-128" "GLFW-127" "GLFW-126" "GLFW-125" "GLFW-124"

$LMON1030009$`TULFW-1354`
cKO2 bKO deltaKO2 alphaKO2 gammaKO2
"GLFW-2652" "GLFW-2651" "GLFW-2650" "GLFW-2649" "GLFW-2648"

$LMON1030009$`TULFW-1353`
betaKO2 epsilonKO2
"GLFW-2647" "GLFW-2646"

$LMON1030009$`TULFW-1355`
aKO
"GLFW-2653"

$HPYL563041
$HPYL563041$`TUC38-527`
bKO2 bKO1 deltaKO alphaKO gammaKO betaKO
"GC38-1112" "GC38-1111" "GC38-1110" "GC38-1109" "GC38-1108" "GC38-1107"
epsilonKO
"GC38-1106"

$HPYL563041$`TUC38-573`
cKO

"GC38-1197"

$HPYL563041$`TUC38-387`
aKO
"GC38-800"

$HPYL907237
$HPYL907237$`TULEJ-481`
bKO2 bKO1 deltaKO alphaKO gammaKO betaKO
"GLEJ-1086" "GLEJ-1085" "GLEJ-1084" "GLEJ-1083" "GLEJ-1082" "GLEJ-1081"
epsilonKO
"GLEJ-1080"

$HPYL907237$`TULEJ-526`
cKO
"GLEJ-1174"

$HPYL907237$`TULEJ-356`
aKO
"GLEJ-795"

$HPYL869727
$HPYL869727$`TULE7-491`
bKO2 bKO1 deltaKO alphaKO gammaKO betaKO
"GLE7-1160" "GLE7-1159" "GLE7-1158" "GLE7-1157" "GLE7-1156" "GLE7-1155"
epsilonKO
"GLE7-1154"

$HPYL869727$`TULE7-535`
cKO
"GLE7-1249"

$HPYL869727$`TULE7-366`
aKO
"GLE7-857"

$HPYL85963
$HPYL85963$`TUJB9-480`
bKO2 bKO1 deltaKO alphaKO gammaKO betaKO
"GJB9-1092" "GJB9-1091" "GJB9-1090" "GJB9-1089" "GJB9-1088" "GJB9-1087"
epsilonKO
"GJB9-1086"

$HPYL85963$`TUJB9-521`
cKO
"GJB9-1171"

$HPYL85963$`TUJB9-340`
aKO
"GJB9-779"

```
$MFUL483219
$MFUL483219$`TUJEO-1296`
    alphaKO
"GJEO-2242"

$MFUL483219$`TUJEO-1357`
  epsilonKO      betaKO     gammaKO
"GJEO-2364" "GJEO-2362" "GJEO-2361"

$MFUL483219$`TUJEO-1298`
    deltaKO
"GJEO-2244"

$MFUL483219$`TUJEO-785`
        aKO         cKO         bKO
"GJEO-1326" "GJEO-1325" "GJEO-1324"


$HPRA572479
$HPRA572479$`TULDS-996`
        aKO         cKO         bKO     deltaKO     alphaKO     gammaKO
"GLDS-2035" "GLDS-2032" "GLDS-2031" "GLDS-2030" "GLDS-2029" "GLDS-2028"
     betaKO   epsilonKO
"GLDS-2027" "GLDS-2026"


$HPYL765962
$HPYL765962$`TUH29-490`
       bKO2        bKO1     deltaKO     alphaKO     gammaKO      betaKO
"GH29-1110" "GH29-1109" "GH29-1108" "GH29-1107" "GH29-1106" "GH29-1105"
  epsilonKO
"GH29-1104"

$HPYL765962$`TUH29-534`
        cKO
"GH29-1201"

$HPYL765962$`TUH29-368`
       aKO
"GH29-832"


$HPYL907238
$HPYL907238$`TULEI-472`
       bKO2        bKO1     deltaKO     alphaKO     gammaKO      betaKO
"GLEI-1098" "GLEI-1097" "GLEI-1096" "GLEI-1095" "GLEI-1094" "GLEI-1093"
  epsilonKO
"GLEI-1092"

$HPYL907238$`TULEI-538`
        cKO
"GLEI-1248"
```

```
$HPYL907238$`TULEI-210`
       aKO
"GLEI-494"


$ASP46234
$ASP46234$`TUL84-2996`
        aKO         cKO        bKO2        bKO1     deltaKO     alphaKO
"GL84-4163" "GL84-4162" "GL84-4161" "GL84-4160" "GL84-4159" "GL84-4158"
    gammaKO
"GL84-4157"

$ASP46234$`TUL84-2112`
     betaKO   epsilonKO
"GL84-2895" "GL84-2894"


$ASP447217
$ASP447217$`TUHB0-2346`
    deltaKO     alphaKO     gammaKO      betaKO   epsilonKO
"GHB0-4546" "GHB0-4545" "GHB0-4544" "GHB0-4543" "GHB0-4542"

$ASP447217$`TUHB0-2344`
        aKO         cKO         bKO
"GHB0-4535" "GHB0-4534" "GHB0-4533"


$RLEG216596
$RLEG216596$`TUKE5-3911`
    deltaKO     alphaKO     gammaKO      betaKO   epsilonKO
"GKE5-4486" "GKE5-4485" "GKE5-4484" "GKE5-4483" "GKE5-4482"

$RLEG216596$`TUKE5-1864`
      bKO2       bKO1        cKO        aKO
"GKE5-957" "GKE5-956" "GKE5-955" "GKE5-954"


$ANIT572480
$ANIT572480$`TUJ62-1003`
       bKO2        bKO1     deltaKO     alphaKO     gammaKO      betaKO
"GJ62-2391" "GJ62-2390" "GJ62-2389" "GJ62-2388" "GJ62-2387" "GJ62-2386"
  epsilonKO
"GJ62-2385"

$ANIT572480$`TUJ62-352`
       cKO
"GJ62-869"

$ANIT572480$`TUJ62-221`
       aKO
"GJ62-529"


$HPYL585535
```

$HPYL585535$`TULE3-560`
bKO2 bKO1 deltaKO alphaKO gammaKO betaKO
"GLE3-1308" "GLE3-1307" "GLE3-1306" "GLE3-1305" "GLE3-1304" "GLE3-1303"
epsilonKO
"GLE3-1302"

$HPYL585535$`TULE3-601`
cKO
"GLE3-1393"

$HPYL585535$`TULE3-309`
aKO
"GLE3-753"

$HPYL570508
$HPYL570508$`TUJ8D-497`
bKO2 bKO1 deltaKO alphaKO gammaKO betaKO
"GJ8D-1134" "GJ8D-1133" "GJ8D-1132" "GJ8D-1131" "GJ8D-1130" "GJ8D-1129"
epsilonKO
"GJ8D-1128"

$HPYL570508$`TUJ8D-537`
cKO
"GJ8D-1217"

$HPYL570508$`TUJ8D-375`
aKO
"GJ8D-850"

$HPYL985081
$HPYL985081$`TULE0-494`
bKO2 bKO1 deltaKO alphaKO gammaKO betaKO
"GLE0-1161" "GLE0-1160" "GLE0-1159" "GLE0-1158" "GLE0-1157" "GLE0-1156"
epsilonKO
"GLE0-1155"

$HPYL985081$`TULE0-540`
cKO
"GLE0-1251"

$HPYL985081$`TULE0-368`
aKO
"GLE0-855"

$HPAR862965
$HPAR862965$`TUH07-288`
bKO deltaKO alphaKO gammaKO betaKO epsilonKO
"GH07-588" "GH07-587" "GH07-586" "GH07-585" "GH07-584" "GH07-583"

$HPAR862965$`TUH07-289`
cKO

"GH07-589"

$HPAR862965$`TUH07-290`
aKO
"GH07-590"

$MGAL710127
$MGAL710127$`TUC09-337`
betaKO1 alphaKO1
"GC09-740" "GC09-739"

$MGAL710127$`TUC09-158`
epsilonKO betaKO2 gammaKO alphaKO2 deltaKO bKO cKO
"GC09-359" "GC09-358" "GC09-357" "GC09-356" "GC09-355" "GC09-354" "GC09-353"
aKO
"GC09-352"

$HPYL512562
$HPYL512562$`TUHHZ-488`
bKO2 bKO1 deltaKO alphaKO gammaKO betaKO
"GHHZ-1163" "GHHZ-1162" "GHHZ-1161" "GHHZ-1160" "GHHZ-1159" "GHHZ-1158"
epsilonKO
"GHHZ-1157"

$HPYL512562$`TUHHZ-530`
cKO
"GHHZ-1254"

$HPYL512562$`TUHHZ-221`
aKO
"GHHZ-534"

$HPYL794851
$HPYL794851$`TULEN-473`
bKO2 bKO1 deltaKO alphaKO gammaKO betaKO
"GLEN-1081" "GLEN-1080" "GLEN-1079" "GLEN-1078" "GLEN-1077" "GLEN-1076"
epsilonKO
"GLEN-1075"

$HPYL794851$`TULEN-518`
cKO
"GLEN-1161"

$HPYL794851$`TULEN-219`
aKO
"GLEN-525"

$HPYL765964
$HPYL765964$`TULEA-481`
bKO2 bKO1 deltaKO alphaKO gammaKO betaKO

"GLEA-1137" "GLEA-1136" "GLEA-1135" "GLEA-1134" "GLEA-1133" "GLEA-1132"
epsilonKO
"GLEA-1131"

$HPYL765964$`TULEA-525`
cKO
"GLEA-1226"

$HPYL765964$`TULEA-360`
aKO
"GLEA-857"

$HPYL637913
$HPYL637913$`TULEV-535`
bKO2 bKO1 deltaKO alphaKO gammaKO betaKO
"GLEV-1151" "GLEV-1150" "GLEV-1149" "GLEV-1148" "GLEV-1147" "GLEV-1146"
epsilonKO
"GLEV-1145"

$HPYL637913$`TULEV-580`
cKO
"GLEV-1240"

$HPYL637913$`TULEV-398`
aKO
"GLEV-842"

$RPIC428406
$RPIC428406$`TUH9Y-2203`
aKO cKO bKO deltaKO alphaKO gammaKO
"GH9Y-3253" "GH9Y-3252" "GH9Y-3251" "GH9Y-3250" "GH9Y-3249" "GH9Y-3248"
betaKO epsilonKO1
"GH9Y-3247" "GH9Y-3246"

$RPIC428406$`TUH9Y-2956`
epsilonKO2
"GH9Y-4666"

$HPYL985080
$HPYL985080$`TULE1-498`
bKO2 bKO1 deltaKO alphaKO gammaKO betaKO
"GLE1-1174" "GLE1-1173" "GLE1-1172" "GLE1-1171" "GLE1-1170" "GLE1-1169"
epsilonKO
"GLE1-1168"

$HPYL985080$`TULE1-544`
cKO
"GLE1-1264"

$HPYL985080$`TULE1-372`
aKO

"GLE1-860"

$HPYL1055531
$HPYL1055531$`TULE8-477`
bKO2 bKO1 deltaKO alphaKO gammaKO betaKO
"GLE8-1070" "GLE8-1069" "GLE8-1068" "GLE8-1067" "GLE8-1066" "GLE8-1065"
epsilonKO
"GLE8-1064"

$HPYL1055531$`TULE8-519`
cKO
"GLE8-1150"

$HPYL1055531$`TULE8-360`
aKO
"GLE8-788"

$`HPYL1321939-WGS`
$`HPYL1321939-WGS`$`TUSOI-444`
bKO1 bKO2 deltaKO alphaKO gammaKO betaKO
"GSOI-1054" "GSOI-1053" "GSOI-1052" "GSOI-1051" "GSOI-1050" "GSOI-1049"
epsilonKO
"GSOI-1048"

$`HPYL1321939-WGS`$`TUSOI-490`
cKO
"GSOI-1143"

$`HPYL1321939-WGS`$`TUSOI-285`
aKO
"GSOI-649"

$HPYL1055532
$HPYL1055532$`TULE9-446`
bKO2 bKO1 deltaKO alphaKO gammaKO betaKO
"GLE9-1059" "GLE9-1058" "GLE9-1057" "GLE9-1056" "GLE9-1055" "GLE9-1054"
epsilonKO
"GLE9-1053"

$HPYL1055532$`TULE9-108`
cKO
"GLE9-260"

$HPYL1055532$`TULE9-249`
aKO
"GLE9-583"

$`HPYL1321938-WGS`
$`HPYL1321938-WGS`$`TUSOL-306`
bKO1 bKO2 deltaKO alphaKO gammaKO betaKO epsilonKO

```
"GSOL-747" "GSOL-746" "GSOL-745" "GSOL-744" "GSOL-743" "GSOL-742" "GSOL-741"

$`HPYL1321938-WGS`$`TUSOL-350`
       cKO
"GSOL-837"

$`HPYL1321938-WGS`$`TUSOL-54`
       aKO
"GSOL-144"


$`HPYL1311573-WGS`
$`HPYL1311573-WGS`$`TUSOH-505`
       bKO1        bKO2      deltaKO      alphaKO      gammaKO      betaKO
"GSOH-1207" "GSOH-1206" "GSOH-1205" "GSOH-1204" "GSOH-1203" "GSOH-1202"
  epsilonKO
"GSOH-1201"

$`HPYL1311573-WGS`$`TUSOH-550`
        cKO
"GSOH-1297"

$`HPYL1311573-WGS`$`TUSOH-253`
       aKO
"GSOH-607"


$MGEN663918
$MGEN663918$`TULHC-154`
       aKO        cKO        bKO    deltaKO    alphaKO     betaKO  epsilonKO
"GLHC-476" "GLHC-475" "GLHC-474" "GLHC-473" "GLHC-472" "GLHC-471" "GLHC-470"

$MGEN663918$noTU
gammaKO
     NA


$`HPYL1352356-WGS`
$`HPYL1352356-WGS`$`TUSOG-537`
       bKO2        bKO1      deltaKO      alphaKO      gammaKO      betaKO
"GSOG-1214" "GSOG-1213" "GSOG-1212" "GSOG-1211" "GSOG-1210" "GSOG-1209"
  epsilonKO
"GSOG-1208"

$`HPYL1352356-WGS`$`TUSOG-587`
        cKO
"GSOG-1304"

$`HPYL1352356-WGS`$`TUSOG-391`
       aKO
"GSOG-880"


$`HPYL1321940-WGS`
```

```
$`HPYL1321940-WGS`$`TUSOJ-524`
       bKO1        bKO2     deltaKO     alphaKO     gammaKO      betaKO
"GSOJ-1253" "GSOJ-1252" "GSOJ-1251" "GSOJ-1250" "GSOJ-1249" "GSOJ-1248"
  epsilonKO
"GSOJ-1247"

$`HPYL1321940-WGS`$`TUSOJ-567`
        cKO
"GSOJ-1342"

$`HPYL1321940-WGS`$`TUSOJ-264`
       aKO
"GSOJ-675"


$HSER757424
$HSER757424$`TUCTT-2512`
        aKO         cKO         bKO     deltaKO     alphaKO     gammaKO
"GCTT-4368" "GCTT-4367" "GCTT-4366" "GCTT-4365" "GCTT-4364" "GCTT-4363"
     betaKO   epsilonKO
"GCTT-4362" "GCTT-4361"


$RPIC402626
$RPIC402626$`TUH94-2536`
        aKO         cKO         bKO     deltaKO     alphaKO     gammaKO
"GH94-3575" "GH94-3574" "GH94-3573" "GH94-3572" "GH94-3571" "GH94-3570"
     betaKO  epsilonKO1
"GH94-3569" "GH94-3568"

$RPIC402626$`TUH94-434`
 epsilonKO2
"GH94-4527"


$HSOM228400
$HSOM228400$`TUHWT-1001`
        aKO         cKO         bKO     deltaKO     alphaKO     gammaKO
"GHWT-1896" "GHWT-1895" "GHWT-1894" "GHWT-1893" "GHWT-1892" "GHWT-1891"
     betaKO   epsilonKO
"GHWT-1890" "GHWT-1889"


$HSOM205914
$HSOM205914$`TUJ7V-950`
        aKO         cKO         bKO     deltaKO     alphaKO     gammaKO
"GJ7V-1783" "GJ7V-1782" "GJ7V-1781" "GJ7V-1780" "GJ7V-1779" "GJ7V-1778"
     betaKO   epsilonKO
"GJ7V-1777" "GJ7V-1776"


$HTHE608538
$HTHE608538$`TUC72-2842`
    alphaKO
```

```
"GC72-1366"

$HTHE608538$`TUC72-2814`
     betaKO    gammaKO1
"GC72-1369" "GC72-1373"

$HTHE608538$`TUC72-3062`
   gammaKO2
"GC72-1374"

$HTHE608538$`TUC72-3070`
       bKO2        bKO1     deltaKO
"GC72-1371" "GC72-1372" "GC72-1375"

$HTHE608538$`TUC72-3104`
  epsilonKO
"GC72-1368"

$HTHE608538$`TUC72-3194`
        cKO         aKO
"GC72-1370" "GC72-1367"


$HSP380749
$HSP380749$`TUH30-323`
       bKO2        bKO1     deltaKO     alphaKO
"GH30-1052" "GH30-1051" "GH30-1050" "GH30-1049"

$HSP380749$`TUH30-325`
     betaKO
"GH30-1057"

$HSP380749$`TUH30-514`
    gammaKO
"GH30-1610"

$HSP380749$`TUH30-348`
  epsilonKO
"GH30-1124"

$HSP380749$`TUH30-276`
       cKO        aKO
"GH30-913" "GH30-911"


$IALB945713
$IALB945713$`TULEW-374`
   gammaKO    alphaKO    deltaKO        bKO        cKO        aKO
"GLEW-779" "GLEW-778" "GLEW-777" "GLEW-776" "GLEW-775" "GLEW-774"

$IALB945713$`TULEW-785`
     betaKO   epsilonKO
"GLEW-1667" "GLEW-1666"
```

$ICAL710696
$ICAL710696$`TUH9U-1259`
aKO cKO bKO deltaKO alphaKO gammaKO
"GH9U-2482" "GH9U-2481" "GH9U-2480" "GH9U-2479" "GH9U-2478" "GH9U-2477"
betaKO
"GH9U-2476"

$ICAL710696$`TUH9U-1257`
epsilonKO
"GH9U-2473"

$`ILOI1321370-WGS`
$`ILOI1321370-WGS`$`TUSOQ-1190`
aKO cKO bKO deltaKO alphaKO gammaKO
"GSOQ-2702" "GSOQ-2701" "GSOQ-2700" "GSOQ-2699" "GSOQ-2698" "GSOQ-2697"
betaKO epsilonKO
"GSOQ-2696" "GSOQ-2695"

$AACI521098
$AACI521098$`TUCIO-1509`
aKO cKO bKO deltaKO alphaKO gammaKO
"GCIO-2846" "GCIO-2845" "GCIO-2844" "GCIO-2843" "GCIO-2842" "GCIO-2841"
betaKO epsilonKO
"GCIO-2840" "GCIO-2839"

$APAS634459
$APAS634459$`TUL7A-296`
epsilonKO betaKO gammaKO alphaKO deltaKO
"GL7A-121" "GL7A-120" "GL7A-119" "GL7A-118" "GL7A-117"

$APAS634459$`TUL7A-1514`
aKO cKO
"GL7A-2465" "GL7A-2464"

$APAS634459$`TUL7A-1513`
bKO2 bKO1
"GL7A-2463" "GL7A-2462"

$MGEN243273
$MGEN243273$`TUH2R-127`
aKO cKO bKO deltaKO alphaKO gammaKO betaKO
"GH2R-462" "GH2R-461" "GH2R-460" "GH2R-459" "GH2R-458" "GH2R-457" "GH2R-456"
epsilonKO
"GH2R-455"

$RSOL859657
$RSOL859657$`TUJJ9-1051`
epsilonKO1 betaKO gammaKO alphaKO deltaKO bKO cKO

"GJJ9-124" "GJJ9-123" "GJJ9-122" "GJJ9-121" "GJJ9-120" "GJJ9-119" "GJJ9-118"
aKO
"GJJ9-117"

$RSOL859657$`TUJJ9-410`
epsilonKO2
"GJJ9-4151"

$ILOI283942
$ILOI283942$`TUI0U-1163`
aKO cKO bKO deltaKO alphaKO gammaKO
"GI0U-2641" "GI0U-2640" "GI0U-2639" "GI0U-2638" "GI0U-2637" "GI0U-2636"
betaKO epsilonKO
"GI0U-2635" "GI0U-2634"

$IPAL575540
$IPAL575540$`TUI5T-424`
aKO cKO bKO deltaKO alphaKO gammaKO betaKO
"GI5T-565" "GI5T-564" "GI5T-563" "GI5T-562" "GI5T-561" "GI5T-560" "GI5T-559"
epsilonKO
"GI5T-558"

$IPOL572544
$IPOL572544$`TUJ9I-686`
epsilonKO betaKO gammaKO alphaKO deltaKO bKO cKO
"GJ9I-225" "GJ9I-224" "GJ9I-223" "GJ9I-222" "GJ9I-221" "GJ9I-220" "GJ9I-219"
aKO
"GJ9I-218"

$IVAR743718
$IVAR743718$`TUI39-1084`
aKO cKO bKO deltaKO alphaKO gammaKO
"GI39-2350" "GI39-2349" "GI39-2348" "GI39-2347" "GI39-2346" "GI39-2345"
betaKO epsilonKO
"GI39-2344" "GI39-2343"

$JSP290400
$JSP290400$`TUI1R-569`
epsilonKO betaKO gammaKO alphaKO deltaKO
"GI1R-1058" "GI1R-1057" "GI1R-1056" "GI1R-1055" "GI1R-1054"

$JSP290400$`TUI1R-428`
cKO
"GI1R-775"

$JSP290400$`TUI1R-427`
aKO
"GI1R-774"

$JSP290400$`TUI1R-429`
bKO2 bKO1
"GI1R-777" "GI1R-776"

$JDEN471856
$JDEN471856$`TUH77-916`
aKO cKO bKO deltaKO alphaKO gammaKO
"GH77-1837" "GH77-1836" "GH77-1835" "GH77-1834" "GH77-1833" "GH77-1832"
betaKO
"GH77-1831"

$JDEN471856$`TUH77-915`
epsilonKO
"GH77-1830"

$`CKIN1208923-WGS`
$`CKIN1208923-WGS`$`TUSI8-117`
epsilonKO betaKO gammaKO alphaKO deltaKO bKO cKO
"GSI8-229" "GSI8-228" "GSI8-227" "GSI8-226" "GSI8-225" "GSI8-224" "GSI8-223"
aKO
"GSI8-222"

$`CKIN1208922-WGS`
$`CKIN1208922-WGS`$`TUSIQ-21`
epsilonKO betaKO gammaKO alphaKO deltaKO bKO cKO aKO
"GSIQ-58" "GSIQ-57" "GSIQ-56" "GSIQ-55" "GSIQ-54" "GSIQ-53" "GSIQ-52" "GSIQ-51"

$`CKIN1267577-WGS`
$`CKIN1267577-WGS`$`TUSI9-341`
epsilonKO betaKO gammaKO alphaKO deltaKO bKO cKO
"GSI9-664" "GSI9-663" "GSI9-662" "GSI9-661" "GSI9-660" "GSI9-659" "GSI9-658"
aKO
"GSI9-657"

$`CKIN1208918-WGS`
$`CKIN1208918-WGS`$`TUSIC-18`
epsilonKO betaKO gammaKO alphaKO deltaKO bKO cKO aKO
"GSIC-57" "GSIC-56" "GSIC-55" "GSIC-54" "GSIC-53" "GSIC-52" "GSIC-51" "GSIC-50"

$HFEL936155
$HFEL936155$`TUHMC-545`
epsilonKO betaKO gammaKO alphaKO deltaKO bKO2
"GHMC-1473" "GHMC-1472" "GHMC-1471" "GHMC-1470" "GHMC-1469" "GHMC-1468"
bKO1
"GHMC-1467"

$HFEL936155$`TUHMC-116`
cKO

"GHMC-287"

$HFEL936155$`TUHMC-426`
aKO
"GHMC-1117"

$`RSOL859655-WGS`
$`RSOL859655-WGS`$`TUSTC-1019`
epsilonKO1 betaKO gammaKO alphaKO deltaKO bKO cKO
"GSTC-149" "GSTC-148" "GSTC-147" "GSTC-146" "GSTC-145" "GSTC-144" "GSTC-143"
aKO
"GSTC-142"

$`RSOL859655-WGS`$`TUSTC-429`
epsilonKO2
"GSTC-4286"

$MGAL708616
$MGAL708616$`TULH2-245`
aKO cKO bKO deltaKO alphaKO1 gammaKO betaKO1
"GLH2-556" "GLH2-555" "GLH2-554" "GLH2-553" "GLH2-552" "GLH2-551" "GLH2-550"
epsilonKO
"GLH2-549"

$MGAL708616$`TULH2-320`
betaKO2 alphaKO2
"GLH2-731" "GLH2-730"

$`CKIN1208919-WGS`
$`CKIN1208919-WGS`$`TUSID-20`
epsilonKO betaKO gammaKO alphaKO deltaKO bKO cKO aKO
"GSID-57" "GSID-56" "GSID-55" "GSID-54" "GSID-53" "GSID-52" "GSID-51" "GSID-50"

$KSP983548
$KSP983548$`TUHAF-704`
gammaKO alphaKO deltaKO bKO cKO aKO
"GHAF-1246" "GHAF-1245" "GHAF-1244" "GHAF-1243" "GHAF-1242" "GHAF-1241"

$KSP983548$`TUHAF-543`
betaKO
"GHAF-941"

$KSP983548$`TUHAF-542`
epsilonKO
"GHAF-940"

$AORE350688
$AORE350688$`TUHBG-1519`
aKO cKO bKO deltaKO alphaKO gammaKO

"GHBG-2679" "GHBG-2678" "GHBG-2677" "GHBG-2676" "GHBG-2675" "GHBG-2674"
betaKO epsilonKO
"GHBG-2673" "GHBG-2672"

$`AORI1156913-WGS`
$`AORI1156913-WGS`$`TUSF9-3242`
alphaKO gammaKO betaKO
"GSF9-6613" "GSF9-6612" "GSF9-6611"

$`AORI1156913-WGS`$`TUSF9-3243`
cKO bKO deltaKO
"GSF9-6616" "GSF9-6615" "GSF9-6614"

$`AORI1156913-WGS`$`TUSF9-3241`
epsilonKO
"GSF9-6610"

$`AORI1156913-WGS`$`TUSF9-3244`
aKO
"GSF9-6617"

$APLE537457
$APLE537457$`TUJI0-1023`
aKO cKO bKO deltaKO alphaKO gammaKO
"GJI0-1778" "GJI0-1777" "GJI0-1776" "GJI0-1775" "GJI0-1774" "GJI0-1773"
betaKO epsilonKO
"GJI0-1772" "GJI0-1771"

$PMAR488538
$PMAR488538$`TUI1I-941`
gammaKO alphaKO deltaKO
"GI1I-1867" "GI1I-1866" "GI1I-1865"

$PMAR488538$`TUI1I-942`
epsilonKO betaKO
"GI1I-1869" "GI1I-1868"

$PMAR488538$`TUI1I-571`
bKO2 bKO1 cKO aKO
"GI1I-1119" "GI1I-1118" "GI1I-1117" "GI1I-1116"

$KFLA479435
$KFLA479435$`TUI0F-2631`
aKO cKO bKO deltaKO alphaKO gammaKO
"GI0F-5095" "GI0F-5094" "GI0F-5093" "GI0F-5092" "GI0F-5091" "GI0F-5090"
betaKO epsilonKO
"GI0F-5089" "GI0F-5088"

$`CKIN1208921-WGS`

```
$`CKIN1208921-WGS`$`TUSIE-23`
epsilonKO    betaKO   gammaKO   alphaKO   deltaKO       bKO       cKO       aKO 
"GSIE-58" "GSIE-57" "GSIE-56" "GSIE-55" "GSIE-54" "GSIE-53" "GSIE-52" "GSIE-51" 


$KKOR523791
$KKOR523791$`TUHCO-1387`
        aKO         cKO         bKO     deltaKO     alphaKO     gammaKO 
"GHCO-2685" "GHCO-2684" "GHCO-2683" "GHCO-2682" "GHCO-2681" "GHCO-2680" 
     betaKO   epsilonKO 
"GHCO-2679" "GHCO-2678" 


$RSOL267608
$RSOL267608$`TUCVU-2698`
        aKO         cKO         bKO     deltaKO     alphaKO     gammaKO 
"GCVU-3384" "GCVU-3383" "GCVU-3382" "GCVU-3381" "GCVU-3380" "GCVU-3379" 
     betaKO  epsilonKO1 
"GCVU-3378" "GCVU-3377" 

$RSOL267608$`TUCVU-430`
 epsilonKO2 
"GCVU-4314" 


$KOXY1191061
$KOXY1191061$`TULEY-3233`
        aKO         cKO         bKO     deltaKO     alphaKO     gammaKO 
"GLEY-5821" "GLEY-5820" "GLEY-5819" "GLEY-5818" "GLEY-5817" "GLEY-5816" 
     betaKO   epsilonKO 
"GLEY-5815" "GLEY-5814" 


$MGAL710128
$MGAL710128$`TULH7-338`
   betaKO1   alphaKO1 
"GLH7-743" "GLH7-742" 

$MGAL710128$`TULH7-158`
 epsilonKO    betaKO2    gammaKO   alphaKO2    deltaKO        bKO        cKO 
"GLH7-360" "GLH7-359" "GLH7-358" "GLH7-357" "GLH7-356" "GLH7-355" "GLH7-354" 
       aKO 
"GLH7-353" 


$KOLE521045
$KOLE521045$`TUHRV-94`
       aKO        cKO        bKO    deltaKO    alphaKO    gammaKO     betaKO 
"GHRV-233" "GHRV-232" "GHRV-231" "GHRV-230" "GHRV-229" "GHRV-228" "GHRV-227" 
 epsilonKO 
"GHRV-226" 


$`CKIN1208920-WGS`
```

```
$`CKIN1208920-WGS`$`TUSIF-19`
epsilonKO    betaKO   gammaKO   alphaKO   deltaKO       bKO       cKO       aKO 
"GSIF-58" "GSIF-57" "GSIF-56" "GSIF-55" "GSIF-54" "GSIF-53" "GSIF-52" "GSIF-51" 


$KOXY1006551
$KOXY1006551$`TUH6O-714`
         aKO          cKO          bKO      deltaKO      alphaKO      gammaKO 
"GH6O-1356" "GH6O-1355" "GH6O-1354" "GH6O-1353" "GH6O-1352" "GH6O-1351" 
     betaKO    epsilonKO 
"GH6O-1350" "GH6O-1349" 


$KPNE507522
$KPNE507522$`TUI0B-3074`
  epsilonKO       betaKO      gammaKO      alphaKO      deltaKO          bKO 
"GI0B-5546" "GI0B-5545" "GI0B-5544" "GI0B-5543" "GI0B-5542" "GI0B-5541" 
         cKO          aKO 
"GI0B-5540" "GI0B-5539" 


$`KPNE1244085-WGS`
$`KPNE1244085-WGS`$`TUSOR-2217`
         aKO          cKO          bKO      deltaKO      alphaKO      gammaKO 
"GSOR-4212" "GSOR-4211" "GSOR-4210" "GSOR-4209" "GSOR-4208" "GSOR-4207" 
     betaKO    epsilonKO 
"GSOR-4206" "GSOR-4205" 


$`KPNE1380908-WGS`
$`KPNE1380908-WGS`$`TUSOT-13`
epsilonKO    betaKO   gammaKO   alphaKO   deltaKO       bKO       cKO       aKO 
"GSOT-11" "GSOT-10"  "GSOT-9"  "GSOT-8"  "GSOT-7"  "GSOT-6"  "GSOT-5"  "GSOT-4" 


$KPNE1125630
$KPNE1125630$`TUJUV-244`
         aKO          cKO          bKO      deltaKO      alphaKO      gammaKO 
"GJUV-5401" "GJUV-5400" "GJUV-5399" "GJUV-5398" "GJUV-5397" "GJUV-5396" 
     betaKO    epsilonKO 
"GJUV-5395" "GJUV-5394" 


$KPNE272620
$KPNE272620$`TUKDC-2479`
         aKO          cKO          bKO      deltaKO      alphaKO      gammaKO 
"GKDC-4180" "GKDC-4179" "GKDC-4178" "GKDC-4177" "GKDC-4176" "GKDC-4175" 
     betaKO    epsilonKO 
"GKDC-4174" "GKDC-4173" 


$RSPH272943
$RSPH272943$`TUJAS-705`
epsilonKO1    betaKO1   gammaKO1   alphaKO1    deltaKO 
```

"GJAS-912" "GJAS-911" "GJAS-910" "GJAS-909" "GJAS-908"

$RSPH272943$`TUJAS-196`
aKO2 bKO3 alphaKO2 gammaKO2
"GJAS-4136" "GJAS-4135" "GJAS-4134" "GJAS-4133"

$RSPH272943$`TUJAS-197`
betaKO2 epsilonKO2
"GJAS-4139" "GJAS-4138"

$RSPH272943$`TUJAS-1600`
aKO1 cKO
"GJAS-2717" "GJAS-2716"

$RSPH272943$`TUJAS-1599`
bKO2 bKO1
"GJAS-2715" "GJAS-2714"

$KPNE1049565
$KPNE1049565$`TULF0-2656`
aKO cKO bKO deltaKO alphaKO gammaKO
"GLF0-4784" "GLF0-4783" "GLF0-4782" "GLF0-4781" "GLF0-4780" "GLF0-4779"
betaKO epsilonKO
"GLF0-4778" "GLF0-4777"

$KPNE1193292
$KPNE1193292$`TULEZ-2661`
epsilonKO betaKO gammaKO alphaKO deltaKO bKO
"GLEZ-5045" "GLEZ-5044" "GLEZ-5043" "GLEZ-5042" "GLEZ-5041" "GLEZ-5040"
cKO aKO
"GLEZ-5039" "GLEZ-5038"

$MGIL350054
$MGIL350054$`TUHK8-1246`
epsilonKO betaKO gammaKO alphaKO deltaKO bKO
"GHK8-2341" "GHK8-2340" "GHK8-2339" "GHK8-2338" "GHK8-2337" "GHK8-2336"
cKO aKO
"GHK8-2335" "GHK8-2334"

$KPNE484021
$KPNE484021$`TUCWL-146`
aKO cKO bKO deltaKO alphaKO gammaKO
"GCWL-5113" "GCWL-5112" "GCWL-5111" "GCWL-5110" "GCWL-5109" "GCWL-5108"
betaKO epsilonKO
"GCWL-5107" "GCWL-5106"

$KRAD266940
$KRAD266940$`TUI4N-590`
aKO cKO bKO deltaKO alphaKO gammaKO betaKO

"GI4N-964" "GI4N-963" "GI4N-962" "GI4N-961" "GI4N-960" "GI4N-959" "GI4N-958"
epsilonKO
"GI4N-957"

$KRHI378753
$KRHI378753$`TUJ8F-566`
epsilonKO betaKO gammaKO alphaKO deltaKO bKO
"GJ8F-1004" "GJ8F-1003" "GJ8F-1002" "GJ8F-1001" "GJ8F-1000" "GJ8F-999"
cKO aKO
"GJ8F-998" "GJ8F-997"

$KSED478801
$KSED478801$`TUI4L-905`
aKO cKO bKO deltaKO alphaKO gammaKO
"GI4L-1818" "GI4L-1817" "GI4L-1816" "GI4L-1815" "GI4L-1814" "GI4L-1813"
betaKO
"GI4L-1812"

$KSED478801$`TUI4L-903`
epsilonKO
"GI4L-1809"

$KSET452652
$KSET452652$`TUJFD-3084`
epsilonKO betaKO gammaKO alphaKO deltaKO bKO
"GJFD-5022" "GJFD-5021" "GJFD-5020" "GJFD-5019" "GJFD-5018" "GJFD-5017"
cKO aKO
"GJFD-5016" "GJFD-5015"

$KVAR640131
$KVAR640131$`TUHXG-2679`
epsilonKO betaKO gammaKO alphaKO deltaKO bKO
"GHXG-5196" "GHXG-5195" "GHXG-5194" "GHXG-5193" "GHXG-5192" "GHXG-5191"
cKO aKO
"GHXG-5190" "GHXG-5189"

$KVUL759362
$KVUL759362$`TULEX-445`
epsilonKO betaKO gammaKO alphaKO deltaKO
"GLEX-475" "GLEX-474" "GLEX-473" "GLEX-472" "GLEX-471"

$KVUL759362$`TULEX-1438`
bKO2 bKO1 cKO aKO
"GLEX-2549" "GLEX-2548" "GLEX-2547" "GLEX-2546"

$RSLO941638
$RSLO941638$`TUJCO-775`
deltaKO alphaKO gammaKO betaKO epsilonKO

"GJCO-1199" "GJCO-1198" "GJCO-1196" "GJCO-1195" "GJCO-1194"

$RSLO941638$`TUJCO-21`
cKO bKO2 bKO1
"GJCO-33" "GJCO-32" "GJCO-31"

$RSLO941638$`TUJCO-75`
aKO1
"GJCO-115"

$RSLO941638$`TUJCO-22`
aKO2
"GJCO-35"

$KVUL880591
$KVUL880591$`TUHQW-639`
epsilonKO betaKO gammaKO alphaKO deltaKO
"GHQW-932" "GHQW-931" "GHQW-930" "GHQW-929" "GHQW-928"

$KVUL880591$`TUHQW-271`
bKO2 bKO1 cKO aKO
"GHQW-96" "GHQW-95" "GHQW-94" "GHQW-93"

$`CLIB1174529-WGS`
$`CLIB1174529-WGS`$`TUSIG-380`
epsilonKO betaKO gammaKO alphaKO deltaKO
"GSIG-596" "GSIG-595" "GSIG-594" "GSIG-593" "GSIG-592"

$`CLIB1174529-WGS`$`TUSIG-618`
aKO cKO bKO2 bKO1
"GSIG-996" "GSIG-995" "GSIG-994" "GSIG-993"

$`LACI1314884-WGS`
$`LACI1314884-WGS`$`TUSOV-411`
epsilonKO betaKO gammaKO alphaKO deltaKO bKO cKO
"GSOV-798" "GSOV-797" "GSOV-796" "GSOV-795" "GSOV-794" "GSOV-793" "GSOV-792"
aKO
"GSOV-791"

$MSP156889
$MSP156889$`TUH36-1930`
deltaKO alphaKO gammaKO betaKO epsilonKO
"GH36-3508" "GH36-3507" "GH36-3506" "GH36-3505" "GH36-3504"

$MSP156889$`TUH36-2064`
bKO cKO aKO
"GH36-3731" "GH36-3730" "GH36-3729"

$`LANG882944-WGS`

$`LANG882944-WGS`$`TUSQ1-672`
 epsilonKO   betaKO   gammaKO   alphaKO   deltaKO   bKO   cKO
"GSQ1-236" "GSQ1-235" "GSQ1-234" "GSQ1-233" "GSQ1-232" "GSQ1-231" "GSQ1-230"
       aKO
"GSQ1-229"

$LACI891391
$LACI891391$`TUHOY-427`
 epsilonKO   betaKO   gammaKO   alphaKO   deltaKO   bKO   cKO
"GHOY-808" "GHOY-807" "GHOY-806" "GHOY-805" "GHOY-804" "GHOY-803" "GHOY-802"
       aKO
"GHOY-801"

$LAMY695560
$LAMY695560$`TUI0Z-478`
 epsilonKO   betaKO   gammaKO   alphaKO   deltaKO   bKO   cKO
"GI0Z-843" "GI0Z-842" "GI0Z-841" "GI0Z-840" "GI0Z-839" "GI0Z-838" "GI0Z-837"
       aKO
"GI0Z-836"

$MGEN662946
$MGEN662946$`TULHD-160`
       aKO   cKO   bKO   deltaKO   alphaKO   betaKO epsilonKO
"GLHD-460" "GLHD-459" "GLHD-458" "GLHD-457" "GLHD-456" "GLHD-455" "GLHD-454"

$MGEN662946$noTU
gammaKO
     NA

$APAS634457
$APAS634457$`TUL7K-296`
 epsilonKO   betaKO   gammaKO   alphaKO   deltaKO
"GL7K-121" "GL7K-120" "GL7K-119" "GL7K-118" "GL7K-117"

$APAS634457$`TUL7K-1514`
        aKO   cKO
"GL7K-2465" "GL7K-2464"

$APAS634457$`TUL7K-1513`
       bKO2   bKO1
"GL7K-2463" "GL7K-2462"

$ARAD311403
$ARAD311403$`TUHU8-3455`
    deltaKO   alphaKO   gammaKO   betaKO   epsilonKO
"GHU8-3343" "GHU8-3342" "GHU8-3341" "GHU8-3340" "GHU8-3339"

$ARAD311403$`TUHU8-2008`
      bKO2   bKO1   cKO   aKO

"GHU8-874" "GHU8-873" "GHU8-872" "GHU8-871"

$`CSAC1332188-WGS`
$`CSAC1332188-WGS`$`TUSJ2-427`
epsilonKO2 betaKO gammaKO alphaKO deltaKO bKO cKO
"GSJ2-933" "GSJ2-932" "GSJ2-930" "GSJ2-928" "GSJ2-927" "GSJ2-926" "GSJ2-925"
aKO
"GSJ2-924"

$`CSAC1332188-WGS`$`TUSJ2-200`
epsilonKO1
"GSJ2-439"

$ASP944547
$ASP944547$`TUL86-908`
bKO2 bKO1 deltaKO alphaKO gammaKO betaKO
"GL86-2109" "GL86-2108" "GL86-2107" "GL86-2106" "GL86-2105" "GL86-2104"
epsilonKO
"GL86-2103"

$ASP944547$`TUL86-977`
cKO
"GL86-2278"

$ASP944547$`TUL86-1091`
aKO
"GL86-2584"

$`APLA696747-WGS`
$`APLA696747-WGS`$`TUSFH-544`
aKO cKO bKO2 bKO1 deltaKO alphaKO gammaKO
"GSFH-830" "GSFH-829" "GSFH-828" "GSFH-827" "GSFH-826" "GSFH-825" "GSFH-824"

$`APLA696747-WGS`$`TUSFH-2077`
betaKO epsilonKO
"GSFH-3178" "GSFH-3177"

$LBRE387344
$LBRE387344$`TUJ8S-722`
aKO cKO bKO deltaKO alphaKO gammaKO
"GJ8S-1285" "GJ8S-1284" "GJ8S-1283" "GJ8S-1282" "GJ8S-1281" "GJ8S-1280"
betaKO epsilonKO
"GJ8S-1279" "GJ8S-1278"

$MGEN662947
$MGEN662947$`TULHB-150`
aKO cKO bKO deltaKO alphaKO gammaKO betaKO
"GLHB-481" "GLHB-480" "GLHB-479" "GLHB-478" "GLHB-477" "GLHB-476" "GLHB-475"
epsilonKO

```
"GLHB-474"


$MGEN662945
$MGEN662945$`TULHE-167`
      aKO        cKO        bKO    deltaKO    alphaKO    gammaKO     betaKO
"GLHE-486" "GLHE-485" "GLHE-484" "GLHE-483" "GLHE-482" "GLHE-481" "GLHE-480"
 epsilonKO
"GLHE-479"


$MHAE941640
$MHAE941640$`TUJSK-29`
   betaKO    gammaKO    alphaKO    deltaKO        bKO        cKO        aKO
"GJSK-146" "GJSK-145" "GJSK-144" "GJSK-143" "GJSK-142" "GJSK-141" "GJSK-140"

$MHAE941640$`TUJSK-416`
  epsilonKO
"GJSK-1527"


$`MHAE1261126-WGS`
$`MHAE1261126-WGS`$`TUSQ2-864`
       aKO         cKO         bKO     deltaKO     alphaKO     gammaKO
"GSQ2-1596" "GSQ2-1595" "GSQ2-1594" "GSQ2-1593" "GSQ2-1592" "GSQ2-1591"
     betaKO   epsilonKO
"GSQ2-1590" "GSQ2-1589"


$`MHAE1366053-WGS`
$`MHAE1366053-WGS`$`TUSQ7-17`
epsilonKO    betaKO   gammaKO   alphaKO   deltaKO       bKO       cKO       aKO
"GSQ7-36" "GSQ7-35" "GSQ7-34" "GSQ7-33" "GSQ7-32" "GSQ7-31" "GSQ7-30" "GSQ7-29"


$`MHAE1311759-WGS`
$`MHAE1311759-WGS`$`TUSRI-801`
  epsilonKO      betaKO     gammaKO     alphaKO     deltaKO         bKO
"GSRI-1482" "GSRI-1481" "GSRI-1480" "GSRI-1479" "GSRI-1478" "GSRI-1477"
        cKO         aKO
"GSRI-1476" "GSRI-1475"


$`MHAE1311760-WGS`
$`MHAE1311760-WGS`$`TUSQ4-885`
  epsilonKO      betaKO     gammaKO     alphaKO     deltaKO         bKO
"GSQ4-1638" "GSQ4-1637" "GSQ4-1636" "GSQ4-1635" "GSQ4-1634" "GSQ4-1633"
        cKO         aKO
"GSQ4-1632" "GSQ4-1631"


$SERY405948
$SERY405948$`TUD36-215114`
        aKO2            cKO            bKO        deltaKO        alphaKO
```

"GD36-206150" "GD36-206149" "GD36-206148" "GD36-206147" "GD36-206146"
gammaKO betaKO epsilonKO
"GD36-206145" "GD36-206144" "GD36-206143"

$SERY405948$`TUD36-214563`
aKO1
"GD36-205222"

$`CMYC1116213-WGS`
$`CMYC1116213-WGS`$`TUSIL-109`
aKO bKO deltaKO alphaKO gammaKO
"GSIL-301" "GSIL-300" "GSIL-299" "GSIL-298" "GSIL-297"

$`CMYC1116213-WGS`$`TUSIL-107`
betaKO
"GSIL-294"

$`CMYC1116213-WGS`$`TUSIL-101`
epsilonKO
"GSIL-278"

$`CMYC1116213-WGS`$`TUSIL-136`
cKO
"GSIL-351"

$MHYD1163748
$MHYD1163748$`TULG7-1926`
aKO cKO bKO deltaKO alphaKO gammaKO
"GLG7-3827" "GLG7-3826" "GLG7-3825" "GLG7-3824" "GLG7-3823" "GLG7-3822"
betaKO epsilonKO
"GLG7-3821" "GLG7-3820"

$MHAE1111676
$MHAE1111676$`TUJSM-33`
betaKO gammaKO alphaKO deltaKO bKO cKO aKO
"GJSM-144" "GJSM-143" "GJSM-142" "GJSM-141" "GJSM-140" "GJSM-139" "GJSM-138"

$MHAE1111676$`TUJSM-381`
epsilonKO
"GJSM-1136"

$LDEL321956
$LDEL321956$`TUI15-352`
epsilonKO betaKO gammaKO alphaKO deltaKO bKO cKO
"GI15-642" "GI15-641" "GI15-640" "GI15-639" "GI15-638" "GI15-637" "GI15-636"
aKO
"GI15-635"

$MHAE859194

```
$MHAE859194$`TULHF-30`
    betaKO    gammaKO    alphaKO    deltaKO        bKO        cKO        aKO 
"GLHF-147" "GLHF-146" "GLHF-145" "GLHF-144" "GLHF-143" "GLHF-142" "GLHF-141" 

$MHAE859194$noTU
epsilonKO 
       NA 



$`MHYP657316-WGS`
$`MHYP657316-WGS`$`TUSQ8-1308`
        aKO         cKO     deltaKO     alphaKO   epsilonKO 
"GSQ8-2120" "GSQ8-2119" "GSQ8-2118" "GSQ8-2117" "GSQ8-2116" 

$`MHYP657316-WGS`$noTU
 betaKO gammaKO     bKO 
     NA      NA      NA 



$MHYO1129369
$MHYO1129369$`TUJVH-176`
       aKO        cKO        bKO    deltaKO   alphaKO1    gammaKO    betaKO1 
"GJVH-371" "GJVH-370" "GJVH-369" "GJVH-368" "GJVH-367" "GJVH-366" "GJVH-365" 
 epsilonKO 
"GJVH-364" 

$MHYO1129369$`TUJVH-319`
  alphaKO2    betaKO2 
"GJVH-710" "GJVH-709" 



$MHYO262719
$MHYO262719$`TUJ59-23`
  betaKO1   gammaKO  alphaKO1   deltaKO       bKO       cKO       aKO 
"GJ59-49" "GJ59-48" "GJ59-47" "GJ59-46" "GJ59-45" "GJ59-44" "GJ59-43" 

$MHYO262719$`TUJ59-254`
  alphaKO2    betaKO2 
"GJ59-497" "GJ59-496" 

$MHYO262719$noTU
epsilonKO 
       NA 



$`CMYC1212765-WGS`
$`CMYC1212765-WGS`$`TUSIK-128`
   gammaKO    alphaKO    deltaKO        bKO       cKO1        aKO 
"GSIK-301" "GSIK-300" "GSIK-299" "GSIK-298" "GSIK-297" "GSIK-296" 

$`CMYC1212765-WGS`$`TUSIK-131`
    betaKO 
"GSIK-304" 
```

```
$`CMYC1212765-WGS`$`TUSIK-180`
 epsilonKO
"GSIK-401"

$`CMYC1212765-WGS`$`TUSIK-146`
      cKO2
"GSIK-334"


$MHYO936139
$MHYO936139$`TULHH-86`
       aKO        cKO        bKO    deltaKO   alphaKO1    gammaKO    betaKO1
"GLHH-175" "GLHH-174" "GLHH-173" "GLHH-172" "GLHH-171" "GLHH-170" "GLHH-169"
 epsilonKO
"GLHH-168"

$MHYO936139$`TULHH-230`
   betaKO2   alphaKO2
"GLHH-460" "GLHH-459"


$LPNE423212
$LPNE423212$`TUHRR-628`
       aKO1        cKO1        bKO1    alphaKO1    gammaKO1
"GHRR-1191" "GHRR-1190" "GHRR-1189" "GHRR-1188" "GHRR-1187"

$LPNE423212$`TUHRR-1757`
       aKO2        cKO2        bKO2     deltaKO    alphaKO2    gammaKO2
"GHRR-3225" "GHRR-3224" "GHRR-3223" "GHRR-3222" "GHRR-3221" "GHRR-3220"
    betaKO2  epsilonKO2
"GHRR-3219" "GHRR-3218"

$LPNE423212$`TUHRR-629`
    betaKO1  epsilonKO1
"GHRR-1194" "GHRR-1193"


$MHYO907287
$MHYO907287$`TULHG-27`
epsilonKO   betaKO1   gammaKO  alphaKO1   deltaKO       bKO       cKO       aKO
"GLHG-54" "GLHG-53" "GLHG-52" "GLHG-51" "GLHG-50" "GLHG-49" "GLHG-48" "GLHG-47"

$MHYO907287$`TULHG-273`
  alphaKO2    betaKO2
"GLHG-520" "GLHG-519"


$MHYO262722
$MHYO262722$`TUHZR-24`
   betaKO   gammaKO  alphaKO1   deltaKO       bKO       cKO       aKO
"GHZR-53" "GHZR-52" "GHZR-51" "GHZR-50" "GHZR-49" "GHZR-48" "GHZR-47"

$MHYO262722$`TUHZR-253`
  alphaKO2
```

"GHZR-500"

$MHYO262722$noTU
epsilonKO
NA

$`MHAE1249531-WGS`
$`MHAE1249531-WGS`$`TUSQ6-14`
aKO cKO bKO deltaKO alphaKO gammaKO betaKO epsilonKO
"GSQ6-32" "GSQ6-31" "GSQ6-30" "GSQ6-29" "GSQ6-28" "GSQ6-27" "GSQ6-26" "GSQ6-25"

$MHYO1118964
$MHYO1118964$`TULHI-129`
epsilonKO betaKO1 gammaKO alphaKO1 deltaKO bKO cKO
"GLHI-250" "GLHI-249" "GLHI-248" "GLHI-247" "GLHI-246" "GLHI-245" "GLHI-244"
aKO
"GLHI-243"

$MHYO1118964$`TULHI-372`
alphaKO2 betaKO2
"GLHI-756" "GLHI-755"

$LBYS649349
$LBYS649349$`TUHFA-776`
alphaKO gammaKO
"GHFA-1633" "GHFA-1632"

$LBYS649349$`TUHFA-1355`
epsilonKO betaKO
"GHFA-2811" "GHFA-2810"

$LBYS649349$`TUHFA-1764`
aKO cKO bKO deltaKO
"GHFA-3665" "GHFA-3664" "GHFA-3663" "GHFA-3662"

$`MHAE1249526-WGS`
$`MHAE1249526-WGS`$`TUSQ5-22`
epsilonKO betaKO gammaKO alphaKO deltaKO bKO cKO aKO
"GSQ5-51" "GSQ5-50" "GSQ5-49" "GSQ5-48" "GSQ5-47" "GSQ5-46" "GSQ5-45" "GSQ5-44"

$`MHAE1316932-WGS`
$`MHAE1316932-WGS`$`TUSQ3-302`
epsilonKO betaKO gammaKO alphaKO deltaKO bKO cKO
"GSQ3-584" "GSQ3-583" "GSQ3-582" "GSQ3-581" "GSQ3-580" "GSQ3-579" "GSQ3-578"
aKO
"GSQ3-577"

$`MHYO1116211-WGS`

$`MHYO1116211-WGS`$`TUSRQ-28`
epsilonKO   betaKO1   gammaKO alphaKO1   deltaKO   bKO   cKO   aKO
"GSRQ-54" "GSRQ-53" "GSRQ-52" "GSRQ-51" "GSRQ-50" "GSRQ-49" "GSRQ-48" "GSRQ-47"

$`MHYO1116211-WGS`$`TUSRQ-273`
alphaKO2   betaKO2
"GSRQ-517" "GSRQ-516"

$`MHYO754503-WGS`
$`MHYO754503-WGS`$`TUSRR-274`
alphaKO1   betaKO2
"GSRR-518" "GSRR-517"

$`MHYO754503-WGS`$`TUSRR-30`
betaKO1   gammaKO alphaKO2   deltaKO   bKO   cKO   aKO
"GSRR-54" "GSRR-53" "GSRR-52" "GSRR-51" "GSRR-50" "GSRR-49" "GSRR-48"

$`MHYO754503-WGS`$noTU
epsilonKO
NA

$MINT487521
$MINT487521$`TULGN-836`
epsilonKO   betaKO   gammaKO   alphaKO   bKO   cKO
"GLGN-1631" "GLGN-1630" "GLGN-1629" "GLGN-1628" "GLGN-1626" "GLGN-1625"
aKO
"GLGN-1624"

$MINT487521$noTU
deltaKO
NA

$`BBAC264462-WGS`
$`BBAC264462-WGS`$`TUSGK-1788`
bKO2   bKO1   deltaKO   alphaKO   gammaKO   betaKO
"GSGK-3617" "GSGK-3616" "GSGK-3615" "GSGK-3614" "GSGK-3613" "GSGK-3612"
epsilonKO
"GSGK-3611"

$`BBAC264462-WGS`$`TUSGK-3`
cKO   aKO
"GSGK-10" "GSGK-9"

$MSP1173027
$MSP1173027$`TULGJ-2459`
bKO1   deltaKO   alphaKO   gammaKO
"GLGJ-3241" "GLGJ-3240" "GLGJ-3239" "GLGJ-3238"

$MSP1173027$`TULGJ-2139`
epsilonKO   betaKO

"GLGJ-2745" "GLGJ-2744"

$MSP1173027$`TULGJ-2461`
aKO cKO
"GLGJ-3244" "GLGJ-3243"

$MSP1173027$`TULGJ-2460`
bKO2
"GLGJ-3242"

$`MIND1232724-WGS`
$`MIND1232724-WGS`$`TUSQZ-795`
epsilonKO betaKO gammaKO alphaKO bKO cKO
"GSQZ-1542" "GSQZ-1541" "GSQZ-1540" "GSQZ-1539" "GSQZ-1537" "GSQZ-1536"
aKO
"GSQZ-1535"

$`MIND1232724-WGS`$noTU
deltaKO
NA

$MSP648999
$MSP648999$`TUHVN-1718`
betaKO gammaKO alphaKO deltaKO bKO cKO
"GHVN-3224" "GHVN-3223" "GHVN-3222" "GHVN-3221" "GHVN-3220" "GHVN-3219"
aKO
"GHVN-3218"

$MSP648999$`TUHVN-1720`
epsilonKO
"GHVN-3226"

$`MINT1138383-WGS`
$`MINT1138383-WGS`$`TUSR0-727`
epsilonKO betaKO gammaKO alphaKO bKO cKO
"GSR0-1377" "GSR0-1376" "GSR0-1375" "GSR0-1374" "GSR0-1372" "GSR0-1371"
aKO
"GSR0-1370"

$`MINT1138383-WGS`$noTU
deltaKO
NA

$MINT1138382
$MINT1138382$`TULGO-818`
epsilonKO betaKO gammaKO alphaKO bKO cKO
"GLGO-1609" "GLGO-1608" "GLGO-1607" "GLGO-1606" "GLGO-1604" "GLGO-1603"
aKO
"GLGO-1602"

```
$MINT1138382$noTU
deltaKO
     NA



$LCAS321967
$LCAS321967$`TUH4S-670`
  epsilonKO      betaKO     gammaKO     alphaKO     deltaKO         bKO
"GH4S-1166" "GH4S-1165" "GH4S-1164" "GH4S-1163" "GH4S-1162" "GH4S-1161"
        cKO         aKO
"GH4S-1160" "GH4S-1159"



$MSP875328
$MSP875328$`TUHLX-709`
  epsilonKO      betaKO     gammaKO     alphaKO         bKO         cKO
"GHLX-1328" "GHLX-1327" "GHLX-1326" "GHLX-1325" "GHLX-1323" "GHLX-1322"
        aKO
"GHLX-1321"

$MSP875328$noTU
deltaKO
     NA



$MSP164757
$MSP164757$`TUHV3-1677`
        aKO         cKO         bKO     deltaKO     alphaKO     gammaKO
"GHV3-3902" "GHV3-3901" "GHV3-3900" "GHV3-3899" "GHV3-3898" "GHV3-3897"
     betaKO   epsilonKO
"GHV3-3896" "GHV3-3895"



$MSP189918
$MSP189918$`TUH4X-2006`
        aKO         cKO         bKO     deltaKO     alphaKO     gammaKO
"GH4X-3994" "GH4X-3993" "GH4X-3992" "GH4X-3991" "GH4X-3990" "GH4X-3989"
     betaKO   epsilonKO
"GH4X-3988" "GH4X-3987"



$ASP1118963
$ASP1118963$`TUL88-1585`
        aKO         cKO         bKO     deltaKO     alphaKO     gammaKO
"GL88-2756" "GL88-2755" "GL88-2754" "GL88-2753" "GL88-2752" "GL88-2751"
     betaKO   epsilonKO
"GL88-2750" "GL88-2749"



$BBAC1069642
$BBAC1069642$`TUL8W-2035`
       bKO2        bKO1     deltaKO     alphaKO     gammaKO      betaKO
"GL8W-3769" "GL8W-3768" "GL8W-3767" "GL8W-3766" "GL8W-3765" "GL8W-3764"
  epsilonKO
```

```
"GL8W-3763"

$BBAC1069642$`TUL8W-5`
     cKO      aKO 
"GL8W-10"  "GL8W-9" 


$ASP290399
$ASP290399$`TUHIF-1649`
      aKO         cKO         bKO     deltaKO     alphaKO     gammaKO 
"GHIF-2672" "GHIF-2671" "GHIF-2670" "GHIF-2669" "GHIF-2668" "GHIF-2667" 
    betaKO   epsilonKO 
"GHIF-2666" "GHIF-2665" 


$ASAL382245
$ASAL382245$`TUJJN-2705`
      aKO         cKO         bKO     deltaKO     alphaKO     gammaKO 
"GJJN-4340" "GJJN-4339" "GJJN-4338" "GJJN-4337" "GJJN-4336" "GJJN-4335" 
    betaKO   epsilonKO 
"GJJN-4334" "GJJN-4333" 


$ASUB443218
$ASUB443218$`TUH9R-1749`
      aKO         cKO         bKO     deltaKO     alphaKO     gammaKO 
"GH9R-3292" "GH9R-3291" "GH9R-3290" "GH9R-3289" "GH9R-3288" "GH9R-3287" 
    betaKO   epsilonKO 
"GH9R-3286" "GH9R-3285" 


$`MKAN557599-WGS`
$`MKAN557599-WGS`$`TUSS1-704`
      aKO         cKO         bKO     alphaKO     gammaKO      betaKO 
"GSS1-1266" "GSS1-1265" "GSS1-1264" "GSS1-1262" "GSS1-1261" "GSS1-1260" 
  epsilonKO 
"GSS1-1259" 

$`MKAN557599-WGS`$noTU
deltaKO 
    NA 


$MLEP561304
$MLEP561304$`TUJP6-683`
  epsilonKO      betaKO     gammaKO     alphaKO 
"GJP6-1164" "GJP6-1163" "GJP6-1162" "GJP6-1161" 

$MLEP561304$`TUJP6-682`
    deltaKO         bKO         cKO 
"GJP6-1160" "GJP6-1159" "GJP6-1158" 

$MLEP561304$`TUJP6-681`
      aKO 
```

"GJP6-1157"

$MLEA880447
$MLEA880447$`TUC0N-72`
epsilonKO betaKO1 gammaKO alphaKO1 deltaKO bKO cKO
"GC0N-132" "GC0N-131" "GC0N-130" "GC0N-129" "GC0N-128" "GC0N-127" "GC0N-126"
aKO
"GC0N-125"

$MLEA880447$`TUC0N-196`
betaKO2 alphaKO2
"GC0N-369" "GC0N-368"

$LCAS543734
$LCAS543734$`TUCHL-727`
epsilonKO betaKO gammaKO alphaKO deltaKO bKO
"GCHL-1367" "GCHL-1366" "GCHL-1365" "GCHL-1364" "GCHL-1363" "GCHL-1362"
cKO aKO
"GCHL-1361" "GCHL-1360"

$`MLEP272631-WGS`
$`MLEP272631-WGS`$`TUSR3-681`
epsilonKO betaKO gammaKO alphaKO deltaKO bKO
"GSR3-1163" "GSR3-1162" "GSR3-1161" "GSR3-1160" "GSR3-1159" "GSR3-1158"
cKO aKO
"GSR3-1157" "GSR3-1156"

$MLEA866629
$MLEA866629$`TULHJ-49`
epsilonKO betaKO1 gammaKO alphaKO1 deltaKO bKO cKO aKO
"GLHJ-99" "GLHJ-98" "GLHJ-97" "GLHJ-96" "GLHJ-95" "GLHJ-94" "GLHJ-93" "GLHJ-92"

$MLEA866629$`TULHJ-87`
betaKO2 alphaKO2
"GLHJ-172" "GLHJ-171"

$`MLIF459424-WGS`
$`MLIF459424-WGS`$`TUSR5-2192`
aKO cKO bKO alphaKO gammaKO betaKO
"GSR5-4037" "GSR5-4036" "GSR5-4035" "GSR5-4033" "GSR5-4032" "GSR5-4031"
epsilonKO
"GSR5-4030"

$`MLIF459424-WGS`$noTU
deltaKO
NA

$SMUT511691

$SMUT511691$`TUH9C-336`
epsilonKO betaKO gammaKO alphaKO deltaKO bKO aKO
"GH9C-631" "GH9C-630" "GH9C-629" "GH9C-628" "GH9C-627" "GH9C-626" "GH9C-625"
cKO
"GH9C-624"

$MLOT266835
$MLOT266835$`TUJ9L-2116`
deltaKO alphaKO gammaKO betaKO epsilonKO
"GJ9L-3169" "GJ9L-3168" "GJ9L-3167" "GJ9L-3166" "GJ9L-3165"

$MLOT266835$`TUJ9L-3669`
bKO2 bKO1 cKO aKO
"GJ9L-5887" "GJ9L-5886" "GJ9L-5885" "GJ9L-5884"

$MLUT465515
$MLUT465515$`TUHH6-432`
epsilonKO betaKO gammaKO alphaKO deltaKO bKO cKO
"GHH6-817" "GHH6-816" "GHH6-815" "GHH6-814" "GHH6-813" "GHH6-812" "GHH6-811"
aKO
"GHH6-810"

$MMAR477641
$MMAR477641$`TULGK-2398`
aKO cKO bKO deltaKO alphaKO gammaKO
"GLGK-4510" "GLGK-4509" "GLGK-4508" "GLGK-4507" "GLGK-4506" "GLGK-4505"
betaKO
"GLGK-4504"

$MMAR477641$`TULGK-2396`
epsilonKO
"GLGK-4502"

$MMOB583345
$MMOB583345$`TUHCF-1308`
aKO cKO bKO deltaKO alphaKO gammaKO
"GHCF-2392" "GHCF-2391" "GHCF-2390" "GHCF-2389" "GHCF-2388" "GHCF-2387"
betaKO epsilonKO
"GHCF-2386" "GHCF-2385"

$MSP164756
$MSP164756$`TUHQ8-1812`
aKO cKO bKO deltaKO alphaKO gammaKO
"GHQ8-3923" "GHQ8-3922" "GHQ8-3921" "GHQ8-3920" "GHQ8-3919" "GHQ8-3918"
betaKO epsilonKO
"GHQ8-3917" "GHQ8-3916"

$MMED717774

```
$MMED717774$`TUCPW-2353`
        aKO         cKO         bKO     deltaKO     alphaKO     gammaKO 
"GCPW-4322" "GCPW-4321" "GCPW-4320" "GCPW-4319" "GCPW-4318" "GCPW-4317" 
     betaKO   epsilonKO 
"GCPW-4316" "GCPW-4315" 


$MMAR216594
$MMAR216594$`TUJOB-2200`
        aKO         cKO         bKO     deltaKO     alphaKO     gammaKO 
"GJOB-4123" "GJOB-4122" "GJOB-4121" "GJOB-4120" "GJOB-4119" "GJOB-4118" 
     betaKO   epsilonKO 
"GJOB-4117" "GJOB-4116" 


$LCAS999378
$LCAS999378$`TULF5-769`
    gammaKO     alphaKO     deltaKO         bKO         cKO         aKO 
"GLF5-1386" "GLF5-1385" "GLF5-1384" "GLF5-1383" "GLF5-1382" "GLF5-1381" 

$LCAS999378$`TULF5-770`
  epsilonKO      betaKO 
"GLF5-1388" "GLF5-1387" 


$`MMOR1124991-WGS`
$`MMOR1124991-WGS`$`TUSQM-238`
 epsilonKO     betaKO    gammaKO    alphaKO    deltaKO        bKO        cKO 
"GSQM-504" "GSQM-503" "GSQM-502" "GSQM-501" "GSQM-500" "GSQM-499" "GSQM-498" 
       aKO 
"GSQM-497" 


$MSP1168287
$MSP1168287$`TULGR-706`
    gammaKO     alphaKO         bKO         cKO         aKO 
"GLGR-1343" "GLGR-1342" "GLGR-1340" "GLGR-1339" "GLGR-1338" 

$MSP1168287$`TULGR-707`
  epsilonKO      betaKO 
"GLGR-1346" "GLGR-1345" 

$MSP1168287$noTU
deltaKO 
     NA 


$HHEP235279
$HHEP235279$`TUHUA-184`
 epsilonKO     betaKO    gammaKO    alphaKO    deltaKO       bKO2       bKO1 
"GHUA-446" "GHUA-445" "GHUA-444" "GHUA-443" "GHUA-442" "GHUA-441" "GHUA-440" 

$HHEP235279$`TUHUA-261`
       cKO 
```

```
"GHUA-613"

$HHEP235279$`TUHUA-675`
       aKO
"GHUA-1536"


$SMUT1155071
$SMUT1155071$`TULL6-339`
 epsilonKO     betaKO    gammaKO    alphaKO    deltaKO        bKO        aKO
"GLL6-646" "GLL6-645" "GLL6-644" "GLL6-643" "GLL6-642" "GLL6-641" "GLL6-640"
      cKO
"GLL6-639"


$MMIT696127
$MMIT696127$`TUI3H-680`
   deltaKO     alphaKO
"GI3H-1119" "GI3H-1118"

$MMIT696127$`TUI3H-679`
   gammaKO      betaKO   epsilonKO
"GI3H-1117" "GI3H-1116" "GI3H-1115"

$MMIT696127$`TUI3H-42`
     aKO       cKO      bKO2      bKO1
"GI3H-87" "GI3H-86" "GI3H-85" "GI3H-84"


$MMAR394221
$MMAR394221$`TUHNB-1498`
   deltaKO     alphaKO     gammaKO      betaKO   epsilonKO
"GHNB-2858" "GHNB-2857" "GHNB-2856" "GHNB-2855" "GHNB-2853"

$MMAR394221$`TUHNB-1170`
       cKO
"GHNB-2251"

$MMAR394221$`TUHNB-1169`
       aKO
"GHNB-2250"

$MMAR394221$`TUHNB-1171`
      bKO2        bKO1
"GHNB-2253" "GHNB-2252"


$JSP375286
$JSP375286$`TUJ8U-2046`
       aKO         cKO         bKO     deltaKO     alphaKO     gammaKO
"GJ8U-3687" "GJ8U-3686" "GJ8U-3685" "GJ8U-3684" "GJ8U-3683" "GJ8U-3682"
    betaKO   epsilonKO
"GJ8U-3681" "GJ8U-3680"
```

```
$MMET857087
$MMET857087$`TUH4A-245`
   betaKO1 epsilonKO1       aKO1       cKO1       bKO1   alphaKO1   gammaKO1 
"GH4A-510" "GH4A-509" "GH4A-506" "GH4A-505" "GH4A-504" "GH4A-503" "GH4A-502" 

$MMET857087$`TUH4A-426`
  gammaKO2   alphaKO2       bKO2 
"GH4A-867" "GH4A-866" "GH4A-865" 

$MMET857087$`TUH4A-2274`
       aKO3        cKO3        bKO3     deltaKO    alphaKO3    gammaKO3 
"GH4A-4561" "GH4A-4560" "GH4A-4559" "GH4A-4558" "GH4A-4557" "GH4A-4556" 
    betaKO3  epsilonKO3 
"GH4A-4555" "GH4A-4554" 

$MMET857087$`TUH4A-424`
      cKO2       aKO2 epsilonKO2    betaKO2 
"GH4A-862" "GH4A-861" "GH4A-859" "GH4A-858" 


$MMAS1198627
$MMAS1198627$`TULGQ-436`
 epsilonKO     betaKO    gammaKO    alphaKO        bKO        aKO 
"GLGQ-779" "GLGQ-778" "GLGQ-777" "GLGQ-776" "GLGQ-774" "GLGQ-773" 

$MMAS1198627$noTU
deltaKO     cKO 
     NA      NA 


$MSP400668
$MSP400668$`TUHKD-1100`
   gammaKO1    alphaKO1        bKO1        cKO1        aKO1  epsilonKO1 
"GHKD-1985" "GHKD-1984" "GHKD-1983" "GHKD-1982" "GHKD-1981" "GHKD-1978" 
    betaKO1 
"GHKD-1977" 

$MSP400668$`TUHKD-2503`
       aKO2        cKO2        bKO2     deltaKO    alphaKO2    gammaKO2 
"GHKD-4579" "GHKD-4578" "GHKD-4577" "GHKD-4576" "GHKD-4575" "GHKD-4574" 
    betaKO2  epsilonKO2 
"GHKD-4573" "GHKD-4572" 


$`MMYC865867-WGS`
$`MMYC865867-WGS`$`TUSRT-378`
  alphaKO1    betaKO1 
"GSRT-678" "GSRT-677" 

$`MMYC865867-WGS`$`TUSRT-534`
       aKO        cKO        bKO    deltaKO   alphaKO2    gammaKO    betaKO2 
"GSRT-973" "GSRT-972" "GSRT-971" "GSRT-970" "GSRT-969" "GSRT-968" "GSRT-967" 
 epsilonKO 
```

"GSRT-966"

$MOPP536019
$MOPP536019$`TUH56-697`
epsilonKO betaKO gammaKO alphaKO deltaKO
"GH56-1219" "GH56-1218" "GH56-1217" "GH56-1216" "GH56-1215"

$MOPP536019$`TUH56-3253`
aKO cKO
"GH56-5739" "GH56-5738"

$MOPP536019$`TUH56-3252`
bKO2 bKO1
"GH56-5737" "GH56-5736"

$LCHO395495
$LCHO395495$`TUHYL-1792`
aKO cKO bKO deltaKO alphaKO gammaKO
"GHYL-3580" "GHYL-3579" "GHYL-3578" "GHYL-3577" "GHYL-3576" "GHYL-3575"
betaKO epsilonKO
"GHYL-3574" "GHYL-3573"

$`CMET671143-WGS`
$`CMET671143-WGS`$`TUSII-1525`
epsilonKO betaKO gammaKO alphaKO deltaKO bKO
"GSII-2928" "GSII-2927" "GSII-2926" "GSII-2925" "GSII-2924" "GSII-2923"

$`CMET671143-WGS`$`TUSII-1385`
aKO cKO
"GSII-2657" "GSII-2656"

$SMAL522373
$SMAL522373$`TUJE8-2118`
aKO cKO bKO deltaKO alphaKO gammaKO
"GJE8-3977" "GJE8-3976" "GJE8-3975" "GJE8-3974" "GJE8-3973" "GJE8-3972"
betaKO epsilonKO
"GJE8-3971" "GJE8-3970"

$MAVI262316
$MAVI262316$`TUCQR-1260`
aKO cKO bKO deltaKO alphaKO gammaKO
"GCQR-2492" "GCQR-2491" "GCQR-2490" "GCQR-2489" "GCQR-2488" "GCQR-2487"
betaKO epsilonKO
"GCQR-2486" "GCQR-2485"

$`MPNE1238993-WGS`
$`MPNE1238993-WGS`$`TUSS8-237`
aKO cKO bKO deltaKO alphaKO gammaKO betaKO

"GSS8-608" "GSS8-607" "GSS8-606" "GSS8-605" "GSS8-604" "GSS8-603" "GSS8-602"
epsilonKO
"GSS8-601"

$MPOS491952
$MPOS491952$`TUI6N-1895`
aKO cKO bKO deltaKO alphaKO gammaKO
"GI6N-3641" "GI6N-3640" "GI6N-3639" "GI6N-3638" "GI6N-3637" "GI6N-3636"
betaKO epsilonKO
"GI6N-3635" "GI6N-3634"

$MPEN272633
$MPEN272633$`TUJBP-32`
gammaKO alphaKO deltaKO bKO cKO aKO
"GJBP-61" "GJBP-60" "GJBP-59" "GJBP-58" "GJBP-57" "GJBP-56"

$MPEN272633$`TUJBP-33`
epsilonKO betaKO
"GJBP-63" "GJBP-62"

$MPUT743965
$MPUT743965$`TUJUK-44`
epsilonKO betaKO1 gammaKO alphaKO1 deltaKO bKO cKO aKO
"GJUK-80" "GJUK-79" "GJUK-78" "GJUK-77" "GJUK-76" "GJUK-75" "GJUK-74" "GJUK-73"

$MPUT743965$`TUJUK-163`
betaKO2 alphaKO2
"GJUK-342" "GJUK-341"

$MPRI660470
$MPRI660470$`TULGB-447`
aKO cKO bKO deltaKO alphaKO gammaKO
"GLGB-1054" "GLGB-1053" "GLGB-1052" "GLGB-1051" "GLGB-1050" "GLGB-1049"
betaKO epsilonKO
"GLGB-1048" "GLGB-1047"

$MPHO1032480
$MPHO1032480$`TUHBY-1571`
aKO cKO bKO deltaKO alphaKO gammaKO
"GHBY-3040" "GHBY-3039" "GHBY-3038" "GHBY-3037" "GHBY-3036" "GHBY-3035"
betaKO epsilonKO
"GHBY-3034" "GHBY-3033"

$MPNE722438
$MPNE722438$`TULHK-308`
aKO bKO deltaKO alphaKO gammaKO betaKO epsilonKO
"GLHK-704" "GLHK-702" "GLHK-701" "GLHK-700" "GLHK-699" "GLHK-698" "GLHK-697"

```
$MPNE722438$noTU
cKO
 NA


$MPNE1112856
$MPNE1112856$`TUJU8-298`
      aKO       cKO       bKO    deltaKO    alphaKO    gammaKO     betaKO 
"GJU8-663" "GJU8-662" "GJU8-661" "GJU8-660" "GJU8-659" "GJU8-658" "GJU8-657" 
 epsilonKO 
"GJU8-656" 


$LCIT349519
$LCIT349519$`TUHNF-957`
       aKO        cKO        bKO     deltaKO     alphaKO     gammaKO 
"GHNF-1685" "GHNF-1684" "GHNF-1683" "GHNF-1682" "GHNF-1681" "GHNF-1680" 
    betaKO   epsilonKO 
"GHNF-1679" "GHNF-1678" 


$`SMAC1116231-WGS`
$`SMAC1116231-WGS`$`TUSXU-418`
 epsilonKO     betaKO    gammaKO    alphaKO    deltaKO        bKO        aKO 
"GSXU-805" "GSXU-804" "GSXU-803" "GSXU-802" "GSXU-801" "GSXU-800" "GSXU-799" 
      cKO 
"GSXU-798" 


$MPNE272634
$MPNE272634$`TUJ6Z-289`
      aKO       cKO       bKO    deltaKO    alphaKO    gammaKO     betaKO 
"GJ6Z-650" "GJ6Z-649" "GJ6Z-648" "GJ6Z-647" "GJ6Z-646" "GJ6Z-645" "GJ6Z-644" 
 epsilonKO 
"GJ6Z-643" 


$MPOP441620
$MPOP441620$`TUHMI-876`
    deltaKO     alphaKO     gammaKO      betaKO   epsilonKO 
"GHMI-1509" "GHMI-1508" "GHMI-1507" "GHMI-1506" "GHMI-1504" 

$MPOP441620$`TUHMI-2004`
       aKO         cKO 
"GHMI-3426" "GHMI-3425" 

$MPOP441620$`TUHMI-2002`
      bKO1 
"GHMI-3423" 

$MPOP441620$`TUHMI-2003`
      bKO2 
"GHMI-3424" 
```

$MPLU940190
$MPLU940190$`TUH20-973`
aKO cKO bKO deltaKO alphaKO gammaKO
"GH20-1571" "GH20-1570" "GH20-1569" "GH20-1568" "GH20-1567" "GH20-1566"
betaKO epsilonKO
"GH20-1565" "GH20-1564"

$ASP134676
$ASP134676$`TUL7H-3843`
aKO cKO bKO deltaKO alphaKO gammaKO
"GL7H-7438" "GL7H-7437" "GL7H-7436" "GL7H-7435" "GL7H-7434" "GL7H-7433"
betaKO epsilonKO
"GL7H-7432" "GL7H-7430"

$`ASUI696748-WGS`
$`ASUI696748-WGS`$`TUSEQ-1119`
epsilonKO betaKO gammaKO alphaKO deltaKO bKO
"GSEQ-1908" "GSEQ-1907" "GSEQ-1906" "GSEQ-1905" "GSEQ-1904" "GSEQ-1903"
cKO aKO
"GSEQ-1902" "GSEQ-1901"

$ASUB746697
$ASUB746697$`TUL7Y-698`
cKO bKO deltaKO alphaKO gammaKO
"GL7Y-1176" "GL7Y-1175" "GL7Y-1174" "GL7Y-1173" "GL7Y-1172"

$ASUB746697$`TUL7Y-800`
betaKO
"GL7Y-1360"

$ASUB746697$`TUL7Y-799`
epsilonKO
"GL7Y-1359"

$ASUB746697$`TUL7Y-699`
aKO
"GL7Y-1177"

$ASUC339671
$ASUC339671$`TUHDX-182`
aKO cKO bKO deltaKO alphaKO gammaKO betaKO
"GHDX-350" "GHDX-349" "GHDX-348" "GHDX-347" "GHDX-346" "GHDX-345" "GHDX-344"
epsilonKO
"GHDX-343"

$MPET420662
$MPET420662$`TUHBE-425`
epsilonKO betaKO gammaKO alphaKO deltaKO bKO cKO

"GHBE-198" "GHBE-197" "GHBE-196" "GHBE-195" "GHBE-194" "GHBE-193" "GHBE-192"
aKO
"GHBE-191"

$`MPLU1090974-WGS`
$`MPLU1090974-WGS`$`TUSQA-365`
epsilonKO betaKO gammaKO alphaKO deltaKO bKO cKO
"GSQA-483" "GSQA-482" "GSQA-481" "GSQA-480" "GSQA-479" "GSQA-478" "GSQA-477"
aKO
"GSQA-476"

$MPIE443254
$MPIE443254$`TUI5V-592`
epsilonKO betaKO gammaKO alphaKO deltaKO bKO
"GI5V-1596" "GI5V-1595" "GI5V-1594" "GI5V-1593" "GI5V-1592" "GI5V-1591"
cKO
"GI5V-1590"

$MPIE443254$`TUI5V-591`
aKO
"GI5V-1589"

$SPNE512566
$SPNE512566$`TUCA3-749`
cKO aKO bKO deltaKO alphaKO gammaKO
"GCA3-1436" "GCA3-1435" "GCA3-1434" "GCA3-1433" "GCA3-1432" "GCA3-1431"
betaKO epsilonKO
"GCA3-1429" "GCA3-1428"

$`LCAS1318635-WGS`
$`LCAS1318635-WGS`$`TUSOX-757`
epsilonKO betaKO gammaKO alphaKO deltaKO bKO
"GSOX-1334" "GSOX-1333" "GSOX-1332" "GSOX-1331" "GSOX-1330" "GSOX-1329"
cKO aKO
"GSOX-1328" "GSOX-1327"

$MTUB419947
$MTUB419947$`TUJ8N-704`
epsilonKO betaKO gammaKO alphaKO deltaKO bKO
"GJ8N-1360" "GJ8N-1359" "GJ8N-1358" "GJ8N-1357" "GJ8N-1356" "GJ8N-1355"
cKO aKO
"GJ8N-1354" "GJ8N-1353"

$MRAD426355
$MRAD426355$`TUJB5-838`
deltaKO alphaKO
"GJB5-610" "GJB5-609"

```
$MRAD426355$`TUJB5-837`
   gammaKO     betaKO  epsilonKO 
"GJB5-608" "GJB5-607" "GJB5-606" 

$MRAD426355$`TUJB5-911`
       aKO        cKO 
"GJB5-719" "GJB5-718" 

$MRAD426355$`TUJB5-910`
      bKO2       bKO1 
"GJB5-717" "GJB5-716" 


$MRHO710685
$MRHO710685$`TUI37-1779`
  epsilonKO      betaKO     gammaKO     alphaKO         bKO         cKO 
"GI37-3828" "GI37-3827" "GI37-3826" "GI37-3825" "GI37-3823" "GI37-3822" 
        aKO 
"GI37-3821" 

$MRHO710685$noTU
deltaKO 
     NA 


$ACIT397945
$ACIT397945$`TUI5W-221`
epsilonKO1     betaKO    gammaKO    alphaKO    deltaKO        bKO        cKO 
"GI5W-373" "GI5W-372" "GI5W-371" "GI5W-370" "GI5W-369" "GI5W-368" "GI5W-367" 
       aKO 
"GI5W-366" 

$ACIT397945$`TUI5W-975`
 epsilonKO2 
"GI5W-1819" 


$KVER204669
$KVER204669$`TUHL8-2479`
       bKO2        bKO1     deltaKO     alphaKO     gammaKO      betaKO 
"GHL8-4376" "GHL8-4375" "GHL8-4374" "GHL8-4373" "GHL8-4372" "GHL8-4371" 
  epsilonKO 
"GHL8-4370" 

$KVER204669$`TUHL8-740`
        cKO 
"GHL8-1312" 

$KVER204669$`TUHL8-739`
        aKO 
"GHL8-1311" 


$`ABAU1096996-WGS`
```

```
$`ABAU1096996-WGS`$`TUSEN-153`
 epsilonKO     betaKO    gammaKO    alphaKO    deltaKO        bKO        cKO
"GSEN-214" "GSEN-213" "GSEN-212" "GSEN-211" "GSEN-210" "GSEN-209" "GSEN-208"
       aKO
"GSEN-207"


$`ABAU945556-WGS`
$`ABAU945556-WGS`$`TUSEP-82`
 epsilonKO     betaKO    gammaKO    alphaKO    deltaKO        bKO        cKO
"GSEP-165" "GSEP-164" "GSEP-163" "GSEP-162" "GSEP-161" "GSEP-160" "GSEP-159"
       aKO
"GSEP-158"


$MROS1191523
$MROS1191523$`TULG8-101`
       aKO        cKO        bKO    deltaKO    alphaKO    gammaKO
"GLG8-288" "GLG8-287" "GLG8-286" "GLG8-285" "GLG8-284" "GLG8-283"

$MROS1191523$`TULG8-1003`
  epsilonKO      betaKO
"GLG8-2401" "GLG8-2400"


$MRUE886377
$MRUE886377$`TUI6V-733`
    gammaKO     alphaKO     deltaKO         bKO         cKO         aKO
"GI6V-1415" "GI6V-1413" "GI6V-1412" "GI6V-1411" "GI6V-1410" "GI6V-1409"

$MRUE886377$`TUI6V-565`
  epsilonKO      betaKO
"GI6V-1095" "GI6V-1094"


$SRUB761659
$SRUB761659$`TUHC6-911`
    alphaKO     deltaKO         bKO         cKO         aKO
"GHC6-1118" "GHC6-1117" "GHC6-1116" "GHC6-1115" "GHC6-1114"

$SRUB761659$`TUHC6-1981`
  epsilonKO      betaKO
"GHC6-2686" "GHC6-2685"

$SRUB761659$`TUHC6-912`
    gammaKO
"GHC6-1119"


$`MSME710686-WGS`
$`MSME710686-WGS`$`TUSRX-2106`
        aKO         cKO         bKO     alphaKO     gammaKO      betaKO
"GSRX-4717" "GSRX-4716" "GSRX-4715" "GSRX-4713" "GSRX-4712" "GSRX-4711"
  epsilonKO
```

```
"GSRX-4710"

$`MSME710686-WGS`$noTU
deltaKO
     NA


$AACI1048834
$AACI1048834$`TUL7E-1591`
       aKO         cKO         bKO     deltaKO     alphaKO     gammaKO
"GL7E-3088" "GL7E-3087" "GL7E-3086" "GL7E-3085" "GL7E-3084" "GL7E-3083"
     betaKO   epsilonKO
"GL7E-3082" "GL7E-3081"


$LCAR1229758
$LCAR1229758$`TULFN-143`
 epsilonKO     betaKO    gammaKO    alphaKO    deltaKO        bKO        cKO
"GLFN-145" "GLFN-144" "GLFN-143" "GLFN-142" "GLFN-141" "GLFN-140" "GLFN-139"
       aKO
"GLFN-138"


$MSP187303
$MSP187303$`TULGG-1855`
    deltaKO     alphaKO     gammaKO      betaKO   epsilonKO
"GLGG-3376" "GLGG-3375" "GLGG-3374" "GLGG-3373" "GLGG-3372"

$MSP187303$`TULGG-1684`
       bKO2        bKO1         cKO         aKO
"GLGG-3084" "GLGG-3083" "GLGG-3082" "GLGG-3081"


$`MSTI1278073-WGS`
$`MSTI1278073-WGS`$`TUSSE-4204`
    alphaKO
"GSSE-7620"

$`MSTI1278073-WGS`$`TUSSE-4140`
     betaKO   epsilonKO
"GSSE-7515" "GSSE-7513"

$`MSTI1278073-WGS`$`TUSSE-4142`
    gammaKO
"GSSE-7517"

$`MSTI1278073-WGS`$`TUSSE-4202`
    deltaKO
"GSSE-7618"

$`MSTI1278073-WGS`$`TUSSE-258`
       bKO        cKO        aKO
"GSSE-433" "GSSE-432" "GSSE-431"
```

$MSUI708248
$MSUI708248$`TUHCI-181`
aKO bKO deltaKO alphaKO gammaKO
"GHCI-465" "GHCI-463" "GHCI-462" "GHCI-461" "GHCI-460"

$MSUI708248$`TUHCI-178`
betaKO
"GHCI-457"

$MSUI708248$noTU
epsilonKO cKO
NA NA

$MSIL395965
$MSIL395965$`TUCND-218`
epsilonKO betaKO gammaKO alphaKO deltaKO
"GCND-350" "GCND-349" "GCND-348" "GCND-347" "GCND-346"

$MSIL395965$`TUCND-2237`
aKO cKO
"GCND-3877" "GCND-3876"

$MSIL395965$`TUCND-2236`
bKO2 bKO1
"GCND-3875" "GCND-3874"

$MSME246196
$MSME246196$`TUJ4Y-2227`
aKO cKO bKO deltaKO alphaKO gammaKO
"GJ4Y-4941" "GJ4Y-4940" "GJ4Y-4939" "GJ4Y-4938" "GJ4Y-4937" "GJ4Y-4936"
betaKO epsilonKO
"GJ4Y-4935" "GJ4Y-4934"

$MSP278137
$MSP278137$`TUHD6-945`
epsilonKO betaKO gammaKO alphaKO deltaKO bKO
"GHD6-1737" "GHD6-1736" "GHD6-1735" "GHD6-1734" "GHD6-1733" "GHD6-1732"
cKO aKO
"GHD6-1731" "GHD6-1730"

$MSUI768700
$MSUI768700$`TUI6L-192`
aKO bKO deltaKO alphaKO gammaKO
"GI6L-513" "GI6L-511" "GI6L-510" "GI6L-509" "GI6L-508"

$MSUI768700$`TUI6L-189`
betaKO
"GI6L-505"

```
$MSUI768700$noTU
epsilonKO       cKO
       NA        NA


$SELO269084
$SELO269084$`TUCDQ-724`
        aKO         cKO        bKO2        bKO1     deltaKO     alphaKO
"GCDQ-1209" "GCDQ-1208" "GCDQ-1207" "GCDQ-1206" "GCDQ-1205" "GCDQ-1204"
    gammaKO
"GCDQ-1203"

$SELO269084$`TUCDQ-1076`
     betaKO   epsilonKO
"GCDQ-1835" "GCDQ-1834"


$BMAN221988
$BMAN221988$`TUHGM-1225`
        aKO         cKO         bKO     deltaKO     alphaKO     gammaKO
"GHGM-2414" "GHGM-2413" "GHGM-2412" "GHGM-2411" "GHGM-2410" "GHGM-2409"
     betaKO   epsilonKO
"GHGM-2408" "GHGM-2407"


$MSYN262723
$MSYN262723$`TUH37-60`
   betaKO1   alphaKO1
"GH37-169" "GH37-168"

$MSYN262723$`TUH37-175`
       aKO        cKO        bKO    deltaKO   alphaKO2    gammaKO    betaKO2
"GH37-415" "GH37-414" "GH37-413" "GH37-412" "GH37-411" "GH37-410" "GH37-409"
 epsilonKO
"GH37-408"

$MSYN262723$`TUH37-201`
  alphaKO3    betaKO3
"GH37-475" "GH37-474"


$MTHE264732
$MTHE264732$`TUH0A-1373`
        aKO         cKO         bKO     deltaKO     alphaKO     gammaKO
"GH0A-2469" "GH0A-2468" "GH0A-2467" "GH0A-2466" "GH0A-2465" "GH0A-2464"
     betaKO   epsilonKO
"GH0A-2463" "GH0A-2462"


$LCRI748671
$LCRI748671$`TUIX1-433`
 epsilonKO     betaKO    gammaKO    alphaKO    deltaKO        bKO        cKO
"GIX1-835" "GIX1-834" "GIX1-833" "GIX1-832" "GIX1-831" "GIX1-830" "GIX1-829"
       aKO
```

```
"GIX1-828"


$MTUB478434
$MTUB478434$`TUH8E-1352`
    alphaKO     gammaKO      betaKO   epsilonKO
"GH8E-2766" "GH8E-2765" "GH8E-2764" "GH8E-2763"

$MTUB478434$`TUH8E-1353`
        aKO         cKO         bKO     deltaKO
"GH8E-2770" "GH8E-2769" "GH8E-2768" "GH8E-2767"


$MTBCDC1551
$MTBCDC1551$`TUT3Z-728`
  epsilonKO      betaKO     gammaKO     alphaKO     deltaKO         bKO
"GT3Z-5724" "GT3Z-5723" "GT3Z-5722" "GT3Z-5721" "GT3Z-5720" "GT3Z-5719"
        cKO         aKO
"GT3Z-5718" "GT3Z-5717"


$MTUB443149
$MTUB443149$`TULGT-694`
  epsilonKO      betaKO     gammaKO     alphaKO         bKO         aKO
"GLGT-1231" "GLGT-1230" "GLGT-1229" "GLGT-1228" "GLGT-1225" "GLGT-1224"

$MTUB443149$noTU
deltaKO     cKO
     NA      NA


$MTUB336982
$MTUB336982$`TUH7I-704`
  epsilonKO      betaKO     gammaKO     alphaKO     deltaKO         bKO
"GH7I-1356" "GH7I-1355" "GH7I-1354" "GH7I-1353" "GH7I-1352" "GH7I-1351"
        cKO         aKO
"GH7I-1350" "GH7I-1349"


$MTUB1091500
$MTUB1091500$`TULGW-733`
  epsilonKO      betaKO     gammaKO     alphaKO         bKO         cKO
"GLGW-1266" "GLGW-1265" "GLGW-1264" "GLGW-1263" "GLGW-1261" "GLGW-1260"
        aKO
"GLGW-1259"

$MTUB1091500$noTU
deltaKO
     NA


$`MTUB1306400-WGS`
$`MTUB1306400-WGS`$`TUSR7-736`
  epsilonKO      betaKO     gammaKO     alphaKO         bKO         cKO
```

```
"GSR7-1401" "GSR7-1400" "GSR7-1399" "GSR7-1398" "GSR7-1396" "GSR7-1395"
       aKO
"GSR7-1394"

$`MTUB1306400-WGS`$noTU
deltaKO
    NA



$SSP1148
$SSP1148$`TUJOT-369`
      aKO       cKO      bKO1      bKO2    deltaKO    alphaKO    gammaKO
"GJOT-164" "GJOT-163" "GJOT-162" "GJOT-161" "GJOT-160" "GJOT-159" "GJOT-158"

$SSP1148$`TUJOT-1337`
  epsilonKO      betaKO
"GJOT-1531" "GJOT-1530"



$MTUB478433
$MTUB478433$`TUH4Y-1351`
       aKO         cKO         bKO     alphaKO     gammaKO      betaKO
"GH4Y-2726" "GH4Y-2725" "GH4Y-2724" "GH4Y-2722" "GH4Y-2721" "GH4Y-2720"
  epsilonKO
"GH4Y-2719"

$MTUB478433$noTU
deltaKO
    NA



$MTUB443150
$MTUB443150$`TULGS-704`
  epsilonKO      betaKO     gammaKO     alphaKO         bKO         aKO
"GLGS-1222" "GLGS-1221" "GLGS-1220" "GLGS-1219" "GLGS-1217" "GLGS-1216"

$MTUB443150$noTU
deltaKO     cKO
    NA      NA



$`MTUB652616-WGS`
$`MTUB652616-WGS`$`TUSRC-797`
  epsilonKO      betaKO     gammaKO     alphaKO         bKO         cKO
"GSRC-1464" "GSRC-1463" "GSRC-1462" "GSRC-1461" "GSRC-1459" "GSRC-1458"
       aKO
"GSRC-1457"

$`MTUB652616-WGS`$noTU
deltaKO
    NA



$MTUB707235
```

```
$MTUB707235$`TULGU-694`
  epsilonKO      betaKO     gammaKO     alphaKO         bKO         cKO 
"GLGU-1344" "GLGU-1343" "GLGU-1342" "GLGU-1341" "GLGU-1339" "GLGU-1338" 
        aKO 
"GLGU-1337" 

$MTUB707235$noTU
deltaKO 
     NA 


$LCAS998820
$LCAS998820$`TULF4-796`
  epsilonKO      betaKO     gammaKO     alphaKO     deltaKO         bKO 
"GLF4-1418" "GLF4-1417" "GLF4-1416" "GLF4-1415" "GLF4-1414" "GLF4-1413" 
        cKO         aKO 
"GLF4-1412" "GLF4-1411" 


$MTRA643867
$MTRA643867$`TUI2X-1477`
    gammaKO     alphaKO     deltaKO         bKO         cKO         aKO 
"GI2X-2607" "GI2X-2606" "GI2X-2605" "GI2X-2604" "GI2X-2603" "GI2X-2602" 

$MTRA643867$`TUI2X-1518`
     betaKO   epsilonKO 
"GI2X-2684" "GI2X-2683" 


$MTBRV
$MTBRV$`TU1G-1812`
epsilonKO    betaKO   gammaKO   alphaKO   deltaKO       bKO       cKO       aKO 
 "RV1311"  "RV1310"  "RV1309"  "RV1308"  "RV1307"  "RV1306"  "RV1305"  "RV1304" 


$`MTUB1138877-WGS`
$`MTUB1138877-WGS`$`TUSRJ-708`
  epsilonKO      betaKO     gammaKO     alphaKO         bKO         cKO 
"GSRJ-1359" "GSRJ-1358" "GSRJ-1357" "GSRJ-1356" "GSRJ-1354" "GSRJ-1353" 
        aKO 
"GSRJ-1352" 

$`MTUB1138877-WGS`$noTU
deltaKO 
     NA 


$`MTUB1310114-WGS`
$`MTUB1310114-WGS`$`TUSR8-812`
  epsilonKO      betaKO     gammaKO     alphaKO         bKO         cKO 
"GSR8-1360" "GSR8-1359" "GSR8-1358" "GSR8-1357" "GSR8-1355" "GSR8-1354" 
        aKO 
"GSR8-1353" 
```

```
$`MTUB1310114-WGS`$noTU
deltaKO
     NA


$`MTUB1310115-WGS`
$`MTUB1310115-WGS`$`TUSRA-729`
  epsilonKO      betaKO     gammaKO     alphaKO         bKO         cKO
"GSRA-1378" "GSRA-1377" "GSRA-1376" "GSRA-1375" "GSRA-1373" "GSRA-1372"
        aKO
"GSRA-1371"

$`MTUB1310115-WGS`$noTU
deltaKO
     NA


$LPNE400673
$LPNE400673$`TUCIT-607`
    betaKO1  epsilonKO1        aKO1        cKO1        bKO1    alphaKO1
"GCIT-1180" "GCIT-1179" "GCIT-1177" "GCIT-1176" "GCIT-1175" "GCIT-1174"

$LPNE400673$`TUCIT-1741`
       aKO2        cKO2        bKO2     deltaKO    alphaKO2    gammaKO2
"GCIT-3240" "GCIT-3239" "GCIT-3238" "GCIT-3237" "GCIT-3236" "GCIT-3235"
    betaKO2  epsilonKO2
"GCIT-3234" "GCIT-3233"

$LPNE400673$`TUCIT-606`
   gammaKO1
"GCIT-1173"


$`MTUB395095-WGS`
$`MTUB395095-WGS`$`TUSRG-712`
  epsilonKO      betaKO     gammaKO     alphaKO         bKO         cKO
"GSRG-1389" "GSRG-1388" "GSRG-1387" "GSRG-1386" "GSRG-1384" "GSRG-1383"
        aKO
"GSRG-1382"

$`MTUB395095-WGS`$noTU
deltaKO
     NA


$CBES521460
$CBES521460$`TUH8H-672`
        aKO         cKO         bKO     deltaKO     alphaKO     gammaKO
"GH8H-1449" "GH8H-1448" "GH8H-1447" "GH8H-1446" "GH8H-1445" "GH8H-1444"
     betaKO   epsilonKO
"GH8H-1443" "GH8H-1442"


$AVAR240292
```

```
$AVAR240292$`TUCY3-2239`
       aKO         cKO        bKO2        bKO1     deltaKO     alphaKO 
"GCY3-2649" "GCY3-2648" "GCY3-2647" "GCY3-2646" "GCY3-2645" "GCY3-2644" 
    gammaKO 
"GCY3-2643" 

$AVAR240292$`TUCY3-2007`
     betaKO   epsilonKO 
"GCY3-2325" "GCY3-2324" 



$`MTUB1306414-WGS`
$`MTUB1306414-WGS`$`TUSRB-704`
  epsilonKO      betaKO     gammaKO     alphaKO         bKO         cKO 
"GSRB-1367" "GSRB-1366" "GSRB-1365" "GSRB-1364" "GSRB-1362" "GSRB-1361" 
        aKO 
"GSRB-1360" 

$`MTUB1306414-WGS`$noTU
deltaKO 
     NA 



$MTUB478435
$MTUB478435$`TULGX-1357`
        aKO         cKO         bKO     alphaKO     gammaKO      betaKO 
"GLGX-2743" "GLGX-2742" "GLGX-2741" "GLGX-2739" "GLGX-2738" "GLGX-2737" 
  epsilonKO 
"GLGX-2736" 

$MTUB478435$noTU
deltaKO 
     NA 



$`LCAS1215914-WGS`
$`LCAS1215914-WGS`$`TUSOY-807`
  epsilonKO      betaKO     gammaKO     alphaKO     deltaKO         bKO 
"GSOY-1432" "GSOY-1431" "GSOY-1430" "GSOY-1429" "GSOY-1428" "GSOY-1427" 
        cKO         aKO 
"GSOY-1426" "GSOY-1425" 



$MULC362242
$MULC362242$`TUCUY-9067`
        aKO         cKO         bKO     deltaKO     alphaKO     gammaKO 
"GCUY-4079" "GCUY-4078" "GCUY-4077" "GCUY-4076" "GCUY-4075" "GCUY-4074" 
     betaKO   epsilonKO 
"GCUY-4073" "GCUY-4072" 



$MVAN350058
$MVAN350058$`TUIWR-1981`
        aKO         cKO         bKO     deltaKO     alphaKO     gammaKO 
```

```
"GIWR-4373" "GIWR-4372" "GIWR-4371" "GIWR-4370" "GIWR-4369" "GIWR-4368"
     betaKO   epsilonKO
"GIWR-4367" "GIWR-4366"


$MWEN1197325
$MWEN1197325$`TULHL-48`
       aKO        bKO    deltaKO    alphaKO    gammaKO
"GLHL-104" "GLHL-103" "GLHL-102" "GLHL-101" "GLHL-100"

$MWEN1197325$`TULHL-45`
   betaKO
"GLHL-97"

$MWEN1197325$`TULHL-108`
       cKO
"GLHL-234"

$MWEN1197325$noTU
epsilonKO
       NA


$MXAN246197
$MXAN246197$`TUIWU-3632`
    alphaKO
"GIWU-6965"

$MXAN246197$`TUIWU-3581`
     betaKO   epsilonKO
"GIWU-6860" "GIWU-6858"

$MXAN246197$`TUIWU-3582`
    gammaKO
"GIWU-6862"

$MXAN246197$`TUIWU-3630`
    deltaKO
"GIWU-6963"

$MXAN246197$`TUIWU-212`
       bKO        cKO        aKO
"GIWU-399" "GIWU-398" "GIWU-397"


$SSP32049
$SSP32049$`TUKF7-711`
      bKO1    deltaKO   alphaKO1   gammaKO1
"GKF7-736" "GKF7-735" "GKF7-734" "GKF7-733"

$SSP32049$`TUKF7-101`
   gammaKO2    alphaKO2        bKO3        cKO2        aKO2
"GKF7-3027" "GKF7-3026" "GKF7-3025" "GKF7-3024" "GKF7-3023"
```

```
$SSP32049$`TUKF7-719`
epsilonKO1    betaKO1
"GKF7-750" "GKF7-749"

$SSP32049$`TUKF7-100`
 epsilonKO2     betaKO2
"GKF7-3020" "GKF7-3019"

$SSP32049$`TUKF7-713`
      aKO1       cKO1
"GKF7-739" "GKF7-738"

$SSP32049$`TUKF7-712`
      bKO2
"GKF7-737"


$`MYON1138871-WGS`
$`MYON1138871-WGS`$`TUSRM-746`
  epsilonKO      betaKO     gammaKO     alphaKO         bKO         cKO
"GSRM-1415" "GSRM-1414" "GSRM-1413" "GSRM-1412" "GSRM-1410" "GSRM-1409"
        aKO
"GSRM-1408"

$`MYON1138871-WGS`$noTU
deltaKO
     NA


$NALB1205910
$NALB1205910$`TULHY-1965`
  epsilonKO      betaKO     gammaKO     alphaKO     deltaKO         bKO
"GLHY-3211" "GLHY-3210" "GLHY-3209" "GLHY-3208" "GLHY-3207" "GLHY-3206"
        cKO         aKO
"GLHY-3205" "GLHY-3204"


$NPRO598659
$NPRO598659$`TUH7N-117`
 epsilonKO     betaKO    gammaKO    alphaKO    deltaKO       bKO2       bKO1
"GH7N-321" "GH7N-320" "GH7N-319" "GH7N-318" "GH7N-317" "GH7N-316" "GH7N-315"

$NPRO598659$`TUH7N-377`
        cKO
"GH7N-1028"

$NPRO598659$`TUH7N-534`
        aKO
"GH7N-1468"


$NARO279238
$NARO279238$`TUHBU-1453`
     betaKO     gammaKO     alphaKO
```

```
"GHBU-2444" "GHBU-2443" "GHBU-2442"

$NARO279238$`TUHBU-1452`
    deltaKO
"GHBU-2441"

$NARO279238$`TUHBU-1454`
  epsilonKO
"GHBU-2445"

$NARO279238$`TUHBU-927`
        cKO
"GHBU-1334"

$NARO279238$`TUHBU-926`
        aKO
"GHBU-1333"

$NARO279238$`TUHBU-928`
       bKO2        bKO1
"GHBU-1336" "GHBU-1335"


$TAZO551115
$TAZO551115$`TUH0O-2473`
    gammaKO     alphaKO     deltaKO        bKO2
"GH0O-3359" "GH0O-3358" "GH0O-3357" "GH0O-3356"

$TAZO551115$`TUH0O-1470`
     betaKO   epsilonKO
"GH0O-1948" "GH0O-1947"

$TAZO551115$`TUH0O-2471`
        cKO         aKO
"GH0O-3354" "GH0O-3353"

$TAZO551115$`TUH0O-2472`
       bKO1
"GH0O-3355"


$NBRA1133849
$NBRA1133849$`TULHW-732`
 epsilonKO1      betaKO     gammaKO     alphaKO     deltaKO        bKO1
"GLHW-1274" "GLHW-1273" "GLHW-1272" "GLHW-1271" "GLHW-1270" "GLHW-1269"
       cKO1        aKO1
"GLHW-1268" "GLHW-1267"

$NBRA1133849$`TULHW-1751`
 epsilonKO2
"GLHW-3134"

$NBRA1133849$`TULHW-3647`
       bKO2        cKO2        aKO2
```

"GLHW-6613" "GLHW-6612" "GLHW-6611"

$NBRA1133849$`TULHW-3714`
bKO3
"GLHW-6742"

$LCAS498216
$LCAS498216$`TUH2S-673`
gammaKO alphaKO deltaKO bKO cKO aKO
"GH2S-1151" "GH2S-1150" "GH2S-1149" "GH2S-1148" "GH2S-1147" "GH2S-1146"

$LCAS498216$`TUH2S-674`
epsilonKO betaKO
"GH2S-1153" "GH2S-1152"

$NSP196162
$NSP196162$`TUH4V-989`
epsilonKO betaKO gammaKO alphaKO deltaKO bKO
"GH4V-1791" "GH4V-1790" "GH4V-1789" "GH4V-1788" "GH4V-1787" "GH4V-1786"
cKO aKO
"GH4V-1785" "GH4V-1784"

$NCYR1127134
$NCYR1127134$`TULHX-631`
alphaKO
"GLHX-1138"

$NCYR1127134$`TULHX-633`
epsilonKO betaKO
"GLHX-1141" "GLHX-1140"

$NCYR1127134$`TULHX-632`
gammaKO
"GLHX-1139"

$NCYR1127134$`TULHX-630`
deltaKO bKO cKO aKO
"GLHX-1137" "GLHX-1136" "GLHX-1135" "GLHX-1134"

$NDAS446468
$NDAS446468$`TUHUM-650`
epsilonKO betaKO gammaKO alphaKO deltaKO bKO cKO
"GHUM-346" "GHUM-345" "GHUM-344" "GHUM-343" "GHUM-342" "GHUM-341" "GHUM-340"
aKO
"GHUM-339"

$SYNWH8102
$SYNWH8102$`TUIOJ-231`
gammaKO alphaKO deltaKO bKO2 bKO1 cKO

"GIOJ-3074" "GIOJ-3073" "GIOJ-3072" "GIOJ-3071" "GIOJ-3070" "GIOJ-3069"
aKO
"GIOJ-3068"

$SYNWH8102$`TUIOJ-235`
betaKO epsilonKO
"GIOJ-3091" "GIOJ-3090"

$NDEF330214
$NDEF330214$`TUI4U-191`
epsilonKO betaKO gammaKO alphaKO deltaKO
"GI4U-370" "GI4U-369" "GI4U-368" "GI4U-367" "GI4U-366"

$NDEF330214$`TUI4U-1986`
aKO cKO bKO
"GI4U-3666" "GI4U-3665" "GI4U-3664"

$NEUT335283
$NEUT335283$`TUHT6-206`
epsilonKO1 betaKO1 gammaKO1 alphaKO1 deltaKO bKO1 cKO1
"GHT6-287" "GHT6-286" "GHT6-285" "GHT6-284" "GHT6-283" "GHT6-282" "GHT6-281"
aKO1
"GHT6-280"

$NEUT335283$`TUHT6-1192`
gammaKO2 alphaKO2 bKO2 cKO2 aKO2 epsilonKO2
"GHT6-2059" "GHT6-2058" "GHT6-2057" "GHT6-2056" "GHT6-2055" "GHT6-2052"
betaKO2
"GHT6-2051"

$NEUR228410
$NEUR228410$`TUJNO-113`
epsilonKO betaKO gammaKO alphaKO deltaKO bKO cKO
"GJNO-215" "GJNO-214" "GJNO-213" "GJNO-212" "GJNO-211" "GJNO-210" "GJNO-209"
aKO
"GJNO-208"

$NFAR247156
$NFAR247156$`TUJ9T-651`
epsilonKO betaKO gammaKO alphaKO deltaKO bKO
"GJ9T-1091" "GJ9T-1090" "GJ9T-1089" "GJ9T-1088" "GJ9T-1087" "GJ9T-1086"
cKO aKO
"GJ9T-1085" "GJ9T-1084"

$NGON521006
$NGON521006$`TUJ73-1462`
epsilonKO betaKO gammaKO alphaKO deltaKO bKO
"GJ73-2700" "GJ73-2699" "GJ73-2698" "GJ73-2697" "GJ73-2696" "GJ73-2695"
cKO aKO

"GJ73-2694" "GJ73-2693"

$NGON242231
$NGON242231$`TUI2G-1189`
epsilonKO betaKO gammaKO alphaKO deltaKO bKO
"GI2G-2042" "GI2G-2041" "GI2G-2040" "GI2G-2039" "GI2G-2038" "GI2G-2037"
cKO aKO
"GI2G-2036" "GI2G-2035"

$NGON940296
$NGON940296$`TULHN-1298`
epsilonKO betaKO gammaKO alphaKO deltaKO bKO
"GLHN-2164" "GLHN-2163" "GLHN-2162" "GLHN-2161" "GLHN-2160" "GLHN-2159"
cKO aKO
"GLHN-2158" "GLHN-2157"

$LDEL390333
$LDEL390333$`TUIXG-376`
epsilonKO betaKO gammaKO alphaKO deltaKO bKO cKO
"GIXG-708" "GIXG-707" "GIXG-706" "GIXG-705" "GIXG-704" "GIXG-703" "GIXG-702"
aKO
"GIXG-701"

$NHAL472759
$NHAL472759$`TUHH2-1078`
aKO1 cKO1 bKO1 alphaKO1 gammaKO1
"GHH2-1932" "GHH2-1931" "GHH2-1930" "GHH2-1929" "GHH2-1928"

$NHAL472759$`TUHH2-1259`
betaKO2 epsilonKO2 aKO2 cKO2 bKO2 alphaKO2
"GHH2-2273" "GHH2-2272" "GHH2-2270" "GHH2-2269" "GHH2-2268" "GHH2-2267"
gammaKO2
"GHH2-2266"

$NHAL472759$`TUHH2-2158`
aKO3 cKO3 bKO3 deltaKO alphaKO3 gammaKO3
"GHH2-4007" "GHH2-4006" "GHH2-4005" "GHH2-4004" "GHH2-4003" "GHH2-4002"
betaKO3 epsilonKO3
"GHH2-4001" "GHH2-4000"

$NHAL472759$`TUHH2-1102`
betaKO1 epsilonKO1
"GHH2-1980" "GHH2-1979"

$NSP261292
$NSP261292$`TUH7H-18`
gammaKO1 alphaKO1 bKO1 cKO1 aKO1 epsilonKO1 betaKO1
"GH7H-46" "GH7H-45" "GH7H-44" "GH7H-43" "GH7H-42" "GH7H-39" "GH7H-38"

$NSP261292$`TUH7H-228`
epsilonKO2 betaKO2 gammaKO2 alphaKO2 deltaKO bKO2 cKO2
"GH7H-378" "GH7H-377" "GH7H-376" "GH7H-375" "GH7H-374" "GH7H-373" "GH7H-372"
aKO2
"GH7H-371"

$HMUS679897
$HMUS679897$`TUJBK-174`
epsilonKO betaKO gammaKO alphaKO deltaKO bKO2 bKO1
"GJBK-457" "GJBK-456" "GJBK-455" "GJBK-454" "GJBK-453" "GJBK-452" "GJBK-451"

$HMUS679897$`TUJBK-305`
cKO
"GJBK-772"

$HMUS679897$`TUJBK-406`
aKO
"GJBK-992"

$`TELO197221-WGS`
$`TELO197221-WGS`$`TUSYB-228`
alphaKO deltaKO bKO2 bKO1 cKO aKO
"GSYB-448" "GSYB-447" "GSYB-446" "GSYB-445" "GSYB-444" "GSYB-443"

$`TELO197221-WGS`$`TUSYB-282`
epsilonKO betaKO
"GSYB-539" "GSYB-538"

$`TELO197221-WGS`$`TUSYB-197`
gammaKO
"GSYB-397"

$NSP387092
$NSP387092$`TUHA5-401`
bKO2 bKO1 deltaKO alphaKO gammaKO betaKO
"GHA5-1264" "GHA5-1263" "GHA5-1262" "GHA5-1261" "GHA5-1260" "GHA5-1259"
epsilonKO
"GHA5-1258"

$NSP387092$`TUHA5-298`
aKO
"GHA5-892"

$NSP387092$noTU
cKO
NA

$NSP153948
$NSP153948$`TUHZ8-265`
epsilonKO betaKO gammaKO alphaKO deltaKO bKO cKO

"GHZ8-350" "GHZ8-349" "GHZ8-348" "GHZ8-347" "GHZ8-346" "GHZ8-345" "GHZ8-344"
aKO
"GHZ8-343"

$NKOR700598
$NKOR700598$`TUHC3-4224`
alphaKO deltaKO bKO cKO aKO
"GHC3-6985" "GHC3-6984" "GHC3-6983" "GHC3-6982" "GHC3-6981"

$NKOR700598$`TUHC3-3372`
betaKO epsilonKO
"GHC3-5574" "GHC3-5573"

$NKOR700598$`TUHC3-3218`
gammaKO
"GHC3-5331"

$NLAC489653
$NLAC489653$`TUJ91-1155`
aKO cKO bKO deltaKO alphaKO gammaKO
"GJ91-1909" "GJ91-1908" "GJ91-1907" "GJ91-1906" "GJ91-1905" "GJ91-1904"
betaKO epsilonKO
"GJ91-1903" "GJ91-1902"

$NMEN122587
$NMEN122587$`TUI3Q-276`
epsilonKO betaKO gammaKO alphaKO deltaKO bKO cKO
"GI3Q-492" "GI3Q-491" "GI3Q-490" "GI3Q-489" "GI3Q-488" "GI3Q-487" "GI3Q-486"
aKO
"GI3Q-485"

$NMEN272831
$NMEN272831$`TUJDX-1068`
aKO cKO bKO deltaKO alphaKO gammaKO
"GJDX-1808" "GJDX-1807" "GJDX-1806" "GJDX-1805" "GJDX-1804" "GJDX-1803"
betaKO epsilonKO
"GJDX-1802" "GJDX-1801"

$`NMEN935599-WGS`
$`NMEN935599-WGS`$`TUSS5-1144`
aKO cKO bKO deltaKO alphaKO gammaKO
"GSS5-1866" "GSS5-1865" "GSS5-1864" "GSS5-1863" "GSS5-1862" "GSS5-1861"
betaKO epsilonKO
"GSS5-1860" "GSS5-1859"

$NMEN122586
$NMEN122586$`TUHGG-1192`
aKO cKO bKO deltaKO alphaKO gammaKO

```
"GHGG-1997" "GHGG-1996" "GHGG-1995" "GHGG-1994" "GHGG-1993" "GHGG-1992"
     betaKO   epsilonKO 
"GHGG-1991" "GHGG-1990" 


$LDEL767455
$LDEL767455$`TUHXE-364`
    betaKO    gammaKO    alphaKO    deltaKO        bKO        cKO        aKO 
"GHXE-661" "GHXE-660" "GHXE-659" "GHXE-658" "GHXE-657" "GHXE-656" "GHXE-655" 

$LDEL767455$`TUHXE-365`
 epsilonKO 
"GHXE-662" 


$NMEN909420
$NMEN909420$`TULHQ-1141`
        aKO         cKO         bKO     deltaKO     alphaKO     gammaKO 
"GLHQ-1879" "GLHQ-1878" "GLHQ-1877" "GLHQ-1876" "GLHQ-1875" "GLHQ-1874" 
     betaKO   epsilonKO 
"GLHQ-1873" "GLHQ-1872" 


$TMOB1110502
$TMOB1110502$`TULMJ-2181`
   gammaKO1    alphaKO1        bKO1        cKO1        aKO1  epsilonKO1 
"GLMJ-1674" "GLMJ-1673" "GLMJ-1672" "GLMJ-1671" "GLMJ-1670" "GLMJ-1667" 
    betaKO1 
"GLMJ-1666" 

$TMOB1110502$`TULMJ-2912`
    deltaKO    alphaKO2 
"GLMJ-3027" "GLMJ-3026" 

$TMOB1110502$`TULMJ-2910`
    betaKO2 
"GLMJ-3024" 

$TMOB1110502$`TULMJ-2911`
   gammaKO2 
"GLMJ-3025" 

$TMOB1110502$`TULMJ-2909`
 epsilonKO2 
"GLMJ-3023" 

$TMOB1110502$`TULMJ-2800`
       cKO2 
"GLMJ-2818" 

$TMOB1110502$`TULMJ-2799`
       aKO2 
"GLMJ-2817" 
```

```
$TMOB1110502$`TULMJ-2801`
      bKO3        bKO2 
"GLMJ-2820" "GLMJ-2819" 


$NMEN662598
$NMEN662598$`TUJMX-147`
 epsilonKO     betaKO    gammaKO    alphaKO    deltaKO        bKO        cKO 
"GJMX-242" "GJMX-241" "GJMX-240" "GJMX-239" "GJMX-238" "GJMX-237" "GJMX-236" 
      aKO 
"GJMX-235" 


$NMUL479431
$NMUL479431$`TUHQL-1149`
    betaKO     gammaKO     alphaKO     deltaKO         bKO         cKO 
"GHQL-2156" "GHQL-2155" "GHQL-2154" "GHQL-2153" "GHQL-2152" "GHQL-2151" 

$NMUL479431$`TUHQL-219`
 epsilonKO 
"GHQL-385" 

$NMUL479431$`TUHQL-1148`
       aKO 
"GHQL-2149" 


$NMEN935591
$NMEN935591$`TULHR-154`
 epsilonKO     betaKO    gammaKO    alphaKO    deltaKO        bKO        cKO 
"GLHR-255" "GLHR-254" "GLHR-253" "GLHR-252" "GLHR-251" "GLHR-250" "GLHR-249" 
      aKO 
"GLHR-248" 


$NMEN374833
$NMEN374833$`TUJ7Z-165`
 epsilonKO     betaKO    gammaKO    alphaKO    deltaKO        bKO        cKO 
"GJ7Z-283" "GJ7Z-282" "GJ7Z-281" "GJ7Z-280" "GJ7Z-279" "GJ7Z-278" "GJ7Z-277" 
      aKO 
"GJ7Z-276" 


$NMEN630588
$NMEN630588$`TULHP-1148`
       aKO         cKO         bKO     deltaKO     alphaKO     gammaKO 
"GLHP-1915" "GLHP-1914" "GLHP-1913" "GLHP-1912" "GLHP-1911" "GLHP-1910" 
    betaKO   epsilonKO 
"GLHP-1909" "GLHP-1908" 


$`AVIN1283331-WGS`
$`AVIN1283331-WGS`$`TUSFK-1036`
   gammaKO1    alphaKO1        bKO1        cKO1        aKO1  epsilonKO1 
```

```
"GSFK-1981" "GSFK-1980" "GSFK-1979" "GSFK-1978" "GSFK-1977" "GSFK-1974"
   betaKO1
"GSFK-1973"

$`AVIN1283331-WGS`$`TUSFK-2740`
      bKO2     deltaKO    alphaKO2    gammaKO2     betaKO2  epsilonKO2
"GSFK-5145" "GSFK-5144" "GSFK-5143" "GSFK-5142" "GSFK-5141" "GSFK-5140"

$`AVIN1283331-WGS`$`TUSFK-2741`
      aKO2        cKO2
"GSFK-5147" "GSFK-5146"


$`AVIN1283330-WGS`
$`AVIN1283330-WGS`$`TUSFL-1036`
   gammaKO1    alphaKO1        bKO1        cKO1        aKO1  epsilonKO1
"GSFL-1981" "GSFL-1980" "GSFL-1979" "GSFL-1978" "GSFL-1977" "GSFL-1974"
   betaKO1
"GSFL-1973"

$`AVIN1283330-WGS`$`TUSFL-2760`
      bKO2     deltaKO    alphaKO2    gammaKO2     betaKO2  epsilonKO2
"GSFL-5186" "GSFL-5185" "GSFL-5184" "GSFL-5183" "GSFL-5182" "GSFL-5181"

$`AVIN1283330-WGS`$`TUSFL-2761`
      aKO2        cKO2
"GSFL-5188" "GSFL-5187"


$AVIN322710
$AVIN322710$`TUJ0M-1043`
   gammaKO1    alphaKO1        bKO1        cKO1        aKO1  epsilonKO1
"GJ0M-1985" "GJ0M-1984" "GJ0M-1983" "GJ0M-1982" "GJ0M-1981" "GJ0M-1978"
   betaKO1
"GJ0M-1977"

$AVIN322710$`TUJ0M-2779`
      bKO2     deltaKO    alphaKO2    gammaKO2     betaKO2  epsilonKO2
"GJ0M-5197" "GJ0M-5196" "GJ0M-5195" "GJ0M-5194" "GJ0M-5193" "GJ0M-5192"

$AVIN322710$`TUJ0M-2780`
      aKO2        cKO2
"GJ0M-5199" "GJ0M-5198"


$NMEN935593
$NMEN935593$`TULHT-1125`
       aKO         cKO         bKO     deltaKO     alphaKO     gammaKO
"GLHT-1876" "GLHT-1875" "GLHT-1874" "GLHT-1873" "GLHT-1872" "GLHT-1871"
    betaKO   epsilonKO
"GLHT-1870" "GLHT-1869"


$LDEL353496
```

```
$LDEL353496$`TULF6-342`
 epsilonKO     betaKO    gammaKO    alphaKO    deltaKO        bKO        cKO
"GLF6-624" "GLF6-623" "GLF6-622" "GLF6-621" "GLF6-620" "GLF6-619" "GLF6-618"
       aKO
"GLF6-617"


$VHAR338187
$VHAR338187$`TUJCH-292`
      bKO1    deltaKO   alphaKO1   gammaKO1    betaKO1 epsilonKO1
"GJCH-418" "GJCH-417" "GJCH-416" "GJCH-415" "GJCH-414" "GJCH-413"

$VHAR338187$`TUJCH-2913`
       aKO2        cKO2        bKO2    alphaKO2    gammaKO2     betaKO2
"GJCH-5095" "GJCH-5094" "GJCH-5093" "GJCH-5091" "GJCH-5090" "GJCH-5089"
 epsilonKO2
"GJCH-5088"

$VHAR338187$`TUJCH-293`
      aKO1       cKO1
"GJCH-421" "GJCH-420"


$NMEN935588
$NMEN935588$`TULHS-1159`
        aKO         cKO         bKO     deltaKO     alphaKO     gammaKO
"GLHS-1869" "GLHS-1868" "GLHS-1867" "GLHS-1866" "GLHS-1865" "GLHS-1864"
     betaKO   epsilonKO
"GLHS-1863" "GLHS-1862"


$`NMEN604162-WGS`
$`NMEN604162-WGS`$`TUSS4-1191`
        aKO         cKO         bKO     deltaKO     alphaKO     gammaKO
"GSS4-1970" "GSS4-1969" "GSS4-1968" "GSS4-1967" "GSS4-1966" "GSS4-1965"
     betaKO   epsilonKO
"GSS4-1964" "GSS4-1963"


$NMUL323848
$NMUL323848$`TUKEC-210`
epsilonKO1    betaKO1   gammaKO1   alphaKO1    deltaKO       bKO1       cKO1
"GKEC-321" "GKEC-320" "GKEC-319" "GKEC-318" "GKEC-317" "GKEC-316" "GKEC-315"
      aKO1
"GKEC-314"

$NMUL323848$`TUKEC-1004`
   alphaKO2    gammaKO2
"GKEC-1684" "GKEC-1683"

$NMUL323848$`TUKEC-1005`
    betaKO2  epsilonKO2        aKO2        cKO2        bKO2
"GKEC-1691" "GKEC-1690" "GKEC-1687" "GKEC-1686" "GKEC-1685"
```

```
$NMEN942513
$NMEN942513$`TULHV-149`
 epsilonKO     betaKO    gammaKO    alphaKO    deltaKO        bKO        cKO
"GLHV-253" "GLHV-252" "GLHV-251" "GLHV-250" "GLHV-249" "GLHV-248" "GLHV-247"
       aKO
"GLHV-246"



$NMEN935589
$NMEN935589$`TULHU-226`
 epsilonKO     betaKO    gammaKO    alphaKO    deltaKO        bKO        cKO
"GLHU-392" "GLHU-391" "GLHU-390" "GLHU-389" "GLHU-388" "GLHU-387" "GLHU-386"
       aKO
"GLHU-385"



$NOCE323261
$NOCE323261$`TUCI3-1758`
        aKO         cKO         bKO     deltaKO     alphaKO     gammaKO
"GCI3-3133" "GCI3-3132" "GCI3-3131" "GCI3-3130" "GCI3-3129" "GCI3-3128"
     betaKO   epsilonKO
"GCI3-3127" "GCI3-3126"



$NSP28072
$NSP28072$`TULI0-3713`
       bKO1     deltaKO     alphaKO     gammaKO
"GLI0-5308" "GLI0-5307" "GLI0-5306" "GLI0-5305"

$NSP28072$`TULI0-3615`
     betaKO   epsilonKO
"GLI0-5158" "GLI0-5157"

$NSP28072$`TULI0-3715`
        cKO
"GLI0-5310"

$NSP28072$`TULI0-3716`
        aKO
"GLI0-5311"

$NSP28072$`TULI0-3714`
       bKO2
"GLI0-5309"



$NSP317936
$NSP317936$`TULHZ-1313`
    gammaKO     alphaKO     deltaKO       bKO2
"GLHZ-1918" "GLHZ-1917" "GLHZ-1916" "GLHZ-1915"

$NSP317936$`TULHZ-3131`
     betaKO   epsilonKO
```

```
"GLHZ-4536" "GLHZ-4535"

$NSP317936$`TULHZ-1311`
      cKO       aKO
"GLHZ-1913" "GLHZ-1912"

$NSP317936$`TULHZ-1312`
     bKO1
"GLHZ-1914"


$NSP702113
$NSP702113$`TUJD2-2092`
  epsilonKO     betaKO1    gammaKO1    alphaKO1     deltaKO
"GJD2-2640" "GJD2-2639" "GJD2-2638" "GJD2-2637" "GJD2-2636"

$NSP702113$`TUJD2-407`
    betaKO2        aKO2        cKO2        bKO3    alphaKO2    gammaKO2
"GJD2-4031" "GJD2-4030" "GJD2-4029" "GJD2-4028" "GJD2-4027" "GJD2-4026"

$NSP702113$`TUJD2-1400`
       bKO2        bKO1        cKO1        aKO1
"GJD2-1328" "GJD2-1327" "GJD2-1326" "GJD2-1325"


$NPUN63737
$NPUN63737$`TUJNP-3755`
    alphaKO
"GJNP-4736"

$NPUN63737$`TUJNP-3445`
     betaKO
"GJNP-4287"

$NPUN63737$`TUJNP-3756`
    gammaKO
"GJNP-4737"

$NPUN63737$`TUJNP-3754`
    deltaKO        bKO2
"GJNP-4735" "GJNP-4734"

$NPUN63737$`TUJNP-3444`
  epsilonKO
"GJNP-4286"

$NPUN63737$`TUJNP-3752`
        cKO         aKO
"GJNP-4732" "GJNP-4731"

$NPUN63737$`TUJNP-3753`
       bKO1
"GJNP-4733"
```

```
$`VPAR1338034-WGS`
$`VPAR1338034-WGS`$`TUSYU-2118`
 epsilonKO     betaKO    gammaKO    alphaKO    deltaKO        bKO        cKO
"GSYU-389" "GSYU-388" "GSYU-387" "GSYU-386" "GSYU-385" "GSYU-384" "GSYU-383"
       aKO
"GSYU-382"



$LSP979982
$LSP979982$`TUHM6-632`
        aKO         cKO         bKO     deltaKO     alphaKO     gammaKO
"GHM6-1194" "GHM6-1193" "GHM6-1192" "GHM6-1191" "GHM6-1190" "GHM6-1189"
     betaKO   epsilonKO
"GHM6-1188" "GHM6-1187"



$NRIS434131
$NRIS434131$`TUJCC-94`
   deltaKO    alphaKO
"GJCC-135" "GJCC-134"

$NRIS434131$`TUJCC-431`
    betaKO  epsilonKO
"GJCC-736" "GJCC-735"

$NRIS434131$`TUJCC-330`
   gammaKO
"GJCC-565"

$NRIS434131$`TUJCC-222`
      bKO2       bKO1        cKO        aKO
"GJCC-382" "GJCC-381" "GJCC-380" "GJCC-379"



$NSAL749222
$NSAL749222$`TUHWN-209`
 epsilonKO     betaKO    gammaKO    alphaKO    deltaKO       bKO2       bKO1
"GHWN-462" "GHWN-461" "GHWN-460" "GHWN-459" "GHWN-458" "GHWN-457" "GHWN-456"

$NSAL749222$`TUHWN-215`
       cKO
"GHWN-477"

$NSAL749222$`TUHWN-341`
       aKO
"GHWN-725"



$NSEN222891
$NSEN222891$`TUHFU-83`
   deltaKO    alphaKO
"GHFU-133" "GHFU-132"
```

```
$NSEN222891$`TUHFU-417`
    betaKO  epsilonKO 
"GHFU-766" "GHFU-765" 

$NSEN222891$`TUHFU-329`
   gammaKO 
"GHFU-590" 

$NSEN222891$`TUHFU-214`
      bKO2       bKO1        cKO        aKO 
"GHFU-400" "GHFU-399" "GHFU-398" "GHFU-397" 


$NTHE457570
$NTHE457570$`TUHRL-1670`
        aKO         cKO         bKO     deltaKO     alphaKO     gammaKO 
"GHRL-2912" "GHRL-2910" "GHRL-2909" "GHRL-2908" "GHRL-2907" "GHRL-2906" 
     betaKO   epsilonKO 
"GHRL-2905" "GHRL-2904" 


$NWAT105559
$NWAT105559$`TUHXU-1708`
        aKO         cKO         bKO     deltaKO     alphaKO     gammaKO 
"GHXU-3186" "GHXU-3185" "GHXU-3184" "GHXU-3183" "GHXU-3182" "GHXU-3181" 
     betaKO   epsilonKO 
"GHXU-3180" "GHXU-3179" 


$NWIN323098
$NWIN323098$`TUJEG-275`
 epsilonKO     betaKO    gammaKO    alphaKO    deltaKO 
"GJEG-441" "GJEG-440" "GJEG-438" "GJEG-437" "GJEG-436" 

$NWIN323098$`TUJEG-151`
       aKO        cKO 
"GJEG-242" "GJEG-241" 

$NWIN323098$`TUJEG-150`
      bKO2       bKO1 
"GJEG-240" "GJEG-239" 


$OACU56110
$OACU56110$`TULI3-895`
       bKO1     deltaKO     alphaKO     gammaKO 
"GLI3-1192" "GLI3-1191" "GLI3-1190" "GLI3-1189" 

$OACU56110$`TULI3-2757`
  epsilonKO      betaKO 
"GLI3-3767" "GLI3-3766" 

$OACU56110$`TULI3-896`
        cKO        bKO2 
```

```
"GLI3-1194" "GLI3-1193"

$OACU56110$`TULI3-897`
       aKO
"GLI3-1196"



$OANT439375
$OANT439375$`TUJIT-871`
  epsilonKO      betaKO     gammaKO     alphaKO     deltaKO
"GJIT-1122" "GJIT-1121" "GJIT-1120" "GJIT-1119" "GJIT-1118"

$OANT439375$`TUJIT-515`
      bKO2       bKO1        cKO       aKO1
"GJIT-509" "GJIT-508" "GJIT-507" "GJIT-506"

$OANT439375$`TUJIT-1690`
       aKO2
"GJIT-2561"



$`OANT391626-WGS`
$`OANT391626-WGS`$`TUSS7-431`
epsilonKO1     betaKO    gammaKO    alphaKO    deltaKO
"GSS7-835" "GSS7-834" "GSS7-833" "GSS7-832" "GSS7-831"

$`OANT391626-WGS`$`TUSS7-2289`
 epsilonKO2
"GSS7-4235"

$`OANT391626-WGS`$`TUSS7-335`
      bKO2       bKO1        cKO        aKO
"GSS7-647" "GSS7-646" "GSS7-645" "GSS7-644"



$VSPL575788
$VSPL575788$`TUH64-912`
       bKO1    deltaKO1    alphaKO1
"GH64-3081" "GH64-3080" "GH64-3079"

$VSPL575788$`TUH64-570`
   alphaKO2    deltaKO2        bKO2
"GH64-3992" "GH64-3991" "GH64-3990"

$VSPL575788$`TUH64-911`
   gammaKO1     betaKO1  epsilonKO1
"GH64-3078" "GH64-3077" "GH64-3076"

$VSPL575788$`TUH64-572`
 epsilonKO2     betaKO2
"GH64-3995" "GH64-3994"

$VSPL575788$`TUH64-571`
   gammaKO2
```

"GH64-3993"

$VSPL575788$`TUH64-913`
cKO1
"GH64-3082"

$VSPL575788$`TUH64-569`
cKO2
"GH64-3989"

$VSPL575788$`TUH64-914`
aKO1
"GH64-3083"

$VSPL575788$`TUH64-568`
aKO2
"GH64-3988"

$OSP511062
$OSP511062$`TUI6E-220`
epsilonKO betaKO gammaKO alphaKO deltaKO bKO cKO
"GI6E-462" "GI6E-461" "GI6E-460" "GI6E-459" "GI6E-458" "GI6E-457" "GI6E-456"
aKO
"GI6E-455"

$LSP111781
$LSP111781$`TULFJ-2961`
bKO1 deltaKO alphaKO gammaKO
"GLFJ-4383" "GLFJ-4382" "GLFJ-4381" "GLFJ-4380"

$LSP111781$`TULFJ-2723`
betaKO epsilonKO
"GLFJ-4021" "GLFJ-4020"

$LSP111781$`TULFJ-2963`
aKO cKO
"GLFJ-4386" "GLFJ-4385"

$LSP111781$`TULFJ-2962`
bKO2
"GLFJ-4384"

$OCAR504832
$OCAR504832$`TUJPZ-2036`
deltaKO alphaKO gammaKO betaKO epsilonKO
"GJPZ-3351" "GJPZ-3350" "GJPZ-3349" "GJPZ-3347" "GJPZ-3346"

$OCAR504832$`TUJPZ-1984`
bKO2 bKO1 cKO aKO
"GJPZ-3251" "GJPZ-3250" "GJPZ-3249" "GJPZ-3248"

```
$OCAR1031710
$OCAR1031710$`TULI1-2016`
   deltaKO     alphaKO     gammaKO      betaKO   epsilonKO
"GLI1-3299" "GLI1-3298" "GLI1-3297" "GLI1-3295" "GLI1-3294"

$OCAR1031710$`TULI1-1965`
      bKO2        bKO1         cKO         aKO
"GLI1-3199" "GLI1-3198" "GLI1-3197" "GLI1-3196"


$OHON926562
$OHON926562$`TUHWV-1247`
   gammaKO     alphaKO     deltaKO         bKO         cKO         aKO
"GHWV-2373" "GHWV-2372" "GHWV-2371" "GHWV-2370" "GHWV-2369" "GHWV-2368"

$OHON926562$`TUHWV-1535`
    betaKO
"GHWV-2861"

$OHON926562$`TUHWV-1536`
 epsilonKO
"GHWV-2862"


$OIHE221109
$OIHE221109$`TUI2A-1688`
       aKO         cKO         bKO     deltaKO     alphaKO     gammaKO
"GI2A-3071" "GI2A-3070" "GI2A-3069" "GI2A-3068" "GI2A-3067" "GI2A-3066"
    betaKO   epsilonKO
"GI2A-3065" "GI2A-3064"


$OULI633147
$OULI633147$`TUHMD-84`
 epsilonKO     betaKO    gammaKO    alphaKO    deltaKO       bKO       cKO
"GHMD-144" "GHMD-143" "GHMD-142" "GHMD-141" "GHMD-140" "GHMD-139" "GHMD-138"
      aKO
"GHMD-137"


$ONIG179408
$ONIG179408$`TULI4-1751`
   gammaKO     alphaKO     deltaKO        bKO2        bKO1         cKO
"GLI4-1869" "GLI4-1868" "GLI4-1867" "GLI4-1866" "GLI4-1865" "GLI4-1864"
       aKO
"GLI4-1863"

$ONIG179408$`TULI4-1565`
    betaKO   epsilonKO
"GLI4-1589" "GLI4-1588"


$OOEN203123
```

```
$OOEN203123$`TUHNL-332`
 epsilonKO     betaKO    gammaKO    alphaKO    deltaKO        bKO        cKO
"GHNL-666" "GHNL-665" "GHNL-664" "GHNL-663" "GHNL-662" "GHNL-661" "GHNL-660"
       aKO
"GHNL-659"


$ORHI867902
$ORHI867902$`TULI2-224`
       aKO        cKO        bKO    deltaKO    alphaKO    gammaKO
"GLI2-423" "GLI2-422" "GLI2-420" "GLI2-419" "GLI2-418" "GLI2-417"

$ORHI867902$`TULI2-774`
  epsilonKO      betaKO
"GLI2-1605" "GLI2-1604"


$BHYO565034
$BHYO565034$`TUJI7-1385`
    alphaKO    deltaKO2    deltaKO1         bKO         cKO         aKO
"GJI7-2180" "GJI7-2179" "GJI7-2178" "GJI7-2177" "GJI7-2176" "GJI7-2175"

$BHYO565034$`TUJI7-151`
    betaKO
"GJI7-203"

$BHYO565034$`TUJI7-1386`
    gammaKO
"GJI7-2181"

$BHYO565034$`TUJI7-777`
  epsilonKO
"GJI7-1205"


$OSPL709991
$OSPL709991$`TUI68-794`
  epsilonKO         aKO         cKO         bKO     deltaKO     alphaKO
"GI68-1515" "GI68-1514" "GI68-1513" "GI68-1512" "GI68-1511" "GI68-1510"
    gammaKO
"GI68-1509"

$OSPL709991$`TUI68-795`
     betaKO
"GI68-1516"


$OTER452637
$OTER452637$`TUHBR-579`
       aKO        cKO        bKO    deltaKO    alphaKO    gammaKO     betaKO
"GHBR-890" "GHBR-889" "GHBR-888" "GHBR-887" "GHBR-886" "GHBR-885" "GHBR-884"
 epsilonKO
"GHBR-883"
```

$AAEO224324
$AAEO224324$`TUJBH-705`
alphaKO
"GJBH-491"

$AAEO224324$`TUJBH-559`
gammaKO2 betaKO
"GJBH-1459" "GJBH-1458"

$AAEO224324$`TUJBH-78`
gammaKO1
"GJBH-156"

$AAEO224324$`TUJBH-425`
deltaKO bKO2 bKO1
"GJBH-1134" "GJBH-1133" "GJBH-1132"

$AAEO224324$`TUJBH-195`
epsilonKO
"GJBH-487"

$AAEO224324$`TUJBH-73`
aKO cKO
"GJBH-141" "GJBH-139"

$LFER1162668
$LFER1162668$`TULFM-34`
epsilonKO betaKO gammaKO alphaKO deltaKO bKO cKO aKO
"GLFM-69" "GLFM-68" "GLFM-67" "GLFM-66" "GLFM-65" "GLFM-64" "GLFM-63" "GLFM-62"

$OTSU357244
$OTSU357244$`TUCA5-337`
alphaKO gammaKO
"GCA5-597" "GCA5-596"

$OTSU357244$`TUCA5-429`
betaKO epsilonKO
"GCA5-758" "GCA5-757"

$OTSU357244$`TUCA5-338`
deltaKO
"GCA5-598"

$OTSU357244$`TUCA5-452`
bKO cKO aKO
"GCA5-793" "GCA5-792" "GCA5-791"

$OTSU334380
$OTSU334380$`TUC7O-605`
gammaKO alphaKO deltaKO

"GC7O-987" "GC7O-986" "GC7O-985"

$OTSU334380$`TUC7O-370`
betaKO epsilonKO
"GC7O-598" "GC7O-597"

$OTSU334380$`TUC7O-251`
aKO cKO bKO2 bKO1
"GC7O-394" "GC7O-393" "GC7O-392" "GC7O-391"

$PAES290512
$PAES290512$`TUHUT-195`
gammaKO1 alphaKO1
"GHUT-271" "GHUT-270"

$PAES290512$`TUHUT-559`
gammaKO2 alphaKO2 bKO1 cKO1 aKO1 epsilonKO2 betaKO2
"GHUT-931" "GHUT-930" "GHUT-929" "GHUT-928" "GHUT-927" "GHUT-924" "GHUT-923"

$PAES290512$`TUHUT-63`
betaKO1 epsilonKO1
"GHUT-50" "GHUT-49"

$PAES290512$`TUHUT-1324`
aKO2 cKO2 bKO2 deltaKO
"GHUT-2297" "GHUT-2296" "GHUT-2295" "GHUT-2294"

$PACN267747
$PACN267747$`TUHO9-638`
aKO cKO bKO deltaKO alphaKO gammaKO
"GHO9-1259" "GHO9-1258" "GHO9-1257" "GHO9-1256" "GHO9-1255" "GHO9-1254"
betaKO epsilonKO
"GHO9-1253" "GHO9-1252"

$`PACN1234380-WGS`
$`PACN1234380-WGS`$`TUSSM-661`
aKO cKO bKO deltaKO alphaKO gammaKO
"GSSM-1296" "GSSM-1295" "GSSM-1294" "GSSM-1293" "GSSM-1292" "GSSM-1291"
betaKO epsilonKO
"GSSM-1290" "GSSM-1289"

$`PACN1134454-WGS`
$`PACN1134454-WGS`$`TUST1-524`
epsilonKO betaKO gammaKO alphaKO deltaKO bKO cKO
"GST1-938" "GST1-937" "GST1-936" "GST1-935" "GST1-934" "GST1-933" "GST1-932"
aKO
"GST1-931"

$BPSE320373

```
$BPSE320373$`TUJ9C-2093`
       aKO1        cKO1        bKO1     deltaKO    alphaKO1    gammaKO1
"GJ9C-3965" "GJ9C-3964" "GJ9C-3963" "GJ9C-3962" "GJ9C-3961" "GJ9C-3960"
    betaKO1  epsilonKO1
"GJ9C-3959" "GJ9C-3958"

$BPSE320373$`TUJ9C-3478`
    betaKO2  epsilonKO2        aKO2        cKO2        bKO2    alphaKO2
"GJ9C-6712" "GJ9C-6711" "GJ9C-6708" "GJ9C-6707" "GJ9C-6706" "GJ9C-6705"
   gammaKO2
"GJ9C-6704"


$PACN1091045
$PACN1091045$`TULIL-860`
  epsilonKO      betaKO     gammaKO     alphaKO     deltaKO         bKO
"GLIL-1655" "GLIL-1654" "GLIL-1653" "GLIL-1652" "GLIL-1651" "GLIL-1650"
        cKO         aKO
"GLIL-1649" "GLIL-1648"


$PAER208964
$PAER208964$`TUCXG-10166`
        aKO         cKO         bKO     deltaKO     alphaKO     gammaKO
"GCXG-5851" "GCXG-5850" "GCXG-5849" "GCXG-5848" "GCXG-5847" "GCXG-5846"
     betaKO   epsilonKO
"GCXG-5845" "GCXG-5844"


$AVER998088
$AVER998088$`TUHKF-2391`
        aKO         cKO         bKO     deltaKO     alphaKO     gammaKO
"GHKF-4153" "GHKF-4152" "GHKF-4151" "GHKF-4150" "GHKF-4149" "GHKF-4148"
     betaKO   epsilonKO
"GHKF-4147" "GHKF-4146"


$AWOO931626
$AWOO931626$`TUI4Q-123`
   gammaKO    alphaKO    deltaKO        bKO
"GI4Q-222" "GI4Q-221" "GI4Q-220" "GI4Q-219"

$AWOO931626$`TUI4Q-124`
 epsilonKO     betaKO
"GI4Q-224" "GI4Q-223"

$AWOO931626$`TUI4Q-121`
      cKO1
"GI4Q-217"

$AWOO931626$`TUI4Q-122`
      cKO2
"GI4Q-218"
```

```
$AWOO931626$`TUI4Q-120`
       aKO
"GI4Q-215"


$LFER334390
$LFER334390$`TUJ2S-224`
 epsilonKO     betaKO    gammaKO    alphaKO    deltaKO        bKO
"GJ2S-466" "GJ2S-465" "GJ2S-464" "GJ2S-463" "GJ2S-462" "GJ2S-461"

$LFER334390$`TUJ2S-223`
       cKO        aKO
"GJ2S-460" "GJ2S-459"


$AXYL698758
$AXYL698758$`TUL81-927`
        bKO     deltaKO     alphaKO     gammaKO      betaKO   epsilonKO
"GL81-1756" "GL81-1755" "GL81-1754" "GL81-1753" "GL81-1752" "GL81-1751"

$AXYL698758$`TUL81-928`
        cKO
"GL81-1757"

$AXYL698758$`TUL81-929`
        aKO
"GL81-1758"


$`AXYL1167634-WGS`
$`AXYL1167634-WGS`$`TUSEM-932`
        aKO         cKO         bKO     deltaKO     alphaKO     gammaKO
"GSEM-1802" "GSEM-1801" "GSEM-1800" "GSEM-1799" "GSEM-1798" "GSEM-1797"
     betaKO   epsilonKO
"GSEM-1796" "GSEM-1795"


$PAER941193
$PAER941193$`TULIQ-3143`
        aKO         cKO         bKO     deltaKO     alphaKO     gammaKO
"GLIQ-5760" "GLIQ-5759" "GLIQ-5758" "GLIQ-5757" "GLIQ-5756" "GLIQ-5755"
     betaKO   epsilonKO
"GLIQ-5754" "GLIQ-5753"


$PAER557722
$PAER557722$`TUHJW-3331`
        aKO         cKO         bKO     deltaKO     alphaKO     gammaKO
"GHJW-6052" "GHJW-6051" "GHJW-6050" "GHJW-6049" "GHJW-6048" "GHJW-6047"
     betaKO   epsilonKO
"GHJW-6046" "GHJW-6045"


$PANA932677
```

$PANA932677$`TULI8-1942`
alphaKO deltaKO bKO cKO aKO
"GLI8-3240" "GLI8-3239" "GLI8-3238" "GLI8-3237" "GLI8-3236"

$PANA932677$`TULI8-1943`
epsilonKO betaKO gammaKO
"GLI8-3244" "GLI8-3243" "GLI8-3242"

$BPIL1133568
$BPIL1133568$`TUL9N-431`
cKO bKO deltaKO2 deltaKO1 alphaKO gammaKO
"GL9N-804" "GL9N-803" "GL9N-802" "GL9N-801" "GL9N-800" "GL9N-798"

$BPIL1133568$`TUL9N-478`
betaKO
"GL9N-884"

$BPIL1133568$`TUL9N-372`
epsilonKO
"GL9N-697"

$BPIL1133568$`TUL9N-432`
aKO
"GL9N-805"

$PACN553199
$PACN553199$`TUHRZ-674`
cKO bKO deltaKO alphaKO gammaKO betaKO
"GHRZ-1298" "GHRZ-1297" "GHRZ-1296" "GHRZ-1295" "GHRZ-1294" "GHRZ-1293"
epsilonKO
"GHRZ-1292"

$PACN553199$`TUHRZ-675`
aKO
"GHRZ-1300"

$PANA706191
$PANA706191$`TUJNK-15`
alphaKO deltaKO bKO
"GJNK-25" "GJNK-24" "GJNK-23"

$PANA706191$`TUJNK-16`
epsilonKO betaKO gammaKO
"GJNK-28" "GJNK-27" "GJNK-26"

$PANA706191$`TUJNK-14`
cKO
"GJNK-22"

$PANA706191$`TUJNK-13`
aKO

"GJNK-21"

$PSP592316
$PSP592316$`TUI0L-2993`
epsilonKO betaKO gammaKO alphaKO deltaKO bKO
"GI0L-4088" "GI0L-4087" "GI0L-4086" "GI0L-4085" "GI0L-4084" "GI0L-4083"
cKO aKO
"GI0L-4082" "GI0L-4081"

$PAER381754
$PAER381754$`TUHMY-3356`
aKO cKO bKO deltaKO alphaKO gammaKO
"GHMY-6360" "GHMY-6359" "GHMY-6358" "GHMY-6357" "GHMY-6356" "GHMY-6355"
betaKO epsilonKO
"GHMY-6354" "GHMY-6353"

$PANA1095774
$PANA1095774$`TULIA-157`
aKO cKO bKO deltaKO alphaKO gammaKO betaKO epsilonKO
"GLIA-69" "GLIA-68" "GLIA-67" "GLIA-66" "GLIA-65" "GLIA-64" "GLIA-63" "GLIA-62"

$`LFER767453-WGS`
$`LFER767453-WGS`$`TUSOZ-246`
epsilonKO betaKO gammaKO alphaKO deltaKO bKO cKO
"GSOZ-499" "GSOZ-498" "GSOZ-497" "GSOZ-496" "GSOZ-495" "GSOZ-494" "GSOZ-493"
aKO
"GSOZ-492"

$PARC259536
$PARC259536$`TUI3A-1495`
aKO cKO bKO deltaKO alphaKO gammaKO
"GI3A-2080" "GI3A-2079" "GI3A-2078" "GI3A-2077" "GI3A-2076" "GI3A-2075"
betaKO epsilonKO
"GI3A-2074" "GI3A-2073"

$PATL342610
$PATL342610$`TUHGT-1568`
gammaKO1 alphaKO1 bKO1 cKO1 aKO1 epsilonKO1
"GHGT-2728" "GHGT-2727" "GHGT-2726" "GHGT-2725" "GHGT-2724" "GHGT-2721"
betaKO1
"GHGT-2720"

$PATL342610$`TUHGT-2469`
aKO2 cKO2 bKO2 deltaKO alphaKO2 gammaKO2
"GHGT-4383" "GHGT-4382" "GHGT-4381" "GHGT-4380" "GHGT-4379" "GHGT-4378"
betaKO2 epsilonKO2
"GHGT-4377" "GHGT-4376"

```
$PAER208963
$PAER208963$`TUI5K-3252`
       aKO         cKO         bKO     deltaKO     alphaKO     gammaKO 
"GI5K-5968" "GI5K-5967" "GI5K-5966" "GI5K-5965" "GI5K-5964" "GI5K-5963" 
     betaKO   epsilonKO 
"GI5K-5962" "GI5K-5961" 


$PACN1114967
$PACN1114967$`TUJTL-629`
       aKO         cKO         bKO     deltaKO     alphaKO     gammaKO 
"GJTL-1225" "GJTL-1224" "GJTL-1223" "GJTL-1222" "GJTL-1221" "GJTL-1220" 
     betaKO   epsilonKO 
"GJTL-1219" "GJTL-1218" 


$`LPNE91891-WGS`
$`LPNE91891-WGS`$`TUSPJ-887`
   gammaKO1    alphaKO1        bKO1        cKO1        aKO1  epsilonKO1 
"GSPJ-1669" "GSPJ-1668" "GSPJ-1667" "GSPJ-1666" "GSPJ-1665" "GSPJ-1663" 
    betaKO1 
"GSPJ-1662" 

$`LPNE91891-WGS`$`TUSPJ-1714`
       aKO2        cKO2        bKO2     deltaKO    alphaKO2    gammaKO2 
"GSPJ-3170" "GSPJ-3169" "GSPJ-3168" "GSPJ-3167" "GSPJ-3166" "GSPJ-3165" 
    betaKO2  epsilonKO2 
"GSPJ-3164" "GSPJ-3163" 


$PACN909952
$PACN909952$`TULIJ-654`
       aKO         cKO         bKO     deltaKO     alphaKO     gammaKO 
"GLIJ-1290" "GLIJ-1289" "GLIJ-1288" "GLIJ-1287" "GLIJ-1286" "GLIJ-1285" 
     betaKO   epsilonKO 
"GLIJ-1284" "GLIJ-1283" 


$PACN1114969
$PACN1114969$`TUJUU-637`
       aKO         cKO         bKO     deltaKO     alphaKO     gammaKO 
"GJUU-1229" "GJUU-1228" "GJUU-1227" "GJUU-1226" "GJUU-1225" "GJUU-1224" 
     betaKO   epsilonKO 
"GJUU-1223" "GJUU-1222" 


$PACN1114966
$PACN1114966$`TUJX4-617`
       aKO         cKO         bKO     deltaKO     alphaKO     gammaKO 
"GJX4-1213" "GJX4-1212" "GJX4-1211" "GJX4-1210" "GJX4-1209" "GJX4-1208" 
     betaKO   epsilonKO 
"GJX4-1207" "GJX4-1206" 
```

```
$PBRA994484
$PBRA994484$`TUJWC-3355`
        aKO         cKO         bKO     deltaKO     alphaKO     gammaKO
"GJWC-6165" "GJWC-6164" "GJWC-6163" "GJWC-6162" "GJWC-6161" "GJWC-6160"
     betaKO   epsilonKO
"GJWC-6159" "GJWC-6158"


$PACI1171373
$PACI1171373$`TULII-977`
        aKO         cKO         bKO     deltaKO     alphaKO     gammaKO
"GLII-1941" "GLII-1940" "GLII-1939" "GLII-1938" "GLII-1937" "GLII-1936"
     betaKO   epsilonKO
"GLII-1935" "GLII-1934"


$PBER314260
$PBER314260$`TUI0G-521`
    betaKO    gammaKO    alphaKO    deltaKO
"GI0G-952" "GI0G-951" "GI0G-949" "GI0G-948"

$PBER314260$`TUI0G-522`
 epsilonKO
"GI0G-956"

$PBER314260$`TUI0G-196`
       cKO
"GI0G-336"

$PBER314260$`TUI0G-195`
       aKO
"GI0G-335"

$PBER314260$`TUI0G-197`
      bKO2       bKO1
"GI0G-338" "GI0G-337"


$LFER1048260
$LFER1048260$`TULFL-46`
epsilonKO    betaKO   gammaKO   alphaKO   deltaKO       bKO       aKO
"GLFL-86" "GLFL-85" "GLFL-84" "GLFL-83" "GLFL-82" "GLFL-81" "GLFL-79"

$LFER1048260$noTU
cKO
 NA


$PBRA756272
$PBRA756272$`TUH5Q-842`
  epsilonKO      betaKO     gammaKO     alphaKO     deltaKO         bKO
"GH5Q-1211" "GH5Q-1210" "GH5Q-1209" "GH5Q-1208" "GH5Q-1207" "GH5Q-1206"
        cKO         aKO
```

```
"GH5Q-1205" "GH5Q-1204"


$PCAR1218933
$PCAR1218933$`TULIC-2465`
        aKO         cKO         bKO     deltaKO     alphaKO     gammaKO
"GLIC-4340" "GLIC-4339" "GLIC-4338" "GLIC-4337" "GLIC-4336" "GLIC-4335"
     betaKO   epsilonKO
"GLIC-4334" "GLIC-4333"


$PCLA701521
$PCLA701521$`TUKFD-378`
 epsilonKO     betaKO    gammaKO    alphaKO    deltaKO        bKO        cKO
"GKFD-649" "GKFD-648" "GKFD-647" "GKFD-646" "GKFD-645" "GKFD-644" "GKFD-643"
       aKO
"GKFD-642"


$`BPSE1241583-WGS`
$`BPSE1241583-WGS`$`TUSHJ-884`
 epsilonKO1     betaKO1    gammaKO1    alphaKO1     deltaKO        bKO1
"GSHJ-1467" "GSHJ-1466" "GSHJ-1465" "GSHJ-1464" "GSHJ-1463" "GSHJ-1462"
       cKO1        aKO1
"GSHJ-1461" "GSHJ-1460"

$`BPSE1241583-WGS`$`TUSHJ-2744`
   alphaKO2    gammaKO2
"GSHJ-4679" "GSHJ-4678"

$`BPSE1241583-WGS`$`TUSHJ-2746`
    betaKO2  epsilonKO2
"GSHJ-4686" "GSHJ-4685"

$`BPSE1241583-WGS`$`TUSHJ-2745`
       aKO2        cKO2        bKO2
"GSHJ-4682" "GSHJ-4681" "GSHJ-4680"


$PACN1031709
$PACN1031709$`TULIK-653`
        aKO         cKO         bKO     deltaKO     alphaKO     gammaKO
"GLIK-1278" "GLIK-1277" "GLIK-1276" "GLIK-1275" "GLIK-1274" "GLIK-1273"
     betaKO   epsilonKO
"GLIK-1272" "GLIK-1271"


$PCRY335284
$PCRY335284$`TUHE9-1751`
        aKO         cKO         bKO     deltaKO     alphaKO
"GHE9-2382" "GHE9-2381" "GHE9-2380" "GHE9-2379" "GHE9-2378"

$PCRY335284$`TUHE9-1750`
    gammaKO      betaKO   epsilonKO
```

```
"GHE9-2377" "GHE9-2376" "GHE9-2375"


$PCAR561230
$PCAR561230$`TUKCK-2451`
  epsilonKO      betaKO     gammaKO     alphaKO     deltaKO         bKO
"GKCK-4362" "GKCK-4361" "GKCK-4360" "GKCK-4359" "GKCK-4358" "GKCK-4357"
        cKO         aKO
"GKCK-4356" "GKCK-4355"


$PDEN318586
$PDEN318586$`TUCVQ-2105`
  epsilonKO      betaKO     gammaKO     alphaKO     deltaKO
"GCVQ-3862" "GCVQ-3861" "GCVQ-3860" "GCVQ-3859" "GCVQ-3858"

$PDEN318586$`TUCVQ-1658`
        aKO         cKO        bKO2        bKO1
"GCVQ-2921" "GCVQ-2920" "GCVQ-2919" "GCVQ-2918"


$PDIS435591
$PDIS435591$`TUCNH-128`
    betaKO  epsilonKO        aKO        cKO        bKO    deltaKO    alphaKO
"GCNH-271" "GCNH-270" "GCNH-268" "GCNH-267" "GCNH-266" "GCNH-265" "GCNH-264"
   gammaKO
"GCNH-263"


$PAER1093787
$PAER1093787$`TULIP-3235`
        aKO         cKO         bKO     deltaKO     alphaKO     gammaKO
"GLIP-5950" "GLIP-5949" "GLIP-5948" "GLIP-5947" "GLIP-5946" "GLIP-5945"
     betaKO   epsilonKO
"GLIP-5944" "GLIP-5943"


$PDEN767031
$PDEN767031$`TUHQS-610`
    gammaKO     alphaKO     deltaKO         bKO         cKO         aKO
"GHQS-1020" "GHQS-1019" "GHQS-1018" "GHQS-1017" "GHQS-1016" "GHQS-1015"
  epsilonKO      betaKO
"GHQS-1013" "GHQS-1012"


$LFER712938
$LFER712938$`TULF8-179`
    betaKO    gammaKO    alphaKO
"GLF8-306" "GLF8-305" "GLF8-304"

$LFER712938$noTU
  deltaKO epsilonKO       cKO       aKO       bKO
       NA        NA        NA        NA        NA
```

```
$`PDEN1294143-WGS`
$`PDEN1294143-WGS`$`TUSSR-2932`
        aKO         cKO         bKO     deltaKO     alphaKO     gammaKO 
"GSSR-5125" "GSSR-5124" "GSSR-5123" "GSSR-5122" "GSSR-5121" "GSSR-5120" 
     betaKO   epsilonKO 
"GSSR-5119" "GSSR-5118" 


$`PDEN908937-WGS`
$`PDEN908937-WGS`$`TUSSI-591`
    gammaKO     alphaKO     deltaKO         bKO         cKO         aKO 
"GSSI-2650" "GSSI-2649" "GSSI-2648" "GSSI-2647" "GSSI-2646" "GSSI-2645" 
  epsilonKO      betaKO 
"GSSI-2642" "GSSI-2641" 


$HPYL290847
$HPYL290847$`TULE5-473`
       bKO2        bKO1     deltaKO     alphaKO     gammaKO      betaKO 
"GLE5-1064" "GLE5-1063" "GLE5-1062" "GLE5-1061" "GLE5-1060" "GLE5-1059" 
  epsilonKO 
"GLE5-1058" 

$HPYL290847$`TULE5-512`
        cKO 
"GLE5-1146" 

$HPYL290847$`TULE5-220`
       aKO 
"GLE5-512" 


$BPSE357348
$BPSE357348$`TUHVF-2181`
       aKO1        cKO1        bKO1     deltaKO    alphaKO1    gammaKO1 
"GHVF-4048" "GHVF-4047" "GHVF-4046" "GHVF-4045" "GHVF-4044" "GHVF-4043" 
    betaKO1  epsilonKO1 
"GHVF-4042" "GHVF-4041" 

$BPSE357348$`TUHVF-3533`
    betaKO2  epsilonKO2        aKO2        cKO2        bKO2    alphaKO2 
"GHVF-6734" "GHVF-6733" "GHVF-6730" "GHVF-6729" "GHVF-6728" "GHVF-6727" 
   gammaKO2 
"GHVF-6726" 


$PDIO675635
$PDIO675635$`TUHMF-1065`
  epsilonKO      betaKO     gammaKO     alphaKO     deltaKO         bKO 
"GHMF-1712" "GHMF-1711" "GHMF-1710" "GHMF-1709" "GHMF-1708" "GHMF-1706" 
        cKO         aKO 
"GHMF-1705" "GHMF-1704" 
```

```
$CPEL1002672
$CPEL1002672$`TUHAA-183`
 epsilonKO     betaKO    gammaKO    alphaKO    deltaKO
"GHAA-499" "GHAA-498" "GHAA-497" "GHAA-496" "GHAA-495"

$CPEL1002672$`TUHAA-260`
       aKO        cKO       bKO2       bKO1
"GHAA-711" "GHAA-710" "GHAA-709" "GHAA-708"


$PENT384676
$PENT384676$`TUJB8-2792`
        aKO         cKO         bKO     deltaKO     alphaKO     gammaKO
"GJB8-5266" "GJB8-5265" "GJB8-5264" "GJB8-5263" "GJB8-5262" "GJB8-5261"
     betaKO   epsilonKO
"GJB8-5260" "GJB8-5259"


$PFLU1037911
$PFLU1037911$`TULIS-2895`
        aKO         cKO         bKO     deltaKO     alphaKO     gammaKO
"GLIS-5418" "GLIS-5417" "GLIS-5416" "GLIS-5415" "GLIS-5414" "GLIS-5413"
     betaKO   epsilonKO
"GLIS-5412" "GLIS-5411"


$PFLU1114970
$PFLU1114970$`TUJXA-3268`
        bKO     deltaKO     alphaKO
"GJXA-5952" "GJXA-5951" "GJXA-5950"

$PFLU1114970$`TUJXA-3267`
    gammaKO      betaKO
"GJXA-5949" "GJXA-5948"

$PFLU1114970$`TUJXA-3266`
  epsilonKO
"GJXA-5947"

$PFLU1114970$`TUJXA-3269`
        cKO
"GJXA-5953"

$PFLU1114970$`TUJXA-3270`
        aKO
"GJXA-5954"


$PFLU220664
$PFLU220664$`TUIX8-3430`
        bKO     deltaKO     alphaKO
"GIX8-6263" "GIX8-6262" "GIX8-6261"
```

```
$PFLU220664$`TUIX8-3429`
    gammaKO      betaKO 
"GIX8-6260" "GIX8-6259" 

$PFLU220664$`TUIX8-3428`
  epsilonKO 
"GIX8-6258" 

$PFLU220664$`TUIX8-3431`
        cKO 
"GIX8-6264" 

$PFLU220664$`TUIX8-3432`
        aKO 
"GIX8-6265" 


$PFLU205922
$PFLU205922$`TUJBD-3020`
        aKO         cKO         bKO     deltaKO     alphaKO     gammaKO 
"GJBD-5820" "GJBD-5819" "GJBD-5818" "GJBD-5817" "GJBD-5816" "GJBD-5815" 
     betaKO   epsilonKO 
"GJBD-5814" "GJBD-5813" 


$PFRE754252
$PFRE754252$`TUI1A-611`
  epsilonKO      betaKO     gammaKO     alphaKO     deltaKO         bKO 
"GI1A-1098" "GI1A-1097" "GI1A-1096" "GI1A-1095" "GI1A-1094" "GI1A-1093" 
        cKO         aKO 
"GI1A-1092" "GI1A-1091" 


$LGAS324831
$LGAS324831$`TUHTY-612`
        cKO         bKO     deltaKO     alphaKO     gammaKO      betaKO 
"GHTY-1243" "GHTY-1242" "GHTY-1241" "GHTY-1240" "GHTY-1239" "GHTY-1238" 
  epsilonKO 
"GHTY-1237" 

$LGAS324831$`TUHTY-613`
        aKO 
"GHTY-1244" 


$AXYL762376
$AXYL762376$`TUJUB-363`
 epsilonKO     betaKO    gammaKO    alphaKO    deltaKO        bKO        cKO 
"GJUB-378" "GJUB-377" "GJUB-376" "GJUB-375" "GJUB-374" "GJUB-373" "GJUB-372" 
       aKO 
"GJUB-371" 


$BPSE320372
```

$BPSE320372$`TUBYB-102`
aKO1 cKO1 bKO1 deltaKO alphaKO1 gammaKO1 betaKO1
"GBYB-185" "GBYB-184" "GBYB-183" "GBYB-182" "GBYB-181" "GBYB-180" "GBYB-179"

$BPSE320372$`TUBYB-2740`
bKO2 alphaKO2 gammaKO2
"GBYB-4847" "GBYB-4846" "GBYB-4845"

$BPSE320372$`TUBYB-2741`
betaKO2 epsilonKO2 aKO2 cKO2
"GBYB-4854" "GBYB-4853" "GBYB-4850" "GBYB-4849"

$BPSE320372$`TUBYB-101`
epsilonKO1
"GBYB-177"

$ACAU438753
$ACAU438753$`TUJF3-2203`
deltaKO alphaKO gammaKO betaKO epsilonKO
"GJF3-4177" "GJF3-4176" "GJF3-4175" "GJF3-4174" "GJF3-4173"

$ACAU438753$`TUJF3-2289`
bKO1 cKO aKO
"GJF3-4317" "GJF3-4316" "GJF3-4315"

$ACAU438753$`TUJF3-2290`
bKO2
"GJF3-4318"

$ASP62928
$ASP62928$`TUCO7-6480`
epsilonKO betaKO gammaKO alphaKO deltaKO bKO cKO
"GCO7-407" "GCO7-406" "GCO7-405" "GCO7-404" "GCO7-403" "GCO7-402" "GCO7-401"
aKO
"GCO7-400"

$PFLU216595
$PFLU216595$`TUBYM-11884`
aKO cKO bKO deltaKO alphaKO gammaKO
"GBYM-5470" "GBYM-5473" "GBYM-5474" "GBYM-5476" "GBYM-5469" "GBYM-5475"
betaKO epsilonKO
"GBYM-5472" "GBYM-5471"

$PFUL743720
$PFUL743720$`TUHQV-2483`
aKO cKO bKO deltaKO alphaKO gammaKO
"GHQV-4564" "GHQV-4563" "GHQV-4562" "GHQV-4561" "GHQV-4560" "GHQV-4559"
betaKO epsilonKO
"GHQV-4558" "GHQV-4557"

$PGAL391619
$PGAL391619$`TULIE-1551`
deltaKO alphaKO gammaKO betaKO epsilonKO
"GLIE-2491" "GLIE-2490" "GLIE-2489" "GLIE-2488" "GLIE-2487"

$PGAL391619$`TULIE-223`
bKO2 bKO1 cKO aKO
"GLIE-72" "GLIE-71" "GLIE-70" "GLIE-69"

$PGAL383629
$PGAL383629$`TULID-1482`
deltaKO alphaKO gammaKO betaKO epsilonKO
"GLID-2311" "GLID-2310" "GLID-2309" "GLID-2308" "GLID-2307"

$PGAL383629$`TULID-1849`
aKO cKO bKO2 bKO1
"GLID-3016" "GLID-3015" "GLID-3014" "GLID-3013"

$PGIL991905
$PGIL991905$`TUJ0L-2333`
deltaKO alphaKO gammaKO betaKO epsilonKO
"GJ0L-4267" "GJ0L-4266" "GJ0L-4265" "GJ0L-4264" "GJ0L-4263"

$PGIL991905$`TUJ0L-1898`
aKO cKO
"GJ0L-3406" "GJ0L-3405"

$PGIL991905$`TUJ0L-1897`
bKO2 bKO1
"GJ0L-3404" "GJ0L-3403"

$PHAL326442
$PHAL326442$`TUJIU-1601`
aKO cKO bKO deltaKO alphaKO gammaKO
"GJIU-3063" "GJIU-3062" "GJIU-3061" "GJIU-3060" "GJIU-3059" "GJIU-3058"
betaKO epsilonKO
"GJIU-3057" "GJIU-3056"

$PHEP485917
$PHEP485917$`TUHL9-201`
gammaKO alphaKO deltaKO bKO cKO aKO
"GHL9-399" "GHL9-398" "GHL9-397" "GHL9-396" "GHL9-395" "GHL9-394"

$PHEP485917$`TUHL9-1771`
epsilonKO betaKO
"GHL9-3453" "GHL9-3452"

$LGEL1229756

```
$LGEL1229756$`TULFO-960`
       cKO         bKO     deltaKO     alphaKO     gammaKO      betaKO
"GLFO-1738" "GLFO-1737" "GLFO-1736" "GLFO-1735" "GLFO-1734" "GLFO-1733"
  epsilonKO
"GLFO-1732"

$LGEL1229756$`TULFO-961`
       aKO
"GLFO-1739"


$BPIL759914
$BPIL759914$`TUHZ5-325`
   alphaKO   deltaKO2   deltaKO1        bKO        cKO        aKO
"GHZ5-568" "GHZ5-567" "GHZ5-566" "GHZ5-565" "GHZ5-564" "GHZ5-563"

$BPIL759914$`TUHZ5-288`
    betaKO
"GHZ5-498"

$BPIL759914$`TUHZ5-326`
   gammaKO
"GHZ5-571"

$BPIL759914$`TUHZ5-1093`
  epsilonKO
"GHZ5-1879"


$PHAL1082931
$PHAL1082931$`TUJXT-1667`
    deltaKO     alphaKO     gammaKO      betaKO   epsilonKO
"GJXT-3173" "GJXT-3172" "GJXT-3171" "GJXT-3170" "GJXT-3169"

$PHAL1082931$`TUJXT-283`
      aKO        cKO       bKO2       bKO1
"GJXT-518" "GJXT-517" "GJXT-516" "GJXT-515"


$PMIK1142394
$PMIK1142394$`TULIF-161`
   alphaKO    deltaKO        bKO        aKO
"GLIF-203" "GLIF-202" "GLIF-201" "GLIF-199"

$PMIK1142394$`TULIF-1997`
     betaKO
"GLIF-3099"

$PMIK1142394$`TULIF-1699`
    gammaKO
"GLIF-2617"

$PMIK1142394$`TULIF-547`
 epsilonKO
```

"GLIF-821"

$PMIK1142394$noTU
cKO
 NA

$PING357804
$PING357804$`TUJBJ-276`
  gammaKO1   alphaKO1   bKO1   cKO1   aKO1 epsilonKO1   betaKO1
"GJBJ-495" "GJBJ-494" "GJBJ-493" "GJBJ-492" "GJBJ-491" "GJBJ-488" "GJBJ-487"

$PING357804$`TUJBJ-2307`
       aKO2   cKO2   bKO2   deltaKO   alphaKO2
"GJBJ-3858" "GJBJ-3857" "GJBJ-3856" "GJBJ-3855" "GJBJ-3854"

$PING357804$`TUJBJ-2306`
   gammaKO2   betaKO2 epsilonKO2
"GJBJ-3853" "GJBJ-3852" "GJBJ-3851"

$PINT246198
$PINT246198$`TULIH-1236`
     betaKO   epsilonKO   aKO   cKO   bKO   deltaKO
"GLIH-1645" "GLIH-1644" "GLIH-1642" "GLIH-1641" "GLIH-1640" "GLIH-1639"
    alphaKO   gammaKO
"GLIH-1638" "GLIH-1637"

$PSP324057
$PSP324057$`TUH5H-3217`
        aKO   cKO   bKO   deltaKO   alphaKO   gammaKO
"GH5H-5947" "GH5H-5946" "GH5H-5945" "GH5H-5944" "GH5H-5943" "GH5H-5942"
     betaKO   epsilonKO
"GH5H-5941" "GH5H-5940"

$PLAV402881
$PLAV402881$`TUHQA-811`
  epsilonKO   betaKO   gammaKO   alphaKO   deltaKO
"GHQA-1483" "GHQA-1482" "GHQA-1481" "GHQA-1480" "GHQA-1479"

$PLAV402881$`TUHQA-393`
       aKO   cKO
"GHQA-707" "GHQA-706"

$PLAV402881$`TUHQA-392`
      bKO2   bKO1
"GHQA-705" "GHQA-704"

$`CPOR1297582-WGS`
$`CPOR1297582-WGS`$`TUSJ0-63`
       aKO   cKO   bKO   deltaKO   alphaKO   gammaKO   betaKO

```
"GSJ0-266" "GSJ0-265" "GSJ0-264" "GSJ0-263" "GSJ0-262" "GSJ0-261" "GSJ0-260"
 epsilonKO
"GSJ0-259"


$`CPOR1206109-WGS`
$`CPOR1206109-WGS`$`TUSJC-10`
epsilonKO    betaKO   gammaKO   alphaKO   deltaKO       bKO       cKO       aKO
"GSJC-34" "GSJC-33" "GSJC-32" "GSJC-31" "GSJC-30" "GSJC-29" "GSJC-28" "GSJC-27"


$PANA1123863
$PANA1123863$`TUJWO-38`
      aKO       cKO       bKO   deltaKO   alphaKO   gammaKO    betaKO epsilonKO
"GJWO-70" "GJWO-69" "GJWO-68" "GJWO-67" "GJWO-66" "GJWO-65" "GJWO-64" "GJWO-63"


$PLIM521674
$PLIM521674$`TUKE7-154`
 epsilonKO     betaKO    gammaKO    alphaKO    deltaKO        bKO        cKO
"GKE7-178" "GKE7-177" "GKE7-176" "GKE7-175" "GKE7-174" "GKE7-173" "GKE7-172"
       aKO
"GKE7-171"


$BPSE272560
$BPSE272560$`TUJNI-2017`
       aKO1        cKO1        bKO1     deltaKO    alphaKO1    gammaKO1
"GJNI-3501" "GJNI-3500" "GJNI-3499" "GJNI-3498" "GJNI-3497" "GJNI-3496"
    betaKO1  epsilonKO1
"GJNI-3495" "GJNI-3494"

$BPSE272560$`TUJNI-3182`
    betaKO2  epsilonKO2        aKO2        cKO2        bKO2    alphaKO2
"GJNI-5529" "GJNI-5528" "GJNI-5525" "GJNI-5524" "GJNI-5523" "GJNI-5522"
   gammaKO2
"GJNI-5521"


$LGAR420889
$LGAR420889$`TUH14-237`
   gammaKO    alphaKO
"GH14-424" "GH14-423"

$LGAR420889$`TUH14-238`
 epsilonKO     betaKO
"GH14-426" "GH14-425"

$LGAR420889$`TUH14-236`
   deltaKO        bKO        aKO
"GH14-422" "GH14-421" "GH14-420"

$LGAR420889$`TUH14-235`
       cKO
```

"GH14-419"

$`CPOR1239881-WGS`
$`CPOR1239881-WGS`$`TUSIZ-7`
epsilonKO betaKO gammaKO alphaKO deltaKO bKO cKO aKO
"GSIZ-30" "GSIZ-29" "GSIZ-28" "GSIZ-27" "GSIZ-26" "GSIZ-25" "GSIZ-24" "GSIZ-23"

$PMIN118163
$PMIN118163$`TULIG-1213`
bKO2 bKO1 deltaKO alphaKO gammaKO
"GLIG-1737" "GLIG-1736" "GLIG-1735" "GLIG-1734" "GLIG-1733"

$PMIN118163$`TULIG-229`
epsilonKO betaKO1
"GLIG-325" "GLIG-324"

$PMIN118163$`TULIG-2001`
betaKO2
"GLIG-2927"

$PMIN118163$`TULIG-1214`
cKO
"GLIG-1738"

$PMIN118163$`TULIG-1215`
aKO
"GLIG-1739"

$PLUT319225
$PLUT319225$`TUHDM-529`
gammaKO1 alphaKO1 bKO1 cKO1 aKO1 epsilonKO2
"GHDM-1078" "GHDM-1077" "GHDM-1076" "GHDM-1075" "GHDM-1074" "GHDM-1071"
betaKO2
"GHDM-1070"

$PLUT319225$`TUHDM-1002`
alphaKO2
"GHDM-1988"

$PLUT319225$`TUHDM-14`
betaKO1 epsilonKO1
"GHDM-21" "GHDM-20"

$PLUT319225$`TUHDM-1001`
gammaKO2
"GHDM-1987"

$PLUT319225$`TUHDM-1052`
bKO2 deltaKO
"GHDM-2100" "GHDM-2099"

```
$PLUT319225$`TUHDM-1053`
      cKO2
"GHDM-2101"

$PLUT319225$`TUHDM-1054`
      aKO2
"GHDM-2102"



$`PLUM243265-WGS`
$`PLUM243265-WGS`$`TUSUC-16`
      aKO       cKO       bKO   deltaKO   alphaKO   gammaKO    betaKO epsilonKO
"GSUC-46" "GSUC-45" "GSUC-44" "GSUC-43" "GSUC-42" "GSUC-41" "GSUC-40" "GSUC-39"



$PMAR146891
$PMAR146891$`TUH90-841`
        aKO         cKO        bKO2        bKO1     deltaKO     alphaKO
"GH90-1689" "GH90-1688" "GH90-1687" "GH90-1686" "GH90-1685" "GH90-1684"
    gammaKO
"GH90-1683"

$PMAR146891$`TUH90-836`
  epsilonKO      betaKO
"GH90-1672" "GH90-1671"



$PMAR167542
$PMAR167542$`TUI3N-857`
        aKO         cKO        bKO2        bKO1     deltaKO     alphaKO
"GI3N-1682" "GI3N-1681" "GI3N-1680" "GI3N-1679" "GI3N-1678" "GI3N-1677"
    gammaKO
"GI3N-1676"

$PMAR167542$`TUI3N-852`
  epsilonKO      betaKO
"GI3N-1665" "GI3N-1664"



$PMAR167555
$PMAR167555$`TUI3K-1107`
        aKO         cKO        bKO2        bKO1     deltaKO     alphaKO
"GI3K-1894" "GI3K-1893" "GI3K-1892" "GI3K-1891" "GI3K-1890" "GI3K-1889"
    gammaKO
"GI3K-1888"

$PMAR167555$`TUI3K-1102`
  epsilonKO      betaKO
"GI3K-1879" "GI3K-1878"



$PMAR59922
$PMAR59922$`TUH54-273`
   gammaKO    alphaKO    deltaKO       bKO2       bKO1        cKO        aKO
```

"GH54-542" "GH54-541" "GH54-540" "GH54-539" "GH54-538" "GH54-537" "GH54-536"

$PMAR59922$`TUH54-282`
betaKO epsilonKO
"GH54-560" "GH54-559"

$PMAR167546
$PMAR167546$`TUH1Y-830`
aKO cKO bKO2 bKO1 deltaKO alphaKO
"GH1Y-1690" "GH1Y-1689" "GH1Y-1688" "GH1Y-1687" "GH1Y-1686" "GH1Y-1685"
gammaKO
"GH1Y-1684"

$PMAR167546$`TUH1Y-825`
epsilonKO betaKO
"GH1Y-1673" "GH1Y-1672"

$`BPSE1335307-WGS`
$`BPSE1335307-WGS`$`TUSHI-1195`
epsilonKO1 betaKO1 gammaKO1 alphaKO1 deltaKO bKO1
"GSHI-1978" "GSHI-1977" "GSHI-1976" "GSHI-1975" "GSHI-1974" "GSHI-1973"
cKO1 aKO1
"GSHI-1972" "GSHI-1971"

$`BPSE1335307-WGS`$`TUSHI-3126`
betaKO2 epsilonKO2 aKO2 cKO2 bKO2 alphaKO2
"GSHI-5344" "GSHI-5343" "GSHI-5340" "GSHI-5339" "GSHI-5338" "GSHI-5337"
gammaKO2
"GSHI-5336"

$PMAR93060
$PMAR93060$`TUI08-889`
aKO cKO bKO2 bKO1 deltaKO alphaKO
"GI08-1779" "GI08-1778" "GI08-1777" "GI08-1776" "GI08-1775" "GI08-1774"
gammaKO
"GI08-1773"

$PMAR93060$`TUI08-883`
epsilonKO betaKO
"GI08-1762" "GI08-1761"

$LGAS762550
$LGAS762550$`TUHH1-1017`
aKO cKO bKO deltaKO alphaKO gammaKO
"GHH1-1098" "GHH1-1101" "GHH1-1102" "GHH1-1104" "GHH1-1097" "GHH1-1103"
betaKO epsilonKO
"GHH1-1100" "GHH1-1099"

$PMAR74546

$PMAR74546$`TUHRG-774`
aKO cKO bKO2 bKO1 deltaKO alphaKO
"GHRG-1586" "GHRG-1585" "GHRG-1584" "GHRG-1583" "GHRG-1582" "GHRG-1581"
gammaKO
"GHRG-1580"

$PMAR74546$`TUHRG-769`
epsilonKO betaKO
"GHRG-1569" "GHRG-1568"

$`PMIR1266738-WGS`
$`PMIR1266738-WGS`$`TUSSO-1765`
epsilonKO betaKO gammaKO alphaKO deltaKO bKO
"GSSO-3079" "GSSO-3078" "GSSO-3077" "GSSO-3076" "GSSO-3075" "GSSO-3074"
cKO aKO
"GSSO-3073" "GSSO-3072"

$PMAR93059
$PMAR93059$`TUHJV-844`
aKO cKO bKO2 bKO1 deltaKO alphaKO
"GHJV-1612" "GHJV-1611" "GHJV-1610" "GHJV-1609" "GHJV-1608" "GHJV-1607"
gammaKO
"GHJV-1606"

$PMAR93059$`TUHJV-842`
epsilonKO betaKO
"GHJV-1595" "GHJV-1594"

$PMEN1001585
$PMEN1001585$`TUIWS-2759`
aKO cKO bKO deltaKO alphaKO gammaKO
"GIWS-5025" "GIWS-5024" "GIWS-5023" "GIWS-5022" "GIWS-5021" "GIWS-5020"
betaKO epsilonKO
"GIWS-5019" "GIWS-5018"

$PMAR59920
$PMAR59920$`TUI1O-1065`
aKO cKO bKO2 bKO1 deltaKO alphaKO
"GI1O-1856" "GI1O-1855" "GI1O-1854" "GI1O-1853" "GI1O-1852" "GI1O-1851"
gammaKO
"GI1O-1850"

$PMAR59920$`TUI1O-1060`
epsilonKO betaKO
"GI1O-1841" "GI1O-1840"

$PMOB403833
$PMOB403833$`TUH51-350`
aKO cKO bKO deltaKO alphaKO gammaKO betaKO

```
"GH51-776" "GH51-775" "GH51-774" "GH51-773" "GH51-772" "GH51-771" "GH51-770"
 epsilonKO
"GH51-769"


$PMUL1075089
$PMUL1075089$`TUJSX-907`
  epsilonKO      betaKO     gammaKO     alphaKO     deltaKO         bKO
"GJSX-1729" "GJSX-1728" "GJSX-1727" "GJSX-1726" "GJSX-1725" "GJSX-1724"
        cKO         aKO
"GJSX-1723" "GJSX-1722"


$PMUC1116391
$PMUC1116391$`TULI5-29`
  alphaKO   deltaKO       bKO
"GLI5-49" "GLI5-48" "GLI5-47"

$PMUC1116391$`TULI5-31`
   betaKO
"GLI5-51"

$PMUC1116391$`TULI5-30`
  gammaKO
"GLI5-50"

$PMUC1116391$`TULI5-32`
epsilonKO
"GLI5-52"

$PMUC1116391$`TULI5-28`
      cKO       aKO
"GLI5-46" "GLI5-45"


$BSP335659
$BSP335659$`TUL9K-248`
    betaKO    gammaKO    alphaKO    deltaKO
"GL9K-403" "GL9K-402" "GL9K-401" "GL9K-400"

$BSP335659$`TUL9K-249`
 epsilonKO
"GL9K-405"

$BSP335659$`TUL9K-468`
       aKO        cKO       bKO2       bKO1
"GL9K-808" "GL9K-807" "GL9K-806" "GL9K-805"


$PMIR529507
$PMIR529507$`TUJIW-1712`
  epsilonKO      betaKO     gammaKO     alphaKO     deltaKO         bKO
"GJIW-3126" "GJIW-3125" "GJIW-3124" "GJIW-3123" "GJIW-3122" "GJIW-3121"
        cKO         aKO
```

```
"GJIW-3120" "GJIW-3119"


$BABO1104320
$BABO1104320$`TUKD4-1003`
    betaKO    gammaKO    alphaKO    deltaKO 
"GKD4-700" "GKD4-699" "GKD4-698" "GKD4-697" 

$BABO1104320$`TUKD4-1004`
 epsilonKO 
"GKD4-701" 

$BABO1104320$`TUKD4-1816`
        aKO         cKO        bKO2        bKO1 
"GKD4-2120" "GKD4-2119" "GKD4-2118" "GKD4-2117" 


$LGAR420890
$LGAR420890$`TULFD-237`
   gammaKO    alphaKO 
"GLFD-424" "GLFD-423" 

$LGAR420890$`TULFD-238`
 epsilonKO     betaKO 
"GLFD-426" "GLFD-425" 

$LGAR420890$`TULFD-236`
   deltaKO        bKO        aKO 
"GLFD-422" "GLFD-421" "GLFD-420" 

$LGAR420890$`TULFD-235`
       cKO 
"GLFD-419" 


$BAPH224915
$BAPH224915$`TUJ9D-5`
epsilonKO    betaKO   gammaKO   alphaKO   deltaKO       bKO       cKO       aKO 
 "GJ9D-9"  "GJ9D-8"  "GJ9D-7"  "GJ9D-6"  "GJ9D-5"  "GJ9D-4"  "GJ9D-3"  "GJ9D-2" 


$BAMB398577
$BAMB398577$`TUH38-222`
 epsilonKO     betaKO    gammaKO    alphaKO    deltaKO        bKO        cKO 
"GH38-111" "GH38-110" "GH38-109" "GH38-108" "GH38-107" "GH38-106" "GH38-105" 
       aKO 
"GH38-104" 


$BADO367928
$BADO367928$`TUHPT-897`
        aKO         cKO         bKO     deltaKO     alphaKO     gammaKO 
"GHPT-1490" "GHPT-1489" "GHPT-1488" "GHPT-1487" "GHPT-1486" "GHPT-1485" 
     betaKO   epsilonKO 
```

```
"GHPT-1484" "GHPT-1483"


$PMUC1036673
$PMUC1036673$`TUJD1-29`
  alphaKO   deltaKO       bKO
"GJD1-52" "GJD1-51" "GJD1-50"

$PMUC1036673$`TUJD1-31`
   betaKO
"GJD1-54"

$PMUC1036673$`TUJD1-30`
  gammaKO
"GJD1-53"

$PMUC1036673$`TUJD1-32`
epsilonKO
"GJD1-55"

$PMUC1036673$`TUJD1-28`
      cKO
"GJD1-49"

$PMUC1036673$`TUJD1-27`
      aKO
"GJD1-48"


$PMUL272843
$PMUL272843$`TUC8W-792`
  epsilonKO      betaKO     gammaKO     alphaKO     deltaKO         bKO
"GC8W-1548" "GC8W-1547" "GC8W-1546" "GC8W-1545" "GC8W-1544" "GC8W-1543"
        cKO         aKO
"GC8W-1542" "GC8W-1541"


$PMUL1132496
$PMUL1132496$`TULIB-936`
  epsilonKO      betaKO     gammaKO     alphaKO     deltaKO         bKO
"GLIB-1797" "GLIB-1796" "GLIB-1795" "GLIB-1794" "GLIB-1793" "GLIB-1792"
        cKO         aKO
"GLIB-1791" "GLIB-1790"


$PMUC997761
$PMUC997761$`TULI6-27`
  gammaKO   alphaKO   deltaKO       bKO
"GLI6-52" "GLI6-51" "GLI6-50" "GLI6-49"

$PMUC997761$`TULI6-28`
   betaKO
"GLI6-53"
```

```
$PMUC997761$`TULI6-29`
epsilonKO
"GLI6-54"

$PMUC997761$`TULI6-26`
      cKO
"GLI6-48"

$PMUC997761$noTU
aKO
 NA


$`BTHA1249661-WGS`
$`BTHA1249661-WGS`$`TUSHM-1376`
epsilonKO1    betaKO1   gammaKO1   alphaKO1    deltaKO       bKO1       cKO1
"GSHM-164" "GSHM-163" "GSHM-162" "GSHM-161" "GSHM-160" "GSHM-159" "GSHM-158"
      aKO1
"GSHM-157"

$`BTHA1249661-WGS`$`TUSHM-822`
   gammaKO2    alphaKO2        bKO2        cKO2        aKO2  epsilonKO2
"GSHM-5034" "GSHM-5033" "GSHM-5032" "GSHM-5031" "GSHM-5030" "GSHM-5027"
    betaKO2
"GSHM-5026"


$PMAR123214
$PMAR123214$`TUIZP-94`
   betaKO1    gammaKO   alphaKO1    deltaKO       bKO2       bKO1
"GIZP-184" "GIZP-183" "GIZP-182" "GIZP-181" "GIZP-180" "GIZP-179"

$PMAR123214$`TUIZP-642`
       aKO2        cKO2        bKO3    alphaKO2     betaKO2  epsilonKO2
"GIZP-1699" "GIZP-1698" "GIZP-1697" "GIZP-1696" "GIZP-1694" "GIZP-1693"

$PMAR123214$`TUIZP-415`
 epsilonKO1
"GIZP-1049"

$PMAR123214$`TUIZP-98`
      cKO1       aKO1
"GIZP-193" "GIZP-191"


$PMEN399739
$PMEN399739$`TUHR6-2540`
        aKO         cKO         bKO     deltaKO     alphaKO     gammaKO
"GHR6-4693" "GHR6-4692" "GHR6-4691" "GHR6-4690" "GHR6-4689" "GHR6-4688"
     betaKO   epsilonKO
"GHR6-4687" "GHR6-4686"


$PMEL553174
```

```
$PMEL553174$`TUH7V-947`
    gammaKO     alphaKO     deltaKO         bKO         cKO         aKO 
"GH7V-1487" "GH7V-1486" "GH7V-1485" "GH7V-1484" "GH7V-1483" "GH7V-1482" 
  epsilonKO      betaKO 
"GH7V-1480" "GH7V-1479" 


$LSP983544
$LSP983544$`TUHSZ-548`
    gammaKO     alphaKO     deltaKO         bKO         cKO         aKO 
"GHSZ-1089" "GHSZ-1088" "GHSZ-1087" "GHSZ-1086" "GHSZ-1085" "GHSZ-1084" 

$LSP983544$`TUHSZ-437`
    betaKO 
"GHSZ-851" 

$LSP983544$`TUHSZ-438`
 epsilonKO 
"GHSZ-852" 


$LHEL405566
$LHEL405566$`TUJEN-405`
 epsilonKO     betaKO    gammaKO    alphaKO    deltaKO        bKO        cKO 
"GJEN-761" "GJEN-760" "GJEN-759" "GJEN-758" "GJEN-757" "GJEN-756" "GJEN-755" 
       aKO 
"GJEN-754" 


$PNAP365044
$PNAP365044$`TUJ8X-676`
  alphaKO1    deltaKO       bKO1       cKO1       aKO1 
"GJ8X-253" "GJ8X-252" "GJ8X-251" "GJ8X-250" "GJ8X-249" 

$PNAP365044$`TUJ8X-1732`
    betaKO2  epsilonKO2        aKO2        cKO2        bKO2    alphaKO2 
"GJ8X-2371" "GJ8X-2370" "GJ8X-2367" "GJ8X-2366" "GJ8X-2365" "GJ8X-2364" 
   gammaKO2 
"GJ8X-2363" 

$PNAP365044$`TUJ8X-677`
epsilonKO1    betaKO1   gammaKO1 
"GJ8X-256" "GJ8X-255" "GJ8X-254" 


$PAER1089456
$PAER1089456$`TULIR-3430`
        aKO         cKO         bKO     deltaKO     alphaKO     gammaKO 
"GLIR-6349" "GLIR-6348" "GLIR-6347" "GLIR-6346" "GLIR-6345" "GLIR-6344" 
     betaKO   epsilonKO 
"GLIR-6343" "GLIR-6342" 


$PNEC452638
```

$PNEC452638$`TUI4T-6`
epsilonKO betaKO gammaKO alphaKO deltaKO bKO cKO aKO
"GI4T-23" "GI4T-22" "GI4T-21" "GI4T-20" "GI4T-19" "GI4T-18" "GI4T-17" "GI4T-16"

$PNEC312153
$PNEC312153$`TUH50-7`
epsilonKO betaKO gammaKO alphaKO deltaKO bKO cKO aKO
"GH50-27" "GH50-26" "GH50-25" "GH50-24" "GH50-23" "GH50-22" "GH50-21" "GH50-20"

$PSP296591
$PSP296591$`TUHI4-557`
alphaKO deltaKO bKO cKO aKO
"GHI4-1046" "GHI4-1045" "GHI4-1044" "GHI4-1043" "GHI4-1042"

$PSP296591$`TUHI4-558`
epsilonKO betaKO gammaKO
"GHI4-1049" "GHI4-1048" "GHI4-1047"

$ACEN574556
$ACEN574556$`TUHF2-106`
alphaKO deltaKO
"GHF2-180" "GHF2-179"

$ACEN574556$`TUHF2-287`
epsilonKO betaKO
"GHF2-521" "GHF2-520"

$ACEN574556$`TUHF2-312`
gammaKO
"GHF2-565"

$ACEN574556$`TUHF2-125`
cKO bKO2 bKO1
"GHF2-216" "GHF2-215" "GHF2-214"

$ACEN574556$`TUHF2-660`
aKO
"GHF2-218"

$PPUT931281
$PPUT931281$`TULIU-28`
bKO deltaKO alphaKO gammaKO betaKO
"GLIU-62" "GLIU-61" "GLIU-60" "GLIU-59" "GLIU-58"

$PPUT931281$`TULIU-27`
epsilonKO
"GLIU-57"

$PPUT931281$`TULIU-29`
aKO

```
"GLIU-63"

$PPUT931281$noTU
cKO
 NA


$PPRO767029
$PPRO767029$`TULIM-658`
  epsilonKO      betaKO     gammaKO     alphaKO     deltaKO         bKO
"GLIM-1414" "GLIM-1413" "GLIM-1412" "GLIM-1411" "GLIM-1410" "GLIM-1409"
        cKO         aKO
"GLIM-1408" "GLIM-1407"


$PPEN278197
$PPEN278197$`TUI4Y-623`
        aKO         cKO         bKO     deltaKO     alphaKO     gammaKO
"GI4Y-1323" "GI4Y-1322" "GI4Y-1321" "GI4Y-1320" "GI4Y-1319" "GI4Y-1318"
     betaKO   epsilonKO
"GI4Y-1317" "GI4Y-1316"


$PPUT351746
$PPUT351746$`TUI26-2841`
        aKO         cKO         bKO     deltaKO     alphaKO     gammaKO
"GI26-5393" "GI26-5392" "GI26-5391" "GI26-5390" "GI26-5389" "GI26-5388"
     betaKO   epsilonKO
"GI26-5387" "GI26-5386"


$PPUT76869
$PPUT76869$`TUIXB-4`
        aKO         cKO         bKO     deltaKO     alphaKO     gammaKO
"GIXB-5522" "GIXB-5521" "GIXB-5520" "GIXB-5519" "GIXB-5518" "GIXB-5517"
     betaKO   epsilonKO
"GIXB-5516" "GIXB-5515"


$LHON557598
$LHON557598$`TUHO5-1598`
        aKO         cKO         bKO     deltaKO     alphaKO     gammaKO
"GHO5-3066" "GHO5-3065" "GHO5-3064" "GHO5-3063" "GHO5-3062" "GHO5-3061"
     betaKO   epsilonKO
"GHO5-3060" "GHO5-3059"


$PPHA324925
$PPHA324925$`TUHBF-1487`
    alphaKO     gammaKO
"GHBF-2759" "GHBF-2758"

$PPHA324925$`TUHBF-26`
   betaKO epsilonKO
```

"GHBF-48" "GHBF-47"

$PPHA324925$`TUHBF-1591`
aKO cKO bKO deltaKO
"GHBF-2939" "GHBF-2938" "GHBF-2937" "GHBF-2936"

$PPUT231023
$PPUT231023$`TULIT-1605`
aKO cKO bKO deltaKO alphaKO gammaKO
"GLIT-2748" "GLIT-2747" "GLIT-2746" "GLIT-2745" "GLIT-2744" "GLIT-2743"
betaKO epsilonKO
"GLIT-2742" "GLIT-2741"

$PPOL886882
$PPOL886882$`TUBY1-3492`
aKO cKO bKO deltaKO alphaKO
"GBY1-5112" "GBY1-5111" "GBY1-5110" "GBY1-5109" "GBY1-5108"

$PPOL886882$`TUBY1-3491`
gammaKO betaKO epsilonKO
"GBY1-5107" "GBY1-5106" "GBY1-5105"

$PPRO694427
$PPRO694427$`TUHIQ-1618`
gammaKO alphaKO deltaKO bKO cKO aKO
"GHIQ-2507" "GHIQ-2506" "GHIQ-2505" "GHIQ-2504" "GHIQ-2503" "GHIQ-2502"
epsilonKO betaKO
"GHIQ-2500" "GHIQ-2499"

$CBOT413999
$CBOT413999$`TUJ72-117`
epsilonKO betaKO gammaKO alphaKO deltaKO bKO cKO
"GJ72-195" "GJ72-194" "GJ72-193" "GJ72-192" "GJ72-191" "GJ72-190" "GJ72-189"
aKO
"GJ72-188"

$`PPRO1124983-WGS`
$`PPRO1124983-WGS`$`TUSUA-3414`
cKO bKO deltaKO alphaKO gammaKO betaKO
"GSUA-6187" "GSUA-6186" "GSUA-6185" "GSUA-6184" "GSUA-6183" "GSUA-6182"
epsilonKO
"GSUA-6181"

$`PPRO1124983-WGS`$`TUSUA-3415`
aKO
"GSUA-6188"

$PPUT1042876

$PPUT1042876$`TUH0R-5`
aKO cKO bKO deltaKO alphaKO gammaKO
"GH0R-5217" "GH0R-5216" "GH0R-5215" "GH0R-5214" "GH0R-5213" "GH0R-5212"
betaKO epsilonKO
"GH0R-5211" "GH0R-5210"

$PPUT160488
$PPUT160488$`TUIXO-4`
aKO cKO bKO deltaKO alphaKO gammaKO
"GIXO-5515" "GIXO-5514" "GIXO-5513" "GIXO-5512" "GIXO-5511" "GIXO-5510"
betaKO epsilonKO
"GIXO-5509" "GIXO-5508"

$`PPUT1215088-WGS`
$`PPUT1215088-WGS`$`TUSSY-70`
aKO cKO bKO deltaKO alphaKO gammaKO betaKO epsilonKO
"GSSY-39" "GSSY-38" "GSSY-37" "GSSY-36" "GSSY-35" "GSSY-34" "GSSY-33" "GSSY-32"

$`PPUT1331671-WGS`
$`PPUT1331671-WGS`$`TUSSW-3412`
aKO cKO bKO deltaKO alphaKO gammaKO
"GSSW-6445" "GSSW-6444" "GSSW-6443" "GSSW-6442" "GSSW-6441" "GSSW-6440"
betaKO epsilonKO
"GSSW-6439" "GSSW-6438"

$PSP1207075
$PSP1207075$`TULIW-3078`
aKO cKO bKO deltaKO alphaKO gammaKO
"GLIW-5527" "GLIW-5526" "GLIW-5525" "GLIW-5524" "GLIW-5523" "GLIW-5522"
betaKO epsilonKO
"GLIW-5521" "GLIW-5520"

$LHEL767462
$LHEL767462$`TULF7-816`
bKO deltaKO alphaKO gammaKO betaKO epsilonKO
"GLF7-1423" "GLF7-1422" "GLF7-1421" "GLF7-1420" "GLF7-1419" "GLF7-1418"

$LHEL767462$`TULF7-817`
aKO cKO
"GLF7-1425" "GLF7-1424"

$PPUT390235
$PPUT390235$`TUHHJ-2845`
aKO cKO bKO deltaKO alphaKO gammaKO
"GHHJ-5301" "GHHJ-5300" "GHHJ-5299" "GHHJ-5298" "GHHJ-5297" "GHHJ-5296"
betaKO epsilonKO
"GHHJ-5295" "GHHJ-5294"

```
$PPUT1196325
$PPUT1196325$`TULIV-2179`
  epsilonKO      betaKO     gammaKO     alphaKO     deltaKO         bKO
"GLIV-3951" "GLIV-3950" "GLIV-3949" "GLIV-3948" "GLIV-3947" "GLIV-3946"
        cKO         aKO
"GLIV-3945" "GLIV-3944"


$PPOL349520
$PPOL349520$`TUH6J-2646`
        aKO         cKO         bKO     deltaKO     alphaKO     gammaKO
"GH6J-4442" "GH6J-4441" "GH6J-4440" "GH6J-4439" "GH6J-4438" "GH6J-4437"
     betaKO   epsilonKO
"GH6J-4436" "GH6J-4435"


$`LPNE1199191-WGS`
$`LPNE1199191-WGS`$`TUSQ0-556`
    betaKO1  epsilonKO1        aKO1        cKO1        bKO1    alphaKO1
"GSQ0-1035" "GSQ0-1034" "GSQ0-1032" "GSQ0-1031" "GSQ0-1030" "GSQ0-1029"
   gammaKO1
"GSQ0-1028"

$`LPNE1199191-WGS`$`TUSQ0-1670`
       aKO2        cKO2        bKO2     deltaKO    alphaKO2    gammaKO2
"GSQ0-3027" "GSQ0-3026" "GSQ0-3025" "GSQ0-3024" "GSQ0-3023" "GSQ0-3022"
    betaKO2  epsilonKO2
"GSQ0-3021" "GSQ0-3020"


$`PPOA1282356-WGS`
$`PPOA1282356-WGS`$`TUSSV-2288`
        aKO         cKO         bKO     deltaKO     alphaKO     gammaKO
"GSSV-4247" "GSSV-4246" "GSSV-4245" "GSSV-4244" "GSSV-4243" "GSSV-4242"
     betaKO   epsilonKO
"GSSV-4241" "GSSV-4240"


$`PAVI1170318-WGS`
$`PAVI1170318-WGS`$`TUSSN-487`
 epsilonKO     betaKO    gammaKO    alphaKO    deltaKO        bKO        cKO
"GSSN-957" "GSSN-956" "GSSN-955" "GSSN-954" "GSSN-953" "GSSN-952" "GSSN-951"
       aKO
"GSSN-950"


$`PRES1245471-WGS`
$`PRES1245471-WGS`$`TUST0-3240`
        aKO         cKO         bKO     deltaKO     alphaKO     gammaKO
"GST0-5728" "GST0-5727" "GST0-5726" "GST0-5725" "GST0-5724" "GST0-5723"
     betaKO   epsilonKO
"GST0-5722" "GST0-5721"
```

$`PSP575614-HMP`
$`PSP575614-HMP`$`TUMOT-144`
betaKO epsilonKO aKO cKO bKO deltaKO alphaKO
"GMOT-280" "GMOT-279" "GMOT-277" "GMOT-276" "GMOT-275" "GMOT-274" "GMOT-273"
gammaKO
"GMOT-272"

$`PAER1340851-WGS`
$`PAER1340851-WGS`$`TUSTA-3155`
aKO cKO bKO deltaKO alphaKO gammaKO
"GSTA-5855" "GSTA-5854" "GSTA-5853" "GSTA-5852" "GSTA-5851" "GSTA-5850"
betaKO epsilonKO
"GSTA-5849" "GSTA-5848"

$BATR720555
$BATR720555$`TUHTA-1803`
aKO cKO bKO deltaKO alphaKO gammaKO
"GHTA-3296" "GHTA-3295" "GHTA-3294" "GHTA-3293" "GHTA-3292" "GHTA-3291"
betaKO epsilonKO
"GHTA-3290" "GHTA-3289"

$BCOA345219
$BCOA345219$`TUH5A-849`
epsilonKO betaKO gammaKO alphaKO deltaKO bKO
"GH5A-1447" "GH5A-1446" "GH5A-1445" "GH5A-1444" "GH5A-1443" "GH5A-1442"
cKO aKO
"GH5A-1441" "GH5A-1440"

$LHEL880633
$LHEL880633$`TULF9-734`
aKO cKO bKO deltaKO alphaKO gammaKO
"GLF9-1363" "GLF9-1362" "GLF9-1361" "GLF9-1360" "GLF9-1359" "GLF9-1358"
betaKO epsilonKO
"GLF9-1357" "GLF9-1356"

$BANT568206
$BANT568206$`TUHVT-3415`
aKO cKO bKO deltaKO alphaKO gammaKO
"GHVT-5531" "GHVT-5530" "GHVT-5529" "GHVT-5528" "GHVT-5527" "GHVT-5526"
betaKO epsilonKO
"GHVT-5525" "GHVT-5524"

$BANT592021
$BANT592021$`TUJAQ-3421`
aKO cKO bKO deltaKO alphaKO gammaKO
"GJAQ-5522" "GJAQ-5521" "GJAQ-5520" "GJAQ-5519" "GJAQ-5518" "GJAQ-5517"
betaKO epsilonKO

"GJAQ-5516" "GJAQ-5515"

$HPYL693745
$HPYL693745$`TUJAE-140`
epsilonKO betaKO gammaKO alphaKO deltaKO bKO2 bKO1
"GJAE-377" "GJAE-376" "GJAE-375" "GJAE-374" "GJAE-373" "GJAE-372" "GJAE-371"

$HPYL693745$`TUJAE-93`
cKO
"GJAE-274"

$HPYL693745$`TUJAE-441`
aKO
"GJAE-1059"

$CCON360104
$CCON360104$`TUHAC-228`
epsilonKO betaKO gammaKO alphaKO deltaKO bKO2 bKO1
"GHAC-473" "GHAC-472" "GHAC-471" "GHAC-470" "GHAC-469" "GHAC-468" "GHAC-467"

$CCON360104$`TUHAC-245`
cKO
"GHAC-511"

$CCON360104$`TUHAC-406`
aKO
"GHAC-898"

$PRUM264731
$PRUM264731$`TUHX6-584`
betaKO epsilonKO aKO cKO bKO deltaKO
"GHX6-1153" "GHX6-1152" "GHX6-1150" "GHX6-1149" "GHX6-1148" "GHX6-1147"
alphaKO gammaKO
"GHX6-1146" "GHX6-1145"

$PPRW349106
$PPRW349106$`TUHZF-159`
epsilonKO betaKO gammaKO alphaKO deltaKO bKO cKO
"GHZF-198" "GHZF-197" "GHZF-196" "GHZF-195" "GHZF-194" "GHZF-193" "GHZF-192"
aKO
"GHZF-191"

$PSTU379731
$PSTU379731$`TUJER-2355`
aKO cKO bKO deltaKO alphaKO gammaKO
"GJER-4192" "GJER-4191" "GJER-4190" "GJER-4189" "GJER-4188" "GJER-4187"
betaKO epsilonKO
"GJER-4186" "GJER-4185"

$PSYR205918
$PSYR205918$`TUJ94-2809`
aKO cKO bKO deltaKO alphaKO gammaKO
"GJ94-5210" "GJ94-5209" "GJ94-5208" "GJ94-5207" "GJ94-5206" "GJ94-5205"
betaKO epsilonKO
"GJ94-5204" "GJ94-5203"

$PSTU1196835
$PSTU1196835$`TULIX-6`
aKO cKO bKO deltaKO alphaKO gammaKO
"GLIX-4366" "GLIX-4365" "GLIX-4364" "GLIX-4363" "GLIX-4362" "GLIX-4361"
betaKO epsilonKO
"GLIX-4360" "GLIX-4359"

$PSPA1045855
$PSPA1045855$`TUH5V-1582`
aKO cKO bKO deltaKO alphaKO gammaKO
"GH5V-2788" "GH5V-2787" "GH5V-2786" "GH5V-2785" "GH5V-2784" "GH5V-2783"
betaKO epsilonKO
"GH5V-2782" "GH5V-2781"

$PSP82654
$PSP82654$`TULIO-1835`
alphaKO deltaKO bKO2 bKO1 cKO aKO
"GLIO-2331" "GLIO-2330" "GLIO-2329" "GLIO-2328" "GLIO-2327" "GLIO-2326"

$PSP82654$`TULIO-824`
epsilonKO betaKO
"GLIO-893" "GLIO-892"

$PSP82654$`TULIO-1314`
gammaKO
"GLIO-1599"

$PSP911045
$PSP911045$`TUJTQ-648`
epsilonKO betaKO gammaKO alphaKO deltaKO
"GJTQ-638" "GJTQ-637" "GJTQ-636" "GJTQ-635" "GJTQ-634"

$PSP911045$`TUJTQ-1228`
bKO2 bKO1 cKO aKO
"GJTQ-1620" "GJTQ-1619" "GJTQ-1618" "GJTQ-1617"

$`LHEL326425-WGS`
$`LHEL326425-WGS`$`TUSP0-451`
epsilonKO betaKO gammaKO alphaKO deltaKO bKO cKO
"GSP0-779" "GSP0-778" "GSP0-777" "GSP0-776" "GSP0-775" "GSP0-774" "GSP0-773"
aKO

"GSP0-772"

$`PAER1280938-WGS`
$`PAER1280938-WGS`$`TUSSP-3187`
aKO cKO bKO deltaKO alphaKO gammaKO
"GSSP-5894" "GSSP-5893" "GSSP-5892" "GSSP-5891" "GSSP-5890" "GSSP-5889"
betaKO epsilonKO
"GSSP-5888" "GSSP-5887"

$CCUR360105
$CCUR360105$`TUJ9P-654`
bKO1 bKO2 deltaKO alphaKO gammaKO betaKO
"GJ9P-1661" "GJ9P-1660" "GJ9P-1659" "GJ9P-1658" "GJ9P-1657" "GJ9P-1656"
epsilonKO
"GJ9P-1655"

$CCUR360105$`TUJ9P-222`
cKO
"GJ9P-567"

$CCUR360105$`TUJ9P-470`
aKO
"GJ9P-1158"

$`PSTU644801-WGS`
$`PSTU644801-WGS`$`TUSTN-2389`
aKO cKO bKO deltaKO alphaKO gammaKO
"GSTN-4351" "GSTN-4350" "GSTN-4349" "GSTN-4348" "GSTN-4347" "GSTN-4346"
betaKO epsilonKO
"GSTN-4345" "GSTN-4344"

$PSTU1157951
$PSTU1157951$`TULIN-1146`
epsilonKO betaKO gammaKO alphaKO deltaKO bKO
"GLIN-2056" "GLIN-2055" "GLIN-2054" "GLIN-2053" "GLIN-2052" "GLIN-2051"
cKO aKO
"GLIN-2050" "GLIN-2049"

$PSTU1123519
$PSTU1123519$`TULIY-2150`
aKO cKO bKO deltaKO alphaKO gammaKO
"GLIY-3878" "GLIY-3877" "GLIY-3876" "GLIY-3875" "GLIY-3874" "GLIY-3873"
betaKO epsilonKO
"GLIY-3872" "GLIY-3871"

$PSTA530564
$PSTA530564$`TUHPR-3186`
epsilonKO betaKO gammaKO alphaKO deltaKO cKO

```
"GHPR-4461" "GHPR-4460" "GHPR-4459" "GHPR-4457" "GHPR-4456" "GHPR-4454"

$PSTA530564$noTU
aKO bKO
 NA  NA


$PSP234831
$PSP234831$`TUH93-2033`
        aKO         cKO         bKO     deltaKO     alphaKO     gammaKO
"GH93-3165" "GH93-3164" "GH93-3163" "GH93-3162" "GH93-3161" "GH93-3160"
     betaKO   epsilonKO
"GH93-3159" "GH93-3158"


$PSAL762903
$PSAL762903$`TUHB2-1357`
        aKO         cKO         bKO     deltaKO     alphaKO     gammaKO
"GHB2-2558" "GHB2-2557" "GHB2-2556" "GHB2-2555" "GHB2-2554" "GHB2-2553"

$PSAL762903$`TUHB2-1264`
  epsilonKO      betaKO
"GHB2-2394" "GHB2-2393"


$PSAV264730
$PSAV264730$`TUKDE-2915`
        aKO         cKO         bKO     deltaKO     alphaKO     gammaKO
"GKDE-5218" "GKDE-5217" "GKDE-5216" "GKDE-5215" "GKDE-5214" "GKDE-5213"
     betaKO   epsilonKO
"GKDE-5212" "GKDE-5211"


$PSTU996285
$PSTU996285$`TULIZ-2390`
        aKO         cKO         bKO     deltaKO     alphaKO     gammaKO
"GLIZ-4356" "GLIZ-4355" "GLIZ-4354" "GLIZ-4353" "GLIZ-4352" "GLIZ-4351"
     betaKO   epsilonKO
"GLIZ-4350" "GLIZ-4349"


$PSYR223283
$PSYR223283$`TUJIX-3168`
        aKO         cKO         bKO     deltaKO     alphaKO     gammaKO
"GJIX-5678" "GJIX-5677" "GJIX-5676" "GJIX-5675" "GJIX-5674" "GJIX-5673"
     betaKO   epsilonKO
"GJIX-5672" "GJIX-5671"


$LINT267671
$LINT267671$`TUHQI-727`
  epsilonKO      betaKO     gammaKO     alphaKO     deltaKO         bKO
"GHQI-1242" "GHQI-1241" "GHQI-1240" "GHQI-1239" "GHQI-1238" "GHQI-1237"
        cKO         aKO
```

"GHQI-1236" "GHQI-1235"

$CEFF196164
$CEFF196164$`TUJW8-733`
epsilonKO betaKO gammaKO alphaKO deltaKO bKO
"GJW8-1336" "GJW8-1335" "GJW8-1334" "GJW8-1333" "GJW8-1332" "GJW8-1331"
cKO aKO
"GJW8-1330" "GJW8-1329"

$PSUW743721
$PSUW743721$`TUH68-188`
epsilonKO betaKO gammaKO alphaKO deltaKO bKO cKO
"GH68-321" "GH68-320" "GH68-319" "GH68-318" "GH68-317" "GH68-316" "GH68-315"
aKO
"GH68-314"

$PSTU96563
$PSTU96563$`TUHIN-2345`
aKO cKO bKO deltaKO alphaKO gammaKO
"GHIN-4297" "GHIN-4296" "GHIN-4295" "GHIN-4294" "GHIN-4293" "GHIN-4292"
betaKO epsilonKO
"GHIN-4291" "GHIN-4290"

$PTER985665
$PTER985665$`TUHT3-555`
aKO cKO bKO deltaKO alphaKO
"GHT3-987" "GHT3-986" "GHT3-985" "GHT3-984" "GHT3-983"

$PTER985665$`TUHT3-554`
gammaKO betaKO epsilonKO
"GHT3-981" "GHT3-980" "GHT3-979"

$PTHE370438
$PTHE370438$`TUCGQ-1632`
bKO deltaKO alphaKO gammaKO betaKO epsilonKO
"GCGQ-2871" "GCGQ-2870" "GCGQ-2869" "GCGQ-2868" "GCGQ-2867" "GCGQ-2866"

$PTHE370438$`TUCGQ-1633`
cKO
"GCGQ-2872"

$PTHE370438$`TUCGQ-1634`
aKO
"GCGQ-2873"

$PTOR313595
$PTOR313595$`TULJ0-1087`
gammaKO alphaKO deltaKO bKO cKO aKO

"GLJ0-1938" "GLJ0-1937" "GLJ0-1936" "GLJ0-1935" "GLJ0-1934" "GLJ0-1933"

$PTOR313595$`TULJ0-818`
betaKO epsilonKO
"GLJ0-1496" "GLJ0-1495"

$CPEL335992
$CPEL335992$`TUH3Z-81`
deltaKO alphaKO gammaKO betaKO epsilonKO
"GH3Z-232" "GH3Z-231" "GH3Z-230" "GH3Z-229" "GH3Z-228"

$CPEL335992$`TUH3Z-48`
bKO2 bKO1 cKO aKO
"GH3Z-121" "GH3Z-120" "GH3Z-119" "GH3Z-118"

$PMUL584721
$PMUL584721$`TULI9-921`
epsilonKO betaKO gammaKO alphaKO deltaKO bKO
"GLI9-1810" "GLI9-1809" "GLI9-1808" "GLI9-1807" "GLI9-1806" "GLI9-1805"
cKO aKO
"GLI9-1804" "GLI9-1803"

$PSP1007105
$PSP1007105$`TUJAF-1278`
bKO deltaKO alphaKO gammaKO betaKO epsilonKO
"GJAF-2478" "GJAF-2477" "GJAF-2476" "GJAF-2475" "GJAF-2474" "GJAF-2473"

$PSP1007105$`TUJAF-1279`
cKO
"GJAF-2480"

$PSP1007105$`TUJAF-1280`
aKO
"GJAF-2481"

$PACA765952
$PACA765952$`TUH3O-1270`
aKO cKO bKO deltaKO alphaKO gammaKO
"GH3O-2410" "GH3O-2409" "GH3O-2408" "GH3O-2407" "GH3O-2406" "GH3O-2405"
betaKO epsilonKO
"GH3O-2404" "GH3O-2403"

$PVAG712898
$PVAG712898$`TUHQ2-2226`
epsilonKO betaKO gammaKO alphaKO deltaKO bKO
"GHQ2-3256" "GHQ2-3255" "GHQ2-3254" "GHQ2-3253" "GHQ2-3252" "GHQ2-3251"
cKO aKO
"GHQ2-3250" "GHQ2-3249"

$CJEJ645464
$CJEJ645464$`TULA6-51`
epsilonKO betaKO gammaKO alphaKO deltaKO bKO2 bKO1
"GLA6-106" "GLA6-105" "GLA6-104" "GLA6-103" "GLA6-102" "GLA6-101" "GLA6-100"

$CJEJ645464$`TULA6-348`
cKO
"GLA6-896"

$CJEJ645464$`TULA6-447`
aKO
"GLA6-1180"

$LINT573825
$LINT573825$`TULFK-1218`
aKO cKO bKO deltaKO alphaKO gammaKO
"GLFK-2263" "GLFK-2262" "GLFK-2261" "GLFK-2260" "GLFK-2259" "GLFK-2258"
betaKO epsilonKO
"GLFK-2257" "GLFK-2256"

$CPHA290318
$CPHA290318$`TUHNQ-847`
alphaKO
"GHNQ-1676"

$CPHA290318$`TUHNQ-15`
betaKO epsilonKO
"GHNQ-25" "GHNQ-24"

$CPHA290318$`TUHNQ-846`
gammaKO
"GHNQ-1675"

$CPHA290318$`TUHNQ-903`
bKO deltaKO
"GHNQ-1791" "GHNQ-1790"

$CPHA290318$`TUHNQ-904`
cKO
"GHNQ-1792"

$CPHA290318$`TUHNQ-905`
aKO
"GHNQ-1793"

$PWAS561231
$PWAS561231$`TUHO0-2593`
epsilonKO betaKO gammaKO alphaKO deltaKO bKO
"GHO0-4648" "GHO0-4647" "GHO0-4646" "GHO0-4645" "GHO0-4644" "GHO0-4643"
cKO aKO

```
"GHO0-4642" "GHO0-4641"


$RAQU1151116
$RAQU1151116$`TULJ4-2809`
  epsilonKO      betaKO     gammaKO     alphaKO     deltaKO         bKO
"GLJ4-4519" "GLJ4-4518" "GLJ4-4517" "GLJ4-4516" "GLJ4-4515" "GLJ4-4514"
        cKO         aKO
"GLJ4-4513" "GLJ4-4512"


$`RANA1271752-WGS`
$`RANA1271752-WGS`$`TUSTW-695`
    gammaKO     alphaKO     deltaKO         bKO         cKO
"GSTW-1248" "GSTW-1247" "GSTW-1246" "GSTW-1245" "GSTW-1244"

$`RANA1271752-WGS`$`TUSTW-1110`
     betaKO   epsilonKO
"GSTW-1929" "GSTW-1928"

$`RANA1271752-WGS`$`TUSTW-694`
        aKO
"GSTW-1243"


$RAFR347255
$RAFR347255$`TUJCT-695`
    alphaKO
"GJCT-1043"

$RAFR347255$`TUJCT-693`
     betaKO
"GJCT-1041"

$RAFR347255$`TUJCT-694`
    gammaKO
"GJCT-1042"

$RAFR347255$`TUJCT-696`
    deltaKO
"GJCT-1044"

$RAFR347255$`TUJCT-692`
  epsilonKO
"GJCT-1040"

$RAFR347255$`TUJCT-21`
      cKO      bKO2      bKO1
"GJCT-25" "GJCT-24" "GJCT-23"

$RAFR347255$`TUJCT-22`
      aKO
"GJCT-26"
```

```
$`RANA1228997-WGS`
$`RANA1228997-WGS`$`TUSTU-926`
    gammaKO     alphaKO     deltaKO         bKO         cKO 
"GSTU-1634" "GSTU-1633" "GSTU-1632" "GSTU-1631" "GSTU-1630" 

$`RANA1228997-WGS`$`TUSTU-90`
    betaKO  epsilonKO 
"GSTU-155" "GSTU-154" 

$`RANA1228997-WGS`$`TUSTU-925`
        aKO 
"GSTU-1629" 


$RSP741091
$RSP741091$`TUHHP-2801`
  epsilonKO      betaKO     gammaKO     alphaKO     deltaKO         bKO 
"GHHP-4520" "GHHP-4519" "GHHP-4518" "GHHP-4517" "GHHP-4516" "GHHP-4515" 
        cKO         aKO 
"GHHP-4514" "GHHP-4513" 


$RAKA293614
$RAKA293614$`TUI4A-758`
    deltaKO     alphaKO     gammaKO      betaKO   epsilonKO 
"GI4A-1166" "GI4A-1165" "GI4A-1164" "GI4A-1163" "GI4A-1162" 

$RAKA293614$`TUI4A-28`
     bKO2      bKO1       cKO       aKO 
"GI4A-49" "GI4A-48" "GI4A-47" "GI4A-46" 


$RALB697329
$RALB697329$`TUIWQ-569`
       aKO        cKO        bKO    deltaKO    alphaKO    gammaKO     betaKO 
"GIWQ-303" "GIWQ-302" "GIWQ-301" "GIWQ-300" "GIWQ-299" "GIWQ-298" "GIWQ-297" 
 epsilonKO 
"GIWQ-296" 


$CSP1042156
$CSP1042156$`TUHDR-630`
  epsilonKO      betaKO     gammaKO     alphaKO     deltaKO        bKO2 
"GHDR-1180" "GHDR-1179" "GHDR-1178" "GHDR-1177" "GHDR-1176" "GHDR-1175" 
        cKO 
"GHDR-1174" 

$CSP1042156$`TUHDR-629`
        aKO 
"GHDR-1173" 

$CSP1042156$`TUHDR-7`
     bKO1 
```

```
"GHDR-18"


$RAMB1105111
$RAMB1105111$`TULAJ-934`
   deltaKO     alphaKO     gammaKO      betaKO   epsilonKO 
"GLAJ-1304" "GLAJ-1303" "GLAJ-1302" "GLAJ-1301" "GLAJ-1300" 

$RAMB1105111$`TULAJ-151`
     bKO2       bKO1        cKO        aKO 
"GLAJ-130" "GLAJ-129" "GLAJ-128" "GLAJ-127" 


$LINT189518
$LINT189518$`TUJBB-1219`
       aKO         cKO         bKO     deltaKO     alphaKO     gammaKO 
"GJBB-2262" "GJBB-2261" "GJBB-2260" "GJBB-2259" "GJBB-2258" "GJBB-2257" 
    betaKO   epsilonKO 
"GJBB-2256" "GJBB-2255" 


$`BAPH713601-WGS`
$`BAPH713601-WGS`$`TUSHF-2`
epsilonKO    betaKO   gammaKO   alphaKO   deltaKO       bKO       cKO       aKO 
 "GSHF-9"  "GSHF-8"  "GSHF-7"  "GSHF-6"  "GSHF-5"  "GSHF-4"  "GSHF-3"  "GSHF-2" 


$BAPH1005090
$BAPH1005090$`TUL9S-6`
epsilonKO    betaKO   gammaKO   alphaKO   deltaKO       bKO       cKO       aKO 
 "GL9S-9"  "GL9S-8"  "GL9S-7"  "GL9S-6"  "GL9S-5"  "GL9S-4"  "GL9S-3"  "GL9S-2" 


$BCER637380
$BCER637380$`TUHO7-3258`
       aKO         cKO         bKO     deltaKO     alphaKO 
"GHO7-5200" "GHO7-5199" "GHO7-5198" "GHO7-5197" "GHO7-5196" 

$BCER637380$`TUHO7-3257`
   gammaKO      betaKO   epsilonKO 
"GHO7-5194" "GHO7-5193" "GHO7-5192" 


$BAMB339670
$BAMB339670$`TUH48-67`
 epsilonKO     betaKO    gammaKO    alphaKO    deltaKO        bKO        cKO 
"GH48-100"  "GH48-99"  "GH48-98"  "GH48-97"  "GH48-96"  "GH48-95"  "GH48-94" 
       aKO 
 "GH48-93" 


$RANA693978
$RANA693978$`TUHF6-240`
      bKO    deltaKO    alphaKO 
```

"GHF6-422" "GHF6-421" "GHF6-420"

$RANA693978$`TUHF6-919`
betaKO
"GHF6-1753"

$RANA693978$`TUHF6-239`
gammaKO
"GHF6-419"

$RANA693978$`TUHF6-920`
epsilonKO
"GHF6-1754"

$RANA693978$`TUHF6-241`
cKO
"GHF6-423"

$RANA693978$`TUHF6-242`
aKO
"GHF6-424"

$`RAQU745277-WGS`
$`RAQU745277-WGS`$`TUST8-2728`
epsilonKO betaKO gammaKO alphaKO deltaKO bKO
"GST8-4533" "GST8-4532" "GST8-4531" "GST8-4530" "GST8-4529" "GST8-4528"
cKO aKO
"GST8-4527" "GST8-4526"

$RANA992406
$RANA992406$`TULJP-963`
alphaKO deltaKO bKO
"GLJP-1807" "GLJP-1806" "GLJP-1805"

$RANA992406$`TULJP-261`
betaKO
"GLJP-460"

$RANA992406$`TULJP-964`
gammaKO
"GLJP-1808"

$RANA992406$`TULJP-260`
epsilonKO
"GLJP-459"

$RANA992406$`TULJP-962`
cKO
"GLJP-1804"

$RANA992406$`TULJP-961`
aKO

"GLJP-1803"

$RAUS1105110
$RAUS1105110$`TULJ8-199`
epsilonKO betaKO gammaKO alphaKO deltaKO
"GLJ8-283" "GLJ8-282" "GLJ8-281" "GLJ8-280" "GLJ8-279"

$RAUS1105110$`TULJ8-44`
cKO bKO2 bKO1
"GLJ8-41" "GLJ8-40" "GLJ8-39"

$RAUS1105110$`TULJ8-45`
aKO
"GLJ8-43"

$CNOV386415
$CNOV386415$`TUH98-1122`
aKO cKO bKO deltaKO alphaKO gammaKO
"GH98-2100" "GH98-2099" "GH98-2098" "GH98-2097" "GH98-2096" "GH98-2095"
betaKO epsilonKO
"GH98-2094" "GH98-2093"

$RBEL336407
$RBEL336407$`TUJCY-56`
alphaKO
"GJCY-96"

$RBEL336407$`TUJCY-57`
epsilonKO betaKO gammaKO
"GJCY-99" "GJCY-98" "GJCY-97"

$RBEL336407$`TUJCY-55`
deltaKO
"GJCY-94"

$RBEL336407$`TUJCY-790`
bKO2 bKO1 cKO aKO
"GJCY-1331" "GJCY-1330" "GJCY-1329" "GJCY-1328"

$RBIF313596
$RBIF313596$`TUH7G-387`
aKO cKO bKO deltaKO alphaKO gammaKO
"GH7G-660" "GH7G-659" "GH7G-658" "GH7G-657" "GH7G-656" "GH7G-655"

$RBIF313596$`TUH7G-561`
epsilonKO betaKO
"GH7G-951" "GH7G-950"

$`LINN272626-WGS`

```
$`LINN272626-WGS`$`TUSPN-104`
epsilonKO1    betaKO1   gammaKO1   alphaKO1   deltaKO1       cKO1
"GSPN-140" "GSPN-139" "GSPN-138" "GSPN-137" "GSPN-136" "GSPN-135"

$`LINN272626-WGS`$`TUSPN-1407`
        aKO        cKO2         bKO    deltaKO2    alphaKO2    gammaKO2
"GSPN-2748" "GSPN-2747" "GSPN-2746" "GSPN-2745" "GSPN-2744" "GSPN-2743"
    betaKO2  epsilonKO2
"GSPN-2742" "GSPN-2741"



$RCAS383372
$RCAS383372$`TUH89-737`
        aKO         cKO         bKO     deltaKO     alphaKO     gammaKO
"GH89-1287" "GH89-1286" "GH89-1285" "GH89-1284" "GH89-1283" "GH89-1282"
     betaKO   epsilonKO
"GH89-1281" "GH89-1280"



$RCAN1105107
$RCAN1105107$`TUJVR-618`
   deltaKO    alphaKO    gammaKO     betaKO  epsilonKO
"GJVR-947" "GJVR-946" "GJVR-945" "GJVR-944" "GJVR-943"

$RCAN1105107$`TUJVR-11`
      aKO       cKO      bKO2      bKO1
"GJVR-21" "GJVR-20" "GJVR-19" "GJVR-18"



$`RCHA213810-WGS`
$`RCHA213810-WGS`$`TUSU3-360`
 epsilonKO     betaKO    gammaKO    alphaKO    deltaKO        bKO        cKO
"GSU3-654" "GSU3-653" "GSU3-652" "GSU3-651" "GSU3-650" "GSU3-649" "GSU3-648"
       aKO
"GSU3-647"



$RCAN293613
$RCAN293613$`TUHI3-655`
    deltaKO     alphaKO     gammaKO      betaKO   epsilonKO
"GHI3-1016" "GHI3-1015" "GHI3-1014" "GHI3-1013" "GHI3-1012"

$RCAN293613$`TUHI3-12`
      aKO       cKO      bKO2      bKO1
"GHI3-22" "GHI3-21" "GHI3-20" "GHI3-19"



$`RCON272944-WGS`
$`RCON272944-WGS`$`TUSTQ-811`
    deltaKO     alphaKO     gammaKO      betaKO   epsilonKO
"GSTQ-1274" "GSTQ-1273" "GSTQ-1272" "GSTQ-1271" "GSTQ-1270"

$`RCON272944-WGS`$`TUSTQ-16`
      aKO       cKO      bKO2      bKO1
```

```
"GSTQ-29" "GSTQ-28" "GSTQ-27" "GSTQ-26"


$RCAP272942
$RCAP272942$`TUJIY-1560`
   deltaKO     alphaKO     gammaKO      betaKO   epsilonKO
"GJIY-3014" "GJIY-3013" "GJIY-3012" "GJIY-3011" "GJIY-3010"

$RCAP272942$`TUJIY-444`
     bKO2       bKO1        cKO        aKO
"GJIY-757" "GJIY-756" "GJIY-755" "GJIY-754"


$RDEN375451
$RDEN375451$`TUJIZ-1782`
   deltaKO     alphaKO     gammaKO      betaKO   epsilonKO
"GJIZ-3332" "GJIZ-3331" "GJIZ-3330" "GJIZ-3329" "GJIZ-3328"

$RDEN375451$`TUJIZ-704`
      bKO2        bKO1         cKO         aKO
"GJIZ-1246" "GJIZ-1245" "GJIZ-1244" "GJIZ-1243"


$CSTI499177
$CSTI499177$`TUJE9-1004`
       aKO         cKO         bKO     deltaKO     alphaKO     gammaKO
"GJE9-2187" "GJE9-2186" "GJE9-2185" "GJE9-2184" "GJE9-2183" "GJE9-2182"
    betaKO   epsilonKO
"GJE9-2181" "GJE9-2180"


$RDEN762948
$RDEN762948$`TUHPW-1140`
  epsilonKO      betaKO     gammaKO     alphaKO     deltaKO         bKO
"GHPW-1772" "GHPW-1771" "GHPW-1770" "GHPW-1769" "GHPW-1768" "GHPW-1767"
        cKO         aKO
"GHPW-1766" "GHPW-1765"


$RETL491916
$RETL491916$`TUH4T-3343`
   deltaKO     alphaKO     gammaKO      betaKO   epsilonKO
"GH4T-4153" "GH4T-4152" "GH4T-4151" "GH4T-4150" "GH4T-4149"

$RETL491916$`TUH4T-1478`
     bKO2       bKO1        cKO        aKO
"GH4T-956" "GH4T-955" "GH4T-954" "GH4T-953"


$CNEC381666
$CNEC381666$`TUJUJ-2292`
       aKO         cKO         bKO     deltaKO     alphaKO     gammaKO
"GJUJ-3607" "GJUJ-3606" "GJUJ-3605" "GJUJ-3604" "GJUJ-3603" "GJUJ-3602"
    betaKO   epsilonKO
```

"GJUJ-3601" "GJUJ-3600"

$`LINT1234378-WGS`
$`LINT1234378-WGS`$`TUSPF-361`
 epsilonKO betaKO gammaKO alphaKO deltaKO bKO2 bKO1
"GSPF-431" "GSPF-430" "GSPF-429" "GSPF-428" "GSPF-427" "GSPF-426" "GSPF-425"

$`LINT1234378-WGS`$`TUSPF-791`
 cKO aKO
"GSPF-1140" "GSPF-1139"

$`RETL1328306-WGS`
$`RETL1328306-WGS`$`TUSTH-3640`
 deltaKO alphaKO gammaKO betaKO epsilonKO
"GSTH-3960" "GSTH-3959" "GSTH-3958" "GSTH-3957" "GSTH-3955"

$`RETL1328306-WGS`$`TUSTH-1877`
 bKO2 bKO1 cKO aKO
"GSTH-881" "GSTH-880" "GSTH-879" "GSTH-878"

$REQU685727
$REQU685727$`TUHKP-879`
 epsilonKO betaKO gammaKO alphaKO deltaKO bKO
"GHKP-1694" "GHKP-1693" "GHKP-1692" "GHKP-1691" "GHKP-1690" "GHKP-1689"
 cKO aKO
"GHKP-1688" "GHKP-1687"

$RERY234621
$RERY234621$`TUHDE-2175`
 aKO cKO bKO deltaKO alphaKO gammaKO
"GHDE-3967" "GHDE-3966" "GHDE-3965" "GHDE-3964" "GHDE-3963" "GHDE-3962"
 betaKO epsilonKO
"GHDE-3961" "GHDE-3960"

$RETL347834
$RETL347834$`TUJJ0-3457`
 deltaKO alphaKO gammaKO betaKO epsilonKO
"GJJ0-3891" "GJJ0-3890" "GJJ0-3889" "GJJ0-3888" "GJJ0-3887"

$RETL347834$`TUJJ0-1649`
 bKO2 bKO1 cKO aKO
"GJJ0-876" "GJJ0-875" "GJJ0-874" "GJJ0-873"

$CPIN264198
$CPIN264198$`TUIW3-2306`
 aKO cKO bKO deltaKO alphaKO gammaKO
"GIW3-3417" "GIW3-3416" "GIW3-3415" "GIW3-3414" "GIW3-3413" "GIW3-3412"
 betaKO epsilonKO

```
"GIW3-3411" "GIW3-3410"


$`RERY1136179-WGS`
$`RERY1136179-WGS`$`TUSVJ-1867`
      aKO         cKO         bKO     deltaKO     alphaKO     gammaKO
"GSVJ-3627" "GSVJ-3626" "GSVJ-3625" "GSVJ-3624" "GSVJ-3623" "GSVJ-3622"
    betaKO   epsilonKO
"GSVJ-3621" "GSVJ-3620"


$ASP861208
$ASP861208$`TUH59-1843`
   deltaKO     alphaKO     gammaKO      betaKO   epsilonKO
"GH59-2588" "GH59-2587" "GH59-2586" "GH59-2585" "GH59-2584"

$ASP861208$`TUH59-722`
     bKO2       bKO1        cKO        aKO
"GH59-664" "GH59-663" "GH59-662" "GH59-661"


$RFEL315456
$RFEL315456$`TUKEK-925`
   deltaKO     alphaKO     gammaKO      betaKO   epsilonKO
"GKEK-1304" "GKEK-1303" "GKEK-1302" "GKEK-1301" "GKEK-1300"

$RFEL315456$`TUKEK-91`
     aKO       cKO      bKO2      bKO1
"GKEK-32" "GKEK-31" "GKEK-30" "GKEK-29"


$AFER338969
$AFER338969$`TUHU9-186`
     aKO1       cKO1       bKO1    deltaKO   alphaKO1   gammaKO1    betaKO1
"GHU9-112" "GHU9-111" "GHU9-110" "GHU9-109" "GHU9-108" "GHU9-107" "GHU9-106"
epsilonKO1
"GHU9-105"

$AFER338969$`TUHU9-711`
  gammaKO2    alphaKO2        bKO2        cKO2        aKO2  epsilonKO2
"GHU9-1172" "GHU9-1171" "GHU9-1170" "GHU9-1169" "GHU9-1168" "GHU9-1165"
   betaKO2
"GHU9-1164"


$RGEL983917
$RGEL983917$`TULJR-108`
      aKO        cKO        bKO    deltaKO    alphaKO    gammaKO     betaKO
"GLJR-230" "GLJR-229" "GLJR-228" "GLJR-227" "GLJR-226" "GLJR-225" "GLJR-224"
 epsilonKO
"GLJR-223"


$RJOS101510
```

```
$RJOS101510$`TUJJ1-1896`
       aKO         cKO         bKO     deltaKO     alphaKO     gammaKO 
"GJJ1-1477" "GJJ1-1476" "GJJ1-1475" "GJJ1-1474" "GJJ1-1473" "GJJ1-1472" 
     betaKO   epsilonKO 
"GJJ1-1471" "GJJ1-1470" 


$`CLIB1261131-WGS`
$`CLIB1261131-WGS`$`TUSIH-648`
    deltaKO     alphaKO     gammaKO      betaKO   epsilonKO 
"GSIH-1038" "GSIH-1037" "GSIH-1036" "GSIH-1035" "GSIH-1034" 

$`CLIB1261131-WGS`$`TUSIH-341`
      bKO2       bKO1        cKO        aKO 
"GSIH-520" "GSIH-519" "GSIH-518" "GSIH-517" 


$APHA212042
$APHA212042$`TUHPM-882`
    deltaKO     alphaKO 
"GHPM-1335" "GHPM-1334" 

$APHA212042$`TUHPM-296`
    betaKO  epsilonKO 
"GHPM-494" "GHPM-493" 

$APHA212042$`TUHPM-439`
  gammaKO1 
"GHPM-707" 

$APHA212042$`TUHPM-511`
  gammaKO2 
"GHPM-813" 

$APHA212042$`TUHPM-786`
       aKO         cKO        bKO2        bKO1 
"GHPM-1192" "GHPM-1191" "GHPM-1190" "GHPM-1189" 


$`RDEN666685-WGS`
$`RDEN666685-WGS`$`TUSTL-2038`
       aKO         cKO         bKO     deltaKO     alphaKO     gammaKO 
"GSTL-3775" "GSTL-3774" "GSTL-3773" "GSTL-3772" "GSTL-3771" "GSTL-3770" 
     betaKO   epsilonKO 
"GSTL-3769" "GSTL-3768" 


$RHEI1032845
$RHEI1032845$`TUI4V-785`
    deltaKO     alphaKO     gammaKO      betaKO   epsilonKO 
"GI4V-1206" "GI4V-1205" "GI4V-1204" "GI4V-1203" "GI4V-1202" 

$RHEI1032845$`TUI4V-23`
      aKO       cKO      bKO2      bKO1 
```

```
"GI4V-33" "GI4V-32" "GI4V-31" "GI4V-30"


$SFRE394
$SFRE394$`TUBYN-3310`
    deltaKO     alphaKO     gammaKO      betaKO
"GBYN-3102" "GBYN-3101" "GBYN-3100" "GBYN-3099"

$SFRE394$`TUBYN-3309`
  epsilonKO
"GBYN-3098"

$SFRE394$`TUBYN-1794`
      bKO2       bKO1        cKO        aKO
"GBYN-449" "GBYN-448" "GBYN-447" "GBYN-446"


$`RINT657315-WGS`
$`RINT657315-WGS`$`TUSTX-1626`
 epsilonKO2     betaKO2    gammaKO2     alphaKO    deltaKO2        cKO2
"GSTX-2909" "GSTX-2908" "GSTX-2907" "GSTX-2906" "GSTX-2905" "GSTX-2904"
        aKO
"GSTX-2903"

$`RINT657315-WGS`$`TUSTX-53`
epsilonKO1    betaKO1   gammaKO1   deltaKO1        bKO       cKO1
 "GSTX-96"  "GSTX-95"  "GSTX-94"  "GSTX-93"  "GSTX-92"  "GSTX-91"


$DMAG573370
$DMAG573370$`TUHJL-318`
epsilonKO2 epsilonKO1     betaKO    gammaKO    alphaKO    deltaKO       bKO2
"GHJL-489" "GHJL-488" "GHJL-487" "GHJL-486" "GHJL-485" "GHJL-484" "GHJL-483"
      bKO1
"GHJL-482"

$DMAG573370$`TUHJL-2482`
        cKO         aKO
"GHJL-4272" "GHJL-4271"


$RSP373994
$RSP373994$`TULJQ-2731`
    gammaKO     alphaKO     deltaKO        bKO2
"GLJQ-3670" "GLJQ-3669" "GLJQ-3668" "GLJQ-3667"

$RSP373994$`TULJQ-1821`
     betaKO   epsilonKO
"GLJQ-2460" "GLJQ-2459"

$RSP373994$`TULJQ-2729`
        cKO
"GLJQ-3665"
```

$RSP373994$`TULJQ-2728`
aKO
"GLJQ-3664"

$RSP373994$`TULJQ-2730`
bKO1
"GLJQ-3666"

$`RINT718255-WGS`
$`RINT718255-WGS`$`TUSUO-1536`
epsilonKO1 betaKO1 gammaKO1 alphaKO deltaKO1 bKO1
"GSUO-2674" "GSUO-2673" "GSUO-2672" "GSUO-2671" "GSUO-2670" "GSUO-2669"
cKO1 aKO
"GSUO-2668" "GSUO-2667"

$`RINT718255-WGS`$`TUSUO-1956`
epsilonKO2 betaKO2 gammaKO2 deltaKO2 bKO2 cKO2
"GSUO-3491" "GSUO-3490" "GSUO-3489" "GSUO-3488" "GSUO-3487" "GSUO-3486"

$RJAP652620
$RJAP652620$`TUJUC-589`
deltaKO alphaKO gammaKO betaKO epsilonKO
"GJUC-915" "GJUC-914" "GJUC-913" "GJUC-912" "GJUC-911"

$RJAP652620$`TUJUC-11`
aKO cKO bKO2 bKO1
"GJUC-21" "GJUC-20" "GJUC-19" "GJUC-18"

$RLEG395491
$RLEG395491$`TUHX2-3743`
deltaKO alphaKO gammaKO betaKO epsilonKO
"GHX2-3994" "GHX2-3993" "GHX2-3992" "GHX2-3991" "GHX2-3989"

$RLEG395491$`TUHX2-1710`
bKO2 bKO1 cKO aKO
"GHX2-564" "GHX2-563" "GHX2-562" "GHX2-561"

$RLIT391595
$RLIT391595$`TUJEH-1557`
deltaKO alphaKO gammaKO betaKO epsilonKO
"GJEH-2721" "GJEH-2720" "GJEH-2719" "GJEH-2718" "GJEH-2717"

$RLIT391595$`TUJEH-1916`
aKO cKO bKO2 bKO1
"GJEH-3442" "GJEH-3441" "GJEH-3440" "GJEH-3439"

$`BAMY1338518-WGS`
$`BAMY1338518-WGS`$`TUSFQ-1973`
aKO cKO bKO deltaKO alphaKO gammaKO

"GSFQ-3693" "GSFQ-3692" "GSFQ-3691" "GSFQ-3690" "GSFQ-3689" "GSFQ-3688"
betaKO epsilonKO
"GSFQ-3687" "GSFQ-3686"

$`APHA1173064-WGS`
$`APHA1173064-WGS`$`TUSFF-810`
deltaKO alphaKO
"GSFF-1237" "GSFF-1236"

$`APHA1173064-WGS`$`TUSFF-294`
betaKO
"GSFF-479"

$`APHA1173064-WGS`$`TUSFF-407`
gammaKO1
"GSFF-662"

$`APHA1173064-WGS`$`TUSFF-468`
gammaKO2
"GSFF-748"

$`APHA1173064-WGS`$`TUSFF-293`
epsilonKO
"GSFF-478"

$`APHA1173064-WGS`$`TUSFF-720`
aKO cKO bKO2 bKO1
"GSFF-1101" "GSFF-1100" "GSFF-1099" "GSFF-1098"

$`BAMY1385727-WGS`
$`BAMY1385727-WGS`$`TUSG5-2004`
aKO cKO bKO deltaKO alphaKO gammaKO
"GSG5-3721" "GSG5-3720" "GSG5-3719" "GSG5-3718" "GSG5-3717" "GSG5-3716"
betaKO epsilonKO
"GSG5-3715" "GSG5-3714"

$`BAMY1150475-WGS`
$`BAMY1150475-WGS`$`TUSHL-1851`
aKO cKO bKO deltaKO alphaKO gammaKO
"GSHL-3444" "GSHL-3443" "GSHL-3442" "GSHL-3441" "GSHL-3440" "GSHL-3439"
betaKO epsilonKO
"GSHL-3438" "GSHL-3437"

$`BAMY1150476-WGS`
$`BAMY1150476-WGS`$`TUSFT-1852`
aKO cKO bKO deltaKO alphaKO gammaKO
"GSFT-3453" "GSFT-3452" "GSFT-3451" "GSFT-3450" "GSFT-3449" "GSFT-3448"
betaKO epsilonKO
"GSFT-3447" "GSFT-3446"

```
$AMED749927
$AMED749927$`TUCZN-3894`
    alphaKO     gammaKO      betaKO
"GCZN-7711" "GCZN-7710" "GCZN-7709"

$AMED749927$`TUCZN-3895`
       cKO2         bKO     deltaKO
"GCZN-7714" "GCZN-7713" "GCZN-7712"

$AMED749927$`TUCZN-3893`
  epsilonKO
"GCZN-7708"

$AMED749927$`TUCZN-2042`
       cKO1
"GCZN-4027"

$AMED749927$`TUCZN-3896`
        aKO
"GCZN-7715"


$RLEG395492
$RLEG395492$`TUJB3-3240`
    deltaKO     alphaKO     gammaKO      betaKO   epsilonKO
"GJB3-3709" "GJB3-3708" "GJB3-3707" "GJB3-3706" "GJB3-3704"

$RLEG395492$`TUJB3-1433`
      bKO2       bKO1        cKO        aKO
"GJB3-522" "GJB3-521" "GJB3-520" "GJB3-519"


$CRUT413404
$CRUT413404$`TUHM7-470`
        aKO         cKO         bKO     deltaKO     alphaKO     gammaKO
"GHM7-1091" "GHM7-1090" "GHM7-1089" "GHM7-1088" "GHM7-1087" "GHM7-1086"
     betaKO   epsilonKO
"GHM7-1085" "GHM7-1084"


$CMET266264
$CMET266264$`TUJ5G-3583`
        aKO         cKO         bKO     deltaKO     alphaKO     gammaKO
"GJ5G-3749" "GJ5G-3748" "GJ5G-3746" "GJ5G-3745" "GJ5G-3744" "GJ5G-3743"
     betaKO   epsilonKO
"GJ5G-3742" "GJ5G-3741"


$RMAR762570
$RMAR762570$`TUJAN-205`
   gammaKO    alphaKO    deltaKO        bKO        cKO        aKO
"GJAN-307" "GJAN-306" "GJAN-305" "GJAN-304" "GJAN-303" "GJAN-302"
```

```
$RMAR762570$`TUJAN-92`
   betaKO epsilonKO
"GJAN-75" "GJAN-74"


$RMAS1105112
$RMAS1105112$`TULJ9-197`
 epsilonKO     betaKO    gammaKO    alphaKO    deltaKO
"GLJ9-293" "GLJ9-292" "GLJ9-291" "GLJ9-290" "GLJ9-289"

$RMAS1105112$`TULJ9-39`
      aKO       cKO      bKO2      bKO1
"GLJ9-46" "GLJ9-45" "GLJ9-44" "GLJ9-43"


$RMON1105114
$RMON1105114$`TULJA-388`
   deltaKO    alphaKO    gammaKO     betaKO  epsilonKO
"GLJA-600" "GLJA-599" "GLJA-598" "GLJA-597" "GLJA-596"

$RMON1105114$`TULJA-482`
       cKO       bKO2       bKO1
"GLJA-747" "GLJA-746" "GLJA-745"

$RMON1105114$`TULJA-483`
       aKO
"GLJA-749"


$RMAR518766
$RMAR518766$`TUJJ8-222`
   gammaKO    alphaKO    deltaKO        bKO        cKO        aKO
"GJJ8-326" "GJJ8-325" "GJJ8-324" "GJJ8-323" "GJJ8-322" "GJJ8-321"

$RMAR518766$`TUJJ8-106`
   betaKO epsilonKO
"GJJ8-78" "GJJ8-77"


$APLE434271
$APLE434271$`TUIX7-1008`
        aKO         cKO         bKO     deltaKO     alphaKO     gammaKO
"GIX7-1725" "GIX7-1724" "GIX7-1723" "GIX7-1722" "GIX7-1721" "GIX7-1720"
     betaKO   epsilonKO
"GIX7-1719" "GIX7-1718"


$RMAS416276
$RMAS416276$`TUJD3-790`
    deltaKO     alphaKO     gammaKO      betaKO   epsilonKO
"GJD3-1284" "GJD3-1283" "GJD3-1282" "GJD3-1281" "GJD3-1280"

$RMAS416276$`TUJD3-25`
      aKO       cKO      bKO2      bKO1
```

"GJD3-30" "GJD3-29" "GJD3-28" "GJD3-27"

$RMUC680646
$RMUC680646$`TUH63-784`
alphaKO
"GH63-1263"

$RMUC680646$`TUH63-782`
betaKO epsilonKO
"GH63-1260" "GH63-1259"

$RMUC680646$`TUH63-783`
gammaKO
"GH63-1262"

$RMUC680646$`TUH63-785`
bKO deltaKO
"GH63-1266" "GH63-1265"

$RMUC680646$`TUH63-786`
cKO
"GH63-1267"

$RMUC680646$`TUH63-787`
aKO
"GH63-1268"

$HPYL585538
$HPYL585538$`TULE6-496`
bKO2 bKO1 deltaKO alphaKO gammaKO betaKO
"GLE6-1180" "GLE6-1179" "GLE6-1178" "GLE6-1177" "GLE6-1176" "GLE6-1175"
epsilonKO
"GLE6-1174"

$HPYL585538$`TULE6-535`
cKO
"GLE6-1266"

$HPYL585538$`TULE6-238`
aKO
"GLE6-592"

$AAVE643561
$AAVE643561$`TUHRD-257`
epsilonKO1 betaKO gammaKO alphaKO deltaKO bKO cKO
"GHRD-443" "GHRD-442" "GHRD-441" "GHRD-440" "GHRD-439" "GHRD-438" "GHRD-437"
aKO
"GHRD-436"

$AAVE643561$`TUHRD-929`
epsilonKO2

"GHRD-1753"

$ROPA632772
$ROPA632772$`TUH0Q-1190`
aKO cKO bKO deltaKO alphaKO gammaKO
"GH0Q-1206" "GH0Q-1205" "GH0Q-1204" "GH0Q-1203" "GH0Q-1202" "GH0Q-1201"
betaKO epsilonKO
"GH0Q-1200" "GH0Q-1199"

$`RORN1286170-WGS`
$`RORN1286170-WGS`$`TUSTG-1999`
epsilonKO betaKO gammaKO alphaKO deltaKO bKO
"GSTG-3787" "GSTG-3786" "GSTG-3785" "GSTG-3784" "GSTG-3783" "GSTG-3782"
cKO aKO
"GSTG-3781" "GSTG-3780"

$`RPAL258594-WGS`
$`RPAL258594-WGS`$`TUSTP-100`
deltaKO alphaKO gammaKO betaKO epsilonKO
"GSTP-182" "GSTP-181" "GSTP-180" "GSTP-179" "GSTP-178"

$`RPAL258594-WGS`$`TUSTP-494`
aKO cKO
"GSTP-858" "GSTP-857"

$`RPAL258594-WGS`$`TUSTP-493`
bKO2 bKO1
"GSTP-856" "GSTP-855"

$RPAL316058
$RPAL316058$`TUHF1-153`
deltaKO alphaKO gammaKO betaKO epsilonKO
"GHF1-271" "GHF1-270" "GHF1-269" "GHF1-268" "GHF1-267"

$RPAL316058$`TUHF1-2668`
cKO aKO
"GHF1-4631" "GHF1-4630"

$RPAL316058$`TUHF1-2669`
bKO2 bKO1
"GHF1-4633" "GHF1-4632"

$RPAL316056
$RPAL316056$`TUH3E-113`
deltaKO alphaKO gammaKO betaKO epsilonKO
"GH3E-181" "GH3E-180" "GH3E-179" "GH3E-178" "GH3E-177"

$RPAL316056$`TUH3E-2747`
aKO cKO

"GH3E-4887" "GH3E-4886"

$RPAL316056$`TUH3E-2746`
bKO2 bKO1
"GH3E-4885" "GH3E-4884"

$RPAL316057
$RPAL316057$`TUHDC-342`
epsilonKO betaKO gammaKO alphaKO deltaKO
"GHDC-574" "GHDC-573" "GHDC-572" "GHDC-571" "GHDC-570"

$RPAL316057$`TUHDC-499`
aKO cKO
"GHDC-850" "GHDC-849"

$RPAL316057$`TUHDC-498`
bKO2 bKO1
"GHDC-848" "GHDC-847"

$RPAL316055
$RPAL316055$`TUHR9-182`
deltaKO alphaKO gammaKO betaKO epsilonKO
"GHR9-286" "GHR9-285" "GHR9-284" "GHR9-283" "GHR9-282"

$RPAL316055$`TUHR9-2830`
aKO cKO
"GHR9-4829" "GHR9-4828"

$RPAL316055$`TUHR9-2829`
bKO2 bKO1
"GHR9-4827" "GHR9-4826"

$RPRO1105098
$RPRO1105098$`TULJG-116`
deltaKO alphaKO gammaKO betaKO epsilonKO
"GLJG-190" "GLJG-189" "GLJG-188" "GLJG-187" "GLJG-186"

$RPRO1105098$`TULJG-180`
aKO cKO bKO2 bKO1
"GLJG-292" "GLJG-291" "GLJG-290" "GLJG-289"

$`APAS1266844-WGS`
$`APAS1266844-WGS`$`TUSEG-801`
epsilonKO betaKO gammaKO alphaKO deltaKO
"GSEG-1552" "GSEG-1551" "GSEG-1550" "GSEG-1549" "GSEG-1548"

$`APAS1266844-WGS`$`TUSEG-613`
aKO cKO bKO2 bKO1
"GSEG-1213" "GSEG-1212" "GSEG-1211" "GSEG-1210"

```
$RPHI481009
$RPHI481009$`TULJC-819`
    deltaKO     alphaKO     gammaKO      betaKO   epsilonKO 
"GLJC-1243" "GLJC-1242" "GLJC-1241" "GLJC-1240" "GLJC-1239" 

$RPHI481009$`TULJC-22`
      aKO       cKO      bKO2      bKO1 
"GLJC-32" "GLJC-31" "GLJC-30" "GLJC-29" 


$LBIF355278
$LBIF355278$`TUHTJ-367`
 epsilonKO     betaKO    gammaKO    alphaKO    deltaKO        bKO        cKO 
"GHTJ-778" "GHTJ-777" "GHTJ-776" "GHTJ-775" "GHTJ-774" "GHTJ-773" "GHTJ-772" 
       aKO 
"GHTJ-771" 


$RPEA562019
$RPEA562019$`TUJD7-435`
   deltaKO    alphaKO    gammaKO     betaKO  epsilonKO 
"GJD7-639" "GJD7-638" "GJD7-637" "GJD7-636" "GJD7-635" 

$RPEA562019$`TUJD7-106`
       aKO        cKO       bKO2       bKO1 
"GJD7-134" "GJD7-133" "GJD7-132" "GJD7-131" 


$`RPRO1290428-WGS`
$`RPRO1290428-WGS`$`TUSTR-480`
 epsilonKO     betaKO    gammaKO    alphaKO    deltaKO 
"GSTR-750" "GSTR-749" "GSTR-748" "GSTR-747" "GSTR-746" 

$`RPRO1290428-WGS`$`TUSTR-401`
      bKO2       bKO1        cKO        aKO 
"GSTR-632" "GSTR-631" "GSTR-630" "GSTR-629" 


$RPHO1150469
$RPHO1150469$`TULJ6-914`
    deltaKO     alphaKO     gammaKO      betaKO   epsilonKO 
"GLJ6-1567" "GLJ6-1566" "GLJ6-1565" "GLJ6-1564" "GLJ6-1563" 

$RPHO1150469$`TULJ6-150`
       aKO        cKO       bKO2       bKO1 
"GLJ6-245" "GLJ6-244" "GLJ6-243" "GLJ6-242" 


$`RPRO1290427-WGS`
$`RPRO1290427-WGS`$`TUSTT-162`
 epsilonKO     betaKO    gammaKO    alphaKO 
"GSTT-255" "GSTT-254" "GSTT-253" "GSTT-252" 
```

```
$`RPRO1290427-WGS`$`TUSTT-161`
   deltaKO
"GSTT-251"

$`RPRO1290427-WGS`$`TUSTT-84`
      bKO2       bKO1        cKO        aKO
"GSTT-139" "GSTT-138" "GSTT-137" "GSTT-136"


$RPRO1105096
$RPRO1105096$`TULJD-509`
   deltaKO    alphaKO    gammaKO     betaKO  epsilonKO
"GLJD-797" "GLJD-796" "GLJD-795" "GLJD-794" "GLJD-793"

$RPRO1105096$`TULJD-12`
      aKO       cKO      bKO2      bKO1
"GLJD-23" "GLJD-22" "GLJD-21" "GLJD-20"


$RPAR1105108
$RPAR1105108$`TULJB-806`
    deltaKO     alphaKO     gammaKO      betaKO   epsilonKO
"GLJB-1226" "GLJB-1225" "GLJB-1224" "GLJB-1223" "GLJB-1222"

$RPAR1105108$`TULJB-20`
      cKO      bKO2      bKO1
"GLJB-31" "GLJB-30" "GLJB-29"

$RPAR1105108$`TULJB-21`
      aKO
"GLJB-33"


$RPRO449216
$RPRO449216$`TULJI-549`
   alphaKO
"GLJI-896"

$RPRO449216$`TULJI-547`
    betaKO
"GLJI-894"

$RPRO449216$`TULJI-548`
   gammaKO
"GLJI-895"

$RPRO449216$`TULJI-550`
   deltaKO
"GLJI-897"

$RPRO449216$`TULJI-546`
 epsilonKO
"GLJI-893"
```

```
$RPRO449216$`TULJI-11`
      aKO       cKO      bKO2      bKO1 
"GLJI-23" "GLJI-22" "GLJI-21" "GLJI-20" 


$`RPRO272947-WGS`
$`RPRO272947-WGS`$`TUSTS-505`
   deltaKO    alphaKO    gammaKO     betaKO  epsilonKO 
"GSTS-799" "GSTS-798" "GSTS-797" "GSTS-796" "GSTS-795" 

$`RPRO272947-WGS`$`TUSTS-11`
      aKO       cKO      bKO2      bKO1 
"GSTS-23" "GSTS-22" "GSTS-21" "GSTS-20" 


$RPRO1105095
$RPRO1105095$`TULJH-511`
   deltaKO    alphaKO    gammaKO     betaKO  epsilonKO 
"GLJH-797" "GLJH-796" "GLJH-795" "GLJH-794" "GLJH-793" 

$RPRO1105095$`TULJH-12`
      aKO       cKO      bKO2      bKO1 
"GLJH-23" "GLJH-22" "GLJH-21" "GLJH-20" 


$LJOH633699
$LJOH633699$`TUJ95-647`
        aKO         cKO         bKO     deltaKO     alphaKO     gammaKO 
"GJ95-1203" "GJ95-1202" "GJ95-1201" "GJ95-1200" "GJ95-1199" "GJ95-1198" 
     betaKO   epsilonKO 
"GJ95-1197" "GJ95-1196" 


$APRO859653
$APRO859653$`TUL7R-3`
epsilonKO    betaKO   gammaKO   alphaKO   deltaKO 
"GL7R-28" "GL7R-27" "GL7R-26" "GL7R-25" "GL7R-24" 

$APRO859653$`TUL7R-30`
       aKO        cKO       bKO2       bKO1 
"GL7R-135" "GL7R-134" "GL7R-133" "GL7R-132" 


$RPAL395960
$RPAL395960$`TUHPC-99`
   deltaKO   alphaKO1 
"GHPC-177" "GHPC-176" 

$RPAL395960$`TUHPC-606`
    betaKO2  epsilonKO2         aKO2         cKO2         bKO3    alphaKO2 
"GHPC-1071" "GHPC-1070" "GHPC-1067" "GHPC-1066" "GHPC-1065" "GHPC-1064" 
   gammaKO2 
"GHPC-1063" 
```

$RPAL395960$`TUHPC-98`
  gammaKO1   betaKO1 epsilonKO1
"GHPC-175" "GHPC-174" "GHPC-173"

$RPAL395960$`TUHPC-517`
      aKO1   cKO1
"GHPC-926" "GHPC-925"

$RPAL395960$`TUHPC-516`
      bKO2   bKO1
"GHPC-924" "GHPC-923"

$RPRO1105099
$RPRO1105099$`TULJJ-509`
   deltaKO   alphaKO   gammaKO   betaKO epsilonKO
"GLJJ-788" "GLJJ-787" "GLJJ-786" "GLJJ-785" "GLJJ-784"

$RPRO1105099$`TULJJ-10`
      aKO   cKO   bKO2   bKO1
"GLJJ-20" "GLJJ-19" "GLJJ-18" "GLJJ-17"

$RPRO1105094
$RPRO1105094$`TULJE-512`
   deltaKO   alphaKO   gammaKO   betaKO epsilonKO
"GLJE-798" "GLJE-797" "GLJE-796" "GLJE-795" "GLJE-794"

$RPRO1105094$`TULJE-12`
      aKO   cKO   bKO2   bKO1
"GLJE-23" "GLJE-22" "GLJE-21" "GLJE-20"

$RPAL652103
$RPAL652103$`TUHQR-281`
 epsilonKO   betaKO   gammaKO   alphaKO   deltaKO
"GHQR-471" "GHQR-470" "GHQR-469" "GHQR-468" "GHQR-467"

$RPAL652103$`TUHQR-576`
        aKO   cKO
"GHQR-1025" "GHQR-1024"

$RPAL652103$`TUHQR-575`
       bKO2   bKO1
"GHQR-1023" "GHQR-1022"

$RPRO1105097
$RPRO1105097$`TULJF-505`
   deltaKO   alphaKO   gammaKO   betaKO epsilonKO
"GLJF-792" "GLJF-791" "GLJF-790" "GLJF-789" "GLJF-788"

$RPRO1105097$`TULJF-12`
      aKO   cKO   bKO2   bKO1

"GLJF-23" "GLJF-22" "GLJF-21" "GLJF-20"

$RRIC1105105
$RRIC1105105$`TUKDS-814`
deltaKO alphaKO gammaKO betaKO epsilonKO
"GKDS-1240" "GKDS-1239" "GKDS-1238" "GKDS-1237" "GKDS-1236"

$RRIC1105105$`TUKDS-25`
aKO cKO bKO2 bKO1
"GKDS-36" "GKDS-35" "GKDS-34" "GKDS-33"

$RRIC1105104
$RRIC1105104$`TUJWU-27`
epsilonKO betaKO gammaKO alphaKO deltaKO
"GJWU-42" "GJWU-41" "GJWU-40" "GJWU-39" "GJWU-38"

$RRIC1105104$`TUJWU-809`
bKO2 bKO1 cKO aKO
"GJWU-1234" "GJWU-1233" "GJWU-1232" "GJWU-1231"

$RRIC1105102
$RRIC1105102$`TUJU0-823`
deltaKO alphaKO gammaKO betaKO epsilonKO
"GJU0-1246" "GJU0-1245" "GJU0-1244" "GJU0-1243" "GJU0-1242"

$RRIC1105102$`TUJU0-26`
aKO cKO bKO2 bKO1
"GJU0-37" "GJU0-36" "GJU0-35" "GJU0-34"

$RRHI1105113
$RRHI1105113$`TULJK-837`
deltaKO alphaKO gammaKO betaKO epsilonKO
"GLJK-1251" "GLJK-1250" "GLJK-1249" "GLJK-1248" "GLJK-1247"

$RRHI1105113$`TULJK-71`
bKO2 bKO1 cKO aKO
"GLJK-92" "GLJK-91" "GLJK-90" "GLJK-89"

$RRUB1036743
$RRUB1036743$`TULJ7-735`
betaKO gammaKO alphaKO deltaKO
"GLJ7-1292" "GLJ7-1291" "GLJ7-1290" "GLJ7-1289"

$RRUB1036743$`TULJ7-736`
epsilonKO
"GLJ7-1293"

$RRUB1036743$`TULJ7-1911`
aKO cKO

"GLJ7-3372" "GLJ7-3371"

$RRUB1036743$`TULJ7-1910`
bKO2 bKO1
"GLJ7-3370" "GLJ7-3369"

$SSAL347253
$SSAL347253$`TULLH-236`
epsilonKO betaKO gammaKO alphaKO deltaKO bKO aKO
"GLLH-471" "GLLH-470" "GLLH-469" "GLLH-468" "GLLH-467" "GLLH-466" "GLLH-465"
cKO
"GLLH-464"

$LJOH909954
$LJOH909954$`TULFA-660`
aKO cKO bKO deltaKO alphaKO gammaKO
"GLFA-1233" "GLFA-1232" "GLFA-1231" "GLFA-1230" "GLFA-1229" "GLFA-1228"
betaKO epsilonKO
"GLFA-1227" "GLFA-1226"

$RRIC1105103
$RRIC1105103$`TUJY3-813`
deltaKO alphaKO gammaKO betaKO epsilonKO
"GJY3-1237" "GJY3-1236" "GJY3-1235" "GJY3-1234" "GJY3-1233"

$RRIC1105103$`TUJY3-26`
aKO cKO bKO2 bKO1
"GJY3-37" "GJY3-36" "GJY3-35" "GJY3-34"

$`BAMY1225788-WGS`
$`BAMY1225788-WGS`$`TUSFO-1882`
aKO cKO bKO deltaKO alphaKO gammaKO
"GSFO-3564" "GSFO-3563" "GSFO-3562" "GSFO-3561" "GSFO-3560" "GSFO-3559"
betaKO epsilonKO
"GSFO-3558" "GSFO-3557"

$ANTHRA
$ANTHRA$`TUFG-15525`
aKO cKO bKO deltaKO alphaKO gammaKO betaKO epsilonKO
"ATPB" "ATPE" "ATPF" "ATPH" "ATPA" "ATPG" "ATPD" "ATPC"

$`BANI1281781-WGS`
$`BANI1281781-WGS`$`TUSGS-878`
aKO cKO bKO deltaKO alphaKO gammaKO
"GSGS-1421" "GSGS-1420" "GSGS-1419" "GSGS-1418" "GSGS-1417" "GSGS-1416"
betaKO epsilonKO
"GSGS-1415" "GSGS-1414"

```
$`BANI1167629-WGS`
$`BANI1167629-WGS`$`TUSGQ-924`
       aKO         cKO         bKO     deltaKO     alphaKO     gammaKO
"GSGQ-1445" "GSGQ-1444" "GSGQ-1443" "GSGQ-1442" "GSGQ-1441" "GSGQ-1440"
     betaKO   epsilonKO
"GSGQ-1439" "GSGQ-1438"


$RRIC392021
$RRIC392021$`TUIY1-794`
    deltaKO     alphaKO     gammaKO      betaKO   epsilonKO
"GIY1-1239" "GIY1-1238" "GIY1-1237" "GIY1-1236" "GIY1-1235"

$RRIC392021$`TUIY1-25`
      aKO       cKO      bKO2      bKO1
"GIY1-37" "GIY1-36" "GIY1-35" "GIY1-34"


$RRIC452659
$RRIC452659$`TUHSN-856`
    deltaKO     alphaKO     gammaKO      betaKO   epsilonKO
"GHSN-1347" "GHSN-1346" "GHSN-1345" "GHSN-1344" "GHSN-1343"

$RRIC452659$`TUHSN-26`
      aKO       cKO      bKO2      bKO1
"GHSN-38" "GHSN-37" "GHSN-36" "GHSN-35"


$RRIC1105100
$RRIC1105100$`TUJW1-816`
    deltaKO     alphaKO     gammaKO      betaKO   epsilonKO
"GJW1-1232" "GJW1-1231" "GJW1-1230" "GJW1-1229" "GJW1-1228"

$RRIC1105100$`TUJW1-39`
      aKO       cKO      bKO2      bKO1
"GJW1-37" "GJW1-36" "GJW1-35" "GJW1-34"


$RRIC1105101
$RRIC1105101$`TUJU5-799`
    deltaKO     alphaKO     gammaKO      betaKO   epsilonKO
"GJU5-1211" "GJU5-1210" "GJU5-1209" "GJU5-1208" "GJU5-1207"

$RRIC1105101$`TUJU5-24`
      aKO       cKO      bKO2      bKO1
"GJU5-35" "GJU5-34" "GJU5-33" "GJU5-32"


$SPAR936154
$SPAR936154$`TUHCN-288`
   alphaKO    deltaKO       bKO       aKO       cKO
"GHCN-532" "GHCN-531" "GHCN-530" "GHCN-529" "GHCN-528"
```

```
$SPAR936154$`TUHCN-289`
 epsilonKO     betaKO
"GHCN-534" "GHCN-533"

$SPAR936154$noTU
gammaKO
    NA



$RSP357808
$RSP357808$`TUH5Z-323`
      aKO        cKO        bKO    deltaKO    alphaKO    gammaKO     betaKO
"GH5Z-522" "GH5Z-521" "GH5Z-520" "GH5Z-519" "GH5Z-518" "GH5Z-517" "GH5Z-516"
 epsilonKO
"GH5Z-515"



$LJOH257314
$LJOH257314$`TUJN3-417`
 epsilonKO     betaKO    gammaKO    alphaKO    deltaKO        bKO        cKO
"GJN3-805" "GJN3-804" "GJN3-803" "GJN3-802" "GJN3-801" "GJN3-800" "GJN3-799"
      aKO
"GJN3-798"



$RRUB269796
$RRUB269796$`TUCN1-741`
  epsilonKO      betaKO     gammaKO     alphaKO     deltaKO
"GCN1-1250" "GCN1-1249" "GCN1-1248" "GCN1-1247" "GCN1-1246"

$RRUB269796$`TUCN1-1888`
       aKO         cKO
"GCN1-3301" "GCN1-3300"

$RRUB269796$`TUCN1-1887`
      bKO2        bKO1
"GCN1-3299" "GCN1-3298"



$RSAL288705
$RSAL288705$`TUHX1-720`
  epsilonKO      betaKO     gammaKO     alphaKO     deltaKO         bKO
"GHX1-1449" "GHX1-1448" "GHX1-1447" "GHX1-1446" "GHX1-1445" "GHX1-1444"
       cKO         aKO
"GHX1-1443" "GHX1-1442"



$UTER471821
$UTER471821$`TUJAD-297`
      aKO        cKO        bKO    alphaKO
"GJAD-499" "GJAD-498" "GJAD-497" "GJAD-495"

$UTER471821$`TUJAD-296`
   gammaKO     betaKO  epsilonKO
```

```
"GJAD-494" "GJAD-493" "GJAD-492"

$UTER471821$noTU
deltaKO
    NA



$`RSOL1262456-WGS`
$`RSOL1262456-WGS`$`TUSTD-2749`
       aKO         cKO         bKO     deltaKO     alphaKO     gammaKO
"GSTD-3422" "GSTD-3421" "GSTD-3420" "GSTD-3419" "GSTD-3418" "GSTD-3417"
    betaKO  epsilonKO1
"GSTD-3416" "GSTD-3415"

$`RSOL1262456-WGS`$`TUSTD-519`
 epsilonKO2
"GSTD-4464"



$RSPH349101
$RSPH349101$`TUHC8-561`
epsilonKO1    betaKO1   gammaKO1   alphaKO1    deltaKO
"GHC8-994" "GHC8-993" "GHC8-992" "GHC8-991" "GHC8-990"

$RSPH349101$`TUHC8-19`
   gammaKO2    alphaKO2        bKO3        cKO2        aKO2  epsilonKO2
"GHC8-4173" "GHC8-4172" "GHC8-4171" "GHC8-4170" "GHC8-4169" "GHC8-4167"
    betaKO2
"GHC8-4166"

$RSPH349101$`TUHC8-1431`
      aKO1        cKO1        bKO2        bKO1
"GHC8-2741" "GHC8-2740" "GHC8-2739" "GHC8-2738"



$RSLI761193
$RSLI761193$`TUHKZ-773`
   alphaKO
"GHKZ-956"

$RSLI761193$`TUHKZ-1180`
    betaKO   epsilonKO
"GHKZ-1630" "GHKZ-1629"

$RSLI761193$`TUHKZ-774`
   gammaKO
"GHKZ-958"

$RSLI761193$`TUHKZ-277`
      aKO        cKO        bKO    deltaKO
"GHKZ-144" "GHKZ-143" "GHKZ-142" "GHKZ-141"



$RSPH557760
```

```
$RSPH557760$`TUH1P-1057`
epsilonKO1    betaKO1   gammaKO1   alphaKO1    deltaKO
"GH1P-629" "GH1P-628" "GH1P-627" "GH1P-626" "GH1P-625"

$RSPH557760$`TUH1P-31`
    betaKO2  epsilonKO2        aKO2        cKO2        bKO3    alphaKO2
"GH1P-4597" "GH1P-4596" "GH1P-4593" "GH1P-4592" "GH1P-4591" "GH1P-4590"
   gammaKO2
"GH1P-4588"

$RSPH557760$`TUH1P-1984`
       aKO1        cKO1        bKO2        bKO1
"GH1P-2463" "GH1P-2462" "GH1P-2461" "GH1P-2460"


$RSOL1031711
$RSOL1031711$`TULJ5-1019`
    betaKO    gammaKO    alphaKO    deltaKO        bKO
"GLJ5-127" "GLJ5-126" "GLJ5-125" "GLJ5-124" "GLJ5-123"

$RSOL1031711$`TULJ5-1020`
epsilonKO1
"GLJ5-128"

$RSOL1031711$`TULJ5-624`
 epsilonKO2
"GLJ5-4517"

$RSOL1031711$`TULJ5-1018`
       cKO
"GLJ5-122"

$RSOL1031711$`TULJ5-1017`
       aKO
"GLJ5-121"


$STHE264199
$STHE264199$`TUI6K-285`
 epsilonKO     betaKO    gammaKO    alphaKO    deltaKO        bKO        aKO
"GI6K-531" "GI6K-530" "GI6K-529" "GI6K-528" "GI6K-527" "GI6K-526" "GI6K-525"
       cKO
"GI6K-524"


$RSPH349102
$RSPH349102$`TUHE1-1703`
    deltaKO     alphaKO     gammaKO      betaKO   epsilonKO
"GHE1-3531" "GHE1-3530" "GHE1-3529" "GHE1-3528" "GHE1-3527"

$RSPH349102$`TUHE1-716`
       bKO2        bKO1         cKO         aKO
"GHE1-1523" "GHE1-1522" "GHE1-1521" "GHE1-1520"
```

```
$RSLO1105109
$RSLO1105109$`TULJL-810`
    deltaKO     alphaKO     gammaKO      betaKO   epsilonKO 
"GLJL-1249" "GLJL-1248" "GLJL-1247" "GLJL-1246" "GLJL-1245" 

$RSLO1105109$`TULJL-22`
      cKO      bKO2      bKO1 
"GLJL-34" "GLJL-33" "GLJL-32" 

$RSLO1105109$`TULJL-23`
      aKO 
"GLJL-36" 


$LKEF1033837
$LKEF1033837$`TUHZJ-619`
       aKO        cKO        bKO    deltaKO    alphaKO    gammaKO     betaKO 
"GHZJ-939" "GHZJ-938" "GHZJ-937" "GHZJ-936" "GHZJ-935" "GHZJ-934" "GHZJ-933" 
 epsilonKO 
"GHZJ-932" 


$RTAT365046
$RTAT365046$`TUHCU-181`
 epsilonKO     betaKO    gammaKO    alphaKO 
"GHCU-375" "GHCU-374" "GHCU-373" "GHCU-372" 

$RTAT365046$`TUHCU-180`
   deltaKO        bKO        cKO 
"GHCU-371" "GHCU-370" "GHCU-369" 

$RTAT365046$`TUHCU-179`
       aKO 
"GHCU-368" 


$RTYP1003202
$RTYP1003202$`TULJM-509`
   deltaKO    alphaKO    gammaKO     betaKO  epsilonKO 
"GLJM-791" "GLJM-790" "GLJM-789" "GLJM-788" "GLJM-787" 

$RTYP1003202$`TULJM-68`
      bKO2       bKO1        cKO        aKO 
"GLJM-111" "GLJM-110" "GLJM-109" "GLJM-108" 


$`RTRO698761-WGS`
$`RTRO698761-WGS`$`TUSTK-3478`
    deltaKO     alphaKO     gammaKO      betaKO   epsilonKO 
"GSTK-3345" "GSTK-3344" "GSTK-3343" "GSTK-3342" "GSTK-3341" 

$`RTRO698761-WGS`$`TUSTK-2007`
       cKO 
```

"GSTK-831"

$`RTRO698761-WGS`$`TUSTK-2006`
aKO
"GSTK-830"

$`RTRO698761-WGS`$`TUSTK-2008`
bKO2 bKO1
"GSTK-833" "GSTK-832"

$RTYP1003201
$RTYP1003201$`TULJN-510`
deltaKO alphaKO gammaKO betaKO epsilonKO
"GLJN-791" "GLJN-790" "GLJN-789" "GLJN-788" "GLJN-787"

$RTYP1003201$`TULJN-67`
bKO2 bKO1 cKO aKO
"GLJN-110" "GLJN-109" "GLJN-108" "GLJN-107"

$RTYP257363
$RTYP257363$`TUJEQ-518`
deltaKO alphaKO gammaKO betaKO epsilonKO
"GJEQ-831" "GJEQ-830" "GJEQ-829" "GJEQ-828" "GJEQ-827"

$RTYP257363$`TUJEQ-66`
bKO2 bKO1 cKO aKO
"GJEQ-116" "GJEQ-115" "GJEQ-114" "GJEQ-113"

$RVAN648757
$RVAN648757$`TUHZT-1965`
epsilonKO betaKO gammaKO alphaKO deltaKO
"GHZT-3311" "GHZT-3310" "GHZT-3309" "GHZT-3308" "GHZT-3307"

$RVAN648757$`TUHZT-905`
cKO aKO
"GHZT-1570" "GHZT-1569"

$RVAN648757$`TUHZT-906`
bKO2 bKO1
"GHZT-1572" "GHZT-1571"

$RXYL266117
$RXYL266117$`TUH8O-635`
aKO cKO bKO deltaKO alphaKO gammaKO
"GH8O-1678" "GH8O-1677" "GH8O-1676" "GH8O-1675" "GH8O-1674" "GH8O-1673"
betaKO epsilonKO
"GH8O-1672" "GH8O-1671"

$SENT99287

```
$SENT99287$`TUCTI-2132`
       aKO         cKO         bKO     deltaKO     alphaKO     gammaKO 
"GCTI-3901" "GCTI-3900" "GCTI-3899" "GCTI-3898" "GCTI-3897" "GCTI-3896" 
     betaKO   epsilonKO 
"GCTI-3895" "GCTI-3894" 


$SAUR451515
$SAUR451515$`TUH3C-1098`
       aKO         cKO         bKO     deltaKO     alphaKO     gammaKO 
"GH3C-2064" "GH3C-2063" "GH3C-2062" "GH3C-2061" "GH3C-2060" "GH3C-2059" 
     betaKO   epsilonKO 
"GH3C-2058" "GH3C-2057" 


$SAUR273036
$SAUR273036$`TUJVS-1087`
       aKO         cKO         bKO     deltaKO     alphaKO     gammaKO 
"GJVS-2058" "GJVS-2057" "GJVS-2056" "GJVS-2055" "GJVS-2054" "GJVS-2053" 
     betaKO   epsilonKO 
"GJVS-2052" "GJVS-2051" 


$SAUR93062
$SAUR93062$`TUCEP-1078`
       aKO         cKO         bKO     deltaKO     alphaKO     gammaKO 
"GCEP-2094" "GCEP-2093" "GCEP-2092" "GCEP-2091" "GCEP-2090" "GCEP-2089" 
     betaKO   epsilonKO 
"GCEP-2088" "GCEP-2087" 


$LKIM762051
$LKIM762051$`TUJOL-745`
  epsilonKO      betaKO     gammaKO     alphaKO     deltaKO         bKO 
"GJOL-1314" "GJOL-1313" "GJOL-1312" "GJOL-1311" "GJOL-1310" "GJOL-1309" 
        cKO         aKO 
"GJOL-1308" "GJOL-1307" 


$`SACI886293-WGS`
$`SACI886293-WGS`$`TUSWQ-1482`
   gammaKO1    alphaKO1        bKO1        cKO1        aKO1  epsilonKO1 
"GSWQ-2031" "GSWQ-2030" "GSWQ-2029" "GSWQ-2028" "GSWQ-2027" "GSWQ-2024" 
    betaKO1 
"GSWQ-2023" 

$`SACI886293-WGS`$`TUSWQ-4704`
   alphaKO2     deltaKO        bKO2        cKO2        aKO2 
"GSWQ-6876" "GSWQ-6875" "GSWQ-6874" "GSWQ-6873" "GSWQ-6872" 

$`SACI886293-WGS`$`TUSWQ-4705`
 epsilonKO2     betaKO2    gammaKO2 
"GSWQ-6880" "GSWQ-6879" "GSWQ-6878" 
```

```
$SAUR681288
$SAUR681288$`TUJ8Z-1109`
       aKO        cKO        bKO    deltaKO    alphaKO    gammaKO 
"GJ8Z-2136" "GJ8Z-2135" "GJ8Z-2134" "GJ8Z-2133" "GJ8Z-2132" "GJ8Z-2131" 
    betaKO  epsilonKO 
"GJ8Z-2130" "GJ8Z-2129" 


$SAUR426430
$SAUR426430$`TUIXC-1088`
       aKO        cKO        bKO    deltaKO    alphaKO    gammaKO 
"GIXC-2077" "GIXC-2076" "GIXC-2075" "GIXC-2074" "GIXC-2073" "GIXC-2072" 
    betaKO  epsilonKO 
"GIXC-2071" "GIXC-2070" 


$SAZO204536
$SAZO204536$`TUHRE-174`
   betaKO   gammaKO   alphaKO   deltaKO      bKO2      bKO1 
"GHRE-458" "GHRE-457" "GHRE-456" "GHRE-455" "GHRE-454" "GHRE-453" 

$SAZO204536$`TUHRE-297`
 epsilonKO 
"GHRE-832" 

$SAZO204536$`TUHRE-196`
      aKO       cKO 
"GHRE-538" "GHRE-536" 


$SAGA208435
$SAGA208435$`TUHVY-445`
 epsilonKO    betaKO   gammaKO   alphaKO   deltaKO       bKO       aKO 
"GHVY-955" "GHVY-954" "GHVY-953" "GHVY-952" "GHVY-951" "GHVY-950" "GHVY-949" 
      cKO 
"GHVY-948" 


$SAGA1117647
$SAGA1117647$`TULK7-1226`
  epsilonKO     betaKO    gammaKO    alphaKO    deltaKO        bKO 
"GLK7-2471" "GLK7-2470" "GLK7-2469" "GLK7-2468" "GLK7-2467" "GLK7-2466" 
       cKO        aKO 
"GLK7-2465" "GLK7-2464" 


$STHE767463
$STHE767463$`TULLS-290`
 epsilonKO    betaKO   gammaKO   alphaKO   deltaKO       bKO       aKO 
"GLLS-534" "GLLS-533" "GLLS-532" "GLLS-531" "GLLS-530" "GLLS-529" "GLLS-528" 
      cKO 
"GLLS-527" 
```

```
$`SAGA1309807-WGS`
$`SAGA1309807-WGS`$`TUSX9-495`
  epsilonKO      betaKO     gammaKO     alphaKO     deltaKO         bKO 
"GSX9-1011" "GSX9-1010" "GSX9-1009" "GSX9-1008" "GSX9-1007" "GSX9-1006" 
        aKO         cKO 
"GSX9-1005" "GSX9-1004" 


$`SAGA1309806-WGS`
$`SAGA1309806-WGS`$`TUSWC-462`
 epsilonKO     betaKO    gammaKO    alphaKO    deltaKO        bKO        aKO 
"GSWC-952" "GSWC-951" "GSWC-950" "GSWC-949" "GSWC-948" "GSWC-947" "GSWC-946" 
       cKO 
"GSWC-945" 


$BAMY692420
$BAMY692420$`TUHU2-1904`
        aKO         cKO         bKO     deltaKO     alphaKO     gammaKO 
"GHU2-3644" "GHU2-3643" "GHU2-3642" "GHU2-3641" "GHU2-3640" "GHU2-3639" 
     betaKO   epsilonKO 
"GHU2-3638" "GHU2-3637" 


$BAPH563178
$BAPH563178$`TUHDF-3`
epsilonKO    betaKO   gammaKO   alphaKO   deltaKO       bKO 
 "GHDF-9"  "GHDF-8"  "GHDF-7"  "GHDF-6"  "GHDF-5"  "GHDF-4" 

$BAPH563178$`TUHDF-2`
     cKO      aKO 
"GHDF-3" "GHDF-2" 


$LLAC272623
$LLAC272623$`TUHSH-1119`
        cKO         aKO         bKO     deltaKO     alphaKO     gammaKO 
"GHSH-1875" "GHSH-1874" "GHSH-1873" "GHSH-1872" "GHSH-1871" "GHSH-1870" 
     betaKO   epsilonKO 
"GHSH-1869" "GHSH-1868" 


$BAMY1114958
$BAMY1114958$`TUJW0-1865`
        bKO     deltaKO     alphaKO     gammaKO      betaKO   epsilonKO 
"GJW0-3556" "GJW0-3555" "GJW0-3554" "GJW0-3553" "GJW0-3552" "GJW0-3551" 

$BAMY1114958$`TUJW0-1866`
        cKO 
"GJW0-3557" 

$BAMY1114958$`TUJW0-1867`
        aKO 
```

```
"GJW0-3558"


$BANT261594
$BANT261594$`TUJ7F-3338`
        aKO         cKO         bKO     deltaKO     alphaKO     gammaKO 
"GJ7F-5414" "GJ7F-5413" "GJ7F-5412" "GJ7F-5411" "GJ7F-5410" "GJ7F-5409" 
     betaKO   epsilonKO 
"GJ7F-5408" "GJ7F-5407" 


$`SAGA1318615-WGS`
$`SAGA1318615-WGS`$`TUSWF-500`
  epsilonKO      betaKO     gammaKO     alphaKO     deltaKO         bKO 
"GSWF-1008" "GSWF-1007" "GSWF-1006" "GSWF-1005" "GSWF-1004" "GSWF-1003" 
        aKO         cKO 
"GSWF-1002" "GSWF-1001" 


$`SAGA1231389-WGS`
$`SAGA1231389-WGS`$`TUSWH-398`
 epsilonKO     betaKO    gammaKO    alphaKO    deltaKO        bKO        aKO 
"GSWH-829" "GSWH-828" "GSWH-827" "GSWH-826" "GSWH-825" "GSWH-824" "GSWH-823" 
       cKO 
"GSWH-822" 


$SAUR359787
$SAUR359787$`TUCG4-1107`
        aKO         cKO         bKO     deltaKO     alphaKO     gammaKO 
"GCG4-2252" "GCG4-2251" "GCG4-2250" "GCG4-2249" "GCG4-2248" "GCG4-2247" 
     betaKO   epsilonKO 
"GCG4-2246" "GCG4-2245" 


$STRO369723
$STRO369723$`TUI49-2017`
        aKO         cKO         bKO     deltaKO     alphaKO     gammaKO 
"GI49-3682" "GI49-3681" "GI49-3680" "GI49-3679" "GI49-3678" "GI49-3677" 
     betaKO 
"GI49-3676" 

$STRO369723$`TUI49-2015`
  epsilonKO 
"GI49-3674" 


$SAUR359786
$SAUR359786$`TUJEM-1100`
        aKO         cKO         bKO     deltaKO     alphaKO     gammaKO 
"GJEM-2219" "GJEM-2218" "GJEM-2217" "GJEM-2216" "GJEM-2215" "GJEM-2214" 
     betaKO   epsilonKO 
"GJEM-2213" "GJEM-2212" 
```

```
$SAGA205921
$SAGA205921$`TUHD7-480`
 epsilonKO     betaKO    gammaKO    alphaKO    deltaKO        bKO        aKO 
"GHD7-988" "GHD7-987" "GHD7-986" "GHD7-985" "GHD7-984" "GHD7-983" "GHD7-982" 
       cKO 
"GHD7-981" 



$SALA317655
$SALA317655$`TUHHY-1259`
  epsilonKO      betaKO     gammaKO     alphaKO 
"GHHY-2323" "GHHY-2322" "GHHY-2321" "GHHY-2320" 

$SALA317655$`TUHHY-614`
        cKO         aKO 
"GHHY-1083" "GHHY-1082" 

$SALA317655$`TUHHY-615`
       bKO2        bKO1 
"GHHY-1085" "GHHY-1084" 



$`SALB457425-WGS`
$`SALB457425-WGS`$`TUSXF-866`
        aKO         cKO         bKO     deltaKO     alphaKO     gammaKO 
"GSXF-1467" "GSXF-1466" "GSXF-1465" "GSXF-1464" "GSXF-1463" "GSXF-1462" 
     betaKO   epsilonKO 
"GSXF-1461" "GSXF-1460" 



$SAUR196620
$SAUR196620$`TUJ9Z-1088`
        aKO         cKO         bKO     deltaKO     alphaKO     gammaKO 
"GJ9Z-2102" "GJ9Z-2101" "GJ9Z-2100" "GJ9Z-2099" "GJ9Z-2098" "GJ9Z-2097" 
     betaKO   epsilonKO 
"GJ9Z-2096" "GJ9Z-2095" 



$LASI537021
$LASI537021$`TUHEM-387`
 epsilonKO     betaKO    gammaKO    alphaKO    deltaKO 
"GHEM-616" "GHEM-615" "GHEM-614" "GHEM-613" "GHEM-612" 

$LASI537021$`TUHEM-634`
        aKO         cKO        bKO2        bKO1 
"GHEM-1021" "GHEM-1020" "GHEM-1019" "GHEM-1018" 



$LLAC272622
$LLAC272622$`TUJUG-1265`
        cKO         aKO         bKO     deltaKO     alphaKO     gammaKO 
"GJUG-1939" "GJUG-1938" "GJUG-1937" "GJUG-1936" "GJUG-1935" "GJUG-1934" 
     betaKO   epsilonKO 
```

```
"GJUG-1933" "GJUG-1932"


$`ABAU1096997-WGS`
$`ABAU1096997-WGS`$`TUSEO-173`
 epsilonKO     betaKO    gammaKO    alphaKO    deltaKO        bKO        cKO
"GSEO-207" "GSEO-206" "GSEO-205" "GSEO-204" "GSEO-203" "GSEO-202" "GSEO-201"
       aKO
"GSEO-200"


$ABAU557600
$ABAU557600$`TUKC3-1987`
        aKO         cKO         bKO     deltaKO     alphaKO     gammaKO
"GKC3-3380" "GKC3-3379" "GKC3-3378" "GKC3-3377" "GKC3-3376" "GKC3-3375"
     betaKO   epsilonKO
"GKC3-3374" "GKC3-3373"


$ABAU405416
$ABAU405416$`TUI27-150`
 epsilonKO     betaKO    gammaKO    alphaKO    deltaKO        bKO        cKO
"GI27-185" "GI27-184" "GI27-183" "GI27-182" "GI27-181" "GI27-180" "GI27-179"
       aKO
"GI27-178"


$STER526218
$STER526218$`TUHLD-138`
 epsilonKO     betaKO    gammaKO    alphaKO    deltaKO        bKO        cKO
"GHLD-210" "GHLD-209" "GHLD-208" "GHLD-207" "GHLD-206" "GHLD-205" "GHLD-204"
       aKO
"GHLD-203"


$ABAU980514
$ABAU980514$`TUL7M-156`
 epsilonKO     betaKO    gammaKO    alphaKO    deltaKO        bKO        cKO
"GL7M-178" "GL7M-177" "GL7M-176" "GL7M-175" "GL7M-174" "GL7M-173" "GL7M-172"
       aKO
"GL7M-171"


$`SAGA211110-WGS`
$`SAGA211110-WGS`$`TUSWU-470`
 epsilonKO     betaKO    gammaKO    alphaKO    deltaKO        bKO        aKO
"GSWU-972" "GSWU-971" "GSWU-970" "GSWU-969" "GSWU-968" "GSWU-967" "GSWU-966"
       cKO
"GSWU-965"


$`SANG862971-WGS`
$`SANG862971-WGS`$`TUSWJ-317`
 epsilonKO     betaKO    gammaKO    alphaKO    deltaKO        bKO        aKO
```

"GSWJ-647" "GSWJ-646" "GSWJ-645" "GSWJ-644" "GSWJ-643" "GSWJ-642" "GSWJ-641"
cKO
"GSWJ-640"

$`SANG862970-WGS`
$`SANG862970-WGS`$`TUSWI-309`
epsilonKO betaKO gammaKO alphaKO deltaKO bKO aKO
"GSWI-638" "GSWI-637" "GSWI-636" "GSWI-635" "GSWI-634" "GSWI-633" "GSWI-632"
cKO
"GSWI-631"

$SAUR93061
$SAUR93061$`TUIWJ-1143`
aKO cKO bKO deltaKO alphaKO gammaKO
"GIWJ-2284" "GIWJ-2283" "GIWJ-2282" "GIWJ-2281" "GIWJ-2280" "GIWJ-2279"
betaKO epsilonKO
"GIWJ-2278" "GIWJ-2277"

$SACI679936
$SACI679936$`TUHYT-1350`
aKO cKO bKO deltaKO alphaKO gammaKO
"GHYT-2888" "GHYT-2887" "GHYT-2886" "GHYT-2885" "GHYT-2884" "GHYT-2883"
betaKO epsilonKO
"GHYT-2882" "GHYT-2881"

$`SAPI1276258-WGS`
$`SAPI1276258-WGS`$`TUSVF-22`
epsilonKO betaKO gammaKO alphaKO deltaKO bKO cKO aKO
"GSVF-54" "GSVF-53" "GSVF-52" "GSVF-51" "GSVF-50" "GSVF-49" "GSVF-48" "GSVF-47"

$`LLAC1111678-WGS`
$`LLAC1111678-WGS`$`TUSSS-1070`
cKO aKO bKO deltaKO alphaKO gammaKO
"GSSS-1665" "GSSS-1664" "GSSS-1663" "GSSS-1662" "GSSS-1661" "GSSS-1660"
betaKO epsilonKO
"GSSS-1659" "GSSS-1658"

$SARE391037
$SARE391037$`TUH66-2257`
aKO cKO bKO deltaKO alphaKO gammaKO
"GH66-4065" "GH66-4064" "GH66-4063" "GH66-4062" "GH66-4061" "GH66-4060"
betaKO
"GH66-4059"

$SARE391037$`TUH66-2255`
epsilonKO
"GH66-4057"

```
$SAUR282458
$SAUR282458$`TUJA5-1151`
        aKO         cKO         bKO     deltaKO     alphaKO     gammaKO
"GJA5-2231" "GJA5-2230" "GJA5-2229" "GJA5-2228" "GJA5-2227" "GJA5-2226"
     betaKO   epsilonKO
"GJA5-2225" "GJA5-2224"


$HPYL684950
$HPYL684950$`TULET-461`
       bKO2        bKO1     deltaKO     alphaKO     gammaKO      betaKO
"GLET-1046" "GLET-1045" "GLET-1044" "GLET-1043" "GLET-1042" "GLET-1041"
  epsilonKO
"GLET-1040"

$HPYL684950$`TULET-500`
        cKO
"GLET-1124"

$HPYL684950$`TULET-221`
       aKO
"GLET-505"


$`SENT209261-WGS`
$`SENT209261-WGS`$`TUSUT-1957`
  epsilonKO      betaKO     gammaKO     alphaKO     deltaKO         bKO
"GSUT-3691" "GSUT-3690" "GSUT-3689" "GSUT-3688" "GSUT-3687" "GSUT-3686"
        cKO         aKO
"GSUT-3685" "GSUT-3684"


$`SAUR282459-WGS`
$`SAUR282459-WGS`$`TUSWB-1102`
        aKO         cKO         bKO     deltaKO     alphaKO     gammaKO
"GSWB-2112" "GSWB-2111" "GSWB-2110" "GSWB-2109" "GSWB-2108" "GSWB-2107"
     betaKO   epsilonKO
"GSWB-2106" "GSWB-2105"


$SACI56780
$SACI56780$`TUHXT-351`
epsilonKO1     betaKO   gammaKO1   alphaKO1    deltaKO       bKO1       bKO2
"GHXT-646" "GHXT-645" "GHXT-644" "GHXT-643" "GHXT-642" "GHXT-641" "GHXT-640"

$SACI56780$`TUHXT-1723`
 epsilonKO2        aKO2        cKO1        bKO3    alphaKO2
"GHXT-3171" "GHXT-3168" "GHXT-3167" "GHXT-3166" "GHXT-3165"

$SACI56780$`TUHXT-1722`
   gammaKO2
"GHXT-3164"
```

```
$SACI56780$`TUHXT-407`
     cKO2
"GHXT-768"

$SACI56780$`TUHXT-1316`
      aKO1
"GHXT-2402"


$SAUR158879
$SAUR158879$`TUJCB-1085`
       bKO     deltaKO     alphaKO     gammaKO      betaKO   epsilonKO
"GJCB-2042" "GJCB-2041" "GJCB-2040" "GJCB-2039" "GJCB-2038" "GJCB-2037"

$SAUR158879$`TUJCB-1086`
       aKO         cKO
"GJCB-2044" "GJCB-2043"


$`SAUR585143-WGS`
$`SAUR585143-WGS`$`TUSVO-1062`
       aKO         cKO         bKO     deltaKO     alphaKO     gammaKO
"GSVO-2019" "GSVO-2018" "GSVO-2017" "GSVO-2016" "GSVO-2015" "GSVO-2014"
    betaKO   epsilonKO
"GSVO-2013" "GSVO-2012"


$SAUR1229492
$SAUR1229492$`TULKG-1113`
       aKO         cKO         bKO     deltaKO     alphaKO     gammaKO
"GLKG-2123" "GLKG-2122" "GLKG-2121" "GLKG-2120" "GLKG-2119" "GLKG-2118"
    betaKO   epsilonKO
"GLKG-2117" "GLKG-2116"


$`SAUR1323661-WGS`
$`SAUR1323661-WGS`$`TUSVT-1104`
       aKO         cKO         bKO     deltaKO     alphaKO     gammaKO
"GSVT-2177" "GSVT-2176" "GSVT-2175" "GSVT-2174" "GSVT-2173" "GSVT-2172"
    betaKO   epsilonKO
"GSVT-2171" "GSVT-2170"


$`SAUR1392476-WGS`
$`SAUR1392476-WGS`$`TUSVP-1009`
       bKO     deltaKO     alphaKO     gammaKO      betaKO   epsilonKO
"GSVP-1899" "GSVP-1898" "GSVP-1897" "GSVP-1896" "GSVP-1895" "GSVP-1894"

$`SAUR1392476-WGS`$`TUSVP-1010`
       cKO
"GSVP-1900"

$`SAUR1392476-WGS`$`TUSVP-1011`
       aKO
```

"GSVP-1901"

$`SAUR1305598-WGS`
$`SAUR1305598-WGS`$`TUSVV-1134`
aKO cKO bKO deltaKO alphaKO gammaKO
"GSVV-2147" "GSVV-2146" "GSVV-2145" "GSVV-2144" "GSVV-2143" "GSVV-2142"
betaKO epsilonKO
"GSVV-2141" "GSVV-2140"

$LLAC684738
$LLAC684738$`TUI3F-1177`
cKO aKO bKO deltaKO alphaKO gammaKO
"GI3F-1992" "GI3F-1991" "GI3F-1990" "GI3F-1989" "GI3F-1988" "GI3F-1987"
betaKO epsilonKO
"GI3F-1986" "GI3F-1985"

$`SAUR1193576-WGS`
$`SAUR1193576-WGS`$`TUSVU-1096`
aKO cKO bKO deltaKO alphaKO gammaKO
"GSVU-2139" "GSVU-2138" "GSVU-2137" "GSVU-2136" "GSVU-2135" "GSVU-2134"
betaKO epsilonKO
"GSVU-2133" "GSVU-2132"

$STHE1051074
$STHE1051074$`TULLQ-290`
epsilonKO betaKO gammaKO alphaKO deltaKO bKO aKO
"GLLQ-575" "GLLQ-574" "GLLQ-573" "GLLQ-572" "GLLQ-571" "GLLQ-570" "GLLQ-569"
cKO
"GLLQ-568"

$`SAUR1321369-WGS`
$`SAUR1321369-WGS`$`TUSVR-1117`
aKO cKO bKO deltaKO alphaKO gammaKO
"GSVR-2138" "GSVR-2137" "GSVR-2136" "GSVR-2135" "GSVR-2134" "GSVR-2133"
betaKO epsilonKO
"GSVR-2132" "GSVR-2131"

$`SAUR1194085-WGS`
$`SAUR1194085-WGS`$`TUSVY-1030`
aKO cKO bKO deltaKO alphaKO gammaKO
"GSVY-1934" "GSVY-1933" "GSVY-1932" "GSVY-1931" "GSVY-1930" "GSVY-1929"
betaKO epsilonKO
"GSVY-1928" "GSVY-1927"

$`SAUR1201010-WGS`
$`SAUR1201010-WGS`$`TUSVZ-1054`
aKO cKO bKO deltaKO alphaKO gammaKO

```
"GSVZ-2017" "GSVZ-2016" "GSVZ-2015" "GSVZ-2014" "GSVZ-2013" "GSVZ-2012"
    betaKO   epsilonKO
"GSVZ-2011" "GSVZ-2010"


$SAUR158878
$SAUR158878$`TUJJ5-1152`
       bKO     deltaKO     alphaKO     gammaKO      betaKO   epsilonKO
"GJJ5-2166" "GJJ5-2165" "GJJ5-2164" "GJJ5-2163" "GJJ5-2162" "GJJ5-2161"

$SAUR158878$`TUJJ5-1153`
       aKO         cKO
"GJJ5-2168" "GJJ5-2167"


$SAUR418127
$SAUR418127$`TUJP9-1117`
       aKO         cKO         bKO     deltaKO     alphaKO     gammaKO
"GJP9-2154" "GJP9-2153" "GJP9-2152" "GJP9-2151" "GJP9-2150" "GJP9-2149"
    betaKO   epsilonKO
"GJP9-2148" "GJP9-2147"


$SAUR451516
$SAUR451516$`TUJQ4-1116`
       aKO         cKO         bKO     deltaKO     alphaKO     gammaKO
"GJQ4-2172" "GJQ4-2171" "GJQ4-2170" "GJQ4-2169" "GJQ4-2168" "GJQ4-2167"
    betaKO   epsilonKO
"GJQ4-2166" "GJQ4-2165"


$SACI1051632
$SACI1051632$`TUH78-384`
 epsilonKO     betaKO    gammaKO    alphaKO    deltaKO        bKO
"GH78-815" "GH78-814" "GH78-813" "GH78-812" "GH78-811" "GH78-810"

$SACI1051632$`TUH78-383`
      cKO        aKO
"GH78-809" "GH78-808"


$SAMA326297
$SAMA326297$`TUH0T-5`
       aKO         cKO         bKO     deltaKO     alphaKO     gammaKO
"GH0T-3780" "GH0T-3779" "GH0T-3778" "GH0T-3777" "GH0T-3776" "GH0T-3775"
    betaKO   epsilonKO
"GH0T-3774" "GH0T-3773"


$SBAR760154
$SBAR760154$`TULLZ-226`
 epsilonKO     betaKO    gammaKO    alphaKO
"GLLZ-572" "GLLZ-571" "GLLZ-570" "GLLZ-569"
```

$SBAR760154$`TULLZ-225`
deltaKO bKO2 bKO1
"GLLZ-568" "GLLZ-567" "GLLZ-566"

$SBAR760154$`TULLZ-260`
cKO
"GLLZ-655"

$SBAR760154$`TULLZ-456`
aKO
"GLLZ-1127"

$LLAC416870
$LLAC416870$`TUCDT-1198`
aKO bKO deltaKO alphaKO gammaKO betaKO
"GCDT-1954" "GCDT-1953" "GCDT-1952" "GCDT-1951" "GCDT-1950" "GCDT-1949"
epsilonKO
"GCDT-1948"

$LLAC416870$`TUCDT-1199`
cKO
"GCDT-1955"

$STHE1187956
$STHE1187956$`TULLR-285`
epsilonKO betaKO gammaKO alphaKO deltaKO bKO aKO
"GLLR-530" "GLLR-529" "GLLR-528" "GLLR-527" "GLLR-526" "GLLR-525" "GLLR-524"
cKO
"GLLR-523"

$SBAL693974
$SBAL693974$`TULK2-2604`
aKO cKO bKO deltaKO alphaKO gammaKO
"GLK2-4441" "GLK2-4440" "GLK2-4439" "GLK2-4438" "GLK2-4437" "GLK2-4436"
betaKO epsilonKO
"GLK2-4435" "GLK2-4434"

$SBOY344609
$SBOY344609$`TUI0O-2406`
epsilonKO betaKO gammaKO alphaKO deltaKO bKO
"GI0O-4186" "GI0O-4185" "GI0O-4184" "GI0O-4183" "GI0O-4182" "GI0O-4181"
cKO aKO
"GI0O-4180" "GI0O-4179"

$SBON218493
$SBON218493$`TUJAH-1881`
aKO cKO bKO deltaKO alphaKO gammaKO
"GJAH-3446" "GJAH-3445" "GJAH-3444" "GJAH-3443" "GJAH-3442" "GJAH-3441"
betaKO epsilonKO

```
"GJAH-3440" "GJAH-3439"


$SBIN749414
$SBIN749414$`TUHKA-2206`
       aKO        cKO        bKO     deltaKO     alphaKO
"GHKA-3805" "GHKA-3804" "GHKA-3803" "GHKA-3802" "GHKA-3801"

$SBIN749414$`TUHKA-2205`
   gammaKO     betaKO   epsilonKO
"GHKA-3799" "GHKA-3798" "GHKA-3797"


$SBAL325240
$SBAL325240$`TUCTA-2714`
       aKO        cKO        bKO     deltaKO     alphaKO     gammaKO
"GCTA-4507" "GCTA-4506" "GCTA-4505" "GCTA-4504" "GCTA-4503" "GCTA-4502"
    betaKO   epsilonKO
"GCTA-4501" "GCTA-4500"


$BAPH198804
$BAPH198804$`TUHMG-2`
epsilonKO    betaKO   gammaKO   alphaKO   deltaKO       bKO       cKO       aKO
 "GHMG-9"  "GHMG-8"  "GHMG-7"  "GHMG-6"  "GHMG-5"  "GHMG-4"  "GHMG-3"  "GHMG-2"


$BAST1147128
$BAST1147128$`TUL95-859`
       aKO        cKO        bKO     deltaKO     alphaKO     gammaKO
"GL95-1511" "GL95-1510" "GL95-1509" "GL95-1508" "GL95-1507" "GL95-1506"
    betaKO   epsilonKO
"GL95-1505" "GL95-1504"


$BANT260799
$BANT260799$`TUJAJ-3013`
       aKO        cKO        bKO     deltaKO     alphaKO     gammaKO
"GJAJ-5236" "GJAJ-5235" "GJAJ-5234" "GJAJ-5233" "GJAJ-5232" "GJAJ-5231"
    betaKO   epsilonKO
"GJAJ-5230" "GJAJ-5229"


$BAPH561501
$BAPH561501$`TUHRN-3`
epsilonKO    betaKO   gammaKO   alphaKO   deltaKO       bKO
 "GHRN-9"  "GHRN-8"  "GHRN-7"  "GHRN-6"  "GHRN-5"  "GHRN-4"

$BAPH561501$`TUHRN-2`
    cKO      aKO
"GHRN-3" "GHRN-2"


$SBAL402882
```

```
$SBAL402882$`TUJ99-2641`
       aKO         cKO         bKO     deltaKO     alphaKO     gammaKO 
"GJ99-4511" "GJ99-4510" "GJ99-4509" "GJ99-4508" "GJ99-4507" "GJ99-4506" 
    betaKO   epsilonKO 
"GJ99-4505" "GJ99-4504" 


$APHE930171
$APHE930171$`TUJHZ-1468`
       aKO         cKO         bKO     deltaKO     alphaKO     gammaKO 
"GJHZ-2467" "GJHZ-2466" "GJHZ-2465" "GJHZ-2464" "GJHZ-2463" "GJHZ-2462" 
    betaKO   epsilonKO 
"GJHZ-2461" "GJHZ-2460" 


$LLAC746361
$LLAC746361$`TULFF-1180`
       cKO         aKO         bKO     deltaKO     alphaKO     gammaKO 
"GLFF-1953" "GLFF-1952" "GLFF-1951" "GLFF-1950" "GLFF-1949" "GLFF-1948" 
    betaKO   epsilonKO 
"GLFF-1947" "GLFF-1946" 


$SBAL399599
$SBAL399599$`TUH6B-2793`
       aKO         cKO         bKO     deltaKO     alphaKO     gammaKO 
"GH6B-4657" "GH6B-4656" "GH6B-4655" "GH6B-4654" "GH6B-4653" "GH6B-4652" 
    betaKO   epsilonKO 
"GH6B-4651" "GH6B-4650" 


$SBOY300268
$SBOY300268$`TUJFL-2142`
  epsilonKO      betaKO     gammaKO     alphaKO     deltaKO         bKO 
"GJFL-3753" "GJFL-3752" "GJFL-3751" "GJFL-3750" "GJFL-3749" "GJFL-3748" 
       cKO 
"GJFL-3747" 

$SBOY300268$noTU
aKO 
 NA 


$SBAL407976
$SBAL407976$`TUJ6Y-2657`
       aKO         cKO         bKO     deltaKO     alphaKO     gammaKO 
"GJ6Y-4452" "GJ6Y-4451" "GJ6Y-4450" "GJ6Y-4449" "GJ6Y-4448" "GJ6Y-4447" 
    betaKO   epsilonKO 
"GJ6Y-4446" "GJ6Y-4445" 


$SBAL693970
$SBAL693970$`TULK3-164`
       aKO         cKO         bKO     deltaKO     alphaKO     gammaKO 
```

"GLK3-4664" "GLK3-4663" "GLK3-4662" "GLK3-4661" "GLK3-4660" "GLK3-4659"
betaKO epsilonKO
"GLK3-4658" "GLK3-4657"

$SBAL693973
$SBAL693973$`TUJDB-2711`
aKO cKO bKO deltaKO alphaKO gammaKO
"GJDB-4679" "GJDB-4678" "GJDB-4677" "GJDB-4676" "GJDB-4675" "GJDB-4674"
betaKO epsilonKO
"GJDB-4673" "GJDB-4672"

$`SBON1197719-WGS`
$`SBON1197719-WGS`$`TUSU9-2140`
aKO cKO bKO deltaKO alphaKO gammaKO
"GSU9-4006" "GSU9-4005" "GSU9-4004" "GSU9-4003" "GSU9-4002" "GSU9-4001"
betaKO epsilonKO
"GSU9-4000" "GSU9-3999"

$SSCA680198
$SSCA680198$`TUJ76-1626`
aKO cKO bKO deltaKO alphaKO gammaKO
"GJ76-2774" "GJ76-2773" "GJ76-2772" "GJ76-2771" "GJ76-2770" "GJ76-2769"
betaKO epsilonKO
"GJ76-2768" "GJ76-2767"

$SPAR1114965
$SPAR1114965$`TULL7-379`
gammaKO alphaKO deltaKO bKO aKO cKO
"GLL7-783" "GLL7-782" "GLL7-781" "GLL7-780" "GLL7-779" "GLL7-778"

$SPAR1114965$`TULL7-380`
epsilonKO betaKO
"GLL7-785" "GLL7-784"

$`SCON862969-WGS`
$`SCON862969-WGS`$`TUSWL-331`
epsilonKO betaKO gammaKO alphaKO deltaKO bKO aKO
"GSWL-669" "GSWL-668" "GSWL-667" "GSWL-666" "GSWL-665" "GSWL-664" "GSWL-663"
cKO
"GSWL-662"

$SPYO1010840
$SPYO1010840$`TULLF-317`
epsilonKO betaKO gammaKO alphaKO deltaKO bKO aKO
"GLLF-606" "GLLF-605" "GLLF-604" "GLLF-603" "GLLF-602" "GLLF-601" "GLLF-600"
cKO
"GLLF-599"

```
$SCHL690566
$SCHL690566$`TUJJA-104`
  deltaKO   alphaKO
"GJJA-70" "GJJA-69"

$SCHL690566$`TUJJA-103`
  gammaKO    betaKO
"GJJA-67" "GJJA-66"

$SCHL690566$`TUJJA-102`
epsilonKO
"GJJA-65"

$SCHL690566$`TUJJA-255`
      bKO2       bKO1        cKO        aKO
"GJJA-350" "GJJA-349" "GJJA-348" "GJJA-347"


$LLAC1104322
$LLAC1104322$`TULFE-540`
 epsilonKO     betaKO    gammaKO    alphaKO    deltaKO        bKO        aKO
"GLFE-739" "GLFE-738" "GLFE-737" "GLFE-736" "GLFE-735" "GLFE-734" "GLFE-733"
       cKO
"GLFE-732"


$`SCOL1214242-WGS`
$`SCOL1214242-WGS`$`TUSXH-3115`
  epsilonKO      betaKO     gammaKO     alphaKO     deltaKO         bKO
"GSXH-5085" "GSXH-5084" "GSXH-5083" "GSXH-5082" "GSXH-5081" "GSXH-5080"
        cKO         aKO
"GSXH-5079" "GSXH-5078"


$SCEL448385
$SCEL448385$`TUJ75-5942`
       bKO2        bKO1     deltaKO     alphaKO     gammaKO
"GJ75-9699" "GJ75-9698" "GJ75-9697" "GJ75-9696" "GJ75-9695"

$SCEL448385$`TUJ75-2812`
  epsilonKO      betaKO
"GJ75-4609" "GJ75-4608"

$SCEL448385$`TUJ75-5080`
        cKO         aKO
"GJ75-8275" "GJ75-8274"


$SCAN929556
$SCAN929556$`TULKC-2080`
        cKO         bKO     deltaKO     alphaKO     gammaKO
"GLKC-3296" "GLKC-3295" "GLKC-3294" "GLKC-3293" "GLKC-3292"
```

$SCAN929556$`TULKC-1755`
epsilonKO betaKO
"GLKC-2790" "GLKC-2789"

$SCAN929556$`TULKC-2081`
aKO
"GLKC-3297"

$`SCON696216-WGS`
$`SCON696216-WGS`$`TUSWM-321`
epsilonKO betaKO gammaKO alphaKO deltaKO bKO aKO
"GSWM-649" "GSWM-648" "GSWM-647" "GSWM-646" "GSWM-645" "GSWM-644" "GSWM-643"
cKO
"GSWM-642"

$`SCON862968-WGS`
$`SCON862968-WGS`$`TUSX0-321`
epsilonKO betaKO gammaKO alphaKO deltaKO bKO aKO
"GSX0-649" "GSX0-648" "GSX0-647" "GSX0-646" "GSX0-645" "GSX0-644" "GSX0-643"
cKO
"GSX0-642"

$SPAR760570
$SPAR760570$`TUI5E-108`
epsilonKO betaKO gammaKO alphaKO deltaKO bKO aKO
"GI5E-230" "GI5E-229" "GI5E-228" "GI5E-227" "GI5E-226" "GI5E-225" "GI5E-224"
cKO
"GI5E-223"

$`SCHR1276227-WGS`
$`SCHR1276227-WGS`$`TUSVG-38`
epsilonKO betaKO gammaKO alphaKO deltaKO bKO cKO aKO
"GSVG-92" "GSVG-91" "GSVG-90" "GSVG-89" "GSVG-88" "GSVG-87" "GSVG-86" "GSVG-85"

$`SCYA111780-WGS`
$`SCYA111780-WGS`$`TUSVN-2499`
aKO cKO bKO2 bKO1 deltaKO alphaKO
"GSVN-3275" "GSVN-3274" "GSVN-3273" "GSVN-3272" "GSVN-3271" "GSVN-3270"
gammaKO
"GSVN-3269"

$`SCYA111780-WGS`$`TUSVN-2286`
epsilonKO betaKO
"GSVN-2970" "GSVN-2969"

$SPYO487215
$SPYO487215$`TULLD-320`
epsilonKO betaKO gammaKO alphaKO deltaKO bKO aKO

```
"GLLD-616" "GLLD-615" "GLLD-614" "GLLD-613" "GLLD-612" "GLLD-611" "GLLD-610"
       cKO
"GLLD-609"


$SCAT1003195
$SCAT1003195$`TUJCM-3494`
  epsilonKO      betaKO     gammaKO     alphaKO     deltaKO         bKO
"GJCM-4232" "GJCM-4231" "GJCM-4230" "GJCM-4229" "GJCM-4228" "GJCM-4227"
        cKO         aKO
"GJCM-4226" "GJCM-4225"


$`SCEL1254432-WGS`
$`SCEL1254432-WGS`$`TUSVD-6508`
        bKO2         bKO1      deltaKO      alphaKO      gammaKO
"GSVD-10499" "GSVD-10498" "GSVD-10497" "GSVD-10496" "GSVD-10495"

$`SCEL1254432-WGS`$`TUSVD-8092`
    betaKO
"GSVD-772"

$`SCEL1254432-WGS`$`TUSVD-3288`
  epsilonKO
"GSVD-5408"

$`SCEL1254432-WGS`$`TUSVD-5647`
        cKO         aKO
"GSVD-9128" "GSVD-9127"


$LLAC929102
$LLAC929102$`TULFG-1114`
        cKO         aKO         bKO     deltaKO     alphaKO     gammaKO
"GLFG-1744" "GLFG-1743" "GLFG-1742" "GLFG-1741" "GLFG-1740" "GLFG-1739"

$LLAC929102$`TULFG-1113`
     betaKO   epsilonKO
"GLFG-1737" "GLFG-1736"


$`SDYS617121-WGS`
$`SDYS617121-WGS`$`TUSX4-380`
 epsilonKO     betaKO    gammaKO    alphaKO    deltaKO        bKO        aKO
"GSX4-698" "GSX4-697" "GSX4-696" "GSX4-695" "GSX4-694" "GSX4-693" "GSX4-692"

$`SDYS617121-WGS`$noTU
cKO
 NA


$SDYS759913
$SDYS759913$`TULKZ-401`
 epsilonKO     betaKO    gammaKO    alphaKO    deltaKO        bKO        aKO
```

```
"GLKZ-771" "GLKZ-770" "GLKZ-769" "GLKZ-768" "GLKZ-767" "GLKZ-766" "GLKZ-765"
       cKO
"GLKZ-764"


$SDYS663954
$SDYS663954$`TULL0-407`
 epsilonKO     betaKO    gammaKO    alphaKO    deltaKO        bKO        aKO
"GLL0-791" "GLL0-790" "GLL0-789" "GLL0-788" "GLL0-787" "GLL0-786" "GLL0-785"
       cKO
"GLL0-784"


$`SDIM1276221-WGS`
$`SDIM1276221-WGS`$`TUSVI-23`
epsilonKO    betaKO   gammaKO   alphaKO   deltaKO       bKO       cKO       aKO
"GSVI-57" "GSVI-56" "GSVI-55" "GSVI-54" "GSVI-53" "GSVI-52" "GSVI-51" "GSVI-50"


$SDEL525898
$SDEL525898$`TUHVA-218`
 epsilonKO     betaKO    gammaKO    alphaKO    deltaKO       bKO2       bKO1
"GHVA-537" "GHVA-536" "GHVA-535" "GHVA-534" "GHVA-533" "GHVA-532" "GHVA-531"

$SDEL525898$`TUHVA-254`
       cKO
"GHVA-622"

$SDEL525898$`TUHVA-471`
        aKO
"GHVA-1163"


$SDEN318161
$SDEN318161$`TUHKQ-2290`
        aKO         cKO         bKO     deltaKO     alphaKO     gammaKO
"GHKQ-3885" "GHKQ-3884" "GHKQ-3883" "GHKQ-3882" "GHKQ-3881" "GHKQ-3880"
     betaKO   epsilonKO
"GHKQ-3879" "GHKQ-3878"


$`SDEN1163617-WGS`
$`SDEN1163617-WGS`$`TUSY7-362`
   betaKO1 epsilonKO1       aKO1       cKO1       bKO1   alphaKO1   gammaKO1
"GSY7-700" "GSY7-699" "GSY7-696" "GSY7-695" "GSY7-694" "GSY7-693" "GSY7-692"

$`SDEN1163617-WGS`$`TUSY7-1513`
       aKO2        cKO2        bKO2     deltaKO    alphaKO2    gammaKO2
"GSY7-3026" "GSY7-3025" "GSY7-3024" "GSY7-3023" "GSY7-3022" "GSY7-3021"
    betaKO2  epsilonKO2
"GSY7-3020" "GSY7-3019"

$`SDEN1163617-WGS`$`TUSY7-23`
    betaKO3  epsilonKO3        aKO3        cKO3        bKO3    alphaKO3
```

"GSY7-3073" "GSY7-3072" "GSY7-3070" "GSY7-3069" "GSY7-3068" "GSY7-3067"
gammaKO3
"GSY7-3066"

$SAUT563040
$SAUT563040$`TUH0V-284`
epsilonKO betaKO gammaKO alphaKO deltaKO bKO2 bKO1
"GH0V-800" "GH0V-799" "GH0V-798" "GH0V-797" "GH0V-796" "GH0V-795" "GH0V-794"

$SAUT563040$`TUH0V-657`
cKO
"GH0V-1827"

$SAUT563040$`TUH0V-524`
aKO
"GH0V-1469"

$SDYS486410
$SDYS486410$`TUHH8-398`
epsilonKO betaKO gammaKO alphaKO deltaKO bKO aKO
"GHH8-729" "GHH8-728" "GHH8-727" "GHH8-726" "GHH8-725" "GHH8-724" "GHH8-723"
cKO
"GHH8-722"

$SPSE984892
$SPSE984892$`TULKW-378`
epsilonKO betaKO gammaKO alphaKO deltaKO bKO cKO
"GLKW-712" "GLKW-711" "GLKW-710" "GLKW-709" "GLKW-708" "GLKW-707" "GLKW-706"
aKO
"GLKW-705"

$`SDAV1214101-WGS`
$`SDAV1214101-WGS`$`TUSXI-1726`
aKO cKO bKO deltaKO alphaKO gammaKO
"GSXI-3059" "GSXI-3058" "GSXI-3057" "GSXI-3056" "GSXI-3055" "GSXI-3054"
betaKO epsilonKO
"GSXI-3053" "GSXI-3052"

$LMES203120
$LMES203120$`TUJ8T-945`
cKO bKO deltaKO alphaKO gammaKO betaKO
"GJ8T-1874" "GJ8T-1873" "GJ8T-1872" "GJ8T-1871" "GJ8T-1870" "GJ8T-1869"
epsilonKO
"GJ8T-1868"

$LMES203120$`TUJ8T-946`
aKO
"GJ8T-1875"

$SDYS300267
$SDYS300267$`TUJEW-2450`
epsilonKO betaKO gammaKO alphaKO deltaKO bKO
"GJEW-4013" "GJEW-4012" "GJEW-4011" "GJEW-4010" "GJEW-4009" "GJEW-4008"
cKO aKO
"GJEW-4007" "GJEW-4006"

$`SDYS754093-WGS`
$`SDYS754093-WGS`$`TUSV4-2731`
epsilonKO betaKO gammaKO alphaKO deltaKO bKO
"GSV4-5338" "GSV4-5337" "GSV4-5336" "GSV4-5335" "GSV4-5334" "GSV4-5333"
cKO aKO
"GSV4-5332" "GSV4-5331"

$SENT454166
$SENT454166$`TUHBA-2140`
aKO cKO bKO deltaKO alphaKO gammaKO
"GHBA-4067" "GHBA-4066" "GHBA-4065" "GHBA-4064" "GHBA-4063" "GHBA-4062"
betaKO epsilonKO
"GHBA-4061" "GHBA-4060"

$SENT909946
$SENT909946$`TULJU-2207`
aKO cKO bKO deltaKO alphaKO gammaKO
"GLJU-4025" "GLJU-4024" "GLJU-4023" "GLJU-4022" "GLJU-4021" "GLJU-4020"
betaKO epsilonKO
"GLJU-4019" "GLJU-4018"

$SENT321314
$SENT321314$`TUJCS-2231`
aKO cKO bKO deltaKO alphaKO gammaKO
"GJCS-3947" "GJCS-3946" "GJCS-3945" "GJCS-3944" "GJCS-3943" "GJCS-3942"
betaKO epsilonKO
"GJCS-3941" "GJCS-3940"

$SEND1199245
$SEND1199245$`TULJX-398`
aKO cKO bKO deltaKO alphaKO gammaKO betaKO
"GLJX-646" "GLJX-645" "GLJX-644" "GLJX-643" "GLJX-642" "GLJX-641" "GLJX-640"
epsilonKO
"GLJX-639"

$SUBE218495
$SUBE218495$`TUJ7D-358`
epsilonKO betaKO gammaKO alphaKO deltaKO bKO aKO
"GJ7D-716" "GJ7D-715" "GJ7D-714" "GJ7D-713" "GJ7D-712" "GJ7D-711" "GJ7D-710"
cKO

```
"GJ7D-709"


$`BAUS1094489-WGS`
$`BAUS1094489-WGS`$`TUSGI-690`
   deltaKO     alphaKO     gammaKO      betaKO   epsilonKO
"GSGI-1200" "GSGI-1199" "GSGI-1198" "GSGI-1197" "GSGI-1196"

$`BAUS1094489-WGS`$`TUSGI-203`
     bKO2       bKO1        cKO        aKO
"GSGI-338" "GSGI-337" "GSGI-336" "GSGI-335"


$BAVI360910
$BAVI360910$`TUCKI-1616`
       aKO         cKO         bKO     deltaKO     alphaKO     gammaKO
"GCKI-3296" "GCKI-3295" "GCKI-3294" "GCKI-3293" "GCKI-3292" "GCKI-3291"
    betaKO   epsilonKO
"GCKI-3290" "GCKI-3289"


$BAPH713600
$BAPH713600$`TUL9T-2`
epsilonKO    betaKO   gammaKO   alphaKO   deltaKO       bKO       cKO       aKO
 "GL9T-9"  "GL9T-8"  "GL9T-7"  "GL9T-6"  "GL9T-5"  "GL9T-4"  "GL9T-3"  "GL9T-2"


$BANT768494
$BANT768494$`TUL8F-3447`
       aKO         cKO         bKO     deltaKO     alphaKO     gammaKO
"GL8F-5419" "GL8F-5418" "GL8F-5417" "GL8F-5416" "GL8F-5415" "GL8F-5414"
    betaKO   epsilonKO
"GL8F-5413" "GL8F-5412"


$`LMON265669-WGS`
$`LMON265669-WGS`$`TUSPV-53`
epsilonKO1    betaKO1   gammaKO1   alphaKO1   deltaKO1       cKO1
"GSPV-111" "GSPV-110" "GSPV-109" "GSPV-108" "GSPV-107" "GSPV-106"

$`LMON265669-WGS`$`TUSPV-1341`
       aKO        cKO2         bKO    deltaKO2    alphaKO2    gammaKO2
"GSPV-2586" "GSPV-2585" "GSPV-2584" "GSPV-2583" "GSPV-2582" "GSPV-2581"
   betaKO2  epsilonKO2
"GSPV-2580" "GSPV-2579"


$SENT439851
$SENT439851$`TUH2Z-2234`
       aKO         cKO         bKO     deltaKO     alphaKO     gammaKO
"GH2Z-4211" "GH2Z-4210" "GH2Z-4209" "GH2Z-4208" "GH2Z-4207" "GH2Z-4206"
    betaKO   epsilonKO
"GH2Z-4205" "GH2Z-4204"
```

```
$SENT423368
$SENT423368$`TUHJB-2242`
       aKO        cKO        bKO    deltaKO    alphaKO    gammaKO 
"GHJB-4118" "GHJB-4117" "GHJB-4116" "GHJB-4115" "GHJB-4114" "GHJB-4113" 
    betaKO   epsilonKO 
"GHJB-4112" "GHJB-4111" 


$`SENT1173427-WGS`
$`SENT1173427-WGS`$`TUSUQ-10`
epsilonKO    betaKO   gammaKO   alphaKO   deltaKO       bKO       cKO       aKO 
"GSUQ-26" "GSUQ-25" "GSUQ-24" "GSUQ-23" "GSUQ-22" "GSUQ-21" "GSUQ-20" "GSUQ-19" 


$`SENT1271863-WGS`
$`SENT1271863-WGS`$`TUSUD-266`
      aKO       cKO       bKO    deltaKO    alphaKO    gammaKO     betaKO 
"GSUD-137" "GSUD-136" "GSUD-135" "GSUD-134" "GSUD-133" "GSUD-132" "GSUD-131" 
 epsilonKO 
"GSUD-130" 


$`SENT1124936-WGS`
$`SENT1124936-WGS`$`TUSUF-604`
      aKO       cKO       bKO    deltaKO    alphaKO    gammaKO     betaKO 
"GSUF-982" "GSUF-981" "GSUF-980" "GSUF-979" "GSUF-978" "GSUF-977" "GSUF-976" 
 epsilonKO 
"GSUF-975" 


$SAUR889933
$SAUR889933$`TULKJ-1060`
       aKO        cKO        bKO    deltaKO    alphaKO    gammaKO 
"GLKJ-1965" "GLKJ-1964" "GLKJ-1963" "GLKJ-1962" "GLKJ-1961" "GLKJ-1960" 
    betaKO   epsilonKO 
"GLKJ-1959" "GLKJ-1958" 


$`SENT866913-WGS`
$`SENT866913-WGS`$`TUSUR-150`
 epsilonKO     betaKO    gammaKO    alphaKO    deltaKO       bKO       cKO 
"GSUR-313" "GSUR-312" "GSUR-311" "GSUR-310" "GSUR-309" "GSUR-308" "GSUR-307" 
      aKO 
"GSUR-306" 


$`SENT1298917-WGS`
$`SENT1298917-WGS`$`TUSUM-1923`
  epsilonKO      betaKO     gammaKO     alphaKO     deltaKO        bKO 
"GSUM-3635" "GSUM-3634" "GSUM-3633" "GSUM-3632" "GSUM-3631" "GSUM-3630" 
       cKO        aKO 
"GSUM-3629" "GSUM-3628" 
```

```
$SENT1008297
$SENT1008297$`TULJT-2135`
        aKO         cKO         bKO     deltaKO     alphaKO     gammaKO 
"GLJT-3783" "GLJT-3782" "GLJT-3781" "GLJT-3780" "GLJT-3779" "GLJT-3778" 
     betaKO   epsilonKO 
"GLJT-3777" "GLJT-3776" 


$SENT550538
$SENT550538$`TUJ93-1911`
   alphaKO    deltaKO        bKO 
"GJ93-3529" "GJ93-3528" "GJ93-3527" 

$SENT550538$`TUJ93-1912`
  epsilonKO      betaKO     gammaKO 
"GJ93-3532" "GJ93-3531" "GJ93-3530" 

$SENT550538$`TUJ93-1910`
        cKO 
"GJ93-3526" 

$SENT550538$`TUJ93-1909`
        aKO 
"GJ93-3525" 


$`SENT1225522-WGS`
$`SENT1225522-WGS`$`TUSUE-2007`
  epsilonKO      betaKO     gammaKO     alphaKO     deltaKO         bKO 
"GSUE-3773" "GSUE-3772" "GSUE-3771" "GSUE-3770" "GSUE-3769" "GSUE-3768" 
        cKO         aKO 
"GSUE-3767" "GSUE-3766" 


$LMON393126
$LMON393126$`TULFU-50`
epsilonKO1    betaKO1   gammaKO1   alphaKO1   deltaKO1       cKO1 
 "GLFU-95"  "GLFU-94"  "GLFU-93"  "GLFU-92"  "GLFU-91"  "GLFU-90" 

$LMON393126$`TULFU-1353`
        aKO        cKO2         bKO    deltaKO2    alphaKO2    gammaKO2 
"GLFU-2653" "GLFU-2652" "GLFU-2651" "GLFU-2650" "GLFU-2649" "GLFU-2648" 
    betaKO2  epsilonKO2 
"GLFU-2647" "GLFU-2646" 


$SENT454169
$SENT454169$`TUHYG-2242`
        aKO         cKO         bKO     deltaKO     alphaKO     gammaKO 
"GHYG-4176" "GHYG-4175" "GHYG-4174" "GHYG-4173" "GHYG-4172" "GHYG-4171" 
     betaKO   epsilonKO 
"GHYG-4170" "GHYG-4169" 
```

$SENT476213
$SENT476213$`TUH8J-2223`
bKO deltaKO alphaKO
"GH8J-4016" "GH8J-4015" "GH8J-4014"

$SENT476213$`TUH8J-2222`
gammaKO betaKO epsilonKO
"GH8J-4013" "GH8J-4012" "GH8J-4011"

$SENT476213$`TUH8J-2224`
cKO
"GH8J-4017"

$SENT476213$`TUH8J-2225`
aKO
"GH8J-4018"

$SENT990282
$SENT990282$`TUHYP-2117`
aKO cKO bKO deltaKO alphaKO gammaKO
"GHYP-3859" "GHYP-3858" "GHYP-3857" "GHYP-3856" "GHYP-3855" "GHYP-3854"
betaKO epsilonKO
"GHYP-3853" "GHYP-3852"

$SENT554290
$SENT554290$`TUJDA-1986`
aKO cKO bKO deltaKO alphaKO gammaKO
"GJDA-3720" "GJDA-3719" "GJDA-3718" "GJDA-3717" "GJDA-3716" "GJDA-3715"
betaKO epsilonKO
"GJDA-3714" "GJDA-3713"

$`SAUR1155084-WGS`
$`SAUR1155084-WGS`$`TUSWN-1053`
aKO cKO bKO deltaKO alphaKO gammaKO
"GSWN-2039" "GSWN-2038" "GSWN-2037" "GSWN-2036" "GSWN-2035" "GSWN-2034"
betaKO epsilonKO
"GSWN-2033" "GSWN-2032"

$SENT1081093
$SENT1081093$`TUJVO-2018`
epsilonKO betaKO gammaKO alphaKO deltaKO bKO
"GJVO-3786" "GJVO-3785" "GJVO-3784" "GJVO-3783" "GJVO-3782" "GJVO-3781"
cKO aKO
"GJVO-3780" "GJVO-3779"

$SENT718274
$SENT718274$`TUI6S-2210`
aKO cKO bKO deltaKO alphaKO gammaKO

```
"GI6S-4101" "GI6S-4100" "GI6S-4099" "GI6S-4098" "GI6S-4097" "GI6S-4096"
    betaKO   epsilonKO
"GI6S-4095" "GI6S-4094"


$`SENT1320309-WGS`
$`SENT1320309-WGS`$`TUSUB-2310`
       aKO         cKO         bKO     deltaKO     alphaKO     gammaKO
"GSUB-4084" "GSUB-4083" "GSUB-4082" "GSUB-4081" "GSUB-4080" "GSUB-4079"
    betaKO   epsilonKO
"GSUB-4078" "GSUB-4077"


$`SENT85569-WGS`
$`SENT85569-WGS`$`TUSUW-2248`
       aKO         bKO     deltaKO     alphaKO     gammaKO      betaKO
"GSUW-3976" "GSUW-3975" "GSUW-3974" "GSUW-3973" "GSUW-3972" "GSUW-3971"
  epsilonKO
"GSUW-3970"

$`SENT85569-WGS`$noTU
cKO
 NA


$`SENT1064551-WGS`
$`SENT1064551-WGS`$`TUSUN-1986`
       aKO         cKO         bKO     deltaKO     alphaKO     gammaKO
"GSUN-3752" "GSUN-3751" "GSUN-3750" "GSUN-3749" "GSUN-3748" "GSUN-3747"
    betaKO   epsilonKO
"GSUN-3746" "GSUN-3745"


$`SENT1271864-WGS`
$`SENT1271864-WGS`$`TUSUH-2396`
       aKO         cKO         bKO     deltaKO     alphaKO     gammaKO
"GSUH-4349" "GSUH-4348" "GSUH-4347" "GSUH-4346" "GSUH-4345" "GSUH-4344"
    betaKO   epsilonKO
"GSUH-4343" "GSUH-4342"


$LAMY695562
$LAMY695562$`TULF1-466`
 epsilonKO     betaKO    gammaKO    alphaKO    deltaKO        bKO        cKO
"GLF1-806" "GLF1-805" "GLF1-804" "GLF1-803" "GLF1-802" "GLF1-801" "GLF1-800"
       aKO
"GLF1-799"


$LMON393130
$LMON393130$`TULFV-49`
epsilonKO1    betaKO1   gammaKO1   alphaKO1   deltaKO1       cKO1
"GLFV-104" "GLFV-103" "GLFV-102" "GLFV-101" "GLFV-100"  "GLFV-99"
```

```
$LMON393130$`TULFV-1319`
        aKO        cKO2         bKO    deltaKO2    alphaKO2    gammaKO2 
"GLFV-2647" "GLFV-2646" "GLFV-2645" "GLFV-2644" "GLFV-2643" "GLFV-2642" 
    betaKO2  epsilonKO2 
"GLFV-2641" "GLFV-2640" 


$`SENT1267753-WGS`
$`SENT1267753-WGS`$`TUSUY-1537`
  epsilonKO      betaKO     gammaKO     alphaKO     deltaKO         bKO 
"GSUY-2857" "GSUY-2856" "GSUY-2855" "GSUY-2854" "GSUY-2853" "GSUY-2852" 
        cKO         aKO 
"GSUY-2851" "GSUY-2850" 


$`SENT877468-WGS`
$`SENT877468-WGS`$`TUSW2-4`
 alphaKO  deltaKO      bKO      cKO      aKO 
"GSW2-9" "GSW2-8" "GSW2-7" "GSW2-6" "GSW2-5" 

$`SENT877468-WGS`$`TUSW2-5`
epsilonKO    betaKO   gammaKO 
"GSW2-13" "GSW2-12" "GSW2-11" 


$ABUT367737
$ABUT367737$`TUHWO-646`
       bKO2        bKO1     deltaKO     alphaKO     gammaKO      betaKO 
"GHWO-1598" "GHWO-1597" "GHWO-1596" "GHWO-1595" "GHWO-1594" "GHWO-1593" 
  epsilonKO 
"GHWO-1592" 

$ABUT367737$`TUHWO-705`
        cKO 
"GHWO-1746" 

$ABUT367737$`TUHWO-813`
        aKO 
"GHWO-2028" 


$PZUC450851
$PZUC450851$`TUHUG-315`
 epsilonKO     betaKO    gammaKO    alphaKO    deltaKO 
"GHUG-242" "GHUG-241" "GHUG-240" "GHUG-239" "GHUG-238" 

$PZUC450851$`TUHUG-503`
       cKO 
"GHUG-590" 

$PZUC450851$`TUHUG-502`
       aKO 
"GHUG-589" 
```

$PZUC450851$`TUHUG-504`
bKO2 bKO1
"GHUG-592" "GHUG-591"

$SAUR685039
$SAUR685039$`TULKK-1119`
aKO cKO bKO deltaKO alphaKO gammaKO
"GLKK-2146" "GLKK-2145" "GLKK-2144" "GLKK-2143" "GLKK-2142" "GLKK-2141"
betaKO epsilonKO
"GLKK-2140" "GLKK-2139"

$`SENT568709-WGS`
$`SENT568709-WGS`$`TUSW6-2076`
aKO cKO bKO deltaKO alphaKO gammaKO
"GSW6-3815" "GSW6-3814" "GSW6-3813" "GSW6-3812" "GSW6-3811" "GSW6-3810"
betaKO epsilonKO
"GSW6-3809" "GSW6-3808"

$`SENT527001-WGS`
$`SENT527001-WGS`$`TUSUS-1931`
epsilonKO betaKO gammaKO alphaKO deltaKO bKO
"GSUS-3613" "GSUS-3612" "GSUS-3611" "GSUS-3610" "GSUS-3609" "GSUS-3608"
cKO aKO
"GSUS-3607" "GSUS-3606"

$SENT588858
$SENT588858$`TUJDZ-2323`
aKO cKO bKO deltaKO alphaKO gammaKO
"GJDZ-4671" "GJDZ-4670" "GJDZ-4669" "GJDZ-4668" "GJDZ-4667" "GJDZ-4666"
betaKO epsilonKO
"GJDZ-4665" "GJDZ-4664"

$SEPI176280
$SEPI176280$`TUCDG-947`
aKO cKO bKO deltaKO alphaKO gammaKO
"GCDG-1756" "GCDG-1755" "GCDG-1754" "GCDG-1753" "GCDG-1752" "GCDG-1751"
betaKO epsilonKO
"GCDG-1750" "GCDG-1749"

$SEQU40041
$SEQU40041$`TUC8B-645`
cKO aKO bKO deltaKO alphaKO gammaKO
"GC8B-1256" "GC8B-1255" "GC8B-1254" "GC8B-1253" "GC8B-1252" "GC8B-1251"
betaKO epsilonKO
"GC8B-1250" "GC8B-1249"

$SEPI176279

```
$SEPI176279$`TUJJB-920`
       aKO        cKO        bKO     deltaKO     alphaKO     gammaKO
"GJJB-1784" "GJJB-1783" "GJJB-1782" "GJJB-1781" "GJJB-1780" "GJJB-1779"
    betaKO   epsilonKO
"GJJB-1778" "GJJB-1777"


$SENT882884
$SENT882884$`TUJ8H-1950`
  epsilonKO      betaKO     gammaKO     alphaKO     deltaKO        bKO
"GJ8H-3781" "GJ8H-3780" "GJ8H-3779" "GJ8H-3778" "GJ8H-3777" "GJ8H-3776"
       cKO        aKO
"GJ8H-3775" "GJ8H-3774"


$`SESP1179773-WGS`
$`SESP1179773-WGS`$`TUSU7-3783`
      aKO2        cKO        bKO     deltaKO     alphaKO     gammaKO
"GSU7-7311" "GSU7-7310" "GSU7-7309" "GSU7-7308" "GSU7-7307" "GSU7-7306"
    betaKO
"GSU7-7305"

$`SESP1179773-WGS`$`TUSU7-3782`
  epsilonKO
"GSU7-7302"

$`SESP1179773-WGS`$`TUSU7-5076`
      aKO1
"GSU7-4124"


$LMON563174
$LMON563174$`TULFX-55`
epsilonKO1    betaKO1   gammaKO1   alphaKO1   deltaKO1       cKO1
"GLFX-127" "GLFX-126" "GLFX-125" "GLFX-124" "GLFX-123" "GLFX-122"

$LMON563174$`TULFX-1322`
       aKO       cKO2        bKO    deltaKO2    alphaKO2    gammaKO2
"GLFX-2599" "GLFX-2598" "GLFX-2597" "GLFX-2596" "GLFX-2595" "GLFX-2594"
   betaKO2  epsilonKO2
"GLFX-2593" "GLFX-2592"


$SENT550537
$SENT550537$`TUJFI-2011`
       aKO        cKO        bKO     deltaKO     alphaKO     gammaKO
"GJFI-3737" "GJFI-3736" "GJFI-3735" "GJFI-3734" "GJFI-3733" "GJFI-3732"
    betaKO   epsilonKO
"GJFI-3731" "GJFI-3730"


$SAUR985006
$SAUR985006$`TULKO-1027`
       aKO        cKO        bKO     deltaKO     alphaKO     gammaKO
```

```
"GLKO-2009" "GLKO-2008" "GLKO-2007" "GLKO-2006" "GLKO-2005" "GLKO-2004"
    betaKO   epsilonKO
"GLKO-2003" "GLKO-2002"


$`SENT1271862-WGS`
$`SENT1271862-WGS`$`TUSV9-2331`
  epsilonKO      betaKO     gammaKO     alphaKO     deltaKO         bKO
"GSV9-4221" "GSV9-4220" "GSV9-4219" "GSV9-4218" "GSV9-4217" "GSV9-4216"
        cKO         aKO
"GSV9-4215" "GSV9-4214"


$`SENT1171376-WGS`
$`SENT1171376-WGS`$`TUSV8-2159`
        aKO         cKO         bKO     deltaKO     alphaKO     gammaKO
"GSV8-3960" "GSV8-3959" "GSV8-3958" "GSV8-3957" "GSV8-3956" "GSV8-3955"
     betaKO   epsilonKO
"GSV8-3954" "GSV8-3953"


$SEQU553482
$SEQU553482$`TUJOY-447`
 epsilonKO     betaKO    gammaKO    alphaKO    deltaKO        bKO        aKO
"GJOY-928" "GJOY-927" "GJOY-926" "GJOY-925" "GJOY-924" "GJOY-923" "GJOY-922"
       cKO
"GJOY-921"


$SENT568708
$SENT568708$`TUJDP-2099`
        aKO         bKO     deltaKO     alphaKO     gammaKO      betaKO
"GJDP-3912" "GJDP-3911" "GJDP-3910" "GJDP-3909" "GJDP-3908" "GJDP-3907"
  epsilonKO
"GJDP-3906"

$SENT568708$noTU
cKO
 NA


$SENT439843
$SENT439843$`TUHHR-2187`
        aKO         cKO         bKO     deltaKO     alphaKO     gammaKO
"GHHR-4554" "GHHR-2676" "GHHR-3210" "GHHR-2008" "GHHR-2042" "GHHR-3677"
     betaKO   epsilonKO
 "GHHR-406"  "GHHR-931"


$SENT1132507
$SENT1132507$`TUKDO-2161`
  epsilonKO      betaKO     gammaKO     alphaKO     deltaKO         bKO
"GKDO-3847" "GKDO-3846" "GKDO-3845" "GKDO-3844" "GKDO-3843" "GKDO-3842"
        cKO         aKO
```

"GKDO-3841" "GKDO-3840"

$SENT216597
$SENT216597$`TUJB7-2088`
aKO cKO bKO deltaKO alphaKO gammaKO
"GJB7-3899" "GJB7-3898" "GJB7-3897" "GJB7-3896" "GJB7-3895" "GJB7-3894"
betaKO epsilonKO
"GJB7-3893" "GJB7-3892"

$SEQU552526
$SEQU552526$`TUH4P-432`
gammaKO alphaKO deltaKO bKO aKO cKO
"GH4P-824" "GH4P-823" "GH4P-822" "GH4P-821" "GH4P-820" "GH4P-819"

$SEQU552526$`TUH4P-433`
epsilonKO betaKO
"GH4P-826" "GH4P-825"

$BAMY326423
$BAMY326423$`TUCM4-1836`
aKO cKO bKO deltaKO alphaKO gammaKO
"GCM4-3400" "GCM4-3399" "GCM4-3398" "GCM4-3397" "GCM4-3396" "GCM4-3395"
betaKO epsilonKO
"GCM4-3394" "GCM4-3393"

$LMES1107880
$LMES1107880$`TULFP-929`
cKO bKO deltaKO alphaKO gammaKO betaKO
"GLFP-1682" "GLFP-1681" "GLFP-1680" "GLFP-1679" "GLFP-1678" "GLFP-1677"
epsilonKO
"GLFP-1676"

$LMES1107880$`TULFP-930`
aKO
"GLFP-1683"

$SAUR523796
$SAUR523796$`TULKR-1136`
aKO cKO bKO deltaKO alphaKO gammaKO
"GLKR-2151" "GLKR-2150" "GLKR-2149" "GLKR-2148" "GLKR-2147" "GLKR-2146"
betaKO epsilonKO
"GLKR-2145" "GLKR-2144"

$BAMY999891
$BAMY999891$`TUL8G-1981`
aKO cKO bKO deltaKO alphaKO gammaKO
"GL8G-3777" "GL8G-3776" "GL8G-3775" "GL8G-3774" "GL8G-3773" "GL8G-3772"
betaKO epsilonKO

"GL8G-3771" "GL8G-3770"

$`SEQU1051072-WGS`
$`SEQU1051072-WGS`$`TUSWO-482`
epsilonKO betaKO gammaKO alphaKO deltaKO bKO aKO
"GSWO-897" "GSWO-896" "GSWO-895" "GSWO-894" "GSWO-893" "GSWO-892" "GSWO-891"
cKO
"GSWO-890"

$SFLA591167
$SFLA591167$`TUI5Y-1335`
aKO cKO bKO deltaKO alphaKO gammaKO
"GI5Y-1972" "GI5Y-1971" "GI5Y-1970" "GI5Y-1969" "GI5Y-1968" "GI5Y-1967"
betaKO epsilonKO
"GI5Y-1966" "GI5Y-1965"

$`SFRE1185652-WGS`
$`SFRE1185652-WGS`$`TUSVQ-3440`
deltaKO alphaKO gammaKO betaKO epsilonKO
"GSVQ-5563" "GSVQ-5562" "GSVQ-5561" "GSVQ-5560" "GSVQ-5559"

$`SFRE1185652-WGS`$`TUSVQ-654`
bKO2 bKO1 cKO aKO
"GSVQ-549" "GSVQ-548" "GSVQ-547" "GSVQ-546"

$SFLE591020
$SFLE591020$`TULK5-2321`
aKO cKO bKO deltaKO alphaKO gammaKO
"GLK5-4060" "GLK5-4059" "GLK5-4058" "GLK5-4057" "GLK5-4056" "GLK5-4055"
betaKO epsilonKO
"GLK5-4054" "GLK5-4053"

$SFRE1117943
$SFRE1117943$`TUJT5-3073`
deltaKO alphaKO gammaKO betaKO epsilonKO
"GJT5-3190" "GJT5-3189" "GJT5-3188" "GJT5-3187" "GJT5-3185"

$SFRE1117943$`TUJT5-1532`
bKO2 bKO1 cKO aKO
"GJT5-521" "GJT5-520" "GJT5-519" "GJT5-518"

$`SFUL1303692-WGS`
$`SFUL1303692-WGS`$`TUSXZ-3019`
epsilonKO betaKO gammaKO alphaKO
"GSXZ-5162" "GSXZ-5161" "GSXZ-5160" "GSXZ-5159"

$`SFUL1303692-WGS`$`TUSXZ-3018`
deltaKO bKO cKO aKO

"GSXZ-5158" "GSXZ-5157" "GSXZ-5156" "GSXZ-5155"

$SFLE198214
$SFLE198214$`TUD39-226080`
aKO cKO bKO deltaKO alphaKO
"GD39-221859" "GD39-221858" "GD39-221857" "GD39-221856" "GD39-221855"
gammaKO betaKO epsilonKO
"GD39-221854" "GD39-221853" "GD39-221852"

$SFRI318167
$SFRI318167$`TUIXS-1835`
gammaKO1 alphaKO1 bKO1 cKO1 aKO1
"GIXS-3171" "GIXS-3170" "GIXS-3169" "GIXS-3168" "GIXS-3167"

$SFRI318167$`TUIXS-2427`
aKO2 cKO2 bKO2 deltaKO alphaKO2 gammaKO2
"GIXS-4184" "GIXS-4183" "GIXS-4182" "GIXS-4181" "GIXS-4180" "GIXS-4179"
betaKO2 epsilonKO2
"GIXS-4178" "GIXS-4177"

$SFRI318167$`TUIXS-1833`
epsilonKO1 betaKO1
"GIXS-3164" "GIXS-3163"

$SFUM335543
$SFUM335543$`TUH6P-1658`
bKO2 bKO1 deltaKO alphaKO gammaKO betaKO
"GH6P-2635" "GH6P-2634" "GH6P-2633" "GH6P-2632" "GH6P-2631" "GH6P-2630"
epsilonKO
"GH6P-2629"

$SFUM335543$`TUH6P-1041`
cKO
"GH6P-1635"

$SFUM335543$`TUH6P-1042`
aKO
"GH6P-1636"

$SAUR985002
$SAUR985002$`TULKP-1067`
aKO cKO bKO deltaKO alphaKO gammaKO
"GLKP-2006" "GLKP-2005" "GLKP-2004" "GLKP-2003" "GLKP-2002" "GLKP-2001"
betaKO epsilonKO
"GLKP-2000" "GLKP-1999"

$LMON653938
$LMON653938$`TUJ8G-1441`
aKO cKO1 bKO deltaKO1 alphaKO1 gammaKO1

```
"GJ8G-2785" "GJ8G-2784" "GJ8G-2783" "GJ8G-2782" "GJ8G-2781" "GJ8G-2780"
   betaKO1  epsilonKO1
"GJ8G-2779" "GJ8G-2778"

$LMON653938$`TUJ8G-1558`
     cKO2    deltaKO2    alphaKO2    gammaKO2     betaKO2  epsilonKO2
"GJ8G-2990" "GJ8G-2989" "GJ8G-2988" "GJ8G-2987" "GJ8G-2986" "GJ8G-2985"


$SFLE373384
$SFLE373384$`TUHZM-2049`
      aKO         cKO         bKO     deltaKO     alphaKO     gammaKO
"GHZM-3760" "GHZM-3759" "GHZM-3758" "GHZM-3757" "GHZM-3756" "GHZM-3755"
    betaKO   epsilonKO
"GHZM-3754" "GHZM-3753"


$SHIGELLA
$SHIGELLA$`TU7V-7921`
alphaKO deltaKO     bKO
 "ATPA"  "ATPH"  "ATPF"

$SHIGELLA$`TU7V-7922`
epsilonKO    betaKO   gammaKO
  "ATPC"    "ATPD"    "ATPG"

$SHIGELLA$`TU7V-7920`
  cKO
"ATPE"

$SHIGELLA$`TU7V-7919`
  aKO
"ATPB"


$SAGA1203670
$SAGA1203670$`TULKY-452`
  gammaKO    alphaKO    deltaKO        bKO        aKO        cKO
"GLKY-932" "GLKY-931" "GLKY-930" "GLKY-929" "GLKY-928" "GLKY-927"

$SAGA1203670$`TULKY-453`
 epsilonKO     betaKO
"GLKY-934" "GLKY-933"


$SGAL990317
$SGAL990317$`TUI5F-439`
 epsilonKO     betaKO    gammaKO    alphaKO    deltaKO        bKO        aKO
"GI5F-855" "GI5F-854" "GI5F-853" "GI5F-852" "GI5F-851" "GI5F-850" "GI5F-849"
     cKO
"GI5F-848"


$SGLO343509
```

```
$SGLO343509$`TUJJC-1560`
  epsilonKO      betaKO     gammaKO     alphaKO     deltaKO         bKO 
"GJJC-2506" "GJJC-2505" "GJJC-2504" "GJJC-2503" "GJJC-2502" "GJJC-2501" 
        cKO         aKO 
"GJJC-2500" "GJJC-2499" 


$SGRA984262
$SGRA984262$`TULJW-1386`
    gammaKO     alphaKO     deltaKO         bKO         cKO         aKO 
"GLJW-2370" "GLJW-2369" "GLJW-2368" "GLJW-2367" "GLJW-2366" "GLJW-2365" 

$SGRA984262$`TULJW-1173`
     betaKO   epsilonKO 
"GLJW-2012" "GLJW-2011" 


$SGOR467705
$SGOR467705$`TUH3R-813`
        cKO         aKO         bKO     deltaKO     alphaKO     gammaKO 
"GH3R-1547" "GH3R-1546" "GH3R-1545" "GH3R-1544" "GH3R-1543" "GH3R-1542" 
     betaKO   epsilonKO 
"GH3R-1541" "GH3R-1540" 


$SGRI455632
$SGRI455632$`TUD3A-1255`
        aKO         cKO         bKO     deltaKO     alphaKO     gammaKO 
"GD3A-2182" "GD3A-2181" "GD3A-2180" "GD3A-2179" "GD3A-2178" "GD3A-2177" 
     betaKO   epsilonKO 
"GD3A-2176" "GD3A-2175" 


$SGAL981539
$SGAL981539$`TULL2-419`
 epsilonKO     betaKO    gammaKO    alphaKO    deltaKO        bKO        aKO 
"GLL2-842" "GLL2-841" "GLL2-840" "GLL2-839" "GLL2-838" "GLL2-837" "GLL2-836" 
       cKO 
"GLL2-835" 


$APHO522306
$APHO522306$`TUHXL-1530`
   alphaKO1        bKO1        cKO1        aKO1  epsilonKO1     betaKO1 
"GHXL-2708" "GHXL-2707" "GHXL-2706" "GHXL-2705" "GHXL-2703" "GHXL-2702" 

$APHO522306$`TUHXL-2380`
       aKO2        cKO2        bKO2     deltaKO    alphaKO2    gammaKO2 
"GHXL-4401" "GHXL-4400" "GHXL-4399" "GHXL-4398" "GHXL-4397" "GHXL-4396" 
    betaKO2  epsilonKO2 
"GHXL-4395" "GHXL-4394" 

$APHO522306$`TUHXL-1531`
   gammaKO1 
```

```
"GHXL-2715"


$SGLY645991
$SGLY645991$`TUHJ4-1672`
       aKO         cKO         bKO     deltaKO     alphaKO     gammaKO 
"GHJ4-3342" "GHJ4-3341" "GHJ4-3340" "GHJ4-3339" "GHJ4-3338" "GHJ4-3337" 
    betaKO   epsilonKO 
"GHJ4-3336" "GHJ4-3335" 


$LMON169963
$LMON169963$`TU9FH-71264`
epsilonKO1    betaKO1   gammaKO1   alphaKO1   deltaKO1       cKO1 
 "LMO0093"  "LMO0092"  "LMO0091"  "LMO0090"  "LMO0089"  "LMO0088" 

$LMON169963$`TU9FH-72557`
      bKO  deltaKO2  alphaKO2  gammaKO2 
"LMO2533" "LMO2532" "LMO2531" "LMO2530" 

$LMON169963$`TU9FH-72556`
   betaKO2 epsilonKO2 
 "LMO2529"  "LMO2528" 

$LMON169963$`TU9FH-72558`
     cKO2 
"LMO2534" 

$LMON169963$`TU9FH-72559`
      aKO 
"LMO2535" 


$SHAE279808
$SHAE279808$`TUJX7-547`
 epsilonKO     betaKO    gammaKO    alphaKO    deltaKO        bKO        cKO 
"GJX7-944" "GJX7-943" "GJX7-942" "GJX7-941" "GJX7-940" "GJX7-939" "GJX7-938" 
       aKO 
"GJX7-937" 


$SENT1160717
$SENT1160717$`TULJS-27`
       aKO         cKO         bKO     deltaKO     alphaKO     gammaKO 
"GLJS-4579" "GLJS-4578" "GLJS-4577" "GLJS-4576" "GLJS-4575" "GLJS-4574" 
    betaKO   epsilonKO 
"GLJS-4573" "GLJS-4572" 


$SSP60480
$SSP60480$`TUI2N-2390`
       aKO         cKO         bKO     deltaKO     alphaKO     gammaKO 
"GI2N-4072" "GI2N-4071" "GI2N-4070" "GI2N-4069" "GI2N-4068" "GI2N-4067" 
    betaKO   epsilonKO 
```

```
"GI2N-4066" "GI2N-4065"


$SSP743722
$SSP743722$`TUH04-3072`
      aKO       cKO       bKO    deltaKO    alphaKO    gammaKO
"GH04-5297" "GH04-5296" "GH04-5295" "GH04-5294" "GH04-5293" "GH04-5292"

$SSP743722$`TUH04-1358`
    betaKO   epsilonKO
"GH04-2316" "GH04-2315"


$SHEL471855
$SHEL471855$`TUH2I-1175`
      cKO       bKO    deltaKO    alphaKO    gammaKO     betaKO
"GH2I-2146" "GH2I-2145" "GH2I-2144" "GH2I-2143" "GH2I-2142" "GH2I-2141"
  epsilonKO
"GH2I-2140"

$SHEL471855$`TUH2I-1176`
      aKO
"GH2I-2147"


$SHAL458817
$SHAL458817$`TUH1X-2641`
      aKO       cKO       bKO    deltaKO    alphaKO    gammaKO
"GH1X-4450" "GH1X-4449" "GH1X-4448" "GH1X-4447" "GH1X-4446" "GH1X-4445"
    betaKO   epsilonKO
"GH1X-4444" "GH1X-4443"


$SSP60481
$SSP60481$`TUHW6-2431`
      aKO       cKO       bKO    deltaKO    alphaKO    gammaKO
"GHW6-4166" "GHW6-4165" "GHW6-4164" "GHW6-4163" "GHW6-4162" "GHW6-4161"
    betaKO   epsilonKO
"GHW6-4160" "GHW6-4159"


$SSP94122
$SSP94122$`TUJ9K-155`
      aKO       cKO       bKO    deltaKO    alphaKO    gammaKO
"GJ9K-4266" "GJ9K-4265" "GJ9K-4264" "GJ9K-4263" "GJ9K-4262" "GJ9K-4261"
    betaKO   epsilonKO
"GJ9K-4260" "GJ9K-4259"


$SSUI945704
$SSUI945704$`TULLM-577`
      cKO       aKO       bKO    deltaKO    alphaKO    gammaKO
"GLLM-1188" "GLLM-1187" "GLLM-1186" "GLLM-1185" "GLLM-1184" "GLLM-1183"
    betaKO   epsilonKO
```

```
"GLLM-1182" "GLLM-1181"


$`SHYG1203460-WGS`
$`SHYG1203460-WGS`$`TUSXK-3840`
     betaKO     gammaKO     alphaKO     deltaKO         bKO         cKO
"GSXK-6283" "GSXK-6282" "GSXK-6281" "GSXK-6280" "GSXK-6279" "GSXK-6278"
        aKO
"GSXK-6277"

$`SHYG1203460-WGS`$`TUSXK-3841`
  epsilonKO
"GSXK-6284"


$SPUT399804
$SPUT399804$`TULK4-2516`
        aKO         cKO         bKO     deltaKO     alphaKO     gammaKO
"GLK4-4408" "GLK4-4407" "GLK4-4406" "GLK4-4405" "GLK4-4404" "GLK4-4403"
     betaKO   epsilonKO
"GLK4-4402" "GLK4-4401"


$LMON882095
$LMON882095$`TULFS-55`
epsilonKO1    betaKO1   gammaKO1   alphaKO1   deltaKO1       cKO1
"GLFS-108" "GLFS-107" "GLFS-106" "GLFS-105" "GLFS-104" "GLFS-103"

$LMON882095$`TULFS-1336`
        aKO        cKO2         bKO    deltaKO2    alphaKO2    gammaKO2
"GLFS-2622" "GLFS-2621" "GLFS-2620" "GLFS-2619" "GLFS-2618" "GLFS-2617"
    betaKO2  epsilonKO2
"GLFS-2616" "GLFS-2615"


$SSP351745
$SSP351745$`TUCOY-2381`
        aKO         cKO         bKO     deltaKO     alphaKO     gammaKO
"GCOY-4181" "GCOY-4180" "GCOY-4179" "GCOY-4178" "GCOY-4177" "GCOY-4176"
     betaKO   epsilonKO
"GCOY-4175" "GCOY-4174"


$SHYG1133850
$SHYG1133850$`TULLU-4173`
     betaKO     gammaKO     alphaKO     deltaKO         bKO         cKO
"GLLU-6511" "GLLU-6510" "GLLU-6509" "GLLU-6508" "GLLU-6507" "GLLU-6506"
        aKO
"GLLU-6505"

$SHYG1133850$`TULLU-4174`
  epsilonKO
"GLLU-6512"
```

```
$`SINT862967-WGS`
$`SINT862967-WGS`$`TUSWS-226`
 epsilonKO     betaKO    gammaKO    alphaKO    deltaKO        bKO        aKO
"GSWS-491" "GSWS-490" "GSWS-489" "GSWS-488" "GSWS-487" "GSWS-486" "GSWS-485"
       cKO
"GSWS-484"



$SINT591365
$SINT591365$`TULL4-605`
        aKO         bKO     deltaKO     alphaKO     gammaKO      betaKO
"GLL4-1150" "GLL4-1149" "GLL4-1148" "GLL4-1147" "GLL4-1146" "GLL4-1145"
  epsilonKO
"GLL4-1144"

$SINT591365$noTU
cKO
 NA



$SINF1069533
$SINF1069533$`TULL3-387`
 epsilonKO     betaKO    gammaKO    alphaKO    deltaKO        bKO        aKO
"GLL3-691" "GLL3-690" "GLL3-689" "GLL3-688" "GLL3-687" "GLL3-686" "GLL3-684"
       cKO
"GLL3-683"



$`SINI1318633-WGS`
$`SINI1318633-WGS`$`TUSWR-632`
        cKO         aKO         bKO     deltaKO     alphaKO     gammaKO
"GSWR-1259" "GSWR-1258" "GSWR-1257" "GSWR-1256" "GSWR-1255" "GSWR-1254"
     betaKO   epsilonKO
"GSWR-1253" "GSWR-1252"



$`RPOM246200-WGS`
$`RPOM246200-WGS`$`TUSU0-1752`
    deltaKO     alphaKO     gammaKO      betaKO   epsilonKO
"GSU0-3188" "GSU0-3187" "GSU0-3186" "GSU0-3185" "GSU0-3184"

$`RPOM246200-WGS`$`TUSU0-1780`
        aKO         cKO        bKO2        bKO1
"GSU0-3259" "GSU0-3258" "GSU0-3257" "GSU0-3256"



$SAUR869816
$SAUR869816$`TULKN-1088`
        bKO     deltaKO     alphaKO     gammaKO      betaKO   epsilonKO
"GLKN-2065" "GLKN-2064" "GLKN-2063" "GLKN-2062" "GLKN-2061" "GLKN-2060"

$SAUR869816$`TULKN-1089`
        aKO         cKO
```

```
"GLKN-2067" "GLKN-2066"


$RSP292414
$RSP292414$`TUHCT-1596`
   deltaKO     alphaKO     gammaKO      betaKO   epsilonKO
"GHCT-2126" "GHCT-2125" "GHCT-2124" "GHCT-2123" "GHCT-2122"

$RSP292414$`TUHCT-1876`
        aKO         cKO
"GHCT-2639" "GHCT-2638"

$RSP292414$`TUHCT-1875`
       bKO2        bKO1
"GHCT-2637" "GHCT-2636"


$`SINT862966-WGS`
$`SINT862966-WGS`$`TUSWT-214`
 epsilonKO     betaKO    gammaKO    alphaKO    deltaKO        bKO        aKO
"GSWT-475" "GSWT-474" "GSWT-473" "GSWT-472" "GSWT-471" "GSWT-470" "GSWT-469"
       cKO
"GSWT-468"


$BANI552531
$BANI552531$`TUL90-174`
       aKO        cKO        bKO    deltaKO    alphaKO    gammaKO     betaKO
"GL90-325" "GL90-324" "GL90-323" "GL90-322" "GL90-321" "GL90-320" "GL90-319"
 epsilonKO
"GL90-318"


$`LMON1234142-WGS`
$`LMON1234142-WGS`$`TUSPW-46`
epsilonKO1    betaKO1   gammaKO1   alphaKO1   deltaKO1       cKO1
 "GSPW-93"  "GSPW-92"  "GSPW-91"  "GSPW-90"  "GSPW-89"  "GSPW-88"

$`LMON1234142-WGS`$`TUSPW-1473`
        aKO        cKO2         bKO    deltaKO2    alphaKO2    gammaKO2
"GSPW-2865" "GSPW-2864" "GSPW-2862" "GSPW-2861" "GSPW-2860" "GSPW-2858"
    betaKO2  epsilonKO2
"GSPW-2855" "GSPW-2854"


$BANI1075106
$BANI1075106$`TUL93-891`
        aKO         cKO         bKO     deltaKO     alphaKO     gammaKO
"GL93-1435" "GL93-1434" "GL93-1433" "GL93-1432" "GL93-1431" "GL93-1430"
     betaKO   epsilonKO
"GL93-1429" "GL93-1428"


$BBAL866536
```

$BBAL866536$`TUL8V-373`
aKO cKO bKO deltaKO alphaKO gammaKO
"GL8V-657" "GL8V-656" "GL8V-655" "GL8V-654" "GL8V-653" "GL8V-652"

$BBAL866536$`TUL8V-294`
epsilonKO betaKO
"GL8V-533" "GL8V-532"

$BBRE358681
$BBRE358681$`TUHYS-2967`
aKO cKO bKO deltaKO alphaKO gammaKO
"GHYS-5627" "GHYS-5626" "GHYS-5625" "GHYS-5624" "GHYS-5623" "GHYS-5622"
betaKO epsilonKO
"GHYS-5621" "GHYS-5620"

$SPNE488222
$SPNE488222$`TUI12-712`
cKO aKO bKO deltaKO alphaKO gammaKO
"GI12-1389" "GI12-1388" "GI12-1387" "GI12-1386" "GI12-1385" "GI12-1384"
betaKO epsilonKO
"GI12-1383" "GI12-1382"

$SJAP452662
$SJAP452662$`TUHEL-1578`
alphaKO deltaKO
"GHEL-2731" "GHEL-2730"

$SJAP452662$`TUHEL-1579`
betaKO gammaKO
"GHEL-2734" "GHEL-2733"

$SJAP452662$`TUHEL-1580`
epsilonKO
"GHEL-2735"

$SJAP452662$`TUHEL-1355`
aKO cKO bKO2 bKO1
"GHEL-2306" "GHEL-2305" "GHEL-2304" "GHEL-2303"

$SKED446469
$SKED446469$`TUHD3-557`
betaKO gammaKO alphaKO deltaKO bKO cKO
"GHD3-1000" "GHD3-999" "GHD3-998" "GHD3-997" "GHD3-996" "GHD3-995"
aKO
"GHD3-994"

$SKED446469$`TUHD3-558`
epsilonKO
"GHD3-1001"

```
$SAUR546342
$SAUR546342$`TULKM-1158`
       bKO     deltaKO     alphaKO     gammaKO      betaKO   epsilonKO
"GLKM-2187" "GLKM-2186" "GLKM-2185" "GLKM-2184" "GLKM-2183" "GLKM-2182"

$SAUR546342$`TULKM-1159`
       aKO         cKO
"GLKM-2189" "GLKM-2188"


$SKUJ709032
$SKUJ709032$`TUHTQ-419`
 epsilonKO     betaKO    gammaKO    alphaKO    deltaKO       bKO2       bKO1
"GHTQ-780" "GHTQ-779" "GHTQ-778" "GHTQ-777" "GHTQ-776" "GHTQ-775" "GHTQ-774"

$SKUJ709032$`TUHTQ-248`
       cKO
"GHTQ-348"

$SKUJ709032$`TUHTQ-446`
       aKO
"GHTQ-845"


$SLUG698737
$SLUG698737$`TUHG4-436`
 epsilonKO     betaKO    gammaKO    alphaKO    deltaKO        bKO        cKO
"GHG4-920" "GHG4-919" "GHG4-918" "GHG4-917" "GHG4-916" "GHG4-915" "GHG4-914"
       aKO
"GHG4-913"


$SLIN504472
$SLIN504472$`TUHKB-4551`
    gammaKO     alphaKO
"GHKB-6583" "GHKB-6582"

$SLIN504472$`TUHKB-3612`
     betaKO
"GHKB-5163"

$SLIN504472$`TUHKB-2362`
        aKO         cKO         bKO     deltaKO
"GHKB-3241" "GHKB-3240" "GHKB-3239" "GHKB-3238"

$SLIN504472$`TUHKB-3613`
  epsilonKO
"GHKB-5164"


$SLUG1034809
$SLUG1034809$`TULKV-462`
 epsilonKO     betaKO    gammaKO    alphaKO    deltaKO        bKO        cKO
```

"GLKV-976" "GLKV-975" "GLKV-974" "GLKV-973" "GLKV-972" "GLKV-971" "GLKV-970"
aKO
"GLKV-969"

$`LMON882096-WGS`
$`LMON882096-WGS`$`TUSQB-43`
epsilonKO1 betaKO1 gammaKO1 alphaKO1 deltaKO1 cKO1
"GSQB-90" "GSQB-89" "GSQB-88" "GSQB-87" "GSQB-86" "GSQB-85"

$`LMON882096-WGS`$`TUSQB-1310`
aKO cKO2 bKO deltaKO2 alphaKO2 gammaKO2
"GSQB-2615" "GSQB-2614" "GSQB-2613" "GSQB-2612" "GSQB-2611" "GSQB-2610"
betaKO2 epsilonKO2
"GSQB-2609" "GSQB-2608"

$SLOI323850
$SLOI323850$`TUHQJ-2290`
aKO cKO bKO deltaKO alphaKO gammaKO
"GHQJ-3975" "GHQJ-3974" "GHQJ-3973" "GHQJ-3972" "GHQJ-3971" "GHQJ-3970"
betaKO epsilonKO
"GHQJ-3969" "GHQJ-3968"

$SLIP643648
$SLIP643648$`TUHUR-1216`
aKO cKO bKO deltaKO alphaKO gammaKO
"GHUR-2341" "GHUR-2340" "GHUR-2339" "GHUR-2338" "GHUR-2337" "GHUR-2336"
betaKO epsilonKO
"GHUR-2335" "GHUR-2334"

$`SLIQ1346614-WGS`
$`SLIQ1346614-WGS`$`TUSUX-2677`
epsilonKO betaKO gammaKO alphaKO deltaKO bKO
"GSUX-4928" "GSUX-4927" "GSUX-4926" "GSUX-4925" "GSUX-4924" "GSUX-4923"
cKO aKO
"GSUX-4922" "GSUX-4921"

$SLIT580332
$SLIT580332$`TUH9F-1361`
aKO cKO bKO deltaKO alphaKO gammaKO
"GH9F-3041" "GH9F-3040" "GH9F-3039" "GH9F-3038" "GH9F-3037" "GH9F-3036"
betaKO epsilonKO
"GH9F-3035" "GH9F-3034"

$`SLUT1076934-WGS`
$`SLUT1076934-WGS`$`TUSWW-348`
epsilonKO betaKO gammaKO alphaKO deltaKO bKO aKO
"GSWW-678" "GSWW-677" "GSWW-676" "GSWW-675" "GSWW-674" "GSWW-673" "GSWW-672"
cKO

```
"GSWW-671"


$SSP436114
$SSP436114$`TUI6I-651`
      bKO2       bKO1     deltaKO     alphaKO     gammaKO      betaKO
"GI6I-1674" "GI6I-1673" "GI6I-1672" "GI6I-1671" "GI6I-1670" "GI6I-1669"

$SSP436114$`TUI6I-626`
  epsilonKO
"GI6I-1614"

$SSP436114$`TUI6I-395`
        cKO         aKO
"GI6I-1015" "GI6I-1013"


$SAVE227882
$SAVE227882$`TUJU1-1794`
        aKO         cKO         bKO     deltaKO     alphaKO     gammaKO
"GJU1-2908" "GJU1-2907" "GJU1-2906" "GJU1-2905" "GJU1-2904" "GJU1-2903"
     betaKO   epsilonKO
"GJU1-2902" "GJU1-2901"


$`SMAR1249634-WGS`
$`SMAR1249634-WGS`$`TUSUZ-2454`
  epsilonKO      betaKO     gammaKO     alphaKO     deltaKO         bKO
"GSUZ-4566" "GSUZ-4565" "GSUZ-4564" "GSUZ-4563" "GSUZ-4562" "GSUZ-4561"
        cKO         aKO
"GSUZ-4560" "GSUZ-4559"


$SMIT365659
$SMIT365659$`TUC91-3814`
        cKO         aKO         bKO     deltaKO     alphaKO     gammaKO
"GC91-1606" "GC91-1603" "GC91-1607" "GC91-1609" "GC91-1602" "GC91-1608"
     betaKO   epsilonKO
"GC91-1605" "GC91-1604"


$SMED366394
$SMED366394$`TUJAL-3251`
    deltaKO     alphaKO     gammaKO      betaKO   epsilonKO
"GJAL-2975" "GJAL-2974" "GJAL-2973" "GJAL-2972" "GJAL-2971"

$SMED366394$`TUJAL-1848`
      bKO2       bKO1        cKO        aKO
"GJAL-457" "GJAL-456" "GJAL-455" "GJAL-454"


$SMEL266834
$SMEL266834$`TUJF6-3368`
    deltaKO     alphaKO     gammaKO      betaKO   epsilonKO
```

"GJF6-3114" "GJF6-3113" "GJF6-3112" "GJF6-3111" "GJF6-3110"

$SMEL266834$`TUJF6-2075`
bKO1 bKO2 cKO aKO
"GJF6-857" "GJF6-856" "GJF6-855" "GJF6-854"

$`LMON1334565-WGS`
$`LMON1334565-WGS`$`TUSPP-43`
epsilonKO1 betaKO1 gammaKO1 alphaKO1 deltaKO1 cKO1
"GSPP-90" "GSPP-89" "GSPP-88" "GSPP-87" "GSPP-86" "GSPP-85"

$`LMON1334565-WGS`$`TUSPP-1309`
aKO cKO2 bKO deltaKO2 alphaKO2 gammaKO2
"GSPP-2602" "GSPP-2601" "GSPP-2600" "GSPP-2599" "GSPP-2598" "GSPP-2597"
betaKO2 epsilonKO2
"GSPP-2596" "GSPP-2595"

$`SMEL1235461-WGS`
$`SMEL1235461-WGS`$`TUSW9-3668`
deltaKO alphaKO gammaKO betaKO epsilonKO
"GSW9-3149" "GSW9-3148" "GSW9-3147" "GSW9-3146" "GSW9-3145"

$`SMEL1235461-WGS`$`TUSW9-2347`
bKO2 bKO1 cKO aKO
"GSW9-828" "GSW9-827" "GSW9-826" "GSW9-825"

$`SMEL1286640-WGS`
$`SMEL1286640-WGS`$`TUSVA-4840`
alphaKO
"GSVA-3997"

$`SMEL1286640-WGS`$`TUSVA-3121`
gammaKO betaKO epsilonKO
"GSVA-3995" "GSVA-3994" "GSVA-3993"

$`SMEL1286640-WGS`$`TUSVA-3122`
deltaKO
"GSVA-3999"

$`SMEL1286640-WGS`$`TUSVA-1589`
bKO1 bKO2 cKO aKO
"GSVA-1117" "GSVA-1116" "GSVA-1115" "GSVA-1114"

$SMON519441
$SMON519441$`TUHY8-410`
aKO cKO bKO deltaKO alphaKO gammaKO
"GHY8-1065" "GHY8-1064" "GHY8-1063" "GHY8-1062" "GHY8-1061" "GHY8-1060"
betaKO epsilonKO
"GHY8-1059" "GHY8-1058"

```
$CSUL444179
$CSUL444179$`TUHLI-10`
      aKO       cKO       bKO   deltaKO   alphaKO   gammaKO
"GHLI-35" "GHLI-34" "GHLI-33" "GHLI-32" "GHLI-31" "GHLI-30"

$CSUL444179$`TUHLI-5`
   betaKO epsilonKO
"GHLI-17" "GHLI-16"


$`USUL1249480-WGS`
$`USUL1249480-WGS`$`TUSZ8-750`
       bKO2        bKO1     deltaKO     alphaKO     gammaKO      betaKO
"GSZ8-1695" "GSZ8-1694" "GSZ8-1693" "GSZ8-1692" "GSZ8-1691" "GSZ8-1690"
  epsilonKO
"GSZ8-1689"

$`USUL1249480-WGS`$`TUSZ8-136`
       cKO
"GSZ8-315"

$`USUL1249480-WGS`$`TUSZ8-724`
        aKO
"GSZ8-1629"


$CSUL641892
$CSUL641892$`TUH69-10`
      aKO       cKO       bKO   deltaKO   alphaKO   gammaKO
"GH69-35" "GH69-34" "GH69-33" "GH69-32" "GH69-31" "GH69-30"

$CSUL641892$`TUH69-6`
   betaKO epsilonKO
"GH69-17" "GH69-16"


$`SMEL1230587-WGS`
$`SMEL1230587-WGS`$`TUSVC-2485`
    deltaKO     alphaKO     gammaKO      betaKO   epsilonKO
"GSVC-2945" "GSVC-2944" "GSVC-2943" "GSVC-2942" "GSVC-2941"

$`SMEL1230587-WGS`$`TUSVC-1130`
      bKO2       bKO1        cKO        aKO
"GSVC-499" "GSVC-498" "GSVC-497" "GSVC-496"


$SMEL693982
$SMEL693982$`TUJDT-1907`
    deltaKO     alphaKO     gammaKO      betaKO   epsilonKO
"GJDT-3143" "GJDT-3142" "GJDT-3141" "GJDT-3140" "GJDT-3139"

$SMEL693982$`TUJDT-505`
      bKO2       bKO1        cKO        aKO
```

"GJDT-552" "GJDT-551" "GJDT-550" "GJDT-549"

$CSUL595499
$CSUL595499$`TUHS7-15`
aKO cKO bKO deltaKO alphaKO gammaKO
"GHS7-41" "GHS7-40" "GHS7-39" "GHS7-38" "GHS7-37" "GHS7-36"

$CSUL595499$`TUHS7-6`
betaKO epsilonKO
"GHS7-16" "GHS7-15"

$SMAL391008
$SMAL391008$`TUH1H-1922`
aKO cKO bKO deltaKO alphaKO gammaKO
"GH1H-3592" "GH1H-3591" "GH1H-3590" "GH1H-3589" "GH1H-3588" "GH1H-3587"
betaKO epsilonKO
"GH1H-3586" "GH1H-3585"

$SMUT210007
$SMUT210007$`TUC7Z-777`
cKO aKO bKO deltaKO alphaKO gammaKO
"GC7Z-1439" "GC7Z-1438" "GC7Z-1437" "GC7Z-1436" "GC7Z-1435" "GC7Z-1434"
betaKO epsilonKO
"GC7Z-1433" "GC7Z-1432"

$`LMON1230340-WGS`
$`LMON1230340-WGS`$`TUSPX-57`
epsilonKO1 betaKO1 gammaKO1 alphaKO1 deltaKO1 cKO1
"GSPX-114" "GSPX-113" "GSPX-112" "GSPX-111" "GSPX-110" "GSPX-109"

$`LMON1230340-WGS`$`TUSPX-1365`
aKO cKO2 bKO deltaKO2 alphaKO2 gammaKO2
"GSPX-2698" "GSPX-2697" "GSPX-2696" "GSPX-2695" "GSPX-2694" "GSPX-2693"
betaKO2 epsilonKO2
"GSPX-2692" "GSPX-2691"

$SMUT1198676
$SMUT1198676$`TULL5-756`
cKO aKO bKO deltaKO alphaKO gammaKO
"GLL5-1382" "GLL5-1381" "GLL5-1380" "GLL5-1379" "GLL5-1378" "GLL5-1377"
betaKO epsilonKO
"GLL5-1376" "GLL5-1375"

$`CSUL1343076-WGS`
$`CSUL1343076-WGS`$`TUSJ3-8`
aKO cKO bKO deltaKO alphaKO gammaKO
"GSJ3-26" "GSJ3-25" "GSJ3-24" "GSJ3-23" "GSJ3-22" "GSJ3-21"

$`CSUL1343076-WGS`$`TUSJ3-4`
betaKO epsilonKO
"GSJ3-10" "GSJ3-9"

$`SMAR435998-WGS`
$`SMAR435998-WGS`$`TUSVH-3`
epsilonKO betaKO gammaKO alphaKO deltaKO bKO cKO aKO
"GSVH-9" "GSVH-8" "GSVH-7" "GSVH-6" "GSVH-5" "GSVH-4" "GSVH-3" "GSVH-2"

$SCAR396513
$SCAR396513$`TUJ9G-856`
aKO cKO bKO deltaKO alphaKO gammaKO
"GJ9G-1665" "GJ9G-1664" "GJ9G-1663" "GJ9G-1662" "GJ9G-1660" "GJ9G-1659"
betaKO epsilonKO
"GJ9G-1658" "GJ9G-1657"

$CSUL706194
$CSUL706194$`TUH8S-13`
aKO cKO bKO deltaKO alphaKO gammaKO
"GH8S-43" "GH8S-42" "GH8S-41" "GH8S-40" "GH8S-39" "GH8S-38"

$CSUL706194$`TUH8S-4`
betaKO epsilonKO
"GH8S-18" "GH8S-17"

$SMEL707241
$SMEL707241$`TULKB-3598`
deltaKO alphaKO gammaKO betaKO
"GLKB-3215" "GLKB-3214" "GLKB-3213" "GLKB-3212"

$SMEL707241$`TULKB-3597`
epsilonKO
"GLKB-3211"

$SMEL707241$`TULKB-2134`
bKO2 bKO1 cKO aKO
"GLKB-491" "GLKB-490" "GLKB-489" "GLKB-488"

$SMAL1163399
$SMAL1163399$`TULKX-1995`
aKO cKO bKO deltaKO alphaKO gammaKO
"GLKX-3704" "GLKX-3703" "GLKX-3702" "GLKX-3701" "GLKX-3700" "GLKX-3699"
betaKO epsilonKO
"GLKX-3698" "GLKX-3697"

$SNAS446470
$SNAS446470$`TUHHC-702`
betaKO gammaKO alphaKO deltaKO bKO cKO

"GHHC-1330" "GHHC-1329" "GHHC-1328" "GHHC-1327" "GHHC-1326" "GHHC-1325"
aKO
"GHHC-1324"

$SNAS446470$`TUHHC-995`
epsilonKO
"GHHC-1855"

$SPNE189423
$SPNE189423$`TUHX7-830`
cKO aKO bKO deltaKO alphaKO gammaKO
"GHX7-1588" "GHX7-1587" "GHX7-1586" "GHX7-1585" "GHX7-1584" "GHX7-1583"
betaKO epsilonKO
"GHX7-1582" "GHX7-1581"

$SPNE525381
$SPNE525381$`TUH2H-906`
cKO aKO bKO deltaKO alphaKO gammaKO
"GH2H-1786" "GH2H-1785" "GH2H-1784" "GH2H-1783" "GH2H-1782" "GH2H-1781"
betaKO epsilonKO
"GH2H-1780" "GH2H-1779"

$BBIF484020
$BBIF484020$`TUL96-1049`
aKO cKO bKO deltaKO alphaKO gammaKO
"GL96-1680" "GL96-1679" "GL96-1678" "GL96-1677" "GL96-1676" "GL96-1675"
betaKO epsilonKO
"GL96-1674" "GL96-1673"

$BSP1186051
$BSP1186051$`TUL9C-26`
gammaKO alphaKO deltaKO bKO cKO aKO
"GL9C-77" "GL9C-76" "GL9C-75" "GL9C-74" "GL9C-73" "GL9C-72"

$BSP1186051$`TUL9C-189`
betaKO epsilonKO
"GL9C-552" "GL9C-551"

$LBUC523794
$LBUC523794$`TUHCR-383`
epsilonKO betaKO gammaKO alphaKO deltaKO bKO cKO
"GHCR-674" "GHCR-673" "GHCR-672" "GHCR-671" "GHCR-670" "GHCR-669" "GHCR-667"
aKO
"GHCR-666"

$`LMON930782-WGS`
$`LMON930782-WGS`$`TUSPQ-1209`
aKO cKO1 bKO deltaKO1 alphaKO1 gammaKO1

"GSPQ-2434" "GSPQ-2433" "GSPQ-2432" "GSPQ-2431" "GSPQ-2430" "GSPQ-2429"
betaKO1 epsilonKO1
"GSPQ-2428" "GSPQ-2427"

$`LMON930782-WGS`$`TUSPQ-1445`
epsilonKO2 betaKO2 gammaKO2 alphaKO2 deltaKO2 cKO2
"GSPQ-2955" "GSPQ-2954" "GSPQ-2953" "GSPQ-2952" "GSPQ-2951" "GSPQ-2950"

$BBRO568707
$BBRO568707$`TUL9B-1785`
epsilonKO betaKO gammaKO alphaKO deltaKO bKO
"GL9B-3859" "GL9B-3858" "GL9B-3857" "GL9B-3856" "GL9B-3855" "GL9B-3854"
cKO aKO
"GL9B-3853" "GL9B-3852"

$BCER405532
$BCER405532$`TUI1K-3152`
aKO cKO bKO deltaKO alphaKO
"GI1K-5384" "GI1K-5383" "GI1K-5382" "GI1K-5381" "GI1K-5380"

$BCER405532$`TUI1K-3151`
gammaKO betaKO epsilonKO
"GI1K-5378" "GI1K-5377" "GI1K-5376"

$BBIF883062
$BBIF883062$`TUH1R-1024`
aKO cKO bKO deltaKO alphaKO gammaKO
"GH1R-1646" "GH1R-1645" "GH1R-1644" "GH1R-1643" "GH1R-1642" "GH1R-1641"
betaKO epsilonKO
"GH1R-1640" "GH1R-1639"

$SPNE1130804
$SPNE1130804$`TULLC-791`
cKO aKO bKO deltaKO alphaKO gammaKO
"GLLC-1448" "GLLC-1447" "GLLC-1446" "GLLC-1445" "GLLC-1444" "GLLC-1443"
betaKO epsilonKO
"GLLC-1442" "GLLC-1441"

$SPNE561276
$SPNE561276$`TUJFJ-738`
cKO aKO bKO deltaKO alphaKO gammaKO
"GJFJ-1416" "GJFJ-1415" "GJFJ-1414" "GJFJ-1413" "GJFJ-1412" "GJFJ-1411"
betaKO epsilonKO
"GJFJ-1410" "GJFJ-1409"

$SPNE869269
$SPNE869269$`TULL8-707`
cKO aKO bKO deltaKO alphaKO gammaKO

"GLL8-1294" "GLL8-1293" "GLL8-1292" "GLL8-1291" "GLL8-1290" "GLL8-1289"
betaKO epsilonKO
"GLL8-1288" "GLL8-1287"

$SPNE488221
$SPNE488221$`TUH4U-791`
cKO aKO bKO deltaKO alphaKO gammaKO
"GH4U-1513" "GH4U-1512" "GH4U-1511" "GH4U-1510" "GH4U-1509" "GH4U-1508"
betaKO epsilonKO
"GH4U-1507" "GH4U-1506"

$SNOV639283
$SNOV639283$`TUCS4-1907`
deltaKO alphaKO gammaKO betaKO epsilonKO
"GCS4-3528" "GCS4-3527" "GCS4-3526" "GCS4-3525" "GCS4-3524"

$SNOV639283$`TUCS4-2386`
cKO aKO
"GCS4-4481" "GCS4-4480"

$SNOV639283$`TUCS4-2387`
bKO2 bKO1
"GCS4-4483" "GCS4-4482"

$SPNE574093
$SPNE574093$`TUHDB-809`
cKO aKO bKO deltaKO alphaKO gammaKO
"GHDB-1534" "GHDB-1533" "GHDB-1532" "GHDB-1531" "GHDB-1530" "GHDB-1529"

$SPNE574093$`TUHDB-808`
betaKO epsilonKO
"GHDB-1528" "GHDB-1527"

$SPNE487213
$SPNE487213$`TUI07-755`
cKO aKO bKO deltaKO alphaKO gammaKO
"GI07-1413" "GI07-1412" "GI07-1411" "GI07-1410" "GI07-1409" "GI07-1408"
betaKO epsilonKO
"GI07-1407" "GI07-1406"

$SPNE869309
$SPNE869309$`TULLB-393`
epsilonKO betaKO gammaKO alphaKO deltaKO bKO aKO
"GLLB-770" "GLLB-769" "GLLB-768" "GLLB-767" "GLLB-766" "GLLB-765" "GLLB-764"
cKO
"GLLB-763"

$`LMON882094-WGS`

```
$`LMON882094-WGS`$`TUSPT-48`
epsilonKO1    betaKO1   gammaKO1   alphaKO1   deltaKO1       cKO1
 "GSPT-98"  "GSPT-97"  "GSPT-96"  "GSPT-95"  "GSPT-94"  "GSPT-93"

$`LMON882094-WGS`$`TUSPT-1316`
        aKO        cKO2         bKO    deltaKO2    alphaKO2    gammaKO2
"GSPT-2572" "GSPT-2571" "GSPT-2570" "GSPT-2569" "GSPT-2568" "GSPT-2567"
    betaKO2  epsilonKO2
"GSPT-2566" "GSPT-2565"


$BCLA696125
$BCLA696125$`TUC2L-757`
    deltaKO     alphaKO     gammaKO      betaKO   epsilonKO
"GC2L-1326" "GC2L-1325" "GC2L-1324" "GC2L-1323" "GC2L-1322"

$BCLA696125$`TUC2L-632`
        cKO         aKO
"GC2L-1107" "GC2L-1106"

$BCLA696125$`TUC2L-633`
       bKO2        bKO1
"GC2L-1109" "GC2L-1108"


$SPNE869216
$SPNE869216$`TULL9-737`
        cKO         aKO         bKO     deltaKO     alphaKO     gammaKO
"GLL9-1360" "GLL9-1359" "GLL9-1358" "GLL9-1357" "GLL9-1356" "GLL9-1355"
     betaKO   epsilonKO
"GLL9-1354" "GLL9-1353"


$SPNE869215
$SPNE869215$`TULLA-700`
        cKO         aKO         bKO     deltaKO     alphaKO     gammaKO
"GLLA-1328" "GLLA-1327" "GLLA-1326" "GLLA-1325" "GLLA-1324" "GLLA-1323"
     betaKO   epsilonKO
"GLLA-1322" "GLLA-1321"


$`SOLI1302863-WGS`
$`SOLI1302863-WGS`$`TUSWX-309`
 epsilonKO     betaKO    gammaKO    alphaKO    deltaKO        bKO        aKO
"GSWX-609" "GSWX-608" "GSWX-607" "GSWX-606" "GSWX-605" "GSWX-604" "GSWX-603"
       cKO
"GSWX-602"


$SONE211586
$SONE211586$`TUK2N-4`
        aKO         cKO         bKO     deltaKO     alphaKO     gammaKO
"GK2N-9040" "GK2N-9039" "GK2N-9038" "GK2N-9037" "GK2N-9036" "GK2N-9035"
     betaKO   epsilonKO
```

```
"GK2N-9034" "GK2N-9033"


$SORA927666
$SORA927666$`TUH8G-546`
        cKO         aKO         bKO     deltaKO     alphaKO     gammaKO 
"GH8G-1127" "GH8G-1126" "GH8G-1125" "GH8G-1124" "GH8G-1123" "GH8G-1122" 
     betaKO   epsilonKO 
"GH8G-1121" "GH8G-1120" 


$SPYO471876
$SPYO471876$`TUC4Q-3205`
 epsilonKO     betaKO    gammaKO    alphaKO    deltaKO        bKO        aKO 
"GC4Q-789" "GC4Q-788" "GC4Q-787" "GC4Q-786" "GC4Q-785" "GC4Q-784" "GC4Q-783" 
       cKO 
"GC4Q-782" 


$SPYO286636
$SPYO286636$`TUHNO-323`
 epsilonKO     betaKO    gammaKO    alphaKO    deltaKO        bKO        aKO 
"GHNO-656" "GHNO-655" "GHNO-654" "GHNO-653" "GHNO-652" "GHNO-651" "GHNO-650" 
       cKO 
"GHNO-649" 


$SPYO319701
$SPYO319701$`TUHAD-306`
 epsilonKO     betaKO    gammaKO    alphaKO    deltaKO        bKO        aKO 
"GHAD-614" "GHAD-613" "GHAD-612" "GHAD-611" "GHAD-610" "GHAD-609" "GHAD-608" 
       cKO 
"GHAD-607" 


$SPUT319224
$SPUT319224$`TUHAP-2363`
        aKO         cKO         bKO     deltaKO     alphaKO     gammaKO 
"GHAP-4091" "GHAP-4090" "GHAP-4089" "GHAP-4088" "GHAP-4087" "GHAP-4086" 
     betaKO   epsilonKO 
"GHAP-4085" "GHAP-4084" 


$SPNE373153
$SPNE373153$`TUIX6-704`
        cKO         aKO         bKO     deltaKO     alphaKO     gammaKO 
"GIX6-1341" "GIX6-1340" "GIX6-1339" "GIX6-1338" "GIX6-1337" "GIX6-1336" 
     betaKO   epsilonKO 
"GIX6-1335" "GIX6-1334" 


$BCER226900
$BCER226900$`TUJEU-3155`
        aKO         cKO         bKO     deltaKO     alphaKO     gammaKO 
```

```
"GJEU-5304" "GJEU-5303" "GJEU-5302" "GJEU-5301" "GJEU-5300" "GJEU-5299"
    betaKO   epsilonKO
"GJEU-5298" "GJEU-5297"


$LMON882097
$LMON882097$`TULG0-43`
epsilonKO1    betaKO1   gammaKO1   alphaKO1   deltaKO1       cKO1
 "GLG0-83"  "GLG0-82"  "GLG0-81"  "GLG0-80"  "GLG0-79"  "GLG0-78"

$LMON882097$`TULG0-1276`
       aKO        cKO2        bKO    deltaKO2    alphaKO2    gammaKO2
"GLG0-2506" "GLG0-2505" "GLG0-2504" "GLG0-2503" "GLG0-2502" "GLG0-2501"
   betaKO2  epsilonKO2
"GLG0-2500" "GLG0-2499"


$SPRO399741
$SPRO399741$`TUI55-29`
epsilonKO    betaKO   gammaKO   alphaKO   deltaKO       bKO       cKO       aKO
 "GI55-9"  "GI55-8"  "GI55-7"  "GI55-6"  "GI55-5"  "GI55-4"  "GI55-3"  "GI55-2"


$SPYO160491
$SPYO160491$`TUJ8O-627`
       cKO         aKO         bKO     deltaKO     alphaKO     gammaKO
"GJ8O-1287" "GJ8O-1286" "GJ8O-1285" "GJ8O-1284" "GJ8O-1283" "GJ8O-1282"
    betaKO   epsilonKO
"GJ8O-1281" "GJ8O-1280"


$SPYO198466
$SPYO198466$`TUJDL-282`
 epsilonKO     betaKO    gammaKO    alphaKO    deltaKO        bKO        aKO
"GJDL-557" "GJDL-556" "GJDL-555" "GJDL-554" "GJDL-553" "GJDL-552" "GJDL-551"
       cKO
"GJDL-550"


$SPYO370552
$SPYO370552$`TUHYF-325`
 epsilonKO     betaKO    gammaKO    alphaKO    deltaKO        bKO        aKO
"GHYF-689" "GHYF-688" "GHYF-687" "GHYF-686" "GHYF-685" "GHYF-684" "GHYF-683"
       cKO
"GHYF-682"


$SPYO370554
$SPYO370554$`TUI3S-364`
 epsilonKO     betaKO    gammaKO    alphaKO    deltaKO        bKO        aKO
"GI3S-718" "GI3S-717" "GI3S-716" "GI3S-715" "GI3S-714" "GI3S-713" "GI3S-712"
       cKO
"GI3S-711"
```

$SPYO370553
$SPYO370553$`TUH2N-338`
epsilonKO betaKO gammaKO alphaKO deltaKO bKO aKO
"GH2N-697" "GH2N-696" "GH2N-695" "GH2N-694" "GH2N-693" "GH2N-692" "GH2N-691"
cKO
"GH2N-690"

$SPYO370551
$SPYO370551$`TUHLY-331`
epsilonKO betaKO gammaKO alphaKO deltaKO bKO aKO
"GHLY-693" "GHLY-692" "GHLY-691" "GHLY-690" "GHLY-689" "GHLY-688" "GHLY-687"
cKO
"GHLY-686"

$SPEA398579
$SPEA398579$`TUHG5-2617`
aKO cKO bKO deltaKO alphaKO gammaKO
"GHG5-4424" "GHG5-4423" "GHG5-4422" "GHG5-4421" "GHG5-4420" "GHG5-4419"
betaKO epsilonKO
"GHG5-4418" "GHG5-4417"

$SPYO186103
$SPYO186103$`TUHJG-328`
epsilonKO betaKO gammaKO alphaKO deltaKO bKO aKO
"GHJG-703" "GHJG-702" "GHJG-701" "GHJG-700" "GHJG-699" "GHJG-698" "GHJG-697"
cKO
"GHJG-696"

$BCER1217984
$BCER1217984$`TUL8H-1231`
epsilonKO betaKO gammaKO alphaKO deltaKO bKO
"GL8H-1878" "GL8H-1877" "GL8H-1876" "GL8H-1875" "GL8H-1874" "GL8H-1873"
cKO aKO
"GL8H-1872" "GL8H-1871"

$SPNE170187
$SPNE170187$`TUHGN-788`
cKO aKO bKO deltaKO alphaKO gammaKO
"GHGN-1520" "GHGN-1519" "GHGN-1518" "GHGN-1517" "GHGN-1516" "GHGN-1515"
betaKO epsilonKO
"GHGN-1514" "GHGN-1513"

$LMON879088
$LMON879088$`TULG1-54`
epsilonKO1 betaKO1 gammaKO1 alphaKO1 deltaKO1 cKO1
"GLG1-111" "GLG1-110" "GLG1-109" "GLG1-108" "GLG1-107" "GLG1-106"

$LMON879088$`TULG1-1335`
aKO cKO2 bKO deltaKO2 alphaKO2 gammaKO2
"GLG1-2615" "GLG1-2614" "GLG1-2613" "GLG1-2612" "GLG1-2611" "GLG1-2610"
betaKO2 epsilonKO2
"GLG1-2609" "GLG1-2608"

$`SPNE869303-WGS`
$`SPNE869303-WGS`$`TUSX2-221`
cKO aKO bKO deltaKO alphaKO gammaKO betaKO
"GSX2-420" "GSX2-419" "GSX2-418" "GSX2-417" "GSX2-416" "GSX2-415" "GSX2-414"
epsilonKO
"GSX2-413"

$`SPNE697283-WGS`
$`SPNE697283-WGS`$`TUSWY-756`
cKO aKO bKO deltaKO alphaKO gammaKO
"GSWY-1472" "GSWY-1471" "GSWY-1470" "GSWY-1469" "GSWY-1468" "GSWY-1467"
betaKO epsilonKO
"GSWY-1466" "GSWY-1465"

$`SPNE869306-WGS`
$`SPNE869306-WGS`$`TUSXM-708`
cKO aKO bKO deltaKO alphaKO gammaKO
"GSXM-1348" "GSXM-1347" "GSXM-1346" "GSXM-1345" "GSXM-1344" "GSXM-1343"
betaKO epsilonKO
"GSXM-1342" "GSXM-1341"

$`SPNE869307-WGS`
$`SPNE869307-WGS`$`TUSX6-709`
cKO aKO bKO deltaKO alphaKO gammaKO
"GSX6-1348" "GSX6-1347" "GSX6-1346" "GSX6-1345" "GSX6-1344" "GSX6-1343"
betaKO epsilonKO
"GSX6-1342" "GSX6-1341"

$`SPNE869304-WGS`
$`SPNE869304-WGS`$`TUSX3-718`
cKO aKO bKO deltaKO alphaKO gammaKO
"GSX3-1359" "GSX3-1358" "GSX3-1357" "GSX3-1356" "GSX3-1355" "GSX3-1354"
betaKO epsilonKO
"GSX3-1353" "GSX3-1352"

$SPNE488223
$SPNE488223$`TUHE7-777`
cKO aKO bKO deltaKO alphaKO gammaKO
"GHE7-1501" "GHE7-1500" "GHE7-1499" "GHE7-1498" "GHE7-1497" "GHE7-1496"
betaKO epsilonKO
"GHE7-1495" "GHE7-1494"

```
$`SENT1016998-WGS`
$`SENT1016998-WGS`$`TUSUK-2325`
       aKO         cKO         bKO     deltaKO     alphaKO     gammaKO
"GSUK-4802" "GSUK-4801" "GSUK-4800" "GSUK-4799" "GSUK-4798" "GSUK-4797"
     betaKO
"GSUK-4796"

$`SENT1016998-WGS`$`TUSUK-2324`
  epsilonKO
"GSUK-4795"



$SPNE171101
$SPNE171101$`TUJC8-723`
       cKO         aKO         bKO     deltaKO     alphaKO     gammaKO
"GJC8-1379" "GJC8-1378" "GJC8-1377" "GJC8-1376" "GJC8-1375" "GJC8-1374"
     betaKO   epsilonKO
"GJC8-1373" "GJC8-1372"



$LMON393127
$LMON393127$`TULFT-49`
epsilonKO1    betaKO1   gammaKO1   alphaKO1   deltaKO1       cKO1
"GLFT-102" "GLFT-101" "GLFT-100"  "GLFT-99"  "GLFT-98"  "GLFT-97"

$LMON393127$`TULFT-1292`
       aKO        cKO2         bKO    deltaKO2    alphaKO2    gammaKO2
"GLFT-2528" "GLFT-2527" "GLFT-2526" "GLFT-2525" "GLFT-2524" "GLFT-2523"
    betaKO2  epsilonKO2
"GLFT-2522" "GLFT-2521"



$SPYO193567
$SPYO193567$`TUHDO-712`
       cKO         aKO         bKO     deltaKO     alphaKO     gammaKO
"GHDO-1420" "GHDO-1419" "GHDO-1418" "GHDO-1417" "GHDO-1416" "GHDO-1415"
     betaKO   epsilonKO
"GHDO-1414" "GHDO-1413"



$SENT295319
$SENT295319$`TUJBZ-1946`
       aKO         cKO         bKO     deltaKO     alphaKO     gammaKO
"GJBZ-3708" "GJBZ-3707" "GJBZ-3706" "GJBZ-3705" "GJBZ-3704" "GJBZ-3703"
     betaKO   epsilonKO
"GJBZ-3702" "GJBZ-3701"



$LMON879090
$LMON879090$`TULG4-41`
epsilonKO1    betaKO1   gammaKO1   alphaKO1   deltaKO1       cKO1
 "GLG4-92"  "GLG4-91"  "GLG4-90"  "GLG4-89"  "GLG4-88"  "GLG4-87"
```

```
$LMON879090$`TULG4-1265`
        aKO         cKO2          bKO     deltaKO2     alphaKO2     gammaKO2
"GLG4-2524" "GLG4-2523" "GLG4-2522" "GLG4-2521" "GLG4-2520" "GLG4-2519"
    betaKO2  epsilonKO2
"GLG4-2518" "GLG4-2517"


$SPNE487214
$SPNE487214$`TUHY0-821`
        cKO         aKO         bKO      deltaKO      alphaKO      gammaKO
"GHY0-1596" "GHY0-1595" "GHY0-1594" "GHY0-1593" "GHY0-1592" "GHY0-1591"
     betaKO   epsilonKO
"GHY0-1590" "GHY0-1589"


$SPNE516950
$SPNE516950$`TUI38-810`
        cKO         aKO         bKO      deltaKO      alphaKO      gammaKO
"GI38-1510" "GI38-1509" "GI38-1508" "GI38-1507" "GI38-1506" "GI38-1505"
     betaKO   epsilonKO
"GI38-1504" "GI38-1503"


$BBAC360095
$BBAC360095$`TUHRY-66`
 epsilonKO     betaKO    gammaKO    alphaKO    deltaKO
"GHRY-115" "GHRY-114" "GHRY-113" "GHRY-112" "GHRY-111"

$BBAC360095$`TUHRY-219`
      bKO2       bKO1        cKO        aKO
"GHRY-379" "GHRY-378" "GHRY-377" "GHRY-376"


$BSP331104
$BSP331104$`TUH6K-185`
       aKO        cKO        bKO    deltaKO    alphaKO    gammaKO
"GH6K-567" "GH6K-566" "GH6K-565" "GH6K-564" "GH6K-563" "GH6K-562"

$BSP331104$`TUH6K-20`
epsilonKO    betaKO
"GH6K-74" "GH6K-73"


$`BBRO1208658-WGS`
$`BBRO1208658-WGS`$`TUSH1-2077`
        aKO         cKO         bKO      deltaKO      alphaKO      gammaKO
"GSH1-4347" "GSH1-4346" "GSH1-4345" "GSH1-4344" "GSH1-4343" "GSH1-4342"
     betaKO   epsilonKO
"GSH1-4341" "GSH1-4340"


$BBIF702459
$BBIF702459$`TUHBC-1009`
        cKO         bKO      deltaKO      alphaKO      gammaKO       betaKO
```

```
"GHBC-1601" "GHBC-1600" "GHBC-1599" "GHBC-1598" "GHBC-1597" "GHBC-1596"
  epsilonKO
"GHBC-1595"

$BBIF702459$`TUHBC-1010`
        aKO
"GHBC-1602"



$SPYO160490
$SPYO160490$`TUJ81-307`
 epsilonKO     betaKO    gammaKO    alphaKO    deltaKO        bKO        aKO
"GJ81-624" "GJ81-623" "GJ81-622" "GJ81-621" "GJ81-620" "GJ81-619" "GJ81-618"
       cKO
"GJ81-617"



$SSUI1005041
$SSUI1005041$`TULLO-550`
        cKO         aKO         bKO     deltaKO     alphaKO     gammaKO
"GLLO-1132" "GLLO-1131" "GLLO-1130" "GLLO-1129" "GLLO-1128" "GLLO-1127"
     betaKO   epsilonKO
"GLLO-1126" "GLLO-1125"



$`SPYO1235829-WGS`
$`SPYO1235829-WGS`$`TUSX7-317`
 epsilonKO     betaKO    gammaKO    alphaKO    deltaKO        bKO        aKO
"GSX7-625" "GSX7-624" "GSX7-623" "GSX7-622" "GSX7-621" "GSX7-620" "GSX7-619"
       cKO
"GSX7-618"



$`SPYO1336746-WGS`
$`SPYO1336746-WGS`$`TUSX8-312`
 epsilonKO     betaKO    gammaKO    alphaKO    deltaKO        bKO        aKO
"GSX8-618" "GSX8-617" "GSX8-616" "GSX8-615" "GSX8-614" "GSX8-613" "GSX8-612"
       cKO
"GSX8-611"



$`SPYO1207470-WGS`
$`SPYO1207470-WGS`$`TUSXA-303`
 epsilonKO     betaKO    gammaKO    alphaKO    deltaKO        bKO        aKO
"GSXA-557" "GSXA-556" "GSXA-555" "GSXA-554" "GSXA-553" "GSXA-552" "GSXA-551"

$`SPYO1207470-WGS`$noTU
cKO
 NA



$LMON879089
$LMON879089$`TULG3-49`
epsilonKO1    betaKO1   gammaKO1   alphaKO1   deltaKO1       cKO1
```

"GLG3-102" "GLG3-101" "GLG3-100" "GLG3-99" "GLG3-98" "GLG3-97"

$LMON879089$`TULG3-1343`
aKO cKO2 bKO deltaKO2 alphaKO2 gammaKO2
"GLG3-2645" "GLG3-2644" "GLG3-2643" "GLG3-2642" "GLG3-2641" "GLG3-2640"
betaKO2 epsilonKO2
"GLG3-2639" "GLG3-2638"

$SPYO293653
$SPYO293653$`TUHFC-319`
epsilonKO betaKO gammaKO alphaKO deltaKO bKO aKO
"GHFC-638" "GHFC-637" "GHFC-636" "GHFC-635" "GHFC-634" "GHFC-633" "GHFC-632"
cKO
"GHFC-631"

$SSP768493
$SSP768493$`TULK0-2712`
epsilonKO betaKO gammaKO alphaKO deltaKO bKO
"GLK0-5119" "GLK0-5118" "GLK0-5117" "GLK0-5116" "GLK0-5115" "GLK0-5114"
cKO aKO
"GLK0-5113" "GLK0-5112"

$`SRAP1343740-WGS`
$`SRAP1343740-WGS`$`TUSXN-2003`
aKO cKO bKO deltaKO alphaKO gammaKO
"GSXN-3411" "GSXN-3410" "GSXN-3409" "GSXN-3408" "GSXN-3407" "GSXN-3406"
betaKO epsilonKO
"GSXN-3405" "GSXN-3404"

$SRUM927704
$SRUM927704$`TULJZ-524`
epsilonKO betaKO gammaKO alphaKO deltaKO bKO cKO
"GLJZ-398" "GLJZ-397" "GLJZ-396" "GLJZ-395" "GLJZ-394" "GLJZ-393" "GLJZ-392"
aKO
"GLJZ-391"

$`SPLY682634-WGS`
$`SPLY682634-WGS`$`TUSV2-2606`
epsilonKO betaKO gammaKO alphaKO deltaKO bKO
"GSV2-4721" "GSV2-4720" "GSV2-4719" "GSV2-4718" "GSV2-4717" "GSV2-4716"
cKO aKO
"GSV2-4715" "GSV2-4714"

$SROS479432
$SROS479432$`TUI0V-970`
epsilonKO betaKO gammaKO alphaKO deltaKO bKO
"GI0V-1686" "GI0V-1685" "GI0V-1684" "GI0V-1683" "GI0V-1682" "GI0V-1681"
cKO aKO

```
"GI0V-1680" "GI0V-1679"


$SSUI1184252
$SSUI1184252$`TULLN-502`
       cKO         aKO         bKO     deltaKO     alphaKO     gammaKO
"GLLN-1027" "GLLN-1026" "GLLN-1025" "GLLN-1024" "GLLN-1023" "GLLN-1022"
    betaKO   epsilonKO
"GLLN-1021" "GLLN-1020"


$SSUI1004951
$SSUI1004951$`TULLP-409`
 epsilonKO     betaKO    gammaKO    alphaKO    deltaKO        bKO        aKO
"GLLP-813" "GLLP-812" "GLLP-811" "GLLP-810" "GLLP-809" "GLLP-808" "GLLP-807"
       cKO
"GLLP-806"


$SPLY768492
$SPLY768492$`TULK1-2712`
  epsilonKO      betaKO     gammaKO     alphaKO     deltaKO         bKO
"GLK1-5118" "GLK1-5117" "GLK1-5116" "GLK1-5115" "GLK1-5114" "GLK1-5113"
        cKO         aKO
"GLK1-5112" "GLK1-5111"


$SSP768490
$SSP768490$`TUH4I-2711`
  epsilonKO      betaKO     gammaKO     alphaKO     deltaKO         bKO
"GH4I-5119" "GH4I-5118" "GH4I-5117" "GH4I-5116" "GH4I-5115" "GH4I-5114"
        cKO         aKO
"GH4I-5113" "GH4I-5112"


$SROT640132
$SROT640132$`TUHEU-338`
 epsilonKO     betaKO    gammaKO    alphaKO        bKO        cKO        aKO
"GHEU-718" "GHEU-717" "GHEU-716" "GHEU-715" "GHEU-712" "GHEU-711" "GHEU-710"

$SROT640132$noTU
deltaKO
     NA


$`LMON882020-WGS`
$`LMON882020-WGS`$`TUSPZ-46`
epsilonKO1    betaKO1   gammaKO1   alphaKO1   deltaKO1       cKO1
 "GSPZ-93"  "GSPZ-92"  "GSPZ-91"  "GSPZ-90"  "GSPZ-89"  "GSPZ-88"

$`LMON882020-WGS`$`TUSPZ-1344`
        aKO        cKO2         bKO    deltaKO2    alphaKO2    gammaKO2
"GSPZ-2675" "GSPZ-2674" "GSPZ-2673" "GSPZ-2672" "GSPZ-2671" "GSPZ-2670"
    betaKO2  epsilonKO2
```

"GSPZ-2669" "GSPZ-2668"

$SRUB309807
$SRUB309807$`TUJJD-679`
gammaKO alphaKO deltaKO bKO cKO aKO
"GJJD-914" "GJJD-913" "GJJD-912" "GJJD-911" "GJJD-910" "GJJD-909"

$SRUB309807$`TUJJD-1757`
epsilonKO betaKO
"GJJD-2427" "GJJD-2426"

$`SPLY1348660-WGS`
$`SPLY1348660-WGS`$`TUSV3-2724`
epsilonKO betaKO gammaKO alphaKO deltaKO bKO
"GSV3-5103" "GSV3-5102" "GSV3-5101" "GSV3-5100" "GSV3-5099" "GSV3-5098"
cKO aKO
"GSV3-5097" "GSV3-5096"

$SSAN388919
$SSAN388919$`TUHEN-388`
epsilonKO betaKO gammaKO alphaKO deltaKO bKO aKO
"GHEN-791" "GHEN-790" "GHEN-789" "GHEN-788" "GHEN-787" "GHEN-786" "GHEN-785"
cKO
"GHEN-784"

$SSUI568814
$SSUI568814$`TUJD0-406`
epsilonKO betaKO gammaKO alphaKO deltaKO bKO aKO
"GJD0-836" "GJD0-835" "GJD0-834" "GJD0-833" "GJD0-832" "GJD0-831" "GJD0-830"
cKO
"GJD0-829"

$SPSE937773
$SPSE937773$`TUH0P-901`
aKO cKO bKO deltaKO alphaKO gammaKO
"GH0P-1839" "GH0P-1838" "GH0P-1837" "GH0P-1836" "GH0P-1835" "GH0P-1834"
betaKO epsilonKO
"GH0P-1833" "GH0P-1832"

$SAUR548473
$SAUR548473$`TULKT-618`
epsilonKO betaKO gammaKO alphaKO deltaKO bKO
"GLKT-1122" "GLKT-1121" "GLKT-1120" "GLKT-1119" "GLKT-1118" "GLKT-1117"
cKO aKO
"GLKT-1116" "GLKT-1115"

$SSED425104

$SSED425104$`TUH7Q-2861`
aKO cKO bKO deltaKO alphaKO gammaKO
"GH7Q-4650" "GH7Q-4649" "GH7Q-4648" "GH7Q-4647" "GH7Q-4646" "GH7Q-4645"
betaKO epsilonKO
"GH7Q-4644" "GH7Q-4643"

$SSUI993512
$SSUI993512$`TULLI-530`
cKO aKO bKO deltaKO alphaKO gammaKO
"GLLI-1090" "GLLI-1089" "GLLI-1088" "GLLI-1087" "GLLI-1086" "GLLI-1085"
betaKO epsilonKO
"GLLI-1084" "GLLI-1083"

$SSPU546271
$SSPU546271$`TUCBU-898`
aKO cKO bKO deltaKO alphaKO gammaKO
"GCBU-1888" "GCBU-1887" "GCBU-1886" "GCBU-1885" "GCBU-1884" "GCBU-1883"
betaKO epsilonKO
"GCBU-1882" "GCBU-1881"

$SSUI218494
$SSUI218494$`TUJDS-538`
cKO aKO bKO deltaKO alphaKO gammaKO
"GJDS-1066" "GJDS-1065" "GJDS-1064" "GJDS-1063" "GJDS-1062" "GJDS-1061"
betaKO epsilonKO
"GJDS-1060" "GJDS-1059"

$`SSON216599-WGS`
$`SSON216599-WGS`$`TUSV7-2506`
aKO cKO bKO deltaKO alphaKO gammaKO
"GSV7-4469" "GSV7-4468" "GSV7-4467" "GSV7-4466" "GSV7-4465" "GSV7-4464"
betaKO epsilonKO
"GSV7-4463" "GSV7-4462"

$`LMON930781-WGS`
$`LMON930781-WGS`$`TUSPS-552`
cKO1 deltaKO1 alphaKO1 gammaKO1 betaKO1 epsilonKO1
"GSPS-1013" "GSPS-1012" "GSPS-1011" "GSPS-1010" "GSPS-1009" "GSPS-1008"

$`LMON930781-WGS`$`TUSPS-783`
epsilonKO2 betaKO2 gammaKO2 alphaKO2 deltaKO2 bKO
"GSPS-1462" "GSPS-1461" "GSPS-1460" "GSPS-1459" "GSPS-1458" "GSPS-1457"
cKO2 aKO
"GSPS-1456" "GSPS-1455"

$SSUI1004952
$SSUI1004952$`TULLJ-390`
epsilonKO betaKO gammaKO alphaKO deltaKO bKO aKO

"GLLJ-795" "GLLJ-794" "GLLJ-793" "GLLJ-792" "GLLJ-791" "GLLJ-790" "GLLJ-789"
cKO
"GLLJ-788"

$SSON300269
$SSON300269$`TUJJF-2317`
epsilonKO betaKO gammaKO alphaKO deltaKO bKO
"GJJF-3883" "GJJF-3882" "GJJF-3881" "GJJF-3880" "GJJF-3879" "GJJF-3878"
cKO aKO
"GJJF-3877" "GJJF-3876"

$SSAP342451
$SSAP342451$`TUKFA-484`
epsilonKO betaKO gammaKO alphaKO deltaKO bKO cKO
"GKFA-794" "GKFA-793" "GKFA-792" "GKFA-791" "GKFA-790" "GKFA-789" "GKFA-788"
aKO
"GKFA-787"

$SSUI1005042
$SSUI1005042$`TULLK-657`
cKO aKO bKO deltaKO alphaKO gammaKO
"GLLK-1343" "GLLK-1342" "GLLK-1341" "GLLK-1340" "GLLK-1339" "GLLK-1338"
betaKO epsilonKO
"GLLK-1337" "GLLK-1336"

$SAUR378806
$SAUR378806$`TUCZI-483`
alphaKO
"GCZI-797"

$SAUR378806$`TUCZI-565`
epsilonKO betaKO
"GCZI-945" "GCZI-943"

$SAUR378806$`TUCZI-5646`
gammaKO
"GCZI-941"

$SAUR378806$`TUCZI-485`
deltaKO
"GCZI-799"

$SAUR378806$`TUCZI-4445`
aKO cKO bKO
"GCZI-8067" "GCZI-8066" "GCZI-8065"

$SSAL1048332
$SSAL1048332$`TUI6B-855`
cKO aKO bKO deltaKO alphaKO gammaKO

"GI6B-1584" "GI6B-1583" "GI6B-1582" "GI6B-1581" "GI6B-1580" "GI6B-1579"
betaKO epsilonKO
"GI6B-1578" "GI6B-1577"

$SSUI568813
$SSUI568813$`TUJFC-546`
cKO aKO bKO deltaKO alphaKO gammaKO
"GJFC-1122" "GJFC-1121" "GJFC-1120" "GJFC-1119" "GJFC-1118" "GJFC-1117"
betaKO epsilonKO
"GJFC-1116" "GJFC-1115"

$SSUI1007064
$SSUI1007064$`TUHXD-601`
cKO aKO bKO deltaKO alphaKO gammaKO
"GHXD-1219" "GHXD-1218" "GHXD-1217" "GHXD-1216" "GHXD-1215" "GHXD-1214"
betaKO epsilonKO
"GHXD-1213" "GHXD-1212"

$SSUI391295
$SSUI391295$`TUHI8-606`
aKO bKO deltaKO alphaKO gammaKO betaKO
"GHI8-1237" "GHI8-1236" "GHI8-1235" "GHI8-1234" "GHI8-1233" "GHI8-1232"
epsilonKO
"GHI8-1231"

$SSUI391295$`TUHI8-607`
cKO
"GHI8-1238"

$`SSUI1340847-WGS`
$`SSUI1340847-WGS`$`TUSXC-394`
epsilonKO betaKO gammaKO alphaKO deltaKO bKO aKO
"GSXC-821" "GSXC-820" "GSXC-819" "GSXC-818" "GSXC-817" "GSXC-816" "GSXC-815"
cKO
"GSXC-814"

$`SSUI1246365-WGS`
$`SSUI1246365-WGS`$`TUSXB-588`
cKO aKO bKO deltaKO alphaKO gammaKO
"GSXB-1092" "GSXB-1091" "GSXB-1090" "GSXB-1089" "GSXB-1088" "GSXB-1087"
betaKO epsilonKO
"GSXB-1086" "GSXB-1085"

$LMON1126011
$LMON1126011$`TULFQ-59`
epsilonKO1 betaKO1 gammaKO1 alphaKO1 deltaKO1 cKO1
"GLFQ-106" "GLFQ-105" "GLFQ-104" "GLFQ-103" "GLFQ-102" "GLFQ-101"

```
$LMON1126011$`TULFQ-1313`
        aKO        cKO2         bKO    deltaKO2    alphaKO2    gammaKO2
"GLFQ-2536" "GLFQ-2535" "GLFQ-2534" "GLFQ-2533" "GLFQ-2532" "GLFQ-2531"
    betaKO2  epsilonKO2
"GLFQ-2530" "GLFQ-2529"


$`SSUI1276647-WGS`
$`SSUI1276647-WGS`$`TUSXE-400`
 epsilonKO     betaKO    gammaKO    alphaKO    deltaKO        bKO        aKO
"GSXE-798" "GSXE-797" "GSXE-796" "GSXE-795" "GSXE-794" "GSXE-793" "GSXE-792"
       cKO
"GSXE-791"


$SSUI391296
$SSUI391296$`TUI2E-608`
        cKO        aKO2        aKO1         bKO     deltaKO     alphaKO
"GI2E-1248" "GI2E-1247" "GI2E-1246" "GI2E-1245" "GI2E-1244" "GI2E-1243"
    gammaKO      betaKO   epsilonKO
"GI2E-1242" "GI2E-1241" "GI2E-1240"


$SSUI423211
$SSUI423211$`TULLL-525`
        aKO         bKO     deltaKO     alphaKO     gammaKO      betaKO
"GLLL-1087" "GLLL-1086" "GLLL-1085" "GLLL-1084" "GLLL-1083" "GLLL-1082"
  epsilonKO
"GLLL-1081"

$SSUI423211$noTU
cKO
 NA


$SSP387093
$SSP387093$`TUH25-861`
       bKO2        bKO1     deltaKO     alphaKO     gammaKO      betaKO
"GH25-1799" "GH25-1798" "GH25-1797" "GH25-1796" "GH25-1795" "GH25-1794"
  epsilonKO
"GH25-1793"

$SSP387093$`TUH25-911`
        cKO
"GH25-1915"

$SSP387093$`TUH25-774`
        aKO
"GH25-1604"


$SUSI234267
$SUSI234267$`TUHSK-210`
 epsilonKO     betaKO    gammaKO    alphaKO    deltaKO       bKO2       bKO1
```

"GHSK-469" "GHSK-468" "GHSK-467" "GHSK-466" "GHSK-465" "GHSK-464" "GHSK-463"

$SUSI234267$`TUHSK-356`
cKO
"GHSK-772"

$SUSI234267$`TUHSK-355`
aKO
"GHSK-771"

$BBRO257310
$BBRO257310$`TU9TZ-34843`
bKO deltaKO alphaKO
"BB4609" "BB4608" "BB4607"

$BBRO257310$`TU9TZ-34841`
betaKO epsilonKO
"BB4605" "BB4604"

$BBRO257310$`TU9TZ-34842`
gammaKO
"BB4606"

$BBRO257310$`TU9TZ-34844`
cKO
"BB4610"

$BBRO257310$`TU9TZ-34845`
aKO
"BB4611"

$`BBRE326426-WGS`
$`BBRE326426-WGS`$`TUSGV-213`
epsilonKO betaKO gammaKO alphaKO deltaKO bKO cKO
"GSGV-339" "GSGV-338" "GSGV-337" "GSGV-336" "GSGV-335" "GSGV-334" "GSGV-333"
aKO
"GSGV-332"

$BBRE866777
$BBRE866777$`TUL99-205`
epsilonKO betaKO gammaKO alphaKO deltaKO bKO cKO
"GL99-328" "GL99-327" "GL99-326" "GL99-325" "GL99-324" "GL99-323" "GL99-322"
aKO
"GL99-321"

$`BCER222523-WGS`
$`BCER222523-WGS`$`TUSFX-3315`
aKO cKO bKO deltaKO alphaKO gammaKO
"GSFX-5565" "GSFX-5564" "GSFX-5563" "GSFX-5562" "GSFX-5561" "GSFX-5560"
betaKO epsilonKO

"GSFX-5559" "GSFX-5558"

$SSP862751
$SSP862751$`TUHMW-2690`
epsilonKO betaKO gammaKO alphaKO deltaKO bKO
"GHMW-4645" "GHMW-4644" "GHMW-4643" "GHMW-4642" "GHMW-4641" "GHMW-4640"
cKO aKO
"GHMW-4639" "GHMW-4638"

$SSP627192
$SSP627192$`TUI2Q-2093`
epsilonKO betaKO gammaKO alphaKO deltaKO
"GI2Q-3792" "GI2Q-3791" "GI2Q-3790" "GI2Q-3789" "GI2Q-3788"

$SSP627192$`TUI2Q-1300`
aKO cKO bKO2 bKO1
"GI2Q-2328" "GI2Q-2327" "GI2Q-2326" "GI2Q-2325"

$`SSYR1276229-WGS`
$`SSYR1276229-WGS`$`TUSVL-50`
epsilonKO betaKO gammaKO alphaKO deltaKO bKO cKO
"GSVL-112" "GSVL-111" "GSVL-110" "GSVL-109" "GSVL-108" "GSVL-107" "GSVL-106"
aKO
"GSVL-105"

$APLE416269
$APLE416269$`TUHV7-1018`
aKO cKO bKO deltaKO alphaKO gammaKO
"GHV7-1708" "GHV7-1707" "GHV7-1706" "GHV7-1705" "GHV7-1704" "GHV7-1703"
betaKO epsilonKO
"GHV7-1702" "GHV7-1701"

$SSYM568817
$SSYM568817$`TUI4K-2`
epsilonKO betaKO gammaKO alphaKO deltaKO bKO cKO aKO
"GI4K-8" "GI4K-7" "GI4K-6" "GI4K-5" "GI4K-4" "GI4K-3" "GI4K-2" "GI4K-1"

$`STAI1276220-WGS`
$`STAI1276220-WGS`$`TUSVM-25`
epsilonKO betaKO gammaKO alphaKO deltaKO bKO cKO aKO
"GSVM-59" "GSVM-58" "GSVM-57" "GSVM-56" "GSVM-55" "GSVM-54" "GSVM-53" "GSVM-52"

$SAUR1006543
$SAUR1006543$`TULKS-1114`
aKO cKO bKO deltaKO alphaKO gammaKO
"GLKS-2143" "GLKS-2142" "GLKS-2141" "GLKS-2140" "GLKS-2139" "GLKS-2138"
betaKO epsilonKO

"GLKS-2137" "GLKS-2136"

$SPAS981540
$SPAS981540$`TUJYA-384`
epsilonKO betaKO gammaKO alphaKO deltaKO bKO aKO
"GJYA-719" "GJYA-718" "GJYA-717" "GJYA-716" "GJYA-715" "GJYA-714" "GJYA-713"
cKO
"GJYA-712"

$STHE299768
$STHE299768$`TUHWB-278`
epsilonKO betaKO gammaKO alphaKO deltaKO bKO aKO
"GHWB-531" "GHWB-530" "GHWB-529" "GHWB-528" "GHWB-527" "GHWB-526" "GHWB-525"
cKO
"GHWB-524"

$SPSE1054460
$SPSE1054460$`TUHLA-752`
cKO aKO bKO deltaKO alphaKO gammaKO
"GHLA-1467" "GHLA-1466" "GHLA-1465" "GHLA-1464" "GHLA-1463" "GHLA-1462"
betaKO epsilonKO
"GHLA-1461" "GHLA-1460"

$STHE322159
$STHE322159$`TUJ9F-273`
epsilonKO betaKO gammaKO alphaKO deltaKO bKO aKO
"GJ9F-522" "GJ9F-521" "GJ9F-520" "GJ9F-519" "GJ9F-518" "GJ9F-517" "GJ9F-516"
cKO
"GJ9F-515"

$SSAL1046629
$SSAL1046629$`TULLG-932`
cKO aKO bKO deltaKO alphaKO gammaKO
"GLLG-1534" "GLLG-1533" "GLLG-1532" "GLLG-1531" "GLLG-1530" "GLLG-1529"
betaKO epsilonKO
"GLLG-1528" "GLLG-1527"

$SPYO798300
$SPYO798300$`TULLE-318`
epsilonKO betaKO gammaKO alphaKO deltaKO bKO aKO
"GLLE-610" "GLLE-609" "GLLE-608" "GLLE-607" "GLLE-606" "GLLE-605" "GLLE-604"
cKO
"GLLE-603"

$STHE292459
$STHE292459$`TUJMM-54`
epsilonKO betaKO gammaKO alphaKO deltaKO bKO cKO

```
"GJMM-108" "GJMM-107" "GJMM-106" "GJMM-105" "GJMM-104" "GJMM-103" "GJMM-102"
       aKO
"GJMM-101"


$STHE479434
$STHE479434$`TUHJN-822`
       aKO        cKO        bKO     deltaKO     alphaKO     gammaKO
"GHJN-1465" "GHJN-1464" "GHJN-1463" "GHJN-1462" "GHJN-1461" "GHJN-1460"
    betaKO   epsilonKO
"GHJN-1459" "GHJN-1458"


$SAUR1118959
$SAUR1118959$`TUJUR-1073`
       aKO        cKO        bKO     deltaKO     alphaKO     gammaKO
"GJUR-2046" "GJUR-2045" "GJUR-2044" "GJUR-2043" "GJUR-2042" "GJUR-2041"
    betaKO   epsilonKO
"GJUR-2040" "GJUR-2039"


$SAUR1028799
$SAUR1028799$`TUH9Q-1005`
       aKO        cKO        bKO     deltaKO     alphaKO     gammaKO
"GH9Q-1938" "GH9Q-1937" "GH9Q-1936" "GH9Q-1935" "GH9Q-1934" "GH9Q-1933"
    betaKO   epsilonKO
"GH9Q-1932" "GH9Q-1931"


$SAUR663951
$SAUR663951$`TULKU-1210`
       aKO        cKO        bKO     deltaKO     alphaKO     gammaKO
"GLKU-2334" "GLKU-2333" "GLKU-2332" "GLKU-2331" "GLKU-2330" "GLKU-2329"
    betaKO   epsilonKO
"GLKU-2328" "GLKU-2327"


$SAUR1074252
$SAUR1074252$`TULKL-1104`
       aKO        cKO        bKO     deltaKO     alphaKO     gammaKO
"GLKL-2105" "GLKL-2104" "GLKL-2103" "GLKL-2102" "GLKL-2101" "GLKL-2100"
    betaKO   epsilonKO
"GLKL-2099" "GLKL-2098"


$`10403S_RAST`
$`10403S_RAST`$`TUAB3-10161`
        bKO     deltaKO1     alphaKO1     gammaKO1
"LMRG_01715" "LMRG_01716" "LMRG_01717" "LMRG_01718"

$`10403S_RAST`$`TUAB3-8865`
  epsilonKO2      betaKO2     gammaKO2     alphaKO2     deltaKO2         cKO2
"LMRG_02342" "LMRG_02341" "LMRG_02340" "LMRG_02339" "LMRG_02969" "LMRG_02337"
```

```
$`10403S_RAST`$`TUAB3-10160`
     betaKO1   epsilonKO1
"LMRG_01719" "LMRG_01720"

$`10403S_RAST`$`TUAB3-10162`
        cKO1
"LMRG_01714"

$`10403S_RAST`$`TUAB3-10163`
         aKO
"LMRG_01713"


$SAUR703339
$SAUR703339$`TULKF-1077`
        aKO         cKO         bKO     deltaKO     alphaKO     gammaKO
"GLKF-2119" "GLKF-2118" "GLKF-2117" "GLKF-2116" "GLKF-2115" "GLKF-2114"
     betaKO   epsilonKO
"GLKF-2113" "GLKF-2112"


$SAUR1123523
$SAUR1123523$`TULKH-1102`
        aKO         cKO         bKO     deltaKO     alphaKO     gammaKO
"GLKH-2121" "GLKH-2120" "GLKH-2119" "GLKH-2118" "GLKH-2117" "GLKH-2116"
     betaKO   epsilonKO
"GLKH-2115" "GLKH-2114"


$`SVEN953739-WGS`
$`SVEN953739-WGS`$`TUSXO-2827`
  epsilonKO      betaKO     gammaKO     alphaKO     deltaKO         bKO
"GSXO-5097" "GSXO-5096" "GSXO-5095" "GSXO-5094" "GSXO-5093" "GSXO-5092"
        cKO         aKO
"GSXO-5091" "GSXO-5090"


$`WSUC273121-WGS`
$`WSUC273121-WGS`$`TUSYY-196`
 epsilonKO     betaKO    gammaKO    alphaKO    deltaKO       bKO2       bKO1
"GSYY-508" "GSYY-507" "GSYY-506" "GSYY-505" "GSYY-504" "GSYY-503" "GSYY-502"

$`WSUC273121-WGS`$`TUSYY-133`
       aKO
"GSYY-312"

$`WSUC273121-WGS`$noTU
cKO
 NA


$SVIR471857
$SVIR471857$`TUHAV-1561`
        aKO         cKO         bKO     deltaKO     alphaKO     gammaKO
```

"GHAV-2948" "GHAV-2947" "GHAV-2946" "GHAV-2945" "GHAV-2944" "GHAV-2943"
betaKO epsilonKO
"GHAV-2942" "GHAV-2941"

$SVIO653045
$SVIO653045$`TUHK6-1140`
epsilonKO betaKO gammaKO alphaKO deltaKO bKO
"GHK6-1458" "GHK6-1457" "GHK6-1456" "GHK6-1455" "GHK6-1454" "GHK6-1453"
cKO aKO
"GHK6-1452" "GHK6-1451"

$SVIO637905
$SVIO637905$`TUCRO-8`
aKO cKO bKO deltaKO alphaKO gammaKO
"GCRO-4510" "GCRO-4509" "GCRO-4508" "GCRO-4507" "GCRO-4506" "GCRO-4505"
betaKO epsilonKO
"GCRO-4504" "GCRO-4503"

$`SWAR1194526-WGS`
$`SWAR1194526-WGS`$`TUSW8-484`
epsilonKO betaKO gammaKO alphaKO deltaKO bKO cKO
"GSW8-793" "GSW8-792" "GSW8-791" "GSW8-790" "GSW8-789" "GSW8-788" "GSW8-787"
aKO
"GSW8-786"

$SWOO392500
$SWOO392500$`TUI2C-3036`
aKO cKO bKO deltaKO alphaKO gammaKO
"GI2C-5069" "GI2C-5068" "GI2C-5067" "GI2C-5066" "GI2C-5065" "GI2C-5064"
betaKO epsilonKO
"GI2C-5063" "GI2C-5062"

$SWIT392499
$SWIT392499$`TUHZK-611`
epsilonKO betaKO gammaKO alphaKO deltaKO
"GHZK-636" "GHZK-635" "GHZK-634" "GHZK-633" "GHZK-632"

$SWIT392499$`TUHZK-2537`
cKO aKO
"GHZK-4534" "GHZK-4533"

$SWIT392499$`TUHZK-2538`
bKO2 bKO1
"GHZK-4536" "GHZK-4535"

$SWOL335541
$SWOL335541$`TUHL1-1411`
bKO deltaKO alphaKO gammaKO betaKO epsilonKO

```
"GHL1-2449" "GHL1-2448" "GHL1-2447" "GHL1-2446" "GHL1-2445" "GHL1-2444"

$SWOL335541$`TUHL1-1412`
        aKO         cKO
"GHL1-2451" "GHL1-2450"



$LMON932919
$LMON932919$`TULG2-83`
epsilonKO1    betaKO1   gammaKO1   alphaKO1   deltaKO1       cKO1
"GLG2-101" "GLG2-100"  "GLG2-99"  "GLG2-98"  "GLG2-97"  "GLG2-96"

$LMON932919$`TULG2-1371`
        aKO        cKO2         bKO    deltaKO2    alphaKO2    gammaKO2
"GLG2-2618" "GLG2-2617" "GLG2-2616" "GLG2-2615" "GLG2-2614" "GLG2-2613"
    betaKO2  epsilonKO2
"GLG2-2612" "GLG2-2611"



$SPIE225849
$SPIE225849$`TUH6V-2`
        aKO         cKO         bKO     deltaKO     alphaKO     gammaKO
"GH6V-5020" "GH6V-5019" "GH6V-5018" "GH6V-5017" "GH6V-5016" "GH6V-5015"
     betaKO   epsilonKO
"GH6V-5014" "GH6V-5013"



$SSP110662
$SSP110662$`TUJ7R-1141`
        aKO         cKO        bKO2        bKO1     deltaKO     alphaKO
"GJ7R-2243" "GJ7R-2242" "GJ7R-2241" "GJ7R-2240" "GJ7R-2239" "GJ7R-2238"
    gammaKO
"GJ7R-2237"

$SSP110662$`TUJ7R-1136`
  epsilonKO      betaKO
"GJ7R-2222" "GJ7R-2221"



$BJAP224911
$BJAP224911$`TUJEJ-253`
   deltaKO    alphaKO    gammaKO     betaKO
"GJEJ-446" "GJEJ-445" "GJEJ-444" "GJEJ-443"

$BJAP224911$`TUJEJ-252`
 epsilonKO
"GJEJ-442"

$BJAP224911$`TUJEJ-716`
        aKO         cKO
"GJEJ-1198" "GJEJ-1197"

$BJAP224911$`TUJEJ-715`
       bKO2        bKO1
```

```
"GJEJ-1196" "GJEJ-1195"


$SSP316279
$SSP316279$`TUJCI-230`
  gammaKO   alphaKO   deltaKO     bKO2      bKO1       cKO       aKO
"GJCI-496" "GJCI-495" "GJCI-494" "GJCI-493" "GJCI-492" "GJCI-491" "GJCI-490"

$SSP316279$`TUJCI-233`
   betaKO  epsilonKO
"GJCI-511" "GJCI-510"


$SSP64471
$SSP64471$`TUIVP-1198`
       aKO        cKO       bKO2       bKO1     deltaKO     alphaKO
"GIVP-2318" "GIVP-2317" "GIVP-2316" "GIVP-2315" "GIVP-2314" "GIVP-2313"
   gammaKO
"GIVP-2312"

$SSP64471$`TUIVP-1189`
  epsilonKO     betaKO
"GIVP-2285" "GIVP-2284"


$SSP195253
$SSP195253$`TULM1-1375`
       cKO       bKO2       bKO1     deltaKO     alphaKO
"GLM1-2294" "GLM1-2293" "GLM1-2292" "GLM1-2291" "GLM1-2290"

$SSP195253$`TULM1-838`
    betaKO   epsilonKO
"GLM1-1405" "GLM1-1404"

$SSP195253$`TULM1-1059`
   gammaKO
"GLM1-1776"

$SSP195253$`TULM1-1376`
       aKO
"GLM1-2295"


$SSP1173263
$SSP1173263$`TULM0-1726`
       aKO        cKO       bKO2       bKO1     deltaKO     alphaKO
"GLM0-2785" "GLM0-2784" "GLM0-2783" "GLM0-2782" "GLM0-2781" "GLM0-2780"

$SSP1173263$`TULM0-700`
    betaKO   epsilonKO
"GLM0-1071" "GLM0-1070"

$SSP1173263$`TULM0-2136`
   gammaKO
```

```
"GLM0-3471"


$SSP1080230
$SSP1080230$`TULM3-112`
       aKO        cKO       bKO2       bKO1    deltaKO    alphaKO    gammaKO
"GLM3-164" "GLM3-163" "GLM3-162" "GLM3-161" "GLM3-160" "GLM3-159" "GLM3-158"

$SSP1080230$`TULM3-1081`
  epsilonKO      betaKO
"GLM3-1528" "GLM3-1527"


$SSP1080229
$SSP1080229$`TULM2-112`
       aKO        cKO       bKO2       bKO1    deltaKO    alphaKO    gammaKO
"GLM2-164" "GLM2-163" "GLM2-162" "GLM2-161" "GLM2-160" "GLM2-159" "GLM2-158"

$SSP1080229$`TULM2-1081`
  epsilonKO      betaKO
"GLM2-1528" "GLM2-1527"


$SSP1080228
$SSP1080228$`TULM4-112`
       aKO        cKO       bKO2       bKO1    deltaKO    alphaKO    gammaKO
"GLM4-164" "GLM4-163" "GLM4-162" "GLM4-161" "GLM4-160" "GLM4-159" "GLM4-158"

$SSP1080228$`TULM4-1081`
  epsilonKO      betaKO
"GLM4-1529" "GLM4-1528"


$TAFR484019
$TAFR484019$`TUJOH-184`
 epsilonKO     betaKO    gammaKO    alphaKO    deltaKO        bKO        cKO
"GJOH-585" "GJOH-584" "GJOH-583" "GJOH-582" "GJOH-581" "GJOH-580" "GJOH-579"
       aKO
"GJOH-578"


$LMON932920
$LMON932920$`TULFZ-76`
epsilonKO1    betaKO1   gammaKO1   alphaKO1   deltaKO1       cKO1
 "GLFZ-93"  "GLFZ-92"  "GLFZ-91"  "GLFZ-90"  "GLFZ-89"  "GLFZ-88"

$LMON932920$`TULFZ-1379`
        aKO        cKO2         bKO    deltaKO2    alphaKO2    gammaKO2
"GLFZ-2675" "GLFZ-2674" "GLFZ-2673" "GLFZ-2672" "GLFZ-2671" "GLFZ-2670"
    betaKO2  epsilonKO2
"GLFZ-2669" "GLFZ-2668"


$TALB638303
```

```
$TALB638303$`TUHKY-348`
    alphaKO
"GHKY-1238"

$TALB638303$`TUHKY-372`
     betaKO     gammaKO
"GHKY-1332" "GHKY-1331"

$TALB638303$`TUHKY-172`
      bKO2       bKO1    deltaKO
"GHKY-572" "GHKY-571" "GHKY-570"

$TALB638303$`TUHKY-342`
  epsilonKO
"GHKY-1204"

$TALB638303$`TUHKY-345`
        aKO         cKO
"GHKY-1228" "GHKY-1227"


$BSP114615
$BSP114615$`TUJN5-241`
 epsilonKO     betaKO    gammaKO    alphaKO    deltaKO
"GJN5-399" "GJN5-398" "GJN5-397" "GJN5-396" "GJN5-395"

$BSP114615$`TUJN5-3708`
       bKO2        bKO1         cKO         aKO
"GJN5-6383" "GJN5-6382" "GJN5-6381" "GJN5-6380"


$TAMM648996
$TAMM648996$`TUI3X-635`
  epsilonKO      betaKO     gammaKO     alphaKO     deltaKO        bKO2
"GI3X-1718" "GI3X-1717" "GI3X-1716" "GI3X-1715" "GI3X-1714" "GI3X-1713"
       bKO1
"GI3X-1712"

$TAMM648996$`TUI3X-616`
        cKO         aKO
"GI3X-1651" "GI3X-1650"


$TASI1008459
$TASI1008459$`TUH5G-62`
 epsilonKO     betaKO    gammaKO    alphaKO    deltaKO        bKO        cKO
"GH5G-156" "GH5G-155" "GH5G-154" "GH5G-153" "GH5G-152" "GH5G-151" "GH5G-150"
       aKO
"GH5G-149"


$BCER347495
$BCER347495$`TUHGC-3185`
        aKO         cKO         bKO     deltaKO     alphaKO
```

"GHGC-5328" "GHGC-5327" "GHGC-5326" "GHGC-5325" "GHGC-5324"

$BCER347495$`TUHGC-3184`
gammaKO betaKO epsilonKO
"GHGC-5322" "GHGC-5321" "GHGC-5320"

$BCER405531
$BCER405531$`TUI5L-3444`
aKO cKO bKO deltaKO alphaKO gammaKO
"GI5L-5435" "GI5L-5434" "GI5L-5433" "GI5L-5432" "GI5L-5431" "GI5L-5430"
betaKO epsilonKO
"GI5L-5429" "GI5L-5428"

$BCEN331272
$BCEN331272$`TUHR7-145`
epsilonKO1 betaKO gammaKO1 alphaKO deltaKO bKO cKO
"GHR7-111" "GHR7-110" "GHR7-109" "GHR7-108" "GHR7-107" "GHR7-106" "GHR7-105"
aKO
"GHR7-104"

$BCEN331272$`TUHR7-2949`
epsilonKO2 gammaKO2
"GHR7-4948" "GHR7-4947"

$`CBLO1240471-WGS`
$`CBLO1240471-WGS`$`TUSI1-2`
epsilonKO betaKO gammaKO alphaKO deltaKO bKO cKO aKO
"GSI1-9" "GSI1-8" "GSI1-7" "GSI1-6" "GSI1-5" "GSI1-4" "GSI1-3" "GSI1-2"

$`TASI1091495-WGS`
$`TASI1091495-WGS`$`TUSYG-294`
epsilonKO betaKO gammaKO alphaKO deltaKO bKO cKO
"GSYG-727" "GSYG-726" "GSYG-725" "GSYG-724" "GSYG-723" "GSYG-722" "GSYG-721"
aKO
"GSYG-720"

$TAUE595494
$TAUE595494$`TUHEF-1662`
aKO cKO bKO deltaKO alphaKO gammaKO
"GHEF-3260" "GHEF-3259" "GHEF-3258" "GHEF-3257" "GHEF-3256" "GHEF-3255"
betaKO epsilonKO
"GHEF-3254" "GHEF-3253"

$TAZO545695
$TAZO545695$`TUHPL-1295`
epsilonKO betaKO gammaKO alphaKO deltaKO bKO
"GHPL-3155" "GHPL-3154" "GHPL-3153" "GHPL-3152" "GHPL-3151" "GHPL-3150"
cKO aKO

"GHPL-3149" "GHPL-3148"

$LMON637381
$LMON637381$`TUH7Z-1385`
aKO cKO1 bKO deltaKO1 alphaKO1 gammaKO1
"GH7Z-2741" "GH7Z-2740" "GH7Z-2739" "GH7Z-2738" "GH7Z-2737" "GH7Z-2736"
betaKO1 epsilonKO1
"GH7Z-2735" "GH7Z-2734"

$LMON637381$`TUH7Z-1502`
cKO2 deltaKO2 alphaKO2 gammaKO2 betaKO2 epsilonKO2
"GH7Z-2946" "GH7Z-2945" "GH7Z-2944" "GH7Z-2943" "GH7Z-2942" "GH7Z-2941"

$`CCOL1358410-WGS`
$`CCOL1358410-WGS`$`TUSHT-588`
bKO2 bKO1 deltaKO alphaKO gammaKO betaKO
"GSHT-1599" "GSHT-1598" "GSHT-1597" "GSHT-1596" "GSHT-1595" "GSHT-1594"
epsilonKO
"GSHT-1593"

$`CCOL1358410-WGS`$`TUSHT-334`
cKO
"GSHT-906"

$`CCOL1358410-WGS`$`TUSHT-420`
aKO
"GSHT-1140"

$TDEN292415
$TDEN292415$`TUHWG-1278`
aKO cKO bKO deltaKO alphaKO gammaKO
"GHWG-2855" "GHWG-2854" "GHWG-2853" "GHWG-2852" "GHWG-2851" "GHWG-2850"
betaKO epsilonKO2
"GHWG-2849" "GHWG-2848"

$TDEN292415$`TUHWG-784`
epsilonKO1
"GHWG-1778"

$TBIS469371
$TBIS469371$`TUHSI-519`
epsilonKO betaKO gammaKO alphaKO deltaKO bKO cKO
"GHSI-918" "GHSI-917" "GHSI-916" "GHSI-915" "GHSI-914" "GHSI-913" "GHSI-912"
aKO
"GHSI-911"

$`TCOM717605-WGS`
$`TCOM717605-WGS`$`TUSY5-2194`
aKO cKO bKO deltaKO alphaKO gammaKO

"GSY5-3858" "GSY5-3857" "GSY5-3856" "GSY5-3855" "GSY5-3854" "GSY5-3853"
betaKO epsilonKO
"GSY5-3852" "GSY5-3851"

$TCUR471852
$TCUR471852$`TUHHD-2404`
aKO cKO bKO deltaKO alphaKO gammaKO
"GHHD-3986" "GHHD-3985" "GHHD-3984" "GHHD-3983" "GHHD-3982" "GHHD-3981"
betaKO epsilonKO
"GHHD-3980" "GHHD-3979"

$TCRU317025
$TCRU317025$`TUHE8-1123`
aKO cKO bKO deltaKO alphaKO gammaKO
"GHE8-2229" "GHE8-2228" "GHE8-2227" "GHE8-2226" "GHE8-2225" "GHE8-2224"
betaKO epsilonKO
"GHE8-2223" "GHE8-2222"

$SDEN326298
$SDEN326298$`TUH9P-561`
bKO2 bKO1 deltaKO alphaKO gammaKO betaKO
"GH9P-1456" "GH9P-1455" "GH9P-1454" "GH9P-1453" "GH9P-1452" "GH9P-1451"
epsilonKO
"GH9P-1450"

$SDEN326298$`TUH9P-176`
cKO
"GH9P-445"

$SDEN326298$`TUH9P-535`
aKO
"GH9P-1387"

$TEQU743973
$TEQU743973$`TULM6-62`
epsilonKO betaKO gammaKO alphaKO deltaKO bKO cKO
"GLM6-163" "GLM6-162" "GLM6-161" "GLM6-160" "GLM6-159" "GLM6-158" "GLM6-157"
aKO
"GLM6-156"

$`TEQU1091497-WGS`
$`TEQU1091497-WGS`$`TUSY1-438`
epsilonKO betaKO gammaKO alphaKO deltaKO bKO
"GSY1-1145" "GSY1-1144" "GSY1-1143" "GSY1-1142" "GSY1-1141" "GSY1-1140"
cKO aKO
"GSY1-1139" "GSY1-1138"

$TEQU937774

```
$TEQU937774$`TUHXS-329`
 epsilonKO     betaKO    gammaKO    alphaKO    deltaKO        bKO        cKO
"GHXS-758" "GHXS-757" "GHXS-756" "GHXS-755" "GHXS-754" "GHXS-753" "GHXS-752"
       aKO
"GHXS-751"


$TERY203124
$TERY203124$`TUJDR-1701`
        aKO         cKO        bKO2        bKO1     deltaKO     alphaKO
"GJDR-2225" "GJDR-2224" "GJDR-2223" "GJDR-2222" "GJDR-2221" "GJDR-2220"
    gammaKO
"GJDR-2219"

$TERY203124$`TUJDR-2624`
  epsilonKO      betaKO
"GJDR-3418" "GJDR-3417"


$CJEJ1211776
$CJEJ1211776$`TULAA-52`
 epsilonKO     betaKO    gammaKO    alphaKO    deltaKO       bKO2       bKO1
"GLAA-104" "GLAA-103" "GLAA-102" "GLAA-101" "GLAA-100"  "GLAA-99"  "GLAA-98"

$CJEJ1211776$`TULAA-328`
       cKO
"GLAA-904"

$CJEJ1211776$`TULAA-417`
        aKO
"GLAA-1175"


$`LMON863767-WGS`
$`LMON863767-WGS`$`TUSPY-83`
epsilonKO1    betaKO1   gammaKO1   alphaKO1   deltaKO1       cKO1
"GSPY-102" "GSPY-101" "GSPY-100"  "GSPY-99"  "GSPY-98"  "GSPY-97"

$`LMON863767-WGS`$`TUSPY-1366`
        aKO        cKO2         bKO    deltaKO2    alphaKO2    gammaKO2
"GSPY-2616" "GSPY-2615" "GSPY-2614" "GSPY-2613" "GSPY-2612" "GSPY-2611"
    betaKO2  epsilonKO2
"GSPY-2610" "GSPY-2609"


$TFOR203275
$TFOR203275$`TUHRQ-334`
    betaKO  epsilonKO        aKO        cKO        bKO    deltaKO    alphaKO
"GHRQ-663" "GHRQ-662" "GHRQ-660" "GHRQ-659" "GHRQ-658" "GHRQ-657" "GHRQ-656"
   gammaKO
"GHRQ-655"


$TFUS269800
```

$TFUS269800$`TUI42-1514`
aKO cKO bKO deltaKO alphaKO gammaKO
"GI42-2440" "GI42-2439" "GI42-2438" "GI42-2437" "GI42-2436" "GI42-2435"
betaKO
"GI42-2434"

$TFUS269800$`TUI42-1513`
epsilonKO
"GI42-2433"

$TSUL396588
$TSUL396588$`TUH5B-1857`
aKO cKO bKO deltaKO alphaKO gammaKO
"GH5B-3363" "GH5B-3362" "GH5B-3361" "GH5B-3360" "GH5B-3359" "GH5B-3358"
betaKO epsilonKO
"GH5B-3357" "GH5B-3356"

$CEND1193729
$CEND1193729$`TULM8-427`
betaKO gammaKO alphaKO deltaKO
"GLM8-675" "GLM8-674" "GLM8-673" "GLM8-672"

$CEND1193729$`TULM8-428`
epsilonKO
"GLM8-676"

$CEND1193729$`TULM8-181`
aKO cKO
"GLM8-286" "GLM8-285"

$CEND1193729$`TULM8-180`
bKO2 bKO1
"GLM8-284" "GLM8-283"

$`TARS426114-WGS`
$`TARS426114-WGS`$`TUSYJ-1575`
aKO cKO bKO deltaKO alphaKO
"GSYJ-3176" "GSYJ-3175" "GSYJ-3174" "GSYJ-3173" "GSYJ-3172"

$`TARS426114-WGS`$`TUSYJ-1574`
gammaKO betaKO epsilonKO
"GSYJ-3171" "GSYJ-3170" "GSYJ-3169"

$THAL945021
$THAL945021$`TUJV6-1011`
aKO cKO bKO deltaKO alphaKO gammaKO
"GJV6-1785" "GJV6-1784" "GJV6-1783" "GJV6-1782" "GJV6-1781" "GJV6-1780"
betaKO epsilonKO
"GJV6-1779" "GJV6-1778"

```
$TIND667014
$TIND667014$`TUI6G-577`
  epsilonKO     betaKO    gammaKO    alphaKO    deltaKO      bKO2
"GI6G-1368" "GI6G-1367" "GI6G-1366" "GI6G-1365" "GI6G-1364" "GI6G-1363"
       bKO1
"GI6G-1362"

$TIND667014$`TUI6G-569`
        cKO        aKO
"GI6G-1340" "GI6G-1339"


$TINT75379
$TINT75379$`TUH6C-1421`
        aKO        cKO        bKO    deltaKO    alphaKO    gammaKO
"GH6C-2801" "GH6C-2800" "GH6C-2799" "GH6C-2798" "GH6C-2797" "GH6C-2796"
     betaKO   epsilonKO
"GH6C-2795" "GH6C-2794"


$TITA580331
$TITA580331$`TUHTM-303`
 epsilonKO    betaKO   gammaKO   alphaKO   deltaKO      bKO      cKO
"GHTM-658" "GHTM-657" "GHTM-656" "GHTM-655" "GHTM-654" "GHTM-653" "GHTM-652"
       aKO
"GHTM-651"


$CJEJ192222
$CJEJ192222$`TUJTS-52`
 epsilonKO    betaKO   gammaKO   alphaKO   deltaKO     bKO2      bKO1
"GJTS-104" "GJTS-103" "GJTS-102" "GJTS-101" "GJTS-100"  "GJTS-99"  "GJTS-98"

$CJEJ192222$`TUJTS-329`
       cKO
"GJTS-904"

$CJEJ192222$`TUJTS-418`
        aKO
"GJTS-1175"


$TPOT635013
$TPOT635013$`TUHIM-1578`
        aKO        cKO        bKO    deltaKO    alphaKO    gammaKO
"GHIM-2957" "GHIM-2956" "GHIM-2955" "GHIM-2954" "GHIM-2953" "GHIM-2952"
     betaKO   epsilonKO
"GHIM-2951" "GHIM-2950"


$LPNE933093
$LPNE933093$`TUH6R-1641`
        aKO        cKO        bKO    deltaKO    alphaKO
```

"GH6R-2964" "GH6R-2963" "GH6R-2962" "GH6R-2961" "GH6R-2960"

$LPNE933093$`TUH6R-1640`
gammaKO betaKO epsilonKO
"GH6R-2959" "GH6R-2958" "GH6R-2957"

$TSP396595
$TSP396595$`TUH8D-1503`
aKO cKO bKO deltaKO alphaKO gammaKO
"GH8D-2649" "GH8D-2648" "GH8D-2647" "GH8D-2646" "GH8D-2645" "GH8D-2644"
betaKO epsilonKO
"GH8D-2643" "GH8D-2642"

$TLET416591
$TLET416591$`TUI64-62`
epsilonKO betaKO gammaKO alphaKO deltaKO bKO cKO
"GI64-170" "GI64-169" "GI64-168" "GI64-167" "GI64-166" "GI64-165" "GI64-164"
aKO
"GI64-163"

$`TMOB765912-WGS`
$`TMOB765912-WGS`$`TUSYI-683`
epsilonKO betaKO gammaKO alphaKO deltaKO bKO
"GSYI-1171" "GSYI-1170" "GSYI-1169" "GSYI-1168" "GSYI-1167" "GSYI-1166"
cKO aKO
"GSYI-1165" "GSYI-1164"

$TMEL391009
$TMEL391009$`TUHM1-93`
aKO cKO bKO deltaKO alphaKO gammaKO betaKO
"GHM1-311" "GHM1-310" "GHM1-309" "GHM1-308" "GHM1-307" "GHM1-306" "GHM1-305"
epsilonKO
"GHM1-304"

$TMAR644966
$TMAR644966$`TUHKT-92`
epsilonKO betaKO gammaKO alphaKO deltaKO bKO cKO
"GHKT-195" "GHKT-194" "GHKT-193" "GHKT-192" "GHKT-191" "GHKT-190" "GHKT-189"
aKO
"GHKT-188"

$TMAT583358
$TMAT583358$`TUHOX-331`
epsilonKO betaKO gammaKO alphaKO deltaKO bKO cKO
"GHOX-726" "GHOX-725" "GHOX-724" "GHOX-723" "GHOX-722" "GHOX-721" "GHOX-720"
aKO
"GHOX-719"

```
$TSP85643
$TSP85643$`TUHEJ-179`
 epsilonKO     betaKO    gammaKO    alphaKO    deltaKO        bKO        cKO 
"GHEJ-215" "GHEJ-214" "GHEJ-213" "GHEJ-212" "GHEJ-211" "GHEJ-210" "GHEJ-209" 
       aKO 
"GHEJ-208" 


$TNEA309803
$TNEA309803$`TUJFG-295`
 epsilonKO     betaKO    gammaKO    alphaKO    deltaKO        bKO        cKO 
"GJFG-876" "GJFG-875" "GJFG-874" "GJFG-873" "GJFG-872" "GJFG-871" "GJFG-870" 
       aKO 
"GJFG-869" 


$`CJEJ1357994-WGS`
$`CJEJ1357994-WGS`$`TUSHU-54`
 epsilonKO     betaKO    gammaKO    alphaKO    deltaKO       bKO2       bKO1 
"GSHU-107" "GSHU-106" "GSHU-105" "GSHU-104" "GSHU-103" "GSHU-102" "GSHU-101" 

$`CJEJ1357994-WGS`$`TUSHU-362`
       cKO 
"GSHU-954" 

$`CJEJ1357994-WGS`$`TUSHU-452`
        aKO 
"GSHU-1213" 


$TNAP590168
$TNAP590168$`TUC5O-373`
  epsilonKO      betaKO     gammaKO     alphaKO     deltaKO         bKO 
"GC5O-1234" "GC5O-1233" "GC5O-1232" "GC5O-1231" "GC5O-1230" "GC5O-1229" 
        cKO         aKO 
"GC5O-1228" "GC5O-1227" 


$LBUC511437
$LBUC511437$`TUJNY-575`
 epsilonKO     betaKO    gammaKO    alphaKO    deltaKO        bKO        cKO 
"GJNY-915" "GJNY-914" "GJNY-913" "GJNY-912" "GJNY-911" "GJNY-910" "GJNY-909" 
       aKO 
"GJNY-908" 


$LPNE297245
$LPNE297245$`TUJD4-1853`
        aKO         cKO         bKO     deltaKO     alphaKO     gammaKO 
"GJD4-3159" "GJD4-3158" "GJD4-3157" "GJD4-3156" "GJD4-3155" "GJD4-3154" 
     betaKO   epsilonKO 
"GJD4-3153" "GJD4-3152" 
```

$TNAR747365
$TNAR747365$`TUH4R-227`
 epsilonKO   betaKO   gammaKO   alphaKO   deltaKO   bKO   cKO
"GH4R-616" "GH4R-615" "GH4R-614" "GH4R-613" "GH4R-612" "GH4R-611" "GH4R-610"
       aKO
"GH4R-609"

$TOCE555079
$TOCE555079$`TUHIS-975`
        aKO   cKO   bKO   deltaKO   alphaKO   gammaKO
"GHIS-2072" "GHIS-2071" "GHIS-2070" "GHIS-2069" "GHIS-2068" "GHIS-2067"
     betaKO   epsilonKO
"GHIS-2066" "GHIS-2065"

$BCIC374463
$BCIC374463$`TUI6Q-87`
       aKO   cKO   bKO   deltaKO   alphaKO   gammaKO   betaKO
"GI6Q-147" "GI6Q-146" "GI6Q-145" "GI6Q-144" "GI6Q-143" "GI6Q-142" "GI6Q-141"
 epsilonKO
"GI6Q-140"

$BCEN216591
$BCEN216591$`TUJI4-103`
epsilonKO   betaKO   gammaKO   alphaKO   deltaKO   bKO   cKO   aKO
"GJI4-38" "GJI4-37" "GJI4-36" "GJI4-35" "GJI4-34" "GJI4-33" "GJI4-32" "GJI4-31"

$BCOA941639
$BCOA941639$`TUHC1-1655`
        aKO   bKO   deltaKO   alphaKO   gammaKO   betaKO
"GHC1-2874" "GHC1-2873" "GHC1-2872" "GHC1-2871" "GHC1-2870" "GHC1-2869"
  epsilonKO
"GHC1-2868"

$BCOA941639$noTU
cKO
 NA

$BCLA66692
$BCLA66692$`TUHMP-2070`
        aKO   cKO   bKO   deltaKO   alphaKO   gammaKO
"GHMP-3937" "GHMP-3936" "GHMP-3935" "GHMP-3934" "GHMP-3933" "GHMP-3932"
     betaKO   epsilonKO
"GHMP-3931" "GHMP-3930"

$`TOLE1298593-WGS`
$`TOLE1298593-WGS`$`TUSY4-1913`
        aKO   cKO   bKO   deltaKO   alphaKO   gammaKO2

```
"GSY4-3700" "GSY4-3699" "GSY4-3698" "GSY4-3697" "GSY4-3696" "GSY4-3695"
     betaKO
"GSY4-3694"

$`TOLE1298593-WGS`$`TUSY4-869`
   gammaKO1
"GSY4-1681"

$`TOLE1298593-WGS`$`TUSY4-1912`
  epsilonKO
"GSY4-3693"


$ALIP862719
$ALIP862719$`TUJAA-3266`
    deltaKO    alphaKO1
"GJAA-2417" "GJAA-2416"

$ALIP862719$`TUJAA-1581`
    betaKO2  epsilonKO2        aKO2        cKO2        bKO3    alphaKO2
"GJAA-5011" "GJAA-5010" "GJAA-5007" "GJAA-5006" "GJAA-5005" "GJAA-5004"
   gammaKO2
"GJAA-5003"

$ALIP862719$`TUJAA-3264`
    betaKO1
"GJAA-2414"

$ALIP862719$`TUJAA-3265`
   gammaKO1
"GJAA-2415"

$ALIP862719$`TUJAA-3263`
 epsilonKO1
"GJAA-2413"

$ALIP862719$`TUJAA-2209`
      cKO1
"GJAA-602"

$ALIP862719$`TUJAA-2210`
      aKO1
"GJAA-603"

$ALIP862719$`TUJAA-2208`
      bKO2       bKO1
"GJAA-601" "GJAA-600"


$`CJEJ1380767-WGS`
$`CJEJ1380767-WGS`$`TUSHV-54`
 epsilonKO     betaKO    gammaKO    alphaKO    deltaKO      bKO2       bKO1
"GSHV-101" "GSHV-100"  "GSHV-99"  "GSHV-98"  "GSHV-97"  "GSHV-96"  "GSHV-95"
```

```
$`CJEJ1380767-WGS`$`TUSHV-344`
      cKO
"GSHV-903"

$`CJEJ1380767-WGS`$`TUSHV-439`
       aKO
"GSHV-1166"



$TSP795359
$TSP795359$`TUI1V-550`
      bKO2       bKO1     deltaKO     alphaKO     gammaKO      betaKO
"GI1V-1600" "GI1V-1599" "GI1V-1598" "GI1V-1597" "GI1V-1596" "GI1V-1595"
  epsilonKO
"GI1V-1594"

$TSP795359$`TUI1V-307`
      cKO       aKO
"GI1V-914" "GI1V-913"



$TPRI545694
$TPRI545694$`TUH5L-1289`
       aKO        cKO        bKO     deltaKO     alphaKO     gammaKO
"GH5L-2774" "GH5L-2773" "GH5L-2772" "GH5L-2771" "GH5L-2770" "GH5L-2769"
    betaKO   epsilonKO
"GH5L-2768" "GH5L-2767"



$TPAU521096
$TPAU521096$`TUI2W-642`
  epsilonKO      betaKO     gammaKO     alphaKO        bKO        cKO
"GI2W-1265" "GI2W-1264" "GI2W-1263" "GI2W-1262" "GI2W-1260" "GI2W-1259"
       aKO
"GI2W-1258"

$TPAU521096$noTU
deltaKO
    NA



$`LPAR537973-HMP`
$`LPAR537973-HMP`$`TUML4-329`
   deltaKO    alphaKO    gammaKO     betaKO  epsilonKO
"GML4-660" "GML4-659" "GML4-658" "GML4-657" "GML4-656"

$`LPAR537973-HMP`$`TUML4-330`
      aKO       cKO       bKO
"GML4-663" "GML4-662" "GML4-661"



$TPET390874
$TPET390874$`TUHJI-364`
  epsilonKO      betaKO     gammaKO     alphaKO     deltaKO        bKO
```

"GHJI-1217" "GHJI-1216" "GHJI-1215" "GHJI-1214" "GHJI-1213" "GHJI-1212"
cKO aKO
"GHJI-1211" "GHJI-1210"

$TPAR869212
$TPAR869212$`TULMM-1252`
epsilonKO betaKO gammaKO alphaKO deltaKO bKO
"GLMM-2443" "GLMM-2442" "GLMM-2441" "GLMM-2440" "GLMM-2439" "GLMM-2438"
cKO aKO
"GLMM-2437" "GLMM-2436"

$TPHA1089553
$TPHA1089553$`TULM9-1369`
aKO cKO bKO deltaKO alphaKO gammaKO
"GLM9-2720" "GLM9-2719" "GLM9-2718" "GLM9-2717" "GLM9-2716" "GLM9-2715"
betaKO epsilonKO
"GLM9-2714" "GLM9-2713"

$TROS309801
$TROS309801$`TUI0S-1009`
aKO cKO bKO deltaKO alphaKO gammaKO
"GI0S-1222" "GI0S-1221" "GI0S-1220" "GI0S-1219" "GI0S-1218" "GI0S-1217"
betaKO epsilonKO
"GI0S-1216" "GI0S-1215"

$ABAU1100841
$ABAU1100841$`TUL7W-234`
epsilonKO betaKO gammaKO alphaKO deltaKO bKO cKO
"GL7W-392" "GL7W-391" "GL7W-390" "GL7W-389" "GL7W-388" "GL7W-387" "GL7W-386"
aKO
"GL7W-385"

$`ABAU1096995-WGS`
$`ABAU1096995-WGS`$`TUSEL-169`
epsilonKO betaKO gammaKO alphaKO deltaKO bKO cKO
"GSEL-202" "GSEL-201" "GSEL-200" "GSEL-199" "GSEL-198" "GSEL-197" "GSEL-196"
aKO
"GSEL-195"

$`CJEJ1383068-WGS`
$`CJEJ1383068-WGS`$`TUSHX-73`
epsilonKO betaKO gammaKO alphaKO deltaKO bKO2 bKO1
"GSHX-100" "GSHX-99" "GSHX-98" "GSHX-97" "GSHX-96" "GSHX-95" "GSHX-94"

$`CJEJ1383068-WGS`$`TUSHX-392`
cKO
"GSHX-943"

```
$`CJEJ1383068-WGS`$`TUSHX-490`
       aKO 
"GSHX-1204" 


$ABAU509170
$ABAU509170$`TUCL9-5242`
  epsilonKO      betaKO     gammaKO     alphaKO     deltaKO         bKO 
"GCL9-2908" "GCL9-2909" "GCL9-2912" "GCL9-2906" "GCL9-2913" "GCL9-2911" 
       cKO         aKO 
"GCL9-2910" "GCL9-2907" 


$ABAU480119
$ABAU480119$`TUHQY-123`
 epsilonKO     betaKO    gammaKO    alphaKO    deltaKO        bKO        cKO 
"GHQY-200" "GHQY-199" "GHQY-198" "GHQY-197" "GHQY-196" "GHQY-195" "GHQY-194" 
      aKO 
"GHQY-193" 


$TSP126740
$TSP126740$`TUH49-386`
       aKO         cKO         bKO     deltaKO     alphaKO     gammaKO 
"GH49-1321" "GH49-1320" "GH49-1319" "GH49-1318" "GH49-1317" "GH49-1316" 
    betaKO   epsilonKO 
"GH49-1315" "GH49-1314" 


$TROS926566
$TROS926566$`TULM7-2225`
      bKO2        bKO1     deltaKO     alphaKO     gammaKO      betaKO 
"GLM7-3853" "GLM7-3852" "GLM7-3851" "GLM7-3850" "GLM7-3849" "GLM7-3848" 
  epsilonKO 
"GLM7-3847" 

$TROS926566$`TULM7-901`
       aKO 
"GLM7-1563" 

$TROS926566$noTU
cKO 
 NA 


$LPLA644042
$LPLA644042$`TUHFY-1108`
       bKO     deltaKO     alphaKO     gammaKO      betaKO   epsilonKO 
"GHFY-2048" "GHFY-2047" "GHFY-2046" "GHFY-2045" "GHFY-2044" "GHFY-2043" 

$LPLA644042$`TUHFY-1109`
       aKO         cKO 
"GHFY-2050" "GHFY-2049" 
```

$TSAA401053
$TSAA401053$`TUHYY-1801`
bKO2 bKO1 deltaKO alphaKO gammaKO betaKO
"GHYY-3308" "GHYY-3307" "GHYY-3306" "GHYY-3305" "GHYY-3303" "GHYY-3302"
epsilonKO
"GHYY-3301"

$TSAA401053$`TUHYY-1351`
aKO cKO
"GHYY-2516" "GHYY-2515"

$TSAC1094508
$TSAC1094508$`TULMA-806`
epsilonKO betaKO gammaKO alphaKO deltaKO bKO
"GLMA-1448" "GLMA-1447" "GLMA-1446" "GLMA-1445" "GLMA-1444" "GLMA-1443"
cKO aKO
"GLMA-1442" "GLMA-1441"

$TTHE688269
$TTHE688269$`TUHXV-109`
epsilonKO betaKO gammaKO alphaKO deltaKO bKO cKO
"GHXV-290" "GHXV-289" "GHXV-288" "GHXV-287" "GHXV-286" "GHXV-285" "GHXV-283"
aKO
"GHXV-282"

$TTHE580327
$TTHE580327$`TUHGH-473`
epsilonKO betaKO gammaKO alphaKO deltaKO bKO cKO
"GHGH-931" "GHGH-930" "GHGH-929" "GHGH-928" "GHGH-927" "GHGH-926" "GHGH-925"
aKO
"GHGH-924"

$`TTHE698948-WGS`
$`TTHE698948-WGS`$`TUSYM-435`
epsilonKO betaKO gammaKO alphaKO deltaKO bKO cKO
"GSYM-841" "GSYM-840" "GSYM-839" "GSYM-838" "GSYM-837" "GSYM-836" "GSYM-835"
aKO
"GSYM-834"

$LINT363253
$LINT363253$`TUH6E-374`
epsilonKO betaKO gammaKO alphaKO deltaKO bKO2 bKO1
"GH6E-415" "GH6E-414" "GH6E-413" "GH6E-412" "GH6E-411" "GH6E-410" "GH6E-409"

$LINT363253$`TUH6E-802`
cKO
"GH6E-1101"

$LINT363253$`TUH6E-801`
aKO
"GH6E-1100"

$TTER525904
$TTER525904$`TUHMJ-31`
aKO cKO bKO deltaKO alphaKO gammaKO betaKO epsilonKO
"GHMJ-69" "GHMJ-68" "GHMJ-67" "GHMJ-66" "GHMJ-65" "GHMJ-64" "GHMJ-63" "GHMJ-62"

$TTUR377629
$TTUR377629$`TUHSU-2376`
aKO cKO bKO deltaKO alphaKO gammaKO
"GHSU-4293" "GHSU-4292" "GHSU-4291" "GHSU-4290" "GHSU-4289" "GHSU-4288"
betaKO epsilonKO
"GHSU-4287" "GHSU-4286"

$TVIO765911
$TVIO765911$`TULMH-1446`
cKO bKO deltaKO alphaKO gammaKO betaKO
"GLMH-2586" "GLMH-2585" "GLMH-2584" "GLMH-2583" "GLMH-2582" "GLMH-2581"
epsilonKO
"GLMH-2580"

$TVIO765911$`TULMH-1447`
aKO
"GLMH-2587"

$TWHI203267
$TWHI203267$`TUJDK-177`
aKO cKO bKO deltaKO alphaKO gammaKO betaKO
"GJDK-461" "GJDK-460" "GJDK-459" "GJDK-458" "GJDK-457" "GJDK-456" "GJDK-455"
epsilonKO
"GJDK-454"

$TWIE697303
$TWIE697303$`TUH3A-367`
epsilonKO betaKO gammaKO alphaKO deltaKO bKO cKO
"GH3A-778" "GH3A-777" "GH3A-776" "GH3A-775" "GH3A-774" "GH3A-773" "GH3A-772"
aKO
"GH3A-771"

$`LPLA220668-WGS`
$`LPLA220668-WGS`$`TUSPK-1093`
aKO cKO bKO deltaKO alphaKO gammaKO
"GSPK-2042" "GSPK-2041" "GSPK-2040" "GSPK-2039" "GSPK-2038" "GSPK-2037"
betaKO epsilonKO
"GSPK-2036" "GSPK-2035"

```
$`TWHI218496-WGS`
$`TWHI218496-WGS`$`TUSYL-148`
 epsilonKO     betaKO    gammaKO    alphaKO    deltaKO        bKO        cKO
"GSYL-341" "GSYL-340" "GSYL-339" "GSYL-338" "GSYL-337" "GSYL-336" "GSYL-335"
       aKO
"GSYL-334"


$TXYL858215
$TXYL858215$`TUHCH-904`
        aKO         cKO         bKO     deltaKO     alphaKO     gammaKO
"GHCH-1902" "GHCH-1901" "GHCH-1900" "GHCH-1899" "GHCH-1898" "GHCH-1897"
     betaKO   epsilonKO
"GHCH-1896" "GHCH-1895"


$TYEL289376
$TYEL289376$`TUH9L-75`
 epsilonKO     betaKO    gammaKO    alphaKO    deltaKO       bKO2       bKO1
"GH9L-242" "GH9L-241" "GH9L-240" "GH9L-239" "GH9L-238" "GH9L-237" "GH9L-236"

$TYEL289376$`TUH9L-479`
        aKO         cKO
"GH9L-1520" "GH9L-1519"


$UPAR505682
$UPAR505682$`TUHAZ-22`
  betaKO1  alphaKO1
"GHAZ-53" "GHAZ-52"

$UPAR505682$`TUHAZ-64`
       aKO        cKO        bKO   deltaKO2   deltaKO1   alphaKO2    gammaKO
"GHAZ-145" "GHAZ-144" "GHAZ-143" "GHAZ-142" "GHAZ-141" "GHAZ-140" "GHAZ-138"
   betaKO2  epsilonKO
"GHAZ-137" "GHAZ-136"


$LPNE272624
$LPNE272624$`TUHDI-551`
    betaKO1  epsilonKO1        aKO1        cKO1        bKO1    alphaKO1
"GHDI-1053" "GHDI-1052" "GHDI-1050" "GHDI-1049" "GHDI-1048" "GHDI-1047"
   gammaKO1
"GHDI-1046"

$LPNE272624$`TUHDI-1647`
       aKO2        cKO2        bKO2     deltaKO    alphaKO2    gammaKO2
"GHDI-2986" "GHDI-2985" "GHDI-2984" "GHDI-2983" "GHDI-2982" "GHDI-2981"
    betaKO2  epsilonKO2
"GHDI-2980" "GHDI-2979"


$UURE565575
```

```
$UURE565575$`TUBZS-28`
  betaKO1  alphaKO1
"GBZS-59" "GBZS-58"

$UURE565575$`TUBZS-74`
      bKO   deltaKO2   deltaKO1   alphaKO2    gammaKO    betaKO2  epsilonKO
"GBZS-150" "GBZS-149" "GBZS-148" "GBZS-147" "GBZS-145" "GBZS-144" "GBZS-143"

$UURE565575$`TUBZS-75`
      aKO        cKO
"GBZS-153" "GBZS-152"


$UPAR273119
$UPAR273119$`TUHVP-27`
  betaKO1  alphaKO1
"GHVP-55" "GHVP-54"

$UPAR273119$`TUHVP-64`
      aKO        cKO        bKO   deltaKO2   deltaKO1   alphaKO2    gammaKO
"GHVP-143" "GHVP-142" "GHVP-141" "GHVP-140" "GHVP-139" "GHVP-138" "GHVP-136"
   betaKO2  epsilonKO
"GHVP-135" "GHVP-134"


$`VALG1219076-WGS`
$`VALG1219076-WGS`$`TUSYO-2189`
       aKO         cKO         bKO     deltaKO     alphaKO     gammaKO
"GSYO-2253" "GSYO-2252" "GSYO-2251" "GSYO-2250" "GSYO-2249" "GSYO-2248"
    betaKO   epsilonKO
"GSYO-2247" "GSYO-2246"


$LANG882102
$LANG882102$`TUIWG-113`
 epsilonKO     betaKO    gammaKO    alphaKO    deltaKO       bKO       cKO
"GIWG-243" "GIWG-242" "GIWG-240" "GIWG-239" "GIWG-238" "GIWG-237" "GIWG-236"
      aKO
"GIWG-235"


$VPAR543728
$VPAR543728$`TUHLL-2257`
       aKO         cKO         bKO     deltaKO     alphaKO     gammaKO
"GHLL-4932" "GHLL-4931" "GHLL-4930" "GHLL-4929" "GHLL-4928" "GHLL-4927"
    betaKO   epsilonKO
"GHLL-4925" "GHLL-4924"


$VCHO914149
$VCHO914149$`TUHG2-1272`
  epsilonKO      betaKO     gammaKO     alphaKO     deltaKO         bKO
"GHG2-2422" "GHG2-2421" "GHG2-2420" "GHG2-2419" "GHG2-2418" "GHG2-2417"
       cKO         aKO
```

"GHG2-2416" "GHG2-2415"

$`LPLA767468-WGS`
$`LPLA767468-WGS`$`TUSP4-1240`
aKO cKO bKO deltaKO alphaKO gammaKO
"GSP4-1998" "GSP4-1997" "GSP4-1996" "GSP4-1995" "GSP4-1994" "GSP4-1993"
betaKO epsilonKO
"GSP4-1992" "GSP4-1991"

$VCHO1134456
$VCHO1134456$`TULMN-2`
aKO cKO bKO deltaKO alphaKO gammaKO
"GLMN-2749" "GLMN-2748" "GLMN-2747" "GLMN-2746" "GLMN-2745" "GLMN-2744"
betaKO epsilonKO
"GLMN-2743" "GLMN-2742"

$VCHO593588
$VCHO593588$`TUI2R-847`
aKO cKO bKO deltaKO alphaKO gammaKO betaKO
"GI2R-497" "GI2R-496" "GI2R-495" "GI2R-494" "GI2R-493" "GI2R-492" "GI2R-491"
epsilonKO
"GI2R-490"

$VCHO935297
$VCHO935297$`TULMO-501`
aKO cKO bKO deltaKO alphaKO gammaKO
"GLMO-2574" "GLMO-2573" "GLMO-2572" "GLMO-2571" "GLMO-2570" "GLMO-2569"
betaKO epsilonKO
"GLMO-2568" "GLMO-2567"

$GFOR411154
$GFOR411154$`TUI79-1677`
aKO cKO bKO deltaKO alphaKO gammaKO
"GI79-3270" "GI79-3269" "GI79-3268" "GI79-3267" "GI79-3266" "GI79-3265"

$GFOR411154$`TUI79-1824`
epsilonKO betaKO
"GI79-3549" "GI79-3548"

$VCHO579112
$VCHO579112$`TUJAW-2`
aKO cKO bKO deltaKO alphaKO gammaKO
"GJAW-2779" "GJAW-2778" "GJAW-2777" "GJAW-2776" "GJAW-2775" "GJAW-2774"
betaKO epsilonKO
"GJAW-2773" "GJAW-2772"

$VCHO345073

```
$VCHO345073$`TUI4W-1955`
  epsilonKO      betaKO     gammaKO     alphaKO     deltaKO         bKO
"GI4W-2566" "GI4W-2565" "GI4W-2564" "GI4W-2563" "GI4W-2562" "GI4W-2561"
        cKO         aKO
"GI4W-2560" "GI4W-2559"


$VEIS391735
$VEIS391735$`TUHY5-259`
 epsilonKO     betaKO    gammaKO    alphaKO    deltaKO        bKO        cKO
"GHY5-482" "GHY5-481" "GHY5-480" "GHY5-479" "GHY5-478" "GHY5-477" "GHY5-476"
       aKO
"GHY5-475"


$BCEN406425
$BCEN406425$`TUHD9-62`
epsilonKO1     betaKO   gammaKO1    alphaKO    deltaKO        bKO        cKO
"GHD9-127" "GHD9-126" "GHD9-125" "GHD9-124" "GHD9-123" "GHD9-122" "GHD9-121"
       aKO
"GHD9-120"

$BCEN406425$`TUHD9-3747`
   gammaKO2  epsilonKO2
"GHD9-5496" "GHD9-5495"


$BCEN331271
$BCEN331271$`TUHKX-1730`
        aKO         cKO         bKO     deltaKO     alphaKO    gammaKO1
"GHKX-3024" "GHKX-3023" "GHKX-3022" "GHKX-3021" "GHKX-3020" "GHKX-3019"
     betaKO  epsilonKO1
"GHKX-3018" "GHKX-3017"

$BCEN331271$`TUHKX-2034`
   gammaKO2  epsilonKO2
"GHKX-3568" "GHKX-3567"


$BCEL649639
$BCEL649639$`TUHTT-2507`
        aKO         cKO         bKO     deltaKO     alphaKO     gammaKO
"GHTT-4184" "GHTT-4183" "GHTT-4182" "GHTT-4181" "GHTT-4180" "GHTT-4179"
     betaKO   epsilonKO
"GHTT-4178" "GHTT-4177"


$LPLA889932
$LPLA889932$`TUHWQ-1185`
        aKO         cKO         bKO     deltaKO     alphaKO     gammaKO
"GHWQ-2057" "GHWQ-2056" "GHWQ-2055" "GHWQ-2054" "GHWQ-2053" "GHWQ-2052"
     betaKO   epsilonKO
"GHWQ-2051" "GHWQ-2050"
```

```
$BPUN1075399
$BPUN1075399$`TUJY7-207`
       aKO        cKO        bKO    deltaKO    alphaKO    gammaKO
"GJY7-531" "GJY7-530" "GJY7-529" "GJY7-528" "GJY7-527" "GJY7-526"

$BPUN1075399$`TUJY7-27`
epsilonKO    betaKO
"GJY7-66" "GJY7-65"


$VSP1116375
$VSP1116375$`TUJV8-2`
       aKO1        cKO1        bKO1     deltaKO    alphaKO1    gammaKO1
"GJV8-3207" "GJV8-3206" "GJV8-3205" "GJV8-3204" "GJV8-3203" "GJV8-3202"
    betaKO1  epsilonKO1
"GJV8-3201" "GJV8-3200"

$VSP1116375$`TUJV8-2433`
    betaKO2  epsilonKO2        aKO2        cKO2        bKO2    alphaKO2
"GJV8-4396" "GJV8-4395" "GJV8-4393" "GJV8-4392" "GJV8-4391" "GJV8-4390"
   gammaKO2
"GJV8-4389"


$VSP150340
$VSP150340$`TUJJG-235`
 epsilonKO     betaKO    gammaKO    alphaKO    deltaKO        bKO        cKO
"GJJG-434" "GJJG-433" "GJJG-432" "GJJG-431" "GJJG-430" "GJJG-429" "GJJG-428"
       aKO
"GJJG-427"


$`AMED713604-WGS`
$`AMED713604-WGS`$`TUSF8-3905`
    alphaKO     gammaKO      betaKO
"GSF8-7712" "GSF8-7711" "GSF8-7710"

$`AMED713604-WGS`$`TUSF8-3906`
       cKO2         bKO     deltaKO
"GSF8-7715" "GSF8-7714" "GSF8-7713"

$`AMED713604-WGS`$`TUSF8-3904`
  epsilonKO
"GSF8-7709"

$`AMED713604-WGS`$`TUSF8-2051`
       cKO1
"GSF8-4028"

$`AMED713604-WGS`$`TUSF8-3907`
        aKO
"GSF8-7716"
```

```
$AFIS312309
$AFIS312309$`TUIWP-26`
       aKO         cKO         bKO     deltaKO     alphaKO     gammaKO 
"GIWP-2734" "GIWP-2733" "GIWP-2732" "GIWP-2731" "GIWP-2730" "GIWP-2729" 
    betaKO   epsilonKO 
"GIWP-2728" "GIWP-2727" 



$`VFIS388396-WGS`
$`VFIS388396-WGS`$`TUSYR-2257`
       aKO         cKO         bKO     deltaKO     alphaKO     gammaKO 
"GSYR-2707" "GSYR-2706" "GSYR-2705" "GSYR-2704" "GSYR-2703" "GSYR-2702" 
    betaKO   epsilonKO 
"GSYR-2701" "GSYR-2700" 



$VFUR903510
$VFUR903510$`TUHFS-209`
epsilonKO1    betaKO1   gammaKO1   alphaKO1    deltaKO       bKO1       cKO1 
"GHFS-362" "GHFS-361" "GHFS-360" "GHFS-359" "GHFS-358" "GHFS-357" "GHFS-356" 
      aKO1 
"GHFS-355" 

$VFUR903510$`TUHFS-2436`
      aKO2        cKO2        bKO2    alphaKO2    gammaKO2     betaKO2 
"GHFS-4387" "GHFS-4386" "GHFS-4385" "GHFS-4383" "GHFS-4382" "GHFS-4381" 
 epsilonKO2 
"GHFS-4380" 



$VMAR263358
$VMAR263358$`TUI1P-2826`
       aKO         cKO         bKO     deltaKO     alphaKO     gammaKO 
"GI1P-5010" "GI1P-5009" "GI1P-5008" "GI1P-5007" "GI1P-5006" "GI1P-5005" 
    betaKO 
"GI1P-5004" 

$VMAR263358$`TUI1P-2824`
  epsilonKO 
"GI1P-5002" 



$`VNIG28173-WGS`
$`VNIG28173-WGS`$`TUSYS-3092`
       aKO         cKO         bKO     deltaKO     alphaKO     gammaKO 
"GSYS-3737" "GSYS-3736" "GSYS-3735" "GSYS-3734" "GSYS-3733" "GSYS-3732" 
    betaKO   epsilonKO 
"GSYS-3731" "GSYS-3730" 



$CVES412965
$CVES412965$`TUHZZ-397`
      aKO        cKO        bKO    deltaKO    alphaKO    gammaKO     betaKO 
```

"GHZZ-951" "GHZZ-950" "GHZZ-949" "GHZZ-948" "GHZZ-947" "GHZZ-946" "GHZZ-945"
epsilonKO
"GHZZ-944"

$VPAR223926
$VPAR223926$`TUHK4-2`
aKO cKO bKO deltaKO alphaKO gammaKO
"GHK4-3218" "GHK4-3217" "GHK4-3216" "GHK4-3215" "GHK4-3214" "GHK4-3213"
betaKO epsilonKO
"GHK4-3212" "GHK4-3211"

$`LPLA1284663-WGS`
$`LPLA1284663-WGS`$`TUSP6-1392`
aKO cKO bKO deltaKO alphaKO gammaKO
"GSP6-2404" "GSP6-2403" "GSP6-2402" "GSP6-2401" "GSP6-2400" "GSP6-2399"
betaKO epsilonKO
"GSP6-2398" "GSP6-2397"

$`VPAR1211705-WGS`
$`VPAR1211705-WGS`$`TUSYT-2636`
aKO cKO bKO deltaKO alphaKO gammaKO
"GSYT-3067" "GSYT-3066" "GSYT-3065" "GSYT-3064" "GSYT-3063" "GSYT-3062"
betaKO epsilonKO
"GSYT-3061" "GSYT-3060"

$`VPAR1246301-WGS`
$`VPAR1246301-WGS`$`TUSYN-2280`
aKO cKO bKO deltaKO alphaKO gammaKO
"GSYN-5058" "GSYN-5057" "GSYN-5056" "GSYN-5055" "GSYN-5054" "GSYN-5053"
betaKO epsilonKO
"GSYN-5052" "GSYN-5051"

$AMAR329726
$AMAR329726$`TUCZJ-1894`
gammaKO1 alphaKO1 deltaKO bKO2 bKO1 cKO1 aKO1
"GCZJ-888" "GCZJ-887" "GCZJ-886" "GCZJ-885" "GCZJ-884" "GCZJ-883" "GCZJ-882"

$AMAR329726$`TUCZJ-821`
gammaKO2 alphaKO2 bKO3 cKO2 aKO2
"GCZJ-7748" "GCZJ-7747" "GCZJ-7746" "GCZJ-7745" "GCZJ-7744"

$AMAR329726$`TUCZJ-4760`
betaKO1 epsilonKO1
"GCZJ-5315" "GCZJ-5314"

$AMAR329726$`TUCZJ-820`
epsilonKO2 betaKO2
"GCZJ-7740" "GCZJ-7739"

```
$VPAR595537
$VPAR595537$`TUHGJ-2634`
       aKO        cKO        bKO     deltaKO     alphaKO     gammaKO 
"GHGJ-5578" "GHGJ-5577" "GHGJ-5576" "GHGJ-5575" "GHGJ-5574" "GHGJ-5573" 
    betaKO   epsilonKO 
"GHGJ-5572" "GHGJ-5571" 


$VPAR479436
$VPAR479436$`TUHOS-765`
       aKO        cKO        bKO     deltaKO     alphaKO     gammaKO 
"GHOS-1600" "GHOS-1599" "GHOS-1598" "GHOS-1597" "GHOS-1596" "GHOS-1595" 
    betaKO   epsilonKO 
"GHOS-1594" "GHOS-1593" 


$`ASAL316275-WGS`
$`ASAL316275-WGS`$`TUSFB-2387`
       aKO        cKO        bKO     deltaKO     alphaKO     gammaKO 
"GSFB-3184" "GSFB-3183" "GSFB-3182" "GSFB-3181" "GSFB-3180" "GSFB-3179" 
    betaKO   epsilonKO 
"GSFB-3178" "GSFB-3177" 


$VVUL914127
$VVUL914127$`TUJJH-9`
epsilonKO    betaKO   gammaKO   alphaKO   deltaKO       bKO       cKO       aKO 
"GJJH-13" "GJJH-12" "GJJH-11" "GJJH-10"  "GJJH-9"  "GJJH-8"  "GJJH-7"  "GJJH-6" 


$VVUL216895
$VVUL216895$`TUIYM-488`
 epsilonKO     betaKO    gammaKO    alphaKO    deltaKO        bKO        cKO 
"GIYM-936" "GIYM-935" "GIYM-934" "GIYM-933" "GIYM-932" "GIYM-931" "GIYM-930" 
       aKO 
"GIYM-929" 


$VVUL196600
$VVUL196600$`TUJ9W-31`
       aKO        cKO        bKO     deltaKO     alphaKO     gammaKO 
"GJ9W-3382" "GJ9W-3381" "GJ9W-3380" "GJ9W-3379" "GJ9W-3378" "GJ9W-3377" 
    betaKO   epsilonKO 
"GJ9W-3376" "GJ9W-3375" 


$WEND292805
$WEND292805$`TUH18-218`
   deltaKO    alphaKO 
"GH18-360" "GH18-359" 

$WEND292805$`TUH18-487`
    betaKO  epsilonKO 
```

"GH18-798" "GH18-797"

$WEND292805$`TUH18-502`
gammaKO
"GH18-819"

$WEND292805$`TUH18-306`
aKO cKO bKO2 bKO1
"GH18-525" "GH18-524" "GH18-523" "GH18-522"

$`WGLO36870-WGS`
$`WGLO36870-WGS`$`TUSYV-2`
epsilonKO betaKO gammaKO alphaKO deltaKO bKO cKO aKO
"GSYV-9" "GSYV-8" "GSYV-7" "GSYV-6" "GSYV-5" "GSYV-4" "GSYV-3" "GSYV-2"

$`LPLA1327988-WGS`
$`LPLA1327988-WGS`$`TUSP3-1211`
bKO deltaKO alphaKO gammaKO betaKO epsilonKO
"GSP3-1881" "GSP3-1880" "GSP3-1879" "GSP3-1878" "GSP3-1877" "GSP3-1876"

$`LPLA1327988-WGS`$`TUSP3-1212`
aKO cKO
"GSP3-1883" "GSP3-1882"

$WCHO716544
$WCHO716544$`TUHGA-538`
epsilonKO betaKO gammaKO alphaKO deltaKO bKO
"GHGA-1129" "GHGA-1128" "GHGA-1127" "GHGA-1126" "GHGA-1125" "GHGA-1124"
cKO aKO
"GHGA-1123" "GHGA-1122"

$HPYL866344
$HPYL866344$`TULEC-483`
bKO2 bKO1 deltaKO alphaKO gammaKO betaKO
"GLEC-1109" "GLEC-1108" "GLEC-1107" "GLEC-1106" "GLEC-1105" "GLEC-1104"
epsilonKO
"GLEC-1103"

$HPYL866344$`TULEC-523`
cKO
"GLEC-1186"

$HPYL866344$`TULEC-224`
aKO
"GLEC-545"

$`WEND1236908-WGS`
$`WEND1236908-WGS`$`TUSZ5-489`
alphaKO deltaKO

```
"GSZ5-872" "GSZ5-871"

$`WEND1236908-WGS`$`TUSZ5-486`
 epsilonKO     betaKO
"GSZ5-836" "GSZ5-835"

$`WEND1236908-WGS`$`TUSZ5-381`
   gammaKO
"GSZ5-649"

$`WEND1236908-WGS`$`TUSZ5-186`
       aKO        cKO       bKO2       bKO1
"GSZ5-324" "GSZ5-323" "GSZ5-322" "GSZ5-321"


$`WEND1236909-WGS`
$`WEND1236909-WGS`$`TUSYX-339`
   deltaKO    alphaKO
"GSYX-567" "GSYX-566"

$`WEND1236909-WGS`$`TUSYX-99`
 epsilonKO     betaKO
"GSYX-169" "GSYX-168"

$`WEND1236909-WGS`$`TUSYX-615`
    gammaKO
"GSYX-1064"

$`WEND1236909-WGS`$`TUSYX-240`
       aKO        cKO       bKO2       bKO1
"GSYX-410" "GSYX-409" "GSYX-408" "GSYX-407"


$WGLO1142511
$WGLO1142511$`TUJU3-2`
 alphaKO  deltaKO      bKO      cKO      aKO
"GJU3-6" "GJU3-5" "GJU3-4" "GJU3-3" "GJU3-2"

$WGLO1142511$`TUJU3-3`
epsilonKO    betaKO   gammaKO
 "GJU3-9"  "GJU3-8"  "GJU3-7"


$WKOR1045854
$WKOR1045854$`TUH3T-317`
       aKO        cKO        bKO    deltaKO    alphaKO    gammaKO     betaKO
"GH3T-593" "GH3T-592" "GH3T-591" "GH3T-590" "GH3T-589" "GH3T-588" "GH3T-587"
 epsilonKO
"GH3T-586"


$WEND163164
$WEND163164$`TUJ8W-376`
   deltaKO    alphaKO
```

"GJ8W-641" "GJ8W-640"

$WEND163164$`TUJ8W-117`
 epsilonKO   betaKO
"GJ8W-198" "GJ8W-197"

$WEND163164$`TUJ8W-693`
    gammaKO
"GJ8W-1220"

$WEND163164$`TUJ8W-257`
      bKO2   bKO1   cKO   aKO
"GJ8W-424" "GJ8W-423" "GJ8W-422" "GJ8W-421"

$WEND100901
$WEND100901$`TULMP-412`
   deltaKO   alphaKO
"GLMP-688" "GLMP-687"

$WEND100901$`TULMP-44`
   betaKO epsilonKO
"GLMP-74" "GLMP-73"

$WEND100901$`TULMP-77`
   gammaKO
"GLMP-143"

$WEND100901$`TULMP-28`
     bKO2   bKO1   cKO   aKO
"GLMP-44" "GLMP-43" "GLMP-42" "GLMP-41"

$WEND570417
$WEND570417$`TUHSW-681`
    alphaKO   deltaKO
"GHSW-1227" "GHSW-1226"

$WEND570417$`TUHSW-676`
  epsilonKO   betaKO
"GHSW-1190" "GHSW-1189"

$WEND570417$`TUHSW-622`
    gammaKO
"GHSW-1102"

$WEND570417$`TUHSW-349`
       aKO   cKO   bKO2   bKO1
"GHSW-631" "GHSW-630" "GHSW-629" "GHSW-628"

$WSP66084
$WSP66084$`TUHAN-288`
   deltaKO   alphaKO

"GHAN-457" "GHAN-456"

$WSP66084$`TUHAN-107`
epsilonKO betaKO
"GHAN-177" "GHAN-176"

$WSP66084$`TUHAN-653`
gammaKO
"GHAN-1139"

$WSP66084$`TUHAN-183`
aKO cKO bKO2 bKO1
"GHAN-295" "GHAN-294" "GHAN-293" "GHAN-292"

$WVIR865938
$WVIR865938$`TUHXX-613`
gammaKO alphaKO deltaKO bKO cKO aKO
"GHXX-1246" "GHXX-1245" "GHXX-1244" "GHXX-1243" "GHXX-1242" "GHXX-1241"

$WVIR865938$`TUHXX-439`
betaKO
"GHXX-909"

$WVIR865938$`TUHXX-440`
epsilonKO
"GHXX-910"

$LRHA1088720
$LRHA1088720$`TULFB-650`
gammaKO alphaKO deltaKO bKO cKO aKO
"GLFB-1152" "GLFB-1151" "GLFB-1150" "GLFB-1149" "GLFB-1148" "GLFB-1147"

$LRHA1088720$`TULFB-651`
epsilonKO betaKO
"GLFB-1155" "GLFB-1154"

$HELO768066
$HELO768066$`TUJEE-1853`
aKO cKO bKO deltaKO alphaKO gammaKO
"GJEE-3533" "GJEE-3532" "GJEE-3531" "GJEE-3530" "GJEE-3529" "GJEE-3528"
betaKO epsilonKO
"GJEE-3527" "GJEE-3526"

$XAXO190486
$XAXO190486$`TUH55-2214`
aKO cKO bKO deltaKO alphaKO gammaKO
"GH55-3655" "GH55-3654" "GH55-3653" "GH55-3652" "GH55-3651" "GH55-3650"
betaKO epsilonKO
"GH55-3649" "GH55-3648"

```
$XALB380358
$XALB380358$`TULMQ-1691`
       aKO         cKO         bKO     deltaKO     alphaKO     gammaKO 
"GLMQ-2904" "GLMQ-2903" "GLMQ-2902" "GLMQ-2901" "GLMQ-2900" "GLMQ-2899" 
    betaKO   epsilonKO 
"GLMQ-2898" "GLMQ-2897" 


$`XAXO1304892-WGS`
$`XAXO1304892-WGS`$`TUSYZ-2144`
       aKO         cKO         bKO     deltaKO     alphaKO     gammaKO 
"GSYZ-3633" "GSYZ-3632" "GSYZ-3631" "GSYZ-3630" "GSYZ-3629" "GSYZ-3628" 
    betaKO   epsilonKO 
"GSYZ-3627" "GSYZ-3626" 


$XAUT78245
$XAUT78245$`TUHS6-1317`
    betaKO     gammaKO     alphaKO     deltaKO 
"GHS6-2102" "GHS6-2101" "GHS6-2100" "GHS6-2099" 

$XAUT78245$`TUHS6-1318`
  epsilonKO 
"GHS6-2104" 

$XAUT78245$`TUHS6-1254`
       cKO 
"GHS6-2000" 

$XAUT78245$`TUHS6-1255`
       aKO 
"GHS6-2001" 

$XAUT78245$`TUHS6-1252`
      bKO1 
"GHS6-1998" 

$XAUT78245$`TUHS6-1253`
      bKO2 
"GHS6-1999" 


$XALF981368
$XALF981368$`TUH9H-2060`
       aKO         cKO         bKO     deltaKO     alphaKO     gammaKO 
"GH9H-3549" "GH9H-3548" "GH9H-3547" "GH9H-3546" "GH9H-3545" "GH9H-3544" 
    betaKO   epsilonKO 
"GH9H-3543" "GH9H-3542" 


$XBOV406818
$XBOV406818$`TUHLH-16`
      aKO       cKO       bKO   deltaKO   alphaKO   gammaKO    betaKO epsilonKO 
```

"GHLH-28" "GHLH-27" "GHLH-26" "GHLH-25" "GHLH-24" "GHLH-23" "GHLH-22" "GHLH-21"

$XCAM509169
$XCAM509169$`TUHW4-2135`
aKO cKO bKO deltaKO alphaKO gammaKO
"GHW4-3887" "GHW4-3886" "GHW4-3885" "GHW4-3884" "GHW4-3883" "GHW4-3882"
betaKO epsilonKO
"GHW4-3881" "GHW4-3880"

$BCER361100
$BCER361100$`TUJ7M-3256`
aKO cKO bKO deltaKO alphaKO gammaKO
"GJ7M-5138" "GJ7M-5137" "GJ7M-5136" "GJ7M-5135" "GJ7M-5134" "GJ7M-5133"
betaKO epsilonKO
"GJ7M-5132" "GJ7M-5131"

$BCER405534
$BCER405534$`TUHXM-3395`
aKO cKO bKO deltaKO alphaKO
"GHXM-5413" "GHXM-5412" "GHXM-5411" "GHXM-5410" "GHXM-5409"

$BCER405534$`TUHXM-3394`
gammaKO betaKO epsilonKO
"GHXM-5407" "GHXM-5406" "GHXM-5405"

$BCAN483179
$BCAN483179$`TUJ7I-1038`
deltaKO alphaKO gammaKO betaKO epsilonKO
"GJ7I-1819" "GJ7I-1818" "GJ7I-1817" "GJ7I-1816" "GJ7I-1815"

$BCAN483179$`TUJ7I-222`
bKO2 bKO1 cKO aKO
"GJ7I-387" "GJ7I-386" "GJ7I-385" "GJ7I-384"

$HPYL102608
$HPYL102608$`TULEE-467`
bKO2 bKO1 deltaKO alphaKO gammaKO betaKO
"GLEE-1101" "GLEE-1100" "GLEE-1099" "GLEE-1098" "GLEE-1097" "GLEE-1096"
epsilonKO
"GLEE-1095"

$HPYL102608$`TULEE-507`
cKO
"GLEE-1180"

$HPYL102608$`TULEE-354`
aKO
"GLEE-817"

```
$`LRHA1318634-WGS`
$`LRHA1318634-WGS`$`TUSPC-680`
   gammaKO     alphaKO     deltaKO         bKO         cKO         aKO 
"GSPC-1228" "GSPC-1227" "GSPC-1226" "GSPC-1225" "GSPC-1224" "GSPC-1223" 

$`LRHA1318634-WGS`$`TUSPC-681`
  epsilonKO      betaKO 
"GSPC-1231" "GSPC-1230" 


$BCEP1009846
$BCEP1009846$`TUL9Y-72`
  gammaKO1    alphaKO    deltaKO        bKO        cKO        aKO 
"GL9Y-100"  "GL9Y-99"  "GL9Y-98"  "GL9Y-97"  "GL9Y-96"  "GL9Y-95" 

$BCEP1009846$`TUL9Y-73`
    betaKO 
"GL9Y-101" 

$BCEP1009846$`TUL9Y-2080`
   gammaKO2  epsilonKO2 
"GL9Y-3606" "GL9Y-3605" 

$BCEP1009846$`TUL9Y-74`
epsilonKO1 
"GL9Y-102" 


$XCAM314565
$XCAM314565$`TUCQG-2150`
        aKO         cKO         bKO     deltaKO     alphaKO     gammaKO 
"GCQG-3709" "GCQG-3708" "GCQG-3707" "GCQG-3706" "GCQG-3705" "GCQG-3704" 
     betaKO   epsilonKO 
"GCQG-3703" "GCQG-3702" 


$XCAM190485
$XCAM190485$`TUIXZ-350`
 epsilonKO     betaKO    gammaKO    alphaKO    deltaKO        bKO        cKO 
"GIXZ-555" "GIXZ-554" "GIXZ-553" "GIXZ-552" "GIXZ-551" "GIXZ-550" "GIXZ-549" 
       aKO 
"GIXZ-548" 


$XCEL446471
$XCEL446471$`TUHA2-1276`
        aKO         cKO         bKO     deltaKO     alphaKO     gammaKO 
"GHA2-2503" "GHA2-2502" "GHA2-2501" "GHA2-2500" "GHA2-2499" "GHA2-2498" 
     betaKO   epsilonKO 
"GHA2-2497" "GHA2-2496" 


$`XCIT1137651-WGS`
```

$`XCIT1137651-WGS`$`TUSZ0-2538`
aKO cKO bKO deltaKO alphaKO gammaKO
"GSZ0-4347" "GSZ0-4346" "GSZ0-4345" "GSZ0-4344" "GSZ0-4343" "GSZ0-4342"
betaKO epsilonKO
"GSZ0-4340" "GSZ0-4339"

$XCAM990315
$XCAM990315$`TULMR-419`
epsilonKO betaKO gammaKO alphaKO deltaKO bKO cKO
"GLMR-706" "GLMR-705" "GLMR-704" "GLMR-703" "GLMR-702" "GLMR-701" "GLMR-700"
aKO
"GLMR-699"

$XCAM316273
$XCAM316273$`TUJF8-2336`
aKO cKO bKO deltaKO alphaKO gammaKO
"GJF8-3876" "GJF8-3875" "GJF8-3874" "GJF8-3873" "GJF8-3872" "GJF8-3871"
betaKO epsilonKO
"GJF8-3870" "GJF8-3869"

$`XFAS160492-WGS`
$`XFAS160492-WGS`$`TUSZ1-685`
aKO cKO bKO deltaKO alphaKO gammaKO
"GSZ1-1177" "GSZ1-1176" "GSZ1-1175" "GSZ1-1174" "GSZ1-1173" "GSZ1-1172"
betaKO epsilonKO
"GSZ1-1171" "GSZ1-1170"

$XFAS788929
$XFAS788929$`TULMT-840`
aKO cKO bKO deltaKO alphaKO gammaKO
"GLMT-1442" "GLMT-1441" "GLMT-1440" "GLMT-1439" "GLMT-1438" "GLMT-1437"
betaKO epsilonKO
"GLMT-1436" "GLMT-1435"

$BSP288000
$BSP288000$`TUJBR-391`
epsilonKO1 betaKO1 gammaKO1 alphaKO deltaKO
"GJBR-395" "GJBR-394" "GJBR-393" "GJBR-392" "GJBR-391"

$BSP288000$`TUJBR-975`
betaKO2 epsilonKO2 aKO2 cKO2 bKO3
"GJBR-1381" "GJBR-1380" "GJBR-1378" "GJBR-1377" "GJBR-1376"

$BSP288000$`TUJBR-973`
gammaKO2
"GJBR-1374"

$BSP288000$`TUJBR-625`
aKO1 cKO1 bKO2 bKO1

```
"GJBR-812" "GJBR-811" "GJBR-810" "GJBR-809"


$HPYL866346
$HPYL866346$`TULEF-475`
      bKO2       bKO1     deltaKO     alphaKO     gammaKO      betaKO
"GLEF-1129" "GLEF-1128" "GLEF-1127" "GLEF-1126" "GLEF-1125" "GLEF-1124"
  epsilonKO
"GLEF-1123"

$HPYL866346$`TULEF-518`
       cKO
"GLEF-1211"

$HPYL866346$`TULEF-363`
      aKO
"GLEF-864"


$XFAS405440
$XFAS405440$`TUH0D-275`
      aKO       cKO       bKO    deltaKO    alphaKO
"GH0D-514" "GH0D-513" "GH0D-512" "GH0D-511" "GH0D-510"

$XFAS405440$`TUH0D-273`
    betaKO  epsilonKO
"GH0D-508" "GH0D-507"

$XFAS405440$`TUH0D-274`
   gammaKO
"GH0D-509"


$LBIF456481
$LBIF456481$`TUCM0-390`
 epsilonKO     betaKO    gammaKO    alphaKO    deltaKO       bKO       cKO
"GCM0-799" "GCM0-798" "GCM0-797" "GCM0-796" "GCM0-795" "GCM0-794" "GCM0-793"
      aKO
"GCM0-792"


$LREU557436
$LREU557436$`TUC7Y-264`
 epsilonKO     betaKO    gammaKO    alphaKO    deltaKO       bKO       cKO
"GC7Y-486" "GC7Y-485" "GC7Y-484" "GC7Y-483" "GC7Y-482" "GC7Y-481" "GC7Y-480"
      aKO
"GC7Y-479"


$XFAS405441
$XFAS405441$`TUJJI-254`
      aKO       cKO       bKO    deltaKO    alphaKO    gammaKO     betaKO
"GJJI-453" "GJJI-452" "GJJI-451" "GJJI-450" "GJJI-449" "GJJI-448" "GJJI-447"
 epsilonKO
```

"GJJI-446"

$XFAS183190
$XFAS183190$`TUIX4-240`
aKO cKO bKO deltaKO alphaKO gammaKO betaKO
"GIX4-434" "GIX4-433" "GIX4-432" "GIX4-431" "GIX4-430" "GIX4-429" "GIX4-428"
epsilonKO
"GIX4-427"

$XNEM406817
$XNEM406817$`TUHY4-120`
aKO cKO bKO deltaKO alphaKO gammaKO betaKO epsilonKO
"GHY4-31" "GHY4-30" "GHY4-29" "GHY4-28" "GHY4-27" "GHY4-26" "GHY4-25" "GHY4-24"

$XORY342109
$XORY342109$`TUIX9-411`
epsilonKO betaKO gammaKO alphaKO deltaKO bKO cKO
"GIX9-682" "GIX9-681" "GIX9-680" "GIX9-679" "GIX9-678" "GIX9-677" "GIX9-676"
aKO
"GIX9-675"

$XORY291331
$XORY291331$`TUJBV-403`
epsilonKO betaKO gammaKO alphaKO deltaKO bKO cKO
"GJBV-670" "GJBV-669" "GJBV-668" "GJBV-667" "GJBV-666" "GJBV-665" "GJBV-664"
aKO
"GJBV-663"

$XORY360094
$XORY360094$`TUI45-2641`
aKO cKO bKO deltaKO alphaKO gammaKO
"GI45-4564" "GI45-4563" "GI45-4562" "GI45-4561" "GI45-4560" "GI45-4559"
betaKO epsilonKO
"GI45-4558" "GI45-4557"

$XORY383407
$XORY383407$`TULMS-2131`
aKO cKO bKO deltaKO alphaKO gammaKO
"GLMS-3818" "GLMS-3817" "GLMS-3816" "GLMS-3815" "GLMS-3814" "GLMS-3813"
betaKO epsilonKO
"GLMS-3812" "GLMS-3811"

$HSP717785
$HSP717785$`TUJ7O-1318`
gammaKO1 alphaKO1 bKO1 cKO1 aKO1 epsilonKO1
"GJ7O-2446" "GJ7O-2445" "GJ7O-2444" "GJ7O-2443" "GJ7O-2442" "GJ7O-2439"
betaKO1

```
"GJ7O-2438"

$HSP717785$`TUJ7O-2574`
 epsilonKO2     betaKO2    gammaKO2    alphaKO2     deltaKO
"GJ7O-4738" "GJ7O-4736" "GJ7O-4734" "GJ7O-4733" "GJ7O-4732"

$HSP717785$`TUJ7O-2284`
      bKO3        bKO2        cKO2        aKO2
"GJ7O-4176" "GJ7O-4175" "GJ7O-4174" "GJ7O-4173"


$`YENT393305-WGS`
$`YENT393305-WGS`$`TUSZ2-2356`
       aKO         cKO         bKO     deltaKO     alphaKO     gammaKO
"GSZ2-4168" "GSZ2-4167" "GSZ2-4166" "GSZ2-4165" "GSZ2-4164" "GSZ2-4163"
    betaKO   epsilonKO
"GSZ2-4162" "GSZ2-4161"


$YENT994476
$YENT994476$`TUHRB-58`
       aKO         cKO         bKO     deltaKO     alphaKO     gammaKO
"GHRB-4111" "GHRB-4110" "GHRB-4109" "GHRB-4108" "GHRB-4107" "GHRB-4106"
    betaKO   epsilonKO
"GHRB-4105" "GHRB-4104"


$YENT930944
$YENT930944$`TULMU-1775`
  epsilonKO      betaKO     gammaKO     alphaKO     deltaKO         bKO
"GLMU-3025" "GLMU-3024" "GLMU-3023" "GLMU-3022" "GLMU-3021" "GLMU-3020"
       cKO         aKO
"GLMU-3019" "GLMU-3018"


$LREU557433
$LREU557433$`TUHNR-260`
 epsilonKO     betaKO    gammaKO    alphaKO    deltaKO        bKO        cKO
"GHNR-480" "GHNR-479" "GHNR-478" "GHNR-477" "GHNR-476" "GHNR-475" "GHNR-474"
      aKO
"GHNR-473"


$YPES360102
$YPES360102$`TUHZU-107`
       aKO         cKO         bKO     deltaKO     alphaKO     gammaKO
"GHZU-4268" "GHZU-4267" "GHZU-4266" "GHZU-4265" "GHZU-4264" "GHZU-4263"
    betaKO   epsilonKO
"GHZU-4262" "GHZU-4261"


$YPSE502801
$YPSE502801$`TUHIH-2440`
  epsilonKO      betaKO     gammaKO     alphaKO     deltaKO         bKO
```

```
"GHIH-4324" "GHIH-4323" "GHIH-4322" "GHIH-4321" "GHIH-4320" "GHIH-4319"
        cKO         aKO 
"GHIH-4318" "GHIH-4317" 


$YPES637382
$YPES637382$`TULMX-96`
        aKO         bKO     deltaKO     alphaKO     gammaKO      betaKO 
"GLMX-3717" "GLMX-3716" "GLMX-3715" "GLMX-3714" "GLMX-3713" "GLMX-3712" 
  epsilonKO 
"GLMX-3711" 

$YPES637382$noTU
cKO 
 NA 


$YPES214092
$YPES214092$`TUKDD-116`
        aKO         cKO         bKO     deltaKO     alphaKO     gammaKO 
"GKDD-4090" "GKDD-4089" "GKDD-4088" "GKDD-4087" "GKDD-4086" "GKDD-4085" 
     betaKO   epsilonKO 
"GKDD-4084" "GKDD-4083" 


$YPES349746
$YPES349746$`TUHPB-129`
        aKO         cKO         bKO     deltaKO     alphaKO     gammaKO 
"GHPB-4221" "GHPB-4220" "GHPB-4219" "GHPB-4218" "GHPB-4217" "GHPB-4216" 
     betaKO   epsilonKO 
"GHPB-4215" "GHPB-4214" 


$YPES547048
$YPES547048$`TULMW-2672`
        aKO         cKO         bKO     deltaKO     alphaKO     gammaKO 
"GLMW-4469" "GLMW-4468" "GLMW-4467" "GLMW-4466" "GLMW-4465" "GLMW-4464" 

$YPES547048$`TULMW-2671`
     betaKO   epsilonKO 
"GLMW-4462" "GLMW-4461" 


$HPYL866345
$HPYL866345$`TULED-104`
 epsilonKO     betaKO    gammaKO    alphaKO    deltaKO       bKO2       bKO1 
"GLED-271" "GLED-270" "GLED-269" "GLED-268" "GLED-267" "GLED-266" "GLED-265" 

$HPYL866345$`TULED-65`
       cKO 
"GLED-186" 

$HPYL866345$`TULED-217`
       aKO 
```

```
"GLED-531"



$YPSE349747
$YPSE349747$`TUH71-107`
       aKO        cKO        bKO     deltaKO     alphaKO     gammaKO 
"GH71-4290" "GH71-4289" "GH71-4288" "GH71-4287" "GH71-4286" "GH71-4285" 
     betaKO   epsilonKO 
"GH71-4284" "GH71-4283" 



$YPES187410
$YPES187410$`TUCPZ-5973`
       aKO        cKO        bKO     deltaKO     alphaKO     gammaKO 
"GCPZ-4497" "GCPZ-4496" "GCPZ-4495" "GCPZ-4494" "GCPZ-4493" "GCPZ-4492" 
     betaKO   epsilonKO 
"GCPZ-4491" "GCPZ-4490" 



$`YPES229193-WGS`
$`YPES229193-WGS`$`TUSZ3-2481`
       aKO        cKO        bKO     deltaKO     alphaKO     gammaKO 
"GSZ3-4117" "GSZ3-4116" "GSZ3-4115" "GSZ3-4114" "GSZ3-4113" "GSZ3-4112" 
     betaKO   epsilonKO 
"GSZ3-4111" "GSZ3-4110" 



$YPES377628
$YPES377628$`TUIXK-69`
       aKO        cKO        bKO     deltaKO     alphaKO     gammaKO 
"GIXK-4085" "GIXK-4084" "GIXK-4083" "GIXK-4082" "GIXK-4081" "GIXK-4080" 
     betaKO   epsilonKO 
"GIXK-4079" "GIXK-4078" 



$LRHA568703
$LRHA568703$`TUCGS-658`
    gammaKO     alphaKO     deltaKO         bKO         cKO         aKO 
"GCGS-1173" "GCGS-1172" "GCGS-1171" "GCGS-1170" "GCGS-1169" "GCGS-1168" 

$LRHA568703$`TUCGS-659`
  epsilonKO      betaKO 
"GCGS-1175" "GCGS-1174" 



$YPES386656
$YPES386656$`TUKD7-2436`
  epsilonKO      betaKO     gammaKO     alphaKO     deltaKO         bKO 
"GKD7-4018" "GKD7-4017" "GKD7-4016" "GKD7-4015" "GKD7-4014" "GKD7-4013" 
        cKO         aKO 
"GKD7-4012" "GKD7-4011" 



$YPSE273123
```

```
$YPSE273123$`TUI1M-75`
        aKO         cKO         bKO     deltaKO     alphaKO     gammaKO 
"GI1M-4093" "GI1M-4092" "GI1M-4091" "GI1M-4090" "GI1M-4089" "GI1M-4088" 
     betaKO   epsilonKO 
"GI1M-4087" "GI1M-4086" 


$YPES1035377
$YPES1035377$`TULMV-638`
  epsilonKO      betaKO     gammaKO     alphaKO     deltaKO         bKO 
"GLMV-1033" "GLMV-1032" "GLMV-1031" "GLMV-1030" "GLMV-1029" "GLMV-1028" 
        cKO         aKO 
"GLMV-1027" "GLMV-1026" 


$YPES637385
$YPES637385$`TULMY-98`
        aKO         bKO     deltaKO     alphaKO     gammaKO      betaKO 
"GLMY-3719" "GLMY-3718" "GLMY-3717" "GLMY-3716" "GLMY-3715" "GLMY-3714" 
  epsilonKO 
"GLMY-3713" 

$YPES637385$noTU
cKO 
 NA 


$YPSE502800
$YPSE502800$`TUH0W-2391`
  epsilonKO      betaKO     gammaKO     alphaKO     deltaKO         bKO 
"GH0W-4290" "GH0W-4289" "GH0W-4288" "GH0W-4287" "GH0W-4286" "GH0W-4285" 
        cKO         aKO 
"GH0W-4284" "GH0W-4283" 


$`AMED1221524-WGS`
$`AMED1221524-WGS`$`TUSF6-3907`
    alphaKO     gammaKO      betaKO 
"GSF6-7712" "GSF6-7711" "GSF6-7710" 

$`AMED1221524-WGS`$`TUSF6-3908`
       cKO2         bKO     deltaKO 
"GSF6-7715" "GSF6-7714" "GSF6-7713" 

$`AMED1221524-WGS`$`TUSF6-3906`
  epsilonKO 
"GSF6-7709" 

$`AMED1221524-WGS`$`TUSF6-2051`
       cKO1 
"GSF6-4028" 

$`AMED1221524-WGS`$`TUSF6-3909`
        aKO 
```

```
"GSF6-7716"


$YPES637386
$YPES637386$`TUKE8-96`
       aKO         bKO     deltaKO     alphaKO     gammaKO      betaKO 
"GKE8-3630" "GKE8-3629" "GKE8-3628" "GKE8-3627" "GKE8-3626" "GKE8-3625" 
  epsilonKO 
"GKE8-3624" 

$YPES637386$noTU
cKO 
 NA 


$ZGAL63186
$ZGAL63186$`TUJN9-994`
   gammaKO1    alphaKO1     deltaKO        bKO1        cKO1        aKO1 
"GJN9-1783" "GJN9-1782" "GJN9-1781" "GJN9-1780" "GJN9-1779" "GJN9-1778" 

$ZGAL63186$`TUJN9-2176`
   gammaKO2    alphaKO2        bKO2        cKO2        aKO2 
"GJN9-3879" "GJN9-3878" "GJN9-3877" "GJN9-3876" "GJN9-3875" 

$ZGAL63186$`TUJN9-797`
    betaKO1  epsilonKO1 
"GJN9-1406" "GJN9-1405" 

$ZGAL63186$`TUJN9-2175`
 epsilonKO2     betaKO2 
"GJN9-3872" "GJN9-3871" 


$ZMOB627344
$ZMOB627344$`TULN0-609`
    deltaKO     alphaKO     gammaKO      betaKO 
"GLN0-1009" "GLN0-1008" "GLN0-1007" "GLN0-1006" 

$ZMOB627344$`TULN0-608`
  epsilonKO 
"GLN0-1005" 

$ZMOB627344$`TULN0-400`
       aKO        cKO       bKO2       bKO1 
"GLN0-634" "GLN0-633" "GLN0-632" "GLN0-631" 


$`ZMOB627343-WGS`
$`ZMOB627343-WGS`$`TUSZ4-650`
    deltaKO     alphaKO     gammaKO      betaKO   epsilonKO 
"GSZ4-1008" "GSZ4-1007" "GSZ4-1006" "GSZ4-1005" "GSZ4-1004" 

$`ZMOB627343-WGS`$`TUSZ4-436`
       aKO        cKO       bKO2       bKO1 
```

"GSZ4-623" "GSZ4-622" "GSZ4-621" "GSZ4-620"

$ZMOB555217
$ZMOB555217$`TULMZ-570`
betaKO gammaKO alphaKO deltaKO
"GLMZ-828" "GLMZ-827" "GLMZ-826" "GLMZ-825"

$ZMOB555217$`TULMZ-571`
epsilonKO
"GLMZ-829"

$ZMOB555217$`TULMZ-448`
aKO cKO
"GLMZ-635" "GLMZ-634"

$ZMOB555217$`TULMZ-447`
bKO2 bKO1
"GLMZ-633" "GLMZ-632"

$LRHA568704
$LRHA568704$`TUHIC-709`
epsilonKO betaKO gammaKO alphaKO deltaKO bKO
"GHIC-1453" "GHIC-1454" "GHIC-1457" "GHIC-1451" "GHIC-1458" "GHIC-1456"
cKO aKO
"GHIC-1455" "GHIC-1452"

$ZMOB622759
$ZMOB622759$`TUI1C-619`
deltaKO alphaKO gammaKO betaKO epsilonKO
"GI1C-1103" "GI1C-1102" "GI1C-1101" "GI1C-1100" "GI1C-1099"

$ZMOB622759$`TUI1C-404`
aKO cKO
"GI1C-716" "GI1C-715"

$ZMOB622759$`TUI1C-403`
bKO2 bKO1
"GI1C-714" "GI1C-713"

$BCER405535
$BCER405535$`TUHSL-3382`
aKO cKO bKO deltaKO alphaKO gammaKO
"GHSL-5417" "GHSL-5416" "GHSL-5415" "GHSL-5414" "GHSL-5413" "GHSL-5412"
betaKO epsilonKO
"GHSL-5411" "GHSL-5410"

$BCAV471853
$BCAV471853$`TUI1Z-663`
betaKO gammaKO alphaKO deltaKO bKO cKO

"GI1Z-1323" "GI1Z-1322" "GI1Z-1321" "GI1Z-1320" "GI1Z-1319" "GI1Z-1318"
aKO
"GI1Z-1317"

$BCAV471853$`TUI1Z-664`
epsilonKO
"GI1Z-1324"

$BCER572264
$BCER572264$`TUH22-3279`
aKO cKO bKO deltaKO alphaKO gammaKO
"GH22-5382" "GH22-5381" "GH22-5380" "GH22-5379" "GH22-5378" "GH22-5377"
betaKO epsilonKO
"GH22-5376" "GH22-5375"

$LWEL386043
$LWEL386043$`TUI5X-240`
epsilonKO1 betaKO1 gammaKO1 alphaKO1 deltaKO1 cKO1
"GI5X-442" "GI5X-441" "GI5X-440" "GI5X-439" "GI5X-438" "GI5X-437"

$LWEL386043$`TUI5X-1318`
aKO cKO2 bKO deltaKO2 alphaKO2 gammaKO2
"GI5X-2559" "GI5X-2558" "GI5X-2557" "GI5X-2556" "GI5X-2555" "GI5X-2554"
betaKO2 epsilonKO2
"GI5X-2553" "GI5X-2552"

$BCYT315749
$BCYT315749$`TUH2A-2327`
aKO cKO bKO deltaKO alphaKO gammaKO
"GH2A-3966" "GH2A-3965" "GH2A-3964" "GH2A-3963" "GH2A-3962" "GH2A-3961"
betaKO epsilonKO
"GH2A-3960" "GH2A-3959"

$ZMOB264203
$ZMOB264203$`TUC4T-2877`
epsilonKO betaKO gammaKO alphaKO deltaKO
"GC4T-382" "GC4T-381" "GC4T-380" "GC4T-379" "GC4T-378"

$ZMOB264203$`TUC4T-3091`
cKO aKO
"GC4T-764" "GC4T-763"

$ZMOB264203$`TUC4T-3092`
bKO2 bKO1
"GC4T-766" "GC4T-765"

$ZMOB579138
$ZMOB579138$`TUJDN-615`
deltaKO alphaKO gammaKO betaKO

```
"GJDN-965" "GJDN-964" "GJDN-963" "GJDN-962"

$ZMOB579138$`TUJDN-614`
 epsilonKO
"GJDN-961"

$ZMOB579138$`TUJDN-394`
       aKO        cKO
"GJDN-585" "GJDN-584"

$ZMOB579138$`TUJDN-393`
      bKO2       bKO1
"GJDN-583" "GJDN-582"


$ZPRO655815
$ZPRO655815$`TUI6J-27`
  gammaKO   alphaKO
"GI6J-66" "GI6J-65"

$ZPRO655815$`TUI6J-2601`
     betaKO
"GI6J-4510"

$ZPRO655815$`TUI6J-26`
  deltaKO       bKO       cKO       aKO
"GI6J-64" "GI6J-63" "GI6J-62" "GI6J-61"

$ZPRO655815$`TUI6J-2600`
  epsilonKO
"GI6J-4509"


$`ECOL405955-WGS`
$`ECOL405955-WGS`$`TUSN2-2206`
    gammaKO      betaKO
"GSN2-3761" "GSN2-3760"

$`ECOL405955-WGS`$`TUSN2-2209`
        aKO
"GSN2-3764"

$`ECOL405955-WGS`$noTU
  alphaKO   deltaKO epsilonKO       cKO       bKO
       NA        NA        NA        NA        NA


$ECOL910348
$ECOL910348$`TUI9X-2247`
        aKO         cKO         bKO     deltaKO     gammaKO      betaKO
"GI9X-3945" "GI9X-3944" "GI9X-3943" "GI9X-3942" "GI9X-3940" "GI9X-3939"
  epsilonKO
"GI9X-3938"
```

```
$ECOL910348$noTU
alphaKO
     NA



$LRUM1069534
$LRUM1069534$`TUJVX-735`
        aKO         cKO         bKO     deltaKO     alphaKO     gammaKO
"GJVX-1415" "GJVX-1414" "GJVX-1413" "GJVX-1412" "GJVX-1411" "GJVX-1410"
     betaKO   epsilonKO
"GJVX-1409" "GJVX-1408"



$`GPAM657308-WGS`
$`GPAM657308-WGS`$`TUSOA-1274`
        bKO     gammaKO      betaKO   epsilonKO
"GSOA-1881" "GSOA-1880" "GSOA-1879" "GSOA-1878"

$`GPAM657308-WGS`$noTU
alphaKO deltaKO     cKO     aKO
     NA      NA      NA      NA



$MTUB1091501
$MTUB1091501$`TULGZ-707`
  epsilonKO      betaKO     gammaKO         bKO         cKO         aKO
"GLGZ-1227" "GLGZ-1226" "GLGZ-1225" "GLGZ-1223" "GLGZ-1222" "GLGZ-1221"

$MTUB1091501$noTU
alphaKO deltaKO
     NA      NA



$`BBAC245012-WGS`
$`BBAC245012-WGS`$`TUSI2-1103`
        aKO         cKO     gammaKO
"GSI2-1627" "GSI2-1626" "GSI2-1625"

$`BBAC245012-WGS`$noTU
  alphaKO    betaKO   deltaKO epsilonKO       bKO
       NA        NA        NA        NA        NA



$`HPYL1248726-WGS`
$`HPYL1248726-WGS`$`TUSOC-466`
       bKO2        bKO1     deltaKO     alphaKO     gammaKO      betaKO
"GSOC-1063" "GSOC-1062" "GSOC-1061" "GSOC-1060" "GSOC-1059" "GSOC-1058"
  epsilonKO
"GSOC-1057"

$`HPYL1248726-WGS`$`TUSOC-509`
        cKO
"GSOC-1144"
```

```
$`HPYL1248726-WGS`$`TUSOC-218`
      aKO
"GSOC-529"


$`MTUB1304279-WGS`
$`MTUB1304279-WGS`$`TUSRF-777`
  epsilonKO    gammaKO        bKO        cKO        aKO
"GSRF-1251" "GSRF-1250" "GSRF-1248" "GSRF-1247" "GSRF-1246"

$`MTUB1304279-WGS`$noTU
alphaKO  betaKO deltaKO
     NA      NA      NA


$FMAG334413
$FMAG334413$`TUJ6M-575`
       bKO
"GJ6M-1131"

$FMAG334413$noTU
  alphaKO    betaKO   gammaKO   deltaKO epsilonKO       cKO       aKO
       NA        NA        NA        NA        NA        NA        NA


$SCOC760011
$SCOC760011$`TUHPJ-387`
      bKO
"GHPJ-794"

$SCOC760011$noTU
  alphaKO    betaKO   gammaKO   deltaKO epsilonKO       cKO       aKO
       NA        NA        NA        NA        NA        NA        NA


$TACI525903
$TACI525903$`TUH4K-215`
      bKO
"GH4K-524"

$TACI525903$noTU
  alphaKO    betaKO   gammaKO   deltaKO epsilonKO       cKO       aKO
       NA        NA        NA        NA        NA        NA        NA


$BCER288681
$BCER288681$`TUHG7-3309`
       aKO         cKO         bKO     deltaKO     alphaKO     gammaKO
"GHG7-5090" "GHG7-5089" "GHG7-5088" "GHG7-5087" "GHG7-5086" "GHG7-5085"
     betaKO   epsilonKO
"GHG7-5084" "GHG7-5083"


$BDEN401473
```

```
$BDEN401473$`TUH09-1158`
       aKO         cKO         bKO     deltaKO     alphaKO     gammaKO 
"GH09-1893" "GH09-1892" "GH09-1891" "GH09-1890" "GH09-1889" "GH09-1888" 
    betaKO   epsilonKO 
"GH09-1887" "GH09-1886" 


$`BEXO1184267-WGS`
$`BEXO1184267-WGS`$`TUSGL-1215`
      bKO2        bKO1     deltaKO     alphaKO     gammaKO      betaKO 
"GSGL-2645" "GSGL-2644" "GSGL-2643" "GSGL-2642" "GSGL-2641" "GSGL-2640" 
  epsilonKO 
"GSGL-2639" 

$`BEXO1184267-WGS`$`TUSGL-3`
     cKO       aKO 
"GSGL-10"  "GSGL-9" 


$`LRHA1316933-WGS`
$`LRHA1316933-WGS`$`TUSPB-626`
   gammaKO     alphaKO     deltaKO         bKO         cKO         aKO 
"GSPB-1142" "GSPB-1141" "GSPB-1140" "GSPB-1139" "GSPB-1138" "GSPB-1137" 

$`LRHA1316933-WGS`$`TUSPB-627`
  epsilonKO      betaKO 
"GSPB-1145" "GSPB-1144" 


$BFAE446465
$BFAE446465$`TUH3P-995`
       cKO         bKO     deltaKO     alphaKO     gammaKO      betaKO 
"GH3P-1913" "GH3P-1912" "GH3P-1911" "GH3P-1910" "GH3P-1909" "GH3P-1908" 
  epsilonKO 
"GH3P-1907" 

$BFAE446465$`TUH3P-996`
       aKO 
"GH3P-1914" 


$BFRA862962
$BFRA862962$`TUHND-1159`
   gammaKO     alphaKO     deltaKO         bKO         cKO         aKO 
"GHND-2212" "GHND-2211" "GHND-2210" "GHND-2209" "GHND-2208" "GHND-2207" 
  epsilonKO      betaKO 
"GHND-2205" "GHND-2204" 


$`HPYL1248725-WGS`
$`HPYL1248725-WGS`$`TUSP8-490`
      bKO2        bKO1     deltaKO     alphaKO     gammaKO      betaKO 
"GSP8-1128" "GSP8-1127" "GSP8-1126" "GSP8-1125" "GSP8-1124" "GSP8-1123" 
  epsilonKO 
```

```
"GSP8-1122"

$`HPYL1248725-WGS`$`TUSP8-529`
        cKO
"GSP8-1207"

$`HPYL1248725-WGS`$`TUSP8-376`
       aKO
"GSP8-855"


$`BFIB657324-WGS`
$`BFIB657324-WGS`$`TUSIP-1395`
       cKO2     deltaKO     alphaKO    gammaKO2     betaKO2  epsilonKO2
"GSIP-2496" "GSIP-2495" "GSIP-2494" "GSIP-2493" "GSIP-2492" "GSIP-2491"

$`BFIB657324-WGS`$`TUSIP-416`
epsilonKO1    betaKO1   gammaKO1       cKO1
"GSIP-724" "GSIP-723" "GSIP-722" "GSIP-721"

$`BFIB657324-WGS`$noTU
aKO bKO
 NA  NA


$BFLO203907
$BFLO203907$`TUHF7-2`
epsilonKO    betaKO   gammaKO   alphaKO   deltaKO       bKO       cKO       aKO
 "GHF7-9"  "GHF7-8"  "GHF7-7"  "GHF7-6"  "GHF7-5"  "GHF7-4"  "GHF7-3"  "GHF7-2"


$`BFRA295405-WGS`
$`BFRA295405-WGS`$`TUSGF-1130`
    gammaKO     alphaKO     deltaKO         bKO         cKO         aKO
"GSGF-2200" "GSGF-2199" "GSGF-2198" "GSGF-2197" "GSGF-2196" "GSGF-2195"
  epsilonKO      betaKO
"GSGF-2193" "GSGF-2192"


$BFRA272559
$BFRA272559$`TUKF0-1132`
    gammaKO     alphaKO     deltaKO         bKO         cKO         aKO
"GKF0-2168" "GKF0-2167" "GKF0-2166" "GKF0-2165" "GKF0-2164" "GKF0-2163"
  epsilonKO      betaKO
"GKF0-2161" "GKF0-2160"


$BGLA999541
$BGLA999541$`TUHSQ-2310`
epsilonKO    betaKO   gammaKO   alphaKO   deltaKO       bKO       cKO       aKO
"GHSQ-93" "GHSQ-92" "GHSQ-91" "GHSQ-90" "GHSQ-89" "GHSQ-88" "GHSQ-87" "GHSQ-86"


$BSP640511
```

$BSP640511$`TUJ7J-2230`
aKO cKO bKO deltaKO alphaKO gammaKO
"GJ7J-3177" "GJ7J-3176" "GJ7J-3175" "GJ7J-3174" "GJ7J-3173" "GJ7J-3172"
betaKO epsilonKO
"GJ7J-3171" "GJ7J-3170"

$BSP640512
$BSP640512$`TUBXV-1141`
betaKO1 epsilonKO1 aKO1 cKO1 bKO1 alphaKO1
"GBXV-1785" "GBXV-1784" "GBXV-1781" "GBXV-1780" "GBXV-1779" "GBXV-1778"
gammaKO1
"GBXV-1777"

$BSP640512$`TUBXV-2139`
aKO2 cKO2 bKO2 deltaKO alphaKO2 gammaKO2
"GBXV-3523" "GBXV-3522" "GBXV-3521" "GBXV-3520" "GBXV-3519" "GBXV-3518"
betaKO2 epsilonKO2
"GBXV-3517" "GBXV-3516"

$BGLU626418
$BGLU626418$`TUJI5-1684`
epsilonKO betaKO gammaKO alphaKO deltaKO bKO cKO aKO
"GJI5-78" "GJI5-77" "GJI5-76" "GJI5-75" "GJI5-74" "GJI5-73" "GJI5-72" "GJI5-71"

$`LREU1358027-WGS`
$`LREU1358027-WGS`$`TUSPA-262`
epsilonKO betaKO gammaKO alphaKO deltaKO bKO cKO
"GSPA-504" "GSPA-503" "GSPA-502" "GSPA-501" "GSPA-500" "GSPA-499" "GSPA-498"
aKO
"GSPA-497"

$BGRA634504
$BGRA634504$`TUJI6-1148`
deltaKO alphaKO gammaKO betaKO epsilonKO
"GJI6-1877" "GJI6-1876" "GJI6-1875" "GJI6-1874" "GJI6-1873"

$BGRA634504$`TUJI6-289`
bKO2 bKO1 cKO aKO
"GJI6-446" "GJI6-445" "GJI6-444" "GJI6-443"

$LIVA881621
$LIVA881621$`TUJTC-665`
epsilonKO1 gammaKO1 alphaKO1 cKO1
"GJTC-897" "GJTC-895" "GJTC-894" "GJTC-891"

$LIVA881621$`TUJTC-2568`
aKO cKO2 bKO deltaKO alphaKO2 gammaKO2
"GJTC-3651" "GJTC-3650" "GJTC-3649" "GJTC-3648" "GJTC-3647" "GJTC-3646"
betaKO

"GJTC-3645"

$LIVA881621$`TUJTC-2567`
epsilonKO2
"GJTC-3643"

$BHAL272558
$BHAL272558$`TUJC5-2140`
aKO cKO bKO deltaKO alphaKO gammaKO
"GJC5-3863" "GJC5-3862" "GJC5-3861" "GJC5-3860" "GJC5-3859" "GJC5-3858"
betaKO epsilonKO
"GJC5-3857" "GJC5-3856"

$BHEN283166
$BHEN283166$`TUIVZ-881`
deltaKO alphaKO gammaKO betaKO epsilonKO
"GIVZ-1530" "GIVZ-1529" "GIVZ-1528" "GIVZ-1527" "GIVZ-1526"

$BHEN283166$`TUIVZ-231`
bKO2 bKO1 cKO aKO
"GIVZ-414" "GIVZ-413" "GIVZ-412" "GIVZ-411"

$BHEL693979
$BHEL693979$`TUHID-1342`
betaKO epsilonKO aKO cKO bKO deltaKO
"GHID-2554" "GHID-2553" "GHID-2551" "GHID-2550" "GHID-2549" "GHID-2548"
alphaKO gammaKO
"GHID-2547" "GHID-2546"

$BIND395963
$BIND395963$`TUJA7-286`
deltaKO alphaKO1
"GJA7-222" "GJA7-221"

$BIND395963$`TUJA7-1938`
gammaKO2 alphaKO2 bKO3 cKO2 aKO2 epsilonKO2
"GJA7-2822" "GJA7-2821" "GJA7-2820" "GJA7-2819" "GJA7-2818" "GJA7-2816"
betaKO2
"GJA7-2815"

$BIND395963$`TUJA7-285`
gammaKO1 betaKO1 epsilonKO1
"GJA7-220" "GJA7-219" "GJA7-218"

$BIND395963$`TUJA7-612`
aKO1 cKO1
"GJA7-754" "GJA7-753"

$BIND395963$`TUJA7-610`
bKO1

```
"GJA7-751"

$BIND395963$`TUJA7-611`
      bKO2
"GJA7-752"



$`BINF1367477-WGS`
$`BINF1367477-WGS`$`TUSFY-2584`
        aKO         cKO         bKO     deltaKO     alphaKO     gammaKO
"GSFY-4720" "GSFY-4719" "GSFY-4718" "GSFY-4717" "GSFY-4716" "GSFY-4715"
     betaKO   epsilonKO
"GSFY-4714" "GSFY-4713"



$BINT1045858
$BINT1045858$`TUL9O-1579`
        aKO         cKO         bKO    deltaKO2    deltaKO1     alphaKO
"GL9O-2499" "GL9O-2498" "GL9O-2497" "GL9O-2496" "GL9O-2495" "GL9O-2494"

$BINT1045858$`TUL9O-1058`
     betaKO
"GL9O-1697"

$BINT1045858$`TUL9O-1578`
    gammaKO
"GL9O-2493"

$BINT1045858$`TUL9O-418`
 epsilonKO
"GL9O-644"



$BSP1127744
$BSP1127744$`TUL8I-1968`
        aKO         cKO         bKO     deltaKO     alphaKO     gammaKO
"GL8I-3879" "GL8I-3878" "GL8I-3877" "GL8I-3876" "GL8I-3875" "GL8I-3874"
     betaKO   epsilonKO
"GL8I-3873" "GL8I-3872"



$BJAP1037409
$BJAP1037409$`TUL9L-248`
   deltaKO    alphaKO    gammaKO     betaKO  epsilonKO
"GL9L-408" "GL9L-407" "GL9L-406" "GL9L-405" "GL9L-404"

$BJAP1037409$`TUL9L-5186`
        cKO
"GL9L-8675"

$BJAP1037409$`TUL9L-5185`
        aKO
"GL9L-8674"
```

```
$BJAP1037409$`TUL9L-5187`
      bKO2        bKO1
"GL9L-8677" "GL9L-8676"


$BANI442563
$BANI442563$`TUHG0-421`
      aKO        cKO        bKO    deltaKO    alphaKO    gammaKO     betaKO
"GHG0-631" "GHG0-630" "GHG0-629" "GHG0-628" "GHG0-627" "GHG0-626" "GHG0-625"
 epsilonKO
"GHG0-624"


$`LREU1340495-WGS`
$`LREU1340495-WGS`$`TUSPM-899`
       aKO          cKO          bKO      deltaKO      alphaKO      gammaKO
"GSPM-1526" "GSPM-1525" "GSPM-1524" "GSPM-1523" "GSPM-1522" "GSPM-1521"
     betaKO   epsilonKO
"GSPM-1520" "GSPM-1519"


$LMON568819
$LMON568819$`TUJF9-97`
epsilonKO1    betaKO1   gammaKO1   alphaKO1   deltaKO1       cKO1
"GJF9-148" "GJF9-147" "GJF9-146" "GJF9-144" "GJF9-143" "GJF9-142"

$LMON568819$`TUJF9-2553`
       aKO        cKO2         bKO    deltaKO2    alphaKO2    gammaKO2
"GJF9-3717" "GJF9-3716" "GJF9-3715" "GJF9-3714" "GJF9-3713" "GJF9-3712"
    betaKO2
"GJF9-3711"

$LMON568819$`TUJF9-2552`
 epsilonKO2
"GJF9-3709"


$BLON890402
$BLON890402$`TUJ8K-752`
    alphaKO     gammaKO      betaKO   epsilonKO
"GJ8K-1144" "GJ8K-1143" "GJ8K-1142" "GJ8K-1141"

$BLON890402$`TUJ8K-753`
       bKO     deltaKO
"GJ8K-1146" "GJ8K-1145"

$BLON890402$`TUJ8K-754`
       cKO
"GJ8K-1147"

$BLON890402$`TUJ8K-755`
       aKO
"GJ8K-1148"
```

```
$BANI580050
$BANI580050$`TUI23-921`
      aKO        cKO        bKO    deltaKO    alphaKO    gammaKO 
"GI23-1435" "GI23-1434" "GI23-1433" "GI23-1432" "GI23-1431" "GI23-1430" 
    betaKO  epsilonKO 
"GI23-1429" "GI23-1428" 



$BLIC279010
$BLIC279010$`TUJ2P-2100`
      bKO    deltaKO    alphaKO 
"GJ2P-3883" "GJ2P-3882" "GJ2P-3881" 

$BLIC279010$`TUJ2P-2099`
   gammaKO     betaKO  epsilonKO 
"GJ2P-3880" "GJ2P-3879" "GJ2P-3878" 

$BLIC279010$`TUJ2P-2101`
      cKO 
"GJ2P-3884" 

$BLIC279010$`TUJ2P-2102`
      aKO 
"GJ2P-3885" 



$BLON565040
$BLON565040$`TUHFW-162`
 epsilonKO     betaKO    gammaKO    alphaKO    deltaKO        bKO        cKO 
"GHFW-250" "GHFW-249" "GHFW-248" "GHFW-247" "GHFW-246" "GHFW-245" "GHFW-244" 
      aKO 
"GHFW-243" 



$ABOR393595
$ABOR393595$`TUHRI-1544`
      aKO        cKO        bKO    deltaKO    alphaKO    gammaKO 
"GHRI-2783" "GHRI-2782" "GHRI-2781" "GHRI-2780" "GHRI-2779" "GHRI-2778" 
    betaKO  epsilonKO 
"GHRI-2777" "GHRI-2776" 



$ABAU889738
$ABAU889738$`TUL7L-2095`
      cKO        bKO    deltaKO    alphaKO    gammaKO     betaKO 
"GL7L-3632" "GL7L-3631" "GL7L-3630" "GL7L-3629" "GL7L-3628" "GL7L-3627" 
  epsilonKO 
"GL7L-3626" 

$ABAU889738$`TUL7L-2096`
      aKO 
"GL7L-3633" 
```

```
$`ABRA1064539-WGS`
$`ABRA1064539-WGS`$`TUSFJ-1157`
 epsilonKO     betaKO    gammaKO    alphaKO    deltaKO 
"GSFJ-933" "GSFJ-932" "GSFJ-931" "GSFJ-930" "GSFJ-929" 

$`ABRA1064539-WGS`$`TUSFJ-1308`
        aKO         cKO        bKO2        bKO1 
"GSFJ-1228" "GSFJ-1227" "GSFJ-1226" "GSFJ-1225" 


$ABUT944546
$ABUT944546$`TUL87-610`
       bKO2        bKO1     deltaKO     alphaKO     gammaKO      betaKO 
"GL87-1515" "GL87-1514" "GL87-1513" "GL87-1512" "GL87-1511" "GL87-1510" 
  epsilonKO 
"GL87-1509" 

$ABUT944546$`TUL87-658`
        cKO 
"GL87-1619" 

$ABUT944546$`TUL87-762`
        aKO 
"GL87-1897" 


$`BLON722911-WGS`
$`BLON722911-WGS`$`TUSGW-917`
        aKO         cKO         bKO     deltaKO     alphaKO     gammaKO 
"GSGW-1403" "GSGW-1402" "GSGW-1401" "GSGW-1400" "GSGW-1399" "GSGW-1398" 
     betaKO   epsilonKO 
"GSGW-1397" "GSGW-1396" 


$`BLIC766760-WGS`
$`BLIC766760-WGS`$`TUSFZ-2142`
        aKO         cKO         bKO     deltaKO     alphaKO     gammaKO 
"GSFZ-3927" "GSFZ-3926" "GSFZ-3925" "GSFZ-3924" "GSFZ-3923" "GSFZ-3922" 
     betaKO   epsilonKO 
"GSFZ-3921" "GSFZ-3920" 


$LPNE297246
$LPNE297246$`TUCO9-2109`
   alphaKO1        bKO1 
"GCO9-3003" "GCO9-3002" 

$LPNE297246$`TUCO9-2675`
       bKO2     deltaKO    alphaKO2    gammaKO2     betaKO2  epsilonKO2 
"GCO9-3878" "GCO9-3877" "GCO9-3876" "GCO9-3875" "GCO9-3874" "GCO9-3873" 

$LPNE297246$`TUCO9-2105`
    betaKO1 
```

"GCO9-2993"

$LPNE297246$`TUCO9-2110`
gammaKO1
"GCO9-3004"

$LPNE297246$`TUCO9-2106`
epsilonKO1
"GCO9-2995"

$LPNE297246$`TUCO9-2108`
cKO1
"GCO9-3000"

$LPNE297246$`TUCO9-2676`
aKO2 cKO2
"GCO9-3881" "GCO9-3880"

$LPNE297246$`TUCO9-2107`
aKO1
"GCO9-2998"

$LREU491077
$LREU491077$`TUH1M-971`
epsilonKO betaKO gammaKO alphaKO deltaKO bKO
"GH1M-1762" "GH1M-1761" "GH1M-1760" "GH1M-1759" "GH1M-1758" "GH1M-1757"
cKO aKO
"GH1M-1756" "GH1M-1755"

$BLON205913
$BLON205913$`TUJB4-736`
cKO bKO deltaKO alphaKO gammaKO betaKO
"GJB4-1165" "GJB4-1164" "GJB4-1163" "GJB4-1162" "GJB4-1161" "GJB4-1160"
epsilonKO
"GJB4-1159"

$BLON205913$`TUJB4-737`
aKO
"GJB4-1166"

$BLON1035817
$BLON1035817$`TUL98-1076`
aKO cKO bKO deltaKO alphaKO gammaKO
"GL98-1694" "GL98-1693" "GL98-1692" "GL98-1691" "GL98-1690" "GL98-1689"
betaKO epsilonKO
"GL98-1688" "GL98-1687"

$BLON759350
$BLON759350$`TUHJA-178`
epsilonKO betaKO gammaKO alphaKO deltaKO bKO cKO

```
"GHJA-276" "GHJA-275" "GHJA-274" "GHJA-273" "GHJA-272" "GHJA-271" "GHJA-270"
       aKO
"GHJA-269"



$BLON565042
$BLON565042$`TUIWN-177`
 epsilonKO     betaKO    gammaKO    alphaKO    deltaKO        bKO        cKO
"GIWN-266" "GIWN-265" "GIWN-264" "GIWN-263" "GIWN-262" "GIWN-261" "GIWN-260"
       aKO
"GIWN-259"



$BLON391904
$BLON391904$`TUCDR-203`
 epsilonKO     betaKO    gammaKO    alphaKO    deltaKO        bKO        cKO
"GCDR-321" "GCDR-320" "GCDR-319" "GCDR-318" "GCDR-317" "GCDR-316" "GCDR-315"
       aKO
"GCDR-314"



$BLON206672
$BLON206672$`TUI1E-668`
        aKO         cKO         bKO     deltaKO     alphaKO     gammaKO
"GI1E-1043" "GI1E-1042" "GI1E-1041" "GI1E-1040" "GI1E-1039" "GI1E-1038"
     betaKO   epsilonKO
"GI1E-1037" "GI1E-1036"



$BANI742729
$BANI742729$`TUL8Z-912`
        aKO         cKO         bKO     deltaKO     alphaKO     gammaKO
"GL8Z-1466" "GL8Z-1465" "GL8Z-1464" "GL8Z-1463" "GL8Z-1462" "GL8Z-1461"
     betaKO   epsilonKO
"GL8Z-1460" "GL8Z-1459"



$BANI555970
$BANI555970$`TUJ22-923`
        aKO         cKO         bKO     deltaKO     alphaKO     gammaKO
"GJ22-1434" "GJ22-1433" "GJ22-1432" "GJ22-1431" "GJ22-1430" "GJ22-1429"
     betaKO   epsilonKO
"GJ22-1428" "GJ22-1427"



$BANI573236
$BANI573236$`TUL92-923`
        aKO         cKO         bKO     deltaKO     alphaKO     gammaKO
"GL92-1441" "GL92-1440" "GL92-1439" "GL92-1438" "GL92-1437" "GL92-1436"
     betaKO   epsilonKO
"GL92-1435" "GL92-1434"



$`CJEJ1380768-WGS`
```

```
$`CJEJ1380768-WGS`$`TUSHW-54`
 epsilonKO     betaKO    gammaKO    alphaKO    deltaKO       bKO2       bKO1
"GSHW-100"  "GSHW-99"  "GSHW-98"  "GSHW-97"  "GSHW-96"  "GSHW-95"  "GSHW-94"

$`CJEJ1380768-WGS`$`TUSHW-370`
       cKO
"GSHW-947"

$`CJEJ1380768-WGS`$`TUSHW-464`
        aKO
"GSHW-1210"


$LSAK314315
$LSAK314315$`TUCKE-1569`
    alphaKO     gammaKO      betaKO
"GCKE-2054" "GCKE-2053" "GCKE-2052"

$LSAK314315$`TUCKE-1570`
        bKO     deltaKO
"GCKE-2057" "GCKE-2056"

$LSAK314315$`TUCKE-1568`
  epsilonKO
"GCKE-2050"

$LSAK314315$`TUCKE-1572`
        cKO
"GCKE-2059"

$LSAK314315$`TUCKE-1573`
        aKO
"GCKE-2061"


$LSEE683837
$LSEE683837$`TUI10-300`
epsilonKO1    betaKO1   gammaKO1   alphaKO1   deltaKO1       cKO1
"GI10-560" "GI10-559" "GI10-558" "GI10-557" "GI10-556" "GI10-555"

$LSEE683837$`TUI10-1260`
        aKO        cKO2         bKO    deltaKO2    alphaKO2    gammaKO2
"GI10-2512" "GI10-2511" "GI10-2510" "GI10-2509" "GI10-2508" "GI10-2507"
    betaKO2  epsilonKO2
"GI10-2506" "GI10-2505"


$BANI1168290
$BANI1168290$`TUL8Y-922`
        aKO         cKO         bKO     deltaKO     alphaKO     gammaKO
"GL8Y-1434" "GL8Y-1433" "GL8Y-1432" "GL8Y-1431" "GL8Y-1430" "GL8Y-1429"
     betaKO   epsilonKO
"GL8Y-1428" "GL8Y-1427"
```

```
$`BMAL243160-WGS`
$`BMAL243160-WGS`$`TUSHH-2897`
 epsilonKO1     betaKO1    gammaKO1    alphaKO1     deltaKO        bKO1
"GSHH-2952" "GSHH-2951" "GSHH-2950" "GSHH-2949" "GSHH-2948" "GSHH-2947"
       cKO1        aKO1
"GSHH-2946" "GSHH-2945"

$`BMAL243160-WGS`$`TUSHH-79`
   gammaKO2    alphaKO2        bKO2        cKO2        aKO2
"GSHH-3525" "GSHH-3524" "GSHH-3523" "GSHH-3522" "GSHH-3521"

$`BMAL243160-WGS`$`TUSHH-78`
 epsilonKO2     betaKO2
"GSHH-3517" "GSHH-3516"


$BABO262698
$BABO262698$`TUJC2-1056`
    deltaKO     alphaKO     gammaKO      betaKO   epsilonKO
"GJC2-1822" "GJC2-1821" "GJC2-1820" "GJC2-1819" "GJC2-1818"

$BABO262698$`TUJC2-244`
      bKO2       bKO1        cKO        aKO
"GJC2-416" "GJC2-415" "GJC2-414" "GJC2-413"


$BABO430066
$BABO430066$`TUHI6-1598`
    deltaKO     alphaKO     gammaKO      betaKO   epsilonKO
"GHI6-1690" "GHI6-1689" "GHI6-1688" "GHI6-1687" "GHI6-1686"

$BABO430066$`TUHI6-792`
      bKO2       bKO1        cKO        aKO
"GHI6-377" "GHI6-376" "GHI6-375" "GHI6-374"


$BMEG592022
$BMEG592022$`TUIVX-3023`
        aKO         cKO         bKO     deltaKO     alphaKO     gammaKO
"GIVX-5140" "GIVX-5139" "GIVX-5138" "GIVX-5137" "GIVX-5136" "GIVX-5135"
     betaKO   epsilonKO
"GIVX-5134" "GIVX-5133"


$BMEL224914
$BMEL224914$`TUCJ0-150`
 epsilonKO     betaKO    gammaKO    alphaKO    deltaKO
"GCJ0-263" "GCJ0-262" "GCJ0-261" "GCJ0-260" "GCJ0-259"

$BMEL224914$`TUCJ0-952`
        aKO         cKO        bKO2        bKO1
"GCJ0-1588" "GCJ0-1587" "GCJ0-1586" "GCJ0-1585"
```

$BMEL359391
$BMEL359391$`TUJOQ-1085`
deltaKO alphaKO gammaKO betaKO epsilonKO
"GJOQ-1854" "GJOQ-1853" "GJOQ-1852" "GJOQ-1851" "GJOQ-1850"

$BMEL359391$`TUJOQ-252`
bKO2 bKO1 cKO aKO
"GJOQ-421" "GJOQ-420" "GJOQ-419" "GJOQ-418"

$BMEL703352
$BMEL703352$`TUL9P-1079`
deltaKO alphaKO gammaKO betaKO epsilonKO
"GL9P-1829" "GL9P-1828" "GL9P-1827" "GL9P-1826" "GL9P-1825"

$BMEL703352$`TUL9P-233`
bKO2 bKO1 cKO aKO
"GL9P-414" "GL9P-413" "GL9P-412" "GL9P-411"

$BMEG1006007
$BMEG1006007$`TUL8N-140`
epsilonKO betaKO gammaKO alphaKO deltaKO bKO cKO
"GL8N-136" "GL8N-135" "GL8N-134" "GL8N-133" "GL8N-132" "GL8N-131" "GL8N-130"
aKO
"GL8N-129"

$MABS561007
$MABS561007$`TUJTG-700`
epsilonKO betaKO gammaKO alphaKO deltaKO bKO
"GJTG-1457" "GJTG-1456" "GJTG-1455" "GJTG-1454" "GJTG-1453" "GJTG-1452"
cKO aKO
"GJTG-1451" "GJTG-1450"

$BMEL546272
$BMEL546272$`TUJOX-1064`
deltaKO alphaKO gammaKO
"GJOX-1801" "GJOX-1800" "GJOX-1799"

$BMEL546272$`TUJOX-1063`
betaKO
"GJOX-1798"

$BMEL546272$`TUJOX-1062`
epsilonKO
"GJOX-1797"

$BMEL546272$`TUJOX-233`
bKO2 bKO1 cKO
"GJOX-402" "GJOX-401" "GJOX-400"

```
$BMEL546272$`TUJOX-232`
      aKO
"GJOX-399"


$LBOR355277
$LBOR355277$`TUHYM-904`
       aKO        cKO        bKO     deltaKO     alphaKO     gammaKO
"GHYM-1737" "GHYM-1736" "GHYM-1735" "GHYM-1734" "GHYM-1733" "GHYM-1732"
     betaKO   epsilonKO
"GHYM-1731" "GHYM-1730"


$LSAL712961
$LSAL712961$`TULFC-418`
    betaKO    gammaKO    alphaKO    deltaKO        bKO
"GLFC-534" "GLFC-533" "GLFC-532" "GLFC-531" "GLFC-530"

$LSAL712961$`TULFC-417`
      aKO
"GLFC-529"

$LSAL712961$noTU
epsilonKO       cKO
       NA        NA


$BMUL395019
$BMUL395019$`TUIYO-2319`
       aKO        cKO        bKO     deltaKO     alphaKO     gammaKO
"GIYO-3163" "GIYO-3162" "GIYO-3161" "GIYO-3160" "GIYO-3159" "GIYO-3158"
     betaKO   epsilonKO
"GIYO-3157" "GIYO-3156"


$BMAL412022
$BMAL412022$`TUJI8-2087`
       aKO        cKO        bKO     deltaKO     alphaKO     gammaKO
"GJI8-1593" "GJI8-1592" "GJI8-1591" "GJI8-1590" "GJI8-1589" "GJI8-1588"
     betaKO   epsilonKO
"GJI8-1587" "GJI8-1586"


$BSP1074889
$BSP1074889$`TUJTT-96`
      aKO       cKO       bKO    deltaKO    alphaKO    gammaKO
"GJTT-283" "GJTT-282" "GJTT-281" "GJTT-280" "GJTT-279" "GJTT-278"

$BSP1074889$`TUJTT-165`
    betaKO  epsilonKO
"GJTT-536" "GJTT-535"


$BMAL320389
```

```
$BMAL320389$`TUH97-2911`
 epsilonKO1     betaKO1    gammaKO1    alphaKO1     deltaKO        bKO1
"GH97-3006" "GH97-3005" "GH97-3004" "GH97-3003" "GH97-3002" "GH97-3001"
       cKO1        aKO1
"GH97-3000" "GH97-2999"

$BMAL320389$`TUH97-79`
   gammaKO2    alphaKO2       bKO2        cKO2        aKO2  epsilonKO2
"GH97-3691" "GH97-3690" "GH97-3689" "GH97-3688" "GH97-3687" "GH97-3684"
    betaKO2
"GH97-3683"


$BMEG545693
$BMEG545693$`TUHSY-3318`
        aKO         cKO         bKO     deltaKO     alphaKO     gammaKO
"GHSY-5154" "GHSY-5153" "GHSY-5152" "GHSY-5151" "GHSY-5150" "GHSY-5149"
     betaKO   epsilonKO
"GHSY-5148" "GHSY-5147"


$BMIC568815
$BMIC568815$`TUJUE-1669`
    deltaKO     alphaKO     gammaKO      betaKO   epsilonKO
"GJUE-1814" "GJUE-1813" "GJUE-1812" "GJUE-1811" "GJUE-1810"

$BMIC568815$`TUJUE-848`
      bKO2       bKO1        cKO       aKO
"GJUE-389" "GJUE-388" "GJUE-387" "GJUE-386"


$BSUI470137
$BSUI470137$`TUJIC-683`
    deltaKO     alphaKO     gammaKO      betaKO   epsilonKO
"GJIC-3281" "GJIC-3280" "GJIC-3279" "GJIC-3278" "GJIC-3277"

$BSUI470137$`TUJIC-996`
      bKO2       bKO1        cKO       aKO
"GJIC-410" "GJIC-409" "GJIC-408" "GJIC-407"


$`MPUL272635-WGS`
$`MPUL272635-WGS`$`TUSRV-114`
   betaKO1   alphaKO1
"GSRV-246" "GSRV-245"

$`MPUL272635-WGS`$`TUSRV-129`
       aKO        cKO        bKO    deltaKO   alphaKO2    gammaKO    betaKO2
"GSRV-284" "GSRV-283" "GSRV-282" "GSRV-281" "GSRV-280" "GSRV-279" "GSRV-278"
 epsilonKO
"GSRV-277"

$`MPUL272635-WGS`$`TUSRV-228`
   betaKO3   alphaKO3
```

```
"GSRV-473" "GSRV-472"

$`MPUL272635-WGS`$`TUSRV-354`
  alphaKO4    betaKO4
"GSRV-733" "GSRV-732"


$BMAL320388
$BMAL320388$`TUHFL-702`
   gammaKO1    alphaKO1        bKO1        cKO1        aKO1
"GHFL-4829" "GHFL-4828" "GHFL-4827" "GHFL-4826" "GHFL-4825"

$BMAL320388$`TUHFL-2785`
       aKO2        cKO2        bKO2     deltaKO    alphaKO2    gammaKO2
"GHFL-3360" "GHFL-3359" "GHFL-3358" "GHFL-3357" "GHFL-3356" "GHFL-3355"
    betaKO2  epsilonKO2
"GHFL-3354" "GHFL-3353"

$BMAL320388$`TUHFL-701`
 epsilonKO1     betaKO1
"GHFL-4821" "GHFL-4820"


$BMEL1029825
$BMEL1029825$`TUL9Q-1042`
    deltaKO     alphaKO     gammaKO      betaKO   epsilonKO
"GL9Q-1756" "GL9Q-1755" "GL9Q-1754" "GL9Q-1753" "GL9Q-1752"

$BMEL1029825$`TUL9Q-232`
      bKO2       bKO1        cKO        aKO
"GL9Q-411" "GL9Q-410" "GL9Q-409" "GL9Q-408"


$BMAR862908
$BMAR862908$`TUJBX-1554`
       bKO2        bKO1     deltaKO     alphaKO     gammaKO      betaKO
"GJBX-3282" "GJBX-3281" "GJBX-3280" "GJBX-3279" "GJBX-3278" "GJBX-3277"
  epsilonKO
"GJBX-3276"

$BMAR862908$`TUJBX-4`
      cKO       aKO
"GJBX-10"  "GJBX-9"


$LSAL362948
$LSAL362948$`TUJDJ-486`
 epsilonKO     betaKO    gammaKO    alphaKO    deltaKO        bKO        cKO
"GJDJ-671" "GJDJ-670" "GJDJ-669" "GJDJ-668" "GJDJ-667" "GJDJ-666" "GJDJ-665"
       aKO
"GJDJ-664"


$BMEL941967
```

```
$BMEL941967$`TUL9M-1051`
    deltaKO     alphaKO     gammaKO      betaKO
"GL9M-1831" "GL9M-1830" "GL9M-1829" "GL9M-1828"

$BMEL941967$`TUL9M-1050`
  epsilonKO
"GL9M-1827"

$BMEL941967$`TUL9M-235`
      bKO2       bKO1        cKO        aKO
"GL9M-418" "GL9M-417" "GL9M-416" "GL9M-415"


$BCER334406
$BCER334406$`TUJ71-3383`
        aKO         cKO         bKO     deltaKO     alphaKO     gammaKO
"GJ71-5375" "GJ71-5374" "GJ71-5373" "GJ71-5372" "GJ71-5371" "GJ71-5370"
     betaKO   epsilonKO
"GJ71-5369" "GJ71-5368"


$BANI703613
$BANI703613$`TUL91-885`
        aKO         cKO         bKO     deltaKO     alphaKO     gammaKO
"GL91-1421" "GL91-1420" "GL91-1419" "GL91-1418" "GL91-1417" "GL91-1416"
     betaKO   epsilonKO
"GL91-1415" "GL91-1414"


$BANI1042403
$BANI1042403$`TUL94-846`
        aKO         cKO         bKO     deltaKO     alphaKO     gammaKO
"GL94-1514" "GL94-1513" "GL94-1512" "GL94-1511" "GL94-1510" "GL94-1509"
     betaKO   epsilonKO
"GL94-1508" "GL94-1507"


$BOVI444178
$BOVI444178$`TUH2V-1568`
    deltaKO     alphaKO     gammaKO      betaKO   epsilonKO
"GH2V-1733" "GH2V-1732" "GH2V-1731" "GH2V-1730" "GH2V-1729"

$BOVI444178$`TUH2V-790`
      bKO2       bKO1        cKO        aKO
"GH2V-395" "GH2V-394" "GH2V-393" "GH2V-392"


$ABAU509173
$ABAU509173$`TUJXF-2080`
        aKO         cKO         bKO     deltaKO     alphaKO     gammaKO
"GJXF-3587" "GJXF-3586" "GJXF-3585" "GJXF-3584" "GJXF-3583" "GJXF-3582"
     betaKO   epsilonKO
"GJXF-3581" "GJXF-3580"
```

```
$ATHE926569
$ATHE926569$`TUH0F-635`
  epsilonKO      betaKO     gammaKO     alphaKO
"GH0F-1407" "GH0F-1406" "GH0F-1405" "GH0F-1404"

$ATHE926569$`TUH0F-484`
        bKO         cKO         aKO
"GH0F-1071" "GH0F-1070" "GH0F-1069"

$ATHE926569$noTU
deltaKO
     NA


$ABAU497978
$ABAU497978$`TUL7S-124`
 epsilonKO     betaKO    gammaKO    alphaKO    deltaKO        bKO        cKO
"GL7S-190" "GL7S-189" "GL7S-188" "GL7S-187" "GL7S-186" "GL7S-185" "GL7S-184"

$ABAU497978$`TUL7S-123`
       aKO
"GL7S-182"


$ACAP240015
$ACAP240015$`TUKF4-580`
       bKO2        bKO1     deltaKO     alphaKO     gammaKO      betaKO
"GKF4-1011" "GKF4-1010" "GKF4-1009" "GKF4-1008" "GKF4-1007" "GKF4-1006"
  epsilonKO
"GKF4-1005"

$ACAP240015$`TUKF4-168`
       cKO        aKO
"GKF4-279" "GKF4-278"


$BPAR257311
$BPAR257311$`TU9TP-27259`
      bKO   deltaKO   alphaKO
"BPP4139" "BPP4138" "BPP4137"

$BPAR257311$`TU9TP-27257`
   betaKO epsilonKO
"BPP4135" "BPP4134"

$BPAR257311$`TU9TP-27258`
  gammaKO
"BPP4136"

$BPAR257311$`TU9TP-27260`
      cKO
"BPP4140"
```

$BPAR257311$`TU9TP-27261`
aKO
"BPP4141"

$`BPAR1208660-WGS`
$`BPAR1208660-WGS`$`TUSH2-2052`
aKO cKO bKO deltaKO alphaKO gammaKO
"GSH2-4274" "GSH2-4273" "GSH2-4272" "GSH2-4271" "GSH2-4270" "GSH2-4269"
betaKO epsilonKO
"GSH2-4268" "GSH2-4267"

$APAS634455
$APAS634455$`TUL7C-296`
epsilonKO betaKO gammaKO alphaKO deltaKO
"GL7C-121" "GL7C-120" "GL7C-119" "GL7C-118" "GL7C-117"

$APAS634455$`TUL7C-1516`
aKO cKO
"GL7C-2467" "GL7C-2466"

$APAS634455$`TUL7C-1515`
bKO2 bKO1
"GL7C-2465" "GL7C-2464"

$BPRO515622
$BPRO515622$`TUHKV-431`
epsilonKO1 betaKO1 gammaKO1 alphaKO1 deltaKO bKO cKO1
"GHKV-179" "GHKV-178" "GHKV-177" "GHKV-176" "GHKV-175" "GHKV-174" "GHKV-173"
aKO1
"GHKV-172"

$BPRO515622$`TUHKV-959`
epsilonKO2 betaKO2 gammaKO2 alphaKO2 cKO2 aKO2
"GHKV-1186" "GHKV-1185" "GHKV-1184" "GHKV-1183" "GHKV-1181" "GHKV-1180"

$BPER1017264
$BPER1017264$`TUL9D-1701`
epsilonKO betaKO gammaKO alphaKO deltaKO bKO
"GL9D-3296" "GL9D-3295" "GL9D-3294" "GL9D-3293" "GL9D-3292" "GL9D-3291"
cKO aKO
"GL9D-3290" "GL9D-3289"

$BPER257313
$BPER257313$`TU9TK-20319`
alphaKO deltaKO bKO
"BP3286" "BP3285" "BP3284"

$BPER257313$`TU9TK-20321`
epsilonKO betaKO

"BP3289" "BP3288"

$BPER257313$`TU9TK-20320`
gammaKO
"BP3287"

$BPER257313$`TU9TK-20318`
cKO
"BP3283"

$BPER257313$`TU9TK-20317`
aKO
"BP3282"

$BPER568706
$BPER568706$`TUL9A-174`
epsilonKO betaKO gammaKO alphaKO deltaKO bKO cKO
"GL9A-357" "GL9A-356" "GL9A-355" "GL9A-354" "GL9A-353" "GL9A-352" "GL9A-351"
aKO
"GL9A-350"

$BPSE398511
$BPSE398511$`TUJI9-2398`
aKO cKO bKO deltaKO alphaKO gammaKO
"GJI9-3798" "GJI9-3797" "GJI9-3796" "GJI9-3795" "GJI9-3794" "GJI9-3793"
betaKO epsilonKO
"GJI9-3792" "GJI9-3791"

$PCAR338963
$PCAR338963$`TUKDU-606`
cKO2 aKO2 epsilonKO1 betaKO1 gammaKO1 alphaKO1
"GKDU-1082" "GKDU-1081" "GKDU-1080" "GKDU-1079" "GKDU-1078" "GKDU-1077"
deltaKO1 bKO2 bKO1
"GKDU-1076" "GKDU-1075" "GKDU-1074"

$PCAR338963$`TUKDU-1854`
alphaKO2 bKO3 cKO3 aKO3 epsilonKO2 betaKO2
"GKDU-3279" "GKDU-3278" "GKDU-3277" "GKDU-3276" "GKDU-3273" "GKDU-3272"

$PCAR338963$`TUKDU-1944`
bKO5 bKO4 deltaKO2 alphaKO3 gammaKO3 betaKO3
"GKDU-3438" "GKDU-3437" "GKDU-3436" "GKDU-3435" "GKDU-3434" "GKDU-3433"
epsilonKO3
"GKDU-3432"

$PCAR338963$`TUKDU-1855`
gammaKO2
"GKDU-3280"

$PCAR338963$`TUKDU-10`
cKO1 aKO1

```
"GKDU-18" "GKDU-17"


$BPHY391038
$BPHY391038$`TUI4Z-1984`
    betaKO1  epsilonKO1        aKO1        cKO1        bKO1    alphaKO1
"GI4Z-1155" "GI4Z-1154" "GI4Z-1151" "GI4Z-1150" "GI4Z-1149" "GI4Z-1148"
   gammaKO1
"GI4Z-1147"

$BPHY391038$`TUI4Z-3118`
       aKO2        cKO2        bKO2     deltaKO    alphaKO2    gammaKO2
"GI4Z-3102" "GI4Z-3101" "GI4Z-3100" "GI4Z-3099" "GI4Z-3098" "GI4Z-3097"
    betaKO2  epsilonKO2
"GI4Z-3096" "GI4Z-3095"


$BSP600809
$BSP600809$`TUHSF-29`
  gammaKO   alphaKO   deltaKO       bKO       cKO       aKO
"GHSF-78" "GHSF-77" "GHSF-76" "GHSF-75" "GHSF-74" "GHSF-73"

$BSP600809$`TUHSF-201`
    betaKO  epsilonKO
"GHSF-563" "GHSF-562"


$`BPIL1042417-WGS`
$`BPIL1042417-WGS`$`TUSHC-16`
      cKO       bKO  deltaKO2  deltaKO1   alphaKO   gammaKO
"GSHC-30" "GSHC-29" "GSHC-28" "GSHC-27" "GSHC-26" "GSHC-23"

$`BPIL1042417-WGS`$`TUSHC-55`
   betaKO
"GSHC-97"

$`BPIL1042417-WGS`$`TUSHC-17`
      aKO
"GSHC-31"

$`BPIL1042417-WGS`$noTU
epsilonKO
       NA


$BPIN520461
$BPIN520461$`TUJF0-1065`
    deltaKO     alphaKO     gammaKO      betaKO   epsilonKO
"GJF0-1856" "GJF0-1855" "GJF0-1854" "GJF0-1853" "GJF0-1852"

$BPIN520461$`TUJF0-236`
      bKO2       bKO1        cKO        aKO
"GJF0-417" "GJF0-416" "GJF0-415" "GJF0-414"
```

```
$BPSE1229785
$BPSE1229785$`TULA0-2189`
      aKO1        cKO1        bKO1     deltaKO    alphaKO1    gammaKO1 
"GLA0-4076" "GLA0-4075" "GLA0-4074" "GLA0-4073" "GLA0-4072" "GLA0-4071" 
    betaKO1  epsilonKO1 
"GLA0-4070" "GLA0-4069" 

$BPSE1229785$`TULA0-3550`
    betaKO2  epsilonKO2        aKO2        cKO2        bKO2    alphaKO2 
"GLA0-6719" "GLA0-6718" "GLA0-6715" "GLA0-6714" "GLA0-6713" "GLA0-6712" 
   gammaKO2 
"GLA0-6711" 



$CAZO511995
$CAZO511995$`TUKF1-284`
      cKO        bKO    deltaKO    alphaKO    gammaKO 
"GKF1-391" "GKF1-390" "GKF1-389" "GKF1-388" "GKF1-387" 

$CAZO511995$`TUKF1-142`
    betaKO  epsilonKO 
"GKF1-141" "GKF1-140" 

$CAZO511995$`TUKF1-285`
       aKO 
"GKF1-392" 



$BPSE536230
$BPSE536230$`TUHVQ-2211`
        aKO         cKO         bKO     deltaKO     alphaKO     gammaKO 
"GHVQ-4139" "GHVQ-4138" "GHVQ-4137" "GHVQ-4136" "GHVQ-4135" "GHVQ-4134" 
     betaKO   epsilonKO 
"GHVQ-4133" "GHVQ-4132" 



$`BPRO543913-WGS`
$`BPRO543913-WGS`$`TUSGM-5`
epsilonKO    betaKO   gammaKO   alphaKO   deltaKO       bKO       cKO       aKO 
"GSGM-23" "GSGM-22" "GSGM-21" "GSGM-20" "GSGM-19" "GSGM-18" "GSGM-17" "GSGM-16" 



$`BBAC245018-WGS`
$`BBAC245018-WGS`$`TUSIN-1009`
 epsilonKO2     betaKO2    gammaKO2     alphaKO    deltaKO2        cKO2 
"GSIN-2019" "GSIN-2018" "GSIN-2017" "GSIN-2016" "GSIN-2015" "GSIN-2014" 
       aKO2 
"GSIN-2013" 

$`BBAC245018-WGS`$`TUSIN-997`
 epsilonKO1     betaKO1    gammaKO1    deltaKO1         bKO        cKO1 
"GSIN-1986" "GSIN-1985" "GSIN-1984" "GSIN-1983" "GSIN-1982" "GSIN-1981" 
       aKO1 
```

"GSIN-1980"

$`BBAC245014-WGS`
$`BBAC245014-WGS`$`TUSHP-522`
epsilonKO betaKO gammaKO alphaKO deltaKO cKO aKO
"GSHP-856" "GSHP-855" "GSHP-854" "GSHP-853" "GSHP-852" "GSHP-851" "GSHP-850"

$`BBAC245014-WGS`$noTU
bKO
NA

$MAGA347257
$MAGA347257$`TUC07-158`
betaKO1 alphaKO1
"GC07-317" "GC07-316"

$MAGA347257$`TUC07-191`
epsilonKO betaKO2 gammaKO alphaKO2 deltaKO bKO cKO
"GC07-381" "GC07-380" "GC07-379" "GC07-378" "GC07-377" "GC07-376" "GC07-375"
aKO
"GC07-374"

$BPET340100
$BPET340100$`TUJBO-163`
epsilonKO betaKO gammaKO alphaKO deltaKO bKO cKO
"GJBO-345" "GJBO-344" "GJBO-343" "GJBO-342" "GJBO-341" "GJBO-340" "GJBO-339"
aKO
"GJBO-338"

$BPUM315750
$BPUM315750$`TUH6N-1767`
aKO cKO bKO deltaKO alphaKO gammaKO
"GH6N-3414" "GH6N-3413" "GH6N-3412" "GH6N-3411" "GH6N-3410" "GH6N-3409"
betaKO epsilonKO
"GH6N-3408" "GH6N-3407"

$`BPIL1161918-WGS`
$`BPIL1161918-WGS`$`TUSHD-1008`
gammaKO alphaKO deltaKO2 deltaKO1 bKO cKO
"GSHD-1891" "GSHD-1888" "GSHD-1887" "GSHD-1886" "GSHD-1885" "GSHD-1884"
aKO
"GSHD-1883"

$`BPIL1161918-WGS`$`TUSHD-969`
betaKO
"GSHD-1814"

$`BPIL1161918-WGS`$`TUSHD-399`
epsilonKO

"GSHD-784"

$BPHE1229205
$BPHE1229205$`TULA3-3974`
alphaKO
"GLA3-3485"

$BPHE1229205$`TULA3-3972`
betaKO
"GLA3-3483"

$BPHE1229205$`TULA3-3973`
gammaKO
"GLA3-3484"

$BPHE1229205$`TULA3-3975`
bKO deltaKO
"GLA3-3487" "GLA3-3486"

$BPHE1229205$`TULA3-3971`
epsilonKO
"GLA3-3482"

$BPHE1229205$`TULA3-3976`
cKO
"GLA3-3488"

$BPHE1229205$`TULA3-3977`
aKO
"GLA3-3489"

$BPHY398527
$BPHY398527$`TUJEX-4436`
aKO1 cKO1 bKO1 deltaKO alphaKO1 gammaKO1
"GJEX-3963" "GJEX-3962" "GJEX-3961" "GJEX-3960" "GJEX-3959" "GJEX-3958"
betaKO1 epsilonKO1
"GJEX-3957" "GJEX-3956"

$BPHY398527$`TUJEX-1041`
betaKO2 epsilonKO2 aKO2 cKO2 bKO2 alphaKO2
"GJEX-5633" "GJEX-5632" "GJEX-5629" "GJEX-5628" "GJEX-5627" "GJEX-5626"
gammaKO2
"GJEX-5625"

$BPSE884204
$BPSE884204$`TULA4-2137`
aKO1 cKO1 bKO1 deltaKO alphaKO1 gammaKO1
"GLA4-3692" "GLA4-3691" "GLA4-3690" "GLA4-3689" "GLA4-3688" "GLA4-3687"
betaKO1 epsilonKO1
"GLA4-3686" "GLA4-3685"

$BPSE884204$`TULA4-3370`
betaKO2 epsilonKO2 aKO2 cKO2 bKO2 alphaKO2
"GLA4-5818" "GLA4-5817" "GLA4-5814" "GLA4-5813" "GLA4-5812" "GLA4-5811"
gammaKO2
"GLA4-5810"

$APAS634452
$APAS634452$`TUI0T-293`
epsilonKO betaKO gammaKO alphaKO deltaKO
"GI0T-121" "GI0T-120" "GI0T-119" "GI0T-118" "GI0T-117"

$APAS634452$`TUI0T-1509`
aKO cKO bKO2 bKO1
"GI0T-2468" "GI0T-2467" "GI0T-2466" "GI0T-2465"

$BAMY1001582
$BAMY1001582$`TUL8C-2045`
bKO deltaKO alphaKO gammaKO betaKO epsilonKO
"GL8C-3922" "GL8C-3921" "GL8C-3920" "GL8C-3919" "GL8C-3918" "GL8C-3917"

$BAMY1001582$`TUL8C-2046`
aKO cKO
"GL8C-3924" "GL8C-3923"

$BQUI1225179
$BQUI1225179$`TUL8X-678`
deltaKO alphaKO gammaKO betaKO epsilonKO
"GL8X-1126" "GL8X-1125" "GL8X-1124" "GL8X-1123" "GL8X-1122"

$BQUI1225179$`TUL8X-195`
bKO2 bKO1 cKO aKO
"GL8X-295" "GL8X-294" "GL8X-293" "GL8X-292"

$BQUI283165
$BQUI283165$`TUHZA-723`
deltaKO alphaKO gammaKO betaKO epsilonKO
"GHZA-1224" "GHZA-1223" "GHZA-1222" "GHZA-1221" "GHZA-1220"

$BQUI283165$`TUHZA-191`
bKO2 bKO1 cKO aKO
"GHZA-316" "GHZA-315" "GHZA-314" "GHZA-313"

$RHOM585394
$RHOM585394$`TUHYQ-1498`
aKO1 cKO1 bKO1 deltaKO1 alphaKO1 gammaKO1
"GHYQ-2981" "GHYQ-2980" "GHYQ-2979" "GHYQ-2978" "GHYQ-2977" "GHYQ-2976"
betaKO1 epsilonKO1
"GHYQ-2975" "GHYQ-2974"

```
$RHOM585394$`TUHYQ-1639`
      bKO2    deltaKO2    alphaKO2    gammaKO2     betaKO2  epsilonKO2
"GHYQ-3253" "GHYQ-3252" "GHYQ-3251" "GHYQ-3250" "GHYQ-3249" "GHYQ-3248"

$RHOM585394$`TUHYQ-1640`
      aKO2        cKO2
"GHYQ-3255" "GHYQ-3254"


$BAMY1126211
$BAMY1126211$`TUL8D-2076`
       aKO         cKO         bKO     deltaKO     alphaKO     gammaKO
"GL8D-3921" "GL8D-3920" "GL8D-3919" "GL8D-3918" "GL8D-3917" "GL8D-3916"
     betaKO   epsilonKO
"GL8D-3915" "GL8D-3914"


$BRHI882378
$BRHI882378$`TUJIB-639`
 epsilonKO     betaKO    gammaKO    alphaKO    deltaKO        bKO        cKO
"GJIB-147" "GJIB-146" "GJIB-145" "GJIB-144" "GJIB-143" "GJIB-142" "GJIB-141"
       aKO
"GJIB-140"


$BMUR526224
$BMUR526224$`TUHIV-1178`
    gammaKO     alphaKO    deltaKO2    deltaKO1         bKO         cKO
"GHIV-2012" "GHIV-2010" "GHIV-2009" "GHIV-2008" "GHIV-2007" "GHIV-2006"
        aKO
"GHIV-2005"

$BMUR526224$`TUHIV-1537`
     betaKO
"GHIV-2637"

$BMUR526224$`TUHIV-499`
 epsilonKO
"GHIV-845"


$BSAL667015
$BSAL667015$`TUHA0-165`
   gammaKO    alphaKO    deltaKO        bKO        cKO        aKO  epsilonKO
"GHA0-315" "GHA0-314" "GHA0-313" "GHA0-312" "GHA0-311" "GHA0-310" "GHA0-308"
    betaKO
"GHA0-307"


$BSUB633149
$BSUB633149$`TUHJJ-63`
 epsilonKO     betaKO    gammaKO    alphaKO    deltaKO
"GHJJ-133" "GHJJ-132" "GHJJ-131" "GHJJ-130" "GHJJ-129"
```

$BSUB633149$`TUHJJ-1426`
cKO aKO
"GHJJ-2747" "GHJJ-2746"

$BSUB633149$`TUHJJ-1427`
bKO2 bKO1
"GHJJ-2749" "GHJJ-2748"

$ABAU400667
$ABAU400667$`TUI0Q-124`
epsilonKO betaKO gammaKO alphaKO deltaKO bKO cKO
"GI0Q-149" "GI0Q-148" "GI0Q-147" "GI0Q-146" "GI0Q-145" "GI0Q-144" "GI0Q-143"

$ABAU400667$`TUI0Q-123`
aKO
"GI0Q-141"

$ACAL871585
$ACAL871585$`TUH86-1824`
epsilonKO betaKO gammaKO alphaKO deltaKO bKO
"GH86-3134" "GH86-3133" "GH86-3132" "GH86-3131" "GH86-3130" "GH86-3129"
cKO aKO
"GH86-3128" "GH86-3127"

$APAS634454
$APAS634454$`TUL7B-296`
epsilonKO betaKO gammaKO alphaKO deltaKO
"GL7B-121" "GL7B-120" "GL7B-119" "GL7B-118" "GL7B-117"

$APAS634454$`TUL7B-1513`
aKO cKO
"GL7B-2466" "GL7B-2465"

$APAS634454$`TUL7B-1512`
bKO2 bKO1
"GL7B-2464" "GL7B-2463"

$AOLE436717
$AOLE436717$`TUHCD-2097`
aKO cKO bKO deltaKO alphaKO gammaKO
"GHCD-3781" "GHCD-3779" "GHCD-3778" "GHCD-3777" "GHCD-3776" "GHCD-3775"
betaKO epsilonKO
"GHCD-3774" "GHCD-3773"

$ACEL351607
$ACEL351607$`TUIXW-345`
epsilonKO betaKO gammaKO alphaKO deltaKO bKO cKO
"GIXW-668" "GIXW-667" "GIXW-666" "GIXW-665" "GIXW-664" "GIXW-663" "GIXW-662"
aKO

"GIXW-661"

$`RSOL859656-WGS`
$`RSOL859656-WGS`$`TUST9-1124`
 epsilonKO   betaKO   gammaKO   alphaKO   deltaKO   bKO   cKO
"GST9-146" "GST9-145" "GST9-144" "GST9-143" "GST9-142" "GST9-141" "GST9-140"
       aKO
"GST9-139"

$BSAX1146883
$BSAX1146883$`TUL9E-2065`
        aKO   cKO   bKO   deltaKO   alphaKO   gammaKO
"GL9E-3801" "GL9E-3800" "GL9E-3799" "GL9E-3798" "GL9E-3797" "GL9E-3796"
     betaKO   epsilonKO
"GL9E-3795" "GL9E-3794"

$BSEL439292
$BSEL439292$`TUHLG-1722`
        aKO   cKO   bKO   deltaKO   alphaKO   gammaKO
"GHLG-3247" "GHLG-3246" "GHLG-3245" "GHLG-3244" "GHLG-3243" "GHLG-3242"
     betaKO   epsilonKO
"GHLG-3241" "GHLG-3240"

$`BSUB1147161-WGS`
$`BSUB1147161-WGS`$`TUSG1-2018`
        aKO   cKO   bKO   deltaKO   alphaKO   gammaKO
"GSG1-3900" "GSG1-3899" "GSG1-3898" "GSG1-3897" "GSG1-3896" "GSG1-3895"
     betaKO   epsilonKO
"GSG1-3894" "GSG1-3893"

$`BSUI204722-WGS`
$`BSUI204722-WGS`$`TUSIT-1048`
    deltaKO   alphaKO   gammaKO   betaKO   epsilonKO
"GSIT-1833" "GSIT-1832" "GSIT-1831" "GSIT-1830" "GSIT-1829"

$`BSUI204722-WGS`$`TUSIT-223`
      bKO2   bKO1   cKO   aKO
"GSIT-391" "GSIT-390" "GSIT-389" "GSIT-388"

$BCAN1104321
$BCAN1104321$`TUJV1-834`
  epsilonKO   betaKO   gammaKO   alphaKO   deltaKO
"GJV1-1457" "GJV1-1456" "GJV1-1455" "GJV1-1454" "GJV1-1453"

$BCAN1104321$`TUJV1-405`
       aKO   cKO   bKO2   bKO1
"GJV1-692" "GJV1-691" "GJV1-690" "GJV1-689"

```
$`BSUB1192196-WGS`
$`BSUB1192196-WGS`$`TUSGZ-221`
 epsilonKO     betaKO    gammaKO    alphaKO    deltaKO        bKO        cKO 
"GSGZ-439" "GSGZ-438" "GSGZ-437" "GSGZ-436" "GSGZ-435" "GSGZ-434" "GSGZ-433" 
       aKO 
"GSGZ-432" 


$BSUB936156
$BSUB936156$`TUHCY-966`
        aKO         cKO         bKO     deltaKO     alphaKO     gammaKO 
"GHCY-1895" "GHCY-1894" "GHCY-1893" "GHCY-1892" "GHCY-1891" "GHCY-1890" 
     betaKO   epsilonKO 
"GHCY-1889" "GHCY-1888" 


$`BSUB645657-WGS`
$`BSUB645657-WGS`$`TUSG4-2116`
        aKO         cKO         bKO     deltaKO     alphaKO     gammaKO 
"GSG4-4024" "GSG4-4023" "GSG4-4022" "GSG4-4021" "GSG4-4020" "GSG4-4019" 
     betaKO   epsilonKO 
"GSG4-4018" "GSG4-4017" 


$LSAN714313
$LSAN714313$`TUIWZ-546`
       aKO        cKO        bKO    deltaKO    alphaKO    gammaKO     betaKO 
"GIWZ-991" "GIWZ-990" "GIWZ-989" "GIWZ-988" "GIWZ-987" "GIWZ-986" "GIWZ-985" 
 epsilonKO 
"GIWZ-984" 


$BSUB1220533
$BSUB1220533$`TUL8K-1960`
        cKO         bKO     deltaKO     alphaKO     gammaKO      betaKO 
"GL8K-3798" "GL8K-3797" "GL8K-3796" "GL8K-3795" "GL8K-3794" "GL8K-3793" 
  epsilonKO 
"GL8K-3792" 

$BSUB1220533$noTU
aKO 
 NA 


$SCO
$SCO$`TU1UA-8972`
   betaKO   gammaKO   alphaKO 
"SCO5373" "SCO5372" "SCO5371" 

$SCO$`TU1UA-8971`
  deltaKO       bKO 
"SCO5370" "SCO5369" 
```

SCO`TU1UA-8973`
epsilonKO
"SCO5374"

SCO`TU1UA-8970`
cKO
"SCO5368"

SCO`TU1UA-8969`
aKO
"SCO5367"

$BSUB1052588
$BSUB1052588$`TUL8O-1977`
aKO cKO bKO deltaKO alphaKO gammaKO
"GL8O-3816" "GL8O-3815" "GL8O-3814" "GL8O-3813" "GL8O-3812" "GL8O-3811"
betaKO epsilonKO
"GL8O-3810" "GL8O-3809"

$BSUB655816
$BSUB655816$`TUCOR-1917`
aKO cKO bKO deltaKO alphaKO gammaKO
"GCOR-3716" "GCOR-3715" "GCOR-3714" "GCOR-3713" "GCOR-3712" "GCOR-3711"
betaKO epsilonKO
"GCOR-3710" "GCOR-3709"

$BSUB1052585
$BSUB1052585$`TUJWW-2081`
bKO deltaKO alphaKO gammaKO betaKO epsilonKO
"GJWW-4023" "GJWW-4022" "GJWW-4021" "GJWW-4020" "GJWW-4019" "GJWW-4018"

$BSUB1052585$`TUJWW-2082`
aKO cKO
"GJWW-4026" "GJWW-4025"

$BSUB
$BSUB$`TU8J2-372`
epsilonKO betaKO gammaKO alphaKO deltaKO bKO cKO
"BSU36800" "BSU36810" "BSU36820" "BSU36830" "BSU36840" "BSU36850" "BSU36860"
aKO
"BSU36870"

$BSUI1112912
$BSUI1112912$`TUJTI-1670`
deltaKO alphaKO gammaKO betaKO epsilonKO
"GJTI-1835" "GJTI-1834" "GJTI-1833" "GJTI-1832" "GJTI-1831"

$BSUI1112912$`TUJTI-845`
bKO2 bKO1 cKO aKO

"GJTI-391" "GJTI-390" "GJTI-389" "GJTI-388"

$`BSUB1233100-WGS`
$`BSUB1233100-WGS`$`TUSGN-1925`
cKO bKO deltaKO alphaKO gammaKO betaKO
"GSGN-3526" "GSGN-3525" "GSGN-3524" "GSGN-3523" "GSGN-3522" "GSGN-3521"
epsilonKO
"GSGN-3520"

$`BSUB1233100-WGS`$noTU
aKO
NA

$`BSUB1302650-WGS`
$`BSUB1302650-WGS`$`TUSG2-1918`
aKO cKO bKO deltaKO alphaKO gammaKO
"GSG2-3682" "GSG2-3681" "GSG2-3680" "GSG2-3679" "GSG2-3678" "GSG2-3677"
betaKO epsilonKO
"GSG2-3676" "GSG2-3675"

$BTHU714359
$BTHU714359$`TUJBQ-3228`
aKO cKO bKO deltaKO alphaKO gammaKO
"GJBQ-5056" "GJBQ-5055" "GJBQ-5054" "GJBQ-5053" "GJBQ-5052" "GJBQ-5051"
betaKO epsilonKO
"GJBQ-5050" "GJBQ-5049"

$BTHU541229
$BTHU541229$`TUL8T-3663`
aKO cKO bKO deltaKO alphaKO gammaKO
"GL8T-5471" "GL8T-5470" "GL8T-5469" "GL8T-5468" "GL8T-5467" "GL8T-5466"
betaKO epsilonKO
"GL8T-5465" "GL8T-5464"

$LSOL658172
$LSOL658172$`TUHHM-50`
deltaKO alphaKO gammaKO betaKO epsilonKO
"GHHM-72" "GHHM-71" "GHHM-70" "GHHM-69" "GHHM-68"

$LSOL658172$`TUHHM-441`
cKO aKO
"GHHM-716" "GHHM-715"

$LSOL658172$`TUHHM-442`
bKO2 bKO1
"GHHM-718" "GHHM-717"

$SDEG203122

```
$SDEG203122$`TUI2M-2417`
        aKO         cKO         bKO     deltaKO     alphaKO     gammaKO 
"GI2M-4022" "GI2M-4021" "GI2M-4020" "GI2M-4019" "GI2M-4018" "GI2M-4017" 
     betaKO   epsilonKO 
"GI2M-4016" "GI2M-4015" 


$BTHA271848
$BTHA271848$`TUJMY-3204`
       aKO1        cKO1        bKO1     deltaKO    alphaKO1    gammaKO1 
"GJMY-3314" "GJMY-3313" "GJMY-3312" "GJMY-3311" "GJMY-3310" "GJMY-3309" 
    betaKO1  epsilonKO1 
"GJMY-3308" "GJMY-3307" 

$BTHA271848$`TUJMY-228`
   gammaKO2    alphaKO2        bKO2        cKO2        aKO2  epsilonKO2 
"GJMY-3771" "GJMY-3770" "GJMY-3769" "GJMY-3768" "GJMY-3767" "GJMY-3764" 
    betaKO2 
"GJMY-3763" 


$BTHU930170
$BTHU930170$`TUL8S-3309`
        aKO         cKO         bKO     deltaKO     alphaKO     gammaKO 
"GL8S-5408" "GL8S-5407" "GL8S-5406" "GL8S-5405" "GL8S-5404" "GL8S-5403" 
     betaKO   epsilonKO 
"GL8S-5402" "GL8S-5401" 


$`BTHU527021-WGS`
$`BTHU527021-WGS`$`TUSG7-3685`
        aKO         cKO         bKO     deltaKO     alphaKO     gammaKO 
"GSG7-5505" "GSG7-5504" "GSG7-5503" "GSG7-5502" "GSG7-5501" "GSG7-5500" 
     betaKO   epsilonKO 
"GSG7-5499" "GSG7-5498" 


$BTHE226186
$BTHE226186$`TUJXV-394`
   alphaKO    deltaKO        bKO        cKO        aKO  epsilonKO     betaKO 
"GJXV-726" "GJXV-725" "GJXV-724" "GJXV-723" "GJXV-722" "GJXV-720" "GJXV-719" 

$BTHE226186$`TUJXV-395`
   gammaKO 
"GJXV-727" 


$`BTHU1286404-WGS`
$`BTHU1286404-WGS`$`TUSGR-4009`
        aKO         cKO         bKO     deltaKO     alphaKO     gammaKO 
"GSGR-5555" "GSGR-5554" "GSGR-5553" "GSGR-5552" "GSGR-5551" "GSGR-5550" 
     betaKO   epsilonKO 
"GSGR-5549" "GSGR-5548" 
```

```
$`BTHU529122-WGS`
$`BTHU529122-WGS`$`TUSGD-3906`
       aKO          cKO          bKO      deltaKO      alphaKO      gammaKO
"GSGD-5973" "GSGD-5972" "GSGD-5971" "GSGD-5970" "GSGD-5969" "GSGD-5968"
     betaKO    epsilonKO
"GSGD-5967" "GSGD-5966"


$BTHU1218175
$BTHU1218175$`TUL8R-2928`
  epsilonKO       betaKO      gammaKO      alphaKO      deltaKO          bKO
"GL8R-4380" "GL8R-4379" "GL8R-4378" "GL8R-4377" "GL8R-4376" "GL8R-4375"
       cKO          aKO
"GL8R-4374" "GL8R-4373"


$BTHU281309
$BTHU281309$`TUJID-3004`
       aKO          cKO          bKO      deltaKO      alphaKO
"GJID-5085" "GJID-5084" "GJID-5083" "GJID-5082" "GJID-5081"

$BTHU281309$`TUJID-3003`
    gammaKO       betaKO    epsilonKO
"GJID-5079" "GJID-5078" "GJID-5077"


$BTHU412694
$BTHU412694$`TUH1W-2741`
       aKO          cKO          bKO      deltaKO      alphaKO      gammaKO
"GH1W-4715" "GH1W-4714" "GH1W-4713" "GH1W-4712" "GH1W-4711" "GH1W-4710"
     betaKO    epsilonKO
"GH1W-4709" "GH1W-4708"


$`BTHU1217737-WGS`
$`BTHU1217737-WGS`$`TUSG8-3464`
       aKO          cKO          bKO      deltaKO      alphaKO      gammaKO
"GSG8-4994" "GSG8-4993" "GSG8-4992" "GSG8-4991" "GSG8-4990" "GSG8-4989"
     betaKO    epsilonKO
"GSG8-4988" "GSG8-4987"


$CJEJ567106
$CJEJ567106$`TULA7-72`
 epsilonKO      betaKO     gammaKO     alphaKO     deltaKO        bKO2        bKO1
"GLA7-105" "GLA7-104" "GLA7-103" "GLA7-102" "GLA7-101" "GLA7-100"  "GLA7-99"

$CJEJ567106$`TULA7-351`
      cKO
"GLA7-907"

$CJEJ567106$`TULA7-440`
       aKO
```

"GLA7-1177"

$SGAL637909
$SGAL637909$`TUJOV-870`
gammaKO alphaKO deltaKO
"GJOV-1292" "GJOV-1291" "GJOV-1290"

$SGAL637909$`TUJOV-872`
betaKO
"GJOV-1294"

$SGAL637909$`TUJOV-873`
epsilonKO
"GJOV-1296"

$SGAL637909$`TUJOV-867`
cKO
"GJOV-1284"

$SGAL637909$`TUJOV-868`
aKO
"GJOV-1286"

$SGAL637909$`TUJOV-869`
bKO
"GJOV-1288"

$LSPH444177
$LSPH444177$`TUJEL-685`
epsilonKO betaKO gammaKO alphaKO deltaKO bKO
"GJEL-1065" "GJEL-1064" "GJEL-1063" "GJEL-1062" "GJEL-1061" "GJEL-1060"
cKO aKO
"GJEL-1059" "GJEL-1058"

$`BTRE1171377-WGS`
$`BTRE1171377-WGS`$`TUSGO-6`
epsilonKO betaKO gammaKO alphaKO deltaKO bKO cKO aKO
"GSGO-19" "GSGO-18" "GSGO-17" "GSGO-16" "GSGO-15" "GSGO-14" "GSGO-13" "GSGO-12"

$`BTHE1254439-WGS`
$`BTHE1254439-WGS`$`TUSGX-1008`
aKO cKO bKO deltaKO alphaKO gammaKO
"GSGX-1604" "GSGX-1603" "GSGX-1602" "GSGX-1601" "GSGX-1600" "GSGX-1599"
betaKO epsilonKO
"GSGX-1598" "GSGX-1597"

$BTRI382640
$BTRI382640$`TUJEK-1137`
deltaKO alphaKO gammaKO betaKO epsilonKO

"GJEK-1954" "GJEK-1953" "GJEK-1952" "GJEK-1951" "GJEK-1950"

$BTRI382640$`TUJEK-333`
bKO2 bKO1 cKO aKO
"GJEK-560" "GJEK-559" "GJEK-558" "GJEK-557"

$KTUS562970
$KTUS562970$`TUHUX-1709`
aKO cKO bKO deltaKO alphaKO gammaKO
"GHUX-3312" "GHUX-3311" "GHUX-3310" "GHUX-3309" "GHUX-3308" "GHUX-3307"
betaKO epsilonKO
"GHUX-3306" "GHUX-3305"

$BAPH713603
$BAPH713603$`TUL9U-2`
epsilonKO betaKO gammaKO alphaKO deltaKO bKO cKO aKO
"GL9U-9" "GL9U-8" "GL9U-7" "GL9U-6" "GL9U-5" "GL9U-4" "GL9U-3" "GL9U-2"

$BAPH107806
$BAPH107806$`TUBZJ-6`
epsilonKO betaKO gammaKO alphaKO deltaKO bKO cKO aKO
"GBZJ-9" "GBZJ-8" "GBZJ-7" "GBZJ-6" "GBZJ-5" "GBZJ-4" "GBZJ-3" "GBZJ-2"

$BSP640510
$BSP640510$`TUI28-2173`
aKO cKO bKO deltaKO alphaKO gammaKO
"GI28-3594" "GI28-3593" "GI28-3592" "GI28-3591" "GI28-3590" "GI28-3589"
betaKO epsilonKO
"GI28-3588" "GI28-3587"

$BAPH1005057
$BAPH1005057$`TUL9X-7`
epsilonKO betaKO gammaKO alphaKO deltaKO bKO cKO aKO
"GL9X-9" "GL9X-8" "GL9X-7" "GL9X-6" "GL9X-5" "GL9X-4" "GL9X-3" "GL9X-2"

$SMAL868597
$SMAL868597$`TUHCG-1936`
aKO cKO bKO deltaKO alphaKO gammaKO
"GHCG-3634" "GHCG-3633" "GHCG-3632" "GHCG-3631" "GHCG-3630" "GHCG-3629"
betaKO epsilonKO
"GHCG-3628" "GHCG-3627"

$`SENT220341-WGS`
$`SENT220341-WGS`$`TUSUP-2187`
epsilonKO betaKO gammaKO alphaKO deltaKO bKO
"GSUP-3719" "GSUP-3718" "GSUP-3717" "GSUP-3716" "GSUP-3715" "GSUP-3714"
cKO aKO

```
"GSUP-3713" "GSUP-3712"


$ACHL452863
$ACHL452863$`TUH1A-1661`
        aKO         cKO         bKO      deltaKO     alphaKO     gammaKO
"GH1A-2393" "GH1A-2392" "GH1A-2391" "GH1A-2390" "GH1A-2389" "GH1A-2388"
     betaKO   epsilonKO
"GH1A-2387" "GH1A-2386"


$LXYL281090
$LXYL281090$`TUH0X-382`
 epsilonKO     betaKO    gammaKO    alphaKO    deltaKO        bKO        cKO
"GH0X-655" "GH0X-654" "GH0X-653" "GH0X-652" "GH0X-651" "GH0X-650" "GH0X-649"
       aKO
"GH0X-648"


$ASP62977
$ASP62977$`TUJVV-85`
 epsilonKO     betaKO    gammaKO    alphaKO    deltaKO        bKO        cKO
"GJVV-180" "GJVV-179" "GJVV-178" "GJVV-177" "GJVV-176" "GJVV-175" "GJVV-174"
       aKO
"GJVV-173"


$ASP358220
$ASP358220$`TUL7N-247`
 epsilonKO     betaKO    gammaKO    alphaKO    deltaKO        bKO        cKO
"GL7N-397" "GL7N-396" "GL7N-395" "GL7N-394" "GL7N-393" "GL7N-392" "GL7N-391"
       aKO
"GL7N-390"


$ALAI441768
$ALAI441768$`TUI40-446`
       aKO        cKO        bKO    deltaKO    alphaKO    gammaKO     betaKO
"GI40-988" "GI40-987" "GI40-986" "GI40-985" "GI40-984" "GI40-983" "GI40-982"
 epsilonKO
"GI40-981"


$BSP416344
$BSP416344$`TUL9Z-88`
epsilonKO1     betaKO   gammaKO1    alphaKO    deltaKO        bKO        cKO
"GL9Z-107" "GL9Z-106" "GL9Z-105" "GL9Z-104" "GL9Z-103" "GL9Z-102" "GL9Z-101"
       aKO
"GL9Z-100"

$BSP416344$`TUL9Z-2908`
 epsilonKO2    gammaKO2
"GL9Z-5059" "GL9Z-5058"
```

```
$BAPH713602
$BAPH713602$`TUL9W-2`
epsilonKO    betaKO   gammaKO   alphaKO   deltaKO       bKO       cKO 
 "GL9W-8"  "GL9W-7"  "GL9W-6"  "GL9W-5"  "GL9W-4"  "GL9W-3"  "GL9W-2" 

$BAPH713602$noTU
aKO 
 NA 



$`BLAT482957-WGS`
$`BLAT482957-WGS`$`TUSHG-1890`
 epsilonKO     betaKO    gammaKO    alphaKO    deltaKO        bKO        cKO 
"GSHG-112" "GSHG-111" "GSHG-110" "GSHG-109" "GSHG-108" "GSHG-107" "GSHG-106" 
       aKO 
"GSHG-105" 



$BVAF859654
$BVAF859654$`TUHAB-2`
epsilonKO    betaKO   gammaKO   alphaKO   deltaKO       bKO       cKO       aKO 
 "GHAB-9"  "GHAB-8"  "GHAB-7"  "GHAB-6"  "GHAB-5"  "GHAB-4"  "GHAB-3"  "GHAB-2" 



$BVIE269482
$BVIE269482$`TUJNA-2630`
epsilonKO1     betaKO   gammaKO1    alphaKO    deltaKO        bKO        cKO 
"GJNA-119" "GJNA-118" "GJNA-117" "GJNA-116" "GJNA-115" "GJNA-114" "GJNA-113" 
       aKO 
"GJNA-112" 

$BVIE269482$`TUJNA-2508`
 epsilonKO2    gammaKO2 
"GJNA-5444" "GJNA-5443" 



$SYNEL
$SYNEL$`TU490-3678`
          gammaKO           alphaKO 
"SYNPCC7942_0337" "SYNPCC7942_0336" 

$SYNEL$`TU490-4870`
           betaKO 
"SYNPCC7942_2315" 

$SYNEL$`TU490-3677`
          deltaKO              bKO2 
"SYNPCC7942_0335" "SYNPCC7942_0334" 

$SYNEL$`TU490-4871`
        epsilonKO 
"SYNPCC7942_2316" 
```

```
$SYNEL$`TU490-3675`
            cKO             aKO
"SYNPCC7942_0332" "SYNPCC7942_0331"

$SYNEL$`TU490-3676`
           bKO1
"SYNPCC7942_0333"


$`BVIN1094497-WGS`
$`BVIN1094497-WGS`$`TUSGJ-762`
   deltaKO    alphaKO    gammaKO     betaKO   epsilonKO
"GSGJ-1348" "GSGJ-1347" "GSGJ-1346" "GSGJ-1345" "GSGJ-1344"

$`BVIN1094497-WGS`$`TUSGJ-181`
     bKO2      bKO1       cKO       aKO
"GSGJ-303" "GSGJ-302" "GSGJ-301" "GSGJ-300"


$BVUL435590
$BVUL435590$`TUH96-1587`
   gammaKO    alphaKO    deltaKO        bKO        cKO        aKO
"GH96-2993" "GH96-2992" "GH96-2991" "GH96-2990" "GH96-2989" "GH96-2988"
  epsilonKO     betaKO
"GH96-2986" "GH96-2985"


$`LBRE1001583-WGS`
$`LBRE1001583-WGS`$`TUSPD-561`
 epsilonKO    betaKO    gammaKO    alphaKO    deltaKO       bKO       cKO
"GSPD-788" "GSPD-787" "GSPD-786" "GSPD-785" "GSPD-784" "GSPD-783" "GSPD-782"
      aKO
"GSPD-781"


$`LXYL1389489-WGS`
$`LXYL1389489-WGS`$`TUSPL-938`
       aKO        cKO        bKO    deltaKO     alphaKO     gammaKO
"GSPL-1769" "GSPL-1768" "GSPL-1767" "GSPL-1766" "GSPL-1765" "GSPL-1764"
    betaKO   epsilonKO
"GSPL-1763" "GSPL-1762"


$BWEI315730
$BWEI315730$`TUHRU-3409`
       aKO        cKO        bKO    deltaKO     alphaKO     gammaKO
"GHRU-5253" "GHRU-5252" "GHRU-5251" "GHRU-5250" "GHRU-5249" "GHRU-5248"
    betaKO   epsilonKO
"GHRU-5247" "GHRU-5246"


$BXEN266265
$BXEN266265$`TUJII-2660`
      aKO1       cKO1       bKO1     deltaKO    alphaKO1    gammaKO1
```

"GJII-4532" "GJII-4531" "GJII-4530" "GJII-4529" "GJII-4528" "GJII-4527"
betaKO1 epsilonKO1
"GJII-4526" "GJII-4525"

$BXEN266265$`TUJII-1576`
betaKO2 epsilonKO2 aKO2 cKO2 bKO2 alphaKO2
"GJII-2653" "GJII-2652" "GJII-2650" "GJII-2649" "GJII-2648" "GJII-2647"
gammaKO2
"GJII-2646"

$BXEN266265$`TUJII-4469`
aKO3 cKO3 bKO3 alphaKO3 gammaKO3
"GJII-7701" "GJII-7700" "GJII-7699" "GJII-7698" "GJII-7697"

$BXEN266265$`TUJII-4470`
betaKO3 epsilonKO3
"GJII-7704" "GJII-7703"

$BAMY1034836
$BAMY1034836$`TUL8J-2006`
aKO cKO bKO deltaKO alphaKO gammaKO
"GL8J-3870" "GL8J-3869" "GL8J-3868" "GL8J-3867" "GL8J-3866" "GL8J-3865"
betaKO epsilonKO
"GL8J-3864" "GL8J-3863"

$`BXYL657309-WGS`
$`BXYL657309-WGS`$`TUSGH-128`
gammaKO alphaKO deltaKO bKO cKO aKO epsilonKO
"GSGH-240" "GSGH-239" "GSGH-238" "GSGH-237" "GSGH-236" "GSGH-235" "GSGH-233"
betaKO
"GSGH-232"

$BAMY1155777
$BAMY1155777$`TUL8E-1998`
aKO cKO bKO deltaKO alphaKO gammaKO
"GL8E-3708" "GL8E-3707" "GL8E-3706" "GL8E-3705" "GL8E-3704" "GL8E-3703"
betaKO epsilonKO
"GL8E-3702" "GL8E-3701"

$BSP1097668
$BSP1097668$`TUKEO-2669`
aKO cKO bKO deltaKO alphaKO gammaKO
"GKEO-2723" "GKEO-2722" "GKEO-2721" "GKEO-2720" "GKEO-2719" "GKEO-2718"
betaKO epsilonKO
"GKEO-2717" "GKEO-2716"

$TMAR243274
$TMAR243274$`TUC6P-511`
aKO cKO bKO deltaKO alphaKO gammaKO

```
"GC6P-1662" "GC6P-1661" "GC6P-1660" "GC6P-1659" "GC6P-1658" "GC6P-1657"
     betaKO   epsilonKO
"GC6P-1656" "GC6P-1655"


$CAKA583355
$CAKA583355$`TUI4D-1213`
  epsilonKO      betaKO     gammaKO     alphaKO     deltaKO         bKO
"GI4D-2152" "GI4D-2151" "GI4D-2150" "GI4D-2149" "GI4D-2148" "GI4D-2147"
        cKO         aKO
"GI4D-2146" "GI4D-2145"


$CACE272562
$CACE272562$`TUJIH-1683`
        bKO     deltaKO     alphaKO     gammaKO      betaKO   epsilonKO
"GJIH-2952" "GJIH-2951" "GJIH-2950" "GJIH-2949" "GJIH-2948" "GJIH-2947"

$CACE272562$`TUJIH-1684`
        aKO         cKO
"GJIH-2954" "GJIH-2953"


$CACI1128398
$CACI1128398$`TULBC-124`
 epsilonKO     betaKO    gammaKO    alphaKO    deltaKO        bKO        cKO
"GLBC-206" "GLBC-205" "GLBC-204" "GLBC-203" "GLBC-202" "GLBC-201" "GLBC-200"
       aKO
"GLBC-199"


$CACE991791
$CACE991791$`TUIVN-1700`
        bKO     deltaKO     alphaKO     gammaKO      betaKO   epsilonKO
"GIVN-2975" "GIVN-2974" "GIVN-2973" "GIVN-2972" "GIVN-2971" "GIVN-2970"

$CACE991791$`TUIVN-1701`
        cKO
"GIVN-2976"

$CACE991791$`TUIVN-1702`
        aKO
"GIVN-2977"


$`MABS1303024-WGS`
$`MABS1303024-WGS`$`TUSQN-667`
  epsilonKO      betaKO     gammaKO     alphaKO         bKO         cKO
"GSQN-1467" "GSQN-1466" "GSQN-1465" "GSQN-1464" "GSQN-1462" "GSQN-1461"
        aKO
"GSQN-1460"

$`MABS1303024-WGS`$noTU
deltaKO
```

```
    NA


$CAGG326427
$CAGG326427$`TUHS8-580`
      aKO         cKO         bKO     deltaKO     alphaKO     gammaKO 
"GHS8-1001" "GHS8-1000"  "GHS8-999"  "GHS8-998"  "GHS8-997"  "GHS8-996" 
    betaKO   epsilonKO 
 "GHS8-995"  "GHS8-994" 


$`CAUT1341692-WGS`
$`CAUT1341692-WGS`$`TUSLA-1385`
  epsilonKO      betaKO     gammaKO     alphaKO     deltaKO         bKO 
"GSLA-2415" "GSLA-2414" "GSLA-2413" "GSLA-2412" "GSLA-2411" "GSLA-2410" 
        cKO         aKO 
"GSLA-2409" "GSLA-2408" 


$CACI479433
$CACI479433$`TUI6Z-696`
  epsilonKO      betaKO     gammaKO     alphaKO     deltaKO         bKO 
"GI6Z-1231" "GI6Z-1230" "GI6Z-1229" "GI6Z-1228" "GI6Z-1227" "GI6Z-1226" 
        cKO         aKO 
"GI6Z-1225" "GI6Z-1224" 


$CSP366602
$CSP366602$`TUH0Y-2958`
    deltaKO     alphaKO     gammaKO      betaKO   epsilonKO 
"GH0Y-4793" "GH0Y-4792" "GH0Y-4790" "GH0Y-4789" "GH0Y-4787" 

$CSP366602$`TUH0Y-2737`
       bKO2        bKO1         cKO         aKO 
"GH0Y-4430" "GH0Y-4429" "GH0Y-4428" "GH0Y-4427" 


$CSP99598
$CSP99598$`TULA8-339`
   gammaKO    alphaKO    deltaKO       bKO2 
"GLA8-518" "GLA8-517" "GLA8-516" "GLA8-515" 

$CSP99598$`TULA8-1836`
  epsilonKO      betaKO 
"GLA8-2664" "GLA8-2663" 

$CSP99598$`TULA8-337`
       cKO        aKO 
"GLA8-513" "GLA8-512" 

$CSP99598$`TULA8-338`
      bKO1 
"GLA8-514" 
```

```
$`TNIT1255043-WGS`
$`TNIT1255043-WGS`$`TUSYW-2060`
  epsilonKO      betaKO     gammaKO     alphaKO     deltaKO         bKO
"GSYW-3726" "GSYW-3725" "GSYW-3724" "GSYW-3723" "GSYW-3722" "GSYW-3721"
        cKO         aKO
"GSYW-3720" "GSYW-3719"


$CPAR1170562
$CPAR1170562$`TULA1-1205`
    gammaKO     alphaKO     deltaKO        bKO2
"GLA1-1585" "GLA1-1584" "GLA1-1583" "GLA1-1582"

$CPAR1170562$`TULA1-830`
     betaKO   epsilonKO
"GLA1-1047" "GLA1-1046"

$CPAR1170562$`TULA1-1203`
        cKO         aKO
"GLA1-1580" "GLA1-1579"

$CPAR1170562$`TULA1-1204`
       bKO1
"GLA1-1581"


$CAPO755178
$CAPO755178$`TULC6-593`
      bKO1    deltaKO    alphaKO    gammaKO
"GLC6-788" "GLC6-787" "GLC6-786" "GLC6-785"

$CAPO755178$`TULC6-2439`
     betaKO   epsilonKO
"GLC6-3374" "GLC6-3373"

$CAPO755178$`TULC6-595`
       aKO        cKO
"GLC6-791" "GLC6-790"

$CAPO755178$`TULC6-594`
      bKO2
"GLC6-789"


$CALG688270
$CALG688270$`TUHJ1-902`
       aKO1        cKO1        bKO1     deltaKO    alphaKO1    gammaKO1
"GHJ1-1567" "GHJ1-1566" "GHJ1-1565" "GHJ1-1564" "GHJ1-1563" "GHJ1-1562"

$CALG688270$`TUHJ1-1678`
   gammaKO2    alphaKO2        bKO2        cKO2        aKO2  epsilonKO2
"GHJ1-3027" "GHJ1-3026" "GHJ1-3025" "GHJ1-3024" "GHJ1-3023" "GHJ1-3020"
    betaKO2
```

"GHJ1-3019"

$CALG688270$`TUHJ1-670`
betaKO1
"GHJ1-1173"

$CALG688270$`TUHJ1-669`
epsilonKO1
"GHJ1-1172"

$CAER926550
$CAER926550$`TULA5-318`
aKO cKO bKO deltaKO alphaKO gammaKO betaKO
"GLA5-519" "GLA5-518" "GLA5-517" "GLA5-516" "GLA5-515" "GLA5-514" "GLA5-513"
epsilonKO
"GLA5-512"

$CAUR548476
$CAUR548476$`TUH9E-595`
epsilonKO betaKO gammaKO alphaKO deltaKO bKO
"GH9E-1098" "GH9E-1097" "GH9E-1096" "GH9E-1095" "GH9E-1094" "GH9E-1093"
cKO aKO
"GH9E-1092" "GH9E-1091"

$MACE188937
$MACE188937$`TUI2O-1599`
betaKO epsilonKO aKO cKO bKO alphaKO
"GI2O-2463" "GI2O-2462" "GI2O-2459" "GI2O-2458" "GI2O-2457" "GI2O-2456"
gammaKO
"GI2O-2455"

$MACE188937$noTU
deltaKO
NA

$CATL216432
$CATL216432$`TUHTE-341`
gammaKO alphaKO deltaKO bKO cKO aKO
"GHTE-624" "GHTE-623" "GHTE-622" "GHTE-621" "GHTE-620" "GHTE-619"

$CATL216432$`TUHTE-160`
betaKO epsilonKO
"GHTE-291" "GHTE-290"

$CAUR324602
$CAUR324602$`TUIXU-1729`
aKO cKO bKO deltaKO alphaKO gammaKO
"GIXU-3096" "GIXU-3095" "GIXU-3094" "GIXU-3093" "GIXU-3092" "GIXU-3091"
betaKO epsilonKO

```
"GIXU-3090" "GIXU-3089"


$CACE863638
$CACE863638$`TULBA-1695`
       bKO     deltaKO     alphaKO     gammaKO      betaKO   epsilonKO
"GLBA-2952" "GLBA-2951" "GLBA-2950" "GLBA-2949" "GLBA-2948" "GLBA-2947"

$CACE863638$`TULBA-1696`
       aKO         cKO
"GLBA-2954" "GLBA-2953"


$`CARG1348662-WGS`
$`CARG1348662-WGS`$`TUSLS-642`
       cKO         bKO     deltaKO     alphaKO     gammaKO      betaKO
"GSLS-1284" "GSLS-1283" "GSLS-1282" "GSLS-1281" "GSLS-1280" "GSLS-1279"
  epsilonKO
"GSLS-1278"

$`CARG1348662-WGS`$`TUSLS-643`
       aKO
"GSLS-1285"


$CSUB273068
$CSUB273068$`TUJEB-853`
    alphaKO
"GJEB-1151"

$CSUB273068$`TUJEB-855`
     betaKO
"GJEB-1155"

$CSUB273068$`TUJEB-854`
    gammaKO
"GJEB-1153"

$CSUB273068$`TUJEB-852`
    deltaKO
"GJEB-1149"

$CSUB273068$`TUJEB-856`
  epsilonKO
"GJEB-1157"

$CSUB273068$`TUJEB-849`
       cKO
"GJEB-1145"

$CSUB273068$`TUJEB-847`
       aKO
"GJEB-1143"
```

$CSUB273068$`TUJEB-850`
bKO
"GJEB-1147"

$CBOT441770
$CBOT441770$`TUH1E-96`
epsilonKO betaKO gammaKO alphaKO deltaKO bKO cKO
"GH1E-183" "GH1E-182" "GH1E-181" "GH1E-180" "GH1E-179" "GH1E-178" "GH1E-177"
aKO
"GH1E-176"

$CBOT498213
$CBOT498213$`TUCNI-244`
epsilonKO betaKO gammaKO alphaKO deltaKO bKO cKO
"GCNI-192" "GCNI-191" "GCNI-190" "GCNI-189" "GCNI-188" "GCNI-187" "GCNI-186"
aKO
"GCNI-185"

$CBUR434924
$CBUR434924$`TUHWU-49`
epsilonKO betaKO gammaKO alphaKO deltaKO bKO cKO aKO
"GHWU-54" "GHWU-53" "GHWU-52" "GHWU-51" "GHWU-50" "GHWU-49" "GHWU-48" "GHWU-47"

$CBUR434922
$CBUR434922$`TUJTP-136`
aKO cKO bKO deltaKO alphaKO gammaKO betaKO
"GJTP-196" "GJTP-195" "GJTP-194" "GJTP-193" "GJTP-192" "GJTP-191" "GJTP-190"
epsilonKO
"GJTP-189"

$CBEI290402
$CBEI290402$`TUHL5-263`
epsilonKO betaKO gammaKO alphaKO deltaKO bKO cKO
"GHL5-475" "GHL5-474" "GHL5-473" "GHL5-472" "GHL5-471" "GHL5-470" "GHL5-469"
aKO
"GHL5-468"

$CBOT441772
$CBOT441772$`TUJIE-110`
epsilonKO betaKO gammaKO alphaKO deltaKO bKO cKO
"GJIE-193" "GJIE-192" "GJIE-191" "GJIE-190" "GJIE-189" "GJIE-188" "GJIE-187"
aKO
"GJIE-186"

$MADH225937
$MADH225937$`TULG5-2083`
aKO1 cKO1 bKO1 deltaKO alphaKO1 gammaKO1

"GLG5-3743" "GLG5-3742" "GLG5-3741" "GLG5-3740" "GLG5-3739" "GLG5-3738"
betaKO1 epsilonKO1
"GLG5-3737" "GLG5-3736"

$MADH225937$`TULG5-2255`
gammaKO2 alphaKO2 bKO2 cKO2 aKO2 epsilonKO2
"GLG5-4058" "GLG5-4057" "GLG5-4056" "GLG5-4055" "GLG5-4054" "GLG5-4051"
betaKO2
"GLG5-4050"

$CBUR434923
$CBUR434923$`TUC8S-25`
epsilonKO betaKO gammaKO alphaKO deltaKO bKO cKO aKO
"GC8S-57" "GC8S-56" "GC8S-55" "GC8S-54" "GC8S-53" "GC8S-52" "GC8S-51" "GC8S-50"

$CBOT441771
$CBOT441771$`TUIWX-98`
epsilonKO betaKO gammaKO alphaKO deltaKO bKO cKO
"GIWX-185" "GIWX-184" "GIWX-183" "GIWX-182" "GIWX-181" "GIWX-180" "GIWX-179"
aKO
"GIWX-178"

$CBOT515621
$CBOT515621$`TUCP3-307`
epsilonKO betaKO gammaKO alphaKO deltaKO bKO cKO
"GCP3-190" "GCP3-189" "GCP3-188" "GCP3-187" "GCP3-186" "GCP3-185" "GCP3-184"
aKO
"GCP3-183"

$`MALC1091494-WGS`
$`MALC1091494-WGS`$`TUSQK-190`
epsilonKO1 betaKO1 gammaKO1 alphaKO1 deltaKO bKO1 cKO1
"GSQK-340" "GSQK-339" "GSQK-338" "GSQK-337" "GSQK-336" "GSQK-335" "GSQK-334"
aKO1
"GSQK-333"

$`MALC1091494-WGS`$`TUSQK-2073`
gammaKO2 alphaKO2 bKO2 cKO2 aKO2 epsilonKO2
"GSQK-3767" "GSQK-3766" "GSQK-3765" "GSQK-3764" "GSQK-3763" "GSQK-3760"
betaKO2
"GSQK-3759"

$ADEH455488
$ADEH455488$`TUH35-2350`
deltaKO alphaKO gammaKO betaKO epsilonKO
"GH35-4562" "GH35-4561" "GH35-4560" "GH35-4559" "GH35-4558"

$ADEH455488$`TUH35-2348`
aKO cKO bKO

"GH35-4551" "GH35-4550" "GH35-4549"

$ACRY349163
$ACRY349163$`TUHET-1088`
  epsilonKO   betaKO   gammaKO   alphaKO   deltaKO
"GHET-1706" "GHET-1705" "GHET-1704" "GHET-1703" "GHET-1702"

$ACRY349163$`TUHET-507`
       aKO   cKO   bKO2   bKO1
"GHET-404" "GHET-403" "GHET-402" "GHET-401"

$ACAL990288
$ACAL990288$`TUJBS-188`
epsilonKO1   betaKO   gammaKO   alphaKO   deltaKO   bKO   cKO
 "GJBS-34" "GJBS-33" "GJBS-32" "GJBS-31" "GJBS-30" "GJBS-29" "GJBS-28"
       aKO
 "GJBS-27"

$ACAL990288$`TUJBS-1021`
 epsilonKO2
"GJBS-1721"

$CBOT941968
$CBOT941968$`TULBI-97`
 epsilonKO   betaKO   gammaKO   alphaKO   deltaKO   bKO   cKO
"GLBI-181" "GLBI-180" "GLBI-179" "GLBI-178" "GLBI-177" "GLBI-176" "GLBI-175"
       aKO
"GLBI-174"

$CBOT508765
$CBOT508765$`TUJ4H-305`
 epsilonKO   betaKO   gammaKO   alphaKO   deltaKO   bKO   cKO
"GJ4H-496" "GJ4H-495" "GJ4H-494" "GJ4H-493" "GJ4H-492" "GJ4H-491" "GJ4H-490"
       aKO
"GJ4H-489"

$CBOT498214
$CBOT498214$`TUH05-348`
 epsilonKO   betaKO   gammaKO   alphaKO   deltaKO   bKO   cKO
"GH05-184" "GH05-183" "GH05-182" "GH05-181" "GH05-180" "GH05-179" "GH05-178"
       aKO
"GH05-177"

$CBOT758678
$CBOT758678$`TULBH-123`
 epsilonKO   betaKO   gammaKO   alphaKO   deltaKO   bKO   cKO
"GLBH-185" "GLBH-184" "GLBH-183" "GLBH-182" "GLBH-181" "GLBH-180" "GLBH-179"
       aKO

```
"GLBH-178"



$MAFR572418
$MAFR572418$`TUJCK-708`
  epsilonKO      betaKO     gammaKO     alphaKO        bKO         cKO
"GJCK-1346" "GJCK-1345" "GJCK-1344" "GJCK-1343" "GJCK-1341" "GJCK-1340"
        aKO
"GJCK-1339"

$MAFR572418$noTU
deltaKO
     NA



$CBOT929506
$CBOT929506$`TUHKW-1640`
        aKO         cKO         bKO     deltaKO     alphaKO     gammaKO
"GHKW-2334" "GHKW-2333" "GHKW-2332" "GHKW-2331" "GHKW-2330" "GHKW-2329"
     betaKO   epsilonKO
"GHKW-2328" "GHKW-2327"



$CBUR360115
$CBUR360115$`TUI0X-1121`
  epsilonKO      betaKO     gammaKO     alphaKO     deltaKO         bKO
"GI0X-2156" "GI0X-2154" "GI0X-2153" "GI0X-2152" "GI0X-2151" "GI0X-2150"
        cKO         aKO
"GI0X-2149" "GI0X-2148"



$VCHO
$VCHO$`TU4-10452`
      aKO       cKO       bKO   deltaKO   alphaKO   gammaKO    betaKO epsilonKO
 "VC2770"  "VC2769"  "VC2768"  "VC2767"  "VC2766"  "VC2765"  "VC2764"  "VC2763"



$CBOT508767
$CBOT508767$`TUHKO-274`
 epsilonKO     betaKO    gammaKO    alphaKO    deltaKO        bKO        cKO
"GHKO-488" "GHKO-487" "GHKO-486" "GHKO-485" "GHKO-484" "GHKO-483" "GHKO-482"
       aKO
"GHKO-481"



$CBUR227377
$CBUR227377$`TUJ7S-943`
  epsilonKO      betaKO     gammaKO     alphaKO     deltaKO         bKO
"GJ7S-1920" "GJ7S-1919" "GJ7S-1918" "GJ7S-1917" "GJ7S-1916" "GJ7S-1915"
        cKO         aKO
"GJ7S-1914" "GJ7S-1913"



$`CBAC946483-WGS`
```

```
$`CBAC946483-WGS`$`TUSLO-956`
    alphaKO
"GSLO-1732"

$`CBAC946483-WGS`$`TUSLO-958`
  epsilonKO      betaKO
"GSLO-1735" "GSLO-1734"

$`CBAC946483-WGS`$`TUSLO-957`
    gammaKO
"GSLO-1733"

$`CBAC946483-WGS`$`TUSLO-955`
    deltaKO         bKO
"GSLO-1731" "GSLO-1730"

$`CBAC946483-WGS`$`TUSLO-954`
        cKO
"GSLO-1729"

$`CBAC946483-WGS`$`TUSLO-953`
        aKO
"GSLO-1728"


$CBOT536232
$CBOT536232$`TUCO3-109`
 epsilonKO     betaKO    gammaKO    alphaKO    deltaKO        bKO        cKO
"GCO3-190" "GCO3-189" "GCO3-188" "GCO3-187" "GCO3-186" "GCO3-185" "GCO3-184"
       aKO
"GCO3-183"


$CCEL573061
$CCEL573061$`TUIXD-1895`
        aKO         cKO         bKO     deltaKO     alphaKO     gammaKO
"GIXD-3142" "GIXD-3141" "GIXD-3140" "GIXD-3139" "GIXD-3138" "GIXD-3137"
     betaKO   epsilonKO
"GIXD-3136" "GIXD-3135"


$CCEL394503
$CCEL394503$`TUJET-164`
 epsilonKO     betaKO    gammaKO    alphaKO    deltaKO        bKO        cKO
"GJET-279" "GJET-278" "GJET-277" "GJET-276" "GJET-275" "GJET-274" "GJET-273"
       aKO
"GJET-272"


$CCHL340177
$CCHL340177$`TUHBW-79`
   alphaKO
"GHBW-146"
```

```
$CCHL340177$`TUHBW-1202`
  epsilonKO      betaKO
"GHBW-2029" "GHBW-2028"

$CCHL340177$`TUHBW-80`
   gammaKO
"GHBW-147"

$CCHL340177$`TUHBW-35`
  deltaKO       bKO
"GHBW-68" "GHBW-67"

$CCHL340177$`TUHBW-34`
      cKO
"GHBW-66"

$CCHL340177$`TUHBW-33`
      aKO
"GHBW-65"


$CCLA720554
$CCLA720554$`TUI2T-183`
 epsilonKO     betaKO    gammaKO    alphaKO    deltaKO        bKO        cKO
"GI2T-302" "GI2T-301" "GI2T-300" "GI2T-299" "GI2T-298" "GI2T-297" "GI2T-296"
       aKO
"GI2T-295"


$MMAG342108
$MMAG342108$`TUJNU-1940`
    deltaKO     alphaKO     gammaKO      betaKO   epsilonKO
"GJNU-4191" "GJNU-4190" "GJNU-4189" "GJNU-4188" "GJNU-4187"

$MMAG342108$`TUJNU-1867`
       bKO2        bKO1         cKO         aKO
"GJNU-4044" "GJNU-4043" "GJNU-4042" "GJNU-4041"


$CCAN860228
$CCAN860228$`TUH4O-115`
   gammaKO    alphaKO
"GH4O-193" "GH4O-192"

$CCAN860228$`TUH4O-62`
 epsilonKO     betaKO
"GH4O-113" "GH4O-112"

$CCAN860228$`TUH4O-114`
   deltaKO        bKO
"GH4O-191" "GH4O-190"

$CCAN860228$`TUH4O-113`
       cKO
```

"GH4O-189"

$CCAN860228$`TUH4O-112`
aKO
"GH4O-188"

$`CDIV1394710-WGS`
$`CDIV1394710-WGS`$`TUSI0-258`
alphaKO gammaKO betaKO epsilonKO
"GSI0-565" "GSI0-564" "GSI0-563" "GSI0-562"

$`CDIV1394710-WGS`$noTU
deltaKO cKO aKO bKO
NA NA NA NA

$`CCAL1121353-WGS`
$`CCAL1121353-WGS`$`TUSLT-668`
epsilonKO betaKO gammaKO alphaKO deltaKO bKO
"GSLT-1141" "GSLT-1140" "GSLT-1139" "GSLT-1138" "GSLT-1137" "GSLT-1136"
cKO aKO
"GSLT-1135" "GSLT-1134"

$`CCOL1367491-WGS`
$`CCOL1367491-WGS`$`TUSIV-111`
bKO2 bKO1 deltaKO alphaKO gammaKO betaKO epsilonKO
"GSIV-305" "GSIV-304" "GSIV-303" "GSIV-302" "GSIV-301" "GSIV-300" "GSIV-299"

$`CCOL1367491-WGS`$`TUSIV-433`
cKO
"GSIV-1128"

$`CCOL1367491-WGS`$`TUSIV-347`
aKO
"GSIV-908"

$`CCAT717962-WGS`
$`CCAT717962-WGS`$`TUSLR-362`
epsilonKO1 betaKO1 gammaKO1 alphaKO deltaKO1 bKO1 cKO1
"GSLR-638" "GSLR-637" "GSLR-636" "GSLR-635" "GSLR-634" "GSLR-633" "GSLR-632"
aKO1
"GSLR-631"

$`CCAT717962-WGS`$`TUSLR-659`
aKO2 cKO2 bKO2 deltaKO2 gammaKO2 betaKO2
"GSLR-1164" "GSLR-1163" "GSLR-1162" "GSLR-1161" "GSLR-1160" "GSLR-1159"
epsilonKO2
"GSLR-1158"

$CCUR469378

```
$CCUR469378$`TUH4Z-620`
        cKO         bKO     deltaKO     alphaKO     gammaKO      betaKO 
"GH4Z-1212" "GH4Z-1211" "GH4Z-1210" "GH4Z-1209" "GH4Z-1208" "GH4Z-1207" 
  epsilonKO 
"GH4Z-1206" 

$CCUR469378$`TUH4Z-621`
        aKO 
"GH4Z-1213" 


$CCOR1144275
$CCOR1144275$`TULBO-214`
   alphaKO 
"GLBO-379" 

$CCOR1144275$`TULBO-4098`
    gammaKO      betaKO   epsilonKO 
"GLBO-7507" "GLBO-7506" "GLBO-7504" 

$CCOR1144275$`TULBO-216`
   deltaKO 
"GLBO-381" 

$CCOR1144275$`TULBO-4149`
        aKO         cKO         bKO 
"GLBO-7607" "GLBO-7606" "GLBO-7605" 


$`CCAL1303518-WGS`
$`CCAL1303518-WGS`$`TUSL8-246`
   gammaKO    alphaKO    deltaKO       bKO2       bKO1        cKO        aKO 
"GSL8-519" "GSL8-517" "GSL8-516" "GSL8-515" "GSL8-514" "GSL8-513" "GSL8-512" 

$`CCAL1303518-WGS`$`TUSL8-279`
 epsilonKO     betaKO 
"GSL8-588" "GSL8-587" 


$CDIP698970
$CDIP698970$`TUHZI-491`
 epsilonKO     betaKO    gammaKO    alphaKO    deltaKO        bKO        cKO 
"GHZI-993" "GHZI-992" "GHZI-991" "GHZI-990" "GHZI-989" "GHZI-988" "GHZI-987" 
       aKO 
"GHZI-986" 


$CDIP698973
$CDIP698973$`TUHAI-517`
  epsilonKO      betaKO     gammaKO     alphaKO     deltaKO         bKO 
"GHAI-1053" "GHAI-1052" "GHAI-1051" "GHAI-1050" "GHAI-1049" "GHAI-1048" 
        cKO         aKO 
"GHAI-1047" "GHAI-1046" 
```

```
$CDIF645462
$CDIF645462$`TUJED-1937`
        bKO     deltaKO     alphaKO     gammaKO      betaKO   epsilonKO
"GJED-3356" "GJED-3355" "GJED-3354" "GJED-3353" "GJED-3352" "GJED-3351"

$CDIF645462$`TUJED-1938`
        aKO         cKO
"GJED-3358" "GJED-3357"


$MAER856793
$MAER856793$`TUJBW-375`
 epsilonKO     betaKO    gammaKO    alphaKO    deltaKO
"GJBW-679" "GJBW-677" "GJBW-675" "GJBW-674" "GJBW-673"

$MAER856793$`TUJBW-652`
        aKO         cKO        bKO2        bKO1
"GJBW-1196" "GJBW-1195" "GJBW-1194" "GJBW-1193"


$ABAU696749
$ABAU696749$`TUL7T-178`
   alphaKO
"GL7T-192"

$ABAU696749$`TUL7T-179`
 epsilonKO     betaKO    gammaKO
"GL7T-195" "GL7T-194" "GL7T-193"

$ABAU696749$`TUL7T-177`
   deltaKO        bKO        cKO
"GL7T-191" "GL7T-190" "GL7T-189"

$ABAU696749$`TUL7T-176`
       aKO
"GL7T-188"


$CDIP698965
$CDIP698965$`TUHJP-484`
 epsilonKO     betaKO    gammaKO    alphaKO    deltaKO        bKO        cKO
"GHJP-980" "GHJP-979" "GHJP-978" "GHJP-977" "GHJP-976" "GHJP-975" "GHJP-974"
       aKO
"GHJP-973"


$CDIP698968
$CDIP698968$`TUI5N-485`
 epsilonKO     betaKO    gammaKO    alphaKO    deltaKO        bKO        cKO
"GI5N-983" "GI5N-982" "GI5N-981" "GI5N-980" "GI5N-979" "GI5N-978" "GI5N-977"
       aKO
"GI5N-976"
```

```
$CDIF272563
$CDIF272563$`TUJFE-2114`
      bKO     deltaKO     alphaKO     gammaKO      betaKO   epsilonKO
"GJFE-3730" "GJFE-3729" "GJFE-3728" "GJFE-3727" "GJFE-3726" "GJFE-3725"

$CDIF272563$`TUJFE-2115`
      aKO         cKO
"GJFE-3732" "GJFE-3731"


$`CDIF699034-WGS`
$`CDIF699034-WGS`$`TUSMS-1973`
      aKO         cKO         bKO     deltaKO     alphaKO     gammaKO
"GSMS-3441" "GSMS-3440" "GSMS-3439" "GSMS-3438" "GSMS-3437" "GSMS-3436"
    betaKO   epsilonKO
"GSMS-3435" "GSMS-3434"


$CDIP698972
$CDIP698972$`TUI1X-467`
 epsilonKO     betaKO    gammaKO    alphaKO    deltaKO        bKO        cKO
"GI1X-950" "GI1X-949" "GI1X-948" "GI1X-947" "GI1X-946" "GI1X-945" "GI1X-944"
      aKO
"GI1X-943"


$`CDIP257309-WGS`
$`CDIP257309-WGS`$`TUSLV-503`
  epsilonKO      betaKO     gammaKO     alphaKO     deltaKO         bKO
"GSLV-1052" "GSLV-1051" "GSLV-1050" "GSLV-1049" "GSLV-1048" "GSLV-1047"
      cKO         aKO
"GSLV-1046" "GSLV-1045"


$CDIF645463
$CDIF645463$`TUJP4-1974`
      aKO         cKO         bKO     deltaKO     alphaKO     gammaKO
"GJP4-3394" "GJP4-3393" "GJP4-3392" "GJP4-3391" "GJP4-3390" "GJP4-3389"
    betaKO   epsilonKO
"GJP4-3388" "GJP4-3387"


$CDIP698966
$CDIP698966$`TUHE3-487`
 epsilonKO     betaKO    gammaKO    alphaKO    deltaKO        bKO        cKO
"GHE3-986" "GHE3-985" "GHE3-984" "GHE3-983" "GHE3-982" "GHE3-981" "GHE3-980"
      aKO
"GHE3-979"


$CDIP698969
$CDIP698969$`TUH8A-477`
 epsilonKO     betaKO    gammaKO    alphaKO    deltaKO        bKO        cKO
```

"GH8A-982" "GH8A-981" "GH8A-980" "GH8A-979" "GH8A-978" "GH8A-977" "GH8A-976"
aKO
"GH8A-975"

$CDIP698963
$CDIP698963$`TUHGE-476`
epsilonKO betaKO gammaKO alphaKO deltaKO bKO cKO
"GHGE-996" "GHGE-995" "GHGE-994" "GHGE-993" "GHGE-992" "GHGE-991" "GHGE-990"
aKO
"GHGE-989"

$CJEJ407148
$CJEJ407148$`TUHCS-48`
epsilonKO betaKO gammaKO alphaKO deltaKO bKO2 bKO1
"GHCS-105" "GHCS-104" "GHCS-103" "GHCS-102" "GHCS-101" "GHCS-100" "GHCS-99"

$CJEJ407148$`TUHCS-338`
cKO
"GHCS-899"

$CJEJ407148$`TUHCS-435`
aKO
"GHCS-1182"

$BPEN291272
$BPEN291272$`TUJ9N-7`
alphaKO deltaKO
"GJ9N-9" "GJ9N-8"

$BPEN291272$`TUJ9N-8`
betaKO gammaKO
"GJ9N-12" "GJ9N-11"

$BPEN291272$`TUJ9N-9`
epsilonKO
"GJ9N-14"

$BPEN291272$`TUJ9N-4`
cKO
"GJ9N-4"

$BPEN291272$`TUJ9N-3`
aKO
"GJ9N-2"

$BPEN291272$`TUJ9N-6`
bKO
"GJ9N-6"

$`MAUS754035-WGS`

$`MAUS754035-WGS`$`TUSQR-692`
epsilonKO betaKO gammaKO alphaKO deltaKO
"GSQR-1228" "GSQR-1227" "GSQR-1226" "GSQR-1225" "GSQR-1224"

$`MAUS754035-WGS`$`TUSQR-2909`
aKO cKO bKO2 bKO1
"GSQR-5168" "GSQR-5167" "GSQR-5166" "GSQR-5165"

$CDIP698967
$CDIP698967$`TUHQ6-485`
epsilonKO betaKO gammaKO alphaKO deltaKO bKO cKO
"GHQ6-986" "GHQ6-985" "GHQ6-984" "GHQ6-983" "GHQ6-982" "GHQ6-981" "GHQ6-980"
aKO
"GHQ6-979"

$`ACYL272123-WGS`
$`ACYL272123-WGS`$`TUSFA-1583`
gammaKO alphaKO deltaKO bKO2 bKO1 cKO
"GSFA-1765" "GSFA-1764" "GSFA-1763" "GSFA-1762" "GSFA-1761" "GSFA-1760"
aKO
"GSFA-1759"

$`ACYL272123-WGS`$`TUSFA-732`
betaKO epsilonKO
"GSFA-536" "GSFA-535"

$ADEH290397
$ADEH290397$`TUI2Z-2220`
alphaKO gammaKO betaKO epsilonKO
"GI2Z-4408" "GI2Z-4407" "GI2Z-4406" "GI2Z-4405"

$ADEH290397$`TUI2Z-2221`
deltaKO
"GI2Z-4409"

$ADEH290397$`TUI2Z-2217`
cKO bKO
"GI2Z-4397" "GI2Z-4396"

$ADEH290397$`TUI2Z-2218`
aKO
"GI2Z-4398"

$ADEG429009
$ADEG429009$`TUHG1-52`
epsilonKO betaKO gammaKO alphaKO deltaKO bKO cKO aKO
"GHG1-86" "GHG1-85" "GHG1-84" "GHG1-83" "GHG1-82" "GHG1-81" "GHG1-80" "GHG1-79"

$ADIE930169

$ADIE930169$`TUL7G-2406`
aKO cKO bKO deltaKO alphaKO gammaKO
"GL7G-4446" "GL7G-4445" "GL7G-4444" "GL7G-4443" "GL7G-4442" "GL7G-4441"
betaKO epsilonKO
"GL7G-4440" "GL7G-4439"

$CDIP698971
$CDIP698971$`TUHYD-459`
epsilonKO betaKO gammaKO alphaKO deltaKO bKO cKO
"GHYD-949" "GHYD-948" "GHYD-947" "GHYD-946" "GHYD-945" "GHYD-944" "GHYD-943"
aKO
"GHYD-942"

$CDIP698964
$CDIP698964$`TUHZP-510`
epsilonKO betaKO gammaKO alphaKO deltaKO bKO
"GHZP-1041" "GHZP-1040" "GHZP-1039" "GHZP-1038" "GHZP-1037" "GHZP-1036"
cKO aKO
"GHZP-1035" "GHZP-1034"

$CDIP698962
$CDIP698962$`TUH9X-545`
epsilonKO betaKO gammaKO alphaKO deltaKO bKO
"GH9X-1082" "GH9X-1081" "GH9X-1080" "GH9X-1079" "GH9X-1078" "GH9X-1077"
cKO aKO
"GH9X-1076" "GH9X-1075"

$CEPI1173022
$CEPI1173022$`TULC4-1551`
gammaKO alphaKO deltaKO bKO2
"GLC4-1951" "GLC4-1950" "GLC4-1949" "GLC4-1948"

$CEPI1173022$`TULC4-1677`
betaKO epsilonKO
"GLC4-2145" "GLC4-2144"

$CEPI1173022$`TULC4-1549`
cKO aKO
"GLC4-1946" "GLC4-1945"

$CEPI1173022$`TULC4-1550`
bKO1
"GLC4-1947"

$BTHU1195464
$BTHU1195464$`TUL8P-3591`
bKO deltaKO alphaKO
"GL8P-4571" "GL8P-4570" "GL8P-4569"

```
$BTHU1195464$`TUL8P-3589`
     betaKO   epsilonKO
"GL8P-4567" "GL8P-4566"

$BTHU1195464$`TUL8P-3590`
    gammaKO
"GL8P-4568"

$BTHU1195464$`TUL8P-3592`
        cKO
"GL8P-4572"

$BTHU1195464$`TUL8P-3593`
        aKO
"GL8P-4573"


$CFET360106
$CFET360106$`TUHTH-562`
       bKO2         bKO1      deltaKO      alphaKO      gammaKO       betaKO
"GHTH-1528" "GHTH-1527" "GHTH-1526" "GHTH-1525" "GHTH-1524" "GHTH-1523"
  epsilonKO
"GHTH-1522"

$CFET360106$`TUHTH-468`
        cKO
"GHTH-1279"

$CFET360106$`TUHTH-288`
       aKO
"GHTH-770"


$`MAER349215-WGS`
$`MAER349215-WGS`$`TUSR4-347`
   gammaKO    alphaKO    deltaKO
"GSR4-591" "GSR4-590" "GSR4-589"

$`MAER349215-WGS`$`TUSR4-348`
 epsilonKO     betaKO
"GSR4-595" "GSR4-593"

$`MAER349215-WGS`$`TUSR4-636`
        aKO          cKO         bKO2         bKO1
"GSR4-1152" "GSR4-1151" "GSR4-1150" "GSR4-1149"


$CFIM590998
$CFIM590998$`TUJFK-1463`
        aKO          cKO          bKO      deltaKO      alphaKO      gammaKO
"GJFK-2843" "GJFK-2842" "GJFK-2841" "GJFK-2840" "GJFK-2839" "GJFK-2838"
     betaKO
"GJFK-2837"
```

```
$CFIM590998$`TUJFK-1462`
  epsilonKO
"GJFK-2836"



$CFLA446466
$CFLA446466$`TUHDJ-623`
     betaKO     gammaKO     alphaKO     deltaKO         bKO         cKO
"GHDJ-1086" "GHDJ-1085" "GHDJ-1084" "GHDJ-1083" "GHDJ-1082" "GHDJ-1081"
        aKO
"GHDJ-1080"

$CFLA446466$`TUHDJ-624`
  epsilonKO
"GHDJ-1087"



$CFUN1005048
$CFUN1005048$`TUJNH-2380`
        aKO         cKO         bKO     deltaKO     alphaKO     gammaKO
"GJNH-4441" "GJNH-4440" "GJNH-4439" "GJNH-4438" "GJNH-4437" "GJNH-4436"
     betaKO   epsilonKO
"GJNH-4435" "GJNH-4434"



$CCE593907
$CCE593907$`TUH26-1258`
        aKO         cKO         bKO     deltaKO     alphaKO     gammaKO
"GH26-2512" "GH26-2511" "GH26-2510" "GH26-2509" "GH26-2508" "GH26-2507"
     betaKO   epsilonKO
"GH26-2506" "GH26-2505"



$CGRA292564
$CGRA292564$`TULC9-1553`
    gammaKO     alphaKO     deltaKO        bKO2        bKO1         cKO
"GLC9-3080" "GLC9-3079" "GLC9-3078" "GLC9-3077" "GLC9-3076" "GLC9-3075"

$CGRA292564$`TULC9-1593`
  epsilonKO      betaKO
"GLC9-3152" "GLC9-3151"

$CGRA292564$`TULC9-1552`
        aKO
"GLC9-3074"



$`CGLU1232383-WGS`
$`CGLU1232383-WGS`$`TUSLZ-733`
  epsilonKO      betaKO     gammaKO     alphaKO     deltaKO         bKO
"GSLZ-1391" "GSLZ-1390" "GSLZ-1389" "GSLZ-1388" "GSLZ-1387" "GSLZ-1386"
        cKO         aKO
"GSLZ-1385" "GSLZ-1384"
```

```
$`CGLU1310161-WGS`
$`CGLU1310161-WGS`$`TUSLY-614`
  epsilonKO      betaKO     gammaKO     alphaKO     deltaKO         bKO 
"GSLY-1246" "GSLY-1245" "GSLY-1244" "GSLY-1243" "GSLY-1242" "GSLY-1241" 
        cKO 
"GSLY-1240" 

$`CGLU1310161-WGS`$`TUSLY-613`
        aKO 
"GSLY-1239" 


$CGLO700015
$CGLO700015$`TUH6A-118`
 epsilonKO     betaKO    gammaKO    alphaKO    deltaKO        bKO        cKO 
"GH6A-196" "GH6A-195" "GH6A-194" "GH6A-193" "GH6A-192" "GH6A-191" "GH6A-190" 
       aKO 
"GH6A-189" 


$`BTHU1279365-WGS`
$`BTHU1279365-WGS`$`TUSH9-3518`
        aKO         cKO         bKO     deltaKO     alphaKO     gammaKO 
"GSH9-5841" "GSH9-5840" "GSH9-5839" "GSH9-5838" "GSH9-5837" "GSH9-5836" 
     betaKO   epsilonKO 
"GSH9-5835" "GSH9-5834" 


$`CGLU1232381-WGS`
$`CGLU1232381-WGS`$`TUSLX-735`
  epsilonKO      betaKO     gammaKO     alphaKO     deltaKO         bKO 
"GSLX-1391" "GSLX-1390" "GSLX-1389" "GSLX-1388" "GSLX-1387" "GSLX-1386" 
        cKO         aKO 
"GSLX-1385" "GSLX-1384" 


$CGLU340322
$CGLU340322$`TUJBE-724`
  epsilonKO      betaKO     gammaKO     alphaKO     deltaKO         bKO 
"GJBE-1341" "GJBE-1340" "GJBE-1339" "GJBE-1338" "GJBE-1337" "GJBE-1336" 
        cKO         aKO 
"GJBE-1335" "GJBE-1334" 


$`MAVI1199187-WGS`
$`MAVI1199187-WGS`$`TUSQP-696`
  epsilonKO      betaKO     gammaKO     alphaKO         bKO         cKO 
"GSQP-1337" "GSQP-1336" "GSQP-1335" "GSQP-1334" "GSQP-1332" "GSQP-1331" 
        aKO 
"GSQP-1330" 

$`MAVI1199187-WGS`$noTU
deltaKO
```

```
    NA


$`CGLU1204414-WGS`
$`CGLU1204414-WGS`$`TUSME-607`
  epsilonKO      betaKO     gammaKO     alphaKO     deltaKO         bKO 
"GSME-1165" "GSME-1164" "GSME-1163" "GSME-1162" "GSME-1161" "GSME-1160" 
        cKO         aKO 
"GSME-1159" "GSME-1158" 



$CHOM360107
$CHOM360107$`TUHCX-272`
 epsilonKO     betaKO    gammaKO    alphaKO    deltaKO       bKO2       bKO1 
"GHCX-693" "GHCX-692" "GHCX-691" "GHCX-690" "GHCX-689" "GHCX-688" "GHCX-687" 

$CHOM360107$`TUHCX-262`
       cKO 
"GHCX-651" 

$CHOM360107$`TUHCX-282`
       aKO 
"GHCX-728" 



$CHYD632292
$CHYD632292$`TUHA8-645`
  epsilonKO      betaKO     gammaKO     alphaKO     deltaKO         bKO 
"GHA8-1357" "GHA8-1356" "GHA8-1355" "GHA8-1354" "GHA8-1353" "GHA8-1352" 
        cKO         aKO 
"GHA8-1351" "GHA8-1350" 



$`CEND1231626-WGS`
$`CEND1231626-WGS`$`TUSJ6-504`
   gammaKO    alphaKO    deltaKO        bKO        cKO        aKO 
"GSJ6-731" "GSJ6-730" "GSJ6-729" "GSJ6-728" "GSJ6-727" "GSJ6-726" 

$`CEND1231626-WGS`$`TUSJ6-149`
    betaKO 
"GSJ6-186" 

$`CEND1231626-WGS`$`TUSJ6-336`
 epsilonKO 
"GSJ6-473" 



$CSP480224
$CSP480224$`TUHIY-1905`
        aKO         cKO         bKO     deltaKO     alphaKO     gammaKO 
"GHIY-3342" "GHIY-3341" "GHIY-3340" "GHIY-3339" "GHIY-3338" "GHIY-3337" 
     betaKO   epsilonKO 
"GHIY-3336" "GHIY-3335" 
```

```
$`CHAL1121362-WGS`
$`CHAL1121362-WGS`$`TUSM0-768`
  epsilonKO      betaKO     gammaKO     alphaKO     deltaKO         bKO
"GSM0-1247" "GSM0-1246" "GSM0-1245" "GSM0-1244" "GSM0-1243" "GSM0-1242"
        cKO         aKO
"GSM0-1241" "GSM0-1240"


$CHUT269798
$CHUT269798$`TUJ83-107`
   gammaKO    alphaKO    deltaKO        bKO        cKO        aKO
"GJ83-195" "GJ83-194" "GJ83-193" "GJ83-192" "GJ83-191" "GJ83-190"

$CHUT269798$`TUJ83-204`
 epsilonKO     betaKO
"GJ83-347" "GJ83-346"


$CAULO
$CAULO$`TU1-7215`
  deltaKO   alphaKO   gammaKO    betaKO epsilonKO
 "CC3450"  "CC3449"  "CC3448"  "CC3447"  "CC3445"

$CAULO$`TU1-5462`
     aKO      cKO     bKO2     bKO1
"CC0368" "CC0367" "CC0366" "CC0365"


$CHYD246194
$CHYD246194$`TUJCN-1046`
        aKO         cKO         bKO     deltaKO     alphaKO     gammaKO
"GJCN-2550" "GJCN-2549" "GJCN-2548" "GJCN-2547" "GJCN-2546" "GJCN-2545"
     betaKO   epsilonKO
"GJCN-2544" "GJCN-2543"


$CJAP498211
$CJAP498211$`TUHIT-2051`
        aKO         cKO         bKO     deltaKO     alphaKO     gammaKO
"GHIT-3801" "GHIT-3800" "GHIT-3799" "GHIT-3798" "GHIT-3797" "GHIT-3796"
     betaKO   epsilonKO
"GHIT-3795" "GHIT-3794"


$CJEJ360109
$CJEJ360109$`TUJDG-61`
 epsilonKO     betaKO    gammaKO    alphaKO    deltaKO       bKO2       bKO1
"GJDG-112" "GJDG-111" "GJDG-110" "GJDG-109" "GJDG-108" "GJDG-107" "GJDG-106"

$CJEJ360109$`TUJDG-346`
       cKO
"GJDG-846"
```

$CJEJ360109$`TUJDG-212`
aKO
"GJDG-507"

$LBOR355276
$LBOR355276$`TUHUQ-990`
aKO cKO bKO deltaKO alphaKO gammaKO
"GHUQ-1956" "GHUQ-1955" "GHUQ-1954" "GHUQ-1953" "GHUQ-1952" "GHUQ-1951"
betaKO epsilonKO
"GHUQ-1950" "GHUQ-1949"

$MHYD351348
$MHYD351348$`TUHYZ-2158`
aKO cKO bKO deltaKO alphaKO
"GHYZ-3943" "GHYZ-3942" "GHYZ-3941" "GHYZ-3940" "GHYZ-3939"

$MHYD351348$`TUHYZ-2157`
gammaKO betaKO epsilonKO
"GHYZ-3938" "GHYZ-3937" "GHYZ-3936"

$CJEJ354242
$CJEJ354242$`TUC51-99`
epsilonKO betaKO gammaKO alphaKO deltaKO bKO2 bKO1
"GC51-118" "GC51-117" "GC51-116" "GC51-115" "GC51-114" "GC51-113" "GC51-112"

$CJEJ354242$`TUC51-396`
cKO
"GC51-917"

$CJEJ354242$`TUC51-495`
aKO
"GC51-1195"

$CJEI306537
$CJEI306537$`TUJ8V-700`
aKO cKO bKO deltaKO alphaKO gammaKO
"GJ8V-1386" "GJ8V-1385" "GJ8V-1384" "GJ8V-1383" "GJ8V-1382" "GJ8V-1381"
betaKO epsilonKO
"GJ8V-1380" "GJ8V-1379"

$CJEJ1201032
$CJEJ1201032$`TULAB-55`
epsilonKO betaKO gammaKO alphaKO deltaKO bKO2 bKO1
"GLAB-108" "GLAB-107" "GLAB-106" "GLAB-105" "GLAB-104" "GLAB-103" "GLAB-102"

$CJEJ1201032$`TULAB-332`
cKO
"GLAB-889"

$CJEJ1201032$`TULAB-420`
aKO
"GLAB-1150"

$CJEJ195099
$CJEJ195099$`TUJC0-52`
epsilonKO betaKO gammaKO alphaKO deltaKO bKO2 bKO1
"GJC0-107" "GJC0-106" "GJC0-105" "GJC0-104" "GJC0-103" "GJC0-102" "GJC0-101"

$CJEJ195099$`TUJC0-383`
cKO
"GJC0-1034"

$CJEJ195099$`TUJC0-509`
aKO
"GJC0-1364"

$CJEJ718271
$CJEJ718271$`TULAE-81`
epsilonKO betaKO gammaKO alphaKO deltaKO bKO2 bKO1
"GLAE-112" "GLAE-111" "GLAE-110" "GLAE-109" "GLAE-108" "GLAE-107" "GLAE-106"

$CJEJ718271$`TULAE-388`
cKO
"GLAE-975"

$CJEJ718271$`TULAE-483`
aKO
"GLAE-1248"

$CAULONA1000
$CAULONA1000$`TU6LA-4634`
deltaKO alphaKO gammaKO betaKO epsilonKO
"CCNA_03563" "CCNA_03562" "CCNA_03561" "CCNA_03560" "CCNA_03558"

$CAULONA1000$`TU6LA-2868`
aKO cKO bKO2 bKO1
"CCNA_00373" "CCNA_00372" "CCNA_00371" "CCNA_00370"

$`CJEJ1347340-WGS`
$`CJEJ1347340-WGS`$`TUSHY-51`
epsilonKO betaKO gammaKO alphaKO deltaKO bKO2 bKO1
"GSHY-102" "GSHY-101" "GSHY-100" "GSHY-99" "GSHY-98" "GSHY-97" "GSHY-96"

$`CJEJ1347340-WGS`$`TUSHY-357`
cKO
"GSHY-931"

$`CJEJ1347340-WGS`$`TUSHY-456`
aKO

"GSHY-1208"

$CKRI632335
$CKRI632335$`TUI3P-668`
aKO cKO bKO deltaKO alphaKO gammaKO
"GI3P-1440" "GI3P-1439" "GI3P-1438" "GI3P-1437" "GI3P-1436" "GI3P-1435"
betaKO epsilonKO
"GI3P-1434" "GI3P-1433"

$CKLU431943
$CKLU431943$`TUJF1-2188`
bKO deltaKO alphaKO gammaKO betaKO
"GJF1-3685" "GJF1-3684" "GJF1-3683" "GJF1-3682" "GJF1-3681"

$CKLU431943$`TUJF1-2187`
epsilonKO
"GJF1-3680"

$CKLU431943$`TUJF1-2189`
cKO
"GJF1-3686"

$CKLU431943$`TUJF1-2190`
aKO
"GJF1-3687"

$CKRO632348
$CKRO632348$`TUI5C-673`
epsilonKO betaKO gammaKO alphaKO deltaKO bKO
"GI5C-1329" "GI5C-1328" "GI5C-1327" "GI5C-1326" "GI5C-1325" "GI5C-1324"
cKO aKO
"GI5C-1323" "GI5C-1322"

$CKOS290338
$CKOS290338$`TUJ8L-48`
alphaKO gammaKO betaKO epsilonKO
"GJ8L-72" "GJ8L-71" "GJ8L-70" "GJ8L-69"

$CKOS290338$`TUJ8L-3297`
deltaKO
"GJ8L-74"

$CKOS290338$`TUJ8L-49`
aKO cKO bKO
"GJ8L-78" "GJ8L-77" "GJ8L-76"

$MAUS697281
$MAUS697281$`TUH33-184`
aKO cKO bKO deltaKO alphaKO gammaKO betaKO

"GH33-401" "GH33-400" "GH33-399" "GH33-398" "GH33-397" "GH33-396" "GH33-395"
epsilonKO
"GH33-394"

$ADEN596154
$ADEN596154$`TUHU6-196`
epsilonKO betaKO gammaKO alphaKO deltaKO bKO cKO
"GHU6-385" "GHU6-384" "GHU6-383" "GHU6-382" "GHU6-381" "GHU6-380" "GHU6-379"
aKO
"GHU6-378"

$ADEN596153
$ADEN596153$`TUHGY-289`
epsilonKO betaKO gammaKO alphaKO deltaKO bKO cKO
"GHGY-440" "GHGY-439" "GHGY-438" "GHGY-437" "GHGY-436" "GHGY-435" "GHGY-434"
aKO
"GHGY-433"

$AEHR187272
$AEHR187272$`TUHAX-1429`
aKO cKO bKO deltaKO alphaKO gammaKO
"GHAX-2931" "GHAX-2930" "GHAX-2929" "GHAX-2928" "GHAX-2927" "GHAX-2926"
betaKO epsilonKO
"GHAX-2925" "GHAX-2924"

$`AEQU1384484-WGS`
$`AEQU1384484-WGS`$`TUSET-444`
epsilonKO betaKO gammaKO alphaKO deltaKO bKO cKO
"GSET-755" "GSET-754" "GSET-753" "GSET-752" "GSET-751" "GSET-750" "GSET-749"
aKO
"GSET-748"

$CGLU196627
$CGLU196627$`TUJDM-630`
epsilonKO betaKO gammaKO alphaKO deltaKO bKO
"GJDM-1199" "GJDM-1198" "GJDM-1197" "GJDM-1196" "GJDM-1195" "GJDM-1194"
cKO aKO
"GJDM-1193" "GJDM-1192"

$CKRO645127
$CKRO645127$`TUI7D-735`
aKO cKO bKO deltaKO alphaKO gammaKO
"GI7D-1271" "GI7D-1270" "GI7D-1269" "GI7D-1268" "GI7D-1267" "GI7D-1266"
betaKO epsilonKO
"GI7D-1265" "GI7D-1264"

$CKLU583346

$CKLU583346$`TUJNQ-2009`
aKO cKO bKO deltaKO alphaKO gammaKO
"GJNQ-3331" "GJNQ-3330" "GJNQ-3329" "GJNQ-3328" "GJNQ-3327" "GJNQ-3326"
betaKO epsilonKO
"GJNQ-3325" "GJNQ-3324"

$CSP755731
$CSP755731$`TUJVM-164`
epsilonKO betaKO gammaKO alphaKO deltaKO bKO cKO
"GJVM-295" "GJVM-294" "GJVM-293" "GJVM-292" "GJVM-291" "GJVM-290" "GJVM-289"
aKO
"GJVM-288"

$CLAC632516
$CLAC632516$`TUHP3-385`
aKO cKO bKO deltaKO alphaKO gammaKO betaKO
"GHP3-812" "GHP3-811" "GHP3-810" "GHP3-809" "GHP3-808" "GHP3-807" "GHP3-806"
epsilonKO
"GHP3-805"

$CCLO642492
$CCLO642492$`TUIWK-2053`
aKO cKO bKO deltaKO alphaKO gammaKO
"GIWK-3770" "GIWK-3768" "GIWK-3767" "GIWK-3766" "GIWK-3765" "GIWK-3764"
betaKO epsilonKO
"GIWK-3763" "GIWK-3762"

$CLIM290315
$CLIM290315$`TUHUH-1340`
alphaKO gammaKO
"GHUH-2383" "GHUH-2382"

$CLIM290315$`TUHUH-21`
betaKO epsilonKO
"GHUH-29" "GHUH-28"

$CLIM290315$`TUHUH-1426`
cKO bKO deltaKO
"GHUH-2542" "GHUH-2541" "GHUH-2540"

$CLIM290315$noTU
aKO
NA

$MART243272
$MART243272$`TUHIZ-17`
epsilonKO betaKO1 gammaKO alphaKO1 deltaKO bKO cKO aKO
"GHIZ-44" "GHIZ-43" "GHIZ-42" "GHIZ-41" "GHIZ-40" "GHIZ-39" "GHIZ-38" "GHIZ-37"

```
$MART243272$`TUHIZ-96`
  alphaKO2    betaKO2
"GHIZ-201" "GHIZ-200"

$MART243272$`TUHIZ-180`
  alphaKO3    betaKO3
"GHIZ-377" "GHIZ-376"


$CLJU748727
$CLJU748727$`TUHMO-147`
 epsilonKO     betaKO    gammaKO    alphaKO    deltaKO        bKO        cKO
"GHMO-244" "GHMO-243" "GHMO-242" "GHMO-241" "GHMO-240" "GHMO-239" "GHMO-238"
       aKO
"GHMO-237"


$CGEN699246
$CGEN699246$`TUI5D-312`
 epsilonKO     betaKO    gammaKO    alphaKO        bKO        cKO        aKO
"GI5D-610" "GI5D-609" "GI5D-608" "GI5D-607" "GI5D-606" "GI5D-605" "GI5D-604"

$CGEN699246$noTU
deltaKO
     NA


$AARI861360
$AARI861360$`TUI6T-797`
  epsilonKO      betaKO     gammaKO     alphaKO     deltaKO         bKO
"GI6T-1369" "GI6T-1368" "GI6T-1367" "GI6T-1366" "GI6T-1365" "GI6T-1364"
        cKO         aKO
"GI6T-1363" "GI6T-1362"


$EREC515619
$EREC515619$`TUHMX-55`
epsilonKO1    betaKO1   gammaKO1   alphaKO1   deltaKO1       bKO1       cKO1
"GHMX-125" "GHMX-124" "GHMX-123" "GHMX-122" "GHMX-121" "GHMX-120" "GHMX-119"
      aKO1
"GHMX-118"

$EREC515619$`TUHMX-1389`
   deltaKO2    alphaKO2    gammaKO2     betaKO2  epsilonKO2
"GHMX-2889" "GHMX-2888" "GHMX-2887" "GHMX-2886" "GHMX-2885"

$EREC515619$`TUHMX-1391`
       cKO2
"GHMX-2891"

$EREC515619$`TUHMX-1392`
       aKO2
"GHMX-2892"
```

```
$EREC515619$`TUHMX-1390`
      bKO2
"GHMX-2890"


$AACT694569
$AACT694569$`TUL7F-629`
  epsilonKO     betaKO    gammaKO    alphaKO    deltaKO        bKO
"GL7F-1180" "GL7F-1179" "GL7F-1178" "GL7F-1177" "GL7F-1176" "GL7F-1175"
       cKO        aKO
"GL7F-1174" "GL7F-1173"


$AACT754507
$AACT754507$`TUHNV-343`
 epsilonKO    betaKO   gammaKO   alphaKO   deltaKO       bKO       cKO
"GHNV-623" "GHNV-622" "GHNV-621" "GHNV-620" "GHNV-619" "GHNV-618" "GHNV-617"
      aKO
"GHNV-616"


$AAPH634176
$AAPH634176$`TUHVL-1010`
  epsilonKO     betaKO    gammaKO    alphaKO    deltaKO        bKO
"GHVL-1964" "GHVL-1963" "GHVL-1962" "GHVL-1961" "GHVL-1960" "GHVL-1959"
       cKO        aKO
"GHVL-1958" "GHVL-1957"


$CLYT867900
$CLYT867900$`TUHJM-731`
    gammaKO    alphaKO    deltaKO        bKO        cKO        aKO
"GHJM-1423" "GHJM-1422" "GHJM-1421" "GHJM-1420" "GHJM-1419" "GHJM-1418"

$CLYT867900$`TUHJM-559`
     betaKO
"GHJM-1066"

$CLYT867900$`TUHJM-558`
  epsilonKO
"GHJM-1065"


$`CMIC1097677-WGS`
$`CMIC1097677-WGS`$`TUSL9-574`
  epsilonKO     betaKO    gammaKO    alphaKO    deltaKO        bKO
"GSL9-1118" "GSL9-1117" "GSL9-1116" "GSL9-1115" "GSL9-1114" "GSL9-1113"
       cKO        aKO
"GSL9-1112" "GSL9-1111"


$`CMAR1224163-WGS`
$`CMAR1224163-WGS`$`TUSNF-627`
  epsilonKO     betaKO    gammaKO    alphaKO    deltaKO        bKO
```

```
"GSNF-1174" "GSNF-1173" "GSNF-1172" "GSNF-1171" "GSNF-1170" "GSNF-1169"
       cKO        aKO
"GSNF-1168" "GSNF-1167"


$CMIC443906
$CMIC443906$`TUCI2-709`
  epsilonKO      betaKO     gammaKO     alphaKO     deltaKO         bKO
"GCI2-1207" "GCI2-1206" "GCI2-1205" "GCI2-1204" "GCI2-1203" "GCI2-1202"
       cKO        aKO
"GCI2-1201" "GCI2-1200"


$MAUR644283
$MAUR644283$`TUHOD-2670`
       aKO         cKO         bKO     deltaKO     alphaKO     gammaKO
"GHOD-5182" "GHOD-5181" "GHOD-5180" "GHOD-5179" "GHOD-5178" "GHOD-5177"
     betaKO
"GHOD-5176"

$MAUR644283$`TUHOD-2668`
  epsilonKO
"GHOD-5174"


$`CMAL1234679-WGS`
$`CMAL1234679-WGS`$`TUSJ7-478`
 epsilonKO     betaKO    gammaKO    alphaKO    deltaKO        bKO        cKO
"GSJ7-794" "GSJ7-793" "GSJ7-792" "GSJ7-791" "GSJ7-790" "GSJ7-789" "GSJ7-788"
      aKO
"GSJ7-787"


$`CMIN1173020-WGS`
$`CMIN1173020-WGS`$`TUSJF-2375`
    gammaKO     alphaKO     deltaKO        bKO2        bKO1         cKO
"GSJF-3089" "GSJF-3088" "GSJF-3087" "GSJF-3086" "GSJF-3085" "GSJF-3084"
       aKO
"GSJF-3083"

$`CMIN1173020-WGS`$`TUSJF-2659`
  epsilonKO      betaKO
"GSJF-3494" "GSJF-3493"


$MAGA2110
$MAGA2110$`TUC0J-156`
   betaKO1   alphaKO1
"GC0J-332" "GC0J-331"

$MAGA2110$`TUC0J-183`
 epsilonKO    betaKO2    gammaKO   alphaKO2    deltaKO        bKO        cKO
"GC0J-393" "GC0J-392" "GC0J-391" "GC0J-390" "GC0J-389" "GC0J-388" "GC0J-387"
      aKO
```

"GC0J-386"

$CMAR880070
$CMAR880070$`TUHDK-620`
aKO cKO bKO deltaKO alphaKO gammaKO
"GHDK-1146" "GHDK-1145" "GHDK-1144" "GHDK-1143" "GHDK-1142" "GHDK-1141"

$CMAR880070$`TUHDK-1180`
betaKO
"GHDK-2087"

$CMAR880070$`TUHDK-1179`
epsilonKO
"GHDK-2086"

$CMIC31964
$CMIC31964$`TUJBN-1132`
aKO cKO bKO deltaKO alphaKO gammaKO
"GJBN-1943" "GJBN-1942" "GJBN-1941" "GJBN-1940" "GJBN-1939" "GJBN-1938"
betaKO epsilonKO
"GJBN-1937" "GJBN-1936"

$CNEC1042878
$CNEC1042878$`TUH0Z-4454`
aKO cKO bKO deltaKO alphaKO gammaKO
"GH0Z-3594" "GH0Z-3593" "GH0Z-3592" "GH0Z-3591" "GH0Z-3590" "GH0Z-3589"
betaKO epsilonKO
"GH0Z-3588" "GH0Z-3587"

$CNIT768670
$CNIT768670$`TUHD1-557`
bKO2 bKO1 deltaKO alphaKO gammaKO betaKO
"GHD1-1630" "GHD1-1629" "GHD1-1628" "GHD1-1627" "GHD1-1626" "GHD1-1625"
epsilonKO
"GHD1-1624"

$CNIT768670$`TUHD1-530`
aKO cKO
"GHD1-1506" "GHD1-1505"

$COBS608506
$COBS608506$`TUH1S-563`
epsilonKO betaKO gammaKO alphaKO deltaKO bKO
"GH1S-1168" "GH1S-1167" "GH1S-1166" "GH1S-1165" "GH1S-1164" "GH1S-1163"
cKO aKO
"GH1S-1162" "GH1S-1161"

$COCH521097

```
$COCH521097$`TUH5D-985`
    alphaKO     gammaKO
"GH5D-1759" "GH5D-1757"

$COCH521097$`TUH5D-519`
    betaKO
"GH5D-932"

$COCH521097$`TUH5D-986`
        aKO         cKO         bKO     deltaKO
"GH5D-1765" "GH5D-1764" "GH5D-1763" "GH5D-1762"

$COCH521097$`TUH5D-518`
 epsilonKO
"GH5D-931"


$CPSE1087454
$CPSE1087454$`TULBQ-487`
 epsilonKO     betaKO    gammaKO    alphaKO    deltaKO        bKO        cKO
"GLBQ-858" "GLBQ-857" "GLBQ-856" "GLBQ-855" "GLBQ-854" "GLBQ-853" "GLBQ-852"
       aKO
"GLBQ-851"


$CPSE1168865
$CPSE1168865$`TULBR-466`
 epsilonKO     betaKO    gammaKO    alphaKO    deltaKO        bKO        cKO
"GLBR-881" "GLBR-880" "GLBR-879" "GLBR-878" "GLBR-877" "GLBR-876" "GLBR-875"
       aKO
"GLBR-874"


$MAVI243243
$MAVI243243$`TUH3Y-755`
  epsilonKO      betaKO     gammaKO     alphaKO     deltaKO         bKO
"GH3Y-1528" "GH3Y-1527" "GH3Y-1526" "GH3Y-1525" "GH3Y-1524" "GH3Y-1523"
        cKO         aKO
"GH3Y-1522" "GH3Y-1521"


$CPSE935697
$CPSE935697$`TULBX-505`
 epsilonKO     betaKO    gammaKO    alphaKO    deltaKO        bKO        cKO
"GLBX-887" "GLBX-886" "GLBX-885" "GLBX-884" "GLBX-883" "GLBX-882" "GLBX-881"
       aKO
"GLBX-880"


$AMAC314275
$AMAC314275$`TUHA7-3`
        aKO         cKO         bKO     deltaKO     alphaKO     gammaKO
"GHA7-4127" "GHA7-4126" "GHA7-4125" "GHA7-4123" "GHA7-4122" "GHA7-4121"
     betaKO   epsilonKO
```

"GHA7-4120" "GHA7-4119"

$CPSE1087451
$CPSE1087451$`TULBU-506`
epsilonKO betaKO gammaKO alphaKO deltaKO cKO aKO
"GLBU-883" "GLBU-882" "GLBU-881" "GLBU-880" "GLBU-879" "GLBU-877" "GLBU-876"

$CPSE1087451$noTU
bKO
NA

$CPSE1089446
$CPSE1089446$`TULBS-489`
epsilonKO betaKO gammaKO alphaKO deltaKO bKO cKO
"GLBS-909" "GLBS-908" "GLBS-907" "GLBS-906" "GLBS-905" "GLBS-904" "GLBS-903"
aKO
"GLBS-902"

$CPSE1087453
$CPSE1087453$`TULBV-477`
epsilonKO betaKO gammaKO alphaKO deltaKO bKO cKO
"GLBV-865" "GLBV-864" "GLBV-863" "GLBV-862" "GLBV-861" "GLBV-860" "GLBV-859"
aKO
"GLBV-858"

$CPSE1161911
$CPSE1161911$`TULBY-486`
epsilonKO betaKO gammaKO alphaKO deltaKO bKO cKO
"GLBY-874" "GLBY-873" "GLBY-872" "GLBY-871" "GLBY-870" "GLBY-869" "GLBY-868"
aKO
"GLBY-867"

$COWE632518
$COWE632518$`TUHVV-554`
aKO cKO bKO deltaKO alphaKO gammaKO
"GHVV-1226" "GHVV-1225" "GHVV-1224" "GHVV-1223" "GHVV-1222" "GHVV-1221"
betaKO epsilonKO
"GHVV-1220" "GHVV-1219"

$`CPAS86416-WGS`
$`CPAS86416-WGS`$`TUSMF-2717`
aKO cKO bKO deltaKO alphaKO gammaKO
"GSMF-4382" "GSMF-4381" "GSMF-4380" "GSMF-4379" "GSMF-4378" "GSMF-4377"
betaKO epsilonKO
"GSMF-4376" "GSMF-4375"

$CPHA331678

```
$CPHA331678$`TUHME-182`
   gammaKO    alphaKO
"GHME-311" "GHME-310"

$CPHA331678$`TUHME-21`
   betaKO epsilonKO
"GHME-31" "GHME-30"

$CPHA331678$`TUHME-1470`
       aKO         cKO         bKO     deltaKO
"GHME-2557" "GHME-2556" "GHME-2555" "GHME-2554"


$CPAR517417
$CPAR517417$`TUH95-626`
   gammaKO1    alphaKO1        bKO1        cKO1        aKO1  epsilonKO2
"GH95-1103" "GH95-1102" "GH95-1101" "GH95-1100" "GH95-1099" "GH95-1096"
    betaKO2
"GH95-1095"

$CPAR517417$`TUH95-1097`
   alphaKO2    gammaKO2
"GH95-1962" "GH95-1961"

$CPAR517417$`TUH95-26`
   betaKO1 epsilonKO1
 "GH95-45"  "GH95-44"

$CPAR517417$`TUH95-1189`
      aKO2        cKO2        bKO2     deltaKO
"GH95-2106" "GH95-2105" "GH95-2104" "GH95-2103"


$CPER195102
$CPER195102$`TUJFM-1406`
       bKO     deltaKO     alphaKO     gammaKO      betaKO   epsilonKO
"GJFM-2254" "GJFM-2253" "GJFM-2252" "GJFM-2251" "GJFM-2250" "GJFM-2249"

$CPER195102$`TUJFM-1407`
       aKO         cKO
"GJFM-2256" "GJFM-2255"


$MBAR269797
$MBAR269797$`TUHUW-2148`
     betaKO   epsilonKO         aKO         cKO         bKO     alphaKO
"GHUW-3156" "GHUW-3155" "GHUW-3152" "GHUW-3151" "GHUW-3150" "GHUW-3149"
    gammaKO
"GHUW-3148"

$MBAR269797$noTU
deltaKO
     NA
```

```
$FPSY402612
$FPSY402612$`TUJEP-3401`
   alphaKO
"GJEP-4295"

$FPSY402612$`TUJEP-148`
   betaKO
"GJEP-192"

$FPSY402612$`TUJEP-3399`
   gammaKO
"GJEP-4293"

$FPSY402612$`TUJEP-3402`
   deltaKO
"GJEP-4297"

$FPSY402612$`TUJEP-150`
 epsilonKO
"GJEP-194"

$FPSY402612$`TUJEP-3405`
       cKO
"GJEP-4301"

$FPSY402612$`TUJEP-3406`
       aKO
"GJEP-4303"

$FPSY402612$`TUJEP-3403`
       bKO
"GJEP-4299"


$CPER195103
$CPER195103$`TUHAW-1492`
       aKO         cKO         bKO     deltaKO     alphaKO     gammaKO
"GHAW-2473" "GHAW-2472" "GHAW-2471" "GHAW-2470" "GHAW-2469" "GHAW-2468"
    betaKO   epsilonKO
"GHAW-2467" "GHAW-2466"


$CPSE1074485
$CPSE1074485$`TULBT-485`
 epsilonKO     betaKO    gammaKO    alphaKO    deltaKO        bKO        cKO
"GLBT-904" "GLBT-903" "GLBT-902" "GLBT-901" "GLBT-900" "GLBT-899" "GLBT-898"
      aKO
"GLBT-897"


$CPHA290317
$CPHA290317$`TUHX4-1479`
   alphaKO
```

"GHX4-2592"

$CPHA290317$`TUHX4-26`
betaKO epsilonKO
"GHX4-49" "GHX4-48"

$CPHA290317$`TUHX4-1478`
gammaKO
"GHX4-2591"

$CPHA290317$`TUHX4-1583`
bKO deltaKO
"GHX4-2763" "GHX4-2762"

$CPHA290317$`TUHX4-1584`
cKO
"GHX4-2764"

$CPHA290317$`TUHX4-1585`
aKO
"GHX4-2765"

$CPIN485918
$CPIN485918$`TUHYR-769`
alphaKO deltaKO bKO cKO aKO
"GHYR-1252" "GHYR-1251" "GHYR-1250" "GHYR-1249" "GHYR-1248"

$CPIN485918$`TUHYR-4250`
betaKO epsilonKO
"GHYR-7169" "GHYR-7168"

$CPIN485918$`TUHYR-4360`
gammaKO
"GHYR-7347"

$CPSE679896
$CPSE679896$`TULBP-491`
epsilonKO betaKO gammaKO alphaKO deltaKO bKO cKO
"GLBP-889" "GLBP-888" "GLBP-887" "GLBP-886" "GLBP-885" "GLBP-884" "GLBP-883"
aKO
"GLBP-882"

$CPSE1087452
$CPSE1087452$`TUJTM-486`
epsilonKO betaKO gammaKO alphaKO deltaKO bKO cKO
"GJTM-888" "GJTM-887" "GJTM-886" "GJTM-885" "GJTM-884" "GJTM-883" "GJTM-882"
aKO
"GJTM-881"

$AEXC573065

```
$AEXC573065$`TUJ7A-300`
    betaKO    gammaKO    alphaKO    deltaKO
"GJ7A-263" "GJ7A-262" "GJ7A-261" "GJ7A-260"

$AEXC573065$`TUJ7A-301`
 epsilonKO
"GJ7A-264"

$AEXC573065$`TUJ7A-792`
        aKO         cKO
"GJ7A-1131" "GJ7A-1130"

$AEXC573065$`TUJ7A-791`
       bKO2        bKO1
"GJ7A-1129" "GJ7A-1128"


$AFER380394
$AFER380394$`TUHE0-1305`
        aKO         cKO         bKO     deltaKO     alphaKO    gammaKO2
"GHE0-2867" "GHE0-2866" "GHE0-2865" "GHE0-2864" "GHE0-2863" "GHE0-2862"
     betaKO  epsilonKO3
"GHE0-2861" "GHE0-2860"

$AFER380394$`TUHE0-753`
   gammaKO1
"GHE0-1739"

$AFER380394$`TUHE0-385`
epsilonKO1
"GHE0-920"

$AFER380394$`TUHE0-922`
 epsilonKO2
"GHE0-2095"


$AFER743299
$AFER743299$`TUH39-1454`
        aKO         cKO         bKO     deltaKO     alphaKO     gammaKO
"GH39-3252" "GH39-3251" "GH39-3250" "GH39-3249" "GH39-3248" "GH39-3247"
     betaKO  epsilonKO2
"GH39-3246" "GH39-3245"

$AFER743299$`TUH39-752`
 epsilonKO1
"GH39-1632"


$AFLA491915
$AFLA491915$`TUHEO-1272`
        bKO     deltaKO     alphaKO
"GHEO-2795" "GHEO-2794" "GHEO-2793"
```

```
$AFLA491915$`TUHEO-1271`
    gammaKO      betaKO   epsilonKO
"GHEO-2792" "GHEO-2791" "GHEO-2790"

$AFLA491915$`TUHEO-1273`
        cKO
"GHEO-2796"

$AFLA491915$`TUHEO-1274`
        aKO
"GHEO-2797"


$CLAR306263
$CLAR306263$`TUH7X-103`
 epsilonKO     betaKO    gammaKO    alphaKO    deltaKO       bKO2       bKO1
"GH7X-196" "GH7X-195" "GH7X-194" "GH7X-193" "GH7X-192" "GH7X-191" "GH7X-190"

$CLAR306263$`TUH7X-426`
        cKO
"GH7X-1199"

$CLAR306263$`TUH7X-429`
        aKO
"GH7X-1206"


$FTAF755732
$FTAF755732$`TUHMH-651`
        aKO         cKO         bKO     deltaKO     alphaKO     gammaKO
"GHMH-1184" "GHMH-1183" "GHMH-1182" "GHMH-1181" "GHMH-1180" "GHMH-1178"

$FTAF755732$`TUHMH-627`
     betaKO
"GHMH-1136"

$FTAF755732$`TUHMH-628`
  epsilonKO
"GHMH-1137"


$MBOV410289
$MBOV410289$`TUJW7-718`
  epsilonKO      betaKO     gammaKO     alphaKO     deltaKO         bKO
"GJW7-1392" "GJW7-1391" "GJW7-1390" "GJW7-1389" "GJW7-1388" "GJW7-1387"
        cKO         aKO
"GJW7-1386" "GJW7-1385"


$CPRO309798
$CPRO309798$`TUH7M-231`
 epsilonKO     betaKO    gammaKO    alphaKO       bKO        cKO        aKO
"GH7M-568" "GH7M-567" "GH7M-566" "GH7M-565" "GH7M-564" "GH7M-563" "GH7M-562"
```

```
$CPRO309798$noTU
deltaKO
    NA



$CPSE1117942
$CPSE1117942$`TULC0-492`
 epsilonKO     betaKO    gammaKO    alphaKO    deltaKO        bKO        cKO
"GLC0-885" "GLC0-884" "GLC0-883" "GLC0-882" "GLC0-881" "GLC0-880" "GLC0-879"
      aKO
"GLC0-878"



$CPSE681645
$CPSE681645$`TULBW-492`
 epsilonKO     betaKO    gammaKO    alphaKO    deltaKO        bKO        cKO
"GLBW-889" "GLBW-888" "GLBW-887" "GLBW-886" "GLBW-885" "GLBW-884" "GLBW-883"
      aKO
"GLBW-882"



$CPER289380
$CPER289380$`TUI76-1332`
       aKO         cKO         bKO     deltaKO     alphaKO     gammaKO
"GI76-2180" "GI76-2179" "GI76-2178" "GI76-2177" "GI76-2176" "GI76-2175"
    betaKO   epsilonKO
"GI76-2174" "GI76-2173"



$CPSY167879
$CPSY167879$`TUI48-27`
epsilonKO    betaKO   gammaKO   alphaKO   deltaKO       bKO       cKO       aKO
"GI48-63" "GI48-62" "GI48-61" "GI48-60" "GI48-59" "GI48-58" "GI48-57" "GI48-56"



$CPSE765874
$CPSE765874$`TUH5Y-484`
 epsilonKO     betaKO    gammaKO    alphaKO    deltaKO        bKO        cKO
"GH5Y-875" "GH5Y-874" "GH5Y-873" "GH5Y-872" "GH5Y-871" "GH5Y-870" "GH5Y-869"
      aKO
"GH5Y-868"



$CPSE889513
$CPSE889513$`TULBZ-483`
 epsilonKO     betaKO    gammaKO    alphaKO    deltaKO        bKO        cKO
"GLBZ-887" "GLBZ-886" "GLBZ-885" "GLBZ-884" "GLBZ-883" "GLBZ-882" "GLBZ-881"
      aKO
"GLBZ-880"



$CPHY357809
$CPHY357809$`TUHCL-2469`
       aKO         cKO         bKO     deltaKO     alphaKO     gammaKO
```

"GHCL-3814" "GHCL-3813" "GHCL-3812" "GHCL-3811" "GHCL-3810" "GHCL-3809"
betaKO epsilonKO
"GHCL-3808" "GHCL-3807"

$CPSE935298
$CPSE935298$`TULC1-490`
epsilonKO betaKO gammaKO alphaKO deltaKO bKO cKO
"GLC1-885" "GLC1-884" "GLC1-883" "GLC1-882" "GLC1-881" "GLC1-880" "GLC1-879"
aKO
"GLC1-878"

$GMET269799
$GMET269799$`TUHNY-1757`
bKO2 bKO1 deltaKO alphaKO gammaKO betaKO
"GHNY-3460" "GHNY-3459" "GHNY-3458" "GHNY-3457" "GHNY-3456" "GHNY-3455"
epsilonKO
"GHNY-3454"

$GMET269799$`TUHNY-1731`
cKO aKO
"GHNY-3408" "GHNY-3407"

$CRUD1202537
$CRUD1202537$`TULAF-1`
betaKO gammaKO alphaKO cKO aKO
"GLAF-9" "GLAF-8" "GLAF-7" "GLAF-4" "GLAF-3"

$CRUD1202537$noTU
deltaKO epsilonKO bKO
NA NA NA

$MBOV956483
$MBOV956483$`TUHJX-207`
aKO cKO bKO deltaKO alphaKO1 gammaKO betaKO1
"GHJX-438" "GHJX-437" "GHJX-436" "GHJX-435" "GHJX-434" "GHJX-433" "GHJX-432"
epsilonKO
"GHJX-431"

$MBOV956483$`TUHJX-235`
alphaKO2 betaKO2
"GHJX-493" "GHJX-492"

$CRES662755
$CRES662755$`TUIVW-835`
aKO cKO bKO deltaKO alphaKO gammaKO
"GIVW-1455" "GIVW-1454" "GIVW-1453" "GIVW-1452" "GIVW-1451" "GIVW-1450"
betaKO epsilonKO
"GIVW-1449" "GIVW-1448"

```
$CRUD1202538
$CRUD1202538$`TULAG-1`
  betaKO  gammaKO  alphaKO      cKO      aKO 
"GLAG-9" "GLAG-8" "GLAG-7" "GLAG-4" "GLAG-3" 

$CRUD1202538$noTU
  deltaKO epsilonKO       bKO 
       NA        NA        NA 


$`CCAR667013-WGS`
$`CCAR667013-WGS`$`TUSI5-1`
  betaKO  gammaKO  alphaKO      cKO      aKO 
"GSI5-9" "GSI5-8" "GSI5-7" "GSI5-4" "GSI5-3" 

$`CCAR667013-WGS`$noTU
  deltaKO epsilonKO       bKO 
       NA        NA        NA 


$CSP208596
$CSP208596$`TUHHT-995`
        aKO         cKO         bKO     deltaKO     alphaKO     gammaKO 
"GHHT-1702" "GHHT-1701" "GHHT-1700" "GHHT-1699" "GHHT-1698" "GHHT-1697" 
     betaKO   epsilonKO 
"GHHT-1696" "GHHT-1695" 


$CROD637910
$CROD637910$`TUJIG-2149`
  epsilonKO      betaKO     gammaKO     alphaKO     deltaKO         bKO 
"GJIG-4060" "GJIG-4059" "GJIG-4058" "GJIG-4057" "GJIG-4056" "GJIG-4055" 
        cKO         aKO 
"GJIG-4054" "GJIG-4053" 


$CCAR387662
$CCAR387662$`TUBZ4-1`
  betaKO  gammaKO  alphaKO      cKO      aKO 
"GBZ4-9" "GBZ4-8" "GBZ4-7" "GBZ4-4" "GBZ4-3" 

$CCAR387662$noTU
  deltaKO epsilonKO       bKO 
       NA        NA        NA 


$CRUD1202539
$CRUD1202539$`TULAH-3`
   betaKO   gammaKO   alphaKO       cKO       aKO 
"GLAH-11" "GLAH-10"  "GLAH-9"  "GLAH-6"  "GLAH-5" 

$CRUD1202539$noTU
  deltaKO epsilonKO       bKO 
```

```
      NA        NA        NA


$CRUD1202536
$CRUD1202536$`TULAD-1`
  betaKO  gammaKO  alphaKO      cKO      aKO
"GLAD-9" "GLAD-8" "GLAD-7" "GLAD-4" "GLAD-3"

$CRUD1202536$noTU
  deltaKO epsilonKO       bKO
      NA        NA        NA


$GSUL663917
$GSUL663917$`TULDN-49`
epsilonKO    betaKO   gammaKO   alphaKO   deltaKO      bKO2      bKO1
"GLDN-93" "GLDN-92" "GLDN-91" "GLDN-90" "GLDN-89" "GLDN-88" "GLDN-87"

$GSUL663917$`TULDN-163`
       aKO        cKO
"GLDN-306" "GLDN-305"


$`CCAR1202540-WGS`
$`CCAR1202540-WGS`$`TUSIR-1`
  betaKO  gammaKO  alphaKO      cKO      aKO
"GSIR-9" "GSIR-8" "GSIR-7" "GSIR-4" "GSIR-3"

$`CCAR1202540-WGS`$noTU
  deltaKO epsilonKO       bKO
      NA        NA        NA


$CSAL290398
$CSAL290398$`TUCJW-7030`
        aKO         cKO         bKO     deltaKO     alphaKO     gammaKO
"GCJW-3559" "GCJW-3558" "GCJW-3557" "GCJW-3556" "GCJW-3555" "GCJW-3554"
     betaKO   epsilonKO
"GCJW-3553" "GCJW-3552"


$`MBOV1206780-WGS`
$`MBOV1206780-WGS`$`TUSQS-728`
  epsilonKO      betaKO     gammaKO     alphaKO         bKO         cKO
"GSQS-1425" "GSQS-1424" "GSQS-1423" "GSQS-1422" "GSQS-1420" "GSQS-1419"
        aKO
"GSQS-1418"

$`MBOV1206780-WGS`$noTU
deltaKO
     NA


$`CSAC1345695-WGS`
```

$`CSAC1345695-WGS`$`TUSLJ-290`
epsilonKO betaKO gammaKO alphaKO deltaKO bKO cKO
"GSLJ-516" "GSLJ-515" "GSLJ-514" "GSLJ-513" "GSLJ-512" "GSLJ-511" "GSLJ-510"
aKO
"GSLJ-509"

$CSAC351627
$CSAC351627$`TUJ17-992`
aKO cKO bKO deltaKO alphaKO gammaKO
"GJ17-2017" "GJ17-2016" "GJ17-2015" "GJ17-2014" "GJ17-2013" "GJ17-2012"
betaKO epsilonKO
"GJ17-2011" "GJ17-2010"

$CSEG509190
$CSEG509190$`TUHVG-145`
epsilonKO betaKO gammaKO alphaKO deltaKO
"GHVG-246" "GHVG-244" "GHVG-243" "GHVG-242" "GHVG-241"

$CSEG509190$`TUHVG-205`
aKO cKO
"GHVG-352" "GHVG-351"

$CSEG509190$`TUHVG-204`
bKO2 bKO1
"GHVG-350" "GHVG-349"

$`CSTA56107-WGS`
$`CSTA56107-WGS`$`TUSM9-595`
aKO cKO bKO2 bKO1 deltaKO alphaKO gammaKO
"GSM9-532" "GSM9-531" "GSM9-530" "GSM9-529" "GSM9-528" "GSM9-527" "GSM9-526"

$`CSTA56107-WGS`$`TUSM9-3270`
betaKO epsilonKO
"GSM9-4494" "GSM9-4493"

$CSAC610130
$CSAC610130$`TUHTP-2116`
aKO1 cKO1 bKO1 deltaKO1 alphaKO1 gammaKO1
"GHTP-4081" "GHTP-4080" "GHTP-4079" "GHTP-4078" "GHTP-4077" "GHTP-4076"
betaKO1 epsilonKO1
"GHTP-4075" "GHTP-4074"

$CSAC610130$`TUHTP-2210`
aKO2 cKO2 bKO2 deltaKO2 alphaKO2 gammaKO2
"GHTP-4254" "GHTP-4253" "GHTP-4252" "GHTP-4251" "GHTP-4250" "GHTP-4249"
betaKO2 epsilonKO2
"GHTP-4248" "GHTP-4247"

$CSAK1138308

```
$CSAK1138308$`TULC5-4`
       aKO         cKO         bKO     deltaKO     alphaKO     gammaKO 
"GLC5-4015" "GLC5-4014" "GLC5-4013" "GLC5-4012" "GLC5-4011" "GLC5-4010" 
     betaKO   epsilonKO 
"GLC5-4009" "GLC5-4008" 


$`CSTA292563-WGS`
$`CSTA292563-WGS`$`TUSM6-58`
      aKO       cKO      bKO2      bKO1   deltaKO   alphaKO   gammaKO 
"GSM6-86" "GSM6-85" "GSM6-84" "GSM6-83" "GSM6-82" "GSM6-81" "GSM6-80" 

$`CSTA292563-WGS`$`TUSM6-466`
    betaKO  epsilonKO 
"GSM6-686" "GSM6-685" 


$GSUL243231
$GSUL243231$`TUH27-61`
 epsilonKO     betaKO    gammaKO    alphaKO    deltaKO       bKO2       bKO1 
"GH27-120" "GH27-119" "GH27-118" "GH27-117" "GH27-116" "GH27-115" "GH27-114" 

$GSUL243231$`TUH27-174`
       aKO        cKO 
"GH27-339" "GH27-338" 


$`CSAK956149-WGS`
$`CSAK956149-WGS`$`TUSM7-2137`
       aKO         cKO         bKO     deltaKO     alphaKO     gammaKO 
"GSM7-3777" "GSM7-3776" "GSM7-3775" "GSM7-3774" "GSM7-3773" "GSM7-3772" 
     betaKO   epsilonKO 
"GSM7-3771" "GSM7-3770" 


$CTEP194439
$CTEP194439$`TUHN0-1078`
    alphaKO 
"GHN0-2074" 

$CTEP194439$`TUHN0-551`
    betaKO1  epsilonKO1        aKO2 
"GHN0-1070" "GHN0-1069" "GHN0-1066" 

$CTEP194439$`TUHN0-1188`
 epsilonKO2     betaKO2 
"GHN0-2284" "GHN0-2283" 

$CTEP194439$`TUHN0-1077`
    gammaKO 
"GHN0-2073" 

$CTEP194439$`TUHN0-12`
      cKO       bKO   deltaKO 
```

```
"GHN0-21" "GHN0-20" "GHN0-19"

$CTEP194439$`TUHN0-13`
    aKO1
"GHN0-22"


$`CTER1200352-WGS`
$`CTER1200352-WGS`$`TUSM3-754`
      aKO         cKO         bKO     deltaKO     alphaKO     gammaKO
"GSM3-1526" "GSM3-1525" "GSM3-1524" "GSM3-1523" "GSM3-1522" "GSM3-1521"
    betaKO   epsilonKO
"GSM3-1520" "GSM3-1519"


$MBOV717522
$MBOV717522$`TUJXR-704`
  epsilonKO      betaKO     gammaKO     alphaKO         bKO         cKO
"GJXR-1361" "GJXR-1360" "GJXR-1359" "GJXR-1358" "GJXR-1356" "GJXR-1355"
      aKO
"GJXR-1354"

$MBOV717522$noTU
deltaKO
    NA


$CTHE203119
$CTHE203119$`TUIW8-1662`
   alphaKO     deltaKO         bKO         cKO         aKO
"GIW8-2697" "GIW8-2696" "GIW8-2695" "GIW8-2694" "GIW8-2693"

$CTHE203119$`TUIW8-1663`
  epsilonKO      betaKO     gammaKO
"GIW8-2700" "GIW8-2699" "GIW8-2698"


$CTHE251229
$CTHE251229$`TULBB-3232`
     bKO1     deltaKO     alphaKO     gammaKO
"GLBB-4330" "GLBB-4329" "GLBB-4328" "GLBB-4327"

$CTHE251229$`TULBB-1499`
  epsilonKO      betaKO
"GLBB-1793" "GLBB-1792"

$CTHE251229$`TULBB-3234`
      aKO         cKO
"GLBB-4333" "GLBB-4332"

$CTHE251229$`TULBB-3233`
     bKO2
"GLBB-4331"
```

$CTAI977880
$CTAI977880$`TULC7-2086`
aKO cKO bKO deltaKO alphaKO gammaKO
"GLC7-3101" "GLC7-3100" "GLC7-3099" "GLC7-3098" "GLC7-3097" "GLC7-3096"
betaKO epsilonKO
"GLC7-3095" "GLC7-3094"

$CTHE981222
$CTHE981222$`TUHDQ-943`
bKO2 bKO1 deltaKO alphaKO gammaKO
"GHDQ-1629" "GHDQ-1628" "GHDQ-1627" "GHDQ-1626" "GHDQ-1625"

$CTHE981222$`TUHDQ-667`
betaKO
"GHDQ-1148"

$CTHE981222$`TUHDQ-110`
epsilonKO
"GHDQ-207"

$CTHE981222$`TUHDQ-546`
cKO
"GHDQ-940"

$CTHE981222$`TUHDQ-545`
aKO
"GHDQ-939"

$CTHA517418
$CTHA517418$`TUHTO-574`
gammaKO alphaKO
"GHTO-885" "GHTO-884"

$CTHA517418$`TUHTO-317`
betaKO epsilonKO
"GHTO-483" "GHTO-482"

$CTHA517418$`TUHTO-910`
deltaKO bKO cKO aKO
"GHTO-1407" "GHTO-1406" "GHTO-1405" "GHTO-1404"

$CTUR693216
$CTUR693216$`TUI0P-143`
epsilonKO betaKO gammaKO alphaKO deltaKO bKO cKO aKO
"GI0P-11" "GI0P-10" "GI0P-9" "GI0P-8" "GI0P-7" "GI0P-6" "GI0P-5" "GI0P-4"

$`HNIT1029756-WGS`
$`HNIT1029756-WGS`$`TUSP5-283`
betaKO gammaKO alphaKO deltaKO

```
"GSP5-473" "GSP5-470" "GSP5-469" "GSP5-468"

$`HNIT1029756-WGS`$`TUSP5-284`
 epsilonKO 
"GSP5-475" 

$`HNIT1029756-WGS`$`TUSP5-650`
       aKO        cKO 
"GSP5-1109" "GSP5-1108" 

$`HNIT1029756-WGS`$`TUSP5-649`
      bKO2       bKO1 
"GSP5-1107" "GSP5-1106" 


$CTHE637887
$CTHE637887$`TULBN-126`
 epsilonKO     betaKO    gammaKO    alphaKO    deltaKO        bKO        cKO 
"GLBN-201" "GLBN-200" "GLBN-199" "GLBN-198" "GLBN-197" "GLBN-196" "GLBN-195" 
      aKO 
"GLBN-194" 


$`CURE1267754-WGS`
$`CURE1267754-WGS`$`TUSM4-408`
 epsilonKO     betaKO    gammaKO    alphaKO    deltaKO        bKO        cKO 
"GSM4-720" "GSM4-719" "GSM4-718" "GSM4-717" "GSM4-716" "GSM4-715" "GSM4-714" 
      aKO 
"GSM4-713" 


$AFER591001
$AFER591001$`TUHUL-893`
       aKO         cKO         bKO     alphaKO     gammaKO      betaKO 
"GHUL-1781" "GHUL-1780" "GHUL-1779" "GHUL-1778" "GHUL-1777" "GHUL-1776" 
  epsilonKO 
"GHUL-1775" 

$AFER591001$noTU
deltaKO 
     NA 


$AFER525909
$AFER525909$`TUHMR-763`
       aKO         cKO         bKO     deltaKO     alphaKO     gammaKO 
"GHMR-1843" "GHMR-1842" "GHMR-1841" "GHMR-1840" "GHMR-1839" "GHMR-1838" 
     betaKO   epsilonKO 
"GHMR-1837" "GHMR-1836" 


$LBUC1071400
$LBUC1071400$`TULF2-551`
 epsilonKO     betaKO    gammaKO    alphaKO    deltaKO        bKO        cKO 
```

"GLF2-973" "GLF2-972" "GLF2-971" "GLF2-970" "GLF2-969" "GLF2-968" "GLF2-967"
aKO
"GLF2-966"

$`MBOV233413-WGS`
$`MBOV233413-WGS`$`TUSQQ-705`
epsilonKO betaKO gammaKO alphaKO deltaKO bKO
"GSQQ-1360" "GSQQ-1359" "GSQQ-1358" "GSQQ-1357" "GSQQ-1356" "GSQQ-1355"
cKO aKO
"GSQQ-1354" "GSQQ-1353"

$AFER243159
$AFER243159$`TUH3S-1409`
aKO cKO bKO deltaKO alphaKO gammaKO2
"GH3S-3204" "GH3S-3203" "GH3S-3202" "GH3S-3201" "GH3S-3200" "GH3S-3199"
betaKO epsilonKO3
"GH3S-3198" "GH3S-3197"

$AFER243159$`TUH3S-1563`
gammaKO1
"GH3S-2043"

$AFER243159$`TUH3S-300`
epsilonKO1
"GH3S-758"

$AFER243159$`TUH3S-1022`
epsilonKO2
"GH3S-2418"

$`AFRI1246995-WGS`
$`AFRI1246995-WGS`$`TUSER-3690`
cKO bKO deltaKO alphaKO gammaKO betaKO
"GSER-7508" "GSER-7507" "GSER-7506" "GSER-7505" "GSER-7504" "GSER-7503"

$`AFRI1246995-WGS`$`TUSER-3688`
epsilonKO
"GSER-7501"

$`AFRI1246995-WGS`$`TUSER-3691`
aKO
"GSER-7509"

$CULC945711
$CULC945711$`TULC2-501`
epsilonKO betaKO gammaKO alphaKO deltaKO bKO cKO
"GLC2-915" "GLC2-914" "GLC2-913" "GLC2-912" "GLC2-911" "GLC2-910" "GLC2-909"
aKO
"GLC2-908"

```
$CULC996634
$CULC996634$`TULC3-552`
  epsilonKO      betaKO     gammaKO     alphaKO     deltaKO         bKO 
"GLC3-1035" "GLC3-1034" "GLC3-1033" "GLC3-1032" "GLC3-1031" "GLC3-1030" 
        cKO         aKO 
"GLC3-1029" "GLC3-1028" 



$HPY
$HPY$`TUI-3010`
     bKO2      bKO1   deltaKO   alphaKO   gammaKO    betaKO epsilonKO 
 "HP1137"  "HP1136"  "HP1135"  "HP1134"  "HP1133"  "HP1132"  "HP1131" 

$HPY$`TUI-3053`
     cKO 
"HP1212" 

$HPY$`TUI-2871`
     aKO 
"HP0828" 



$CULC945712
$CULC945712$`TUHG6-506`
 epsilonKO     betaKO    gammaKO    alphaKO    deltaKO        bKO        cKO 
"GHG6-930" "GHG6-929" "GHG6-928" "GHG6-927" "GHG6-926" "GHG6-925" "GHG6-924" 
       aKO 
"GHG6-923" 



$CURE504474
$CURE504474$`TUJ8Y-411`
 epsilonKO     betaKO    gammaKO    alphaKO    deltaKO        bKO        cKO 
"GJ8Y-731" "GJ8Y-730" "GJ8Y-729" "GJ8Y-728" "GJ8Y-727" "GJ8Y-726" "GJ8Y-725" 
       aKO 
"GJ8Y-724" 



$CVAR858619
$CVAR858619$`TUHOU-1055`
        aKO         cKO         bKO     deltaKO     alphaKO     gammaKO 
"GHOU-1900" "GHOU-1899" "GHOU-1898" "GHOU-1897" "GHOU-1896" "GHOU-1895" 
     betaKO   epsilonKO 
"GHOU-1894" "GHOU-1893" 



$CVIO243365
$CVIO243365$`TUHUD-373`
 epsilonKO     betaKO    gammaKO    alphaKO    deltaKO        bKO        cKO 
"GHUD-684" "GHUD-683" "GHUD-682" "GHUD-681" "GHUD-680" "GHUD-679" "GHUD-678" 
       aKO 
"GHUD-677" 
```

```
$CWOE469383
$CWOE469383$`TUH82-1971`
        aKO         cKO         bKO     deltaKO     alphaKO     gammaKO 
"GH82-4662" "GH82-4661" "GH82-4660" "GH82-4659" "GH82-4658" "GH82-4657" 
     betaKO   epsilonKO 
"GH82-4656" "GH82-4655" 


$SSP321327
$SSP321327$`TUHFX-1373`
        cKO        bKO2        bKO1     deltaKO     alphaKO     gammaKO 
"GHFX-2109" "GHFX-2108" "GHFX-2107" "GHFX-2106" "GHFX-2105" "GHFX-2104" 

$SSP321327$`TUHFX-264`
    betaKO 
"GHFX-417" 

$SSP321327$`TUHFX-999`
  epsilonKO 
"GHFX-1534" 

$SSP321327$`TUHFX-1374`
        aKO 
"GHFX-2111" 


$MSP490759
$MSP490759$`TULG6-131`
   betaKO1 epsilonKO1       aKO1       cKO1       bKO1   alphaKO1 
"GLG6-237" "GLG6-236" "GLG6-233" "GLG6-232" "GLG6-231" "GLG6-230" 

$MSP490759$`TULG6-2133`
       aKO2        cKO2        bKO2     deltaKO    alphaKO2     gammaKO 
"GLG6-3924" "GLG6-3923" "GLG6-3922" "GLG6-3921" "GLG6-3920" "GLG6-3919" 
    betaKO2  epsilonKO2 
"GLG6-3918" "GLG6-3917" 


$SSP321332
$SSP321332$`TUH1B-1745`
        aKO         cKO        bKO2        bKO1     deltaKO     alphaKO 
"GH1B-2679" "GH1B-2677" "GH1B-2676" "GH1B-2675" "GH1B-2674" "GH1B-2673" 
    gammaKO 
"GH1B-2672" 

$SSP321332$`TUH1B-1974`
     betaKO 
"GH1B-2502" 

$SSP321332$`TUH1B-1451`
  epsilonKO 
"GH1B-2222" 
```

```
$CSP65393
$CSP65393$`TUJP7-2093`
        aKO         cKO        bKO2        bKO1     deltaKO     alphaKO 
"GJP7-2657" "GJP7-2656" "GJP7-2655" "GJP7-2654" "GJP7-2653" "GJP7-2652" 
    gammaKO 
"GJP7-2651" 

$CSP65393$`TUJP7-3775`
     betaKO   epsilonKO 
"GJP7-5146" "GJP7-5145" 


$CSP395962
$CSP395962$`TUJC3-2289`
        aKO         cKO        bKO2        bKO1     deltaKO     alphaKO 
"GJC3-3421" "GJC3-3420" "GJC3-3419" "GJC3-3418" "GJC3-3417" "GJC3-3416" 
    gammaKO 
"GJC3-3415" 

$CSP395962$`TUJC3-2507`
     betaKO   epsilonKO 
"GJC3-3756" "GJC3-3755" 


$LACI272621
$LACI272621$`TUJO8-938`
    alphaKO     deltaKO 
"GJO8-1278" "GJO8-1277" 

$LACI272621$`TUJO8-940`
     betaKO 
"GJO8-1282" 

$LACI272621$`TUJO8-939`
    gammaKO 
"GJO8-1280" 

$LACI272621$`TUJO8-941`
  epsilonKO 
"GJO8-1284" 

$LACI272621$`TUJO8-935`
        cKO 
"GJO8-1273" 

$LACI272621$`TUJO8-934`
        aKO 
"GJO8-1271" 

$LACI272621$`TUJO8-937`
        bKO 
"GJO8-1275" 
```

```
$CSP497965
$CSP497965$`TUJAC-4533`
        aKO         cKO        bKO2        bKO1     deltaKO     alphaKO
"GJAC-5564" "GJAC-5563" "GJAC-5562" "GJAC-5561" "GJAC-5560" "GJAC-5559"
    gammaKO
"GJAC-5558"

$CSP497965$`TUJAC-3560`
     betaKO   epsilonKO
"GJAC-4068" "GJAC-4067"


$CSP395961
$CSP395961$`TUJDE-1057`
    gammaKO     alphaKO     deltaKO        bKO2
"GJDE-1312" "GJDE-1311" "GJDE-1310" "GJDE-1309"

$CSP395961$`TUJDE-3257`
     betaKO   epsilonKO
"GJDE-4863" "GJDE-4862"

$CSP395961$`TUJDE-1055`
        cKO
"GJDE-1307"

$CSP395961$`TUJDE-1054`
        aKO
"GJDE-1306"

$CSP395961$`TUJDE-1056`
       bKO1
"GJDE-1308"


$CSP41431
$CSP41431$`TUHLK-1861`
    gammaKO     alphaKO     deltaKO        bKO2        bKO1
"GHLK-2752" "GHLK-2751" "GHLK-2750" "GHLK-2749" "GHLK-2748"

$CSP41431$`TUHLK-2485`
     betaKO
"GHLK-3704"

$CSP41431$`TUHLK-2484`
  epsilonKO
"GHLK-3703"

$CSP41431$`TUHLK-1860`
        cKO
"GHLK-2747"

$CSP41431$`TUHLK-1859`
        aKO
```

```
"GHLK-2746"


$CSP385025
$CSP385025$`TULCA-1030`
        aKO         cKO         bKO     deltaKO     alphaKO     gammaKO 
"GLCA-2247" "GLCA-2246" "GLCA-2245" "GLCA-2244" "GLCA-2243" "GLCA-2242" 
     betaKO   epsilonKO 
"GLCA-2241" "GLCA-2240" 


$CSP43989
$CSP43989$`TUKC8-1049`
       aKO1        cKO1        bKO1    alphaKO1 
"GKC8-1532" "GKC8-1531" "GKC8-1530" "GKC8-1527" 

$CSP43989$`TUKC8-2970`
   gammaKO2    alphaKO2     deltaKO        bKO3        bKO2        cKO2 
"GKC8-4544" "GKC8-4543" "GKC8-4542" "GKC8-4541" "GKC8-4540" "GKC8-4539" 
       aKO2 
"GKC8-4538" 

$CSP43989$`TUKC8-1050`
    betaKO1  epsilonKO1 
"GKC8-1536" "GKC8-1535" 

$CSP43989$`TUKC8-1881`
    betaKO2  epsilonKO2 
"GKC8-2849" "GKC8-2848" 

$CSP43989$`TUKC8-939`
   gammaKO1 
"GKC8-1366" 


$CUCY713887
$CUCY713887$`TUI71-90`
       aKO        cKO       bKO2       bKO1    deltaKO    alphaKO    gammaKO 
"GI71-129" "GI71-128" "GI71-127" "GI71-126" "GI71-125" "GI71-124" "GI71-123" 

$CUCY713887$`TUI71-180`
    betaKO  epsilonKO 
"GI71-252" "GI71-251" 


$`CZAN1198232-WGS`
$`CZAN1198232-WGS`$`TUSM8-1177`
        aKO         cKO         bKO     deltaKO     alphaKO     gammaKO 
"GSM8-2528" "GSM8-2527" "GSM8-2526" "GSM8-2525" "GSM8-2524" "GSM8-2523" 
     betaKO   epsilonKO 
"GSM8-2522" "GSM8-2521" 


$MBOV561275
```

```
$MBOV561275$`TUHDN-704`
  epsilonKO      betaKO     gammaKO     alphaKO     deltaKO         bKO
"GHDN-1363" "GHDN-1362" "GHDN-1361" "GHDN-1360" "GHDN-1359" "GHDN-1358"
        cKO         aKO
"GHDN-1357" "GHDN-1356"



$DACI398578
$DACI398578$`TUHK3-226`
 epsilonKO     betaKO    gammaKO    alphaKO    deltaKO        bKO        cKO
"GHK3-422" "GHK3-421" "GHK3-420" "GHK3-419" "GHK3-418" "GHK3-417" "GHK3-416"
       aKO
"GHK3-415"



$DACE485916
$DACE485916$`TUHUF-2383`
        aKO         cKO         bKO     deltaKO     alphaKO     gammaKO
"GHUF-4266" "GHUF-4265" "GHUF-4264" "GHUF-4263" "GHUF-4262" "GHUF-4261"
     betaKO   epsilonKO
"GHUF-4260" "GHUF-4259"



$MAER449447
$MAER449447$`TUHO8-3385`
    gammaKO     alphaKO     deltaKO        bKO2        bKO1         cKO
"GHO8-5056" "GHO8-5055" "GHO8-5054" "GHO8-5053" "GHO8-5052" "GHO8-5051"
        aKO
"GHO8-5050"

$MAER449447$`TUHO8-62`
epsilonKO    betaKO
"GHO8-93" "GHO8-92"



$DAFR690850
$DAFR690850$`TUHYW-424`
   betaKO1 epsilonKO1       aKO1       cKO1       bKO1   alphaKO1   gammaKO1
"GHYW-764" "GHYW-763" "GHYW-760" "GHYW-759" "GHYW-758" "GHYW-757" "GHYW-756"

$DAFR690850$`TUHYW-925`
 epsilonKO2     betaKO2    gammaKO2    alphaKO2     deltaKO        bKO3
"GHYW-1602" "GHYW-1601" "GHYW-1600" "GHYW-1599" "GHYW-1598" "GHYW-1597"
       bKO2
"GHYW-1596"

$DAFR690850$`TUHYW-473`
      aKO2       cKO2
"GHYW-838" "GHYW-837"



$DACI646529
$DACI646529$`TULCJ-2828`
        cKO         bKO     deltaKO     alphaKO     gammaKO      betaKO
```

"GLCJ-4618" "GLCJ-4617" "GLCJ-4616" "GLCJ-4615" "GLCJ-4614" "GLCJ-4613"
epsilonKO
"GLCJ-4612"

$DACI646529$`TULCJ-2829`
aKO
"GLCJ-4619"

$DALK589865
$DALK589865$`TUHTX-977`
bKO2 bKO1 deltaKO alphaKO gammaKO betaKO
"GHTX-1942" "GHTX-1941" "GHTX-1940" "GHTX-1939" "GHTX-1938" "GHTX-1937"
epsilonKO
"GHTX-1936"

$DALK589865$`TUHTX-1316`
cKO aKO
"GHTX-2592" "GHTX-2591"

$DALK439235
$DALK439235$`TUHP2-2079`
bKO2 bKO1 deltaKO alphaKO gammaKO betaKO
"GHP2-3680" "GHP2-3679" "GHP2-3678" "GHP2-3677" "GHP2-3676" "GHP2-3675"
epsilonKO
"GHP2-3674"

$DALK439235$`TUHP2-1653`
cKO1 aKO1
"GHP2-2889" "GHP2-2888"

$DALK439235$`TUHP2-2449`
bKO3 cKO2 aKO2
"GHP2-4365" "GHP2-4364" "GHP2-4362"

$DALK439235$`TUHP2-2450`
bKO4
"GHP2-4366"

$DACE880072
$DACE880072$`TUHK9-1460`
epsilonKO betaKO gammaKO alphaKO deltaKO bKO2
"GHK9-2500" "GHK9-2499" "GHK9-2498" "GHK9-2497" "GHK9-2496" "GHK9-2495"
bKO1
"GHK9-2494"

$DACE880072$`TUHK9-1701`
cKO aKO
"GHK9-2940" "GHK9-2939"

$DACE522772

$DACE522772$`TUHC2-387`
epsilonKO betaKO gammaKO alphaKO deltaKO bKO2 bKO1
"GHC2-898" "GHC2-897" "GHC2-896" "GHC2-895" "GHC2-894" "GHC2-893" "GHC2-892"

$DACE522772$`TUHC2-191`
cKO aKO
"GHC2-475" "GHC2-474"

$DARO159087
$DARO159087$`TUI5B-1887`
epsilonKO betaKO gammaKO alphaKO deltaKO bKO
"GI5B-4194" "GI5B-4193" "GI5B-4192" "GI5B-4191" "GI5B-4190" "GI5B-4189"
cKO aKO
"GI5B-4188" "GI5B-4187"

$DAES643562
$DAES643562$`TUH9Z-1627`
bKO2 bKO1 deltaKO alphaKO gammaKO betaKO
"GH9Z-3173" "GH9Z-3172" "GH9Z-3171" "GH9Z-3170" "GH9Z-3169" "GH9Z-3168"
epsilonKO
"GH9Z-3167"

$DAES643562$`TUH9Z-1632`
aKO cKO
"GH9Z-3184" "GH9Z-3183"

$MBOV289397
$MBOV289397$`TUHDH-177`
betaKO1 alphaKO1
"GHDH-384" "GHDH-383"

$MBOV289397$`TUHDH-212`
epsilonKO betaKO2 gammaKO alphaKO2 deltaKO bKO cKO
"GHDH-450" "GHDH-449" "GHDH-448" "GHDH-447" "GHDH-446" "GHDH-445" "GHDH-444"
aKO
"GHDH-443"

$DAUT177437
$DAUT177437$`TUHLR-738`
betaKO1 epsilonKO1 aKO1 cKO1 bKO1 alphaKO1
"GHLR-1339" "GHLR-1338" "GHLR-1335" "GHLR-1334" "GHLR-1333" "GHLR-1332"
gammaKO1
"GHLR-1331"

$DAUT177437$`TUHLR-1818`
bKO3 bKO2 deltaKO alphaKO2 gammaKO2 betaKO2
"GHLR-3520" "GHLR-3519" "GHLR-3518" "GHLR-3517" "GHLR-3516" "GHLR-3515"
epsilonKO2
"GHLR-3514"

$DAUT177437$`TUHLR-962`
aKO2 cKO2
"GHLR-1781" "GHLR-1780"

$MBOV767465
$MBOV767465$`TULH0-220`
aKO cKO bKO deltaKO alphaKO1 gammaKO betaKO1
"GLH0-456" "GLH0-455" "GLH0-454" "GLH0-453" "GLH0-452" "GLH0-451" "GLH0-450"
epsilonKO
"GLH0-449"

$MBOV767465$`TULH0-256`
alphaKO2 betaKO2
"GLH0-521" "GLH0-520"

$DAUD477974
$DAUD477974$`TUH0B-1157`
aKO cKO bKO deltaKO alphaKO gammaKO
"GH0B-2197" "GH0B-2196" "GH0B-2195" "GH0B-2194" "GH0B-2193" "GH0B-2192"
betaKO epsilonKO
"GH0B-2191" "GH0B-2190"

$DBAC525897
$DBAC525897$`TUI50-497`
epsilonKO1 aKO1 cKO1 bKO1 alphaKO1 gammaKO1
"GI50-979" "GI50-976" "GI50-975" "GI50-974" "GI50-973" "GI50-972"

$DBAC525897$`TUI50-1717`
epsilonKO2 betaKO2 gammaKO2 alphaKO2 deltaKO bKO3
"GI50-3433" "GI50-3432" "GI50-3431" "GI50-3430" "GI50-3429" "GI50-3428"

$DBAC525897$`TUI50-498`
betaKO1
"GI50-980"

$DBAC525897$`TUI50-1445`
aKO2 cKO2
"GI50-2853" "GI50-2852"

$DBAC525897$`TUI50-1716`
bKO2
"GI50-3427"

$DBAA644282
$DBAA644282$`TUH2J-145`
epsilonKO betaKO gammaKO alphaKO deltaKO bKO2 bKO1
"GH2J-293" "GH2J-292" "GH2J-291" "GH2J-290" "GH2J-289" "GH2J-288" "GH2J-287"

$DBAA644282$`TUH2J-1272`
cKO aKO

"GH2J-2767" "GH2J-2766"

$DCAR868595
$DCAR868595$`TUHXC-1441`
aKO cKO bKO deltaKO alphaKO gammaKO
"GHXC-2723" "GHXC-2722" "GHXC-2721" "GHXC-2720" "GHXC-2719" "GHXC-2718"
betaKO epsilonKO
"GHXC-2717" "GHXC-2716"

$DDAD579405
$DDAD579405$`TUHJU-2241`
epsilonKO betaKO gammaKO alphaKO deltaKO bKO
"GHJU-4093" "GHJU-4092" "GHJU-4091" "GHJU-4090" "GHJU-4089" "GHJU-4088"
cKO aKO
"GHJU-4087" "GHJU-4086"

$DDAD590409
$DDAD590409$`TUHDW-2335`
epsilonKO betaKO gammaKO alphaKO deltaKO bKO
"GHDW-4263" "GHDW-4262" "GHDW-4261" "GHDW-4260" "GHDW-4259" "GHDW-4258"
cKO aKO
"GHDW-4257" "GHDW-4256"

$DDAD198628
$DDAD198628$`TUHFQ-2593`
aKO cKO bKO deltaKO alphaKO gammaKO
"GHFQ-4684" "GHFQ-4683" "GHFQ-4682" "GHFQ-4681" "GHFQ-4680" "GHFQ-4679"
betaKO epsilonKO
"GHFQ-4678" "GHFQ-4677"

$DALA207559
$DALA207559$`TUH1L-442`
bKO2 bKO1 deltaKO alphaKO gammaKO betaKO epsilonKO
"GH1L-852" "GH1L-851" "GH1L-850" "GH1L-849" "GH1L-848" "GH1L-847" "GH1L-846"

$DALA207559$`TUH1L-1268`
cKO aKO
"GH1L-2417" "GH1L-2416"

$DDEH756499
$DDEH756499$`TULCG-2260`
cKO bKO deltaKO alphaKO gammaKO betaKO
"GLCG-4095" "GLCG-4094" "GLCG-4093" "GLCG-4092" "GLCG-4091" "GLCG-4090"
epsilonKO
"GLCG-4089"

$DDEH756499$`TULCG-2261`
aKO

"GLCG-4096"

$MCAP243233
$MCAP243233$`TUCH7-4413`
epsilonKO1 betaKO1 gammaKO1 alphaKO1 deltaKO bKO1 cKO1
"GCH7-246" "GCH7-245" "GCH7-244" "GCH7-243" "GCH7-242" "GCH7-241" "GCH7-240"
aKO1
"GCH7-239"

$MCAP243233$`TUCH7-5669`
gammaKO2 alphaKO2
"GCH7-2841" "GCH7-2840"

$MCAP243233$`TUCH7-5130`
epsilonKO2 betaKO2
"GCH7-1732" "GCH7-1731"

$MCAP243233$`TUCH7-5815`
epsilonKO3
"GCH7-3139"

$MCAP243233$`TUCH7-5666`
bKO2 cKO2 aKO2
"GCH7-2836" "GCH7-2835" "GCH7-2834"

$`MCON572263-WGS`
$`MCON572263-WGS`$`TUSRN-163`
betaKO1 alphaKO1
"GSRN-345" "GSRN-344"

$`MCON572263-WGS`$`TUSRN-308`
aKO cKO bKO deltaKO alphaKO2 gammaKO betaKO2
"GSRN-648" "GSRN-647" "GSRN-646" "GSRN-645" "GSRN-644" "GSRN-643" "GSRN-642"
epsilonKO
"GSRN-641"

$`DDIC871963-WGS`
$`DDIC871963-WGS`$`TUSMV-1905`
aKO cKO bKO deltaKO alphaKO gammaKO
"GSMV-3434" "GSMV-3433" "GSMV-3432" "GSMV-3431" "GSMV-3430" "GSMV-3429"
betaKO epsilonKO
"GSMV-3428" "GSMV-3427"

$DDES641491
$DDES641491$`TUH21-1360`
bKO2 bKO1 deltaKO alphaKO gammaKO betaKO
"GH21-2618" "GH21-2617" "GH21-2616" "GH21-2615" "GH21-2614" "GH21-2613"
epsilonKO
"GH21-2612"

```
$DDES641491$`TUH21-1325`
       aKO        cKO 
"GH21-2553" "GH21-2552" 


$ASP404589
$ASP404589$`TUHMT-2377`
    deltaKO     alphaKO     gammaKO      betaKO   epsilonKO 
"GHMT-4552" "GHMT-4551" "GHMT-4550" "GHMT-4549" "GHMT-4548" 

$ASP404589$`TUHMT-2376`
        aKO         cKO         bKO 
"GHMT-4541" "GHMT-4540" "GHMT-4539" 


$AHYD380703
$AHYD380703$`TUH2M-2382`
        aKO         cKO         bKO     deltaKO     alphaKO     gammaKO 
"GH2M-4268" "GH2M-4267" "GH2M-4266" "GH2M-4265" "GH2M-4264" "GH2M-4263" 
     betaKO   epsilonKO 
"GH2M-4262" "GH2M-4261" 


$AHAE644284
$AHAE644284$`TUI54-174`
 epsilonKO     betaKO    gammaKO    alphaKO    deltaKO        bKO        cKO 
"GI54-378" "GI54-377" "GI54-376" "GI54-375" "GI54-374" "GI54-373" "GI54-372" 
       aKO 
"GI54-371" 


$DDES525146
$DDES525146$`TUIWF-1303`
  epsilonKO      betaKO     gammaKO     alphaKO     deltaKO        bKO2 
"GIWF-2255" "GIWF-2254" "GIWF-2253" "GIWF-2252" "GIWF-2251" "GIWF-2250" 

$DDES525146$`TUIWF-1344`
        cKO         aKO 
"GIWF-2322" "GIWF-2321" 

$DDES525146$`TUIWF-1302`
       bKO1 
"GIWF-2249" 


$DSP216389
$DSP216389$`TUH6D-257`
 epsilonKO     betaKO    gammaKO    alphaKO    deltaKO        bKO        cKO 
"GH6D-547" "GH6D-546" "GH6D-545" "GH6D-544" "GH6D-543" "GH6D-542" "GH6D-541" 
       aKO 
"GH6D-540" 


$DSP1131462
```

$DSP1131462$`TULCD-1670`
aKO cKO bKO deltaKO alphaKO gammaKO
"GLCD-2937" "GLCD-2936" "GLCD-2935" "GLCD-2934" "GLCD-2933" "GLCD-2932"
betaKO epsilonKO
"GLCD-2931" "GLCD-2930"

$DSP1147129
$DSP1147129$`TULCC-1657`
aKO cKO bKO deltaKO alphaKO gammaKO
"GLCC-2936" "GLCC-2935" "GLCC-2934" "GLCC-2933" "GLCC-2932" "GLCC-2931"
betaKO epsilonKO
"GLCC-2930" "GLCC-2929"

$DSP633145
$DSP633145$`TUHAK-223`
epsilonKO betaKO gammaKO alphaKO deltaKO bKO cKO
"GHAK-509" "GHAK-508" "GHAK-507" "GHAK-506" "GHAK-505" "GHAK-504" "GHAK-503"
aKO
"GHAK-502"

$DDES639282
$DDES639282$`TUJ90-748`
bKO2 bKO1 deltaKO alphaKO gammaKO betaKO
"GJ90-1886" "GJ90-1885" "GJ90-1884" "GJ90-1883" "GJ90-1882" "GJ90-1881"
epsilonKO
"GJ90-1880"

$DDES639282$`TUJ90-707`
aKO cKO
"GJ90-1724" "GJ90-1723"

$MGAL1159203
$MGAL1159203$`TULH1-143`
epsilonKO betaKO gammaKO alphaKO1 deltaKO bKO cKO
"GLH1-341" "GLH1-340" "GLH1-339" "GLH1-338" "GLH1-337" "GLH1-336" "GLH1-335"
aKO
"GLH1-334"

$MGAL1159203$`TULH1-330`
alphaKO2
"GLH1-727"

$MCHU710421
$MCHU710421$`TULGL-2073`
aKO cKO bKO alphaKO gammaKO betaKO
"GLGL-3772" "GLGL-3771" "GLGL-3770" "GLGL-3768" "GLGL-3767" "GLGL-3766"
epsilonKO
"GLGL-3765"

```
$MCHU710421$noTU
deltaKO
     NA



$DSP255470
$DSP255470$`TUJXW-197`
 epsilonKO     betaKO    gammaKO    alphaKO    deltaKO        bKO        cKO
"GJXW-456" "GJXW-455" "GJXW-454" "GJXW-453" "GJXW-452" "GJXW-451" "GJXW-450"
       aKO
"GJXW-449"



$DSP742013
$DSP742013$`TUH2F-238`
 epsilonKO     betaKO    gammaKO    alphaKO    deltaKO        bKO        cKO
"GH2F-435" "GH2F-434" "GH2F-433" "GH2F-432" "GH2F-431" "GH2F-430" "GH2F-429"
       aKO
"GH2F-428"



$DETH243164
$DETH243164$`TUJNF-251`
 epsilonKO     betaKO    gammaKO    alphaKO    deltaKO        bKO        cKO
"GJNF-565" "GJNF-564" "GJNF-563" "GJNF-562" "GJNF-561" "GJNF-560" "GJNF-559"
       aKO
"GJNF-558"



$DSP311424
$DSP311424$`TUJ8J-213`
 epsilonKO     betaKO    gammaKO    alphaKO    deltaKO        bKO        cKO
"GJ8J-486" "GJ8J-485" "GJ8J-484" "GJ8J-483" "GJ8J-482" "GJ8J-481" "GJ8J-480"
       aKO
"GJ8J-479"



$DFER471854
$DFER471854$`TUI24-2755`
    alphaKO     gammaKO
"GI24-4674" "GI24-4673"

$DFER471854$`TUI24-2221`
  epsilonKO      betaKO
"GI24-3785" "GI24-3784"

$DFER471854$`TUI24-3001`
        bKO     deltaKO
"GI24-5065" "GI24-5064"

$DFER471854$`TUI24-3002`
        aKO
"GI24-5067"
```

```
$DFER471854$noTU
cKO
 NA


$`DGIG1121448-WGS`
$`DGIG1121448-WGS`$`TUSMJ-338`
      bKO2       bKO1    deltaKO    alphaKO    gammaKO     betaKO  epsilonKO
"GSMJ-636" "GSMJ-635" "GSMJ-634" "GSMJ-633" "GSMJ-632" "GSMJ-631" "GSMJ-630"

$`DGIG1121448-WGS`$`TUSMJ-740`
        cKO         aKO
"GSMJ-1443" "GSMJ-1442"


$`DGIB767817-WGS`
$`DGIB767817-WGS`$`TUSMI-2429`
       aKO1        cKO1        bKO1    alphaKO1    gammaKO1
"GSMI-4149" "GSMI-4148" "GSMI-4147" "GSMI-4146" "GSMI-4145"

$`DGIB767817-WGS`$`TUSMI-2671`
       aKO2        cKO2        bKO2     deltaKO    alphaKO2    gammaKO2
"GSMI-4585" "GSMI-4584" "GSMI-4583" "GSMI-4582" "GSMI-4581" "GSMI-4580"
    betaKO2  epsilonKO2
"GSMI-4579" "GSMI-4578"

$`DGIB767817-WGS`$`TUSMI-2430`
    betaKO1  epsilonKO1
"GSMI-4153" "GSMI-4152"


$DHAF272564
$DHAF272564$`TUCV8-2746`
        aKO         cKO         bKO     deltaKO     alphaKO     gammaKO
"GCV8-4883" "GCV8-4882" "GCV8-4881" "GCV8-4880" "GCV8-4879" "GCV8-4878"
     betaKO   epsilonKO
"GCV8-4877" "GCV8-4876"


$AEBR535289
$AEBR535289$`TUHOO-175`
 epsilonKO     betaKO    gammaKO    alphaKO    deltaKO        bKO        cKO
"GHOO-304" "GHOO-303" "GHOO-302" "GHOO-301" "GHOO-300" "GHOO-299" "GHOO-298"
       aKO
"GHOO-297"


$MGAL1159204
$MGAL1159204$`TULH4-142`
 epsilonKO     betaKO    gammaKO   alphaKO1    deltaKO        bKO        cKO
"GLH4-340" "GLH4-339" "GLH4-338" "GLH4-337" "GLH4-336" "GLH4-335" "GLH4-334"
       aKO
"GLH4-333"
```

```
$MGAL1159204$`TULH4-313`
  alphaKO2
"GLH4-711"



$DIND653733
$DIND653733$`TUHGZ-1081`
  epsilonKO      betaKO     gammaKO     alphaKO     deltaKO        bKO2
"GHGZ-2298" "GHGZ-2297" "GHGZ-2296" "GHGZ-2295" "GHGZ-2294" "GHGZ-2293"
       bKO1
"GHGZ-2292"

$DIND653733$`TUHGZ-349`
       cKO        aKO
"GHGZ-772" "GHGZ-771"



$MCRO512564
$MCRO512564$`TUI47-55`
  alphaKO1    betaKO1
"GI47-102" "GI47-101"

$MCRO512564$`TUI47-177`
       aKO        cKO        bKO    deltaKO   alphaKO2    gammaKO    betaKO2
"GI47-366" "GI47-365" "GI47-364" "GI47-363" "GI47-362" "GI47-361" "GI47-360"
 epsilonKO
"GI47-359"



$DKUZ760568
$DKUZ760568$`TUHV4-1869`
        aKO         cKO         bKO     deltaKO     alphaKO     gammaKO
"GHV4-3481" "GHV4-3480" "GHV4-3479" "GHV4-3478" "GHV4-3477" "GHV4-3476"
     betaKO   epsilonKO
"GHV4-3475" "GHV4-3474"



$DLYK552811
$DLYK552811$`TUH8P-283`
 epsilonKO     betaKO    gammaKO    alphaKO    deltaKO        bKO        cKO
"GH8P-569" "GH8P-568" "GH8P-567" "GH8P-566" "GH8P-565" "GH8P-564" "GH8P-563"
       aKO
"GH8P-562"



$`DMCC1193806-WGS`
$`DMCC1193806-WGS`$`TUSMA-222`
 epsilonKO     betaKO    gammaKO    alphaKO    deltaKO        bKO        cKO
"GSMA-526" "GSMA-525" "GSMA-524" "GSMA-523" "GSMA-522" "GSMA-521" "GSMA-520"
       aKO
"GSMA-519"



$`DMCC1193807-WGS`
```

```
$`DMCC1193807-WGS`$`TUSMB-249`
 epsilonKO     betaKO    gammaKO    alphaKO    deltaKO        bKO        cKO
"GSMB-571" "GSMB-570" "GSMB-569" "GSMB-568" "GSMB-567" "GSMB-566" "GSMB-565"
       aKO
"GSMB-564"


$`DMCC1388758-WGS`
$`DMCC1388758-WGS`$`TUSMC-214`
 epsilonKO     betaKO    gammaKO    alphaKO    deltaKO        bKO        cKO
"GSMC-498" "GSMC-497" "GSMC-496" "GSMC-495" "GSMC-494" "GSMC-493" "GSMC-492"
       aKO
"GSMC-491"


$DMER768704
$DMER768704$`TULCK-2648`
        cKO         bKO     deltaKO     alphaKO     gammaKO      betaKO
"GLCK-4556" "GLCK-4555" "GLCK-4554" "GLCK-4553" "GLCK-4552" "GLCK-4551"
  epsilonKO
"GLCK-4550"

$DMER768704$`TULCK-2649`
        aKO
"GLCK-4557"


$DNOD246195
$DNOD246195$`TUHHS-491`
        aKO         cKO         bKO     deltaKO     alphaKO     gammaKO
"GHHS-1145" "GHHS-1144" "GHHS-1143" "GHHS-1142" "GHHS-1141" "GHHS-1140"
     betaKO   epsilonKO
"GHHS-1139" "GHHS-1138"


$DOLE96561
$DOLE96561$`TUHF3-343`
 epsilonKO     betaKO    gammaKO    alphaKO    deltaKO       bKO2       bKO1
"GHF3-614" "GHF3-613" "GHF3-612" "GHF3-611" "GHF3-610" "GHF3-609" "GHF3-608"

$DOLE96561$`TUHF3-359`
      cKO1       aKO1
"GHF3-646" "GHF3-645"

$DOLE96561$`TUHF3-461`
      cKO2       aKO2
"GHF3-823" "GHF3-822"

$DOLE96561$`TUHF3-462`
      bKO4       bKO3
"GHF3-825" "GHF3-824"


$LLON661367
```

```
$LLON661367$`TUJAR-2150`
       aKO        cKO        bKO     deltaKO     alphaKO     gammaKO 
"GJAR-3677" "GJAR-3676" "GJAR-3675" "GJAR-3674" "GJAR-3673" "GJAR-3672" 
     betaKO   epsilonKO 
"GJAR-3671" "GJAR-3670" 


$DORI768706
$DORI768706$`TUHQZ-3085`
       cKO        bKO     deltaKO     alphaKO     gammaKO      betaKO 
"GHQZ-5514" "GHQZ-5513" "GHQZ-5512" "GHQZ-5511" "GHQZ-5510" "GHQZ-5509" 
  epsilonKO 
"GHQZ-5508" 

$DORI768706$`TUHQZ-3086`
       aKO 
"GHQZ-5515" 


$`DPRO673862-WGS`
$`DPRO673862-WGS`$`TUSMD-480`
   alphaKO    deltaKO        bKO        cKO 
"GSMD-845" "GSMD-844" "GSMD-842" "GSMD-841" 

$`DPRO673862-WGS`$`TUSMD-385`
 epsilonKO     betaKO 
"GSMD-690" "GSMD-689" 

$`DPRO673862-WGS`$`TUSMD-245`
   gammaKO 
"GSMD-460" 

$`DPRO673862-WGS`$`TUSMD-160`
       aKO 
"GSMD-274" 


$MCAN1048245
$MCAN1048245$`TUJCJ-712`
  epsilonKO      betaKO     gammaKO     alphaKO 
"GJCJ-1337" "GJCJ-1336" "GJCJ-1335" "GJCJ-1334" 

$MCAN1048245$`TUJCJ-711`
       bKO         cKO 
"GJCJ-1332" "GJCJ-1331" 

$MCAN1048245$`TUJCJ-710`
       aKO 
"GJCJ-1330" 

$MCAN1048245$noTU
deltaKO 
     NA 
```

```
$`DPIE1322246-WGS`
$`DPIE1322246-WGS`$`TUSML-1365`
  epsilonKO      betaKO     gammaKO     alphaKO     deltaKO        bKO2
"GSML-2580" "GSML-2579" "GSML-2578" "GSML-2577" "GSML-2576" "GSML-2575"
       bKO1
"GSML-2574"

$`DPIE1322246-WGS`$`TUSML-1380`
        cKO         aKO
"GSML-2606" "GSML-2605"


$DPRO577650
$DPRO577650$`TUH80-698`
   gammaKO1    alphaKO1        bKO1        cKO1        aKO1  epsilonKO1
"GH80-1290" "GH80-1289" "GH80-1288" "GH80-1287" "GH80-1286" "GH80-1283"
    betaKO1
"GH80-1282"

$DPRO577650$`TUH80-1458`
       bKO3        bKO2     deltaKO    alphaKO2    gammaKO2     betaKO2
"GH80-2702" "GH80-2701" "GH80-2700" "GH80-2699" "GH80-2698" "GH80-2697"
 epsilonKO2
"GH80-2696"

$DPRO577650$`TUH80-1620`
       cKO2        aKO2
"GH80-3012" "GH80-3011"


$DPSY177439
$DPSY177439$`TUJW5-548`
    betaKO    gammaKO    alphaKO    deltaKO       bKO2       bKO1
"GJW5-861" "GJW5-860" "GJW5-859" "GJW5-858" "GJW5-857" "GJW5-856"

$DPSY177439$`TUJW5-549`
 epsilonKO
"GJW5-862"

$DPSY177439$`TUJW5-539`
       cKO
"GJW5-843"

$DPSY177439$`TUJW5-538`
       aKO
"GJW5-842"


$DRED349161
$DRED349161$`TUHP6-1787`
        aKO         cKO         bKO     deltaKO     alphaKO
"GHP6-3245" "GHP6-3244" "GHP6-3243" "GHP6-3242" "GHP6-3241"
```

```
$DRED349161$`TUHP6-1786`
    gammaKO      betaKO   epsilonKO
"GHP6-3240" "GHP6-3239" "GHP6-3238"


$DRET485915
$DRET485915$`TUHRJ-1365`
       bKO2        bKO1     deltaKO     alphaKO     gammaKO      betaKO
"GHRJ-2272" "GHRJ-2271" "GHRJ-2270" "GHRJ-2269" "GHRJ-2268" "GHRJ-2267"
  epsilonKO
"GHRJ-2266"

$DRET485915$`TUHRJ-1299`
        aKO         cKO
"GHRJ-2135" "GHRJ-2134"


$DRUM696281
$DRUM696281$`TUCNL-7154`
        aKO         cKO         bKO     deltaKO     alphaKO     gammaKO
"GCNL-3954" "GCNL-3953" "GCNL-3952" "GCNL-3951" "GCNL-3950" "GCNL-3949"
     betaKO   epsilonKO
"GCNL-3948" "GCNL-3947"


$DSAL526222
$DSAL526222$`TUHES-1185`
   alphaKO1    gammaKO1
"GHES-2274" "GHES-2273"

$DSAL526222$`TUHES-1883`
       bKO2     deltaKO    alphaKO2    gammaKO2     betaKO2  epsilonKO2
"GHES-3563" "GHES-3562" "GHES-3561" "GHES-3560" "GHES-3559" "GHES-3558"

$DSAL526222$`TUHES-1186`
    betaKO1  epsilonKO1        aKO1        cKO1        bKO1
"GHES-2281" "GHES-2280" "GHES-2277" "GHES-2276" "GHES-2275"

$DSAL526222$`TUHES-2034`
       aKO2        cKO2
"GHES-3831" "GHES-3830"

$DSAL526222$`TUHES-1884`
       bKO3
"GHES-3564"


$MGAL1159202
$MGAL1159202$`TULH3-145`
 epsilonKO     betaKO    gammaKO   alphaKO1    deltaKO        bKO        cKO
"GLH3-343" "GLH3-342" "GLH3-341" "GLH3-340" "GLH3-339" "GLH3-338" "GLH3-337"
       aKO
"GLH3-336"
```

```
$MGAL1159202$`TULH3-319`
  alphaKO2
"GLH3-716"


$`DSUL1167006-WGS`
$`DSUL1167006-WGS`$`TUSMH-551`
   betaKO1 epsilonKO1       aKO1       cKO1       bKO1   alphaKO1   gammaKO1
"GSMH-974" "GSMH-973" "GSMH-970" "GSMH-969" "GSMH-968" "GSMH-967" "GSMH-966"

$`DSUL1167006-WGS`$`TUSMH-926`
 epsilonKO2     betaKO2    gammaKO2    alphaKO2     deltaKO        bKO3
"GSMH-1699" "GSMH-1698" "GSMH-1697" "GSMH-1696" "GSMH-1695" "GSMH-1694"
       bKO2
"GSMH-1693"

$`DSUL1167006-WGS`$`TUSMH-904`
       cKO2        aKO2
"GSMH-1657" "GSMH-1656"


$DSHI398580
$DSHI398580$`TUKEL-540`
  gammaKO1   alphaKO1       bKO1       cKO1       aKO1 epsilonKO1    betaKO1
"GKEL-451" "GKEL-450" "GKEL-449" "GKEL-448" "GKEL-447" "GKEL-444" "GKEL-443"

$DSHI398580$`TUKEL-1834`
    deltaKO    alphaKO2    gammaKO2     betaKO2  epsilonKO2
"GKEL-2975" "GKEL-2974" "GKEL-2973" "GKEL-2972" "GKEL-2971"

$DSHI398580$`TUKEL-1889`
       aKO2        cKO2        bKO3        bKO2
"GKEL-3069" "GKEL-3068" "GKEL-3067" "GKEL-3066"


$DSAL13035
$DSAL13035$`TULCB-2142`
        cKO        bKO2        bKO1     deltaKO     alphaKO     gammaKO
"GLCB-3583" "GLCB-3582" "GLCB-3581" "GLCB-3580" "GLCB-3579" "GLCB-3578"

$DSAL13035$`TULCB-2109`
  epsilonKO      betaKO
"GLCB-3521" "GLCB-3520"

$DSAL13035$`TULCB-2143`
        aKO
"GLCB-3584"


$MCHL440085
$MCHL440085$`TUCXT-1260`
    deltaKO     alphaKO
"GCXT-1793" "GCXT-1792"
```

```
$MCHL440085$`TUCXT-1259`
    gammaKO      betaKO   epsilonKO 
"GCXT-1791" "GCXT-1790" "GCXT-1788" 

$MCHL440085$`TUCXT-2351`
        aKO         cKO 
"GCXT-3561" "GCXT-3560" 

$MCHL440085$`TUCXT-2349`
       bKO1 
"GCXT-3558" 

$MCHL440085$`TUCXT-2350`
       bKO2 
"GCXT-3559" 


$AORY640081
$AORY640081$`TUHAS-413`
 epsilonKO     betaKO    gammaKO    alphaKO    deltaKO        bKO        cKO 
"GHAS-858" "GHAS-857" "GHAS-856" "GHAS-855" "GHAS-854" "GHAS-853" "GHAS-852" 

$AORY640081$`TUHAS-412`
       aKO 
"GHAS-851" 


$DHAF138119
$DHAF138119$`TUHT5-2996`
        aKO         cKO         bKO     deltaKO     alphaKO     gammaKO 
"GHT5-4992" "GHT5-4991" "GHT5-4990" "GHT5-4989" "GHT5-4988" "GHT5-4987" 
     betaKO   epsilonKO 
"GHT5-4986" "GHT5-4985" 


$DTHE868864
$DTHE868864$`TUHGF-439`
  epsilonKO      betaKO     gammaKO     alphaKO     deltaKO        bKO2 
"GHGF-1289" "GHGF-1288" "GHGF-1287" "GHGF-1286" "GHGF-1285" "GHGF-1284" 
       bKO1 
"GHGF-1283" 

$DTHE868864$`TUHGF-498`
        aKO         cKO 
"GHGF-1448" "GHGF-1447" 


$DTHE309799
$DTHE309799$`TUHF9-611`
        aKO         cKO         bKO     alphaKO     gammaKO      betaKO 
"GHF9-1845" "GHF9-1844" "GHF9-1843" "GHF9-1842" "GHF9-1841" "GHF9-1840" 
  epsilonKO 
"GHF9-1839" 
```

```
$DTHE309799$noTU
deltaKO
     NA


$`AHYD1288394-WGS`
$`AHYD1288394-WGS`$`TUSEU-2622`
        aKO         cKO         bKO     deltaKO     alphaKO     gammaKO 
"GSEU-4558" "GSEU-4557" "GSEU-4556" "GSEU-4555" "GSEU-4554" "GSEU-4553" 
     betaKO   epsilonKO 
"GSEU-4552" "GSEU-4551" 


$AINT568816
$AINT568816$`TUHMB-827`
        aKO         cKO         bKO     alphaKO     gammaKO      betaKO 
"GHMB-1785" "GHMB-1784" "GHMB-1783" "GHMB-1782" "GHMB-1781" "GHMB-1780" 
  epsilonKO 
"GHMB-1779" 

$AINT568816$noTU
deltaKO
     NA


$MGAL1159199
$MGAL1159199$`TULH6-147`
 epsilonKO     betaKO    gammaKO    alphaKO    deltaKO        bKO        cKO 
"GLH6-347" "GLH6-346" "GLH6-345" "GLH6-344" "GLH6-343" "GLH6-342" "GLH6-341" 
       aKO 
"GLH6-340" 


$ASP232721
$ASP232721$`TUHWE-231`
 epsilonKO     betaKO    gammaKO    alphaKO    deltaKO        bKO        cKO 
"GHWE-309" "GHWE-308" "GHWE-307" "GHWE-306" "GHWE-305" "GHWE-304" "GHWE-303" 
       aKO 
"GHWE-302" 


$AKAS1036672
$AKAS1036672$`TUL7X-449`
 epsilonKO     betaKO    gammaKO    alphaKO    deltaKO        bKO        cKO 
"GL7X-753" "GL7X-752" "GL7X-751" "GL7X-749" "GL7X-748" "GL7X-747" "GL7X-746" 
       aKO 
"GL7X-745" 


$DTIE706587
$DTIE706587$`TULCI-2291`
       bKO2        bKO1     deltaKO     alphaKO     gammaKO      betaKO 
"GLCI-3707" "GLCI-3706" "GLCI-3705" "GLCI-3704" "GLCI-3703" "GLCI-3702" 
  epsilonKO 
```

"GLCI-3701"

$DTIE706587$`TULCI-787`
cKO aKO
"GLCI-1249" "GLCI-1248"

$DTOL651182
$DTOL651182$`TULCH-1082`
epsilonKO betaKO gammaKO alphaKO deltaKO bKO2
"GLCH-1990" "GLCH-1989" "GLCH-1988" "GLCH-1987" "GLCH-1986" "GLCH-1985"
bKO1
"GLCH-1984"

$DTOL651182$`TULCH-1009`
cKO aKO
"GLCH-1853" "GLCH-1852"

$APAR521095
$APAR521095$`TUH57-686`
aKO cKO bKO deltaKO alphaKO gammaKO
"GH57-1350" "GH57-1349" "GH57-1348" "GH57-1347" "GH57-1346" "GH57-1345"
betaKO epsilonKO
"GH57-1344" "GH57-1343"

$DTUR515635
$DTUR515635$`TUH4F-52`
aKO cKO bKO alphaKO gammaKO betaKO epsilonKO
"GH4F-135" "GH4F-134" "GH4F-133" "GH4F-132" "GH4F-131" "GH4F-130" "GH4F-129"

$DTUR515635$noTU
deltaKO
NA

$DVUL573059
$DVUL573059$`TULCM-487`
bKO2 bKO1 deltaKO alphaKO gammaKO betaKO epsilonKO
"GLCM-742" "GLCM-741" "GLCM-740" "GLCM-739" "GLCM-738" "GLCM-737" "GLCM-736"

$DVUL573059$`TULCM-557`
aKO cKO
"GLCM-872" "GLCM-871"

$DVUL391774
$DVUL391774$`TUHS0-1264`
epsilonKO betaKO gammaKO alphaKO deltaKO bKO2
"GHS0-2259" "GHS0-2258" "GHS0-2257" "GHS0-2256" "GHS0-2255" "GHS0-2254"
bKO1
"GHS0-2253"

```
$DVUL391774$`TUHS0-1196`
       cKO 
"GHS0-2127" 

$DVUL391774$`TUHS0-1195`
       aKO 
"GHS0-2126" 


$DVUL883
$DVUL883$`TUCJ5-1851`
  epsilonKO      betaKO     gammaKO     alphaKO     deltaKO        bKO2 
"GCJ5-2907" "GCJ5-2906" "GCJ5-2905" "GCJ5-2904" "GCJ5-2903" "GCJ5-2902" 
       bKO1 
"GCJ5-2901" 

$DVUL883$`TUCJ5-900`
       cKO         aKO 
"GCJ5-1427" "GCJ5-1426" 


$DVUL882
$DVUL882$`TUJIL-512`
     bKO2       bKO1    deltaKO    alphaKO    gammaKO     betaKO  epsilonKO 
"GJIL-803" "GJIL-802" "GJIL-801" "GJIL-800" "GJIL-799" "GJIL-798" "GJIL-797" 

$DVUL882$`TUJIL-586`
       aKO        cKO 
"GJIL-942" "GJIL-941" 


$MGAL1159198
$MGAL1159198$`TULH5-147`
 epsilonKO     betaKO    gammaKO   alphaKO1    deltaKO        bKO        cKO 
"GLH5-347" "GLH5-346" "GLH5-345" "GLH5-344" "GLH5-343" "GLH5-342" "GLH5-341" 
       aKO 
"GLH5-340" 

$MGAL1159198$`TULH5-320`
  alphaKO2 
"GLH5-718" 


$DZEA561229
$DZEA561229$`TUJ85-2360`
  epsilonKO      betaKO     gammaKO     alphaKO     deltaKO         bKO 
"GJ85-4296" "GJ85-4295" "GJ85-4294" "GJ85-4293" "GJ85-4292" "GJ85-4291" 
       cKO         aKO 
"GJ85-4290" "GJ85-4289" 


$ECOL655817
$ECOL655817$`TUI9N-2124`
       aKO         cKO         bKO     deltaKO     alphaKO     gammaKO 
```

"GI9N-4108" "GI9N-4107" "GI9N-4106" "GI9N-4105" "GI9N-4104" "GI9N-4103"
betaKO epsilonKO
"GI9N-4102" "GI9N-4101"

$EAER1028307
$EAER1028307$`TUHNA-772`
aKO cKO bKO deltaKO alphaKO gammaKO
"GHNA-1453" "GHNA-1452" "GHNA-1451" "GHNA-1450" "GHNA-1449" "GHNA-1448"
betaKO epsilonKO
"GHNA-1447" "GHNA-1446"

$EAMY665029
$EAMY665029$`TUCM3-24`
aKO cKO bKO deltaKO alphaKO
"GCM3-3773" "GCM3-3772" "GCM3-3771" "GCM3-3770" "GCM3-3769"

$EAMY665029$`TUCM3-22`
betaKO
"GCM3-3767"

$EAMY665029$`TUCM3-23`
gammaKO
"GCM3-3768"

$EAMY665029$`TUCM3-21`
epsilonKO
"GCM3-3766"

$EANT1087448
$EANT1087448$`TULD8-1313`
cKO bKO deltaKO alphaKO gammaKO betaKO
"GLD8-2561" "GLD8-2560" "GLD8-2559" "GLD8-2558" "GLD8-2557" "GLD8-2556"
epsilonKO
"GLD8-2555"

$EANT1087448$`TULD8-1314`
aKO
"GLD8-2562"

$APRO744985
$APRO744985$`TUL7Q-82`
deltaKO alphaKO gammaKO betaKO epsilonKO
"GL7Q-199" "GL7Q-198" "GL7Q-197" "GL7Q-196" "GL7Q-195"

$APRO744985$`TUL7Q-284`
aKO cKO bKO2 bKO1
"GL7Q-768" "GL7Q-767" "GL7Q-766" "GL7Q-765"

$APAS634458

```
$APAS634458$`TUL78-294`
 epsilonKO     betaKO    gammaKO    alphaKO    deltaKO
"GL78-121" "GL78-120" "GL78-119" "GL78-118" "GL78-117"

$APAS634458$`TUL78-1476`
       aKO        cKO
"GL78-2396" "GL78-2395"

$APAS634458$`TUL78-1475`
      bKO2        bKO1
"GL78-2394" "GL78-2393"


$`EAER935296-WGS`
$`EAER935296-WGS`$`TUSMP-2068`
  epsilonKO      betaKO     gammaKO     alphaKO     deltaKO         bKO
"GSMP-3865" "GSMP-3864" "GSMP-3863" "GSMP-3862" "GSMP-3861" "GSMP-3860"
       cKO         aKO
"GSMP-3859" "GSMP-3858"


$EASB640513
$EASB640513$`TUKDM-2417`
  epsilonKO      betaKO     gammaKO     alphaKO     deltaKO         bKO
"GKDM-4550" "GKDM-4549" "GKDM-4548" "GKDM-4547" "GKDM-4546" "GKDM-4545"
       cKO         aKO
"GKDM-4544" "GKDM-4543"


$ESP360911
$ESP360911$`TUI4R-705`
       aKO        cKO        bKO     deltaKO     alphaKO     gammaKO
"GI4R-1321" "GI4R-1320" "GI4R-1319" "GI4R-1318" "GI4R-1317" "GI4R-1316"
    betaKO   epsilonKO
"GI4R-1315" "GI4R-1314"


$MGAL1159200
$MGAL1159200$`TULH8-150`
 epsilonKO     betaKO    gammaKO   alphaKO1    deltaKO       bKO       cKO
"GLH8-349" "GLH8-348" "GLH8-347" "GLH8-346" "GLH8-345" "GLH8-344" "GLH8-343"
      aKO
"GLH8-342"

$MGAL1159200$`TULH8-326`
  alphaKO2
"GLH8-724"


$EAMY716540
$EAMY716540$`TUJAV-68`
       aKO        cKO        bKO     deltaKO     alphaKO     gammaKO
"GJAV-3581" "GJAV-3580" "GJAV-3579" "GJAV-3578" "GJAV-3577" "GJAV-3576"
    betaKO   epsilonKO
```

```
"GJAV-3575" "GJAV-3574"


$AARO76114
$AARO76114$`TUJTA-1110`
  epsilonKO      betaKO     gammaKO     alphaKO     deltaKO         bKO 
"GJTA-1720" "GJTA-1719" "GJTA-1718" "GJTA-1717" "GJTA-1716" "GJTA-1715" 
        cKO         aKO 
"GJTA-1714" "GJTA-1713" 


$ECOL866768
$ECOL866768$`TUHSD-2313`
  epsilonKO      betaKO     gammaKO     alphaKO     deltaKO         bKO 
"GHSD-4397" "GHSD-4396" "GHSD-4395" "GHSD-4394" "GHSD-4393" "GHSD-4392" 
        cKO         aKO 
"GHSD-4391" "GHSD-4390" 


$ECOL469008
$ECOL469008$`TUIYE-1959`
        aKO         cKO         bKO     deltaKO     alphaKO     gammaKO 
"GIYE-3643" "GIYE-3642" "GIYE-3641" "GIYE-3640" "GIYE-3639" "GIYE-3638" 
     betaKO   epsilonKO 
"GIYE-3637" "GIYE-3636" 


$`EBAC693444-WGS`
$`EBAC693444-WGS`$`TUSNN-2359`
  epsilonKO      betaKO     gammaKO     alphaKO     deltaKO         bKO 
"GSNN-4515" "GSNN-4514" "GSNN-4513" "GSNN-4512" "GSNN-4511" "GSNN-4510" 
        cKO         aKO 
"GSNN-4509" "GSNN-4508" 


$EBIL634500
$EBIL634500$`TUHYX-195`
        aKO         cKO         bKO     deltaKO     alphaKO     gammaKO 
"GHYX-4691" "GHYX-4690" "GHYX-4689" "GHYX-4688" "GHYX-4687" "GHYX-4686" 
     betaKO   epsilonKO 
"GHYX-4685" "GHYX-4684" 


$APAS634456
$APAS634456$`TUL79-296`
 epsilonKO     betaKO    gammaKO    alphaKO    deltaKO 
"GL79-121" "GL79-120" "GL79-119" "GL79-118" "GL79-117" 

$APAS634456$`TUL79-1516`
        aKO         cKO 
"GL79-2467" "GL79-2466" 

$APAS634456$`TUL79-1515`
       bKO2        bKO1 
```

```
"GL79-2465" "GL79-2464"


$ECOL413997
$ECOL413997$`TUCQD-8393`
        aKO         cKO         bKO     deltaKO     alphaKO     gammaKO
"GCQD-3856" "GCQD-3855" "GCQD-3854" "GCQD-3853" "GCQD-3852" "GCQD-3851"
     betaKO   epsilonKO
"GCQD-3850" "GCQD-3849"


$EBLA630626
$EBLA630626$`TULCZ-2103`
  epsilonKO      betaKO     gammaKO     alphaKO     deltaKO         bKO
"GLCZ-3981" "GLCZ-3980" "GLCZ-3979" "GLCZ-3978" "GLCZ-3977" "GLCZ-3976"
        cKO         aKO
"GLCZ-3975" "GLCZ-3974"


$ECOL595496
$ECOL595496$`TUI18-1875`
        aKO         cKO         bKO     deltaKO     alphaKO     gammaKO
"GI18-3560" "GI18-3559" "GI18-3558" "GI18-3557" "GI18-3556" "GI18-3555"
     betaKO   epsilonKO
"GI18-3554" "GI18-3553"


$MGAL1159197
$MGAL1159197$`TULH9-155`
 epsilonKO     betaKO    gammaKO   alphaKO1    deltaKO        bKO        cKO
"GLH9-359" "GLH9-358" "GLH9-357" "GLH9-356" "GLH9-355" "GLH9-354" "GLH9-353"
       aKO
"GLH9-352"

$MGAL1159197$`TULH9-328`
  alphaKO2
"GLH9-732"


$PATR218491
$PATR218491$`TUJNB-1`
        aKO         cKO         bKO     deltaKO     alphaKO     gammaKO
"GJNB-4611" "GJNB-4610" "GJNB-4609" "GJNB-4608" "GJNB-4607" "GJNB-4606"
     betaKO   epsilonKO
"GJNB-4605" "GJNB-4604"


$`ECAS565655-WGS`
$`ECAS565655-WGS`$`TUSMW-1259`
        aKO         cKO         bKO     deltaKO     alphaKO     gammaKO
"GSMW-2382" "GSMW-2381" "GSMW-2380" "GSMW-2379" "GSMW-2378" "GSMW-2377"
     betaKO   epsilonKO
"GSMW-2376" "GSMW-2375"
```

```
$ECOL199310
$ECOL199310$`TUBJ-82788`
  alphaKO   gammaKO    betaKO epsilonKO
  "C4660"   "C4659"   "C4658"   "C4657"

$ECOL199310$`TUBJ-83332`
deltaKO
"C4662"

$ECOL199310$`TUBJ-82789`
    aKO     cKO     bKO
"C4666" "C4665" "C4664"


$ECOL316385
$ECOL316385$`TUJ8B-1990`
        aKO         cKO         bKO     deltaKO     alphaKO     gammaKO
"GJ8B-3760" "GJ8B-3759" "GJ8B-3758" "GJ8B-3757" "GJ8B-3756" "GJ8B-3755"
     betaKO   epsilonKO
"GJ8B-3754" "GJ8B-3753"


$ECOO157
$ECOO157$`TU7E-9691`
      aKO       cKO       bKO   deltaKO   alphaKO   gammaKO    betaKO epsilonKO
   "ATPB"    "ATPE"    "ATPF"    "ATPH"    "ATPA"    "ATPG"    "ATPD"    "ATPC"


$ECOL444450
$ECOL444450$`TUHOB-2785`
        aKO         cKO         bKO     deltaKO     alphaKO     gammaKO
"GHOB-5155" "GHOB-5154" "GHOB-5153" "GHOB-5152" "GHOB-5151" "GHOB-5150"
     betaKO   epsilonKO
"GHOB-5149" "GHOB-5148"


$ECOL574521
$ECOL574521$`TUJAO-2210`
        bKO     deltaKO     alphaKO
"GJAO-4189" "GJAO-4188" "GJAO-4187"

$ECOL574521$`TUJAO-2209`
    gammaKO      betaKO   epsilonKO
"GJAO-4186" "GJAO-4185" "GJAO-4184"

$ECOL574521$`TUJAO-2211`
        cKO
"GJAO-4190"

$ECOL574521$`TUJAO-2212`
        aKO
"GJAO-4191"
```

```
$`APHA1184253-WGS`
$`APHA1184253-WGS`$`TUSFD-797`
    deltaKO     alphaKO
"GSFD-1225" "GSFD-1224"

$`APHA1184253-WGS`$`TUSFD-291`
    betaKO
"GSFD-480"

$`APHA1184253-WGS`$`TUSFD-401`
  gammaKO1
"GSFD-663"

$`APHA1184253-WGS`$`TUSFD-462`
  gammaKO2
"GSFD-748"

$`APHA1184253-WGS`$`TUSFD-290`
 epsilonKO
"GSFD-479"

$`APHA1184253-WGS`$`TUSFD-709`
        aKO         cKO        bKO2        bKO1
"GSFD-1090" "GSFD-1089" "GSFD-1088" "GSFD-1087"


$ECHA205920
$ECHA205920$`TUJNR-77`
   alphaKO    deltaKO
"GJNR-132" "GJNR-131"

$ECHA205920$`TUJNR-346`
 epsilonKO     betaKO
"GJNR-576" "GJNR-575"

$ECHA205920$`TUJNR-386`
   gammaKO
"GJNR-654"

$ECHA205920$`TUJNR-660`
       bKO2        bKO1         cKO         aKO
"GJNR-1092" "GJNR-1091" "GJNR-1090" "GJNR-1089"


$ECOL364106
$ECOL364106$`TUHPQ-2131`
    alphaKO     gammaKO      betaKO   epsilonKO
"GHPQ-4249" "GHPQ-4248" "GHPQ-4247" "GHPQ-4246"

$ECOL364106$`TUHPQ-2681`
    deltaKO
"GHPQ-4251"
```

```
$ECOL364106$`TUHPQ-2132`
       aKO         cKO         bKO
"GHPQ-4255" "GHPQ-4254" "GHPQ-4253"


$MGAL1159201
$MGAL1159201$`TULHA-143`
 epsilonKO     betaKO    gammaKO   alphaKO1    deltaKO        bKO        cKO
"GLHA-340" "GLHA-339" "GLHA-338" "GLHA-337" "GLHA-336" "GLHA-335" "GLHA-334"
      aKO
"GLHA-333"

$MGAL1159201$`TULHA-316`
  alphaKO2
"GLHA-710"


$ECOL316407
$ECOL316407$`TU9PC-27160`
 alphaKO  deltaKO      bKO
"JW3712" "JW3713" "JW3714"

$ECOL316407$`TU9PC-27161`
epsilonKO    betaKO   gammaKO
 "JW3709"  "JW3710"  "JW3711"

$ECOL316407$`TU9PC-27159`
    cKO
"JW3715"

$ECOL316407$`TU9PC-27158`
    aKO
"JW3716"


$ECOL585055
$ECOL585055$`TUJOM-2237`
       aKO         cKO         bKO     deltaKO     alphaKO     gammaKO
"GJOM-4281" "GJOM-4280" "GJOM-4279" "GJOM-4278" "GJOM-4277" "GJOM-4276"
    betaKO   epsilonKO
"GJOM-4275" "GJOM-4274"


$ECOL481805
$ECOL481805$`TUI3G-2285`
  epsilonKO      betaKO     gammaKO     alphaKO     deltaKO         bKO
"GI3G-4380" "GI3G-4379" "GI3G-4378" "GI3G-4377" "GI3G-4376" "GI3G-4375"
       cKO         aKO
"GI3G-4374" "GI3G-4373"


$`ECLO718254-WGS`
$`ECLO718254-WGS`$`TUSMR-210`
      aKO        cKO        bKO    deltaKO    alphaKO    gammaKO     betaKO
```

"GSMR-364" "GSMR-363" "GSMR-362" "GSMR-361" "GSMR-360" "GSMR-359" "GSMR-358"
epsilonKO
"GSMR-357"

$ECOL439855
$ECOL439855$`TUHHB-2225`
aKO cKO bKO deltaKO alphaKO gammaKO
"GHHB-4102" "GHHB-4101" "GHHB-4100" "GHHB-4099" "GHHB-4098" "GHHB-4097"
betaKO epsilonKO
"GHHB-4096" "GHHB-4095"

$ECAN269484
$ECAN269484$`TUI02-55`
alphaKO deltaKO
"GI02-89" "GI02-88"

$ECAN269484$`TUI02-307`
epsilonKO betaKO
"GI02-488" "GI02-487"

$ECAN269484$`TUI02-261`
gammaKO
"GI02-414"

$ECAN269484$`TUI02-569`
bKO2 bKO1 cKO aKO
"GI02-912" "GI02-911" "GI02-910" "GI02-909"

$ASP715451
$ASP715451$`TUHV1-789`
betaKO1 epsilonKO1 aKO1 cKO1 bKO1 alphaKO1
"GHV1-1421" "GHV1-1420" "GHV1-1417" "GHV1-1416" "GHV1-1415" "GHV1-1414"
gammaKO1
"GHV1-1413"

$ASP715451$`TUHV1-2423`
aKO2 cKO2 bKO2 deltaKO alphaKO2 gammaKO2
"GHV1-4437" "GHV1-4436" "GHV1-4435" "GHV1-4434" "GHV1-4433" "GHV1-4432"
betaKO2 epsilonKO2
"GHV1-4431" "GHV1-4430"

$AVIN572477
$AVIN572477$`TUCJK-113`
epsilonKO betaKO gammaKO alphaKO deltaKO bKO cKO aKO
"GCJK-47" "GCJK-46" "GCJK-45" "GCJK-44" "GCJK-43" "GCJK-42" "GCJK-41" "GCJK-40"

$MCIC765698
$MCIC765698$`TUHQ5-911`
epsilonKO betaKO gammaKO alphaKO deltaKO

"GHQ5-1188" "GHQ5-1187" "GHQ5-1186" "GHQ5-1185" "GHQ5-1184"

$MCIC765698$`TUHQ5-3179`
aKO cKO
"GHQ5-5153" "GHQ5-5152"

$MCIC765698$`TUHQ5-3178`
bKO2 bKO1
"GHQ5-5151" "GHQ5-5150"

$`AMAR234826-WGS`
$`AMAR234826-WGS`$`TUSFC-498`
deltaKO alphaKO
"GSFC-863" "GSFC-862"

$`AMAR234826-WGS`$`TUSFC-298`
betaKO epsilonKO
"GSFC-490" "GSFC-489"

$`AMAR234826-WGS`$`TUSFC-281`
gammaKO
"GSFC-458"

$`AMAR234826-WGS`$`TUSFC-474`
bKO2 bKO1 cKO
"GSFC-817" "GSFC-816" "GSFC-815"

$`AMAR234826-WGS`$`TUSFC-614`
aKO
"GSFC-813"

$MHOM347256
$MHOM347256$`TUBZD-14`
epsilonKO betaKO1 gammaKO alphaKO1 deltaKO bKO cKO aKO
"GBZD-29" "GBZD-28" "GBZD-27" "GBZD-26" "GBZD-25" "GBZD-24" "GBZD-23" "GBZD-22"

$MHOM347256$`TUBZD-157`
alphaKO2 betaKO2
"GBZD-343" "GBZD-342"

$AMAC1004786
$AMAC1004786$`TUL7O-156`
aKO cKO bKO deltaKO alphaKO gammaKO
"GL7O-4116" "GL7O-4115" "GL7O-4114" "GL7O-4113" "GL7O-4112" "GL7O-4111"
betaKO epsilonKO
"GL7O-4110" "GL7O-4109"

$ECOLI
$ECOLI$`TU0-6636`
epsilonKO betaKO gammaKO alphaKO deltaKO bKO cKO aKO

```
"EG10100" "EG10101" "EG10104" "EG10098" "EG10105" "EG10103" "EG10102" "EG10099"

$ECOLI$`TU0-6635`
epsilonKO    betaKO   gammaKO   alphaKO   deltaKO       bKO       cKO       aKO
"EG10100" "EG10101" "EG10104" "EG10098" "EG10105" "EG10103" "EG10102" "EG10099"

$ECOLI$TU00243
epsilonKO    betaKO   gammaKO   alphaKO   deltaKO       bKO       cKO       aKO
"EG10100" "EG10101" "EG10104" "EG10098" "EG10105" "EG10103" "EG10102" "EG10099"

$ECOLI$`TU0-42328`
epsilonKO
"EG10100"


$`ECOL1274814-WGS`
$`ECOL1274814-WGS`$`TUSN4-2370`
       aKO         cKO         bKO     deltaKO     alphaKO     gammaKO
"GSN4-4586" "GSN4-4585" "GSN4-4584" "GSN4-4583" "GSN4-4582" "GSN4-4581"
    betaKO   epsilonKO
"GSN4-4580" "GSN4-4579"


$`ECOL1382700-WGS`
$`ECOL1382700-WGS`$`TUSNB-2120`
       bKO     deltaKO     alphaKO     gammaKO      betaKO   epsilonKO
"GSNB-4074" "GSNB-4073" "GSNB-4072" "GSNB-4071" "GSNB-4070" "GSNB-4069"

$`ECOL1382700-WGS`$`TUSNB-2121`
       cKO
"GSNB-4075"

$`ECOL1382700-WGS`$`TUSNB-2122`
       aKO
"GSNB-4076"


$`ECOL1110693-WGS`
$`ECOL1110693-WGS`$`TUSN5-1647`
       bKO     deltaKO     alphaKO     gammaKO      betaKO   epsilonKO
"GSN5-3112" "GSN5-3111" "GSN5-3110" "GSN5-3109" "GSN5-3108" "GSN5-3107"

$`ECOL1110693-WGS`$`TUSN5-1648`
       cKO
"GSN5-3113"

$`ECOL1110693-WGS`$`TUSN5-1649`
       aKO
"GSN5-3114"


$`ECOL1335916-WGS`
$`ECOL1335916-WGS`$`TUSN8-1982`
       aKO         cKO         bKO     deltaKO     alphaKO     gammaKO
```

"GSN8-3883" "GSN8-3882" "GSN8-3881" "GSN8-3880" "GSN8-3879" "GSN8-3878"
betaKO epsilonKO
"GSN8-3877" "GSN8-3876"

$ECOL362663
$ECOL362663$`TUIY5-2108`
aKO cKO bKO deltaKO alphaKO gammaKO
"GIY5-3966" "GIY5-3965" "GIY5-3964" "GIY5-3963" "GIY5-3962" "GIY5-3961"
betaKO epsilonKO
"GIY5-3960" "GIY5-3959"

$ECOL585397
$ECOL585397$`TUJCU-2318`
aKO cKO bKO deltaKO alphaKO gammaKO
"GJCU-4470" "GJCU-4469" "GJCU-4468" "GJCU-4467" "GJCU-4466" "GJCU-4465"
betaKO epsilonKO
"GJCU-4464" "GJCU-4463"

$ECOL585034
$ECOL585034$`TUJ84-2012`
aKO cKO bKO deltaKO alphaKO gammaKO
"GJ84-3950" "GJ84-3949" "GJ84-3948" "GJ84-3947" "GJ84-3946" "GJ84-3945"
betaKO epsilonKO
"GJ84-3944" "GJ84-3943"

$MCAS458233
$MCAS458233$`TUI03-966`
aKO bKO deltaKO alphaKO gammaKO betaKO
"GI03-1812" "GI03-1811" "GI03-1810" "GI03-1809" "GI03-1808" "GI03-1807"
epsilonKO
"GI03-1806"

$MCAS458233$noTU
cKO
NA
