# Supplementary material for: Phylogenetic Profiling of Mitochondrial Proteins and Integration Analysis of Bacterial Transcription Units Suggest Evolution of F1Fo ATP Synthase from Multiple Modules
Source: J Mol Evol. 2017 Nov 24;85(5):219–33. doi: 10.1007/s00239-017-9819-3 (PMC5709465; doi:10.1007/s00239-017-9819-3)
Supplement: Supplementary file 3 — Supplementary material 3 (DOCX 38 KB) [file 239_2017_9819_MOESM3_ESM.docx]

**Supple. Figure 1: A)** The distribution of detected human F1Fo ATP synthase homologs by the BLAST method with different E-value threshold. The homologs from F-type, V-type, and A-type ATPase are determined by KEGG Ontology (KO) database. The homologs type “other” represents homologs with no KO annotation or the ones belong to other KO terms. **B)** The percentage of A/B subunits in the detected homologs that are from V/A-type ATPase.

**Supple. Figure 2:** **A–C)** The distance trees of mitochondria F1Fo ATP synthase subunits with different thresholds of BLASTP E-value (0.1, 0.01, and 0.0001 for A, B and C, respectively).

**Supple. Figure 3:** **A)** and **B**) The PCA plot of mitochondrial F1Fo ATP synthase subunits with group numbers set to 2 and 3, respectively.

**Supple. Figure 4:** Frequency of transcription unit modules of raw transcription unit data retrieved from the BioCyc database.

**Supple. Figure 5: A–C)** Examples of δ/b Rosetta Stone and single δ and b from *Gordonia bronchialis* (KEGG species ID “gbr”), *Mycobacterium smegmatis* (“msm”), and *Escherichia coli* (“eco”), respectively. The blue bars “pf:OSCP” and “pf:ATP-synt_B” represent the conserved motifs of δ and b subunits, respectively.

**Supple. Figure 6: A–D)** The reconstructed phylogenetic tree combined with predicted transcription units containing F1Fo ATP synthase subunits in Alphaproteobacteria, Clostridia, Gammaproteobacteria, and Tenericutes, respectively. Species in one genus are marked with the same color in bars and tree branches. **E)** and **F**) The representative architectures of chimera fusions “β-ε-a-c-b-α-γ” and “β-α”, respectively.

**Supple. Table 1:** Species list used for generating the phylogenetic profiles.

**Supple. Table 2:**  Raw data of the phylogenetic profiles of the human mitochondrial proteins using the set of 758 species.

**Supple. Table 3:** Hierarchical clusters from the phylogenetic profiles of the F1Fo ATP synthase phylogenetic profiles using the entire set of 758 species (with human genome as the reference).

**Supple. Table 4:** Raw data phylogenetic profiles of the mitochondrial proteins with 490 randomly selected species.

**Supple. Table 5:** Hierarchical clusters from phylogenetic profiles with 490 randomly selected species.

**Supple. Table 6:** The bacterial species list used for the transcription unit analysis combining with transcription units modules and chimera fusions in each species.

**Supple. Table 7:** The list of bacterial species containing the δ/b Rosetta Stone.

**Supple. Table 8:** Summary table of the dominant patterns of transcription units in bacterial classes and phyla.
