# Supplementary material for: Phylogenetic Profiling of Mitochondrial Proteins and Integration Analysis of Bacterial Transcription Units Suggest Evolution of F1Fo ATP Synthase from Multiple Modules
Source: J Mol Evol. 2017 Nov 24;85(5):219–33. doi: 10.1007/s00239-017-9819-3 (PMC5709465; doi:10.1007/s00239-017-9819-3)
Supplement: Supplementary file 4 — Supplementary material 4 (PDF 2284 KB) [file 239_2017_9819_MOESM4_ESM.pdf]

A

B

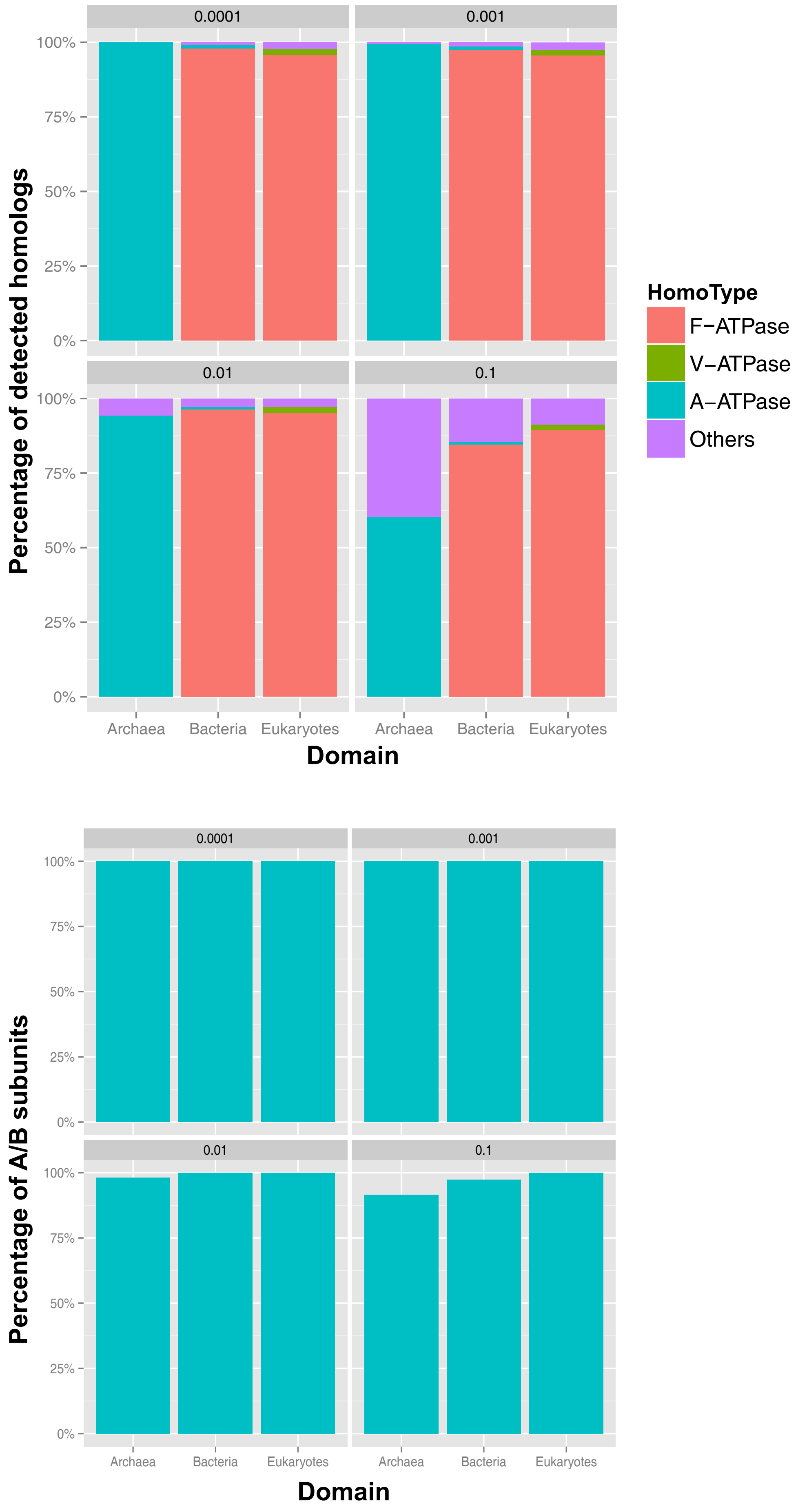

Supplementary Figure 1

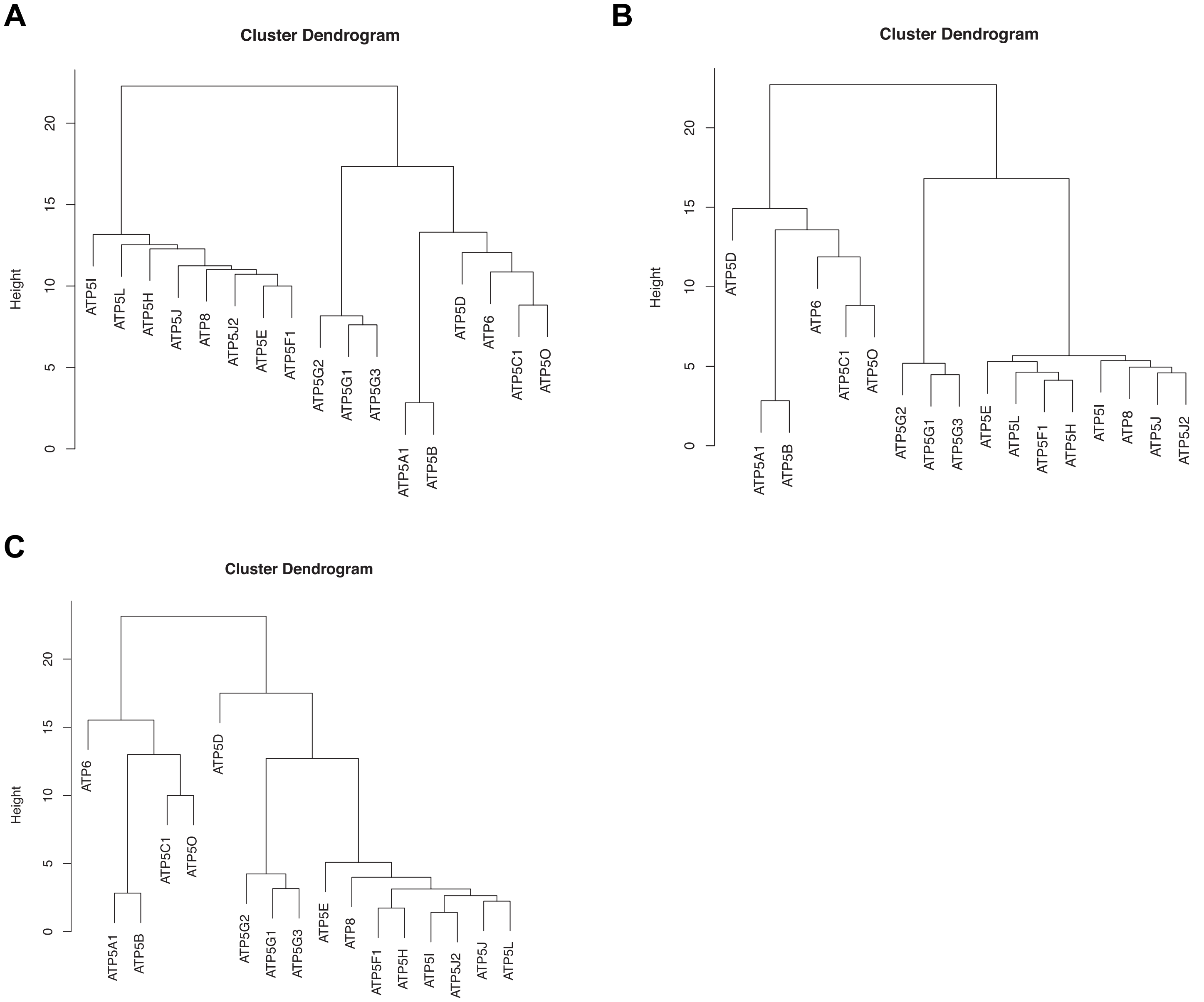

Supplementary Figure 2

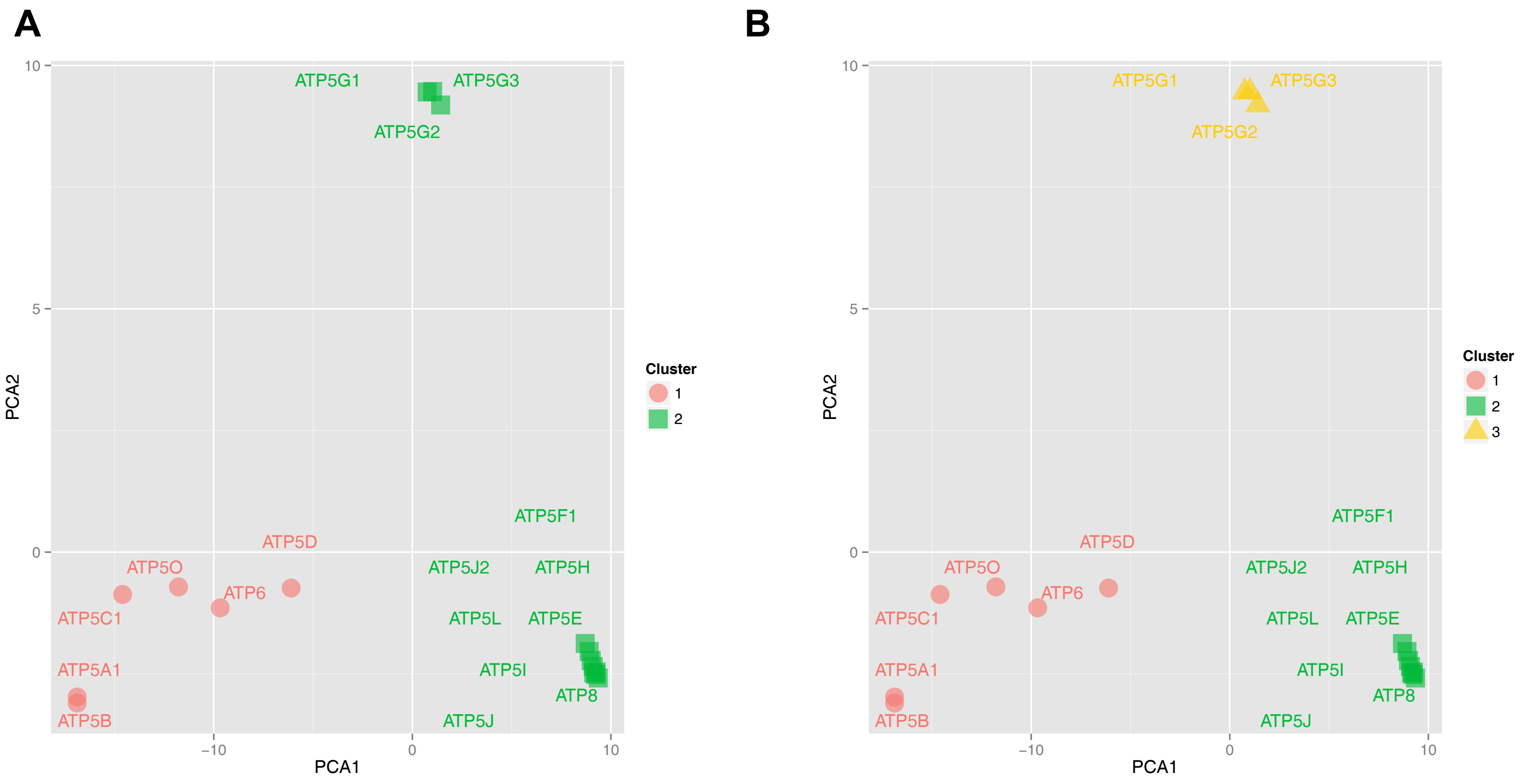

Supplementary Figure 3

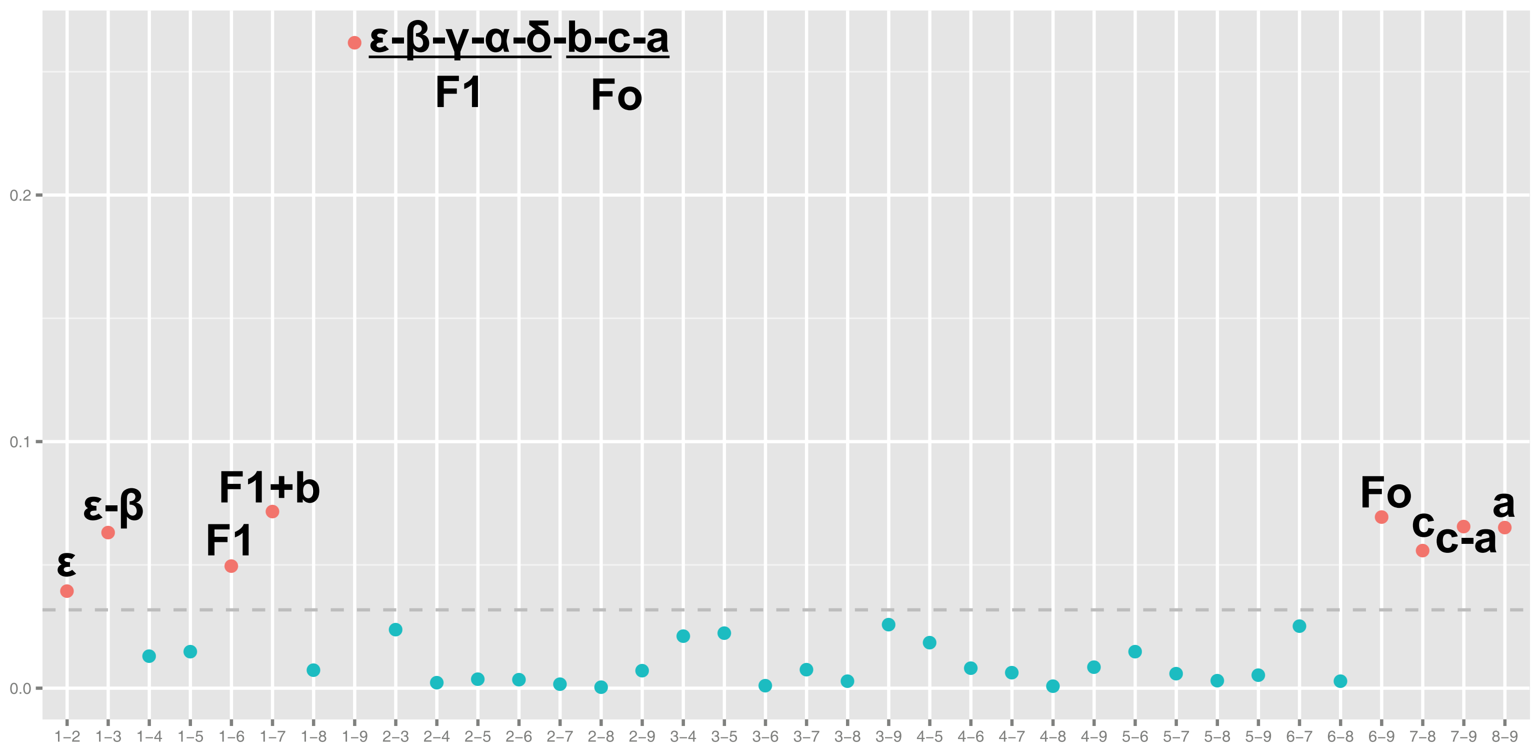

Supplementary Figure 4

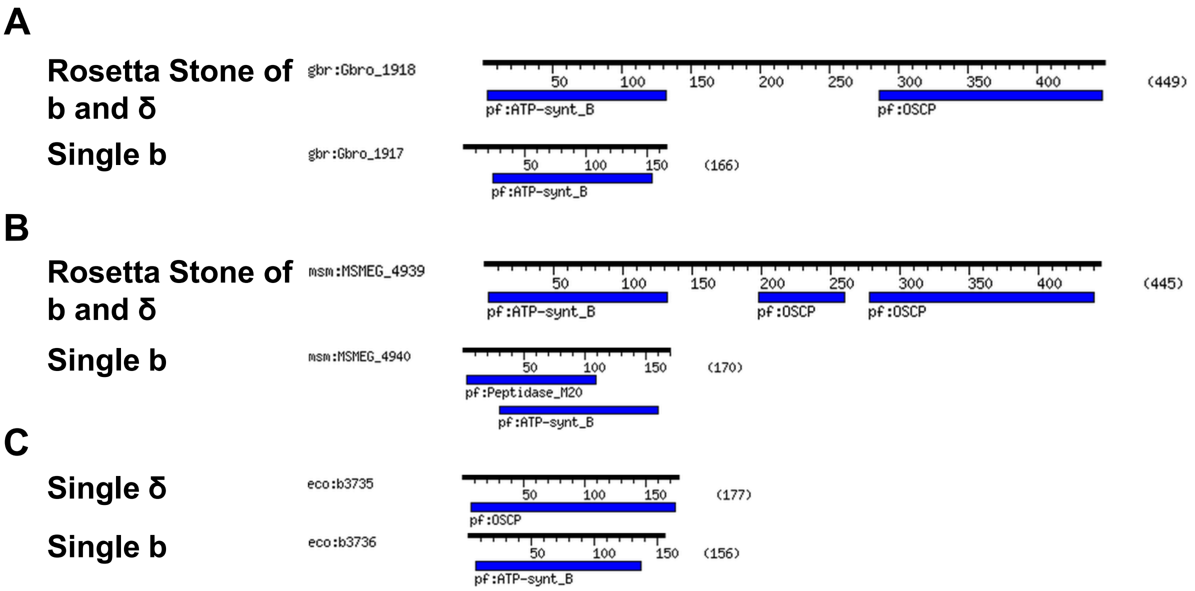

Supplementary Figure 5

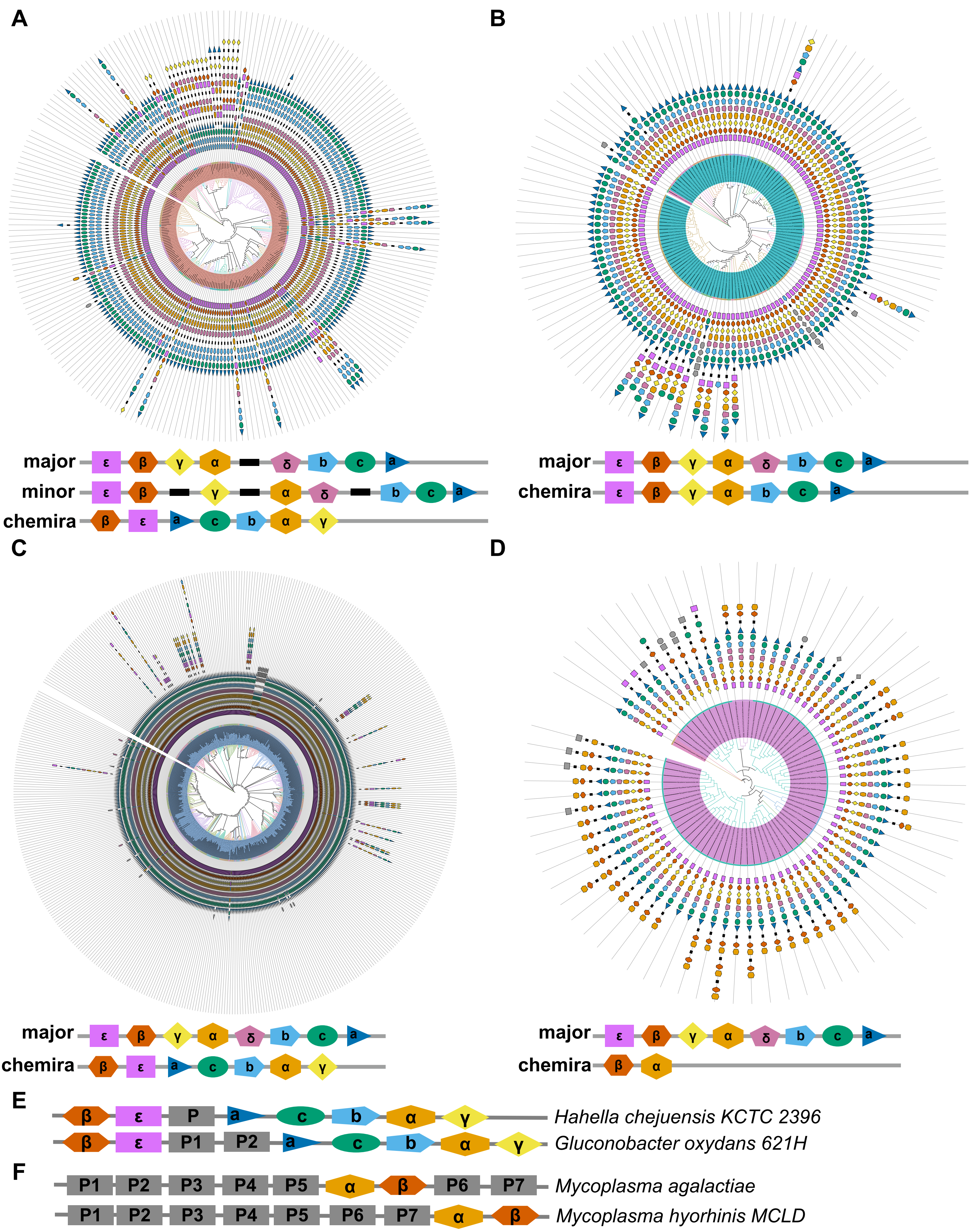

Supplementary Figure 6
